# Amino acids biosynthesis and nitrogen assimilation pathways: a great genomic deletion during eukaryotes evolution

**DOI:** 10.1186/1471-2164-12-S4-S2

**Published:** 2011-12-22

**Authors:** RLM Guedes, F Prosdocimi, GR Fernandes, LK Moura, HAL Ribeiro, JM Ortega

**Affiliations:** 1 Departamento de Bioquímica e Imunologia, Instituto de Ciências Biológicas, Universidade Federal de Minas Gerais, Belo Horizonte, 31270-901, MG, Brazil; 2 Programa de Pós-Graduação em Ciências Genômicas e Biotecnologia, Universidade Católica de Brasilia, Brasilia, 70790-160, DF, Brazil; 3 Instituto de Bioquímica Médica, Universidade Federal do Rio de Janeiro, Rio de Janeiro, 21941-902, RJ, Brazil

## Abstract

**Background:**

Besides being building blocks for proteins, amino acids are also key metabolic intermediates in living cells. Surprisingly a variety of organisms are incapable of synthesizing some of them, thus named Essential Amino Acids (EAAs). How certain ancestral organisms successfully competed for survival after losing key genes involved in amino acids anabolism remains an open question. Comparative genomics searches on current protein databases including sequences from both complete and incomplete genomes among diverse taxonomic groups help us to understand amino acids auxotrophy distribution.

**Results:**

Here, we applied a methodology based on clustering of homologous genes to seed sequences from autotrophic organisms *Saccharomyces cerevisiae* (yeast) and *Arabidopsis thaliana* (plant). Thus we depict evidences of presence/absence of EAA biosynthetic and nitrogen assimilation enzymes at phyla level. Results show broad loss of the phenotype of EAAs biosynthesis in several groups of eukaryotes, followed by multiple secondary gene losses. A subsequent inability for nitrogen assimilation is observed in derived metazoans.

**Conclusions:**

A Great Deletion model is proposed here as a broad phenomenon generating the phenotype of amino acids essentiality followed, in metazoans, by organic nitrogen dependency. This phenomenon is probably associated to a relaxed selective pressure conferred by heterotrophy and, taking advantage of available homologous clustering tools, a complete and updated picture of it is provided.

## Background

Creation and analysis of groups of orthologous genes have been widely used for gene function prediction, evolutionary and divergence time studies [1]. Moreover, orthology is also a valuable source for evolutionary comprehension of pathways through phylogenetic analysis. In respect to a central issue on cellular metabolism, the order of appearance for universal cellular metabolisms was estimated by Cunchillos and Lecointre [2,3], with amino acid catabolism and anabolism being respectively the first and second pathways to appear, even earlier than glycolysis and gluconeogenesis. The amino acids biosynthesis, rather than linear and universal series of reactions with homologues occurring in different organisms, sometimes relies on alternative pathways, as shown by Hernández-Montes et al. [4]. Moreover, gene loss and pathway depletion, important events in genome evolution, can be inferred from the orthologous groups through comparative genomics. Today, a vast amount of information is provided by intensive genome sequencing, and the efforts of grouping homologous genes had reached great standards.

Amino acid anabolism is responsible for about 20% of the energy that cells spend on protein synthesis [5,6]. The nutritional requirements of essential amino acids and nitrogen are of striking importance and they have been estimated as ~22mg/kg of EAAs and 3mg/kg of N in human body [7,8]. More recent approaches for dietary requirement calculations, using amino acid oxidation as an indicator, reveal that the requirement is over five fold what the classical approaches indicated, and the requirement has now been determined for each of the nine human EAAs [9]. It is of general understanding that plant, as well as fungi, synthesize all amino acids required for protein synthesis and that evolutionary processes culminated in human inability to synthesize nine amino acids (histidine, phenylalanine, tryptophan, valine, isoleucine, leucine, lysine, methionine and threonine), thus called essential amino acids (EAAs), which must be obtained through diet. Amino acids also constitute our source of organic nitrogen. There have been few attempts to understand why some amino acids have become essential. However, genome deletion events have happened in the past and many organisms have lost a number of important enzymes necessary for *de novo* biosynthetic pathways. Hitherto, the pattern of loss versus retention for amino acids biosynthetic pathways was analyzed for a few protists and metazoans by Payne and Loomis [10]. They verified that the set of essential amino acids is the same in animals and protists. Curiously, most of the retained amino acids are intermediates in secondary pathways like purine ring biosynthesis and nitrogen metabolism.

An overview for the presence/absence of the enzymes which compose the amino acid biosynthetic pathways, among distinct phyla in the tree of life, could be accomplished with (i) rich protein databases such as the UniProt Knowledgebase (UniProtKB) [11] comprising over 10 million full-length sequences and (ii) the current initiatives to group these proteins by evolutionary relatedness - called homologues - such as COG-Cluster of Orthologous Groups [12] and KEGG Orthology [13]. Unfortunately these initiatives consider only proteins derived from complete genomes and thus a large amount of information is currently lost, with over 6 million remaining full-length proteins that belong to organisms with still incomplete genomes.

Here, we applied a methodology that takes into account all available protein information to depict, at phyla level, the EAA biosynthetic and nitrogen assimilation enzymes scenarios to inspect how and when amino acid auxotrophy has first appeared along evolution.

A Great Genomic Deletion model is proposed to explain the phenotypic inability to synthesize amino acids that appears independently in distinct phylogenetically distant clades of eukaryotes. Such events should be followed by subsequent steps of gene loss due to relaxed selective pressure in already incomplete pathways, leading to an eventual loss of all genes for a particular biosynthesis pathway in some clades. Accordingly, in metazoans but Cnidaria, dependence on organic nitrogen accompanies the evolution of heterotrophy, thus organisms become dependent even on NEAA for supplying their nitrogen requirements.

## Results

### Clustering homologues of amino acid biosynthetic enzymes

**Figure 1:**
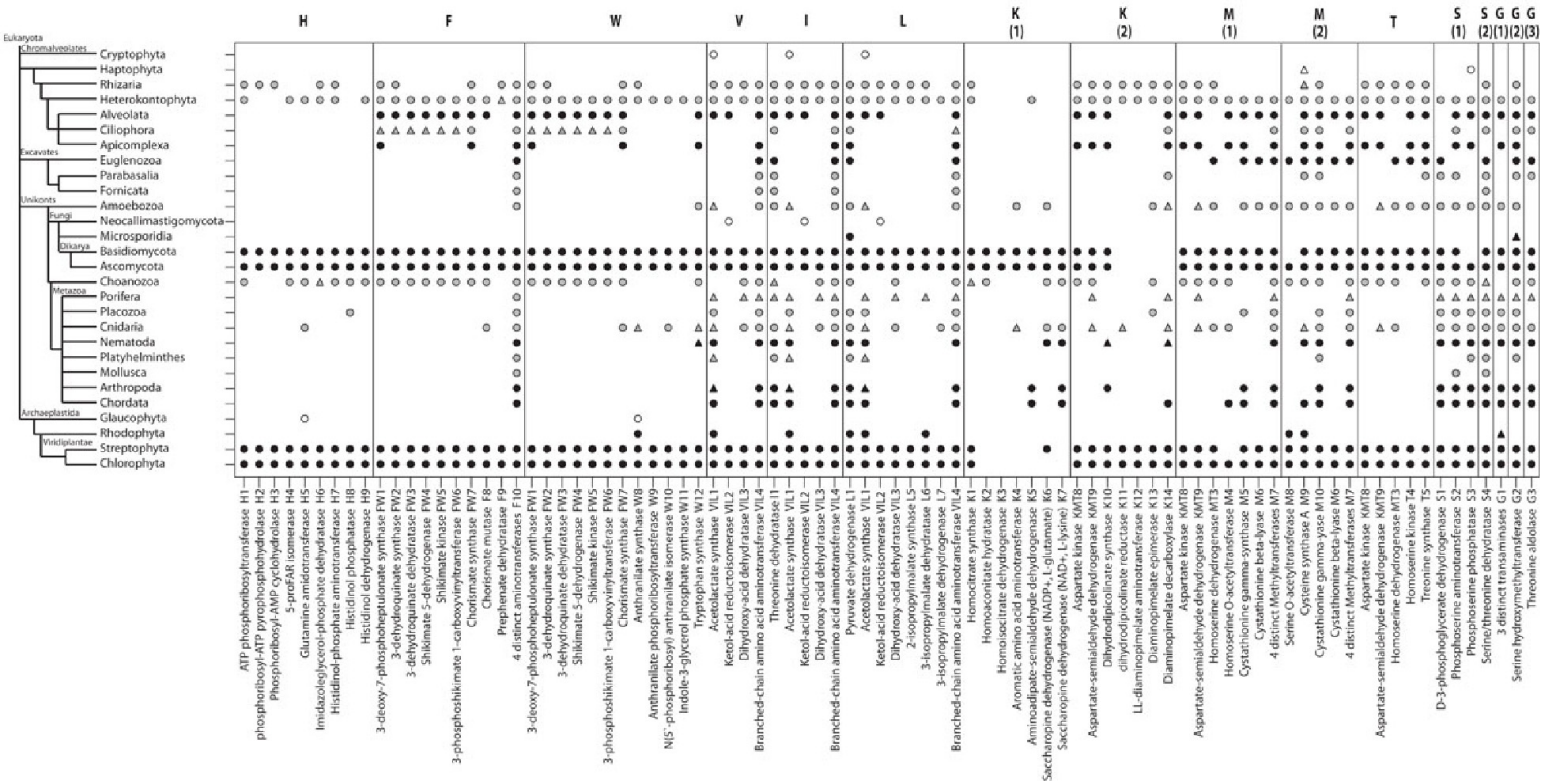
**Essential amino acid anabolic pathways.** Schematic representation for presence/absence of anabolic enzymes for nine essential amino acids and the non-essential amino acids serine and glycine. Eukaryotic taxonomic tree displayed at phyla level. Circles represent detection of complete proteins and triangles detection of complete and fragmented proteins. Black: phyla containing complete genomes; Grey: at most organisms with draft genomes; White: phyla with no complete or draft genomes. *Saccharomyces cerevisiae* (Ascomycota) and *Arabidopsis thaliana* (Streptophyta) were used as seeds. The 4 distinct aminotransferases in phenylalanine pathway are: (i) aspartate aminotransferase (ii) histidinol-phosphate aminotransferase (iii) aromatic amino acid aminotransferase (iv) tyrosine aminotransferase. The 4 distinct methyltransferases in methionine pathway are: (i) 5-methyltetrahydropteroyltriglutamate--homocysteine methyltransferase (ii) homocysteine S-methyltransferase (iii) betaine-homocysteine methyltransferase (iv) 5-methyltetrahydrofolate--homocysteine methyltransferase. The 3 distinct transaminases in glycine pathway are: alanine-glyoxylate transaminase, serine-glyoxylate transaminase and serine-pyruvate transaminase.

To determine the distribution of amino acid biosynthetic enzymes, a homologue clustering process was developed to allow the use of both complete and incomplete genomes [14,15]. The procedure starts with Seed Linkage software [14] that clusters cognate proteins from multiple organisms beginning with a single seed sequence through connectivity saturation with it. Since basal eukaryotes such as plants and fungi are autotrophic, sequences coding for all the enzymes used in the biosynthesis of EAAs from the plant *Arabidopsis thaliana* and the fungus *Saccharomyces cerevisiae* were manually inspected using KEGG Pathway and used as seeds to search for homologues. Moreover, our group has been developing a procedure to enrich secondary databases such as COG [12] and KEGG Orthology (to be published) with UniRef50 clusters [16] available from UniProt, therefore allowing the inclusion of data from incompletely sequenced genomes. Additional file 1: Sequences and genome status distribution reflects the abundance of proteins derived from incomplete genomes and evidences the importance of their inclusion. In this work we took advantage of a home-built UniRef50 Enriched KEGG Orthology database (UEKO) to additionally cluster sequences with the seed sequences mentioned above. Since these searches recruit sequences from diverse clades, which may or may not contain organisms with completely sequenced genomes, we represented this information in Figure 1 as: (a) black filled circles for phyla containing complete genomes; (b) grey filled circles comprise clades with at least one draft genome available, but no complete genome, and (c) empty circles represent phyla with no complete nor draft genomes. Protein fragments are not included in the search for homologues because they may represent partial sequenced full length proteins at mRNA level or incompletely modeled from genome. Moreover since some full length proteins might have not been captured in databases due to high sequence divergence, a second search round used UniProt to query all clustered sequences. This step also captures partial sequences (entries labeled as fragments in UniProt) which were approved by the coverage filtering applied (see Methods for details). These additional significant hits are represented by triangles in Figure 1. Furthermore, enzymes required for the biosynthesis of the indicated amino acids are ordered in the anabolic pathway from left to right. All pathways refer to EAAs biosynthesis except serine and glycine (the rightmost ones) used as experimental controls. Serine is represented with two alternative pathways observed in human and other eukaryotes: S(1), from 3P-D-glycerate; and S(2), from pyruvate. Glycine is also represented by two pathways: G(1) and G(2), both coming from serine; and G(3), coming from threonine. As expected, serine and glycine biosynthesis were found to be potentially proficient in almost all phyla. This control supports the searching mechanism and attest for the efficacy of methods applied. A few exceptions were observed and deserve comments: (i) Serine biosynthetic pathways was found to be absent in Rhodophyta, although the complete genome of *Cyanidioschyzon merolae* is available. We manually inspected this result with regular BLAST searches and did not find additional evidence, although a translation of partial CDS was obtained for glycine biosynthetic enzyme G1 (Figure 1, triangle); (ii) Serine biosynthesis seems absent in Apicomplexa as well, a clade comprising two *Plasmodium* complete genomes lacking enzymes S1 and S4; (iii) Considering the animals, besides being able to find serine biosynthetic enzymes, we fail to support the NEAA character of glycine for Mollusca. However, evidences could be obtained for ancient organisms such as Placozoa and Porifera. For the Microsporidia *E. cuniculi*, an obligatory intracellular parasitic fungus with complete genome, it has been reported that “the repertoire for the biosynthesis of amino acids is restricted to asparagines synthetase and serine hydroxymethyltransferase genes”, then serine was known as an EAA [17]. Thus, absence of evidence may not guarantee the absence of the gene. However, out of 28 phyla, discarding both the four clades with no genome project or in progress (open circles) and the ones with complete genome (filled symbols), we could not provide evidence of glycine biosynthesis for two phyla (Fornicata and Mollusca). However evidence for serine has been provided in all of them.

Data presented in Figure 1 clearly depicts the presence of complete biosynthetic pathways for EAAs in both plants (Chlorophyta and Streptophyta) and fungi (Ascomycota and Basidiomycota), as stated above. In previous work we hypothesized that a great event of genome deletion on which many of the intermediate enzymes for biosynthetic pathways for amino acids have vanished, ended up affecting the usage of EAAs in chordate proteomes [18,19]. In 2006, Payne and Loomis [10] using pFam protein signatures reported that protists and animals share essentiality for the nine amino acids. Here we provide a broader analysis covering all genomes available today and trying to map how and when the Great Genomic Deletion has happened. Evidence was found suggesting that this loss of capability to synthesize EAAs is conspicuous at the base of metazoan evolution, simultaneously affecting the complete set of EAAs. The phenomenon is characterized as an initial phenotypic deficiency, observed in Choanozoa, followed by multiple secondary gene losses. Accordingly, some enzymes found in Chordata such as K14, M4 and M9 are missing in Arthropoda. Remarkably, some components such as VIL1 and M7 are maintained in most metazoan clades, despite of pathway loss.

Actually, a Great Deletion causing concurrent phenotypic loss of amino acid biosynthesis capability affects both metazoan and non-metazoan eukaryotes. Several clades containing complete genomes (black filled symbols) such as Rhodophyta, Euglenozoa and Apicomplexa, show similar EAAs pattern. Moreover, some evidence is provided suggesting the absence of complete pathways in the non-Dikarya Fungi Microsporidia and Neocallimastigomycota. This gives support to separate events of Great Genomic Deletion for the origin of EAAs auxotrophy in at least three other branches. Similarly to Choanozoa, clades such as Heterokontophyta and Rhizaria present various enzymes and some complete pathways. Evidences of complete pathways for all EAAs but histidine (H) were obtained in Heterokontophyta. Valine (V), isoleucine (I), lysine (K) and threonine (T) are potentially synthesized in Rhizaria as well as methionine (M) in Euglenozoa and Amoebozoa. However it is possible that other EAAs may also be synthesized in some of these clades. The anabolic capabilities suggested by the current data might be underestimated because we have only draft genomes available for most of these organisms. The Choanozoa clade contains only draft genomes. Though we observed more enzymes than in metazoan clades, a final picture of Choanozoan phenylalanine biosynthesis, for example, might require completion of genome sequencing. Further gene loss occurs during metazoan evolution; however, for Placozoa, Porifera and Cnidaria, the Great Genomic Deletion seems to be well established. Since the first available sponge genome is still an ongoing project and its proteins are not yet deposited in UniProt, we manually inspected the deduced proteome using regular BLAST alignments (see Methods) and evidenced auxotrophy for all nine EAAs. The same simple approach was applied to all phyla (Figure 1, triangles). Other clades that do not present any enzymes were omitted from Figure 1, such as Apusozoa and Jakobida.

### Lysine biosynthesis

**Figure 2:**
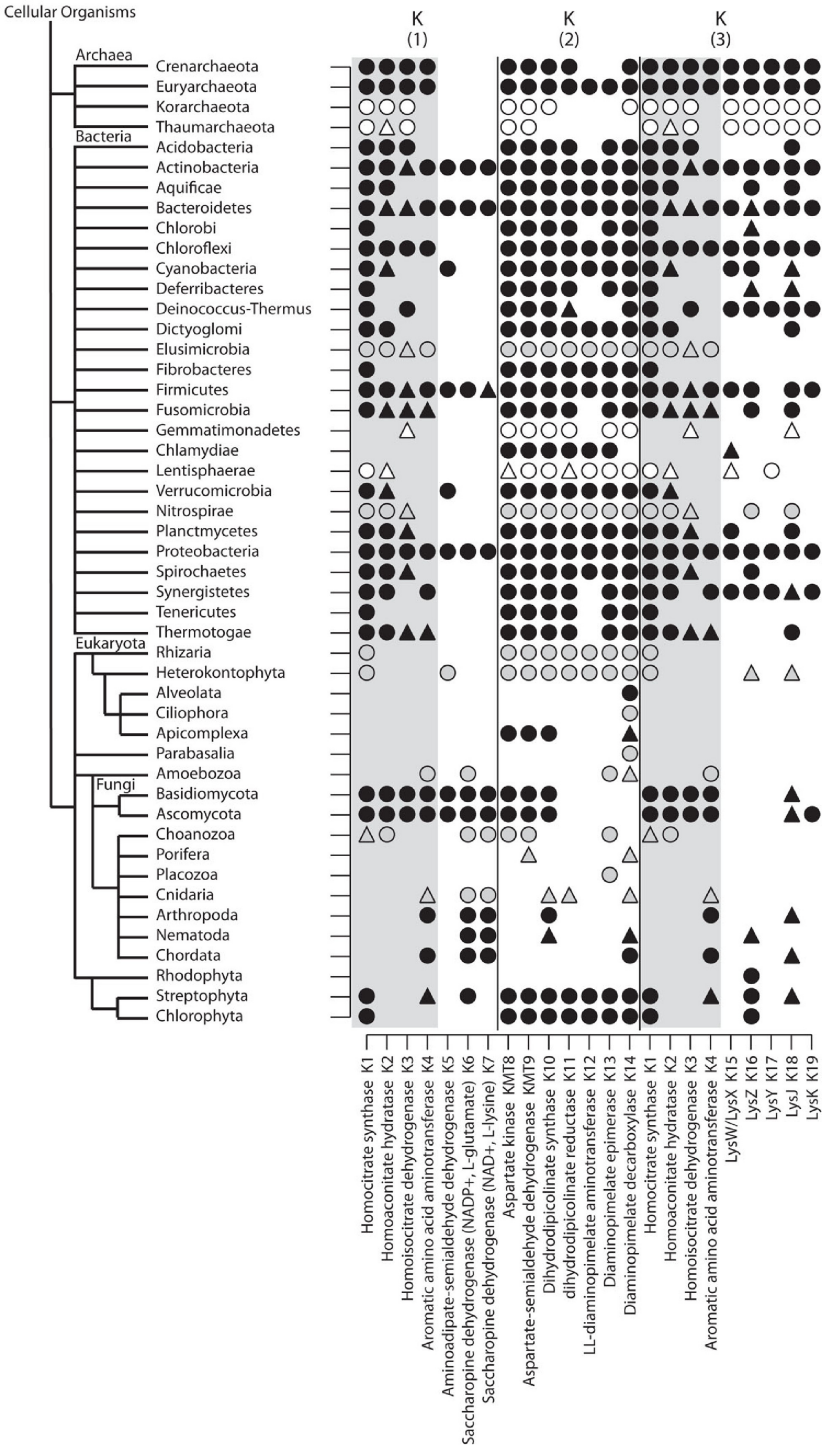
**Lysine anabolic pathways.** Schematic representation for presence/absence of enzymes involved in lysine biosynthesis. K(1) represents Fungi α-aminoadipate (AAA) pathway; K(2) bacteria, plants, and algae diaminopimelate (DAP) pathway; K(3) archaea α-aminoadipate (AAA) variant pathway. Taxonomic tree displayed at phyla level. Circles represent detection of complete proteins and triangles detection of complete and fragmented proteins. Colors are as for Figure 1. *Saccharomyces cerevisiae* (Ascomycota), *Arabidopsis thaliana* (Streptophyta) and *Pyrococcus horikoshii* (Euryarchaeota) were used as seeds.

Inspection of Figure 1 depicts a remarkable difference on lysine (K) biosynthesis pathways present in fungi and plants. Since the occurrence of an α-aminoadipate (AAA) pathway K(1) in Fungi [20] as opposite to a diaminopimelate (DAP) pathway K(2) known to be present in plants, algae and bacteria [21,22] has already been reported, we set up to depict the complete scenario for K biosynthesis including prokaryotes (Figure 2). A third pathway K(3) preferentially used by Archaea but also reported to exist in bacterial groups [23] was also considered, therefore sequences from the *Pyrococcus horikoshii* archaea were also used as seed for homologue sequence clustering. Data supports the view that the K(2) pathway, found to be complete in plants, is often present in prokaryotic clades of bacteria and archaea, in agreement with previous findings [21,22]. Curiously, nine bacterial clades (Acidobacteria, Chlorobi, Deferribacteres, Deinococcus-Thermus, Fusobacteria, Chlamydiae, Synergistetes, Tenericutes and Thermotogae) -- all of which contain complete genomes -- do not present K12 enzyme, but there are three other alternative subsets of enzymes present in prokaryotes that could circumvent this step in lysine biosynthesis. Chlamydiae may represent an evidence of amino acid essentiality extended to prokaryotes, since diaminopimelate decarboxylase (K14) is absent and there are no known alternatives to this reaction. The set of enzymes responsible for the K(3) pathway, was found to occur in prokaryotes, and it is complete in the archaeal clades Crenarchaeota and Euryarcheota, as well as in the bacterial clades Chloroflexi and Proteobacteria, and probably in Actinobacteria and Bacteroidetes. Remarkably, the first four enzymes that constitute this pathway are coincident with the K(1) pathway (indicated by gray shading). The complete K(1) pathway occurs in Proteobacteria (and possibly in Actinobacteria, Bacteroidetes and Firmicutes, as evidenced by regular BLAST) and fungi. Thus, it is tempting to assume that a variant synthesis of K occurred in Archaea and, being modified in one of the four bacterial phyla above (with the addition of three enzymes: aminoadipate-semialdehyde dehydrogenase, saccharopine dehydrogenase NADP+ and saccharopine dehydrogenase NAD+), ended up constituting the fungi-occurring K biosynthetic pathway. The eukaryotic clades Rhizaria and Heterokontophyta, which present the K(2) pathway, appear to group with plants.

### Nitrogen auxotrophy

**Figure 3:**
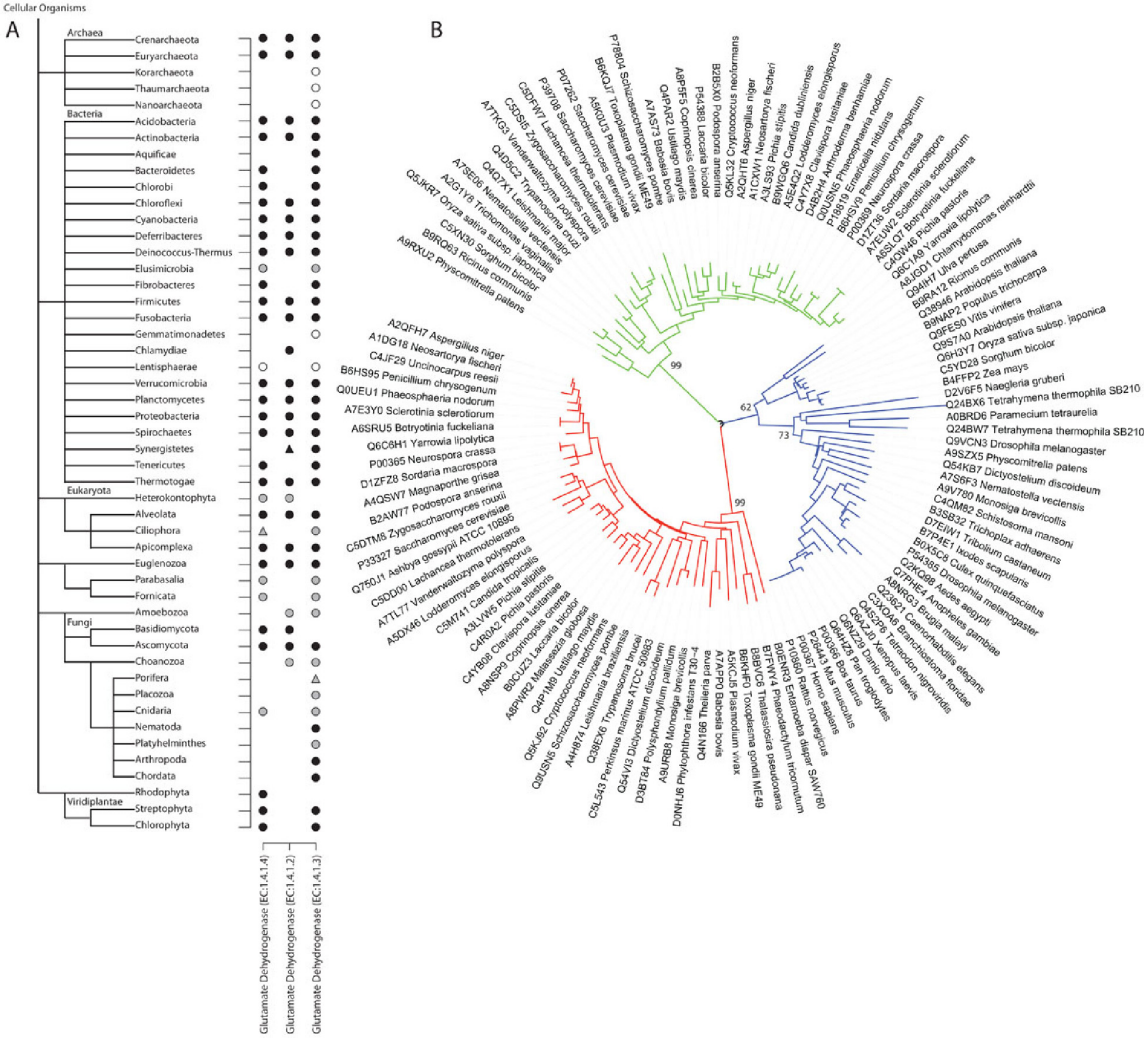
**Glutamate dehydrogenases.** Schematic representation for presence/absence of glutamate dehydrogenases. A: Left column: assimilative *GDH1* and *GDH3* from *Saccharomyces cerevisiae* and putative *GDH* from *Arabdopsis thaliana*; Central column: catabolic *GDH2* from *Saccharomyces cerevisiae*; Right column: catabolic *GDH1*, *GDH2* and *GDH3* from *Arabdopsis thaliana*. Taxonomic tree displayed at phyla level. Circles represent detection of complete proteins and triangles detection of complete and fragmented proteins. Colors are as for Figure 1. *Saccharomyces cerevisiae* (Ascomycota) and *Arabidopsis thaliana* (Streptophyta) were used as seeds. B: Phylogenetic tree with eukaryotic sequences from glutamate dehydrogenase isoforms. Green branches: EC1.4.1.4; Red branches: EC:1.4.1.2; Blue branches: EC:1.4.1.3.

Consumption of amino acids is an important route for nitrogen assimilation in other biological compounds for heterotrophic organisms, such as those comprised by some of the clades shown in Figure 1 (e.g. Chordata). Assimilation of free ammonium in eukaryotes is done by a cytoplasmatic reaction catalyzed by glutamate dehydrogenase (EC:1.4.1.4) which incorporates ammonium into alpha-ketoglutarate yielding glutamate, using electrons from a reduced cytoplasmatic co-enzyme NADPH. Two isoforms are present in fungi and one in plants, the latter having the additional option to not only assimilate nitrogen, but also to fixate it, often with the association of nitrogen-fixating bacteria. Thus, to investigate if the Great Genomic Deletion of biosynthetic enzymes for EAAs co-occurred with the heterotrophy for nitrogen, we generated clusters of the assimilative isoforms (EC:1.4.1.4) and, as a control, the mitochondrial enzymes (EC:1.4.1.2) which tend to operate in the reverse direction, i.e. glutamate degradation, by oxidizing it and delivering ammonium, loading electrons in NAD+ co-enzyme. In yeast, the cytoplasmic assimilative isoforms are named *GDH1* and *GDH3*, and the catabolic (mitochondrial) is known as *GDH2*. *Arabidopsis thaliana* proteins were also used as seed together with the *Saccharomyces cerevisiae* sequences: one known as putative *GDH* which grouped with the fungi assimilative ones, and three catabolic *GDHs*, that grouped with the human mitochondrial *GLUD1*, though not with the yeast catabolic *GHD2*. Results are shown in Figure 3A. The left column shows a cluster that groups assimilative isoforms with the two from yeast and the putative *GDH* from *A. thaliana*. The catabolic mitochondrial isoforms from yeast (central column) and plant (right column) formed two independent clusters. In metazoan organisms, an assimilative enzyme was found in the basal group Cnidaria, all others being dependent on amino acid consumption to build nitrogenated compounds such as DNA, Porifera included. Assimilative isoforms were also lacking in Choanozoa although complete genomes are unavailable. The same was observed for Placozoa. Comparing these results with those shown in Figure 1, it is remarkable that Choanozoa, while still registering many amino acid biosynthetic enzymes (37 out of 61, redundancy eliminated) shows a simultaneous deletion in both EAAs biosynthesis and nitrogen assimilation. It is also apparent that the Great Genomic Deletion attains its almost final broad distribution in Cnidaria, which may be the last metazoan clade still capable to assimilate nitrogen from free ammonium. Therefore a few biosynthetic enzymes remain, in this clade and other Metazoa, probably by connective functions in metabolism (e.g. EC: 1.2.1.31 aminoadipate-semialdehyde dehydrogenase K5 and EC: 1.5.1.7 saccharopine dehydrogenase K7 also participates in the lysine degradation pathway). We have also observed that mammalian *GDH* (*GLUD1*) presents a specialized allosteric control [24] which might have turned the enzyme toward glutamate catabolism rather than anabolism. Such control was first observed in Ciliophora [25] and it is thought to have been transferred by lateral gene transfer to the metazoan ancestor [26]. To confirm the grouping in three clusters of enzymes with so similar activities, Figure 3B shows a phylogenetic tree built with eukaryotic glutamate dehydrogenase sequences, which clustered the isoforms in total accordance with data shown in Figure 3A.

The non-Metazoa eukaryotes with complete genomes, such as Alveolata, Apicomplexa and Euglenozoa, lack EAA biosynthetic enzymes (Figure 1) but keep the capability of nitrogen assimilation (Figure 3). Fornicata and Parabasalia, although represented only by draft genomes, have shown to contain the nitrogen assimilation enzyme even if they appear to be auxotrophic for all EAAs. Lacking detection of any isoform of glutamate dehydrogenase and with available draft genomes is Rhizaria (no complete genomes available), which still presents some EAA biosynthetic capability. It is possible that the dependency of organic nitrogen has been attained earlier in Rhizaria, although complete sequencing is required for a sound conclusion. In general, data support a tendency for nitrogen heterotrophy succeeding the amino acid essentiality. In Rhodophyta, a clade containing complete genomes sequenced, surprisingly no catabolic homologues were found; however a sequence that clusters with the assimilative isoforms has been found.

We also investigated nitrogen assimilation in prokaryotes. Homologues of assimilative enzymes are present and detected by our clustering procedure, but besides finding homologues of the catabolic seeds in bacterial clades, assimilative enzymes were not found in Aquificae, Chlamydiae and Synergistetes, all of them containing complete genomes available. This absence is consistent with the lysine auxotrophy suggested in Chlamydiae (Figure 2) and support the idea that EAA auxotrophy is associated with the lack of nitrogen assimilation even in the prokaryotic clades. It is hard to infer differential enzymatic activity in prokaryotes, since the annotated sequences available often report mixed use of coenzyme, either NADPH or NAD, although the homologous tools had grouped them distinctively. If the homology is related to function, it may indicate that these organisms also demand the consumption of NEAA to constitute a source of organic nitrogen. The presented scenario suggests that the loss of nitrogen assimilation forcing consumption of NEAA shortly succeeds the Great Genomic Deletion of EAA biosynthetic enzymes in metazoans. If this hypothesis is true, the Cnidaria would be an exception.

### EAA biosynthetic enzymes maintained

**Figure 4:**
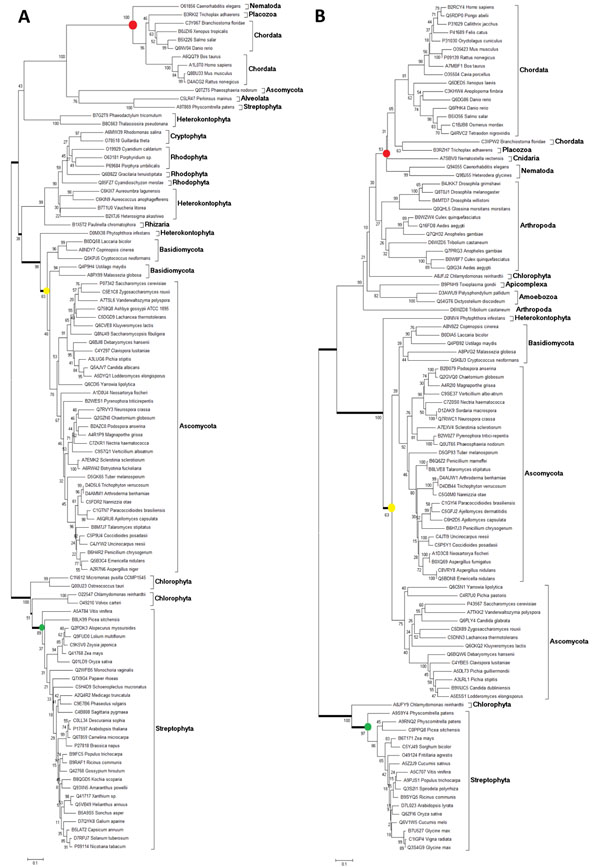
**Phylogenetic analyses for EAA and NEAA enzymes.** Phylogenetic trees for (A) acetolactate synthase (VIL1 code in Figure 1), an enzyme for EAA valine, isoleucine and leucine biosynthesis and (B) a group of alanine-glyoxylate, serine-glyoxylate and serine-pyruvate transaminases (G1 code in Figure 1), a NEAA biosynthetic enzyme for glycine biosynthesis. The green, yellow and red circles are marking the plant (Streptophyta), fungi (Dikarya) and animals (Metazoa) branches, respectively. In (A), the distance (given by substitutions per site) from the green circle to the yellow and red circles are, respectively, 0.4 and 1.0. In (B), these values are, respectively, 0.7 and 0.7.

The remaining EAA biosynthetic enzymes in organisms that do not have the complete amino acid pathway (Figure 1) are more susceptible to evolutionary modifications. It is also possible that paralogue subfunctionalization occurred in the common ancestor of animals, fungi and plants, and thus the divergent copy has remained in detriment of the original gene. Considering both hypothesis we set up to analyze enzymes from EAA and functional NEAA pathways present in metazoans. Phylogenetic trees for acetolactate synthase (VIL1 code in Figure 1) and for a group of alanine-glyoxylate, serine-glyoxylate and serine-pyruvate transaminases (G1 code in Figure 1) are represented in Figure 4. As expected, the distance between the ancestors of the two prototrophic groups varies, plant (green circles) and fungi (yellow circles): 0.4 and 0.7, for VIL1 (Figure 4A) and G1 (Figure 4B), respectively. The distance from the ancestors of plant (green circles) to metazoans (red circles) are relatively higher for the remaining enzyme VIL1: 1.0 (as compared to 0.4 measured from plant to fungi, 2.5 fold) than for the NEAA biosynthetic enzyme G1: 0.7 (as compared to 0.7 measured from plant to fungi, 1.0 fold). Thus, the remaining EAA enzymes are experiencing higher divergence after the attainment of amino acids auxotrophy.

**Figure 5:**
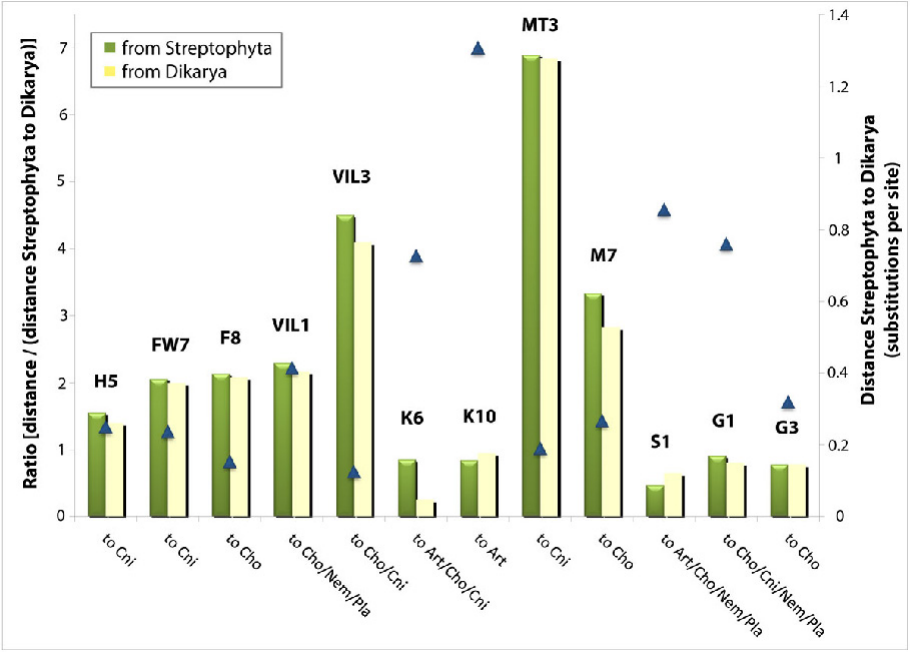
**Relative distance of Metazoa enzymes from homologues of EAA and from NEAA biosynthetic enzymes present in plant and fungi.** Phylogenetic trees were obtained for 12 enzymes, using all eukaryotic clustered proteins. Codes for enzymes are the same as in Figure 1 and are shown over the bars. For normalization, a background distance from the plant phylum Streptophyta to the fungi subkingdom Dikarya was measured and represented by triangles (right Y axis). The distance “from” either Streptophyta (green bars) or Dikarya (yellow bars), “to” the branches that group the clades indicated below the bars, were measured and normalized by the distance Streptophyta/Dikarya, yielding the ratio represented by bars (left Y axis). Only the three enzymes on the right (S1, G1 and G2) participate of biosynthesis of NEAAs: serine (S1) and glycine (G1 and G2). K6 and K10 are enzymes that compose lysine biosynthetic pathways which are not complete, respectively, in Streptophyta or Dikarya (see Figure 1). Abbreviations: Art, Arthropoda; Cho, Choanozoa; Cni, Cnidaria; Nem, Nematoda; Pla, Placozoa.

To support this observation, Figure 5 shows the ratios calculated for 12 enzymes. Only trees that show significant bootstraps for the branches of interest were considered. Enzyme codes in bars are described as in Figure 1. The Y axis at the right side corresponds to the distance measured from plant (Streptophyta) to the ancestor of fungi (Dikarya). This distance was assumed as a background distance to normalize the distances measured “from” plant (green bars) “to” the clades indicated in the X axis. The three enzymes on the right, S1, G1 and G2, belong to NEAA pathways, and the ratios are low. For the enzymes H5, FW7, F8, VIL1, VIL3, MT3 and M7, the ratio shown by green bars are conversely high, ranging from around 1.5 up to 7 fold. These preliminary data suggest that the additional evolutionary modifications have occurred in distinct levels in the enzymes maintained after the loss of biosynthetic capability. M(2) pathway appears as incomplete in Basidiomycota (Figure 1; M8 is absent), however MT3 enzyme used here is present in threonine pathway which is complete in this clade. K6 and K10 are involved in incomplete pathways, respectively, in plants and fungi. Accordingly, the distance measured from plant to fungi is high, and so is the drift between plant to Chordata (K6) or to Arthropoda (K10), therefore yielding balanced lower ratios. Since the ancestor of fungi and plants seems to be equally distant from both of these two groups, and the divergence between plant and Fungi/Metazoa group tends to a trifurcation (see Figure 4), the yellow bars (which represent the distance from fungi to the animal clades in the X axis divided by the background distance from plant to fungi) are similar to the ratios represented by the green bars, independently of how much modification has been occurred to the animal sequences (e.g. VIL1, MT3, G1). Furthermore, a detailed inspection of phylogenetic trees seems to indicate that subfunctionalized paralogues have appeared in basal clades such as Fungi, and those divergent paralogues remain in the more recent groups of organisms, while the copy that previously participated in the biosynthesis was actually deleted in animals. Note some Streptophyta and Ascomycota divergent paralogues (outparalogues) [27] grouped with animal sequences under 100% bootstrap (Figure 4A). Accordingly, similar divergent paralogues were observed for M7 enzyme (Ascomycota and Basidiomycota divergent paralogues grouped with animal sequences, 98% bootstrap, see additional file 2: Phylogenetic tree of 5-methyltetrahydropteroyltriglutamate--homocysteine methyltransferase (M7)). Moreover, for K10 enzyme that participates in the K biosynthetic pathway which is defective in fungi, a divergent paralogue from Streptophyta groups with fungi enzymes (92% bootstrap) near the Arthropoda sequence (Additional file 3: Phylogenetic tree of dihydrodipicolinate synthase (K10)). Thus, the enzymes remaining from biosynthetic pathways show higher divergence, and this might have been acquired due to subfunctionalization in ancient clades.

## Discussion

The advance on genome sequencing and computational methods for clustering homologous proteins has been helping the scientific community to reevaluate several aspects of basic biology. Here we have applied clustering of protein sequences chosen from two clades of organisms that are known to be autotrophic for the biosynthesis of Essential Amino Acids (EAAs). Furthermore, we searched for the enzymes responsible for nitrogen assimilation, incorporating ammonium into glutamate. Lack of cytoplasmic glutamate dehydrogenase leads to a dependency of amino acids consumption as the source of organic nitrogen, i.e., the organism in a certain sense actually becomes auxotrophic to both EAAs and NEAAs (Non-Essential Amino Acids), in order to build other nitrogen-containing molecules.

The work presented here takes advantage of both the Seed Linkage software and a home-built UniProt Enriched KEGG Orthology database (UEKO) as source of information, to rapidly group homologues of fungi and plant amino acid sequences, respectively represented by *Saccharomyces cerevisiae* and *Arabidopsis thaliana*. KEGG Orthology contains to date more than 1 million sequences from nearly 1,000 genomes and it was enriched by a procedure developed by our group to attain 2,442,384 sequences from 25,024 organisms, constituting the UEKO database (UniRef50 enriched KEGG Orthology database, to be published elsewhere and further distributed). Counting the total recruited sequences reported in this work (31,392), the percentage of recruitment by (i) Seed Linkage, (ii) original KO or (iii) the enriched portion of KO (UEKO) was, respectively, 6%, 44% and 50%. Moreover, 26% of all detected enzymes for the phyla represented in Figures 1, 2 and 3 were exclusively detected by Seed Linkage software and/or UEKO database. These numbers reinforce the relevance on the development of homologous searching capability, improving the ability of KEGG Orthology database to build a scenario for the biological processes of interest such as those presented here. Moreover, on top of the search for homologues represented by circles in the Figures, a complementary search using the 31,392 clustered sequences allowed the investigation of all UniProt sequences, including fragments (e.g. UniProt accession B7QGP4, VIL1 from Arthropoda) and some full length proteins not accessed by the initial search (e.g. UniProt accession D3AYE6, complete protein K14, from Amoebozoa; actually a more recent version of KO already incorporates this entry). It is important to notice that, in UniProt, the technical term fragment is applied to partial CDS sequences, a product of incompletely sequenced mRNA, as well as amino acid sequences modeled from the genome that lack initial methionine. Thus they might represent additional evidence of the enzyme presence rather than a reminiscent pseudogene. Stringent criteria (1x10^-10^ e-value, 50% identity and 50% subject coverage cutoffs) were adjusted with extensive manual inspection and additional evidences were included as triangles in the Figures. One evidence collected as triangle claimed our attention, since it came from a clade bearing the complete genome of the well annotated organism *Drosophila melanogaster* (Figure 1, enzyme VIL1, phylum Arthropoda). Manual inspection reveals that the evidence yielded by the additional search (represented by triangle) returned a hit from *Ixodes scapularis* (a genome under “assembly” status), but remarkably, the gene was found to be missing in the fly. Thus, this represents a recent gene loss within a non functional pathway.

The main interest of this work was to depict the evolution of amino acids essentiality, or heterotrophy. Grouping organisms into phyla level allowed easy labeling of clades that comprise organisms with sequenced or draft genomes, as shown in Figures 1, 2 and 3, making it possible to infer deletion events distinctively in these clades. It is important to notice that many phyla contain complete genomes, which allowed us to figure out the deletion process with more certainty. However, the picturing of the entire scenario allowed the analysis to be extended to the branched clades, although this requires additional caution on interpretation. Even escaping the scope of this work, it suggests a demand for planned choice of genomes to be completely sequenced, since as clearly shown here we lack information from several phyla such as the ones represented with empty circles (e.g. Cryptophyta, Haptophyta, Neocallimastigomycota and Glaucophyta). Enzymes not found by our analysis requires further attention and search using more sensitive methods and detailed manual or even experimental analysis, to detect divergent sequences; in other words, the absence of evidence is not evidence of absence. However, the present work exemplifies a method that can be easily applied to other scenarios of gene/pathway loss.

The scenario of amino acid auxotrophy supports the hypothesis of a Great Genomic Deletion model of amino acid biosynthesis in association with heterotrophy. This phenomenon has probably occurred several times, particularly at the origin of metazoans. This deletion has been likely associated with endosymbiotic relationships or with the development of systems specialized in nutrient absorption. It seems that amino acid essentiality has been originated as a phenotypic loss of pathways early in Choanozoa, followed by multiple losses during metazoan evolution. Similar progresses of deletions occur closer to Heterokontophyta and Rhizaria, culminating in Apicomplexa. Rhodophyta and Microsporidia also attain the auxotrophy.

Moreover, remaining enzymes set apart from their original roles in amino acid biosynthetic metabolism seem to be more prone to evolutionary changes whilst enzymes present in complete pathways are more structurally conserved among distant phyla (Figures 4 and 5). Although a detailed investigation is needed, our preliminary analysis suggests that the copies which remained in metazoan genomes may have suffered subfunctionalization and sometimes this might have occurred in more ancestral organisms (Figure 4 and additional files 2 and 3). Thus, in some sense, the orthologue enzyme might actually have been deleted in animals, and the divergent copy is the one remaining. These divergent copies are sometimes named outparalogues. We are currently investigating substitution rate ratios and promoter elements in these genes.

Subsequent deletion includes the enzymes implicated in nitrogen assimilation, which takes place just after the broad deletion of EAAs biosynthetic enzymes (since except metazoans, other eukaryotic clades lack biosynthetic pathways and contains a nitrogen assimilative enzyme), as observed in more derived metazoans, but not Cnidaria. Most Cnidaria are carnivorous, so one possibility is that Cnidaria may benefit from the assimilation of organic nitrogen under long periods of fasting, however this finding needs additional investigation. Thus, the simplest explanation, is that the loss of nitrogen assimilative enzymes are related to lower selective pressure associated with the origin of the most heterotrophic organisms, animals.

To our knowledge this is the first initiative to clarify the complete scenario using powerful homologous grouping approaches and the total repertoire of sequenced genomes.

## Conclusions

The procedures described here provide a deeper analysis of amino acid and nitrogen heterotrophy among distinct taxa, extended to include the entire set of available proteins. They show that amino acid essentiality was a broad phenomenon in eukaryotes, followed by the subsequent nutritional requirement of organic nitrogen, in animals.

## Methods

### Software and databases

Seed Linkage clustering software [14] and detailed explanation of usability can be obtained at http://www.biodados.icb.ufmg.br/eaa/. Seed Linkage requires BLAST (version used was 2.2.20), MySQL (version 5.0.77) [28] and PHP (version 5.1.6) [29].

The protein database is composed of UniProtKB entries (version used was 2010_09) available at http://www.biodados.icb.ufmg.br/eaa/. Except where otherwise indicated, all fragmented proteins were removed from analyses by parsing the description line in FASTA files.

To enrich KEGG Orthology clusters with incomplete genome proteins UniRef50 Enriched KEGG Orthology (UEKO) was built with the procedure described by Fernandes *et al *[15]. A local MySQL database was used.

### Procedure

Amino acid biosynthetic pathways were depicted with KEGG Pathway [30] manual inspection where UniProtKB identifiers for the enzymes used in this work could be retrieved for the model autotrophic organisms *Saccharomyces cerevisiae*, *Arabidopsis thaliana* and, for the archaeal lysine biosynthesis, *Pyrococcus horikoshii*. The procedure starts with the selected sequences used as seed for Seed Linkage search in UniProtKB. The homologous cluster is enriched by (i) entries in KEGG Orthology (KO) belonging to the same KO where the seed is found and (ii) UEKO entries for this same KO. All steps were conducted with MySQL consults and PERL v5.8.8 [31] scripts. To verify the recruitment, seed sequences were used in PSI-BLAST alignments with the recruited sequences, having the PSI-BLAST iterations stopped whenever the score obtained for the seed sequence itself decreases to below 50% of the initial score. Results of search for homologues are represented by circles in the Figures. For more details see additional file 4: List of seed sequences and additional file 5: List of clusters.

Simple BLASTp analysis (10^-10^ e-value cutoff) were also conducted with all UniProt proteins, comprising both UniProt complete and fragment entries, for each phylum against all clustered proteins in this project. Resulting output was filtered to remove alignments with less than both 50% identity and 50% subject coverage. Results of this analysis are represented by triangles in the Figures.

### Taxonomy information

All UniProtKB identifiers could be associated with an organism taxonomy ID with the file available at ftp://ftp.uniprot.org/pub/databases/uniprot/current_release/knowledgebase/idmapping.

Further association of organism taxonomy ID with phyla classification was achieved through a local database built with NCBI taxonomy information obtained at ftp://ftp.ncbi.nih.gov/pub/taxonomy.

Genome statuses were obtained by NCBI Genome Project analysis at: http://www.ncbi.nlm.nih.gov/genomeprj.

### Phylogenetic analyses

For phylogenetic analysis Prankster [32] was used for multiple sequence alignment and MEGA4 [33] to construct the phylogenetic tree using the neighbor-joining method [34] with 500 bootstrap replicates. Branch distances were obtained from phylogenetic trees, from the ancestors of Streptophyta, Dikarya and clades of metazoans. Only branches with significant bootstrap were used. With the distances, a ratio was calculated as below:

where F (from) is either Streptophyta or Dikarya ancestor and T (to) is an animal ancestor (see Figure 5, X axis); and S and D are the ancestors of Streptophyta and Dikarya, respectively. Phylogenetic trees used to compose Figure 5 can be accessed at our server at http://www.biodados.icb.ufmg.br/eaa/.

## List of abbreviations

COG: Cluster of Orthologous Groups; EAAs: Essential Amino Acids; GDH: Glutamate dehydrogenase; KEGG: Kyoto Encyclopedia of Genes and Genomes; KO: KEGG Orthology; NEAAs: Non-Essential Amino Acids; UEKO: UniRef50 Enriched KEGG Orthology.

## Authors' contributions

The work presented here was carried out in collaboration between all authors. FP and JMO defined the research theme. RLMG developed the clustering procedure, created the dataset and conducted the experiments. RLMG and GFR created the figures. RLMG, FP and LKM conducted phylogenetic analyses. GRF created the procedure of Uniref50 enrichment of KEGG Orthology database. HALR developed the PSI-BLAST validation method. JMO, FP and RLMG wrote the paper. All authors supervised and approved the final manuscript.

## Competing interests

The authors declare that they have no competing interests.

## Supplementary Material

Additional file 1 **Sequences and genome status distribution.** Distribution of UniProtKB sequences among available genomes in three sequencing status groups: Complete, Draft plus In Progress and Incomplete. Click here for file

Additional file 2 **Phylogenetic tree of 5-methyltetrahydropteroyltriglutamate--homocysteine methyltransferase (M7).** A phylogenetic tree of one of the four methyltransferases illustrated in Figure 1 for methionine biosynthesis. Red circle represents Chordata and Cnidaria ancestor; Yellow circle Dikarya ancestor and green circle Streptophyta ancestor. Available at [http://www.biodados.icb.ufmg.br/eaa/]. Click here for file

Additional file 3 **Phylogenetic tree of dihydrodipicolinate synthase (K10).** A phylogenetic tree of one of the enzymes illustrated in Figure 1 for lysine biosynthesis. Red circle represents Arthropoda; Yellow circle Dikarya ancestor and green circle Streptophyta and Chlorophyta ancestor. Available at [http://www.biodados.icb.ufmg.br/eaa/]. Click here for file

Additional file 4 **List of seed sequences.** A detailed list of sequences used as initiators for clustering process with UniProtKB identifier, NCBI taxonomy identifier and Enzyme Commission (EC) number. Available at [http://www.biodados.icb.ufmg.br/eaa/]. Click here for file

Additional file 5 **List of clusters.** A detailed list of created clusters for all enzymes with UniProtKB identifier and NCBI taxonomy identifier. Available at [http://www.biodados.icb.ufmg.br/eaa/]. Click here for file
