# Supplementary material for: Amino acids biosynthesis and nitrogen assimilation pathways: a great genomic deletion during eukaryotes evolution
Source: BMC Genomics. 2011 Dec 22;12(Suppl 4):S2. doi: 10.1186/1471-2164-12-S4-S2 (PMC3287585; doi:10.1186/1471-2164-12-S4-S2)
Supplement: Additional file 1 — Sequences and genome status distribution. Distribution of UniProtKB sequences among available genomes in three sequencing status groups: Complete, Draft plus In Progress and Incomplete. [file 1471-2164-12-S4-S2-S1.pdf]

**Kingdom**	**Phylum** / **Status**	**Genomes**			**Sequences**		
		**Complete**	**Draft/Progress**	**Incomplete**	**Complete**	**Draft/Progress**	**Incomplete**
Archaea	Euryarchaeota	36	18	241	43736	6309	101270
	Crenarchaeota	14	4	81	12739	20	46514
	Thaumarchaeota	0	0	67	0	0	6588
	Korarchaeota	0	0	1	0	0	1602
	Nanoarchaeota	0	0	1	0	0	536
Bacteria	Proteobacteria	260	217	3378	409449	313468	2787726
	Firmicutes	101	120	1404	120564	87088	1645424
	Actinobacteria	72	48	1133	193954	56153	487490
	Cyanobacteria	27	20	695	70476	37473	113079
	Tenericutes	23	15	201	8410	5431	22797
	Bacteroidetes	19	51	148	46888	100788	231833
	Spirochaetes	15	9	140	10249	2850	46771
	Chloroflexi	11	0	24	13653	0	38614
	Chlamydiae	6	2	25	5736	67	21562
	Aquificae	6	2	11	4977	3050	10287
	Chlorobi	6	0	13	2296	0	25167
	Deinococcus-Thermus	5	4	51	9775	105	16464
	Fusobacteria	4	11	14	7812	17164	21627
	Thermotogae	4	1	26	2032	3	20820
	Planctomycetes	2	0	13	11528	0	21677
	Verrucomicrobia	1	6	11	4	15585	19169
	Synergistetes	1	3	4	1737	3864	8561
	Acidobacteria	1	1	7	2	4873	16318
	Deferribacteres	1	1	1	2901	1	2338
	Dictyoglomi	1	0	3	5	0	3658
	Fibrobacteres	1	0	2	13	0	3099
	Nitrospirae	0	1	7	0	144	9664
	Elusimicrobia	0	1	2	0	8	2295
	Lentisphaerae	0	0	2	0	0	8863
	Gemmatimonadetes	0	0	1	0	0	3932
Eukaryota	Ascomycota	13	82	863	50653	132052	377064
	Alveolata	5	21	307	6340	74820	75161
	Apicomplexa	5	14	146	6340	34770	40162
	Streptophyta	3	65	15248	91651	282174	134334
	Chlorophyta	3	6	176	16067	10378	17496
	Euglenozoa	3	5	100	22568	26141	12956
	Chordata	2	93	14054	5180	256829	107078
	Arthropoda	2	47	3390	30323	248453	30723
	Microsporidia	2	3	8	1926	149	4714
	Nematoda	1	23	152	23947	29205	1863
	Basidiomycota	1	14	222	9784	7287	44142
	Rhodophyta	1	1	131	276	97	1415
	Amoebozoa	0	10	24	0	21310	20727
	Ciliophora	0	7	104	0	39989	16005
	Heterokontophyta	0	6	300	0	441	40114
	Platyhelminthes	0	5	96	0	17402	1369
	Fornicata	0	5	7	0	396	11652
	Mollusca	0	4	474	0	410	5121
	Rhizaria	0	3	9	0	1342	94
	Cnidaria	0	2	301	0	13310	1663
	Choanozoa	0	2	2	0	8323	10
	Parabasalia	0	1	14	0	44596	73
	Haptophyta	0	1	13	0	177	93
	Placozoa	0	1	11	0	9640	70
	Cryptophyta	0	0	17	0	0	1485
	Neocallimastigomycota	0	0	12	0	0	90
	Glaucophyta	0	0	3	0	0	175
	**Total**	658	956	43891	1243991	1914135	6691594
		45505			9849720		
