# Supplementary figures and images for: Amino acids biosynthesis and nitrogen assimilation pathways: a great genomic deletion during eukaryotes evolution
Source: BMC Genomics. 2011 Dec 22;12(Suppl 4):S2. doi: 10.1186/1471-2164-12-S4-S2 (PMC3287585; doi:10.1186/1471-2164-12-S4-S2)

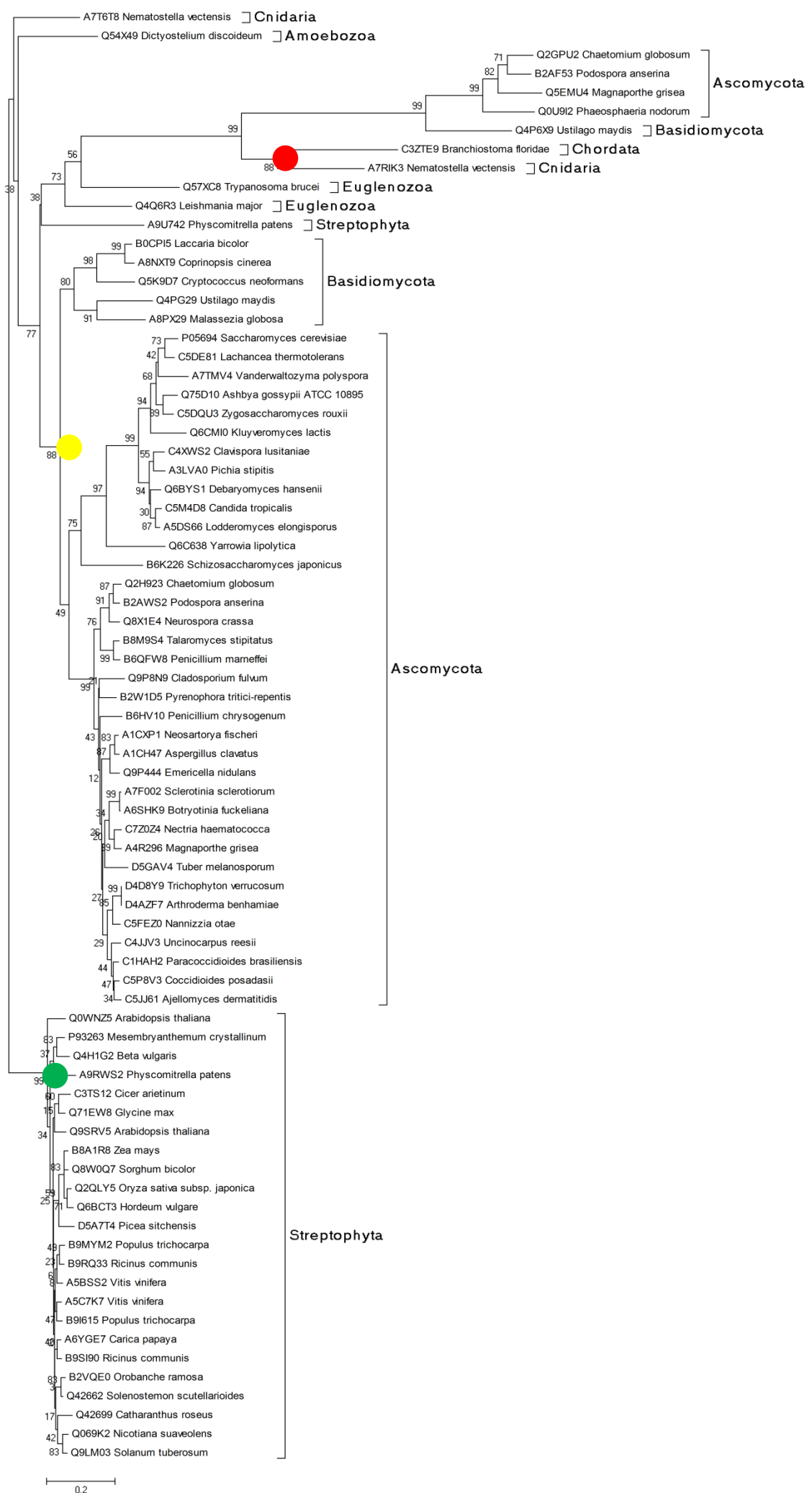

Supplement: Additional file 2 — Phylogenetic tree of 5-methyltetrahydropteroyltriglutamate--homocysteine methyltransferase (M7). A phylogenetic tree of one of the four methyltransferases illustrated in Figure 1 for methionine biosynthesis. Red circle represents Chordata and Cnidaria ancestor; Yellow circle Dikarya ancestor and green circle Streptophyta ancestor. Available at [http://www.biodados.icb.ufmg.br/eaa/]. [file 1471-2164-12-S4-S2-S2.pdf]

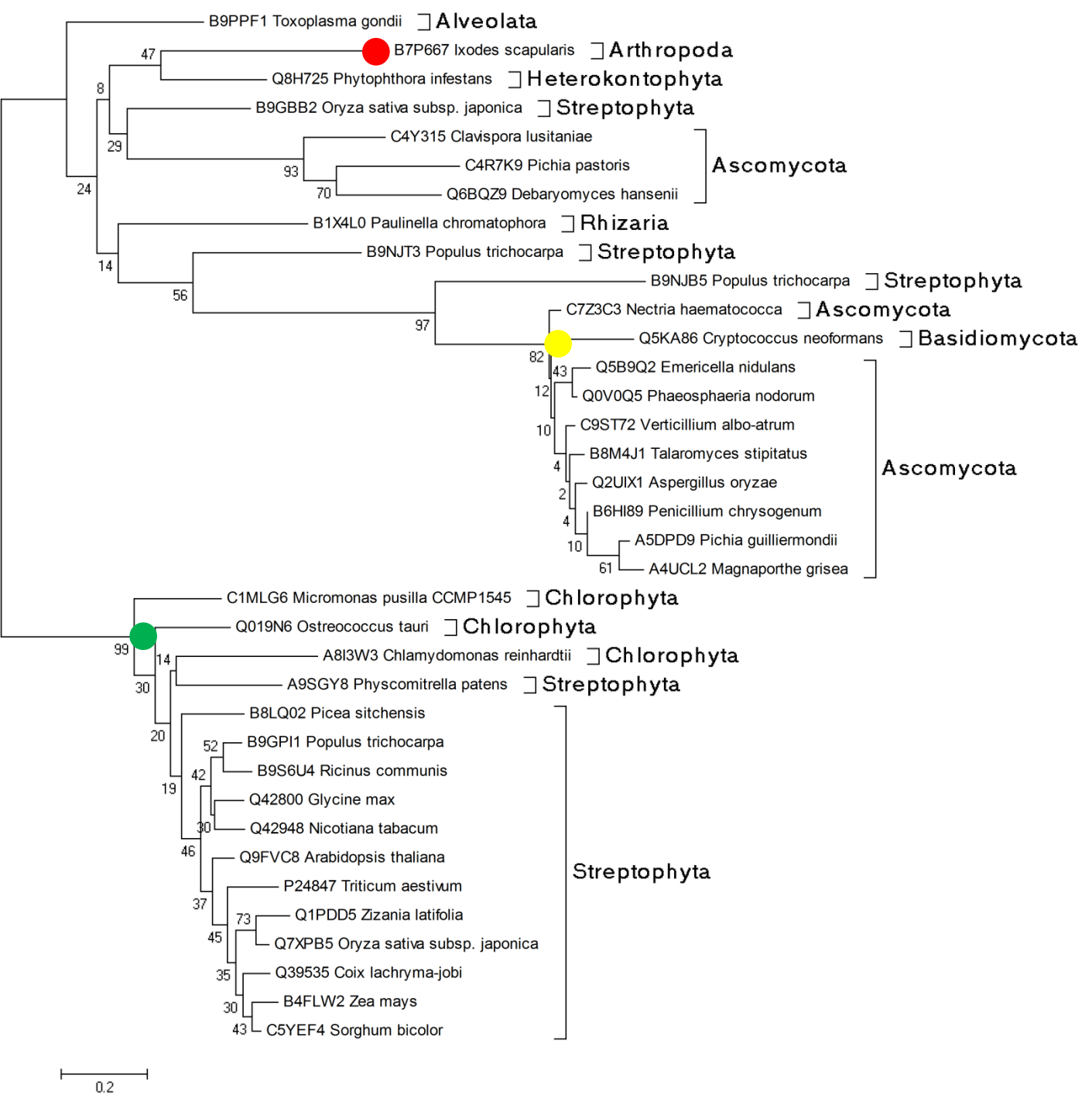

Supplement: Additional file 3 — Phylogenetic tree of dihydrodipicolinate synthase (K10). A phylogenetic tree of one of the enzymes illustrated in Figure 1 for lysine biosynthesis. Red circle represents Arthropoda; Yellow circle Dikarya ancestor and green circle Streptophyta and Chlorophyta ancestor. Available at [http://www.biodados.icb.ufmg.br/eaa/]. [file 1471-2164-12-S4-S2-S3.pdf]
