# Supplementary material for: Amino acids biosynthesis and nitrogen assimilation pathways: a great genomic deletion during eukaryotes evolution
Source: BMC Genomics. 2011 Dec 22;12(Suppl 4):S2. doi: 10.1186/1471-2164-12-S4-S2 (PMC3287585; doi:10.1186/1471-2164-12-S4-S2)
Supplement: Additional file 4 — List of seed sequences. A detailed list of sequences used as initiators for clustering process with UniProtKB identifier, NCBI taxonomy identifier and Enzyme Commission (EC) number. Available at [http://www.biodados.icb.ufmg.br/eaa/]. [file 1471-2164-12-S4-S2-S4.pdf]

Enzime	Seed(s)	Taxonomy	E.C.
ATP phosphoribosyltransferase	Q8GSJ1<br>P00498	3702<br>4932	2.4.2.17
oribosyl-ATP pyrophosphohydrolase / phosphoribosyl-AMP cyclohydrolase	O82768	3702	3.6.1.31/3.5.4.19
5 proFAR isomerase	P40545<br>O82782	4932<br>3702	5.3.1.16
glutamine amidotransferase	P33734<br>Q9SZ30	4932<br>3702	2.4.2.-
imidazoleglycerol-phosphate dehydratase	A8MQQ8<br>P06633	3702<br>4932	4.2.1.19
histidinol-phosphate aminotransferase	Q949X3<br>B9DHD3<br>P07172	3702<br>3702<br>4932	2.6.1.9
histidinol-phosphatase	P38635	4932	3.1.3.15
histidinol dehydrogenase	P00815<br>Q9C5U8	4932<br>3702	1.1.1.23
3-deoxy-7-phosphoheptulonate synthase	Q9SK84<br>P32449	3702<br>4932	2.5.1.54
Pentafunctional AROM polypeptide	P08566	3702	4.2.3.4 / 4.2.1.10/ 1.1.1.25 /2.7.1.71/ 2.5.1.19
3-dehydroquinate synthase	Q8VYV7	3702	4.2.3.4
ehydroquinate dehydratase I/shikimate 5-dehydrogenase	Q9SQT8	3702	4.2.1.10 / 1.1.1.25
shikimate kinase	A8MRG1	3702	2.7.1.71
3-phosphoshikimate 1-carboxyvinyltransferase	Q9FVP6	3702	2.5.1.19
chorismate synthase	P28777<br>Q2V4I0	4932<br>3702	4.2.3.5
chorismate mutase	P32178<br>Q9C544	4932<br>3702	5.4.99.5
prephenate dehydratase	P32452	4932	4.2.1.51
aspartate aminotransferase	Q01802<br>P46646	4932<br>3702	2.6.1.1
histidinol-phosphate aminotransferase	P07172<br>Q949X3<br>B9DHD3	4932<br>3702<br>3702	2.6.1.9
aromatic amino acid aminotransferase	P53090<br>P38840	4932<br>4932	2.6.1.57
tyrosine aminotransferase	Q9LVY1	3702	2.6.1.5
anthranilate synthase	P00899<br>Q9FXK1	3702<br>4932	4.1.3.27
ıctional indole-3-glycerol phosphate synthase/anthranilate synthase	P00937	3702	4.1.3.27<br>4.1.1.48
anthranilate phosphoribosyltransferase	Q02166<br>P07285<br>Q02166	3702<br>4932<br>3702	2.4.2.18
N (5-Phosphoribosyl) anthranilate isomerase	B3H4J9<br>P00912	3702<br>4932	4.3.1.24
indole-3-glycerol phosphate synthase	P49572<br>Q1EBW5	3702<br>3702	4.1.1.48
tryptophan synthase	Q42529<br>P00931<br>P25269<br>Q0WS13<br>Q9FFW8	3702<br>4932<br>3702<br>3702<br>3702	4.2.1.20
acetolactate synthase	P17597<br>P07342<br>P25605	3702<br>4932<br>4932	2.2.1.6
ketol-acid reductoisomerase	Q05758<br>P06168	3702<br>4932	1.1.1.86
dihydroxy-acid dehydratase	P39522<br>Q9LIR4	4932<br>3702	4.2.1.9
branched-chain amino acid aminotransferase	Q9M439<br>B9DHH5<br>P38991<br>P47176	3702<br>3702<br>4932<br>4932	2.6.1.42
threonine dehydratase	Q9ZSS6	3702	4.3.1.19

	P00927	4932	
Pyruvate dehydrogenase	O24457<br>Q8H1Y0<br>Q9C6Z3<br>P52901<br>O64688<br>Q38799	4932<br>4932<br>4932<br>4932<br>4932<br>4932	1.2.4.1
2-isopropylmalate synthase	Q9LPR4<br>Q9C550<br>P06208	3702<br>3702<br>4932	2.3.3.13
3-isopropylmalate dehydratase	P07264<br>Q94AR8<br>Q9ZW85	4932<br>3702<br>3702	4.2.1.33
3-isopropylmalate dehydrogenase	Q9SA14<br>P04173	3702<br>4932	1.1.1.85
homocitrate synthase	Q12122	4932	2.3.3.14
homoaconitate hydratase	P49367	4932	4.2.1.36
homoisocitrate dehydrogenase	P40495	4932	1.1.1.87
aminoadipate-semialdehyde dehydrogenase	P07702<br>P50113	4932<br>4932	1.2.1.31
charopine dehydrogenase (NADP+, L-glutamate forming)	P38999	4932	1.5.1.10
accharopine dehydrogenase (NAD+, L-lysine forming)	P38998	4932	1.5.1.17
aspartate kinase	Q9S702<br>Q9LYU8<br>O23653	3702<br>3702<br>3702	2.7.2.4
bifunctional aspartokinase/homoserine dehydrogenase	Q9SA18	4932	2.7.2.4/ 1.1.1.3
aspartate-semialdehyde dehydrogenase	Q8VYI4	3702	1.2.1.11
dihydrodipicolinate synthase	Q9FVC8<br>Q0WSN6	3702<br>3702	4.2.1.52
dihydrodipicolinate reductase	Q9FJ82	3702	1.3.1.26
LL-diaminopimelate aminotransferase	Q93ZN9	3702	2.6.1.83
diaminopimelate epimerase	Q9LFG2	3702	5.1.1.7
diaminopimelate decarboxylase	Q949X7	3702	4.1.1.20
homoserine dehydrogenase	P31116	4932	1.1.1.3
homoserine O-acetyltransferase	P08465	4932	2.3.1.31
cystathionine gamma-synthase	P55217<br>P47164	3702<br>4932	2.5.1.48
cystathionine beta-lyase	P53780<br>B9DGA0<br>P43623	3702<br>3702<br>4932	4.4.1.8
5-methyltetrahydropteroyltriglutamate--homocysteine methyltransferase	Q0WNZ5<br>P05694	3702<br>4932	2.1.1.14
homocysteine S-methyltransferase	Q8LAX0	3702	2.1.1.10
betaine-homocysteine methyltransferase	Q93088	9606	2.1.1.5
methyltetrahydrofolate--homocysteine methyltransferase	Q99707	9606	2.1.1.13
serine O-acetyltransferase	Q42588<br>Q42538	3702<br>3702	2.3.1.30
cysteine synthase A	P53206<br>P06106	4932<br>4932	2.5.1.47
cystathionine gamma-lyase	P31373	4932	4.4.1.1
homoserine kinase	Q8L7R2<br>P17423	3702<br>4932	2.7.1.39
threonine synthase	Q9SSP5<br>P16120	3702<br>4932	4.2.3.1
D-3-phosphoglycerate dehydrogenase	O04130<br>Q9LT69<br>O49485<br>P40054<br>P40510	3702<br>3702<br>3702<br>4932<br>4932	1.1.1.95
phosphoserine aminotransferase	P33330<br>Q9SHP0<br>Q96255<br>Q1EBU1	4932<br>3702<br>3702<br>3702	2.6.1.52

phosphoserine phosphatase	O82796<br>P42941	3702<br>4932	3.1.3.3
Serine/<br>threonine dehydratase	P25379<br>P00927<br>Q9ZSS6	4932<br>4932<br>3702	4.3.1.17 / 4.3.1.19
Serine-glyoxylate / Alanine—glyoxylate /<br>serine-pyruvate transaminases	P43567<br>Q56YA5	4932<br>3702	2.6.1.45
glycine hydroxymethyltransferase	Q9LM59<br>P37292<br>P37291	3702<br>4932<br>4932	2.1.2.1
threonine aldolase	P37303<br>Q8RXU4	4932<br>3702	4.1.2.5
glutamate dehydrogenase, NAD-specific	P33327	4932	1.4.1.2
glutamate dehydrogenase, NAD(P)	Q38946<br>Q43314<br>Q9S7A0	3702<br>3702<br>3702	1.4.1.3
glutamate dehydrogenase, NADP-specific	P39708<br>P07262<br>Q9C8I0	4932<br>4932<br>3702	1.4.1.4
