# Supplementary material for: Amino acids biosynthesis and nitrogen assimilation pathways: a great genomic deletion during eukaryotes evolution
Source: BMC Genomics. 2011 Dec 22;12(Suppl 4):S2. doi: 10.1186/1471-2164-12-S4-S2 (PMC3287585; doi:10.1186/1471-2164-12-S4-S2)
Supplement: Additional file 5 — List of clusters. A detailed list of created clusters for all enzymes with UniProtKB identifier and NCBI taxonomy identifier. Available at [http://www.biodados.icb.ufmg.br/eaa/]. [file 1471-2164-12-S4-S2-S5.pdf]

ATP phosphoribosyltransferase

UniProt	TXID
A1CDD6	5057
A1DCB4	331117
A2R1F8	425011
A2XC59	39946
A3ADU6	39947
A3LQN3	4924
A4RN67	148305
A4RQW7	436017
A5DB59	4929
A5E3B2	36914
A6R7R9	339724
A6RIQ7	332648
A6ZR07	307796
A6ZXA7	307796
A7EFF7	665079
A7TGA4	436907
A8NRN1	240176
A8PXL8	425265
A9RE17	145481
A9TKG9	145481
A9V418	81824
B0CV00	486041
B0YCF8	451804
B1X5W0	39717
B2AYV5	5145
B2CNY2	37727
B2VZY6	426418
B3LS69	285006
B4FED7	4577
B5VHI8	545124
B6K7N1	402676
B6QDW1	441960
B7FG77	3880
B7FKJ6	3880
B7FST0	556484
B8C4E1	35128
B8LKY9	3332
B8MAE4	441959
B8MW29	332952
B9IQ50	3694
B9N0B8	3694
B9TB20	3988

phosphoribosyl-ATP pyrophosphohydrolase / phosphoribosyl-AMP cyclohydrolase

UniProt	TXID
O82768	3702
D7KGN4	81972
B9N630	3694
A9PAQ7	3694
C6TGF5	3847
C5XH91	4558
C5YRI4	4558
B6TR63	4577
B4FTI8	4577
Q5NBQ1	39947
A2WNF2	39946
A9NSD7	3332
C1FFV9	296587
C1MQ66	564608
A4RVN0	436017
A9TNG9	145481
A8HTA8	3055
Q01BC3	70448
B1X3F5	39717

phosphoribosylformimino-5-aminoimidazole carboxamide ribotide isomerase

UniProt	TXID
P40545	4932
D6VVQ9	559292
C8ZAN0	643680
C7GPW8	574961
B5VKS1	545124
B3LTM8	285006
A6ZVP7	307796
C5DHI6	559295
C5DWL4	559307
Q6FPN5	5478
Q6CQL7	28985
Q75AP1	33169
Q6C2U0	4952
C4XY51	306902
Q595W2	4922
C4R7L1	644223
A5DQ45	4929
Q6BUV9	4959
C5MDV0	294747
C4YGS6	5476
Q5A461	5476
Q9HFV5	5476
Q0TVM2	13684
B9WGL6	573826
C7YQZ0	660122
A3GF99	4924
A5E4R0	36914
A8NXH0	240176
Q5KMJ6	5207
Q4WTU8	5085
B0Y3C5	451804
A4QTQ4	148305
B2AVV1	5145
A1C9V9	5057
A1CZF2	331117
B0DEY9	486041
A7F4D3	665079
Q0CA71	341663
C1G297	502780
Q7RXQ8	5141
B6K0F2	402676
Q2HA31	38033

B9WI74	573826
C0NWV6	447093
C0PKL2	4577
C0SJH4	482561
C1E526	296587
C1FD92	296587
C1GN18	502780
C1HE96	502779
C1MKU1	564608
C1N2F4	564608
C4JZZ0	336963
C4QXU6	644223
C4Y1R3	306902
C4YS40	5476
C5DBN7	559295
C5DWQ6	559307
C5FYG9	554155
C5GR28	559297
C5K2L1	559298
C5MJ95	294747
C5PBG2	222929
C6HBE9	544712
C6TAE9	3847
C7GX46	574961
C7YJX1	660122
C8V7A0	227321
C8Z760	643680
C9SJI6	526221
D0NCB8	403677
D1ZGP9	5147
D3DLV8	559292
D4B1U9	663331
D4D7Y3	663202
D5GKQ7	39416
D7KJU4	81972
D7KY77	81972
D7KY80	81972
O46887	60913
P00498	4932
P40373	4896
P46586	5476
Q01GN6	70448
Q0CPP7	341663
Q0UWH6	13684
Q10S55	39947
Q2HG71	38033
Q10184	4896
A2R9J4	425011
C0SG53	482561
D1ZBG9	5147
C5P783	222929
B8MBV5	441959
B2VV69	426418
B6QGA6	441960
B6H0P4	500485
D5GEF5	39416
C1GPG6	502779
C5FVZ9	554155
A6RU76	332648
A9UW68	81824
C5JMG9	559298
C5GAK6	559297
C0NQY6	447093
Q2UTQ1	5062
D4DCL9	663202
D4AII3	663331
A6RFY4	339724
C6HPY0	544712
D0NQR0	403677
B8CDV9	35128
D7LIP8	81972
Q5TKS8	3702
O82782	3702
C0Z2T9	3702
B9FPI1	39947
B7GE60	556484
A8JDM6	3055
B7FHU9	3880
C1MIV3	564608
C1MIV2	564608
C6TJR8	3847
B6TRF8	4577
C4IYN8	4577
B4FIP2	4577
C0PFD1	4577
B6UEA7	4577
C5WWI2	4558
C5YXM8	4558
D7G369	2880
A4RZJ6	436017
B8AY22	39946
Q015Z9	70448

Q2UT69	5062
Q4P1J6	5270
Q4WGB9	5085
Q56US4	226036
Q56US5	226036
Q56UT2	226033
Q56UT3	226033
Q595W5	4922
Q5B6T2	162425
Q5F0I1	42374
Q5KIW5	5207
Q6BRU4	4959
Q6CID6	28985
Q6FLT7	5478
Q75AK8	33169
Q7SDF1	5141
Q8GSJ1	3702
Q8H8D3	39947
Q8L9K8	3702
Q99145	4952
Q9S762	3702
Q9SLW4	3702

C9SVG4	526221
C1E011	296587
Q5BAY7	162425
C8VN40	227321
A8Q0I1	425265
B9SRY4	3988
A5ASH5	29760
A9STZ1	145481
C4JNC3	336963
C7J278	39947

glutamine amidotransferase

UniProt	TXID
P33734	4932
D6VQP4	559292
D3UEZ5	643680
B3LMR4	285006
C7GMY3	574961
A6ZLI7	307796
C5E1L7	559307
Q6FX00	5478
C5E2I9	559295
Q6CL11	28985
Q754W3	33169
C4R8P0	644223
Q0CL18	341663
Q2UMI6	5062
A7EIH3	665079
Q6CCR8	4952
A6SEB1	332648
Q870Y9	5141
Q0TX23	13684
D1ZSZ1	5147
A1DG44	331117

imidazoleglycerol-phosphate dehydratase

UniProt	TXID
A0JJW7	100272
A0JJW8	100272
A1CM65	5057
A1DLV8	331117
A2R4J2	425011
A4S3Y5	436017
A5DCL8	4929
A5DUG7	36914
A6P4B5	135859
A6P4B6	5599
A6R119	339724
A6RJP8	332648
A6ZP46	307796
A7E563	665079
A7TQC3	436907
A8I4F6	3055
A8MQQ8	3702
A8NGC3	240176
A8Q9P0	425265
A9P0A9	3332
B0D3S7	486041

histidinol-phosphate aminotransferase

UniProt	TXID
A1CP53	5057
A1D207	331117
A2QAL3	425011
A3LSV4	4924
A4R5D4	148305
A4RSZ6	436017
A5DDL3	4929
A5E3V6	36914
A6R8T8	339724
A6RQF2	332648
A6ZVG1	307796
A7E9J3	665079
A7THE9	436907
A8J3D3	3055
A8MRI5	3702
A8P5U9	240176
A8PCL5	240176
A8Q8A0	425265
A9NX10	3332
A9T6Y6	145481
A9VDN0	81824

C7YRY8	660122	B0YCY2	451804	B0DTT8	486041
C1GP40	502779	B1X4R8	39717	B0XMN9	451804
C0SFE2	482561	B2VVA2	426418	B1X4B7	39717
A1C5W0	5057	B4F8F3	4577	B2AS75	5145
Q4WH91	5085	B4FI45	4577	B2VYI8	426418
B0XW23	451804	B5VS85	545124	B3LTW3	285006
B6HK56	500485	B6HGA3	500485	B4FAW7	4577
B5VEJ8	545124	B6JW23	402676	B5VKJ3	545124
Q9P4P9	162425	B6QVE9	441960	B6HAF7	500485
C8VBA3	227321	B6TC53	4577	B6K373	402676
C1GL59	502780	B8AUF4	39946	B6QBB4	441960
B6Q367	441960	B8MS43	441959	B6TRS1	4577
C5FFA9	554155	B8ND57	332952	B7G747	556484
C0NQK4	447093	B9P9G9	3694	B8AHA9	39946
Q2GYU2	38033	B9RTS0	3988	B8BPZ8	296543
B2AC63	5145	B9WBE6	573826	B8M3C4	441959
A6QXC8	339724	C0NQI6	447093	B8N6W4	332952
A2QFK7	425011	C0PA62	4577	B8P2C7	561896
C6HQA9	544712	C0SGZ0	482561	B9DHD3	3702
C5K3V3	559298	C1GG64	502780	B9SB59	3988
C5GYK0	559297	C1GXF3	502779	B9WFD8	573826
B8LT94	441959	C4JNN5	336963	C0NL35	447093
D5GBP0	39416	C4R5K6	644223	C0S401	482561
O94303	4896	C4XVR0	306902	C1FED0	296587
A4RFT8	148305	C5DET9	559295	C1FYG5	502780
C5P669	222929	C5FW12	554155	C1GUI5	502779
D4B2A2	663331	C5GCN7	559297	C1ML32	564608
D4D7G4	663202	C5JW68	559298	C3SA65	15368
B6K508	402676	C5M6Z5	294747	C4JYD9	336963
C4JT92	336963	C5P226	222929	C4R1F7	644223
A5DAA2	4929	C5YG33	4558	C4XWD3	306902
A3LSB3	4924	C6HSB7	544712	C4YI23	5476
Q6BZ52	4959	C6T988	3847	C5DMK2	559295
C4XX45	306902	C6YXH4	39947	C5DPA3	559307
A5E679	36914	C6YXL2	39947	C5FC63	554155
Q00ZE6	70448	C7GWH9	574961	C5G7N6	559297
B9WJS1	573826	C7YUB2	660122	C5JKH7	559298
A4S499	436017	C8V0L8	227321	C5M214	294747
A9UYA3	81824	C8ZGV8	643680	C5PAB2	222929
C1FJN8	296587	C9S945	526221	C5Y092	4558
C4YT06	5476	D0NH24	403677	C6TN17	3847
Q9HFV7	5476	D0P2W5	403677	C7Z4V1	660122
C1N0H6	564608	D1ZHS3	5147	C8VRC2	227321
B2VVH1	426418	D4AIG9	663331	C8ZAC8	643680
D0N3W2	403677	D4DCK2	663202	C9S832	526221
A9S2M5	145481	D4NXF8	318829	D0MY30	403677

Q10NW0	39947
B9F6U6	39947
B8AKB1	39946
A8N1B5	240176
Q9SZ30	3702
Q8GX14	3702
D7MEL3	81972
C4J411	4577
C5WQU1	4558
B9SVA2	3988
B9GFE9	3694
B9HNP1	3694
B0CPN4	486041
Q4P7Q5	5270
B8LDL7	296543
B7FVQ7	556484
Q5KNA0	5207
A7SPR8	45351
A8PRW0	425265
C9S5S7	526221
B8NPG7	332952
A2XM35	39946
C0HF10	4577
P48262	2762
Q7Y020	39947
D5GDC8	39416
D6W2Q9	559292
D7FNK5	2880
D7MBA2	81972
O23346	3702
O42621	148305
O94126	4903
O94153	5421
P06633	4932
P28624	4792
P34041	5544
P34047	3702
P40374	4896
P40919	5207
P56090	5476
Q02986	4934
Q0CH64	341663
Q0UFU5	13684
Q12578	5478
Q25AR2	4530
Q2HAY0	38033
Q2U9D6	5062
Q43072	3888
Q4PFJ4	5270
Q4WDG8	5085
Q58FL5	5061
Q59LL5	5476
Q5AYU4	162425
Q67YN9	3702
Q67YZ9	3702
Q682C0	3702
Q6BTY3	4959
Q6C3L0	4952
Q6CLR6	28985
Q6JV40	4911
Q6XD66	4950
Q75B47	33169
Q7LV87	5478
Q7XTN6	39947
Q92447	4922
Q96UK2	4954
Q9C1D4	4924
Q9C1R3	162425
Q9HEG3	5141
Q9UVE1	559307
D1ZCJ6	5147
D4AV67	663331
D4D1H8	663202
D5GI57	39416
D6VVH1	559292
D7FPJ8	2880
D7KZ00	81972
O82030	4097
P07172	4932
P36605	4896
P56099	5479
Q0D0C3	341663
Q0DY86	39947
Q0UQ19	13684
Q27GM5	3702
Q2H2S0	38033
Q2UCT8	5062
Q4P634	5270
Q4WS41	5085
Q595W3	4922
Q59SJ5	5476
Q5BFG3	162425
Q5KM46	5207
Q6BMG0	4959
Q6C7F4	4952
Q6CLF0	28985
Q6FX16	5478
Q6MY81	5085
Q6YV10	39947
Q75B26	33169
Q7S9U6	5141
Q8S3S8	39947
Q949X3	3702
Q9FEW2	4092

histidinol-phosphatase		histidinol dehydrogenase		threonine dehydratase	
**UniProt**	**TXID**	**UniProt**	**TXID**	**UniProt**	**TXID**
P38635	4932	P00815	4932	A0BE77	5888
D6VTQ5	559292	D6VQY5	559292	A0CM66	5888
B3LUN4	285006	C7GSY7	574961	A0D3F4	5888
C8Z7W0	643680	B5VES8	545124	A0D573	5888
B5VI52	545124	B3LU52	285006	A0DHM0	5888
C7GYD0	574961	A6ZTG0	307796	A0E6U7	5888
A7A262	307796	Q12670	4931	A0FKE6	4081
C5E1R2	559307	Q6FX86	5478	A1CH94	5057
A7TDX0	436907	C5DZ21	559307	A1CHC6	5057
Q6FQ56	5478	B2G433	4956	A1CLE7	5057
C5E3F8	559295	O13471	28985	A1CR51	5057
Q757N4	33169	C5DK34	559295	A1CXG1	331117
Q6CXG5	28985	A7TFF6	436907	A1CXJ1	331117
B5RTB4	4959	Q751X2	33169	A1D414	331117
A3LY67	4924	A1BPP9	27317	A1D5X3	331117
C5MGD3	294747	C4QXL7	644223	A2FA06	5722
B9WKJ5	573826	P45353	4922	A2FH13	5722
C4YSZ9	5476	Q12201	4922	A2FRC0	5722
Q59R21	5476	C5M233	294747	A2QI44	425011
A5DQT0	4929	B9WF89	573826	A2QI80	425011
C4Y0D4	306902	O74712	5476	A2QU10	425011
A5E6D7	36914	Q59UT2	5476	A2XWA9	39946
Q6C6Z5	4952	C4YI61	5476	A3LW23	4924
B0D579	486041	A5E3Y9	36914	A4H4N7	5660
B0DA81	486041	A3LSS5	4924	A4HSW5	5671
B6JV46	402676	Q6CGV2	4952	A4QUE4	148305
D5GJN0	39416	A5DE04	4929	A4RI26	148305
A8NZG4	240176	Q6BM15	4959	A4S5M8	436017
Q2PIQ9	5062	C4Y9Q9	306902	A5B3Z7	29760
Q2TW36	5062	C5JU56	559298	A5DG69	4929
B8NYT7	332952	C5GK98	559297	A5DWY6	36914
B8NX22	332952	C5PAF1	222929	A5HUN0	4513
B8PEW0	561896	C5FCK3	554155	A6RBB5	339724
Q0CVP7	341663	D4AV25	663331	A6RGX2	339724
A8Q7S5	425265	A1DRJ2	13684	A6RUK3	332648
A9UY93	81824	A2IBN3	13684	A6SSX5	332648
Q595W4	4922	Q2PPB3	13684	A6ZR41	307796
C4QX01	644223	Q0UA12	13684	A6ZTD3	307796
A2R2I3	425011	A1DRK4	13684	A7A071	307796
O14059	4896	D1ZH00	5147	A7EEW4	665079
Q4W9R1	5085	B2W4V9	426418	A7F680	665079
B0YEF3	451804	P07685	5141	A7RTQ7	45351
Q0U6E0	13684	A1DRK5	54790	A7TFC2	436907
A1D9M3	331117	A1DRK6	54790	A7TKZ0	436907

A7ETF6	665079
A6RSI8	332648
B6H3B9	500485
B6QGE8	441960
B2W097	426418
C4JH85	336963
A1C968	5057
C9SUM3	526221
C5P3X8	222929
Q5AXD6	162425
C8VB59	227321
Q5KJZ9	5207
Q55W98	5207
C7YYF4	660122
A4QWC7	148305
C0SJ61	482561
C1GMX2	502780
B2ASC1	5145
Q4P8I0	5270
C1HCC3	502779
C5JX61	559298
C5GRN2	559297
C0NVK0	447093
C5FG56	554155
D1ZBW9	5147
C6HH95	544712
Q7SAH5	5141
D4D1N0	663202
D4B512	663331
B3RWH6	10228
A6REF8	339724
A6QYA0	339724
B8M2Z5	441959
C0NEX3	447093
A6RN57	332648
C1H3R3	502779
A1DRK7	289418
C1GHM0	502780
A1DRJ7	215456
A1DRJ8	215456
A7F2X1	665079
C0SE67	482561
B2AYL5	5145
C7YID0	660122
B6QAZ4	441960
A4RL35	148305
A1D1W0	331117
A1CP07	5057
A8NXP9	240176
Q4WRZ4	5085
B0XMT5	451804
A2QAS4	425011
C4JY90	336963
Q0D075	341663
B6H9W1	500485
Q5BF83	162425
C8VQH9	227321
Q2ULB1	5062
B8N416	332952
B0CYG3	486041
Q7ZA54	76867
C6HNT6	544712
A8Q4R2	425265
Q4P7D9	5270
Q5KHK8	5207
Q2HFX2	38033
D5GLU8	39416
Q9P777	4896
B6JZU7	402676
C9SQK1	526221
A4RS01	436017
A9RLK5	145481
A9THQ4	145481
C1N4U9	564608
C1EJ66	296587
P24226	3716
Q9C5U8	3702
A8BFR5	184922
A8I9R1	3055
A8J977	3055
A8N6Z1	240176
A8PY36	425265
A8WNC1	6238
A9NZE1	3332
A9PI64	3694
A9T7L7	145481
A9TYA8	145481
B0D0M2	486041
B0E7X3	370354
B0EJK9	370354
B0EK81	370354
B0WUZ1	7176
B0XPH8	451804
B0XZX9	451804
B0Y5X4	451804
B0Y607	451804
B1N2N4	5759
B1X3V5	39717
B2WAH4	426418
B2WP86	426418
B3LQP1	285006
B3LRK3	285006
B3LU22	285006
B3M069	7217
B3P1R0	7220
B4FIW6	4577
B4GP49	7234
B4HKF1	7238
B4JI58	7222
B4K4Y7	7230
B4LW10	7244
B4NAJ0	7260
B4PUM3	7245
B4QWN7	7240
B5VHL2	545124
B5VLU7	545124
B6GW79	500485
B6H312	500485
B6H3S9	500485
B6H461	500485
B6H8Y6	500485
B6JWQ1	402676
B6QHL6	441960

D7MQI7	81972
B9GH07	3694
B9N5Q4	3694
B7FNC7	3880
B9SBW3	3988
A8IA89	3055
Q5NAY4	39947
Q0JPA8	39947
A2WMG4	39946
B8A2L1	4577
C0HDR0	4577
B6TZ66	4577
A9NV24	3332
A9VA77	81824
A1D0Y3	331117
A2R4B4	425011
B8MW43	332952
O59667	4896
Q0UK43	13684
Q2UT59	5062
Q4WR38	5085
A1DRJ3	215456
A1DRJ6	215456
A1DRK0	13684
A1DRK1	13684
A1DRK2	54790
A1DRK3	54790
A9QVF0	13684
A9QVF1	13684
A9QVF3	13684
A9QVF6	478522
B0XPL0	451804
B2WPW7	426418
B6K2D2	402676
C7ZNF3	660122
C8VK05	227321
C9SN00	526221
C9SNW0	526221
D0NQD1	403677
D5GLU9	39416
Q5B9Q7	162425

B6QVU8	441960
B7FKK4	3880
B7FV67	556484
B7G9B5	556484
B8C651	35128
B8CA45	35128
B8LQZ5	3332
B8LR80	3332
B8M831	441959
B8MJJ4	441959
B8MTE8	441959
B8N9L9	332952
B8N9Q5	332952
B8NEE4	332952
B8NST7	332952
B8NXW6	332952
B8P126	561896
B8PIK6	561896
B9FB69	39947
B9GKJ4	3694
B9MTL7	3694
B9P9D4	3694
B9RC97	3988
B9SRK6	3988
B9TLI3	3988
B9WBR7	573826
C0NUL1	447093
C0NZL1	447093
C0RYW0	482561
C0SHC7	482561
C1E7U4	296587
C1G792	502780
C1GGJ3	502780
C1GX28	502779
C1GYL4	502779
C1MX69	564608
C1MZ44	564608
C3Y9T1	7739
C4JHR3	336963
C4JWT5	336963
C4LUI9	5759
C4LVZ3	5759
C4Q4Y7	6183
C4QXV5	644223
C4Y8M5	306902
C4YIT5	5476

C5DCQ0	559295
C5DJY9	559295
C5DQT2	559307
C5FMW6	554155
C5FSQ8	554155
C5GDJ4	559297
C5GRI2	559297
C5JIT2	559298
C5JRJ5	559298
C5M6K9	294747
C5P446	222929
C5PCI1	222929
C5WN25	4558
C6H216	544712
C6HPI2	544712
C6LRX6	598745
C7GIZ5	574961
C7GRR8	574961
C7GT17	574961
C7YIV4	660122
C7YRV2	660122
C7ZH37	660122
C8V6G4	227321
C8V6Q6	227321
C8VPK7	227321
C8Z427	643680
C8Z799	643680
C8ZBW2	643680
C9SF28	526221
D0MT19	403677
D0N7P9	403677
D1ZRE9	5147
D2VDG8	5762
D3B961	670386
D3DLZ2	559292
D4AVK5	663331
D4DDK1	663202
D5GA40	39416
D6VQV4	559292
D6VWY5	559292
D6WSN2	7070
D7L8V0	81972
D7LZN3	81972
O42615	37620
O59791	4896
O94634	4896

P00927	4932
P25306	4081
P25379	4932
P36007	4932
Q0CLL4	341663
Q0CLP5	341663
Q0CS26	341663
Q0CTG7	341663
Q0U0P1	13684
Q0V4E4	13684
Q10DZ5	39947
Q17F43	7159
Q17F44	7159
Q21080	6239
Q22B57	312017
Q23ML3	312017
Q23WP3	312017
Q29BL4	46245
Q2GMH0	38033
Q2HGP8	38033
Q2PGG3	3702
Q2TYD7	5062
Q2U839	5062
Q2UGM2	5062
Q2UGQ4	5062
Q2UUE9	5062
Q39469	3827
Q4P450	5270
Q4QJ04	5664
Q4WJF6	5085
Q4WP28	5085
Q4WP60	5085
Q4WYW2	5085
Q55JX2	5207
Q59P56	5476
Q5B5G3	162425
Q5B6G4	162425
Q5B6K0	162425
Q5BAA5	162425
Q5K9M8	5207
Q6BMV7	4959
Q6CAI4	4952
Q6CE17	4952
Q6CMJ3	28985
Q6CPW0	28985
Q6FQM1	5478

Q6JAG1	4558
Q751S7	33169
Q75BN9	33169
Q7PQF6	7165
Q7S456	5141
Q7XSN8	39947
Q86AP7	44689
Q86QZ7	5741
Q86ZL8	5145
Q8GUG5	3702
Q8GW70	3702
Q8W314	39947
Q8X0J0	5141
Q95XY8	6239
Q9AUQ1	4530
Q9AXU4	49451
Q9T0D1	3702
Q9VHF0	7227
Q9ZSS6	3702

acetolactate synthase

**UniProt**	**TXID**
A0A174	3702
A0PCE4	44972
A1CH32	5057
A1CKD6	5057
A1CMZ4	5057
A1CXQ6	331117
A1D0U4	331117
A1D735	331117
A1L0T0	9606
A1Z0Y7	277990
A1Z0Y8	277990
A1Z0Y9	277990
A1Z0Z0	277990
A2Q4R2	3880
A2QFR5	425011
A2R7N6	425011
A2XSS8	39946
A2XST3	39946
A3BBP3	39947
A3GGC4	4924
A3LUG6	4924
A4R1P9	148305
A4R4K6	148305
A4S4D9	436017

ketol-acid reductoisomerase

**UniProt**	**TXID**
Q05758	3702
D7LW57	81972
A5AGN5	29760
B9H2U7	3694
B9N3F9	3694
O82043	3888
Q9SM58	3888
B9S999	3988
B9SGJ6	3988
D6QSY0	4058
B5LAT1	4072
D2DKE6	3847
Q01292	3562
C5XF87	4558
C5YW13	4558
B6TF69	4577
B4FFZ2	4577
C0PC62	4577
Q65XK0	39947
B9FLQ7	39947
A2ZW27	39947
Q5VPB9	39947
B8AWV4	39946
A9TGX1	145481

dihydroxy-acid dehydratase

**UniProt**	**TXID**
P39522	4932
D6VWJ2	559292
D3UF79	643680
B3LQB4	285006
A6ZPY4	307796
C7GWK6	574961
A7TET1	436907
Q6FXQ1	5478
Q6CJ26	28985
C5DCB1	559295
C5DSB2	559307
C5E0C7	559307
Q75CN6	33169
A5DB65	4929
B9WI83	573826
C5MJA4	294747
C4YS49	5476
Q5AJY2	5476
A5E395	36914
A3LQP2	4924
Q6BRS8	4959
C4QYI7	644223
Q6CAY2	4952
B2WHE3	426418

A4S8I1	436017	A9TQQ1	145481	B6K5X3	402676
A4SAU4	436017	C1E3E5	296587	A7EU45	665079
A4UYK6	63789	Q01CQ3	70448	Q0U5T0	13684
A5AAL4	425011	A8IAT4	3055	A2QGU4	425011
A5AT84	29760	C1N3E5	564608	A2RAB2	425011
A5BKL2	29760	A4RUC7	436017	A2R373	425011
A5DBM1	4929	D7FQZ4	2880	D5GLK3	39416
A5DED0	4929	B8C486	35128	B8M7R3	441959
A5DYQ1	36914	B7GDA6	556484	A6SA17	332648
A5E3P3	36914	B7S3L8	556484	A1D0V8	331117
A5X2Z8	39946	B8LR30	3332	A1D5V5	331117
A5X2Z9	39946	D0MXI4	403677	A1DHX3	331117
A5X300	39946	Q5KFA0	5207	B6Q502	441960
A5X301	39946	Q96VZ5	5207	Q0CPG9	341663
A5X302	39946	A8NTG0	240176	Q0CZ04	341663
A6MVU2	52970	B0D9T8	486041	Q2UBS1	5062
A6MW39	52970	C5PCI0	222929	Q2UIB0	5062
A6QQT9	9913	Q2U860	5062	B8N7B7	332952
A6QRU8	339724	B8NEC1	332952	B8N7W8	332952
A6R7H6	339724	C1L3C1	5127	Q872F8	5141
A6RUW4	332648	C5GRI3	559297	C7Z247	660122
A6RW42	332648	C7YRV3	660122	A4RNV7	148305
A6ZMG5	307796	Q75CW4	33169	Q2GXE7	38033
A6ZTH5	307796	C4JWT6	336963	B6HUP3	500485
A7EF35	665079	B2B4E9	5145	Q10318	4896
A7EMK2	665079	C6HPI3	544712	B2B590	5145
A7LIU5	28502	B2VW72	426418	B0XTT1	451804
A7TQ79	436907	A1CLE8	5057	Q4X099	5085
A7TSL6	436907	D1ZDQ2	5147	C5FMZ6	554155
A8J1U3	3055	P38674	5141	C5PC55	222929
A8N6M2	240176	C5M977	294747	C4JX57	336963
A8NDY7	240176	A6RUK2	332648	A1C7D3	5057
A8NDZ1	240176	C0NZL2	447093	Q5AZD4	162425
A8PUD2	425265	A7EEW5	665079	C8V0Z3	227321
A8PX99	425265	A2QU08	425011	C5JSR5	559298
A9RF63	145481	P78827	4896	C5GDN4	559297
A9RWH6	145481	B6QVU9	441960	D0MU74	403677
A9S0R7	145481	C1GGJ2	502780	D0P3A5	403677
A9T869	145481	C0SHC8	482561	C1GNB9	502779
B0D8X6	486041	Q4P572	5270	A6R2Z2	339724
B0DQ58	486041	Q0CS25	341663	C0NA31	447093
B0FBK3	75626	B6H313	500485	C1GJ05	502780
B0FBK4	75626	Q5BAA4	162425	A8QAI2	425265
B0FBK5	3880	C8VPK8	227321	D4DK18	663202
B0FBK6	3880	A4RJJ1	148305	A9SVA5	145481
B0JZX6	8364	Q59WW5	5476	A8NSD6	240176

B0XXW9	451804	Q59XR8	5476	C6HKE7	544712
B0Y5R3	451804	D4AX03	663331	Q5KCL8	5207
B1X3H1	39717	D4CZD1	663202	C5YN64	4558
B1X5T2	39717	B9WIG8	573826	A4RUR5	436017
B2AZC0	5145	C9S595	526221	Q6YZH8	39947
B2B064	5145	D5GPG5	39416	A2YY43	39946
B2G4B6	4956	A8Q731	425265	B9RWL5	3988
B2W1S1	426418	Q6CAF8	4952	B8C011	35128
B2WES1	426418	O94135	73868	D2DKF0	3847
B2XT33	536047	B6JVI5	402676	B0CVQ3	486041
B2XT40	536047	A3LUW4	4924	C0P869	4577
B2XTI9	536046	C4XZU7	306902	B4FWX5	4577
B2XTJ6	536046	A5E4Z1	36914	B9H085	3694
B3LLZ9	285006	Q6CY71	28985	B9GMG3	3694
B3LU66	285006	C4QX36	644223	C1MJD7	564608
B3RKI2	10228	C5DNI3	559295	Q9LIR4	3702
B3U3M8	39946	B5VNP5	545124	Q94BS6	3702
B4G0C6	4577	Q0UNT9	13684	D7L507	81972
B5A9S4	50193	P06168	4932	C1DZG4	296587
B5A9S5	50193	D6VYZ3	559292	Q4P2C3	5270
B5LAT2	4072	C8ZDW2	643680	A8IX80	3055
B5VPN3	545124	B3RHM4	285006	B8PA25	561896
B5X226	8030	A7A1N3	307796	C0SBK0	482561
B6H4R2	500485	C7GRY0	574961	D4AJM2	663331
B6HUL3	500485	A7THN4	436907	D1ZAT5	5147
B6HVF8	500485	Q6BLZ4	4959	Q5K661	121759
B6K5L0	402676	B8MTE6	441959	C9SSD1	526221
B6Q1J9	441960	A6RGX3	339724	A8N737	240176
B6Q3W1	441960	Q4WYW4	5085	Q01CC3	70448
B6QBQ1	441960	B0XZX8	451804	A1C7W6	5057
B6TXK4	4577	A1D5X2	331117	A1CQT3	5057
B6U4G3	4577	B4FA74	4577	A1CRV3	5057
B7G2T9	556484	C5DRF8	559307	A1D3P6	331117
B7T1U0	109269	Q6FXG6	5478	A1D4R0	331117
B7T1Y6	109269	A2WTA6	39946	A1DII9	331117
B7ZZM4	4577	B1X3L2	39717	A2QPL6	425011
B8AFH3	39946	B7FLH4	3880	A2R655	425011
B8BJX9	39946	B9DH71	3702	A2RAS2	425011
B8C663	35128	C1GX30	502779	A4R3M5	148305
B8LK99	3332	C4J776	4577	A7EU65	665079
B8LTV0	441959	C5FMW7	554155	A7RMM9	45351
B8M3I0	441959	Q02340	4932	B2AEH4	5145
B8M7J7	441959	Q02341	4932	B6H1J6	500485
B8N1U6	332952	Q56WU3	3702	B6HQ90	500485
B8N9W8	332952			B8NQW9	332952
B8NE74	332952			B8NRS2	332952

B8NPM9	332952
B8QGD3	83154
B8QGD4	83154
B8QGD5	83154
B8QGD8	83154
B8QGD9	83154
B8QGE2	83154
B8QGE4	83154
B8QGE6	83154
B8QGE9	83154
B8QGF0	83154
B8QGF5	83154
B8QGF7	83154
B8QGF8	83154
B8QGF9	83154
B8QGG2	83154
B8QGG3	83154
B8QGG4	83154
B8QGG6	83154
B9DFL8	3702
B9F068	39947
B9F104	39947
B9FEW3	39947
B9HGI0	3694
B9HYF8	3694
B9IFC5	3694
B9NI63	3694
B9RAF1	3988
B9RM03	3988
B9SV00	3988
B9WDK1	573826
B9WFK0	573826
C0HIZ0	4577
C0L093	4097
C0LL34	89411
C0NIU4	447093
C0NU24	447093
C0PJP3	4577
C0PKS4	4577
C0S393	482561
C0SIR4	482561
C1EI66	296587
C1FJR3	296587
C1G117	502780
C1GLR3	502780
C1GTN7	502779

B8NVE1	332952
C4JJJ2	336963
C4Y276	306902
C8VCJ2	227321
C8VF01	227321
Q0V613	13684
Q2TXQ3	5062
Q2UAU5	5062
Q2UU88	5062
Q4WJ40	5085
Q4WK65	5085
Q4WZN7	5085
Q5KEP2	5207
Q7SBQ5	5141
A2QXK2	425011
A4RCZ6	148305
A4UBS4	148305
A6R4L9	339724
A6S9Z9	332648
A9V147	81824
B0D0Q9	486041
B0XN07	451804
B0XPV2	451804
B0XUZ5	451804
B1X452	39717
B2WL59	426418
B6Q2T5	441960
B6QIE5	441960
B6QKY2	441960
B8MKQ3	441959
B8MPX6	441959
B9P5K9	3694
C0NBL4	447093
C0SF90	482561
C1GK23	502780
C1H4T6	502779
C5FT56	554155
C5GD80	559297
C5JQP4	559298
C5P1G6	222929
C6H9W2	544712
C7Z2V6	660122
C7Z808	660122
C7Z9A5	660122
C8V5I0	227321
C9SCM6	526221

C1HAF1	502779
C1N612	564608
C3Y067	7739
C4B808	258217
C4JW49	336963
C4JYW2	336963
C4R141	644223
C4R783	644223
C4Y185	306902
C4Y297	306902
C4YHU4	5476
C5DGD9	559295
C5E1C8	559307
C5E3D1	559295
C5E4D3	559307
C5FDR2	554155
C5FF05	554155
C5G9R2	559297
C5GE50	559297
C5H4D6	316505
C5H4D7	316505
C5H4D9	316505
C5JJ85	559298
C5JY03	559298
C5KW14	423536
C5L187	423536
C5LR47	423536
C5M292	294747
C5MA99	294747
C5P8T8	222929
C5P9U4	222929
C5XSM1	4558
C5XX95	4558
C5Y7K7	4558
C6H1H6	544712
C6H869	544712
C6KIN9	44056
C6KIS8	44056
C6KIX7	44058
C6KJ25	44058
C7GQK9	574961
C7GWA1	574961
C7YPF2	660122
C7Z3H6	660122
C7ZKR1	660122
C8V8Q9	227321
D4AWX6	663331
D5GG74	39416
Q0C814	341663
Q0CC82	341663
Q0CT86	341663
Q0CVG7	341663
Q2H7J0	38033
Q2HCA1	38033
Q2TV70	4905
Q4PA83	5270
Q5AWH2	162425
Q5B2U2	162425
Q5B5X2	162425

C8V916	227321
C8Z476	643680
C8ZEW1	643680
C9E7B6	3885
C9K5V0	309978
C9S7Q1	526221
C9SAN7	526221
D0MX38	403677
D0N068	403677
D1ZA93	5147
D1ZFR7	5147
D2IS67	2898
D3JJ57	3708
D3K640	29788
D4ACG2	10116
D4AMM1	663331
D4AZH5	663331
D4D5L6	663202
D4D8X1	663202
D5G405	39416
D5GK65	39416
D6VR03	559292
D6VZT1	559292
D7LDP8	81972
D7LRY3	81972
D7M833	81972
D7QYK8	29788
D7RPJ7	4113
O13331	148305
O19929	2771
O22026	45157
O22035	2771
O22547	3055
O22578	3067
O49210	3067
O49229	3055
O60086	4896
O61856	6239
O63181	77580
O78451	55529
O78518	55529
P07342	4932
P09114	4097
P09342	4097
P14874	3708
P17597	3702

P25605	4932
P27818	3708
P27819	3708
P36620	4896
P51230	2787
P69683	2787
P69684	2786
P78995	27292
Q00U23	70448
Q00WB5	70448
Q00ZA1	70448
Q01154	148305
Q01LD9	4530
Q05767	3708
Q0CD88	341663
Q0CLE4	341663
Q0CP19	341663
Q0CTB6	341663
Q0E0Z3	39947
Q0TZT5	13684
Q0UJB2	13684
Q0USR1	13684
Q14TG2	280076
Q14TG4	280076
Q1XDF6	2788
Q1XDQ7	2788
Q2GNZ0	38033
Q2GZN0	38033
Q2PDK3	81473
Q2U9N8	5062
Q2UGG1	5062
Q2UL29	5062
Q2WFB4	44972
Q2WFB5	44972
Q2WFB8	44972
Q2WFB9	44972
Q38795	47306
Q3E6W5	3702
Q3EBQ7	3702
Q41716	36591
Q41717	36591
Q41768	4577
Q41769	4577
Q42767	3635
Q42768	3635
Q4P4E2	5270

Q4P9H4	5270
Q4WNW7	5085
Q4WXQ3	5085
Q53L28	39947
Q560J9	5207
Q5AJI0	5476
Q5AJV7	5476
Q5AMJ6	5476
Q5AN02	5476
Q5B3C4	162425
Q5B4V0	162425
Q5K716	5207
Q5KPJ5	5207
Q5VB42	4232
Q5VB43	4232
Q5VB44	4232
Q5VB45	4232
Q5VB46	4232
Q5VB47	4232
Q5VB48	4232
Q5VB49	4232
Q6B8N6	285951
Q6B8Z2	285951
Q6BJI8	4959
Q6BNH8	4959
Q6CCG2	4952
Q6CDI5	4952
Q6CKA0	28985
Q6CVE8	28985
Q6FN19	5478
Q6FS30	5478
Q6K1X7	39947
Q6K2E8	39947
Q6NV04	7955
Q6T858	71324
Q6T859	71324
Q6T860	71324
Q758Q8	33169
Q75ET7	33169
Q7RVY3	5141
Q7X9G4	33128
Q7XKQ8	39947
Q85FZ7	45157
Q85G80	45157
Q8BU33	10090
Q8L7Y7	3702

Q8NJ49	4944
Q93XN5	158579
Q93XN6	124763
Q93YZ7	3702
Q94B64	3702
Q96V04	148305
Q96VZ6	5207
Q9FFF4	3702
Q9FUD0	4521
Q9MS98	130081
Q9P6B4	5141
Q9SKB9	3702
Q9SMC2	4092
Q9TLY1	2771
Q9ZSU3	83154

branched-chain amino acid aminotransferase

UniProt	TXID
A0CQD8	5888
A0CWV8	5888
A0DHX8	5888
A0DP04	5888
A0DQ59	5888
A0E905	5888
A0E931	5888
A1C693	5057
A1CCC2	5057
A1CFM6	5057
A1CGS8	5057
A1CSC6	5057
A1CY02	331117
A1D591	331117
A1DGW8	331117
A2DZ77	5722
A2E498	5722
A2Q939	425011
A2QD81	425011
A2QHM8	425011
A2QTG2	425011
A2QV14	425011
A2WML4	39946
A2XBJ1	39946
A2XE78	39946
A2Y2A9	39946
A2ZA03	39946
A3AD92	39947

2-isopropylmalate synthase

UniProt	TXID
A1CHY9	5057
A1CNV7	5057
A1CWS1	331117
A1D1R7	331117
A2QAX8	425011
A2QJ88	425011
A3GFB4	4924
A3GGR1	4924
A3LU20	4924
A3LUA5	4924
A4QR67	148305
A4RD53	148305
A4RRV6	436017
A5DCX1	4929
A5DDX9	4929
A5DEU6	4929
A5DK19	4929
A5DUL2	36914
A5DVS2	36914
A5DWR8	36914
A5DYI7	36914
A6QYH1	339724
A6RAW0	339724
A6S6B4	332648
A6SEV6	332648
A6ZNV6	307796
A6ZRX6	307796
A6ZXE9	307796

3-isopropylmalate dehydrogenase

UniProt	TXID
A1CNN3	5057
A1CUK0	5057
A1D1I2	331117
A1DP80	331117
A2QB74	425011
A2QKC6	425011
A2XK82	39946
A3AL03	39947
A4QSZ8	148305
A4QUQ9	148305
A4SBH9	436017
A4Z4U6	4911
A5DLX7	4929
A5E7H0	36914
A6QY03	339724
A6RDC6	339724
A6SPE0	332648
A6ZTG8	307796
A7EP53	665079
A7TGM5	436907
A8DWH8	45351
A8I7N4	3055
A8I7N8	3055
A8NYJ8	240176
A8PVM0	425265
A9RIX2	145481
A9YTV4	119927
B0CXS4	486041

A3C731	39947	A6ZXJ7	307796	B0XM71	451804
A3LNY1	4924	A7F9C4	665079	B0XNI9	451804
A3LVU4	4924	A7F9M5	665079	B2AWZ1	5145
A4HFY0	5660	A7TF22	436907	B2WKF7	426418
A4I2W8	5671	A7TNA0	436907	B2ZBW4	27301
A4IFQ7	9913	A8NEF9	240176	B5LAV1	4072
A4R037	148305	A8NU10	240176	B5VET6	545124
A4R2M4	148305	A8PWG4	425265	B6HAM6	500485
A4RDL3	148305	A8QCA5	425265	B6HPJ8	500485
A4RWN3	436017	A9S4K9	145481	B6K4K0	402676
A4S1G0	436017	A9T9B8	145481	B6QBS6	441960
A4SB33	436017	B0D9Z2	486041	B6THR9	4577
A5DGQ7	4929	B0XN47	451804	B6TVD6	4577
A5DUJ5	36914	B0Y6N3	451804	B7FHQ0	3880
A5DY11	36914	B1X5H2	39717	B7G1X8	556484
A6R0Z5	339724	B2AQI3	5145	B8C2I2	35128
A6RH28	339724	B2AUM7	5145	B8M1Y5	441959
A6RI10	332648	B2D157	3712	B8M3W0	441959
A6S587	332648	B2WGW9	426418	B8MZ75	332952
A6SCW1	332648	B2WKN1	426418	B8NXT4	332952
A6SP39	332648	B3LH22	285006	B8PNS1	561896
A6ZQA4	307796	B3LH66	285006	B9GG50	3694
A6ZTB5	307796	B3LJF1	285006	B9SWT3	3988
A7ENI6	665079	B3LNW3	285006	B9WJH1	573826
A7EYY1	665079	B5KJ60	51350	C0NEM8	447093
A7F5D9	665079	B5KJ62	51351	C0NYY5	447093
A7SLW1	45351	B5LAV3	4072	C0PD04	4577
A7TPV1	436907	B5VF91	545124	C0SED7	482561
A7TT81	436907	B5VRZ8	545124	C1EFQ3	296587
A8BLB2	184922	B6HIA0	500485	C1GHD5	502780
A8HT91	3055	B6K1B6	402676	C1H3I5	502779
A8I0S9	3055	B6K7H6	402676	C1L3C2	5127
A8I7X6	3055	B6QAU4	441960	C1MWL6	564608
A8N0V2	240176	B6QAU5	441960	C4JGP0	336963
A8PXJ5	6279	B6QPX9	441960	C4JIA5	336963
A8PZH7	425265	B6SH97	4577	C4R3D0	644223
A8WM75	6238	B6SWM0	4577	C4YB62	306902
A8X039	6238	B6SWN1	4577	C4YTB1	5476
A9JS68	8355	B7FFB9	3880	C5E3C4	559295
A9NN10	3332	B7FLQ1	3880	C5FBH4	554155
A9RS79	145481	B7FUP6	556484	C5GME9	559297
A9SPS3	145481	B7G5N8	556484	C5GYE1	559297
A9SY36	145481	B8A2E9	4577	C5JY74	559298
A9SZC8	145481	B8BJ19	39946	C5K2Z8	559298
A9T3K1	145481	B8BLZ1	39946	C5MFT1	294747
A9U6P0	145481	B8BRI7	296543	C5P3K3	222929

A9UZ24	81824	B8C732	35128	C5P893	222929
B0CPH7	486041	B8LWR9	441959	C5YB10	4558
B0E9E9	370354	B8M2U2	441959	C6H5A5	544712
B0X8N7	7176	B8MZH6	332952	C6HNJ4	544712
B0XRD4	451804	B8N0W8	332952	C6TGV1	3847
B0XS77	451804	B8NK98	332952	C6TKN3	3847
B0Y5G1	451804	B8PD45	561896	C7GQK2	574961
B1X3T0	39717	B9SX30	3988	C7ZET2	660122
B2AAZ8	5145	B9W6H7	573826	C7ZPF5	660122
B2AR54	5145	B9W7P6	573826	C8VJK4	227321
B2B756	5145	B9WBB7	573826	C8VUR0	227321
B2DBI4	66420	B9WCP0	573826	C8Z469	643680
B2KFA0	10090	C0NF36	447093	C9K7B7	5599
B2KFA1	10090	C0NMX2	447093	C9SV85	526221
B2KFA2	10090	C0P8I4	4577	C9SYQ0	526221
B2VTC9	426418	C0S541	482561	D0N196	403677
B2VZK6	426418	C0SDV3	482561	D0V7M9	199306
B2W177	426418	C1FID7	296587	D1ZPB1	5147
B2WPB1	426418	C1G3U9	502780	D4B107	663331
B3H658	3702	C1GHU8	502780	D4DIX5	663202
B3KSI3	9606	C1H3Y8	502779	D5GE49	39416
B3KY27	9606	C1H8B2	502779	D6VQZ6	559292
B3LQM7	285006	C1N4J5	564608	D7KM07	81972
B3LSW9	285006	C4J3S6	4577	D7KX44	81972
B3MXN6	7217	C4JRR1	336963	D7KX45	81972
B3NWN8	7220	C4JY82	336963	D7M613	81972
B3SBH7	10228	C4QWR7	644223	O14429	5478
B4F9X9	4577	C4QYE7	644223	O59930	5306
B4FA16	4577	C4R0L2	644223	O60027	33169
B4FB06	4577	C4XX33	306902	O94114	4924
B4FD32	4577	C4XZ37	306902	P04173	4932
B4FQ91	4577	C4Y5K7	306902	P07139	5479
B4H2W7	7234	C4YAG2	306902	P08791	4903
B4IGS4	7238	C4YFW0	5476	P18120	4952
B4JLQ2	7222	C4YKP2	5476	P18869	4896
B4L1S1	7230	C5DGX9	559295	P23390	28985
B4MEI3	7244	C5DII8	559295	P29102	3708
B4NEH1	7260	C5DP09	559307	P34733	4905
B4NVP8	7240	C5DRM6	559307	P34738	5141
B4Q2B4	7245	C5DT42	559307	P41766	4911
B5VLT5	545124	C5DTL1	559307	P41926	34356
B5X837	8030	C5E265	559295	P87186	5476
B6GY28	500485	C5FUR8	554155	P87256	5061
B6HJE5	500485	C5FUW4	554155	P87257	5061
B6HR72	500485	C5GFR2	559297	P93832	3702
B6HRY8	500485	C5GKV1	559297	Q00VM0	70448

B6HU88	500485
B6K620	402676
B6KTN2	508771
B6Q223	441960
B6QES1	441960
B6QEX8	441960
B6QEX9	441960
B6QUE3	441960
B6RFK8	4100
B6STR2	4577
B6THP1	4577
B7FJP0	3880
B7FT57	556484
B7FTZ3	556484
B7P5K8	6945
B7Z2M5	9606
B7Z5L0	9606
B8AFD4	39946
B8ASX0	39946
B8AWI0	39946
B8AZW6	39946
B8BY53	35128
B8LQL0	3332
B8LUG0	441959
B8LZT6	441959
B8MBA9	441959
B8MBB0	441959
B8MIC4	441959
B8MQ34	441959
B8MYP9	332952
B8N7W4	332952
B8NA84	332952
B8NB44	332952
B8NC67	332952
B9DHH5	3702
B9EUL1	39947
B9F6M2	39947
B9FBX6	39947
B9FNG3	39947
B9FNG4	39947
B9GNK4	3694
B9HFT9	3694
B9HN46	3694
B9HZQ8	3694
B9IKL7	3694
B9Q185	5811
C5H9M9	51351
C5J4N7	3707
C5J4N9	3707
C5J4P1	3707
C5JII3	559298
C5K0P5	559298
C5M530	294747
C5M6W3	294747
C5MDZ9	294747
C5MF43	294747
C5PAF9	222929
C5PCV6	222929
C5Y4G6	4558
C5YQI8	4558
C6H2L0	544712
C6HP01	544712
C7GMM8	574961
C7YJ23	660122
C7YV47	660122
C8VL66	227321
C8VQ30	227321
C8Z6C2	643680
C8Z6H2	643680
C8ZGC6	643680
C8ZI69	643680
C9SFE9	526221
C9SML8	526221
C9SR17	526221
D0MYD7	403677
D0VY45	34305
D1ZKQ0	5147
D2UXY9	5762
D4B0A2	663331
D4D4U1	663202
D4DIY3	663202
D5GJ82	39416
D5GJS6	39416
D5KUG5	4932
D6VRH0	559292
D6VRL8	559292
D6W175	559292
D6W2G7	559292
D7KGE8	81972
D7KRQ1	81972
D7M1L9	81972
O04973	28526
Q01987	5477
Q0CM68	341663
Q0CSC7	341663
Q0DPW7	39947
Q12545	5044
Q12590	5479
Q12591	5479
Q12592	5479
Q2HAA2	38033
Q2TYA5	5062
Q2UQS4	5062
Q2V379	3702
Q2V380	3702
Q4PF68	5270
Q4WLG7	5085
Q4WRM6	5085
Q5AFI8	5476
Q5B9I7	162425
Q5BEW8	162425
Q5F0I0	5479
Q5KP37	5207
Q5XF32	3702
Q6B458	4959
Q6PY58	51915
Q6TWC4	5147
Q7Y096	39947
Q877A9	5062
Q8GXH6	3702
Q8LB97	3702
Q8LEF4	3702
Q8NKB8	37620
Q96WI0	559307
Q96WT9	34358
Q9FMT1	3702
Q9HDQ1	4927
Q9HDQ5	5481
Q9P3Y0	4954
Q9P8C1	13684
Q9SA14	3702
Q9Y897	54734

B9QHR5	432359	O04974	28526
B9R6T8	3988	O59736	4896
B9R6T9	3988	O94225	500485
B9R6U0	3988	P06208	4932
B9RS41	3988	P48570	4932
B9S7U2	3988	Q01FR2	70448
B9SBB9	3988	Q0CZH8	341663
B9SX80	3988	Q0CZN6	341663
B9TIE0	3988	Q0IUQ0	39947
B9WB98	573826	Q0UH08	13684
B9WEE3	573826	Q0V1B4	13684
C0HAX7	8030	Q12122	4932
C0HF14	4577	Q12166	4932
C0LAM2	5341	Q12726	4952
C0NIH3	447093	Q1JRZ0	81972
C0NQG3	447093	Q1JRZ1	81972
C0P6R1	4577	Q1JRZ2	81972
C0P886	4577	Q1JRZ3	115915
C0PA18	4577	Q1JRZ4	115915
C0PTG0	3332	Q1JRZ5	115915
C0S9L5	482561	Q1JRZ6	97979
C0SFX8	482561	Q1JRZ7	97979
C1EDP6	296587	Q1JRZ8	97979
C1GC48	502780	Q1JRZ9	59691
C1GL38	502780	Q1JS00	59691
C1H0V7	502779	Q1JS01	59691
C1H5W2	502779	Q2H478	38033
C1MRQ3	564608	Q2QXY9	39947
C1MXY2	564608	Q2RAP8	39947
C3Z1L6	7739	Q2U786	5062
C4JP84	336963	Q2UJL3	5062
C4JT41	336963	Q2UQI7	5062
C4JUC0	336963	Q30DX9	69183
C4LSR8	5759	Q39891	3847
C4R7A4	644223	Q4P6V7	5270
C4XWX3	306902	Q4P6Z8	5270
C4YIA7	5476	Q4WPU2	5085
C5DFQ6	559295	Q4WRV1	5085
C5E1Q1	559307	Q59TC4	5476
C5FF64	554155	Q59VG1	5476
C5FWI3	554155	Q59YF1	5476
C5G0G2	554155	Q5AB86	5476
C5G9D5	559297	Q5BBU0	162425
C5GC46	559297	Q5BF40	162425
C5H9M5	51351	Q5FAL5	3712
C5H9M6	51351	Q5KCL7	5207

C5JN83	559298	Q5KIZ5	5207
C5JPW9	559298	Q60D83	3712
C5KPI8	423536	Q60D84	3712
C5L4N6	423536	Q6B1Y9	4932
C5LE91	423536	Q6BM39	4959
C5LSR3	423536	Q6BRX1	4959
C5M6U4	294747	Q6BW96	4959
C5MJG1	294747	Q6BY18	4959
C5NZB0	222929	Q6CFF6	4952
C5P8J6	222929	Q6CIW1	28985
C5XJI9	4558	Q6CL60	28985
C5YDX1	4558	Q6CM17	28985
C5YVA1	4558	Q6CQU3	28985
C6H7T8	544712	Q6FNT3	5478
C6HS95	544712	Q6FP59	5478
C6M0D6	598745	Q6FSZ1	5478
C6T8V8	3847	Q6FT78	5478
C6TLT9	3847	Q71QG1	3712
C7GN93	574961	Q755P2	33169
C7GPU1	574961	Q75A20	33169
C7YVH8	660122	Q75AD2	33169
C7Z035	660122	Q7RVT2	5141
C7Z586	660122	Q7SFM2	5141
C7Z616	660122	Q8VX04	3702
C8V3E5	227321	Q9C550	3702
C8V3Y1	227321	Q9FG67	3702
C8V3Y3	227321	Q9FN52	3702
C8V969	227321	Q9LPR4	3702
C8VTP8	227321	Q9Y823	4896
C8ZA09	643680		
C8ZBU8	643680		
C9STB5	526221		
C9ZJG3	679716		
C9ZJG4	679716		
D0NRA1	403677		
D1Z3T0	5147		
D2VAR7	5762		
D3BSA4	670386		
D3DLF7	559292		
D4B2E4	663331		
D4B2S3	663331		
D4DG90	663202		
D5GAR6	39416		
D5GAR7	39416		
D5GD45	39416		
D5GN05	39416		

D6VWW6	559292
D6WAG1	7070
D7KFV4	81972
D7KFV6	81972
D7KFV8	81972
D7KKL8	81972
D7LA26	81972
D7LSM8	81972
D7MNW6	81972
D7MUM0	81972
O14370	4896
O15382	9606
O35854	10116
O35855	10090
O88374	10090
P24288	10090
P38891	4932
P47176	4932
P54687	9606
P54688	6239
P54690	10116
Q013H3	70448
Q01AA4	70448
Q01H95	4530
Q0CHM6	341663
Q0CN11	341663
Q0CPA6	341663
Q0DJP6	39947
Q0E243	39947
Q0P4T8	8364
Q0UGM1	13684
Q0UN77	13684
Q0UT06	13684
Q0UX59	13684
Q0V7U6	3702
Q0WQT2	3702
Q0WUX7	3702
Q0WW34	3702
Q0WW83	3702
Q10PK7	39947
Q10PK8	39947
Q10PL0	39947
Q170J5	7159
Q29IR8	46245
Q2GSR0	38033
Q2HEX6	38033

Q2U042	5062
Q2U1F9	5062
Q2UG50	5062
Q2UIB4	5062
Q2UR77	5062
Q2V4P2	3702
Q3TJN1	10090
Q3TKJ1	10090
Q3ULU3	10090
Q4FYB5	347515
Q4P2X7	5270
Q4PAT0	5270
Q4PIE8	5270
Q4WKQ2	5085
Q4WNL4	5085
Q4X1C8	5085
Q54N47	44689
Q55HM3	5207
Q586R0	5691
Q586R1	5691
Q59YS9	5476
Q5AHJ9	5476
Q5AHX4	5476
Q5AV02	162425
Q5AV04	162425
Q5B0H3	162425
Q5B557	162425
Q5BGE5	162425
Q5DQ95	4081
Q5E9U7	9913
Q5EA40	9913
Q5K761	5207
Q5NAM3	39947
Q5REP0	9601
Q5RHB8	7955
Q5W706	39947
Q640F5	8355
Q68DQ7	9606
Q6AT43	39947
Q6BSQ6	4959
Q6CAN4	4952
Q6CX88	28985
Q6DG83	7955
Q6DIN6	8364
Q6FK92	5478
Q6FTS6	5478

Q6K7U8	39947
Q6P784	10116
Q6PC10	7955
Q70KX9	4513
Q75BE8	33169
Q7QEI9	7165
Q7S699	5141
Q7SFT9	5141
Q7XPR5	39947
Q86QZ8	5741
Q8CAX1	10090
Q8CBC8	10090
Q8GRH8	39947
Q8H7T7	39947
Q8L493	3702
Q94CF8	4072
Q94LV4	39947
Q99JD5	10116
Q9CXX6	10090
Q9FYA6	3702
Q9GKM4	9940
Q9HEB7	5141
Q9LE06	3702
Q9LPM8	3702
Q9LPM9	3702
Q9M401	3702
Q9M439	3702
Q9SNY8	4113
Q9SNY9	4113
Q9VYD5	7227
Q9XXD8	6239

**3-isopropylmalate dehydratase**		**3-isopropylmalate dehydratase/(R)-2-methylmalate dehydratase large subunit**		**3-isopropylmalate dehydratase/(R)-2-methylmalate dehydratase small subunit**	
**UniProt**	**TXID**	**UniProt**	**TXID**	**UniProt**	**TXID**
P07264	4932	Q94AR8	3702	Q9ZW85	3702
D6VUC8	559292	D7MV68	81972	D7LJX7	81972
B3LIM4	285006	B9SQS5	3988	B9SXE3	3988
A6ZUY4	307796	B9HSU8	3694	B5LAV2	4072
C8Z8N4	643680	B9HKV5	3694	A9PJA7	3695
C7GK64	574961	Q6Z702	39947	A5B9M3	29760
Q6FXV1	5478	B8AGY8	39946	A9NMS1	3332
A7TKW5	436907	C5XT62	4558	C0PQ56	3332
C5DHW7	559295	C4JBG7	4577	B6SZA5	4577
Q6CXX1	28985	C4IZQ5	4577	B6UGK8	4577

C4R0G5	644223	B6TYJ6	4577	A8HYG8	3055
C5E063	559307	B6U6T6	4577	D5LB14	3055
B9WKN6	573826	B4FG11	4577	Q4PLC1	33097
Q6BUD7	4959	A9TGU1	145481	C5XRZ8	4558
Q74ZM9	33169	B8LQY6	3332	Q949D1	4530
C4YAQ8	306902	B8LQ24	3332	Q6URQ0	39947
Q5AAJ1	5476	A8JG03	3055	Q6H6I1	39947
C4YND7	5476	Q6B920	285951	A2X7X9	39946
C5M4Q5	294747	D7G5W8	2880	A1C6H0	5057
A5DCZ2	4929	A1C6H0	5057	A1DH42	331117
A3GH13	4924	A1DH42	331117	A2QCE1	425011
Q00464	5479	A2QCE1	425011	A3GH13	4924
Q6CG29	4952	A3GH13	4924	A4RKB1	148305
A5DRP1	36914	A4RKB1	148305	A4RQI6	436017
B8M3Q6	441959	A4RQI6	436017	A5DCZ2	4929
B6QPH5	441960	A5DCZ2	4929	A5DRP1	36914
Q7RZ87	5141	A5DRP1	36914	A6RBU6	339724
B2AZG8	5145	A6RBU6	339724	A6ZUY4	307796
C5JE09	559298	A6SLR4	332648	A7TKW5	436907
C5GPC4	559297	A6ZUY4	307796	A8NY17	240176
P55251	4840	A7EWS8	665079	A8PWL7	425265
D1ZFL4	5147	A7TKW5	436907	B0CYV1	486041
Q5B0P4	162425	A8NY17	240176	B0XSF6	451804
C8UZW6	227321	A8PWL7	425265	B1X4T5	39717
Q0U3J2	13684	A9PBQ8	3694	B2AZG8	5145
B6H6J7	500485	B0CYV1	486041	B2WMF3	426418
C6H331	544712	B0E3H5	486041	B3LIM4	285006
C0NNE2	447093	B0XSF6	451804	B6H6J7	500485
Q2UEQ5	5062	B1X4T4	39717	B6K3Q2	402676
B8NG70	332952	B2AZG8	5145	B6QPH5	441960
C5PJM4	222929	B2WMF3	426418	B7FPZ8	556484
C1G7L2	502780	B3LIM4	285006	B8CEE0	35128
B6K3Q2	402676	B6H6J7	500485	B8M3Q6	441959
C5FFF4	554155	B6K3Q2	402676	B8NG70	332952
Q0CXY4	341663	B6QPH5	441960	B9WKN6	573826
B2WMF3	426418	B7FPZ8	556484	C0NNE2	447093
Q4X144	5085	B8CEE0	35128	C1FEI6	296587
B0XSF6	451804	B8M3Q6	441959	C1G7L2	502780
C9S7W0	526221	B8NG70	332952	C1GY13	502779
O14289	4896	B9WKN6	573826	C1MKS1	564608
C7Z4P2	660122	C0NNE2	447093	C4JG58	336963
A1DH42	331117	C1FEI6	296587	C4R0G5	644223
A2QCE1	425011	C1G7L2	502780	C4YAQ8	306902
D4AYR1	663331	C1GY13	502779	C4YND7	5476
D4DJK6	663202	C1MKS1	564608	C5DHW7	559295
D5GE77	39416	C4JG58	336963	C5E063	559307

A1C6H0	5057
A4RKB1	148305
C4JG58	336963
P18250	4837
A8NY17	240176
Q2H013	38033
Q5KPM3	5207
P55811	4844
C1GY13	502779
P49601	5270
B0CYV1	486041
A8PWL7	425265
A4RQI6	436017
C1FEI6	296587
C1MKS1	564608
A6RBU6	339724
B8CEE0	35128
B7FPZ8	556484
P17279	4841
Q01CM0	70448
A6SLR4	332648
A6SLR5	332648
A7EWS8	665079
A7EWS9	665079
B0DTJ7	486041
B0E3H5	486041
Q2GMU0	38033
A8JG03	3055
A9TGU1	145481
B4FG11	4577
B6TYJ6	4577
B9HKV5	3694
B9HSU8	3694
B9SQS5	3988
C5XT62	4558
Q6Z702	39947
Q94AR8	3702
A9PBQ8	3694
B1X4T4	39717
B6U6T6	4577
B8AGY8	39946
B8LQ24	3332
B8LQY6	3332
C4IZQ5	4577
C4JBG7	4577
D7MV68	81972

C4R0G5	644223
C4YAQ8	306902
C4YND7	5476
C5DHW7	559295
C5E063	559307
C5FFF4	554155
C5GPC4	559297
C5JE09	559298
C5M4Q5	294747
C5PJM4	222929
C6H331	544712
C7GK64	574961
C7Z4P2	660122
C8UZW6	227321
C8Z8N4	643680
C9S7W0	526221
D4AYR1	663331
D4DJK6	663202
D5GE77	39416
D6VUC8	559292
O14289	4896
P07264	4932
P17279	4841
P18250	4837
P49601	5270
P55251	4840
P55811	4844
Q00464	5479
Q01CM0	70448
Q0CXY4	341663
Q0U3J2	13684
Q2GMU0	38033
Q2H013	38033
Q2UEQ5	5062
Q4X144	5085
Q5AAJ1	5476
Q5B0P4	162425
Q5KPM3	5207
Q6BUD7	4959
Q6CG29	4952
Q6CXX1	28985
Q6FXV1	5478
Q74ZM9	33169
Q7RZ87	5141

C5FFF4	554155
C5GPC4	559297
C5JE09	559298
C5M4Q5	294747
C5PJM4	222929
C6H331	544712
C7GK64	574961
C7Z4P2	660122
C8UZW6	227321
C8Z8N4	643680
C9S7W0	526221
D4AYR1	663331
D4DJK6	663202
D5GE77	39416
D6VUC8	559292
D7LW92	81972
O14289	4896
P07264	4932
P17279	4841
P18250	4837
P49601	5270
P55251	4840
P55811	4844
Q00464	5479
Q0CXY4	341663
Q0U3J2	13684
Q2GMU0	38033
Q2H013	38033
Q2UEQ5	5062
Q4X144	5085
Q5AAJ1	5476
Q5B0P4	162425
Q5KPM3	5207
Q6B919	285951
Q6BUD7	4959
Q6CG29	4952
Q6CXX1	28985
Q6FXV1	5478
Q74ZM9	33169
Q7RZ87	5141
Q9LYT7	3702

Q6B920	285951
A5B9M3	29760
A8HYG8	3055
B6SZA5	4577
B9SXE3	3988
C5XRZ8	4558
Q6H6I1	39947
Q9ZW85	3702
A2X7X9	39946
A9NMS1	3332
A9PJA7	3695
B1X4T5	39717
B5LAV2	4072
B6UGK8	4577
C0PQ56	3332
D5LB14	3055
D7LJX7	81972
D7LW92	81972
Q4PLC1	33097
Q6B919	285951
Q6URQ0	39947
Q949D1	4530
Q9LYT7	3702

**homocitrate synthase**		**homoaconitate hydratase**		**homoisocitrate dehydrogenase**	
**UniProt**	**TXID**	**UniProt**	**TXID**	**UniProt**	**TXID**
Q12122	4932	P49367	4932	P40495	4932
D6VRL8	559292	D6VSL5	559292	D6VVJ3	559292
C8Z6H2	643680	C7GVB9	574961	C8ZAF6	643680
B3LH22	285006	B3LG54	285006	C7GKM1	574961
A6ZXJ7	307796	C8Z5E5	643680	B5VKL3	545124
Q6CL60	28985	A6ZYI7	307796	B3LTU1	285006
Q6FNT3	5478	C5DI88	559295	Q6FTG9	5478
C5E265	559295	Q6CT61	28985	C5DPB3	559307
A7TF22	436907	Q6FM51	5478	C5DMN7	559295
C5DTL1	559307	A7THY8	436907	Q6CP13	28985
Q75A20	33169	C5DQB1	559307	A7TLZ7	436907
A5DWR8	36914	A3LS85	4924	Q75AY4	33169
A5DUL2	36914	C4R8Y9	644223	A6ZVI3	307796
B9W7P6	573826	A5DDR9	4929	Q6BTP8	4959
B9WBB7	573826	Q5A644	5476	C5M4F5	294747
C4XZ37	306902	C4YH28	5476	Q5A9D9	5476
C4YAG2	306902	C5MCG0	294747	C4YNM9	5476
A5DDX9	4929	B9WGA9	573826	B9WKX4	573826
C5M6W3	294747	Q2HZ33	5480	A5DS53	36914
C5MF43	294747	Q6BM98	4959	A3M0H4	4924

Q6BM39	4959	Q6C791	4952	C4R2N2	644223
Q6BRX1	4959	C4XXK3	306902	D5GNF9	39416
Q59VG1	5476	A5E7S0	36914	Q0CRJ3	341663
Q59TC4	5476	Q75DX9	33169	A1DME0	331117
C4QWR7	644223	Q9UT74	4896	D1Z7V4	5147
C4QYE7	644223	D5GP05	39416	C4JF94	336963
A3LUA5	4924	B2B627	5145	Q6C9K0	4952
B6K7H6	402676	C8V0K3	227321	A2R529	425011
D5GJS6	39416	B6JYB3	402676	Q7SH15	5141
Q9Y823	4896	Q870W1	5141	C0S5V1	482561
Q5BBU0	162425	Q92412	162425	C1G2X0	502780
C8VL66	227321	C5P2A9	222929	B6HCD5	500485
A2QJ88	425011	A4QU78	148305	B0Y7C8	451804
Q4WPU2	5085	D4D9Z3	663202	C5FFW5	554155
B0Y6N3	451804	D4AIQ8	663331	C5PJ88	222929
A1CWS1	331117	Q2U9G3	5062	Q4WN70	5085
Q0CZH8	341663	B6H7G0	500485	B8MWA5	332952
C6H2L0	544712	B1P2N4	5076	B6QLL4	441960
C0NMX2	447093	C0SGP6	482561	C1H6J6	502779
A1CHY9	5057	Q58FL6	425011	C0NXP5	447093
C5PCV6	222929	B8ND30	332952	Q2UPP6	5062
Q2UJL3	5062	C1GXN8	502779	B8MGT9	441959
Q2U786	5062	C1GGZ6	502780	B2WQ48	426418
C4JRR1	336963	Q2GN26	38033	C5K3H3	559298
B8LWR9	441959	C5FRR7	554155	C5GWA1	559297
C5GFR2	559297	Q4HVQ9	5518	O14104	4896
C5JII3	559298	B2W2S7	426418	C6H8L2	544712
B8N0W8	332952	A6SLA2	332648	B2AB72	5145
B8NK98	332952	Q0V4D3	13684	A6RDS7	339724
O94225	500485	A7E6V1	665079	Q6DTN1	162425
B2AQI3	5145	C6HGU3	544712	C8VF81	227321
B6QPX9	441960	C0NZQ0	447093	A1CT29	5057
A6RAW0	339724	A6R963	339724	Q0V4L7	13684
C0S541	482561	B6QV43	441960	A4RK98	148305
C1H8B2	502779	A1CAH1	5057	B6JVT5	402676
Q0UH08	13684	B8MSQ2	441959	A6RLD8	332648
B2WGW9	426418	Q4WUL6	5085	A7EB51	665079
D4D4U1	663202	B0Y440	451804	Q2HEK1	38033
D1ZBU6	5147	A1DEU5	331117	D4APM6	663331
D4B0E9	663331	D1ZLH5	5147	D4D3K3	663202
C5FUR8	554155	Q0CKV7	341663	Q4P4U0	5270
D2JWV4	5208	D6RM87	240176	Q55U60	5207
Q7RVT2	5141	B0D9W3	486041	Q5KI84	5207
B4VKF4	118168	C9S550	526221	A8NWK9	240176
D1CIS7	525904	Q5K9V9	5207	B0CUW7	486041
Q2IHS7	290397	A8Q727	425265	A8Q6L7	425265

B8JCA9	455488	Q4P521	5270	C9SEZ4	526221
B4UFB7	447217	C7Z6F8	660122	D2VFR4	5762
Q9RUZ2	1299	C7ZFG6	660122	D7BEI7	526227
B8G9T8	326427	D2VQI0	5762	Q8RQU4	274
B9LI57	480224	Q1IMD4	204669	Q72IW9	262724
A9WD10	324602	C5GXJ3	559297	Q5SIJ1	300852
Q1IYY1	319795	C5K3L0	559298	B7A5P1	498848
A9B804	316274	A9UZ48	81824	B5U8Z6	271
Q4J989	2285	C4JP58	336963	Q8TX93	2320
Q4JA78	2285	D1B6F6	525903	Q8TW22	2320
A0B9D1	349307	D1B740	525903	D7CVN0	649638
A0B8Q2	349307	D1B6F4	525903	D3PNU1	504728
A0B5D7	349307	B8E2Q9	515635	D5E914	547558
A4YHL8	399549	B8E2X0	515635	D5VRX3	573063
A4YEE6	399549	B5IEF0	439481	D5VQV4	573063
C7P6A0	573064	B5IER5	439481	C7P8K1	573064
C7P602	573064	B5I9M1	439481	C7P893	573064
C7P7J6	573064	C0WAT0	563191	D3S5L2	644281
A6UVA9	419665	C0WBH9	563191	D3S6U8	644281
A6UUT7	419665	B5YA67	309799	A5US63	357808
A6UVQ0	419665	B5YEF5	309799	Q8PZ52	2209
C4KGW9	426118	C9RD99	429009	Q58130	2190
C4KKB2	426118	C9RAX0	429009	Q58991	2190
C3NHU6	419942	Q6L0K5	263820	Q2NE99	339860
C3NMC8	419942	Q6KZ03	263820	Q2NGI1	339860
C3NDV7	439386	D4KGT7	657316	C9RFZ3	579137
C3N935	439386	D4KG72	657316	C9RGA8	579137
C3N5A3	427318	C8X2U8	485915	A9B807	316274
C3N144	427318	D3SL46	638303	A0B7H0	349307
C3MYM1	427317	D3SQ81	638303	O29627	2234
C3MU56	427317	D4UP39	246199	Q6LYV7	39152
C3MPM7	429572	D4UJU7	246199	Q6LZT4	39152
D2PJS1	425944	D6SS75	555779	O26286	187420
D2PEP7	425944	Q1Q122	174633	O27441	187420
A4G035	402880	B4U7U5	380749	A6VFH2	426368
A4FXB6	402880	B4U832	380749	A6VJJ0	426368
A4FXG1	402880	C2KW91	585501	B9LI60	480224
Q6M0W5	39152	A1HLS2	401526	A9WD13	324602
Q6LYC8	39152	D1Z2Z1	304371	A9AB63	444158
Q6LYH3	39152	D1Z011	304371	A9A765	444158
D3E1C0	634498	D1YYL1	304371	D3E2X3	634498
D3E403	634498	A8UWH6	392423	D3DYN5	634498
D3E0J1	634498	A8UUM9	392423	A4FXR5	402880
B5YA66	309799	C0EDN6	537013	A4FYT7	402880
B5YEF4	309799	C0EH67	537013	D7E7V1	644295
A9A8E1	444158	D4MII6	717961	B8G9T5	326427

A9AAN5	444158	B0MLF2	428128	C7NTB6	519442
A9AAT0	444158	C0R088	565034	A6UX38	419665
B8E2Q8	515635	D3LVK7	699218	A6UWX0	419665
B8E2W9	515635	D3RWK9	589924	A6UN83	406327
D4TDM3	533240	D3S2A2	589924	A6USW3	406327
D2RHN2	572546	D5U4B3	526224	D1Z013	304371
D2RHQ3	572546	D4JF94	717960	A7NPM5	383372
A6TMV5	293826	D4JEG8	717960	Q46FW0	269797
A6TTX4	293826	D4JQP8	657319	B9AH59	483214
A6TTR8	293826	D4XYJ8	552811	B9AFU0	483214
A6VIE3	426368	D4XXV9	552811	Q8TJN2	2214
A6VG10	426368	A8ABG2	453591	Q1IYD1	319795
A6VFV4	426368	A8AB09	453591	D2RGM3	572546
Q97ZE0	2287	D1C1T4	479434	D2ZMH5	521002
Q97W36	2287	D1C5D4	479434	D2ZNT2	521002
D0KTU9	555311	A0B7V1	349307	Q2FKU1	323259
D0KMX5	555311	A0B7A3	349307	A5UK50	420247
D3T718	580331	D1U9X6	643562	A5UMS5	420247
D3T543	580331	D1U8I0	643562	Q0W1Q6	351160
A5CZ95	370438	C9LTJ0	546271	D7DTJ0	456320
A5D4W0	370438	Q9WYC7	2336	D2RXB1	543526
Q8RCF9	119072	Q9WZ24	2336	A8AB60	453591
Q8RDK3	119072	Q9UZ07	29292	B3TCK6	455613
B8CW93	373903	Q9V1J0	29292	C1D1S7	546414
B8CX21	373903	D3L1B9	592015	B3T5X6	455578
D7ASQ9	583358	D3L117	592015	B8GDU3	521011
D7AQD3	583358	D3L5P1	592015	Q9RTT2	1299
D7DQU6	456320	D3L0J8	592015	Q4JB37	2285
D7DTR3	456320	B7RDD0	443254	A8MDZ3	397948
D7DS06	456320	B7RB96	443254	A9A4I7	436308
D5XD93	635013	A5IKB8	390874	A4YCW3	399549
D5XCE4	635013	A5IJM1	390874	Q028N1	234267
D5XAF0	635013	D2C7S7	590168	P50455	111955
O27525	187420	D2C651	590168	A8TZR8	331869
O26819	187420	B1L9K1	126740	A8TLN2	331869
O27667	187420	B1L8U1	126740	A7I5V9	456442
C7IRR0	589861	D2RHT2	572546	A2SQ05	410358
C7IRE6	589861	D2RHD9	572546	Q9UXB2	2287
C7HMQ2	588857	D3E0J0	634498	D0KTC8	555311
C7HNL3	588857	D3DYN7	634498	D2PC68	425944
C5UBV2	509193	O27668	187420	C4KHF3	426118
C5UAT5	509193	O27439	187420	C3NHB2	419942
C5RRT7	573062	B9KC23	309803	C3NEE0	439386
C5RSG9	573062	B9KB94	309803	C3MVH5	427317
B0KCA2	340099	B1I3K5	477974	C3MQ57	429572
B0KAH4	340099	B1I1Y2	477974	Q3IRQ6	348780

B0K353	399726
B0K0Y1	399726
Q64A78	286720
C9RD98	429009
C9RAW9	429009
Q8THA5	2214
Q8TJJ1	2214
Q8TKQ6	2214
P58968	2209
P58966	2209
P58967	2209
Q74EM5	35554
Q74BX2	35554
D7AH81	663917
D7AKA3	663917
Q46DX8	269797
Q46FZ1	269797
Q46AH2	269797
Q2RH53	264732
Q2RG97	264732
A0AK92	386043
A0AWZ9	331272
A0JX36	290399
A0K933	331272
A0KAN5	331272
A0KGM9	380703
A0KLS9	380703
A0L1R0	94122
A0L629	156889
A0L692	156889
A0LBW3	156889
A0LF05	335543
A0LSS5	351607
A0LT07	351607
A0M365	411154
A0PVE6	362242
A0Q408	401614
A0Q9T9	243243
A0R5Q2	246196
A0RBL2	412694
A0RQK7	360106
A0ZZP3	367928
A1A7C2	405955
A1AL90	338966
A1AWA1	413404
A1B9A2	318586
D4RX38	511680
Q0QLE2	1528
A4J6Z5	349161
A4J182	349161
B2V844	436114
C0QS35	123214
A9BIA6	403833
A9BGP4	403833
Q30WD3	207559
B2KBD7	445932
O28084	2234
O28316	2234
Q8TQZ3	2214
Q8TLF1	2214
A5CZ94	370438
A5D4W1	370438
Q2NE97	339860
Q2NFC0	339860
C1DUH4	204536
C7LW15	525897
D4LZ50	657313
D4LZ48	657313
C6PDK3	580327
C4V4K3	638302
D4S5Q9	585503
D6TDR7	485913
D6TMS0	485913
C4FI11	432331
D5E9U8	547558
D5E882	547558
D2BHR4	311424
D2BGU6	311424
Q46AJ7	269797
Q46B45	269797
C4FRW1	546273
D6KKJ9	457416
C8WM14	479437
A4XJ48	351627
A4XHI4	351627
Q3Z894	243164
Q3Z9A7	243164
C3XG48	613026
B9MLV4	521460
B9MNX1	521460
D1BQH1	479436
D4L5N8	657321
B1L5I7	374847
C3N5S4	427318
Q8U299	2261
A3CT70	368407
A5DKS4	4929
A5DKS5	4929

A1B9D4	318586	D4L7L8	657321
A1BES0	290317	D2ZNG5	521002
A1BES3	290317	D2ZNT0	521002
A1BJ89	290317	B9AG62	483214
A1CNV7	5057	B9AFU3	483214
A1D1R7	331117	C6LJ75	478749
A1JJH7	393305	Q7M9Z9	844
A1KAC3	62928	C5EVL2	457421
A1KBD3	62928	C5EE69	457421
A1KQ42	410289	C5EK99	457421
A1KTV6	272831	C5EVU7	457421
A1R6V4	290340	D1YNY3	686660
A1REY0	351745	Q8PUG1	2209
A1RU85	384616	Q8PZT3	2209
A1RVH5	384616	A7VNU8	428125
A1RVI1	384616	A7VWI4	428125
A1S2E0	326297	C0CLF7	476272
A1SI02	196162	A5UL50	420247
A1SM31	196162	A5UMS7	420247
A1SRG8	357804	C4G1B4	592010
A1SZX5	357804	Q8TVF2	2320
A1TGI1	350058	Q8TW29	2320
A1TI48	397945	B8J4F9	525146
A1TRT4	397945	D2RND4	591001
A1U1E7	351348	D2RMM0	591001
A1U3I3	351348	D6KRX7	450749
A1UMW8	189918	D5EGY8	572547
A1UZQ2	320388	D5ECG6	572547
A1V2K5	320388	D6E7M6	657308
A1VAE8	391774	C6PQI2	536227
A1VCV3	391774	Q2RG98	264732
A1VN06	365044	Q2RHA7	264732
A1W1X2	354242	C0B4V9	470146
A1W6T6	232721	C0B4W1	470146
A1W6T7	232721	D2L8F5	644968
A1WNY5	391735	D2L2I7	644968
A1WUW0	349124	Q18AJ2	272563
A2BRP4	146891	Q18A28	272563
A2BX52	167542	A4FW44	402880
A2C3L7	167555	A4FZC1	402880
A2C859	59922	A6UQC3	406327
A2QAX8	425011	A6URZ3	406327
A2S698	412022	A6UUR4	419665
A2SHL8	420662	A6VH65	426368
A2SQ02	410358	A6VJ16	426368
A2SR21	410358	A9A6R8	444158

A3CMI9	388919	A9A9I4	444158
A3CT66	368407	C7P5Q8	573064
A3CW48	368407	C7P908	573064
A3CZK5	325240	C9RG34	579137
A3DF94	203119	C9RHV4	579137
A3DIE2	203119	D3S8I8	644281
A3GFB4	4924	Q58409	2190
A3M1Y3	400667	Q58667	2190
A3MQ98	320389	Q6LX72	39152
A3MVJ7	410359	Q6M090	39152
A3MWJ6	410359	A2SR25	410358
A3MWK4	410359	A3CWX5	368407
A3MZB1	416269	A4XJ49	351627
A3N5I3	320373	A4YF02	399549
A3N7K7	320373	A5FS16	216389
A3NR79	357348	A5IKB7	390874
A3NT94	357348	A6DBJ4	391592
A3PDH0	167546	A6Q2Y1	387092
A3PJH5	349101	A6UTS7	419665
A3Q7A2	164757	A7IA28	456442
A3QIP0	323850	A8A985	453591
A4CLQ7	313596	A8UYD4	392423
A4F6E9	405948	B0A9M6	445973
A4FLG7	405948	B0MLF0	428128
A4FMP9	405948	B1H0A5	471821
A4G3X8	204773	B1L5I8	374847
A4G4I1	204773	B1L9K0	126740
A4IRH8	420246	B1YDA5	444157
A4J025	418136	B2KBD8	445932
A4J181	349161	B4U7M0	380749
A4J185	349161	B5IBC3	439481
A4JGE0	269482	B5ICW9	439481
A4JI05	269482	B5YA68	309799
A4QAP0	340322	B5YEF6	309799
A4QR67	148305	B5YHE5	289376
A4RRV6	436017	B7RDC9	443254
A4SCL9	290318	B8E2R0	515635
A4SFC7	290318	B8E2X1	515635
A4SFD0	290318	B8I4G1	394503
A4SLG1	382245	B9KC21	309803
A4SR62	382245	B9L5K2	598659
A4SXR0	312153	B9MLV3	521460
A4T617	350054	C3MKR6	429572
A4TQA2	386656	C3MUB4	427317
A4VNV6	379731	C3N1A2	427318
A4VPH4	379731	C3N992	439386

A4VXS6	391295	C3NMW1	419942
A4W423	391296	C3XFH7	613026
A4W6H9	399742	C4KKU5	426118
A4WDP9	399742	C5CJ09	521045
A4WMI8	340102	C7II83	588581
A4WMJ2	340102	C9RDA0	429009
A4WMM6	340102	D0KN35	555311
A4WSX7	349102	D2BGU7	311424
A4WU99	349102	D2C7S8	590168
A4X1F9	369723	D2PF89	425944
A4X4B3	369723	D2RD58	572546
A4XIL8	351627	D2RE08	572546
A4XIL9	351627	D3DH48	608538
A4XQZ1	399739	D3L1C0	592015
A4XY24	399739	D3MYU5	547146
A4Y2M0	319224	D3S0L5	589924
A4YII3	399549	D3S625	644281
A4YX00	114615	D3SHV0	633145
A4YZ76	114615	D3SMX6	638303
A5CRB9	443906	D3TBK5	439481
A5CWZ3	412965	D4XYJ9	552811
A5D4W4	370438	D5VRI3	573063
A5DK19	4929	D7DR01	456320
A5DVS2	36914	O67399	63363
A5DYI7	36914	Q0W1Q4	351160
A5EHF0	288000	Q0W5L6	351160
A5EP79	288000	Q12TV2	259564
A5FR43	216389	Q12UW0	259564
A5FR51	216389	Q2FSZ6	323259
A5FVX6	349163	Q3Z9A6	243164
A5G3X8	351605	Q3ZZJ9	255470
A5G7V0	351605	Q46FW4	269797
A5GM47	32051	Q703X7	2271
A5GRZ0	316278	Q97EE1	1488
A5IJM2	390874	Q97VY3	2287
A5IJM3	390874	Q9WYC8	2336
A5IRF4	359786		
A5IUK3	359786		
A5MZ76	431943		
A5N495	431943		
A5U936	419947		
A5UD85	374930		
A5UMM3	420247		
A5UUP7	357808		
A5UUP9	357808		
A5V3R3	392499		

A5VPL4	444178
A5VRU2	444178
A5VZB6	351746
A5WE78	349106
A5WTS8	336982
A6L1V7	435590
A6LDN1	435591
A6LPX3	290402
A6LVG2	290402
A6Q4V5	387092
A6QBP1	387093
A6QIQ3	426430
A6S6B4	332648
A6SX91	375286
A6SZY8	375286
A6T4L9	272620
A6TCV1	272620
A6U083	359787
A6U3E2	359787
A6U7E4	366394
A6UBT9	366394
A6UP71	406327
A6V0X2	381754
A6V355	381754
A6VQK8	339671
A6VSA9	400668
A6VWW7	400668
A6WDF2	266940
A6WIB5	402882
A6WZB1	439375
A6X268	439375
A7F9C4	665079
A7FM84	349747
A7GMU1	315749
A7GWY5	360105
A7H667	360109
A7HB60	404589
A7HBI1	404589
A7HP03	402881
A7HS43	402881
A7I0N8	360107
A7I5V5	456442
A7I9C0	456442
A7IEY5	78245
A7IPI8	78245
A7K4A1	150340

A7MIC5	290339
A7MWC1	338187
A7NJH4	383372
A7NJH6	383372
A7TNA0	436907
A7X4N1	418127
A7Z7B8	326423
A7ZES2	360104
A7ZHG6	331111
A7ZW25	331112
A8AA75	453591
A8AB61	453591
A8ABX3	453591
A8ALM5	290338
A8ES07	367737
A8EV45	367737
A8F3U8	416591
A8FAK7	315750
A8FFW5	315750
A8FP35	407148
A8FQ83	425104
A8G5D5	93060
A8G9R1	399741
A8GCY8	399741
A8H9A1	398579
A8I4Y2	438753
A8IEW7	438753
A8L551	298653
A8L558	298653
A8LLR0	398580
A8LPL9	398580
A8LYI4	391037
A8M5G7	391037
A8MB07	397948
A8MCN2	397948
A8MDZ2	397948
A8NEF9	240176
A8PWG4	425265
A8Z4W0	451516
A8Z5S1	444179
A8ZTR1	96561
A8ZTR2	96561
A9A1L0	436308
A9A4I8	436308
A9AEW4	395019
A9AJN4	395019

A9AZ36	316274
A9AZM4	316274
A9BB43	93059
A9BGP1	403833
A9BME6	398578
A9BME7	398578
A9BNM7	398578
A9FJU5	448385
A9FMN1	448385
A9GW82	448385
A9GW85	448385
A9HMA2	272568
A9HW07	340100
A9KIN4	357809
A9KRN2	357809
A9KY13	399599
A9LYZ1	374833
A9M6X7	483179
A9MA52	483179
A9MZK0	272994
A9R142	349746
A9S4K9	145481
A9T9B8	145481
A9VLG7	315730
A9WC27	324602
A9WML8	288705
B0BTP8	434271
B0C3D3	329726
B0CEB7	329726
B0CI01	470137
B0CL04	470137
B0JFN2	449447
B0JGK2	449447
B0K6M2	399726
B0K7V2	340099
B0KRD9	76869
B0RCQ8	31964
B0RP28	509169
B0S9H2	355278
B0SF06	355278
B0SN40	456481
B0SS61	456481
B0T0G9	366602
B0TCR1	498761
B0TCR5	498761
B0TQM0	458817

B0TW82	484022
B0U2S5	405440
B0UKW3	426117
B0USF6	228400
B0V4R9	509173
B0VL50	509170
B1GYX1	471821
B1H0A7	471821
B1HR98	444177
B1I1Y1	477974
B1I1Y4	477974
B1IC50	487214
B1IRA4	481805
B1JDI2	390235
B1JK98	502800
B1JVQ1	406425
B1JYQ9	406425
B1K7V8	406425
B1K8B2	406425
B1KKZ4	392500
B1L3C4	374847
B1L8U2	126740
B1L8U3	126740
B1LG11	439855
B1M239	426355
B1MF23	561007
B1MFN6	561007
B1MVR2	349519
B1VIR4	504474
B1VY56	455632
B1VZ53	455632
B1WQQ4	43989
B1X098	43989
B1XHY4	32049
B1XLQ9	32049
B1XUJ3	452638
B1XYA8	395495
B1XYA9	395495
B1YBE0	444157
B1YDA0	444157
B1YDA6	444157
B1YN62	398577
B1YTR7	398577
B1ZDN3	441620
B1ZZJ0	452637
B2A0B6	452637

B2AHM5	164546
B2AUM7	5145
B2FT78	522373
B2GHT7	378753
B2HKD3	216594
B2I3B7	405416
B2I596	405441
B2IED9	395963
B2IFN7	395963
B2IU89	63737
B2J7J8	63737
B2JCD3	391038
B2JDN6	391038
B2K4C9	502801
B2S4T8	430066
B2S726	430066
B2SNG9	360094
B2SXZ9	398527
B2T2D7	398527
B2TJA4	508765
B2U281	344609
B2U7S0	402626
B2UJY9	402626
B2ULC1	349741
B2V4X2	508767
B2V7D9	436114
B2V9M9	436114
B2VDA7	338565
B3DUA5	205913
B3DX88	481448
B3DYI6	481448
B3E3D5	398767
B3EBJ2	398767
B3EHI0	290315
B3EHN6	290315
B3EKI8	331678
B3EKV3	331678
B3EKV6	331678
B3H0Q7	537457
B3PEP5	498211
B3PUR8	491916
B3PV47	491916
B3QB91	395960
B3QCS7	395960
B3QCX5	395960
B3QM95	517417

B3QRE0	517417
B3QSN5	517418
B3QSN8	517418
B3R3V8	164546
B4E5N2	216591
B4EAQ3	216591
B4EHL9	216591
B4F194	529507
B4RFC9	450851
B4RLF0	521006
B4RVU7	314275
B4S3P6	290512
B4S6M8	290512
B4S6N1	290512
B4SBC0	324925
B4SDK9	324925
B4SDL2	324925
B4SI67	391008
B4SU36	423368
B4TJ72	454169
B4TWW1	439843
B4U9Q7	380749
B4U9S8	380749
B4UAM9	447217
B4UL75	447217
B4UML4	447217
B5BLB5	554290
B5E4Z4	512566
B5EHU7	404380
B5EI20	404380
B5EJU9	380394
B5EMT9	380394
B5F7U9	454166
B5FGH4	388396
B5FI58	439851
B5R2K9	550537
B5RGE4	550538
B5XVC6	507522
B5Y1W2	507522
B5YEF8	309799
B5YJS7	289376
B5YK61	289376
B5YZB0	444450
B5ZWA7	395492
B5ZY91	395492
B6ELK0	316275

B6HIA0	500485
B6HZ24	409438
B6ISG5	414684
B6ITR2	414684
B6JB87	504832
B6JHT5	504832
B6SH97	4577
B6SWM0	4577
B6YRN0	511995
B7FUP6	556484
B7G5N8	556484
B7GH19	491915
B7GT76	391904
B7H097	557600
B7HHF5	405532
B7HKC4	405534
B7I4X5	480119
B7IN93	405531
B7J7R3	243159
B7JC84	243159
B7JFY5	405535
B7JUG5	41431
B7JYP4	41431
B7KF92	65393
B7KJX8	65393
B7KQH6	440085
B7LFU5	585055
B7LWE2	585054
B7M119	585034
B7MAJ9	585035
B7MNT2	585397
B7N7U9	585056
B7NHI2	585057
B7UIC4	574521
B7UWV9	557722
B7UXH6	557722
B7VIF2	575788
B8A2E9	4577
B8BRI7	296543
B8C732	35128
B8CM39	225849
B8CX25	373903
B8DBU4	552536
B8DM91	883
B8DPE5	883
B8DVK8	442563

B8E2X3	515635
B8E4K4	407976
B8EMY4	395965
B8EP66	395965
B8F7W2	557723
B8FHI2	439235
B8FHI3	439235
B8FYI5	272564
B8G7X2	326427
B8GDQ8	521011
B8GDT9	521011
B8GUB3	396588
B8H607	565050
B8H8Q9	452863
B8HST9	395961
B8I1T6	394503
B8I1T7	394503
B8IHS8	460265
B8J4F8	525146
B8J737	455488
B8J824	455488
B8JGT3	455488
B8MZH6	332952
B8ZT16	561304
B9DMJ6	396513
B9DXW3	583346
B9E371	583346
B9IUZ0	361100
B9J7D4	311403
B9JCA0	311403
B9JHD2	311403
B9JUR0	311402
B9JZ80	311402
B9KB95	309803
B9KB96	309803
B9KPM0	557760
B9KRV9	557760
B9KY22	309801
B9L0Y0	309801
B9L7G8	598659
B9LGM8	480224
B9LUZ7	416348
B9M2T5	316067
B9M5A8	316067
B9MA32	535289
B9MA33	535289

B9MNV2	521460
B9MNV4	521460
B9SX30	3988
B9W6H7	573826
B9WCP0	573826
C0Q5H1	476213
C0QDD0	177437
C0QDD1	177437
C0QM22	177437
C0QPV4	123214
C0QQB9	123214
C0R089	565034
C0REK0	546272
C0RI20	546272
C0Z635	358681
C0Z7H0	358681
C0Z9C8	358681
C0ZI85	358681
C0ZN67	234621
C0ZRN0	234621
C1AIG4	561275
C1AUX2	632772
C1B9P5	632772
C1C7N7	488221
C1CWU2	546414
C1DA11	557598
C1DE40	322710
C1DFH2	322710
C1DUG8	204536
C1DWG8	204536
C1EMA9	572264
C1F702	240015
C3K1K7	216595
C3L9Q7	568206
C3LR32	579112
C3M992	394
C3MFP8	394
C3MJ64	429572
C3MYV6	427317
C3N004	427318
C3N887	439386
C3NF24	419942
C3NVB2	593588
C3P4Z6	592021
C3PJR0	548476
C4JY82	336963

C4KAF1	85643
C4KJ45	426118
C4KPJ3	536230
C4KU56	536230
C4LAV7	595494
C4LGL7	645127
C4R0L2	644223
C4X378	484021
C4XCF1	484021
C4XPA8	573370
C4XQB4	573370
C4XX33	306902
C4Y5K7	306902
C4Z0W1	515620
C4ZDA3	515619
C4ZPZ7	595496
C5AAL9	626418
C5ADL5	626418
C5AHP0	626418
C5AK37	626418
C5AUL0	272630
C5B7R4	634503
C5BQI9	377629
C5BQS2	377629
C5C2J4	471853
C5C4P2	471853
C5CCC3	465515
C5CYP1	543728
C5CYP2	543728
C5D5M0	471223
C5DGX9	559295
C5DRM6	559307
C5DT42	559307
C5M530	294747
C5MDZ9	294747
C5VVA9	218494
C5Y4G6	4558
C5YQI8	4558
C6A662	580050
C6AGS0	555970
C6APR9	634176
C6AT55	395491
C6ATJ8	395491
C6BJQ8	428406
C6BLN0	428406
C6BSG8	526222

C6BV83	526222
C6C9J2	579405
C6CD53	579405
C6CJV8	561229
C6CWB7	324057
C6CYA6	324057
C6DEV8	561230
C6DJY7	561230
C6DNF0	478434
C6E2T7	443144
C6E526	443144
C6GSE1	568813
C6GXA3	568814
C6S6Y5	662598
C6SPV9	511691
C6UM38	413997
C6UVR1	544404
C6VV52	471854
C6WF52	446462
C6WG56	446462
C6WYB6	583345
C6X713	582744
C6X7K6	582744
C6XN10	582402
C6XSQ5	485917
C7BQ10	553480
C7C7H0	661410
C7JAV8	634452
C7LAY8	568815
C7LDF9	568815
C7LK15	595499
C7LRJ6	525897
C7LW16	525897
C7M0E4	525909
C7M0F3	525909
C7MD85	446465
C7MDN4	446465
C7MNU3	469378
C7MQR7	471857
C7MXE9	471857
C7N750	471855
C7NBT9	523794
C7NTA3	519442
C7NZI9	485914
C7PC99	485918
C7Q5A2	479433

C7QE20	479433
C7QJN7	479433
C7QKV4	395962
C7QUU4	395962
C7QYV9	471856
C7R518	471856
C7RAA1	523791
C7RNH2	522306
C7RP33	522306
C7RRT0	522306
C8TGK5	573235
C8U1E6	585395
C8UGU5	585396
C8VYG5	485916
C8W660	485916
C8W663	485916
C8WB59	622759
C8WI89	479437
C8WKR4	479437
C8WRC1	521098
C8WUC8	521098
C8X2U9	485915
C8X3J3	485915
C8X8C8	479431
C8XEL4	479431
C9RAX2	429009
C9RGX2	579137
C9RKZ6	59374
C9RN88	59374
C9RU47	544556
C9XME2	645462
C9XVN1	413502
C9YJU2	645463
C9Z581	680198
C9ZAQ8	680198
D0FPG3	79967
D0J5D5	688245
D0J5D6	688245
D0J7V6	688245
D0J9Z9	600809
D0JAB2	331104
D0K6U4	681288
D0KMH9	561231
D0L133	555778
D0LDG8	526226
D0LGL9	502025

D0LNR8	502025
D0MG82	518766
D0MG85	518766
D0ZC67	498217
D1A9T8	471852
D1ABQ4	471852
D1AJT5	526218
D1AJT6	526218
D1B3V3	525898
D1B739	525903
D1BDG4	446469
D1BEZ8	446469
D1BQH0	479436
D1BUW1	446471
D1BVS8	446471
D1C1T3	479434
D1CDV6	525904
D1CDV9	525904
D1Z010	304371
D1Z2F8	304371
D2AVP7	479432
D2AZP6	479432
D2BHR1	311424
D2BHR6	311424
D2BQX0	684738
D2BRQ8	590409
D2BWM4	590409
D2C649	590168
D2C650	590168
D2NT82	680646
D2NVH1	653938
D2P6U6	637381
D2PE76	425944
D2PW56	479435
D2Q0U4	479435
D2Q7U5	401473
D2QSS8	504472
D2QX79	530564
D2R560	530564
D2RKU9	591001
D2RXB9	543526
D2S666	526225
D2SEJ5	526225
D2TGD2	637910
D2UCH0	380358
D3DHH1	608538

D3DJ10	608538
D3EA49	481743
D3EBC1	481743
D3EER3	481743
D3F0A4	469383
D3F0B7	469383
D3FWD7	398511
D3H9B8	365659
D3HCJ2	637909
D3NWT9	137722
D3NXD0	137722
D3P8S8	639282
D3PDH5	639282
D3PT79	504728
D3PT80	504728
D3PZI9	446470
D3Q5Q6	446470
D3QIN4	698737
D3QVG0	701177
D3RBN0	640131
D3RJH6	640131
D3RN33	572477
D3RT05	572477
D3S1E8	589924
D3S3U6	644281
D3SAZ5	396595
D3SIR4	633145
D3SIR9	633145
D3SLJ2	638303
D3SLM7	638303
D3SQK2	547559
D3T3R9	580331
D3UIF2	679897
D3UPT7	683837
D3V4N2	406818
D3V8R4	406817
D4GEB2	706191
D4GJR3	706191
D4GYF6	309800
D4H3V8	522772
D4H837	522772
D4HDA0	553199
D4I011	665029
D4IFX8	716540
D4Z111	452662
D4ZDW0	637905

D5AT02	272942
D5AU62	272942
D5B0U3	637386
D5BLR2	655815
D5BRM3	488538
D5BT69	488538
D5BWV8	472759
D5C8G8	716541
D5CH57	716541
D5CNR5	580332
D5CUG7	580332
D5D8H8	641892
D5DLN1	592022
D5DTM4	545693
D5E8E5	547558
D5EBW6	547558
D5ECG7	572547
D5EJA6	583355
D5EJL6	583355
D5EYZ7	264731
D5SN19	521674
D5SYW5	521674
D5T3U8	762051
D5TUA6	714359
D5U4B2	526224
D5UG58	446466
D5ULN2	446466
D5UN64	521096
D5V2E3	572480
D5V4I8	572480
D5VCR0	749219
D5VFU9	509190
D5W5W9	640511
D5W9H9	640511
D5WW56	562970
D5X0W9	75379
D5XAF4	635013
D6W175	559292
D6XXJ0	439292
D6Y692	469371
D6Y8J0	469371
D6Z0M0	589865
D6Z1T6	589865
D6ZCA8	640132
D6ZGP8	548479
D6ZGQ2	548479

D6ZS81	525381
D6ZXJ9	759350
D7A2B8	639283
D7AQ76	583358
D7AU69	446468
D7AYI6	446468
D7BHS1	526227
D7BHS2	526227
D7CJ19	643648
D7CJ23	643648
D7CXY7	649638
D7CXY8	649638
D7D291	691437
D7DM18	666681
D7DWL3	551115
D7DY92	551115
D7E0I5	551115
D7E5V1	644295
D7E808	644295
D7GD07	754252
D7GE89	754252
O30020	2234
O31046	1902
O59390	53953
O66682	63363
O67862	63363
O86511	1902
P42455	1718
P43861	727
P48575	103690
P48576	1148
P58899	196620
P63476	158878
P63477	158879
P74269	1148
P94565	1423
P96420	1773
Q01UL9	234267
Q02J53	208963
Q02RU6	208963
Q02YX5	272622
Q03KB0	322159
Q03UM0	203120
Q04DA4	203123
Q04QM8	355277
Q04T27	355277

Q051E9	355276
Q054A1	355276
Q05FR2	387662
Q07PI3	316055
Q07Q38	316055
Q07WG9	318167
Q0AB82	187272
Q0AGN5	335283
Q0AV20	335541
Q0AV24	335541
Q0BBP5	339670
Q0BDB9	339670
Q0BS32	391165
Q0C523	228405
Q0HZT4	60481
Q0I2G1	205914
Q0IBI3	64471
Q0K537	381666
Q0KCT8	381666
Q0RDJ1	326424
Q0RDJ9	326424
Q0S690	101510
Q0S8X0	101510
Q0T8C4	373384
Q0TLR5	362663
Q0V1B4	13684
Q0VLR3	393595
Q0VRW2	393595
Q0W1Q3	351160
Q0W3Q1	351160
Q0W6C5	351160
Q10Y65	203124
Q112U2	203124
Q119Q8	203124
Q11G93	266779
Q11JQ9	266779
Q11NN9	269798
Q12B47	296591
Q12B48	296591
Q12SE7	318161
Q12V56	259564
Q12XM1	259564
Q138Z2	316057
Q139D5	316057
Q13WM0	266265
Q142I2	266265

Q14JJ0	393115
Q15QR4	342610
Q15VT6	342610
Q164A5	375451
Q16AY3	375451
Q18AI7	272563
Q1ARE0	266117
Q1ARE2	266117
Q1B282	164756
Q1BKJ9	331271
Q1BTF6	331271
Q1BV03	331271
Q1C1Z6	360102
Q1CMP5	377628
Q1DCT2	246197
Q1GEA2	292414
Q1GHH9	292414
Q1GUI1	117207
Q1H3C1	265072
Q1H400	265072
Q1I5K2	384676
Q1J0T3	319795
Q1LAT0	266264
Q1LPX2	266264
Q1MDH6	216596
Q1MJ27	216596
Q1QBL0	335284
Q1QJV3	323097
Q1QN85	323097
Q1QUH6	290398
Q1QUJ6	290398
Q1RGC3	364106
Q212A9	316056
Q216P3	316056
Q21HP6	203122
Q21IC1	203122
Q21VZ1	338969
Q24PE3	138119
Q28L52	290400
Q2A5S3	376619
Q2A5S4	376619
Q2FWK3	93061
Q2FZS6	93061
Q2G619	279238
Q2IDZ9	290397
Q2IJC1	290397

Q2IJL5	290397
Q2IUP3	316058
Q2IUT4	316058
Q2J6V5	106370
Q2J6W2	106370
Q2JLJ8	321332
Q2JLX0	321332
Q2JSL4	321327
Q2JWS4	321327
Q2K5Q2	347834
Q2KAB4	347834
Q2KTP9	360910
Q2L077	360910
Q2LU50	56780
Q2LWJ3	56780
Q2NAV6	314225
Q2NFC1	339860
Q2NHL7	339860
Q2NVW3	343509
Q2P761	342109
Q2QXY9	39947
Q2RGA1	264732
Q2RT17	269796
Q2RUD3	269796
Q2RWJ6	269796
Q2S0M2	309807
Q2S0M7	309807
Q2S9W6	349521
Q2SBZ8	349521
Q2SZP5	271848
Q2T1D2	271848
Q2UQI7	5062
Q2W1F9	342108
Q2W1K3	342108
Q2W5E1	342108
Q2YBW0	323848
Q2YN18	359391
Q2YRT1	359391
Q2YUF2	273036
Q30PB7	326298
Q30WD4	207559
Q30ZQ0	207559
Q31AF9	74546
Q31EF5	317025
Q31I16	317025
Q31NC9	1140

Q326G1	300268
Q32K20	300267
Q390S3	269483
Q39CT0	269483
Q39ED5	269483
Q39UG6	269799
Q39W73	269799
Q3A3A9	338963
Q3A5U9	338963
Q3AEQ1	246194
Q3AEQ5	246194
Q3AIA2	110662
Q3APC7	340177
Q3AQ00	340177
Q3AYY2	316279
Q3B596	319225
Q3B599	319225
Q3B6K3	319225
Q3BPJ9	316273
Q3IJS2	326442
Q3IQK7	348780
Q3IV11	272943
Q3J2V6	272943
Q3J3Z9	272943
Q3J877	323261
Q3JUB9	320372
Q3JWA5	320372
Q3K7C3	205922
Q3M862	240292
Q3MBA3	240292
Q3SHE7	292415
Q3SQ49	323098
Q3ST98	323098
Q3Z5T6	300269
Q3Z892	243164
Q3Z897	243164
Q3ZXI3	255470
Q3ZXI8	255470
Q46K01	59920
Q470Y1	264198
Q473V1	264198
Q47BI0	159087
Q47IR3	159087
Q47RM9	269800
Q47SB2	269800
Q47WG4	167879

Q48LY5	264730
Q493R0	291272
Q49Z12	342451
Q4FKW1	198252
Q4FLI8	198252
Q4FSC9	259536
Q4J6H1	2285
Q4JSM3	306537
Q4K6V7	220664
Q4K963	220664
Q4L7U0	279808
Q4P6V7	5270
Q4QLS4	281310
Q4UYG1	314565
Q4WRV1	5085
Q4ZX14	205918
Q57BV2	235
Q57E61	235
Q57TE6	28901
Q58595	2190
Q5BF40	162425
Q5E856	312309
Q5F8D4	242231
Q5FQ74	442
Q5HEE4	93062
Q5HMF9	176279
Q5HS76	195099
Q5JFV5	311400
Q5KCL7	5207
Q5KWJ3	1462
Q5LAB0	272559
Q5LRJ7	89184
Q5LWC0	89184
Q5LZF2	299768
Q5M404	264199
Q5N341	269084
Q5N5T3	269084
Q5NI38	119856
Q5NP33	542
Q5NXN2	76114
Q5SJ04	300852
Q5SJ06	300852
Q5V523	2238
Q5WEN3	66692
Q5WQ01	372461
Q5YVA5	37329

Q5Z341	37329
Q605K7	414
Q62FA8	13373
Q63DX8	288681
Q63VP3	28450
Q63XN2	28450
Q64QP0	817
Q65GI8	279010
Q65V04	221988
Q66EM1	633
Q67KX4	2734
Q67MX0	2734
Q6A972	1747
Q6AED3	59736
Q6AJI3	84980
Q6AQI4	84980
Q6CFF6	4952
Q6CQU3	28985
Q6D0G8	29471
Q6FEQ2	62977
Q6FSZ1	5478
Q6G7Q1	282459
Q6GF16	282458
Q6KZ01	263820
Q6L0K4	263820
Q6LV24	74109
Q6N0U6	1076
Q6N858	1076
Q6N8A4	1076
Q6NJX5	1717
Q71Y35	265669
Q726X5	882
Q72AS7	882
Q72JC9	262724
Q72JD1	262724
Q73BA0	222523
Q744M9	1770
Q74C76	35554
Q755P2	33169
Q75AD2	33169
Q7M9W4	844
Q7MP77	196600
Q7N129	141679
Q7NI93	33072
Q7NNJ0	33072
Q7P0H2	536

Q7SFM2	5141
Q7TUV5	74547
Q7TVV6	1765
Q7U892	84588
Q7UI51	265606
Q7UXI0	265606
Q7V121	59919
Q7VBG1	1219
Q7VH30	32025
Q7VQJ6	203907
Q7W071	520
Q7W0I9	520
Q7W2R5	519
Q7WCA0	519
Q7WDR3	518
Q7WQA4	518
Q81G12	226900
Q81T68	1392
Q820M0	915
Q82BV3	33903
Q82JP4	33903
Q83SP0	623
Q87CL8	183190
Q87SS7	670
Q88P28	160488
Q89A49	224915
Q89GB0	375
Q89NP6	375
Q8A6L6	818
Q8CNL3	176280
Q8DEE1	672
Q8DJ32	197221
Q8DKK9	197221
Q8DPJ2	171101
Q8DTG2	1309
Q8E9N2	70863
Q8EN67	182710
Q8F445	173
Q8F8T4	173
Q8FL75	217992
Q8FU05	152794
Q8FZC4	29461
Q8G1N3	29461
Q8G6Z2	216816
Q8GA98	1140
Q8KAP9	1097

Q8KES0	1097
Q8TW28	2320
Q8TYB1	2320
Q8U0C1	2261
Q8U298	2261
Q8U2A2	2261
Q8UD63	176299
Q8XSZ5	305
Q8XXP1	305
Q8Y5R9	1639
Q8YG91	29459
Q8YIJ3	29459
Q8YRC6	103690
Q8ZIG8	632
Q8ZW35	13773
Q8ZW39	13773
Q8ZXD7	13773
Q92A28	1642
Q92R15	382
Q96YG8	111955
Q971S5	111955
Q974X3	111955
Q97A78	273116
Q97ED9	1488
Q97MC5	1488
Q97QF7	1313
Q980Z1	2287
Q984X3	381
Q98HN3	381
Q9A823	155892
Q9C550	3702
Q9CB76	1769
Q9CJN5	747
Q9FG67	3702
Q9FN52	3702
Q9HXK5	287
Q9I4C1	287
Q9K8E8	86665
Q9KP83	666
Q9PCG3	2371
Q9PLV9	197
Q9RUA9	1299
Q9UZ04	29292
Q9UZ08	29292
Q9V1J1	29292
Q9WZ22	2336

Q9WZ23	2336
Q9X7L2	382
A0B606	349307
A0LKA4	335543
A0NKT1	379360
A0NMQ8	384765
A0NVJ4	384765
A0RV79	46770
A0RWW4	46770
A0Y448	156578
A0YAU0	247633
A0YCU0	247633
A0YYJ6	313612
A0Z321	247639
A0ZGD0	313624
A0ZJV5	313624
A1ARF0	338966
A1ER46	345076
A1F8B2	412614
A1HLS3	401526
A2PBC5	412966
A2PVK0	412883
A2TTX6	313590
A2TXI5	313598
A2UXP4	399804
A2VVB3	350702
A2VWP4	350702
A2W1G1	350702
A2W1I4	350702
A2W7B3	350701
A2W8P5	350701
A3ELP4	345075
A3EQC8	419542
A3EV91	419542
A3GGR1	4924
A3GR24	417399
A3H155	417400
A3I6Q3	388400
A3IEM3	388400
A3IRU3	391612
A3IY86	391612
A3JAB7	270374
A3JMT2	388401
A3JTY8	388401
A3JYN8	388399
A3K8V5	388399

A3KRG0	350704
A3KY57	350704
A3L9L4	350703
A3LEP3	350703
A3LU20	4924
A3PIF0	349101
A3RQN7	342110
A3RYN0	342110
A3SB79	52598
A3SGB0	52598
A3SI82	89187
A3SJK8	89187
A3SVG7	314267
A3SWK6	314267
A3TM83	313589
A3TR71	313589
A3TTT5	252305
A3U3L9	252305
A3UY16	314291
A3V1U0	314232
A3V4F0	314232
A3V9K5	314271
A3VJU0	314271
A3VR94	314260
A3VXD2	314264
A3VYC0	314264
A3WCN4	237727
A3WU66	314253
A3WWD4	314253
A3XAZ2	314262
A3XD03	314262
A3XM62	398720
A3Y2P4	314290
A3YF22	314277
A3YF59	314277
A3YN73	360111
A3YTB2	360108
A3YY55	69042
A3Z7T3	221360
A3ZFU8	360112
A3ZKI5	360110
A3ZTL4	314230
A4A900	314285
A4AGE3	312284
A4AUW5	313603
A4BJ60	314283

A4BLL9	314278
A4BVJ0	314278
A4BY79	313594
A4CER5	87626
A4CVP9	59931
A4EEV9	391593
A4EKK2	391593
A4EQL7	388739
A4ERG8	388739
A4GJN5	415444
A4KPE7	412422
A4LJH5	425067
A4LUK2	425067
A4MW40	374927
A4MW41	374927
A4N1S5	375432
A4N870	375177
A4NDG2	374928
A4NLZ9	374932
A4NQ55	374933
A4NW92	375063
A4RD53	148305
A4TZ97	55518
A4U0T7	55518
A4U2F2	55518
A5DCX1	4929
A5DEU6	4929
A5F5F1	345073
A5FFY8	376686
A5J666	334802
A5KHS7	398000
A5KLA4	411460
A5L188	391574
A5LDK0	406556
A5LMW4	406557
A5LXG9	406558
A5M3I2	406559
A5M7L2	406560
A5MGP7	406561
A5MP83	406562
A5MP84	406562
A5MSR5	406563
A5P8D1	161528
A5TIF1	370895
A5TSK7	393480
A5UL49	420247

A5XS50	334803
A5Z8D6	411463
A5ZLQ7	411901
A5ZYM6	411459
A6A6F8	417398
A6AGG0	417397
A6AS64	410291
A6B5R4	419109
A6BIJ6	411462
A6BUX3	412420
A6CAS9	344747
A6CEN2	344747
A6D7A5	391591
A6DDK1	391592
A6DPR2	313628
A6DY52	391613
A6E667	391613
A6EQE9	50743
A6F2W3	443152
A6F536	443152
A6FCC4	58051
A6FIS1	58051
A6FRB8	351016
A6FU82	351016
A6GEH6	391625
A6GIC5	391625
A6GJ21	391625
A6GM55	391597
A6GS69	391597
A6NZF3	411467
A6QFI7	426430
A6QYH1	339724
A6SEV6	332648
A6URD4	406327
A6Y082	404974
A6Y6N0	345074
A6ZNV6	307796
A6ZRX6	307796
A6ZXE9	307796
A7A3F7	411481
A7AK39	411477
A7AYH5	411470
A7BE59	411466
A7BY33	422289
A7F9M5	665079
A7HCZ9	404589

A7JDJ9	430557
A7JFD9	430558
A7JPC3	442346
A7JU64	272629
A7M3B6	411476
A7V0M4	411479
A7VC76	411489
A7VNV2	428125
A7VT64	428125
A7X0J5	418127
A7YR14	430556
A8E983	360118
A8EG84	360118
A8KJ50	331978
A8KTW7	331978
A8NU10	240176
A8QCA5	425265
A8RY41	411902
A8S7H2	411485
A8SX95	411474
A8T8G6	314289
A8TMZ9	331869
A8TTA3	331869
A8UJQ1	391603
A8US86	392423
A8USM2	392423
A8YAL3	267872
A8YBI6	267872
A8Z082	451516
A9BGP5	403833
A9D229	411684
A9DB92	411684
A9DLH7	391587
A9DV85	391624
A9E5V6	391624
A9EKZ7	314608
A9F8P0	383629
A9FHR3	383629
A9FPS3	391619
A9FTR1	391619
A9HS14	391595
A9HVF8	391595
A9JZR6	412021
A9W659	419610
A9Z8J8	373665
B0A519	404214

B0A9M8	445973
B0AQJ5	486624
B0D9Z2	486041
B0G5U5	411461
B0GJ33	404218
B0GX24	404217
B0H9V1	404216
B0HGZ1	404215
B0HRQ3	360099
B0MIX5	411490
B0MPI7	428128
B0N940	445974
B0ND18	411468
B0NQ62	449673
B0NXP5	411484
B0PBC1	445972
B0PBH3	445972
B0Q2H2	486619
B0QJ14	486621
B0QTI6	456298
B0XN47	451804
B1C4Q1	428126
B1C9W0	445971
B1EMJ2	502347
B1EXN2	486623
B1FB32	396596
B1FJC7	396596
B1FYA3	396598
B1G9D2	396598
B1GHA0	486620
B1HBR0	320374
B1HG31	320374
B1HZL8	444177
B1HZL9	444177
B1QZX4	447214
B1R1D5	447214
B1S7G1	473819
B1SFI4	471872
B1SZA1	396597
B1T9X4	396597
B1UPR6	486622
B1X5H2	39717
B2D157	3712
B2DNJ4	453363
B2EB00	486409
B2H1I3	331109

B2H2D4	331109
B2IPM2	516950
B2J7L4	63737
B2ND11	344610
B2NWU1	444451
B2PAZ8	444452
B2PN01	444453
B2Q2B0	471874
B2SXF3	398527
B2UP78	349741
B2WKN1	426418
B3A7L3	478004
B3AUP6	478005
B3B7S0	478006
B3BN80	478008
B3C1M9	478007
B3CIP9	471870
B3HFJ5	340184
B3HY42	340197
B3IBE3	340185
B3IQ54	340186
B3J961	405536
B3JN27	470145
B3LH66	285006
B3LJF1	285006
B3LNW3	285006
B3T3A6	455568
B3T4B1	455574
B3T5X7	455578
B3TAF1	455603
B3TCF0	455612
B3TCK7	455613
B3V5P6	526639
B3WR77	344601
B3X4W1	358708
B3XIL4	358709
B3YL65	439842
B3YX14	405917
B3ZGF8	451707
B3ZVW1	451709
B4A3M5	454168
B4AM30	536229
B4ARV6	545422
B4AWS2	497965
B4B3Y2	497965
B4B7I5	497965

B4BQE3	495036
B4D232	497964
B4DBF9	497964
B4V4A9	465541
B4V574	465541
B4VQW7	118168
B4VRZ4	118168
B4WG76	91464
B4WMH5	91464
B4WQ98	91464
B4WXC9	236097
B4X4L7	236097
B5C202	439846
B5C8P5	454165
B5CS19	471875
B5CXZ4	484018
B5GJ39	465543
B5H0F3	443255
B5H3M9	443255
B5H722	457429
B5HJW6	457429
B5HM74	463191
B5ING5	180281
B5J4W8	391626
B5J9E5	391626
B5JEF9	382464
B5JEX9	382464
B5JSG5	391615
B5K8F6	391616
B5K8N4	391616
B5KJ60	51350
B5KJ62	51351
B5LAV3	4072
B5ML48	439847
B5MV75	440534
B5N7Y2	454167
B5NMC6	454231
B5P5S8	454164
B5PHV2	465518
B5PV90	465516
B5Q229	465517
B5QFV5	478547
B5S2B7	564065
B5S9M5	305
B5SA89	305
B5SIJ0	564066

B5UIC4	405533
B5VAX9	451708
B5VF91	545124
B5VRZ8	545124
B5W5P9	513049
B5W9X5	513049
B5W9X6	513049
B5WFC3	516466
B5WKI7	516466
B5WNP7	516466
B6AN22	419541
B6ANX8	419541
B6AXP6	314270
B6AYN7	314270
B6B711	439496
B6BE88	439496
B6BHX8	439483
B6BQR8	439493
B6BT71	439493
B6BWJ2	314607
B6C685	473788
B6FS23	500632
B6K1B6	402676
B6QAU4	441960
B6QAU5	441960
B6R018	439495
B6R7V7	439495
B6SWN1	4577
B6W1C7	483217
B6WXT0	411464
B6XKC9	520999
B6XXL2	566552
B6ZSE5	502346
B7A8W7	498848
B7A9U4	498848
B7A9U6	498848
B7AHE0	483216
B7AQK9	483218
B7CHH2	557724
B7CJI2	557724
B7DMS5	543302
B7DVI5	543302
B7FFB9	3880
B7FLQ1	3880
B7KIJ7	65393
B7QRX7	439497
B7QYF0	439497
B7R898	391606
B7RB76	443254
B7RB77	443254
B7RGZ1	391589
B7RQ06	391589
B7S2H6	247634
B7WWN3	399795

B7X0V2	399795
B7X0V3	399795
B8BLZ1	39946
B8KCX2	391586
B8KLW0	566466
B8KUK0	565045
B8L930	391601
B8M2U2	441959
B8PD45	561896
B9AFN1	483214
B9AG63	483214
B9B3J6	513051
B9B536	513051
B9BQ31	513052
B9BU44	513052
B9CBJ7	513053
B9CBW1	513053
B9CVH3	553212
B9D596	553218
B9NRA6	467661
B9NRD8	467661
B9P2F2	93058
B9QW62	244592
B9R0E4	244592
B9WX02	286604
B9XB18	320771
B9XNF8	320771
B9YY96	279714
C0A256	278957
C0AEA7	278957
C0AWH7	471881
C0B728	470146
C0BKB1	487796
C0BR44	547043
C0BWJ9	553973
C0CLH8	476272
C0CU78	518636
C0DRR9	546274
C0DZP7	566549
C0EF38	537013
C0ENH9	546264
C0EU60	411469
C0FPY7	622312
C0G560	595497
C0G7I7	595497
C0GDS3	555088
C0GHP2	555088
C0N6S5	637616
C0N9N4	637616
C0NF36	447093
C0P8I4	4577
C0SDV3	482561
C0VNQ1	525244
C0VRK7	548477

C0W316	525246
C0WAT1	563191
C0WHW2	525260
C0WPK2	525318
C0XN12	525327
C0XU51	525263
C0Y2R1	595498
C0YEE7	595498
C0YKN4	525257
C1CVX4	546414
C1FID7	296587
C1G3U9	502780
C1GHU8	502780
C1H3Y8	502779
C1HPN3	469598
C1HXJ6	528345
C1I926	457396
C1IA85	457396
C1MCI7	469595
C1N4J5	564608
C1NC19	457400
C1PE06	345219
C1V8I3	469382
C2BKT1	525264
C2BZ04	525367
C2C529	592313
C2CS87	525268
C2D0W1	525310
C2DN10	525281
C2FX06	525372
C2FX07	525372
C2G5S5	548473
C2GJE6	548478
C2GSR6	548480
C2HWQ0	593585
C2I5B5	593586
C2IJN0	593589
C2IS56	593590
C2JFB9	593587
C2KM53	586220
C2LIM8	525369
C2LR08	596322
C2LWA8	629742
C2MI66	526973
C2MY93	526980
C2NF10	526970
C2NWA2	526967
C2PCC0	526971
C2PTD5	526972
C2Q9D7	526968
C2QQE6	526977
C2R5H9	526969
C2RKH6	526974
C2S169	526975

C2SHQ0	526976
C2SYD1	526978
C2TDY2	526979
C2TUP8	526981
C2UBA1	526982
C2USS2	526983
C2V970	526984
C2VR31	526985
C2W5R9	526986
C2WJV1	526987
C2WM62	526987
C2WZ79	526988
C2X981	526989
C2XRD6	526990
C2Y7Z3	526991
C2YP22	526992
C2Z5A5	526993
C2ZLX1	526994
C3A3F6	526997
C3AJE5	526998
C3APH1	526998
C3B0T0	526999
C3B6W5	526999
C3BHX6	527000
C3BNV9	527000
C3BZQ7	527024
C3CG67	527021
C3CZ23	527025
C3DH90	527026
C3E0Y9	527027
C3EI82	527023
C3EZ10	527022
C3FHK3	527031
C3G0C8	527032
C3GG76	527029
C3GYF3	527030
C3HFW0	527028
C3HXT8	527019
C3IGW0	527020
C3IY03	550542
C3JNY6	596309
C3JVJ3	596309
C3Q6F9	457395
C3QJC9	556258
C3QP42	469590
C3RF70	556260
C3RJS4	556270
C3TQV7	562
C3X6V7	556268
C3X7A0	556269
C3XFW7	613026
C3XKT5	556267
C4APL4	320390
C4ATU4	320390

C4EY09	521005
C4F2E8	521004
C4FCH7	518635
C4FJA6	432331
C4FK94	432331
C4FRW0	546273
C4G7I2	592010
C4GA07	626523
C4GLX0	629741
C4H2I2	547047
C4HJG8	547046
C4IEY6	632245
C4IG98	632245
C4IPX7	641140
C4IR72	641140
C4J3S6	4577
C4RBA6	219305
C4REA0	219305
C4S227	349968
C4SDX3	349967
C4SIZ4	349966
C4SW76	349965
C4TRZ3	527012
C4U060	527012
C4UCL7	527002
C4ULQ6	527005
C4UY78	527004
C4V3I3	638302
C4WCT2	596319
C4WE71	641118
C4WFI6	641118
C4WMA8	641118
C4YFW0	5476
C4YKP2	5476
C4ZL29	85643
C5DII8	559295
C5DP09	559307
C5E9D9	537937
C5EF15	457421
C5EZI5	537972
C5FUW4	554155
C5GKV1	559297
C5H9M9	51351
C5J4N7	3707
C5J4N9	3707
C5J4P1	3707
C5K0P5	559298
C5MZ49	450394
C5NC34	436115
C5NDS4	436115
C5PAF9	222929
C5PJU2	525373
C5Q4E2	548474
C5Q688	525374

C5QHT0	548475
C5QKQ0	548475
C5QLP3	525378
C5QUK8	525376
C5RIH1	573061
C5RMR5	573061
C5RRZ9	573062
C5S0P4	637911
C5SI86	573065
C5SK24	573065
C5T5A8	573060
C5TPH5	596320
C5TV94	573059
C5U2E7	573059
C5U936	509193
C5UX23	536233
C5V3M8	395494
C5V678	395494
C5VA14	553207
C5W368	511693
C5ZCI0	357347
C5ZJ88	357347
C5ZVF3	537970
C6EAY0	469008
C6HP01	544712
C6HYG3	412449
C6HZV3	412449
C6I7I8	457392
C6IRQ7	469586
C6IVA3	621372
C6J4S5	621372
C6J6H4	621372
C6J9T8	457412
C6KVT5	77133
C6LJ70	478749
C6M9J2	547045
C6MF56	153948
C6ML81	443143
C6MN13	443143
C6NS60	637389
C6NS61	637389
C6P8Q9	580327
C6PQI3	536227
C6QGH1	582899
C6QIH1	582899
C6QKF2	581103
C6R4L8	553201
C6R7K4	553206
C6RIJ5	553219
C6RQZ6	596318
C6RT17	596318
C6S2B7	661513
C6SEE8	663926
C6SM79	295996

C6TSP4	320371
C6TX44	320371
C6YGE6	345072
C6YN52	543737
C6YTJ0	539329
C6Z5G7	457394
C7DAW2	633131
C7DES7	633131
C7GFU8	536231
C7GMM8	574961
C7H4B8	411483
C7HGQ0	572545
C7HGY2	572545
C7HLI8	588857
C7ILY9	588581
C7ILZ0	588581
C7INP1	588581
C7IU29	589861
C7JJK8	634453
C7JTS6	634454
C7K418	634455
C7KD87	634456
C7KMK3	634457
C7KWW8	634458
C7L6F5	634459
C7XCB2	563193
C7YJ23	660122
C7YV47	660122
C7ZVJ1	585143
C7ZYU5	585143
C8A327	585145
C8A6D4	585145
C8AAJ6	585146
C8ADS3	585146
C8AI70	585153
C8ALG0	585153
C8AQL2	585158
C8ATW3	585158
C8JRQ5	393124
C8K2H1	393125
C8KBS3	393128
C8KNL2	452948
C8KTD4	455227
C8KX84	591023
C8L3G4	553565
C8L6G1	553565
C8LCD6	553567
C8LD70	553567
C8LFX4	553568
C8LPQ1	553571
C8LUT2	553571
C8LWV7	553573
C8M2A4	553573
C8M5C6	553581

C8M6D8	553581
C8MBT6	553583
C8MIP8	553588
C8MPJ2	553588
C8MRC1	553592
C8MWR7	553592
C8MYW5	553596
C8N4R0	553596
C8N7F2	638300
C8NR86	196164
C8NXJ3	585529
C8PLJ2	553220
C8PZZ0	553217
C8Q572	592316
C8QAB0	592316
C8RV49	525262
C8RZN6	371731
C8S015	371731
C8SMV9	536019
C8SX15	536019
C8T8U5	667127
C8TBF1	667127
C8Z6C2	643680
C8ZGC6	643680
C8ZI69	643680
C8ZY47	565653
C9A795	565655
C9AVD3	565652
C9CJ24	565654
C9CV77	644076
C9D4A9	644076
C9KJ09	500635
C9KS70	483215
C9L6E1	537007
C9LU41	546271
C9MB75	656913
C9MG28	656912
C9MXF8	634994
C9MXF9	634994
C9N7C9	591167
C9NC11	591167
C9NMX8	675814
C9P1N8	675813
C9PF85	675811
C9PL75	675811
C9PML0	667128
C9Q6T2	675810
C9QMA2	675816
C9QS78	536056
C9RDQ0	579137
C9SFE9	526221
C9SML8	526221
C9SR17	526221
C9T5D7	520459

C9T8B9	520459
C9TFF6	520460
C9THJ4	520460
C9TPA6	520463
C9TR34	520463
C9TUF7	520461
C9TY22	520461
C9U2S0	520454
C9U527	520454
C9UBY9	520452
C9UE33	520452
C9UL65	520451
C9UNL5	520451
C9UX72	520450
C9UZL3	520450
C9V9L1	520456
C9VCC4	520456
C9VGX4	520457
C9VHZ0	520457
C9VRV6	520455
C9VT92	520455
C9WZN5	604162
C9X6A6	568708
C9Y8F0	667019
C9YAE3	667019
D0AWZ0	575591
D0B0W5	575591
D0B2B0	224914
D0B9M0	224914
D0BC68	520488
D0BEX9	520488
D0BW26	575564
D0CAH5	575584
D0CH66	166314
D0CPN4	644107
D0CQ42	644107
D0D0T6	501479
D0D9X1	501479
D0GDV2	520465
D0GG81	520465
D0GNC2	596323
D0GQJ8	675806
D0H242	675807
D0HHY3	675820
D0HTN7	675808
D0I2M5	675809
D0I8F3	675812
D0IMA5	675815
D0JEP3	637382
D0JP60	637385
D0KRH6	555311
D0P925	520489
D0PC10	520489
D0PK01	520487

D0PLR5	520487
D0RGJ9	437701
D0RJ59	437701
D0RNJ0	684719
D0RPU8	684719
D0RU58	469609
D0S5E6	575585
D0SD03	575586
D0SMY5	575587
D0SSD1	575588
D0T6Q2	575589
D0TGX7	469589
D0TXC5	469588
D0VY45	34305
D0W1D2	546262
D0W7D2	546265
D0WHP2	649764
D0WN48	649743
D0WT23	674977
D0X9C1	673519
D0XMR0	633149
D0Z099	675817
D0ZJU2	588858
D1CWL8	520449
D1CX49	520449
D1D5M4	528346
D1DD24	528352
D1DJ04	528354
D1DNV2	528356
D1DVS1	528355
D1E2H7	528358
D1E8T7	528359
D1EF95	528360
D1ENK7	520462
D1ERH3	520462
D1EWZ7	520464
D1EY96	520464
D1F7U6	520466
D1FA76	520466
D1FEL5	520458
D1FJD7	520458
D1GQK1	663951
D1GRM8	663951
D1JEY9	115547
D1JS94	469587
D1K0G2	457391
D1KBM1	655186
D1NJJ1	492476
D1NNE3	492476
D1NVW5	561180
D1P451	500637
D1PA78	537011
D1PMT1	411471
D1PZE7	585502

D1Q4Y8	545431
D1Q9L1	553594
D1QBP1	553594
D1QGK8	553601
D1QKM9	553601
D1R0D8	553574
D1R3Q2	553574
D1RPH9	682634
D1RX02	682634
D1S1G3	644283
D1S8Q5	644283
D1SQQ4	643561
D1SSJ8	643561
D1TU26	687916
D1U5I1	643562
D1U9X7	643562
D1URS6	640510
D1UTR7	640510
D1VNZ8	298654
D1VQS3	298654
D1WII5	596317
D1WZM9	649189
D1X323	649189
D1XDZ0	647653
D1XFE8	647653
D1Y9A0	679194
D1YNY4	686660
D1YVP6	304371
D2AHV4	591020
D2ALA8	510831
D2ATJ4	479432
D2CL18	54398
D2EQQ9	563037
D2ES80	585543
D2F5K8	585149
D2F8W5	585149
D2FFA3	585151
D2FNW9	585152
D2FU02	585159
D2FX95	585159
D2FZY3	585160
D2G2Z9	585160
D2G8D1	585161
D2GBG3	585161
D2GHZ3	585148
D2GP47	585150
D2GTH8	585150
D2L0J0	644968
D2L8F4	644968
D2LID2	648757
D2LII0	648757
D2M5L9	652103
D2MBT1	652103
D2MK46	700750

D2MVX9	683083
D2N8Y6	523796
D2NBN2	431946
D2T4H9	644651
D2U1R2	638
D2UP57	585155
D2UQS2	585147
D2UTD9	585147
D2UXY9	5762
D2YDD8	671074
D2YRQ7	671076
D2Z713	469381
D2Z9L2	500639
D2ZJG1	500639
D2ZNG4	521002
D2ZNY5	521002
D2ZTU9	546266
D3A670	546268
D3AIV9	566550
D3BWQ2	653733
D3BZ11	653733
D3C957	648999
D3C9C8	648999
D3D042	102897
D3D049	102897
D3EV70	703339
D3EY86	703339
D3FPI6	567106
D3H457	216592
D3I0W9	575611
D3L3K4	592015
D3LBM8	655225
D3LLV6	596312
D3LVK6	699218
D3M3L1	656024
D3M3M7	656024
D3MCE0	679195
D3MKB4	686659
D3MUY4	547146
D3MV04	547146
D3N2S1	640512
D3N9M6	640512
D3NIE5	663278
D3NM44	663278
D3R3B7	552531
D3RYJ8	589924
D3S6J7	644281
D3U0T3	118101
D4B0A2	663331
D4BF67	500640
D4BLX3	518634
D4C0G4	521000
D4CH96	411486
D4CNM6	608534

D4CVW3	546275
D4DIY3	663202
D4DS42	546263
D4DX80	667129
D4E7R6	667129
D4F239	500638
D4FM58	525375
D4FTC9	655813
D4FZN8	645657
D4IX80	657324
D4IX81	657324
D4JBR9	717962
D4JF95	717960
D4JH58	657317
D4JT95	657319
D4K0X5	718252
D4K851	657322
D4KGT6	657316
D4KKZ5	657315
D4L445	718255
D4L536	657321
D4LEP6	213810
D4LM70	657323
D4M638	657313
D4MM93	717961
D4N1A6	245018
D4PN54	393117
D4PV40	393131
D4Q362	401650
D4S1N7	511680
D4S4G1	585503
D4SAB8	585531
D4SD13	585531
D4SYR9	427081
D4T7B1	427082
D4TE40	533240
D4TLB2	533240
D4TNH2	533247
D4TQP9	533247
D4TXB9	649742
D4U2J0	553590
D4U765	553590
D4UDH8	553580
D4UHS5	553580
D4URP0	246199
D4V5Y9	702446
D4VF84	702447
D4WM24	702443
D4WY67	702444
D4X5T7	742159
D4XTN3	707232
D4XXV8	552811
D4XXW2	552811
D4Y3S6	634956

D4YKP2	585530
D4ZPE6	696747
D4ZWG6	696747
D5AK53	423211
D5AK54	423211
D5CV06	714962
D5EAA6	547558
D5GJ82	39416
D5HB86	761659
D5HB91	761659
D5HHT4	751585
D5KUG5	4932
D5MF72	671143
D5N152	703612
D5NDS7	243261
D5NJX7	243261
D5NVD1	649754
D5P919	525368
D5PKD7	525370
D5Q1E2	525259
D5QCA8	714995
D5QLC9	595536
D5QSZ7	595536
D5R4T5	642492
D5R5P5	642492
D5R5P6	642492
D5RIV0	525371
D5RW35	525258
D5S8A1	760570
D5SFP8	762948
D5TFC4	573236
D5VRC2	573063
D5VTV1	573063
D5WAV0	640511
D5XNL4	515617
D5Y9W2	520141
D5YLG0	520140
D5YXY3	515616
D5ZAT5	537209
D5ZVU9	566461
D6A3U0	566461
D6AAY9	457431
D6AQS9	457431
D6B052	457425
D6B8U3	457425
D6CSZ4	426114
D6DAL8	722911
D6DGK6	717608
D6DX46	718254
D6DYT4	657318
D6E6J3	657308
D6E7T8	657308
D6EEB7	457428
D6ERP4	457428

D6FTX5	611304
D6GI24	469608
D6GMK7	469608
D6GYG4	585156
D6H1R1	585156
D6H883	528348
D6HFH5	585144
D6HIN7	585144
D6HR67	552396
D6I4X0	550672
D6II37	550676
D6IJY3	656380
D6J254	585157
D6J5T7	550677
D6JM63	528351
D6JVN6	575565
D6K047	645465
D6K1X7	645465
D6KKJ8	457416
D6KRX8	450749
D6KY17	641147
D6LDS9	469621
D6LLV7	520448
D6LPN6	520448
D6LWE3	585154
D6LZR8	585154
D6M4H6	465543
D6SES1	548470
D6SFM0	548470
D6SQU8	555779
D6SS74	555779
D6T6U5	553577
D6T9R9	553577
D6TBI4	485913
D6TJ31	485913
D6UBW0	762962
D6UDK5	762962
D6UUE2	682795
D6V2N6	666684
D6V5Q6	666684
D6VJC6	596153
D6VJC7	596153
D6VRH0	559292
D6W2G7	559292
D6XBK2	463191
D7AJM9	663917
D7C5A7	749414
D7CCD1	749414
D7CX02	649638
D7E8M8	644295
D7EX23	515615
D7GZS5	520453
D7H2M2	520453
D7HPW6	412967

D7HX61	693985
D7IIG4	469585
D7IRY9	469592
D7J8R6	585544
D7JHP1	656379
D7KAE4	457390
D7KGE8	81972
D7KRQ1	81972
D7M1L9	81972
D7MZ21	641149
O04973	28526
O04974	28526
O59736	4896
O85070	118101
O87198	262724
P06208	4932
P48570	4932
P58898	98799
P94907	1126
Q01FR2	70448
Q01Z83	234267
Q02141	1360
Q05XR9	221359
Q065B6	313625
Q08ME7	378806
Q096Y3	378806
Q0CZN6	341663
Q0F2J9	314345
Q0FCE5	367336
Q0FG53	367336
Q0FL30	314265
Q0FMQ1	314265
Q0G5V7	314231
Q0HE65	60480
Q0IUQ0	39947
Q0YPL2	377431
Q0YQD3	377431
Q0YQD6	377431
Q12166	4932
Q12726	4952
Q12X21	259564
Q1IMI1	204669
Q1JRZ0	81972
Q1JRZ1	81972
Q1JRZ2	81972
Q1JRZ3	115915
Q1JRZ4	115915
Q1JRZ5	115915
Q1JRZ6	97979
Q1JRZ7	97979
Q1JRZ8	97979
Q1JRZ9	59691
Q1JS00	59691
Q1JS01	59691

Q1JY32	281689
Q1K340	281689
Q1N0C6	207949
Q1N6M9	207949
Q1NB58	314266
Q1NR58	262489
Q1NSC1	262489
Q1NYU8	374675
Q1Q0M4	174633
Q1Q5P8	174633
Q1UZJ8	314261
Q1V0A5	314261
Q1V6T3	314288
Q1VVM5	313595
Q1YLU2	287752
Q1YRP0	314287
Q1Z4R3	314280
Q1ZFE7	314282
Q1ZM14	314292
Q24M83	9
Q28QY6	290400
Q2B6M7	313627
Q2BKT8	207954
Q2BR03	207954
Q2BZP9	121723
Q2CD25	314256
Q2CGZ5	314256
Q2FKU8	323259
Q2FSZ7	323259
Q2H478	38033
Q2RAP8	39947
Q2VP65	115547
Q2Y4F2	115547
Q30DX9	69183
Q39891	3847
Q3EX79	339854
Q3R3J5	155920
Q3R938	155920
Q3RFR8	155919
Q46ET8	269797
Q4BZE1	165597
Q4C7B5	165597
Q4EH15	267410
Q4EQF2	267409
Q4HEA1	306254
Q4MSM6	269801
Q4P6Z8	5270
Q4ZHS8	77133
Q57926	2190
Q59YF1	5476
Q5AB86	5476
Q5FAL5	3712
Q5HHA8	93062
Q5KIZ5	5207

Q5SH16	300852
Q5WQ07	261317
Q5XQB0	285360
Q60D83	3712
Q60D84	3712
Q64D65	285362
Q6B1Y9	4932
Q6BW96	4959
Q6BY18	4959
Q6CIW1	28985
Q6CM17	28985
Q6FP59	5478
Q6FT78	5478
Q6GAU9	282459
Q6GIB0	282458
Q703X8	2271
Q71QG1	3712
Q7A1B0	196620
Q7A6G4	158879
Q847R4	35779
Q8PY62	2209
Q8RL85	1422
Q8TI92	2214
Q8TYM1	2320
Q8VX04	3702
Q99VB3	158878
Q9LPR4	3702
A0L6V8	156889
A1AUN8	338966
A1BHQ7	290317
A1K2W3	62928
A1WTR1	349124
A3PLR8	349101
A4J6Z6	349161
A4JRK9	269482
A4SFU4	290318
A4VJ96	379731
A4WRZ5	349102
A4YYU2	114615
A5ENS4	288000
A5FPD6	216389
A5G6V7	351605
A5N918	431943
A6LPH9	290402
A6LV02	290402
A7IBL6	78245
A8IIC6	438753
A8L6W7	298653
A9BIA5	403833
A9H5Z1	272568
B0TAQ1	498761
B0TDH7	498761
B0UAP6	426117
B1I3K4	477974

B1VMJ5	455632
B1WPB8	43989
B1Y707	395495
B2IEU7	395963
B2J701	63737
B2JYB9	391038
B2KBD6	445932
B2THY7	508765
B2UXP8	508767
B3DXX9	481448
B3E299	398767
B3EHJ3	290315
B3EL77	331678
B3QB34	395960
B3QQ09	517417
B3QSR5	517418
B4S9H2	290512
B4SC35	324925
B5ECQ1	404380
B5ER60	380394
B5XPG2	507522
B5YFP3	289376
B6IXL8	414684
B6YRB4	511995
B7JA83	243159
B7JWX3	41431
B7KG83	65393
B7TB41	713887
B8DR73	883
B8EJ12	395965
B8FAB3	439235
B8FP49	272564
B8FZP5	272564
B8HWE6	395961
B9E2F6	583346
B9KKS7	557760
B9M0S1	316067
C0QKL7	177437
C1DH20	322710
C4L9I8	595494
C5BTG0	377629
C6BX35	526222
C6C8U5	579405
C6E9A3	443144
C7LNR6	525897
C7QV24	395962
C7RQK4	522306
C8W230	485916
C8WE03	622759
C9XM62	645462
C9YJL2	645463
D1KS34	640131
D2BJU3	311424
D3DID5	608538

D3NSB3	137722
D3RTC3	572477
D3SKQ6	633145
D3SPG6	638303
D4GMW4	706191
D4H6W4	522772
D5ARX1	272942
D5CPA5	580332
D5EQN0	583355
D5V3P1	572480
D5WN34	640511
D6Z0A9	589865
D7CKQ7	643648
D7E3V7	551115
P58637	103690
Q07HZ4	316055
Q0AZZ0	335541
Q0RAV3	326424
Q13C65	316057
Q13N13	266265
Q18A29	272563
Q20Y13	316056
Q24PJ1	138119
Q24QM0	138119
Q2J1G8	316058
Q2J4F7	106370
Q2JP75	321332
Q2JTM1	321327
Q2RS26	269796
Q2W709	342108
Q39XU0	269799
Q3A2Q7	338963
Q3AD34	246194
Q3AR85	340177
Q3B2P8	319225
Q3J0I3	272943
Q3M5U0	240292
Q3M625	240292
Q3MEM4	240292
Q3Z640	243164
Q3ZW76	255470
Q44290	103690
Q47G84	159087
Q60C57	414
Q6D300	29471
Q6N102	1076
Q7M8U7	844
Q8KC96	1097
Q97MD7	1488
Q9RQC0	542
A0YP05	313612
A0ZAX8	313624
A1KYC7	43989
A1KYI9	309035

A1VPP0	365044
A2V893	167942
A3IL18	391612
A4TZW9	55518
B0AC54	445973
B1R1V6	447214
B4AZB2	497965
B4WR31	91464
B6G170	500633
C0GHS8	555088
C1IAR6	457396
C4IMK3	632245
C5RN57	573061
C5RQ83	573061
C5TI33	555217
C5UUQ4	536233
C6MRT0	443143
C6PCG7	580327
C8Q2L5	592316
C8S0S2	371731
D1WUS8	649189
D2M8F4	652103
D3BYV3	653733
D3DE76	102897
D3M538	656024
D3MPC2	596329
D4TIC9	533240
D4Y040	552811
D5N782	243261
D5Q163	525259
D5RWB3	525258
D6AQL6	457431
D6GAU9	469608
P05342	354
P05345	573
P23122	355
P54610	1857
Q00853	1501
Q01181	1063
Q07179	1061
Q0YUZ6	377431
Q0ZQ46	359131
Q1K1F5	281689
Q47884	1859
Q4C2Z5	165597
Q52070	549
Q53UA5	2698
Q7BKY3	1520
Q8RKT1	47227
Q9FA12	33996
Q9L4J8	1172
Q9L4J9	1172

**saccharopine dehydrogenase**	**saccharopine dehydrogenase**	**LysW**

(NADP+, L-glutamate forming)		(NAD+, L-lysine forming)			
**UniProt**	**TXID**	**UniProt**	**TXID**	**UniProt**	**TXID**
P38999	4932	P38998	4932	Q5SH22	300852
D6W1M5	559292	D6VVW5	559292	Q9ZND7	274
C7GLK7	574961	C7GLS2	574961	B7A8X3	498848
B5VR75	545124	B5VKX1	545124	D3PNS8	504728
B3LPJ3	285006	B3LTH6	285006	D7B9U5	526227
A6ZSC4	307796	A6ZVV4	307796	A1RR75	384616
C8ZFS9	643680	C8ZAT6	643680	A3MT39	410359
Q6FWR8	5478	C5DUQ8	559307	A4WJI1	340102
Q6CSQ4	28985	Q6FU27	5478	A4YD42	399549
A7TFY2	436907	C5DDF5	559295	A5US64	357808
C5DWU2	559307	Q6CP29	28985	A7NPM4	383372
B2G4L1	4956	A7TPI1	436907	A8ACC6	453591
B2G3V4	4956	A5E7N5	36914	A9A1K4	436308
B2G3U5	4956	A3GF76	4924	A9B808	316274
C5DC40	559295	Q6BUY2	4959	A9WD14	324602
C4R4U1	644223	C4Y8H9	306902	A9WD15	324602
Q75DA8	33169	B9WGJ1	573826	B1L529	374847
C4YP33	5476	C4QX51	644223	B1VMJ6	455632
Q5A2W3	5476	C5MDX3	294747	B1YB52	444157
C4Y7M8	306902	Q75BV4	33169	B8G9T3	326427
C5MI17	294747	P43065	5476	B8G9T4	326427
Q9C2G6	5141	C4YGU8	5476	B9KZQ2	309801
Q2HB62	38033	A5DQ14	4929	B9LI61	480224
Q9P4R4	148305	P38997	4952	B9LI62	480224
C5PG82	222929	Q7SFX6	5141	B9LTZ4	416348
A6SQH9	332648	B6K2C0	402676	C1CVR6	546414
Q4WQ27	5085	C9SQX9	526221	C3MJ47	429572
B0Y6W5	451804	B2AYG0	5145	C3MYT9	427317
Q5B1H9	162425	D5GCD9	39416	C3MZU4	427318
C8VG04	227321	Q09694	4896	C3N869	439386
C0NJU5	447093	D1ZGX9	5147	C3NF43	419942
A6QW18	339724	A7ELG9	665079	C4KJ27	426118
Q2UJT2	5062	Q0CNM1	341663	C7NPR1	519442
B8N3C2	332952	B2W3J7	426418	C7NZZ4	485914
B8LW01	441959	A6RLN2	332648	C7QK76	479433
B6QQG5	441960	Q4WY41	5085	D1B8S6	525903
O59711	4896	B0XXB5	451804	D1C6B3	479434
C5JXA5	559298	A1D6Q4	331117	D1CE81	525904
C0S6P3	482561	B6Q4A4	441960	D2PE56	425944
Q0UFZ0	13684	B6HC95	500485	D2RUT7	543526
C5FUG2	554155	Q0U4J1	13684	D3SUS8	547559
A5DLT8	4929	B8N1M4	332952	D4GYP2	309800
B0CXB5	486041	A2QDL3	425011	Q05E08	56636
Q4P245	5270	B8M547	441959	Q18E34	362976

D4DC41	663202
C5GWE1	559297
D2VWF2	5762
Q99K67	10090
Q3UEQ9	10090
Q3UWN2	10090
D4ACE9	10116
D4A608	10116
A2VCW9	10116
Q5XLU9	8022
Q6GN76	8355
B0W052	7176
Q5I0C2	8364
D7EHU4	7070
Q54NG9	44689
A7SDG1	45351
Q8H770	3635
A3XL75	398720
A3HXU2	388413
Q1VW87	313595
A4AVY8	313603
A5FK66	376686
A6ES54	50743
A4BZY1	313594
A3U5A5	216432
A9DKV3	391587
A8UJ36	391603
B7PT93	6945
C0BLE5	487797
B9NLJ0	467661
Q5LNA3	89184
D4ZD04	637905
C9CZ34	644076
C6WAW5	446462
B7QSN1	439497
Q0FRV0	314265
A6FLW8	351016
A3K518	388399
B4BQN3	495036
A4IK78	420246
A3I843	388400
A2QIZ4	425011
A3LQ43	4924
Q1GKH7	292414
Q6C844	4952
A1C5F9	5057
A1CKS0	5057
Q870G1	162425
C8VJB0	227321
A4RKX5	148305
Q2UKP1	5062
Q2HFZ2	38033
C5JV27	559298
C5GPV4	559297
D4ANC1	663331
C5FBR9	554155
C1H506	502779
Q5KHJ2	5207
A8N200	240176
C6HLP4	544712
A6QUT9	339724
C1L3C4	5127
A9UYX5	81824
C7Z0M9	660122
C5PF70	222929
B0CQP7	486041
C0NQ08	447093
D4D7C7	663202
C1GFV8	502780
C0SDQ1	482561
Q4P6J5	5270
C4JL09	336963
A8PQZ8	425265
D4ZD03	637905
A7S2W5	45351
C9CZ35	644076
D0CTY5	644107
A6FLW9	351016
Q1GKH6	292414
D2V3E3	5762
A1B5J9	318586
A3XDP7	314262
B6BEW9	439496
B9NLJ1	467661
A3STM1	314267
A3PND5	349101
Q6LIA6	74109
B9KP22	557760
Q3IYW2	272943
Q1Z6I5	314280
A3S8Q8	52598
A3VXP5	314264
Q1IZ85	319795
Q3IME0	348780
Q4JAQ0	2285
Q5JFV9	311400
Q5UZ48	2238
Q6KYZ4	263820
Q72HE5	262724
Q88Z74	1590
Q8U0B8	2261
Q8ZU98	13773
Q976J8	111955
Q980W8	2287
A0RXL0	46770
A8M8Q7	397948
B3T3B2	455568
B5GG72	465543
C1V897	469382
C9N7U4	591167
D0KRK5	555311
D1WUS7	649189
D2Z853	469381
D7B9U6	526227

A1CI66	5057	A3V2U7	314232
A1CWN5	331117	Q2CKD5	314256
A2QB21	425011	B7QSN0	439497
A5E050	36914	A6E4H9	391613
A7F6I4	665079	Q0FFN0	367336
A8P1I1	240176	A4WWR4	349102
A8PXU8	425265	A9DXX4	391624
A9V958	81824	A9HX34	391595
B2B555	5145	Q5LNA5	89184
B2VUT2	426418	Q16C99	375451
B6HV69	500485	A4ESV1	388739
B6JXD3	402676	B6AV24	314270
B8NIE7	332952	A3SLD2	89187
B9WEP0	573826	A4ELZ0	391593
C1G452	502780	B7RKP5	391589
C1GVZ8	502779	Q28K30	290400
C6HIX0	544712	A9FD76	383629
C7YTT0	660122	A9GJL6	391619
C9SG84	526221	B5K5L8	391616
D0CTY4	644107	Q0FRV1	314265
D4B1K0	663331	B5IZ49	391626
D5GD57	39416	A3K517	388399
Q0D1W0	341663	A3JMZ7	388401
Q2UNU0	5062	B5I7U4	463191
Q5KEA8	5207	Q82M72	33903
Q6B170	4932	C9ZBN4	680198
Q6BL02	4959	A8M4N3	391037
Q96TW2	5076	C6WAW4	446462
		D5WN65	640511
		C0BMF0	487797
		A4CM31	313596
		C3YJJ0	7739
		C3ZFX2	7739
		Q9UDR5	9606
		Q9VLX0	7227
		A2VCW9	10116
		A4D0W4	9606
		A8E657	9913
		A8XI56	6238
		B0W052	7176
		B3MV19	7217
		B3N6N9	7220
		B4G8F0	7234
		B4HYA5	7238
		B4JA33	7222
		B4KGK4	7230

B4LSS5	7244
B4MWK8	7260
B4NZG7	7245
B4Q5T6	7240
C9K2E7	44386
D7EHU4	7070
O44503	6239
Q16FJ9	7159
Q29N04	46245
Q3UEQ9	10090
Q3UWN2	10090
Q5I0C2	8364
Q5XLU9	8022
Q6GN76	8355
Q99K67	10090

**LysX**		**LysZ**		**LysY**	
**UniProt**	**TXID**	**UniProt**	**TXID**	**UniProt**	**TXID**
Q5SH23	300852	Q5SH27	300852	Q5SH26	300852
Q72HE6	262724	O50147	262724	O50146	262724
O50144	274	B7A8X6	498848	B7A8X5	498848
B7A8X4	498848	D7BIA1	526227	D7BIA2	526227
D7B9U4	526227	D3PNS4	504728	D3PNS6	504728
D3PNS7	504728	C1CUR7	546414	Q1J0J7	319795
Q1IZ86	319795	D7CX13	649638	D7CX09	649638
C1CVR5	546414	Q9RUG6	1299	C1CUQ6	546414
D7CX06	649638	Q1J0K4	319795	Q9RVQ9	1299
D1CE82	525904	B8GBE4	326427	B9KZP8	309801
D1CE84	525904	A7NPM0	383372	D1CE85	525904
Q9RSD1	1299	B9LAR5	480224	D1C6B7	479434
B9LI63	480224	A9WIL8	324602	B9LI64	480224
A9WD16	324602	D1CE86	525904	A9WD17	324602
B8G9T2	326427	A5US68	357808	A5US66	357808
D1C955	479434	B9KZP6	309801	D6TDS1	485913
A7NPM3	383372	A9B811	316274	B8G9T1	326427
A5US65	357808	D1C6B9	479434	A9B810	316274
D6TDS2	485913	D6TDS0	485913	A7NPM2	383372
A9B809	316274	Q3IMD7	348780	D4GYP0	309800
B9L301	309801	Q5UZ51	2238	B5HTW4	463191
D3SUS7	547559	C7NP88	519442	D6K9G9	645465
D2RUT6	543526	B5GG75	465543	D2RUT5	543526
D6K9H0	645465	D3SUS5	547559	B9LTZ2	416348
D4GYP1	309800	D2RUT4	543526	D3SUS6	547559
C1V898	469382	Q18E31	362976	D1XEU1	647653
B9LTZ3	416348	C7NZZ7	485914	Q3IMD8	348780
C7NP90	519442	D4GYN9	309800	C7NP89	519442

Q3IMD9	348780	B3T3A9	455568	Q5UZ50	2238
D3L663	592015	C1V8A0	469382	Q18E32	362976
D3L1N9	592015	A9A1K7	436308	A4YD39	399549
D1B8S7	525903	B9LTZ1	416348	Q980X1	2287
Q5UZ49	2238	C7QK85	479433	D0KRK2	555311
C7NZZ5	485914	D3L1P1	592015	D2PE59	425944
B5GG73	465543	A0RWW1	46770	C3NF40	419942
Q18E33	362976	C9N7U1	591167	C3N872	439386
B1L530	374847	B4XYB4	285525	C3MJ50	429572
D2Z854	469381	B5HTW3	463191	Q8ZUA0	13773
Q8U482	2261	D2Z856	469381	C4KJ30	426118
Q8U0B7	2261	A1RR74	384616	C3MZY9	427318
Q5JFW0	311400	A3MT40	410359	C3MYU2	427317
A9A1L2	436308	Q2RG64	264732	B5GG74	465543
A9A1K5	436308	B1L528	374847	Q9V1I6	29292
O59396	53953	A8M8Q8	397948	C7QK84	479433
C7QK69	479433	D4L5Y8	657321	Q976J5	111955
Q9V1I7	29292	Q4JAQ2	2285	Q8U0B6	2261
B3TAF0	455603	Q2NGV4	339860	Q5JFW1	311400
B3T4B0	455574	B1YB53	444157	B3T3B0	455568
A0RTV2	46770	D5XAD7	635013	C7NZZ6	485914
A0RW42	46770	Q8ZU99	13773	O59397	53953
B3T3B1	455568	A4YD40	399549	A1RR73	384616
C9N7U3	591167	Q8TUX2	2320	Q4JAQ3	2285
B4XYC2	285525	C6PAB9	580327	B1YB54	444157
B5HTZ2	463191	B9RYI2	3988	C1V899	469382
C3MZU3	427318	B9T9H8	3988	A3MT41	410359
C3MYT8	427317	D4UJF2	246199	A9A1K6	436308
D2PE55	425944	Q5JFW2	311400	A4WJH9	340102
C4KJ26	426118	C9KK90	500635	A0RWW0	46770
C3N868	439386	O28988	2234	A8M8Q9	397948
C3MJ46	429572	A4WJI0	340102	C9N7U2	591167
C3NF44	419942	D2RDX4	572546	D2Z855	469381
D1XEU0	647653	A6UR56	406327	B1L527	374847
A8A8I9	453591	D3S0X1	589924	D3L1P0	592015
D0KRK6	555311	O26285	187420	A8AAF8	453591
A4YD43	399549	D1B8T2	525903	Q6KYZ5	263820
Q976J9	111955	D5RZU8	525258	B4XYB3	285525
Q7SI95	2287	D5Q3T6	525259	D0LU59	502025
A1RU22	384616	D5EC30	547558	Q096B0	378806
A1RTS1	384616	A9A8M9	444158	Q9YBY8	56636
A4WHN7	340102	D7ASW6	583358	D1WUS5	649189
A4WH93	340102	C7HLW6	588857	D1B8T1	525903
A3MVY3	410359	C5RRN9	573062	B1VMJ8	455632
A3MSD6	410359	B0K4D4	399726	D1NAS7	340101
B1YB81	444157	C5UCF4	509193	D0MH57	518766

B1YAL4	444157	B0KBW1	340099	A5CSI9	443906
Q8ZXK1	13773	Q8U0B5	2261	B0RHD0	31964
Q8ZY47	13773	C9YMY6	645463	C2CPJ6	525268
Q6KYZ8	263820	C9XJQ3	645462		
Q4JAP9	2285	Q188A2	272563		
A8M8Q6	397948	Q6B8Z0	285951		
Q9YBY7	56636	Q46D93	269797		
A0B877	349307	A4G0C7	402880		
Q8TKX5	2214	Q6M154	39152		
Q466V5	269797	D3T5L2	580331		
Q6LZC7	39152	D2RIT8	591001		
D7DS28	456320	A6VI50	426368		
Q8Q0M5	2209	A7VCH7	411489		
D1JIG7	115547	Q0AUM4	335541		
A6UUH7	419665	Q8PXJ8	2209		
A6USK0	406327	Q8THJ9	2214		
B3TCF1	455612	C6MF16	153948		
C7P665	573064	O59398	53953		
D5E7D9	547558	B6C5S6	473788		
A6VK09	426368	Q3J6V9	323261		
A4FY98	402880	A9KHN3	357809		
A9A7Q7	444158	Q97GH8	1488		
Q58037	2190	C7ITI9	589861		
B5HEI5	457429	A8AA51	453591		
D5MJV6	671143	Q1GZ15	265072		
Q12WH9	259564	B8GTE7	396588		
B1VMJ7	455632	B8GLR6	396588		
D1WUS6	649189	Q7NR63	536		
C9RGE7	579137	Q7NSR5	536		
Q1YFX7	287752	C1EFW2	296587		
Q4JIT4	333425	A8SYH7	411474		
D5C3I2	472759	A6UT97	419665		
D7A5F6	639283	C0QYU9	565034		
D7E6J7	644295	C4KJ29	426118		
D2QWP6	530564	C3N871	439386		
Q4JIN4	333372	C3MZY8	427318		
D3S0R0	589924	C3MYU1	427317		
D5SQ35	521674	C3MJ49	429572		
Q1DFE7	246197	D2PE58	425944		
Q1D7V4	246197	D2ZMH7	521002		
Q88Z73	1590	B9AH57	483214		
D5VR43	573063	A5UK52	420247		
C0N3H5	637616	C8W633	485916		
D6SMD0	555779	D5HH43	751585		
D6SMI0	555779	D7E9X1	644295		
A2SJ25	420662	Q0ADI4	335283		

D3S4E9	644281	Q980X0	2287
A7JPP7	272629	D0KRK3	555311
A2BIU1	415426	C5RKJ3	573061
A9KNJ7	357809	C7N8R2	523794
Q4BZD1	165597	C6X9A3	582744
Q1ZUU0	314292	D5U6S6	526224
Q1ZKN6	314292	D0KWP5	555778
Q099S1	378806	C3NF41	419942
Q08SZ3	378806	A9BEC0	93059
Q65UJ6	221988	B9NA36	3694
B1XQE7	32049	B9P734	3694
D5CFE9	716541	C5UXM5	536233
C9NNL8	675814	A8RJD5	411902
D2ZAA9	500639	Q9V1I5	29292
Q30PW7	326298	C6WYH2	583345
B8KAC1	391586	Q3A9W2	246194
B8KB21	391586	A5KJM5	411460
Q3JEF7	323261	Q2JHF7	321332
B6C344	473788	C4IM51	632245
A8AIR5	290338	B1QZ98	447214
D5QL58	595536	B2UYI4	508767
P45241	727	D3RRI4	572477
C9MGR4	656912	D1YZV3	304371
C9QMM9	675816	A7VYH2	428125
A4C713	87626	D7CJG3	643648
A4C7D6	87626	B0TCA8	498761
A4CEZ0	87626	B1C853	445971
B3GX62	537457	C0WEM3	563191
A3MZK2	416269	Q0VT62	393595
C6MY82	658187	D3AQH7	566550
B7LN16	585054	D1PRJ4	411471
B1ZIC6	441620	Q2YA02	323848
C9PBD2	675811	A6M1Z8	290402
D7DAJ8	591019	C3MF41	394
A4NRJ0	374933	D4DPG2	546263
B7KXE0	440085	B0CAM3	329726
A9W3S5	419610	Q1WLF7	382
B4B4R3	497965	Q92SG0	382
Q8KMJ4	661410	D7DV65	456320
A0Y2F4	156578	Q1XDF8	2788
C4F183	521005	P59299	173
C5B143	272630	D5C3A9	472759
A4NMR8	374932	B9J8D8	311403
A5Z789	411463	B7K814	65393
A5Z784	411463	D7DMJ3	666681
Q3IG57	326442	D1KDM1	655186

Q9HTZ2	287	B2IX33	63737
Q02EG7	208963	Q2JXF5	321327
B7V3U4	557722	D5VSU2	573063
A6VDX3	381754	Q1MM66	216596
A3LIT0	350703	Q2KD36	347834
A3L267	350704	B0MCG0	411490
D4BC08	500640	C4FP41	546273
C9Y013	413502	Q12ZP8	259564
A7H686	404589	A4J169	349161
A7HCI8	404589	C6AZ71	395491
A7HC70	404589	Q2FTZ8	323259
B5FBB1	388396	B3Q031	491916
A7MF38	290339	Q31ED4	317025
A4N9R9	375177	D1JER2	115547
D2TQG7	637910	P69366	2786
B0BU89	434271	P69365	2787
C4F348	521004	D1AJ75	526218
A3DMI7	399550	A5ZVL7	411459
A5UEH4	374931	D4LUR6	657314
D6DUS5	718254	Q7NEE9	33072
C8MFP6	553583	C7RK63	522306
D0KB49	561231	C5XRY3	4558
A4NFG1	374928	C5YDH0	4558
Q9FA40	408	Q976J6	111955
D1MX69	288	B0G879	411461
A5UCC6	374930	D3A7K9	546268
Q9CMJ8	747	B4X0G7	236097
Q5E743	312309	Q8R7C0	119072
C9MF42	656913	B7R645	391606
B3PCD4	498211	B1I1C7	477974
A6CF71	344747	B1BZS8	428126
Q6R3H8	107634	C9LRW1	546271
Q4QKP3	281310	B0SU21	456481
Q0G4J6	314231	B0SIF3	355278
A1U360	351348	B8GFN4	521011
C1M9F6	469595	Q603M3	414
B8GEE4	521011	Q606D4	414
A5KT07	443342	A4SBC5	436017
D4E8J2	667129	B0PD03	445972
Q2C0C6	121723	C5EPV7	457421
A4W8L7	399742	B9Z8B8	279714
A4N0R1	374927	B9Z6H2	279714
D3HLC6	661367	B4FSD4	4577
D1RJG4	638315	B6U787	4577
C6DEP6	561230	B6TEB2	4577
C9Q698	675810	C0N8P6	637616

Q6D3R5	29471	A6U5K5	366394
C6VRW5	471854	C5TNA2	596320
Q9KPT1	666	D3E2W9	634498
D7HQF5	412967	A8UV02	392423
D7HE67	345074	A8V071	392423
D0HXJ9	675809	B6FJL5	500632
D0H4U1	675807	B2TQ27	508765
C6S2V0	661513	D4S8Q5	585503
A8ACC7	453591	B8I8A5	394503
B9KZP9	309801	B4VRU9	118168
C3MQE2	429572	A0NSE3	384765
C3MW20	427317	C6PQM1	536227
C3N663	427318	C9L6Z0	537007
C3NEL9	439386	D4TUK4	533247
C3NH24	419942	D4THV1	533240
C4KHM6	426118	Q60382	2190
D1C6B6	479434	D3S7W9	644281
D2PCE1	425944	Q5KSL5	39947
Q970U6	111955	Q6H6G1	39947
A4YI79	399549	A3A9S7	39947
D0KT52	555311	Q7XU28	39947
Q4J8E7	2285	C8PVS4	553217
Q9UX35	2287	Q949B4	4530

**LysJ**		**LysK**		**dihydrodipicolinate synthase**	
**UniProt**	**TXID**	**UniProt**	**TXID**	**UniProt**	**TXID**
Q5SHH5	300852	Q5SHH3	300852	Q9FVC8	3702
Q93R93	262724	Q8VUS5	262724	Q0WSN6	3702
B7A5Y2	498848	B7A5Y1	498848	Q9LZX6	3702
D7BI15	526227	D3PKC7	504728	D7LCJ3	81972
Q1IYH3	319795	D7BI14	526227	B9S6U4	3988
Q9RW75	1299	D7CX18	649638	B9GPI1	3694
C1CWU6	546414	Q1IYJ8	319795	B9IC12	3694
A9B812	316274	A7NPL3	383372	Q42948	4097
D7CX15	649638	A5UWU3	357808	C6TM29	3847
B9LF89	480224	Q9RUH3	1299	C6TG34	3847
A9WB46	324602	C1CX65	546414	Q42800	3847
D1CE87	525904	B9LBG7	480224	B8LQ02	3332
B8GCW9	326427	A9WJY2	324602	P24846	4565
D6TDR9	485913	B8GCL9	326427	P24847	4565
A5UWU2	357808	B9KZQ0	309801	Q0JEK1	39947
B9KZP5	309801	D1CE83	525904	Q7XM43	39947
A7NPL4	383372	D1C6B5	479434	A3ARP8	39947
D1C6C0	479434	A9B813	316274	Q7XPB5	39947
Q3IMD6	348780	Q8U0B3	2261	Q01HC8	4530
Q976K0	111955	C9N7U0	591167	Q9LWB9	4530

Q12U07	259564	Q9V1I3	29292	A2XWN2	39946
A4YD44	399549	O59402	53953	B8ARJ0	39946
Q4JAP8	2285	Q5JFW4	311400	Q1PDD5	58934
A9A1K8	436308	D6XAH2	463191	A9SGY8	145481
D3S5L1	644281	C1V8A2	469382	A9RR71	145481
D7EBQ7	644295	C7QK68	479433	A8I3W3	3055
Q8PX16	2209	B4XYB5	285525	A4RX84	436017
C7NZZ8	485914	C7NZZ9	485914	C1MLG6	564608
C9RFZ2	579137	Q3IMD5	348780	A0XYC9	156578
Q0W469	351160	Q18E29	362976	A4C4H7	87626
C7NP87	519442	A9A1L3	436308	C6X6Q1	582744
A0RWW2	46770	D4GYN7	309800	Q2KWY6	360910
Q7SI94	2287	C8MFP2	553583	D3PAV3	639282
D0KRK7	555311	B3T4A9	455574	A1U1A6	351348
Q58131	2190	B3TAE9	455603	B5YKK4	289376
Q8TUE8	2214	B3TCE9	455612	D0CFC3	575584
C4KJ25	426118	Q5UZ53	2238	B7I287	480119
C3NF45	419942	B9LTY9	416348	B7GV32	557600
D2PE54	425944	D6K9G7	645465	B2I2E1	405416
C3N867	439386	B5GG76	465543	B0VPX3	509170
C3MYT7	427317	C7NP86	519442	B0VA02	509173
C3MJ45	429572	D2RUT2	543526	A3MA68	400667
C3MZU2	427318	B5HEI1	457429	D0C463	575564
Q1PX83	174633	A0RTV3	46770	Q3ACY3	246194
B3T3A8	455568	Q88Z70	1590	Q60B13	414
D5VSV8	573063	D3SUS3	547559	C9RDP4	579137
Q46E47	269797	D1XET4	647653	D0S542	575585
Q5UZ52	2238	D3L1P3	592015	A7VTW0	428125
D1YVL2	304371	Q6KYZ9	263820	A7VX17	428125
D4GYN8	309800	D2Z858	469381	C4L2D2	360911
B9LTZ0	416348	D1B8T4	525903	C8WA08	521095
C7P8K0	573064	A8MAC2	397948	C7TFF5	568704
Q18E30	362976	B1L532	374847	C2JTM0	525361
D5E9X0	547558	Q4JAP7	2285	Q76E52	17
A0B5Y4	349307	Q9YBY3	56636	D6JW95	575565
D3FVI6	398511	A8AA94	453591	C1DQP7	322710
Q8R7C1	119072	Q980W5	2287	D4EGM7	694569
B7R646	391606	D0KRK8	555311	C9R4Y0	668336
A9AAK5	444158	A4WJ85	340102	B5QQ65	486408
D3SUS4	547559	Q8ZUG2	13773	Q82SD7	915
D2RUT3	543526	Q976K1	111955	C7T8R1	568703
O30156	2234	C3NF46	419942	C7NIU8	478801
O27392	187420	C3N866	439386	Q2BHH0	207954
Q9K8V5	86665	C3MYT6	427317	Q2Y8S8	323848
A8MCU6	397948	C3MJ44	429572	D3RNG1	572477
A6UPB2	406327	C3MZU1	427318	D5R626	642492

A6VG46	426368	D2PE53	425944	D5R2T4	642492
B5YJU8	289376	C4KJ24	426118	D3L1V3	592015
C1V8A1	469382	A4YD45	399549	Q88NH2	160488
A4FX68	402880	A1RUC2	384616	C6BSH4	526222
A9VG69	315730	B1YBH4	444157	A5VZW8	351746
D6UNY1	682795	Q0W5T9	351160	Q1I6H5	384676
Q67KD4	2734	B5E9K5	404380	C5NUT8	546270
A6UUQ2	419665	A3MVL6	410359	D0T7Y7	575589
A3HVZ0	388413	C6E2X3	443144	C6RJF3	596318
C9RY11	544556	C6MVA5	443143	C6LFD1	478749
C3J1U5	550542	D5WWB0	562970	C6LB88	478749
A7GSF1	315749	D5WQV4	562970	A8RZS7	411902
Q731G6	222523	C8NVI2	585529	A8RUZ7	411902
B4BII4	495036	A4QD73	340322	A3CVI7	368407
A4IL49	420246	Q59284	1718	B0KSY3	76869
Q8ZV07	13773	D0WNP3	649743	Q3KGJ2	205922
A4WKP7	340102	C0VT95	548477	A9DKW4	314608
A6TTJ0	293826	D1Z2T1	304371	C3KED4	216595
Q6LY90	39152	A5G9L2	351605	C6MBX7	153948
B5V757	451708	C2GIV4	548478	A7B7M3	411470
D7D1S9	691437	C0WFI4	525260	Q8F132	173
A3MSV3	410359	D5NZC4	649754	A3VB56	314271
D2C831	590168	D0MJ50	518766	D5CLS3	580332
O66442	63363	Q093A1	378806	D1U3I3	643562
Q4MMY1	269801	Q09E40	378806	A3IT21	391612
A8ZW63	96561	D6Z638	589865	D3SNF3	638303
A1RVT8	384616	Q1D263	246197	D7HXD8	693985
D7DTP3	456320	C0D6K6	518636	D7I2D1	693985
C9R9D7	429009	C0D7V9	518636	D3DIN3	608538
C5CHX0	521045	B1MLJ7	561007	B3PC19	498211
C7ITJ0	589861	A5DDR6	4929	D4ZJN9	637905
B6FJL4	500632	D7HUX0	693985	D2LZH9	649639
D7AM62	663917	D7I6V5	693985	D2LSB9	649639
C7HLW7	588857	D7HVL8	693985	D4L875	657321
C5RRP0	573062	Q2LXL7	56780	C4FJZ6	432331
B0K4D5	399726	Q4ZZU6	205918	Q48LD5	264730
D2ZNL7	521002	Q500E9	205918	D0SP20	575587
Q74GU3	35554	Q4ZYE3	205918	A1WZ50	349124
B1Y8L7	444157			Q1WU86	362948
C0GH80	555088			C2EEY0	525364
Q2LT99	56780			B0PB24	445972
C5UCF3	509193			D7LRV3	81972
B0KBW2	340099			C5Z796	4558
Q97GH9	1488			C5YEF4	4558
B9IXC6	361100			B4F8D4	4577
A5UMZ5	420247			P26259	4577

Q5L1V2	1462	Q39535	4505
D4YA57	634956	Q019N6	70448
C6QLP0	581103	Q3IC52	326442
B9AG10	483214	Q47XX2	167879
Q9X2A5	2336	A3WKW6	314276
B7REP3	443254	Q3SJU8	292415
A5ILL6	390874	D3G0R4	398511
Q3A9W3	246194	D3FTZ0	398511
B7HNP6	405534	Q5QU03	135577
Q8U0B4	2261	D4XGH7	742159
B5YAL6	309799	A8U9V9	333990
A3CWS7	368407	B4BIY4	495036
D2RDX8	572546	B4BN30	495036
A5KJM6	411460	A4IMF1	420246
B5USS9	405533	A4ISN1	420246
Q2NI52	339860	C9S0E0	544556
Q6GKC1	282458	C9RV39	544556
Q6GID1	282458	C3J5C2	550542
D6SE09	548470	C3J5R4	550542
D6SFP0	548470	D7CZH8	691437
D6LUB8	585154	D7D0J3	691437
D6LW47	585154	A9HXM3	340100
D6J403	585157	D4Y4Q2	634956
D6IYV1	585157	D4YCI7	634956
D6HDI3	585144	C6QMF0	581103
D6HFF6	585144	C6QUA5	581103
D6H2X8	585156	Q2B0K6	313627
D6GYE5	585156	D2Z760	469381
D2UW78	585147	B4ADT3	536229
D2UQQ3	585147	C6WYD7	583345
D2GN00	585150	A8FDE6	315750
D2GP27	585150	A8FHB5	315750
D2GIL4	585148	A4G724	204773
D2GES0	585148	B2UMF2	349741
D2G691	585161	C6NYU0	637389
D2G8B2	585161	Q7W884	519
D2G595	585160	Q7VXZ8	520
D2FZW4	585160	D3SDV5	396595
D2FRZ7	585159	Q5MZT3	269084
D2FTY2	585159	Q31M42	1140
D2FHM8	585151	C5V6T0	395494
D2FBQ1	585151	Q7WLT9	518
D2F3K4	585149	C5D9E4	471223
D2F572	585149	D7DM41	666681
D1GM11	663951	Q93RJ8	406
D1GRD1	663951	B2JIV8	391038

C8ALT2	585158
C8AQJ3	585158
C8AG34	585153
C8AI51	585153
C8A7K3	585146
C8AAH7	585146
C8A170	585145
C8A308	585145
C7ZTI9	585143
C7ZVH0	585143
C2G705	548473
C2G977	548473
C3JQR6	596309
D7ASW5	583358
D3T5L1	580331
D2N3P0	523796
D2N5R9	523796
C1A054	234621
C5D767	471223
A4J170	349161
D2PM78	479435
C5QEC1	548475
C5QHR0	548475
C4Z4C5	515620
Q8NYM5	196620
P60299	196620
Q6GCU1	282459
Q6GAW9	282459
Q5HJI8	93062
Q5HHC8	93062
Q2G1H3	93061
Q2FZU6	93061
D4U5U0	553590
D4U8Z6	553590
D1Q8E3	553594
D1Q9J1	553594
D1P0J6	500637
C8KQE4	455227
C8KUA7	455227
C8KIW3	452948
C8KLW2	452948
C5Q443	548474
C5PY84	548474
D2UIW2	585155
D2UKS2	585155
D2FIN0	585152
A6EWS7	443152
B1XL16	32049
B3DY26	481448
A5GD89	351605
Q5L0H4	1462
Q5KVG9	1462
B0JL91	449447
A7Z4U8	326423
Q9KC32	86665
Q9KA91	86665
Q2SCM7	349521
Q2S9K4	349521
C7RSI3	522306
Q0AI27	335283
Q1H2N2	265072
B8Y632	1471
D6Z6B7	589865
D4FWS6	645657
B7GG61	491915
A6SXH5	375286
D5MZ25	703612
D0WHJ0	649764
A0ZER9	313624
Q04796	1423
C4G2G1	592010
A1ATI8	338966
B0SL58	456481
B0SCT0	355278
Q8DJK4	197221
A1US27	360095
B9Z1L2	279714
C5CHX9	521045
Q2SXB3	271848
Q65JG7	279010
Q65F34	279010
Q65EJ2	279010
A6CQ70	161544
D3BWB3	653733
A6DKZ4	313628
Q21HE7	203122
B1YSQ0	398577
B1Z3T9	398577
B1TEF9	396597
B1FEF0	396596
Q8YQY1	103690
D5W928	640511

D2FKI0	585152	C7QW46	395962
D6UEF0	762962	B7JW78	41431
D6UBX9	762962	B1JDB7	390235
A8Z0F0	451516	Q0VRH4	393595
A8Z063	451516	A4JFX3	269482
D1AJ76	526218	D1MH64	208964
C6D4U8	324057	B6BWH8	314607
C6CWH2	324057	B5WST8	516466
D2LY46	649639	Q9I4W3	287
Q07907	1422	Q02IH5	208963
B8GIB1	521011	B7UXT3	557722
C8LB01	553567	A3LMP3	350703
C8LCF5	553567	A3L5Q8	350704
Q635F3	288681	A8YMD4	267872
B8E0N6	515635	Q13X19	266265
B7JLY4	405535	D3HPZ3	661367
Q2RG65	264732	D1RHY5	638315
B3ZVK7	451709	Q0A5S1	187272
C6PAB8	580327	B9C7S5	513053
A0RIC9	412694	B9BZY0	513052
D5WRY7	562970	B9BE04	513051
C5N161	450394	A9AHL4	395019
C5N437	450394	Q0BDT5	339670
D4FUZ0	645657	C6AK98	634176
D5SR97	521674	Q1ZCA3	314282
Q3Z729	243164	Q67P59	2734
B7HB01	405532	A6V9K4	381754
C0BVS9	553973	Q3M723	240292
C0C3U3	553973	A2VX57	350702
A6QDG9	426430	D5EC78	572547
A6QFG7	426430	Q1BHQ8	331271
B1LAN9	126740	Q1BVB3	331271
B3Z662	451707	A0K8L9	331272
B3YVY5	405917	A0KCW1	331272
A9BIA0	403833	B1JUX4	406425
P60296	158879	B1K9V2	406425
P60298	158879	C5EGS9	457421
P60295	158878	C5EU98	457421
P60297	158878	D0WAK2	546265
D6T5F7	553577	D3UN22	683837
D6T6S4	553577	Q1QTP6	290398
D4UCC9	553580	A6D078	391591
D4UDF7	553580	D1DVH8	528355
D3ES13	703339	D1D8X1	528352
D3EV50	703339	D6DIN8	717608
D1QYH8	553574	D4CA91	411486

D1R0B9	553574
D1QEC3	553601
D1QGI9	553601
D0K7U2	681288
D0K3M5	681288
C8N122	553596
C8N4S9	553596
C8MTK6	553592
C8MRE0	553592
C8MMH3	553588
C8MPL1	553588
C8M968	553581
C8M6F7	553581
C8M1I2	553573
C8LWX6	553573
C8LUB4	553571
C8LUV1	553571
C8LHV6	553568
C8LM31	553568
C8L756	553565
C8L6I0	553565
A7WXH6	418127
A7X0H0	418127
A6TXW9	359787
A6U064	359787
A5IP54	359786
A5IRD5	359786
O59401	53953
Q81M98	1392
C8MFZ0	553583
C8MG49	553583
C3P7R5	592021
C3LJQ6	568206
B3J2X8	405536
B1US03	486622
B1GIN4	486620
B1EWQ2	486623
B0QH41	486621
B0Q342	486619
B0ASS2	486624
Q4HNL7	306264
D5TWX0	714359
P36839	1423
Q2YUZ5	273036
Q2YWS9	273036
C1ER03	572264
C4KQI4	536230
C6SF52	663926
A1KTJ9	272831
Q63SR2	28450
Q62J21	13373
Q3JQR8	320372
C6TTP0	320371
C5ZJN1	357347
C5NFJ2	436115
C4AR21	320390
B7CLW6	557724
B2H9U2	331109
B1HDV3	320374
A9K9G1	412021
A8KVU3	331978
A8EC25	360118
A5XYN5	334803
A5XH85	334802
A5TJS9	370895
A4LDD6	425067
A3NX01	357348
A3NB70	320373
A3ML64	320389
A2SAV9	412022
A1V5I9	320388
Q72AX2	882
C5TVE0	573059
A1VCZ9	391774
C4GKY1	629741
Q1N0S5	207949
Q1MPX7	363253
B4W098	118168
B4WY53	236097
A2W938	350701
Q473F1	264198
P0A6L3	623
D2AGS4	591020
C9QPU6	536056
C8UE47	585396
C8TVH4	573235
C4ZX53	595496
B7M7I1	585034
B1XAF0	316385
B1IWI1	481805
D1E285	528358
C9RB84	429009

C6M311	547045
A5GDA2	351605
Q0SI55	101510
A3TLW8	313589
A4T9W7	350054
D4LZ95	657313
D0KB91	561231
A8RJD7	411902
B7IWP3	405531
Q3EYI4	339854
Q818W2	226900
A8AB22	453591
B1L531	374847
Q5JFW3	311400
Q6KYZ7	263820
Q9V1I4	29292
Q9YBY6	56636

D0W0Y0	546262
D1DIR0	528354
C1HXA4	528345
C6S6J8	662598
D1E8J7	528359
D1D5D3	528346
C6CC66	579405
B9K865	309803
Q0KCC6	381666
B4RL41	521006
B2IT87	63737
A0AIN8	386043
A0AX47	331272
A0AZP9	331272
A0B3X0	331272
A0B4G1	331272
A0B7E1	349307
A0JRB5	290399
A0JTV2	290399
A0JUX6	290399
A0KB17	331272
A0KCE3	331272
A0KCT3	331272
A0KGP3	380703
A0KPP8	380703
A0KVS0	94122
A0LDB5	156889
A0LEA7	335543
A0LV15	351607
A0LZ30	411154
A0PPF2	362242
A0PQH1	362242
A0PZM3	386415
A0Q8L5	401614
A0QD34	243243
A0QI95	243243
A0QIT3	243243
A0QQU1	246196
A0QRL2	246196
A0QVT1	246196
A0RF35	412694
A0RHH0	412694
A0RP26	360106
A0RPU1	360106
A1A2Z5	367928
A1A8U2	405955

A1ADY3	405955
A1AGZ7	405955
A1AVV3	413404
A1AZX2	318586
A1B7K1	318586
A1BBC0	318586
A1BBN3	318586
A1BBP2	318586
A1BE04	290317
A1JL07	393305
A1K4F8	62928
A1KM94	410289
A1R311	290340
A1R3Q3	290340
A1R540	290340
A1RBM7	290340
A1RKF2	351745
A1RRA5	384616
A1S0N5	368408
A1S0T1	368408
A1S5T2	326297
A1S6V7	326297
A1SCP8	196162
A1SCU6	196162
A1SJ35	196162
A1SVX2	357804
A1T2A0	350058
A1T2K0	350058
A1T7Q1	350058
A1TLJ6	397945
A1TLU0	397945
A1TLZ6	397945
A1TPD7	397945
A1UDL8	189918
A1UEV2	189918
A1UG95	189918
A1V243	320388
A1VLI3	365044
A1VSV6	365044
A1VZF4	354242
A1W4S3	232721
A1WFU7	391735
A1WGR5	391735
A1WH64	391735
A1WKF7	391735
A1WLE2	391735

A1WLH4	391735
A1WNQ3	391735
A1WQB1	391735
A1WRX6	391735
A2BTN4	146891
A2BZ39	167542
A2C5B7	167555
A2C5R9	59922
A2QW51	425011
A2RJL6	416870
A2S216	412022
A2S4J0	412022
A2SIX7	420662
A2SQU8	410358
A3CN43	388919
A3D5N2	325240
A3DE17	203119
A3LZU9	4924
A3M186	400667
A3M354	400667
A3M4A6	400667
A3MCU2	320389
A3MHW8	320389
A3MT18	410359
A3N0Q9	416269
A3N4Z9	320373
A3N6S9	320373
A3NGV3	320373
A3NQP4	357348
A3NSG6	357348
A3P2F4	357348
A3PFE1	167546
A3PMS8	349101
A3PX37	164757
A3PYB2	164757
A3PZU7	164757
A3QE16	323850
A3QFI0	323850
A3U8W0	216432
A4CLV4	313596
A4CNW0	313596
A4F953	405948
A4FA73	405948
A4FCR4	405948
A4FEQ1	405948
A4FIJ8	405948

A4FK27	405948
A4FM07	405948
A4FYQ3	402880
A4IPG7	420246
A4J5V5	349161
A4JDT0	269482
A4JIC5	269482
A4JT68	269482
A4QEX8	340322
A4QH46	340322
A4SG05	290318
A4SIE4	382245
A4SR49	382245
A4SW25	312153
A4SX51	312153
A4T3J4	350054
A4TCJ3	350054
A4TDQ0	350054
A4TMN2	386656
A4UCL2	148305
A4VN86	379731
A4VU96	391295
A4W0I7	391296
A4W612	399742
A4WD58	399742
A4WJK5	340102
A4WNS1	349102
A4WU69	349102
A4X4Q6	369723
A4XDX9	279238
A4XJU0	351627
A4XSW1	399739
A4Y644	319224
A4YIF1	399549
A4YMT9	114615
A4YN22	114615
A4YP59	114615
A4YVY2	114615
A4YWD9	114615
A5CCM7	357244
A5CP49	443906
A5CSM7	443906
A5CXB6	412965
A5D2Q5	370438
A5DPD9	4929
A5EB71	288000

A5EDS6	288000
A5EKM8	288000
A5ELG2	288000
A5EQ17	288000
A5ER01	288000
A5ERA1	288000
A5EXM4	246195
A5FE82	376686
A5FQT5	216389
A5FUZ2	349163
A5FZS7	349163
A5G1C8	349163
A5G247	349163
A5GHT4	32051
A5GQ13	316278
A5I6N2	441771
A5IEB3	400673
A5IM62	390874
A5ISS7	359786
A5N263	431943
A5U6A6	419947
A5UAN5	374930
A5UG62	374931
A5ULF8	420247
A5UZA6	357808
A5V3L9	392499
A5V6L4	392499
A5VJ58	557436
A5VPI5	444178
A5VTX7	444178
A5VU88	444178
A5VZY5	351746
A5W2B9	351746
A5W6S0	351746
A5WB90	351746
A5WHC3	349106
A5WR04	336982
A6H0M8	402612
A6L420	435590
A6L5G7	435590
A6LA57	435591
A6LP58	391009
A6LUD7	290402
A6M0V8	290402
A6M2Q3	290402
A6M3G3	290402

A6Q2V4	387092
A6Q8F8	387093
A6QGU6	426430
A6SYZ5	375286
A6TB61	272620
A6TCA1	272620
A6TJU6	293826
A6TT17	293826
A6U1L6	359787
A6U799	366394
A6UFH8	366394
A6UGA5	366394
A6UGL7	366394
A6UH42	366394
A6UKQ2	366394
A6US71	406327
A6UVG7	419665
A6UY20	381754
A6V8V7	381754
A6VJM9	426368
A6VKA2	339671
A6VQQ5	339671
A6VRB6	400668
A6VX86	400668
A6VYR7	400668
A6VZF5	400668
A6W3Q5	400668
A6W828	266940
A6W9L0	266940
A6WPI6	402882
A6WVT6	439375
A6X2A0	439375
A6X302	439375
A6X446	439375
A6X488	439375
A6X799	439375
A7FG49	349747
A7FYA1	441770
A7GI19	441772
A7GMD7	315749
A7GRC9	315749
A7GYB0	360105
A7H443	360109
A7H4Q4	360109
A7H773	404589
A7HJ56	381764

A7HX36	402881
A7I0Y0	360107
A7I853	456442
A7IE29	78245
A7IIB6	78245
A7IM10	78245
A7K0D0	150340
A7MP70	290339
A7MRG1	290339
A7MY41	338187
A7NRX2	383372
A7X271	418127
A7Z1T0	326423
A7ZDQ5	360104
A7ZPS4	331111
A8A2X1	331112
A8ADA9	290338
A8AL19	290338
A8ARR9	290338
A8ET60	367737
A8EYZ4	293613
A8F1K3	416276
A8F364	416591
A8F565	416591
A8F9S2	315750
A8FLL9	407148
A8FU50	425104
A8FW16	425104
A8G7F8	93060
A8G9M3	399741
A8GCV1	399741
A8GDY3	399741
A8GHL7	399741
A8GNF1	293614
A8GS25	392021
A8GWS1	391896
A8H343	398579
A8H415	398579
A8I0D2	438753
A8I8D6	438753
A8I8N7	438753
A8I8Q6	438753
A8IA87	438753
A8ICX7	438753
A8IH60	438753
A8IQ17	438753

A8L6H1	298653
A8LKQ5	398580
A8LS87	398580
A8M766	391037
A8MDB1	397948
A8MF40	350688
A8YUT3	405566
A8Z3X3	451516
A8Z624	444179
A8ZX91	96561
A9A656	444158
A9AK76	395019
A9AQW8	395019
A9ASX4	395019
A9AZJ7	316274
A9BD97	93059
A9BHR8	403833
A9BMR7	398578
A9BN23	398578
A9BNE7	398578
A9BYA8	398578
A9BZS2	398578
A9CFV4	176299
A9CGZ4	176299
A9CHR2	176299
A9CL94	176299
A9CL97	176299
A9FHA5	448385
A9HIW2	272568
A9HQ09	272568
A9I9W9	340100
A9IDS5	340100
A9IHG0	340100
A9IRK3	382640
A9KFS0	434922
A9KHY6	357809
A9L573	399599
A9M4C8	374833
A9MA21	483179
A9MDZ3	483179
A9MEF3	483179
A9MTU8	272994
A9N2Z3	272994
A9NDT3	360115
A9NGC2	441768
A9R2G8	349746

A9VIH7	315730
A9VS41	315730
A9VYY6	419610
A9W0K2	419610
A9WE23	324602
A9WN30	288705
A9WNM3	288705
A9WP00	288705
A9WQ22	288705
A9WXU1	470137
A9WY94	470137
B0B7T0	471472
B0BBZ5	471473
B0BPI4	434271
B0BXJ1	452659
B0C142	329726
B0CKX2	470137
B0K4I3	399726
B0KAL7	340099
B0KG52	76869
B0KHK3	76869
B0KR34	76869
B0KSR4	76869
B0KST8	76869
B0RGX9	31964
B0RHT4	31964
B0RRM1	509169
B0RTW6	509169
B0T1M4	366602
B0THT2	498761
B0TK92	458817
B0TVJ5	458817
B0TWJ4	484022
B0U4J6	405440
B0U7H2	426117
B0U9P8	426117
B0UPL4	426117
B0UQL9	426117
B0UVZ8	228400
B0V931	509173
B0VB24	509173
B0VCE3	509173
B1GYN1	471821
B1HR20	444177
B1HSZ5	444177
B1I383	477974

B1IBH4	487214
B1IMW8	498213
B1JSG5	502800
B1JZR4	406425
B1K2U3	406425
B1K8Y4	406425
B1K9G9	406425
B1KAV7	406425
B1KBX4	406425
B1KCA6	406425
B1KDR2	392500
B1KJ77	392500
B1L0V1	498214
B1L3S1	374847
B1L3T8	374847
B1LBR1	126740
B1LNC8	439855
B1LT14	426355
B1LTD9	426355
B1LTL8	426355
B1M0Q9	426355
B1M5D2	426355
B1MD41	561007
B1MJI1	561007
B1MZM8	349519
B1VDC4	504474
B1VNL4	455632
B1VXI9	455632
B1VXY9	455632
B1W126	455632
B1W5U4	455632
B1W5V3	455632
B1X104	43989
B1XE11	316385
B1XES2	316385
B1XUZ8	452638
B1Y7F0	395495
B1YB29	444157
B1YFC4	262543
B1YJ39	262543
B1YNT2	398577
B1YNZ9	398577
B1YY77	398577
B1YY96	398577
B1Z2C4	398577
B1Z3J3	398577

B1ZJQ2	441620
B1ZN19	452637
B2A3B2	457570
B2AI21	164546
B2FL61	522373
B2G6M9	557433
B2GC11	334390
B2GH95	378753
B2GKH9	378753
B2HKZ1	216594
B2HNA5	216594
B2HV62	405416
B2HXN6	405416
B2I8G2	405441
B2IBL5	395963
B2IDQ5	395963
B2IPH2	516950
B2JR13	391038
B2JRB0	391038
B2JRS1	391038
B2JTH4	391038
B2K995	502801
B2KAQ5	445932
B2RMB9	431947
B2SAC4	430066
B2SBL1	430066
B2SBZ3	430066
B2SUG9	360094
B2SV49	360094
B2SXT7	398527
B2T2T6	398527
B2TAX4	398527
B2TD18	398527
B2TD34	398527
B2TFX8	398527
B2TR83	508765
B2TS68	508765
B2TXQ4	344609
B2U6L4	402626
B2U6Q6	402626
B2U9D8	402626
B2UB44	402626
B2USR7	512562
B2V104	508767
B2V5A8	508767
B2V6F1	436114

B2VE56	338565
B3CM06	570417
B3CQP2	334380
B3DTC8	205913
B3E936	398767
B3EH29	290315
B3EMS4	331678
B3GXP5	537457
B3PPG3	491916
B3PQ74	491916
B3PTP6	491916
B3PUM9	491916
B3PW83	491916
B3Q3A0	491916
B3Q3G8	491916
B3Q4S7	491916
B3Q5C0	491916
B3Q5M4	491916
B3Q7F3	395960
B3Q8S5	395960
B3QAY0	395960
B3QF89	395960
B3QJG3	395960
B3QM33	517417
B3QUT2	517418
B3R4G0	164546
B3W7E5	543734
B4E967	216591
B4ED89	216591
B4EGM8	216591
B4EHE5	216591
B4ELG9	216591
B4ETW3	529507
B4EY78	529507
B4EZP1	529507
B4FK44	4577
B4RCW4	450851
B4RZW5	314275
B4S5J5	290512
B4SE03	324925
B4SRX7	391008
B4STK9	391008
B4SVM9	423368
B4T0L6	423368
B4T864	454169
B4TD64	454169

B4TR62	439843
B4U7D7	380749
B4UHY1	447217
B5BB16	554290
B5E4D5	512566
B5EGX4	404380
B5ELM5	380394
B5ESB0	388396
B5F0L5	454166
B5F8P8	454166
B5FGX4	388396
B5FKF1	439851
B5FQH9	439851
B5R391	550537
B5R4J6	550537
B5R7I3	550538
B5RCV9	550538
B5XNR4	507522
B5XPX2	507522
B5XQV8	507522
B5Y2B8	507522
B5Y2Y6	507522
B5Y9P1	309798
B5YEB3	309799
B5Z015	444450
B5Z6I0	563041
B5ZFF8	272568
B5ZSP3	395492
B5ZVU7	395492
B5ZW64	395492
B6A0A0	395492
B6A0C9	395492
B6A0E6	395492
B6A0Z3	395492
B6A110	395492
B6A1H6	395492
B6A1Y8	395492
B6A210	395492
B6A4N5	395492
B6A4U8	395492
B6EJT2	316275
B6HI89	500485
B6I548	409438
B6IN13	414684
B6IZQ6	434923
B6J7N2	434924

B6JF33	504832
B6JGA4	504832
B6JL09	570508
B6YQ20	511995
B6YU51	523850
B7GNP0	391904
B7GNQ1	391904
B7GPM8	391904
B7GUF5	391904
B7GYH4	557600
B7H1R3	557600
B7H4G7	557600
B7HA53	405532
B7HDS2	405532
B7HLD2	405534
B7HUE3	405534
B7I214	480119
B7I2F0	480119
B7I7Z1	480119
B7IF13	484019
B7IJQ6	405531
B7ITP9	405531
B7J6C4	243159
B7JJ76	405535
B7JRU9	405535
B7KG19	65393
B7KVC7	440085
B7KZ98	440085
B7LCL6	585055
B7LKG3	585054
B7LWE4	585054
B7MDV0	585035
B7MFU2	585035
B7MHX1	585035
B7MMI3	585035
B7MYB7	585397
B7N660	585056
B7NGU5	585056
B7NQL6	585057
B7UGM8	574521
B7UIB2	574521
B7UKV6	574521
B7UWS4	557722
B7V2D6	557722
B7V6B8	557722
B7VAY6	557722

B7VIM2	575788
B8CLL5	225849
B8CMS5	225849
B8CNA7	225849
B8CWF6	373903
B8D5P3	490899
B8D702	561501
B8D8P8	563178
B8DE74	552536
B8DKU0	883
B8DWI2	442563
B8E2S8	515635
B8E724	407976
B8ESC8	395965
B8F3R4	557723
B8FAR9	439235
B8FM58	439235
B8FQY8	272564
B8GB87	326427
B8GKG7	521011
B8GN57	396588
B8H481	565050
B8H6L6	452863
B8HG81	452863
B8HGK2	452863
B8HGR8	452863
B8HGT2	452863
B8HYL7	395961
B8I0P6	394503
B8I2H0	394503
B8I6L6	394503
B8IEW0	460265
B8INC9	460265
B8INF0	460265
B8IPM2	460265
B8IQH1	460265
B8ISM1	460265
B8IW08	460265
B8J1F6	525146
B8J2R9	525146
B8JAN5	455488
B8N0F3	332952
B8ZPG7	561276
B8ZRR3	561304
B9DP23	396513
B9DS79	218495

B9E5W5	583346
B9EBZ6	458233
B9IV88	361100
B9J360	361100
B9JC65	311403
B9JDW8	311403
B9JHT9	311403
B9JJ86	311403
B9JJX3	311403
B9JL23	311403
B9JLK6	311403
B9JLL7	311403
B9JLY0	311403
B9JNI0	311403
B9JNQ3	311403
B9JUM8	311402
B9JXZ4	311402
B9K0G4	311402
B9K1C6	311402
B9K1Y2	311402
B9K4Z4	311402
B9KFX0	306263
B9KI57	320483
B9KMM3	557760
B9KTQ5	557760
B9KTV4	557760
B9KY71	309801
B9L9Q7	598659
B9LGR9	480224
B9LTM4	416348
B9LUB2	416348
B9M380	316067
B9MER6	535289
B9MRD1	521460
C0PYR9	476213
C0Q0K6	476213
C0QD66	177437
C0QT41	123214
C0R176	565034
C0R398	66084
C0RHY8	546272
C0RKH4	546272
C0RKV1	546272
C0ZF35	358681
C0ZJG4	358681
C0ZMA0	234621

C0ZRA5	234621
C0ZYK5	234621
C1A0Q0	234621
C1A409	379066
C1AFL5	561275
C1ARW0	632772
C1ASP1	632772
C1B2L1	632772
C1B363	632772
C1B513	632772
C1C6Z0	488221
C1CE08	488222
C1CK93	488223
C1CRD6	487213
C1D1Y8	546414
C1D3H3	546414
C1D433	546414
C1DD55	557598
C1DXN1	204536
C1EP21	572264
C1EXW3	572264
C1F5T7	240015
C1F658	240015
C1FL29	536232
C3K6P5	216595
C3KCC5	216595
C3KCV2	216595
C3KD56	216595
C3KL84	394
C3KMD1	394
C3KQJ0	394
C3KTL4	515621
C3L7C8	568206
C3LG19	568206
C3LPG2	579112
C3M951	394
C3MBN0	394
C3MHP5	394
C3MJG0	429572
C3MKQ5	429572
C3ML51	429572
C3MS79	427317
C3MSA8	427317
C3MTU2	427317
C3N0F2	427318
C3N1K0	427318

C3N1M9	427318
C3N8E0	439386
C3NA77	439386
C3NAA9	439386
C3NJB5	419942
C3NL95	419942
C3NLC2	419942
C3NNM5	593588
C3P5K2	592021
C3PD58	592021
C3PEF2	548476
C3PH04	548476
C3PNF5	347255
C4K288	562019
C4K791	572265
C4KB29	85643
C4KJK0	426118
C4KKN0	426118
C4KKR1	426118
C4KNL7	536230
C4KRY6	536230
C4LB09	595494
C4LDF6	595494
C4LJ67	645127
C4LL82	645127
C4PR36	580049
C4X871	484021
C4XAH9	484021
C4XBU9	484021
C4XUG2	573370
C4Z2K5	515620
C4Z902	515619
C4ZG60	515619
C4ZHF4	515619
C4ZRG0	595496
C5A8D5	626418
C5A9S9	626418
C5ADX3	626418
C5AHB8	626418
C5AIY9	626418
C5AIZ6	626418
C5ALP8	626418
C5AU72	272630
C5AY41	272630
C5B7A2	634503
C5BE76	634503

C5BQD1	377629
C5BWQ0	471853
C5BZF7	471853
C5BZP0	471853
C5C2K7	471853
C5C3V4	471853
C5C541	471853
C5C5P2	471853
C5C6J0	471853
C5C9U5	465515
C5CAU0	465515
C5CFI2	521045
C5CPI2	543728
C5CRE8	543728
C5CSU2	543728
C5CUQ8	543728
C5CZ39	543728
C5CZG3	543728
C5D0U0	543728
C5D0X0	543728
C5D1Q7	543728
C5VVH7	218494
C5ZZZ4	604354
C6A4E2	604354
C6A980	580050
C6ACI7	634504
C6AFC2	555970
C6ARM9	395491
C6ATG1	395491
C6ATS4	395491
C6AVE9	395491
C6AY49	395491
C6B4B0	395491
C6B4Q5	395491
C6B5M5	395491
C6B5P6	395491
C6BA02	395491
C6BB07	395491
C6BCF4	428406
C6BDC7	428406
C6BG76	428406
C6C2X7	579405
C6C501	579405
C6C663	579405
C6C7K0	579405
C6CA25	579405

C6CDL4	579405
C6CK12	561229
C6CQL0	561229
C6D2D9	324057
C6D630	324057
C6DBS0	561230
C6DEB8	561230
C6DGJ2	561230
C6DLF4	478434
C6E9Q9	443144
C6GS15	568813
C6GTM8	568814
C6SQR7	511691
C6UB86	413997
C6UQM8	544404
C6V639	434131
C6VJ20	644042
C6VR51	644042
C6VS77	471854
C6VYY4	471854
C6W118	471854
C6WDI3	446462
C6WN40	446462
C6X0P9	531844
C6XDF4	582744
C6XHK6	537021
C6XJY7	582402
C6XTJ4	485917
C6Y0I5	485917
C6Y1M1	485917
C6Y470	485917
C7BGW6	553480
C7BK04	553480
C7BYI2	592205
C7CBF3	661410
C7CC66	661410
C7JH79	634452
C7LAV8	568815
C7LH83	568815
C7LHP8	568815
C7LKC0	595499
C7LN00	525897
C7LXZ2	525909
C7M875	521097
C7M9Z3	446465
C7MB37	446465

C7MBC1	446465
C7MN92	469378
C7MNH1	469378
C7MZF2	471857
C7N471	471855
C7NAR3	523794
C7NV35	519442
C7NV78	519442
C7NWM0	485914
C7P2G1	485914
C7P6S9	573064
C7PAP9	485918
C7PMD2	485918
C7QF16	479433
C7QFA5	479433
C7QI35	479433
C7R3F2	471856
C7R3Y7	471856
C7RCP7	523791
C7TGH3	568704
C8U801	585395
C8VJZ9	227321
C8W4M0	485916
C8W7B8	521095
C8W8B5	521095
C8WBL5	622759
C8WDY8	622759
C8WHK3	479437
C8WJI4	479437
C8WRJ5	521098
C8WWK4	521098
C8X411	485915
C8X840	479431
C8XJF3	479431
C9RKP4	59374
C9XQ20	645462
C9XRH2	645462
C9XRH4	645462
C9XST0	645462
C9XVI1	413502
C9XXL1	413502
C9Y5E4	413502
C9YQH0	645463
C9YR65	645463
C9YR67	645463
C9YSC0	645463

C9YYA9	680198
C9YYD5	680198
C9Z234	680198
C9Z6M9	680198
C9Z7W8	680198
C9ZAR0	680198
D0B673	224914
D0FNJ5	79967
D0FQB5	79967
D0FQH7	79967
D0IX32	688245
D0J2D8	688245
D0J602	688245
D0J9L9	600809
D0JAP4	331104
D0K4X0	681288
D0KBI2	561231
D0KCD2	561231
D0KIX6	561231
D0KLW1	561231
D0KLW5	561231
D0KYK6	555778
D0LBM4	526226
D0LJC8	502025
D0M0B9	150340
D0MEU6	518766
D0MJ86	518766
D0R4B4	633699
D0ZEY7	498217
D1AAD5	471852
D1ANM6	526218
D1ASG1	574556
D1AYT8	519441
D1B1W3	525898
D1BJ50	446469
D1BMK4	479436
D1C071	446471
D1C5L0	479434
D1C8S2	479434
D1CAQ9	479434
D1YV26	304371
D1YWA9	304371
D2AV88	479432
D2AXY0	479432
D2AYJ0	479432
D2B182	479432

D2B2X1	479432
D2BFP3	479432
D2BI24	311424
D2BKQ5	684738
D2BQY9	684738
D2BUR9	590409
D2BVR9	590409
D2BWH3	590409
D2BY70	590409
D2BZ30	590409
D2BZN5	590409
D2C301	590409
D2C3R9	590168
D2NNS6	680646
D2P260	653938
D2P4Z6	637381
D2PE01	425944
D2PFU0	425944
D2PFW3	425944
D2PLV8	479435
D2PWS2	479435
D2PYI2	479435
D2Q1Q5	479435
D2Q5X9	401473
D2QG66	504472
D2QGP8	504472
D2QN45	504472
D2QUR4	504472
D2QW61	530564
D2R485	530564
D2R9F4	530564
D2RDF8	572546
D2RKF2	591001
D2RMZ3	591001
D2RW27	543526
D2RX78	543526
D2RZP5	543526
D2S0Y4	543526
D2S1K0	543526
D2S1M1	543526
D2S4C0	526225
D2SD07	526225
D2SDU6	526225
D2TIF2	637910
D2TK04	637910
D2TTB3	637910

D2TU27	637910
D2TU80	637910
D2TUM2	637910
D2U8E6	380358
D2UEC0	380358
D3DYX1	634498
D3E6N4	481743
D3EEF0	481743
D3EIE3	481743
D3EJZ4	481743
D3EL06	481743
D3EMT9	713887
D3EZY8	469383
D3FDP9	469383
D3FE39	469383
D3H934	365659
D3HDJ4	637909
D3HGD9	637909
D3NUF4	137722
D3P161	137722
D3P2I0	137722
D3P6D4	137722
D3P7S9	137722
D3PRL4	504728
D3Q111	446470
D3Q1E3	446470
D3Q2I9	446470
D3QCX6	698737
D3QM57	701177
D3R8S4	640131
D3RCU8	640131
D3REW3	640131
D3RFW7	640131
D3RKF1	640131
D3RY72	589924
D3S6L2	644281
D3SJ26	633145
D3SSE1	547559
D3SZP0	547559
D3T7X5	580331
D3UJI0	679897
D3V7E2	406818
D3VFM1	406817
D3VH41	406817
D3VI77	406817
D4G7V5	515618

D4GCS7	706191
D4GEP2	706191
D4GIJ4	706191
D4GIW6	706191
D4GK06	706191
D4GP99	309800
D4GPZ5	309800
D4GW28	309800
D4H4C2	522772
D4HAE1	553199
D4HCG6	553199
D4HY75	665029
D4HYD7	665029
D4I8B5	716540
D4I8H1	716540
D4Z688	452662
D4ZJ32	637905
D4ZKI7	637905
D5AM74	272942
D5B1C1	637386
D5BIF1	655815
D5BK41	655815
D5BNX6	488538
D5BR88	488538
D5BWV0	472759
D5C6Z8	716541
D5C8V4	716541
D5CBB0	716541
D5CF43	716541
D5D8T2	641892
D5DDJ9	592022
D5DKV8	592022
D5DQ48	545693
D5E1Y2	545693
D5EC21	547558
D5EH49	572547
D5EJ55	583355
D5ESD5	264731
D5H2T9	748671
D5SQS5	521674
D5SVD4	521674
D5T5I1	762051
D5T7I7	423212
D5TNQ9	714359
D5TUT4	714359
D5UAX2	526224

D5UD90	446466
D5UMA0	521096
D5UPJ0	521096
D5V0G9	572480
D5V3A6	572480
D5V3H2	572480
D5VA07	749219
D5VKW2	509190
D5WE15	640511
D5WGB0	640511
D5WI55	640511
D5WIR3	640511
D5WLV7	640511
D5WPM0	562970
D5WPM5	562970
D5WY73	75379
D5XF37	635013
D6XU19	439292
D6Y7Y3	469371
D6YVH1	716544
D6Z916	640132
D6ZLC6	525381
D6ZSW1	759350
D6ZWJ9	759350
D6ZY10	759350
D6ZZ88	639283
D7A0U5	639283
D7A0Y5	639283
D7A101	639283
D7A5B9	639283
D7AAC5	639283
D7AMY7	583358
D7AYA7	446468
D7B664	446468
D7BIJ2	526227
D7BP32	644284
D7CM70	643648
D7CQM5	649638
D7D631	691437
D7DRN6	456320
D7E164	551115
D7EB04	644295
D7FES9	693745
D7GF03	754252
O05969	782
O25657	210

O26892	187420
O29352	2234
O58577	53953
O67216	63363
O84366	813
O86841	1902
P19808	1718
P43797	727
P49423	1219
P58207	381
P63943	217992
P63945	1773
P63946	1765
P63947	158878
P63948	158879
Q01R27	234267
Q024Z1	234267
Q02D66	234267
Q02T10	208963
Q02UL2	208963
Q02XU7	272622
Q03CW3	321967
Q03HT2	278197
Q03K16	322159
Q03YE2	203120
Q04FS1	203123
Q04KR9	373153
Q04U80	355277
Q053P3	355276
Q058B1	372461
Q05FN5	387662
Q07KS7	316055
Q07M65	316055
Q082V3	318167
Q0APB2	394221
Q0AXH6	335541
Q0B2H2	339670
Q0B5F4	339670
Q0B5Y2	339670
Q0BBC4	339670
Q0BG20	339670
Q0BSV9	391165
Q0BYG4	228405
Q0HHQ6	60480
Q0HU08	60481
Q0I260	205914

Q0IE16	64471
Q0K061	381666
Q0K2U1	381666
Q0K4R0	381666
Q0PB32	197
Q0RDU6	326424
Q0RXH3	101510
Q0S0T5	101510
Q0S1R7	101510
Q0S5X0	101510
Q0SCP1	101510
Q0SCQ5	101510
Q0SJ38	101510
Q0SRS5	289380
Q0T239	373384
Q0TEZ8	362663
Q0TK08	362663
Q0TP55	195103
Q0V0Q5	13684
Q0W314	351160
Q0W675	351160
Q118P2	203124
Q11AT3	266779
Q11BU9	266779
Q11DL4	266779
Q11F73	266779
Q11JT7	266779
Q11VI2	269798
Q125T0	296591
Q128F8	296591
Q129P0	296591
Q12BF6	296591
Q12BH0	296591
Q12NF7	318161
Q12ZG2	259564
Q132Q2	316057
Q135A1	316057
Q136X0	316057
Q13DE3	316057
Q13J43	266265
Q13LF8	266265
Q13MZ2	266265
Q13PW0	266265
Q146P4	266265
Q15SZ4	342610
Q16BK4	375451

Q16C65	375451
Q17WU7	382638
Q17ZW8	272563
Q17ZX0	272563
Q181M9	272563
Q184J1	272563
Q18HQ1	362976
Q18K17	362976
Q1ATU0	266117
Q1AZ77	266117
Q1AZV1	266117
Q1B8R2	164756
Q1BA60	164756
Q1BBC9	164756
Q1BIC8	331271
Q1BJT5	331271
Q1BKC4	331271
Q1BKP7	331271
Q1BMP2	331271
Q1BT22	331271
Q1BV84	331271
Q1C5Q4	360102
Q1CK27	377628
Q1CU72	357544
Q1CXM5	246197
Q1CZ39	246197
Q1CZV8	246197
Q1GG89	292414
Q1GME4	292414
Q1GSC8	117207
Q1I6I8	384676
Q1IAX8	384676
Q1IEM7	384676
Q1INQ6	204669
Q1LCR5	266264
Q1LD20	266264
Q1LPH2	266264
Q1LQM4	266264
Q1M341	216596
Q1M3T7	216596
Q1M4D2	216596
Q1M599	216596
Q1M709	216596
Q1M7N5	216596
Q1M8Q7	216596
Q1MAR8	216596

Q1MAX6	216596
Q1MD94	216596
Q1ME92	216596
Q1MIA4	216596
Q1MJ63	216596
Q1QBF5	335284
Q1QEG1	335284
Q1QL43	323097
Q1QU05	290398
Q1R5G0	364106
Q1R8P9	364106
Q1REP2	364106
Q1RIJ3	336407
Q20Y81	316056
Q211L1	316056
Q214K7	316056
Q216I5	316056
Q21A63	316056
Q21WN2	338969
Q21XY7	338969
Q21ZN5	338969
Q221U8	338969
Q24UK5	138119
Q255G0	264202
Q28JT1	290400
Q28SB0	290400
Q28SE2	290400
Q2FNR0	323259
Q2G8D6	279238
Q2GCK3	222891
Q2GG09	205920
Q2IGX5	290397
Q2IRF2	316058
Q2IT09	316058
Q2IWV5	316058
Q2IXK2	316058
Q2J746	106370
Q2JQ67	321332
Q2JWL7	321327
Q2JYP8	347834
Q2JZ58	347834
Q2JZJ9	347834
Q2K187	347834
Q2K1W0	347834
Q2K3G4	347834
Q2K5H1	347834

Q2K6H5	347834
Q2KAF2	347834
Q2KCL4	347834
Q2KXD5	360910
Q2L1M0	360910
Q2LTA1	56780
Q2N9J7	314225
Q2NHW2	339860
Q2NS74	343509
Q2P143	342109
Q2P1P8	342109
Q2RJK7	264732
Q2RKS9	264732
Q2RP09	269796
Q2RT80	269796
Q2S2M2	309807
Q2S3M1	309807
Q2SHE8	349521
Q2T0E0	271848
Q2T1R8	271848
Q2T3J3	271848
Q2UIX1	5062
Q2W478	342108
Q2W4Y2	342108
Q2YJT0	359391
Q2YK64	359391
Q2YMY1	359391
Q2YXX4	273036
Q30R77	326298
Q310Q2	207559
Q317Y9	74546
Q31FC9	317025
Q31Y13	300268
Q32D87	300267
Q390E9	269483
Q394V7	269483
Q396X5	269483
Q39BG6	269483
Q39CC4	269483
Q39EX1	269483
Q39LG7	269483
Q39LK1	269483
Q39Z67	269799
Q3A1U7	338963
Q3ACI0	246194
Q3ANI5	110662

Q3APU0	340177
Q3B0T8	316279
Q3B2G4	319225
Q3BRY8	316273
Q3BUP1	316273
Q3IJ34	326442
Q3ISQ0	348780
Q3IVH2	272943
Q3IWM4	272943
Q3IWS2	272943
Q3IZG9	272943
Q3J869	323261
Q3JHB0	320372
Q3JV31	320372
Q3JWT2	320372
Q3KGQ6	205922
Q3KLZ5	315277
Q3SRA2	323098
Q3SU94	323098
Q3YSK1	269484
Q3YZ74	300269
Q3Z7V3	243164
Q3ZXV3	255470
Q46DC4	269797
Q46ID7	59920
Q47HS9	159087
Q47RT8	269800
Q485S4	167879
Q48L68	264730
Q492F5	291272
Q49XJ7	342451
Q4FLS1	198252
Q4FP92	198252
Q4FVD6	259536
Q4JC96	2285
Q4JV64	306537
Q4KBF7	220664
Q4KGP8	220664
Q4KGU1	220664
Q4KGV9	220664
Q4L6A0	279808
Q4QNT3	281310
Q4ULR2	42862
Q4UTT0	314565
Q4UW06	314565
Q4W8W3	5085

Q4ZW06	205918
Q4ZW75	205918
Q55513	1148
Q57695	2190
Q577J6	235
Q57E96	235
Q57IU4	28901
Q57LM2	28901
Q5E3I3	312309
Q5F849	242231
Q5FE59	254945
Q5FHF1	302409
Q5FKQ9	1579
Q5FUW9	442
Q5GT73	292805
Q5HBR0	254945
Q5HG25	93062
Q5HPE7	176279
Q5HUY6	195099
Q5HVY5	195099
Q5JGK8	311400
Q5KA86	5207
Q5KYJ0	1462
Q5L5G7	83555
Q5L8P3	272559
Q5LCH9	272559
Q5LKW2	89184
Q5LMK7	89184
Q5LPG6	89184
Q5LSR5	89184
Q5LZ67	299768
Q5M3S2	264199
Q5NLD3	542
Q5NPL6	542
Q5P838	76114
Q5PB64	234826
Q5SJQ1	300852
Q5V3H1	2238
Q5V5D4	2238
Q5WBX0	66692
Q5WDL1	66692
Q5WFV6	66692
Q5WGE0	66692
Q5WJ04	66692
Q5WLJ0	66692
Q5WUD4	297245

Q5X183	297246
Q5X2X5	297246
Q5YSX4	37329
Q5YWC2	37329
Q5ZT51	272624
Q62BA2	13373
Q62IC0	13373
Q636M7	288681
Q63AC1	288681
Q63NG8	28450
Q63WF2	28450
Q63Y42	28450
Q64TM6	817
Q65VX1	221988
Q65VY8	221988
Q65WI6	221988
Q668F7	633
Q6A699	1747
Q6AA18	1747
Q6AFF4	59736
Q6AR63	84980
Q6CYT0	29471
Q6D0N5	29471
Q6D7R3	29471
Q6D8H2	29471
Q6F6U2	62977
Q6G091	803
Q6G468	38323
Q6G9G6	282459
Q6GH13	282458
Q6KZI8	263820
Q6LN85	74109
Q6LZP8	39152
Q6MDD9	264201
Q6MRM9	959
Q6N4Z3	1076
Q6N5H6	1076
Q6N6D1	1076
Q6N9H8	1076
Q6NBD0	1076
Q6NGP4	1717
Q6NJA1	1717
Q719I7	235
Q71ZN5	265669
Q72K27	262724
Q732S4	222523
Q736N9	222523
Q73H02	163164
Q73VZ7	1770
Q73XB7	1770
Q741N1	1770
Q74GT6	35554
Q7CU96	176299
Q7D0E8	176299

Q7D313	176299
Q7D3Z9	176299
Q7M7L6	844
Q7MC05	196600
Q7MIL2	196600
Q7MTB9	837
Q7N3H1	141679
Q7N4S4	141679
Q7N6L9	141679
Q7NLV1	33072
Q7NS49	536
Q7NU78	536
Q7NWE2	536
Q7UA33	84588
Q7UGE1	265606
Q7UMJ1	265606
Q7USB5	265606
Q7UVB1	265606
Q7UXD2	265606
Q7UZK9	59919
Q7V990	74547
Q7VFP9	32025
Q7VM87	730
Q7VRT1	203907
Q7VS12	520
Q7VT65	520
Q7W1K7	519
Q7W287	519
Q7WEB4	518
Q7WND2	518
Q7WPJ8	518
Q7WR54	518
Q819Z9	226900
Q81CD9	226900
Q81PH3	1392
Q81WN7	1392
Q822H0	83557
Q829P3	33903
Q829R6	33903
Q82BU7	33903
Q82K87	33903
Q82MC9	33903
Q836D1	1351
Q83CA6	777
Q87AT3	183190
Q87MI2	670
Q87Q15	670
Q88JL0	160488
Q88L99	160488
Q88NF4	160488
Q88U46	1590
Q88VD7	1590
Q891S3	1513
Q89AY0	224915

Q89C29	375
Q89C45	375
Q89DQ7	375
Q89E15	375
Q89FB7	375
Q89JF2	375
Q89LX0	375
Q89NF6	375
Q89Q23	375
Q89Q28	375
Q8A3Z0	818
Q8A9C0	818
Q8CP96	176280
Q8D2M3	36870
Q8DBB0	672
Q8DPZ9	171101
Q8DUE5	1309
Q8EFT7	70863
Q8EMH5	182710
Q8EMJ7	182710
Q8EQJ1	182710
Q8FJW9	217992
Q8FWP0	29461
Q8FX50	29461
Q8G1R0	29461
Q8G527	216816
Q8KC06	1097
Q8NMD2	1718
Q8NWS5	196620
Q8PXL7	2209
Q8RBI5	119072
Q8RQM8	152794
Q8THP1	2214
Q8TUZ4	2320
Q8U319	2261
Q8UGL3	176299
Q8XJ56	1502
Q8XXK2	305
Q8Y099	305
Q8Y1M5	305
Q8Y766	1639
Q8YBN7	29459
Q8YG60	29459
Q8ZCD0	632
Q8ZU75	13773
Q92BS0	1642
Q92I25	781
Q92LF2	382
Q92R55	382
Q92U05	382
Q92WT0	382
Q92X22	382
Q92ZS6	382
Q93RY0	1902

Q96YD3	111955
Q979B0	273116
Q97D80	1488
Q97GI9	1488
Q97R25	1313
Q97UF0	2287
Q97UI2	2287
Q97WF2	2287
Q988B6	381
Q989L2	381
Q989T0	381
Q98F18	381
Q98LI3	381
Q9A900	155892
Q9AKE4	785
Q9CBW4	1769
Q9CF61	1360
Q9CLZ7	747
Q9EZ12	93061
Q9HJ17	2303
Q9HWJ3	287
Q9I490	287
Q9I6R5	287
Q9KQ47	666
Q9PER5	2371
Q9PK33	83560
Q9PPB4	197
Q9RH76	375
Q9UZ94	29292
Q9X1K9	2336
Q9X9W0	1902
Q9X9Y3	1902
Q9YD89	56636
Q9Z6K9	83558
Q9ZM13	85963
A0B400	331272
A0KDT3	331272
A0KDV3	331272
A0NJU4	379360
A0NM70	384765
A0NMA5	384765
A0NP47	384765
A0NXQ6	384765
A0P144	384765
A0XX40	156578
A0YE32	247633
A0YKN3	313612
A0Z8V0	247639
A1BB33	318586
A1ELC2	345076
A1F3Z4	412614
A1HSD2	401526
A1ZPG2	313606
A1ZYV5	313606

A2P5L0	412966
A2PS52	412883
A2TC91	13690
A2TRQ1	313590
A2TSZ4	313590
A2U4B1	313598
A2V5G2	399804
A2VL89	348776
A2VUX9	350702
A2W1Q2	350702
A2W277	350702
A2W2A4	350702
A2W4I7	350702
A2W520	350702
A2W5N6	350702
A2W702	350701
A2WAR3	350701
A2WEE9	350701
A2WH12	350701
A2WH36	350701
A2WJ95	350701
A3ENF8	345075
A3EQW5	419542
A3GPI5	417399
A3H0F0	417400
A3HVZ6	388413
A3I2H3	388413
A3I9D5	388400
A3ICC5	388400
A3J098	391598
A3J824	270374
A3JGP4	270374
A3JI71	270374
A3JRQ3	388401
A3K1H8	388399
A3K3V8	388399
A3K8S9	388399
A3KHZ7	278992
A3KT92	350704
A3L0K0	350704
A3L5Z2	350703
A3LFT1	350703
A3LH34	350703
A3PP87	349101
A3PPD5	349101
A3RTG3	342110
A3RW68	342110
A3RXR3	342110
A3RYI1	342110
A3S025	342110
A3SG05	52598
A3SIA3	89187
A3SJ32	89187
A3SLP3	89187

A3SQ55	89187
A3SQV4	89187
A3SV61	314267
A3TMG3	313589
A3TT45	252305
A3TWJ5	252305
A3UC80	314254
A3UUQ4	314291
A3V206	314232
A3V529	314232
A3VCB4	314271
A3VUG8	314260
A3VYD1	314264
A3W5D0	314264
A3W8S6	314264
A3WBB4	237727
A3WVY4	314253
A3XD47	314262
A3XEW9	314262
A3XKJ8	398720
A3XZB3	314290
A3Y578	314277
A3Y8A4	314277
A3YE11	314277
A3YFI5	314277
A3YIX7	360111
A3YM52	360111
A3YSL6	360108
A3YWM7	69042
A3Z412	221360
A3ZE51	360112
A3ZJ06	360110
A3ZKB8	360110
A3ZUS3	314230
A3ZV89	314230
A3ZZV5	314230
A4ACJ4	314285
A4AHI3	312284
A4AHS6	312284
A4ARE1	313603
A4AV15	313603
A4BHX5	314283
A4BPN1	314278
A4BRP0	314278
A4C204	313594
A4C7V1	87626
A4CCE4	87626
A4CYI2	59931
A4E7M5	411903
A4ECP0	411903
A4EHQ9	391593
A4EMG3	391593
A4ENS4	388739
A4EWP5	388739

A4JKL4	269482
A4KK27	395095
A4LK20	425067
A4LVD2	425067
A4MG82	425067
A4MXJ0	374927
A4N4L0	375432
A4N8J6	375177
A4NG05	374928
A4NLT4	374932
A4NU55	374933
A4NVH7	375063
A4U1M6	55518
A4U2H4	55518
A4ZH45	326425
A5DJY9	4929
A5F699	345073
A5J3T9	334802
A5J7P2	334802
A5KF36	398000
A5KI83	398000
A5KPW2	411460
A5L605	391574
A5LD17	406556
A5LKR5	406557
A5LW41	406558
A5M3X4	406559
A5MAU9	406560
A5MEL3	406561
A5MQJ4	406562
A5MTN1	406563
A5P744	161528
A5TMI5	370895
A5XKV7	334803
A5XTM6	334803
A5ZAT0	411463
A5ZCG9	411901
A5ZN98	411459
A6A2I8	417398
A6AB47	417397
A6ARV9	410291
A6AYY2	419109
A6B040	419109
A6BDM6	411462
A6BHQ5	411462
A6BN72	412420
A6C7C0	344747
A6CKS9	161544
A6CZS2	391591
A6DCI4	391592
A6E1D4	391613
A6E2Y6	391613
A6E795	391596
A6EST7	50743

A6FEJ1	58051
A6FT56	351016
A6FYB9	391625
A6GJN0	391625
A6GV02	391597
A6XY86	404974
A6Y3V2	345074
A7A7E6	411481
A7AES8	411477
A7JF57	430558
A7JP23	442346
A7JTU4	272629
A7K0J5	150340
A7LXQ1	411476
A7V359	411479
A7VCL5	411489
A8E8R7	360118
A8EIR4	360118
A8H391	398579
A8KJN4	331978
A8KPJ4	331978
A8KTN1	331978
A8PKT2	59196
A8RF55	428127
A8RLY9	411902
A8S1Q9	411902
A8S9N9	411485
A8SFA5	411485
A8STN4	411474
A8T4X4	314289
A8TK78	331869
A8TLV0	331869
A8TLW4	331869
A8U3A2	331869
A8UMX2	391603
A8UTD8	392423
A9AKX4	395019
A9CW65	411684
A9D0X1	411684
A9DBF7	411684
A9DSY7	391624
A9DTL9	391624
A9DWX7	391624
A9E4Z0	391587
A9ECC1	391587
A9EEV2	391624
A9EFY5	391624
A9EIR3	383629
A9ER44	383629
A9FWL0	391619
A9GAY4	391619
A9H3K8	391595
A9K2B6	412021
A9K345	412021

A9Z339	373665
A9ZIP8	360117
B0A1A7	404214
B0A7C6	445973
B0ACU3	445973
B0AK64	486624
B0AWN2	486624
B0G383	411461
B0GD00	404218
B0GYZ7	404217
B0H4L3	404216
B0HHL2	404215
B0HY28	360099
B0M935	411490
B0MFE5	411490
B0MPQ8	428128
B0NJJ8	411468
B0NPT1	449673
B0NXE4	411484
B0PDA9	445972
B0PYH7	486619
B0Q499	486619
B0QC30	486621
B0QN01	486621
B0QU22	456298
B0UFW5	426117
B0YE04	451804
B1BDZ1	445337
B1BIV8	445334
B1BYB2	451755
B1EJ32	502347
B1ETN6	486623
B1EZ83	486623
B1F7T9	396596
B1FAB9	396596
B1FAF0	396596
B1FBJ7	396596
B1FCA2	396596
B1FK30	396596
B1FQE8	396596
B1FVL2	396598
B1G033	396598
B1G5U5	396598
B1G6J3	396598
B1G9W2	396598
B1G9X7	396598
B1GE13	486620
B1GKS7	486620
B1HBZ0	320374
B1HEW5	320374
B1HGJ5	320374
B1K2R5	406425
B1KAT7	406425
B1QA87	445335

B1QLX9	445336
B1QSR0	447214
B1QWQ3	447214
B1QXJ9	447214
B1R337	451754
B1RHP0	451756
B1RL46	451757
B1RZX9	453362
B1S575	473819
B1SEP8	471872
B1SXE7	396597
B1SXW5	396597
B1SZ87	396597
B1T3Y4	396597
B1T569	396597
B1T8F9	396597
B1TAF4	396597
B1UJC2	486622
B1UR08	486622
B1V7F8	488537
B1X4L0	39717
B1XVT2	452638
B1Z2G0	398577
B2DL35	453361
B2DMZ5	453363
B2DV69	453364
B2E197	453365
B2E5X9	453366
B2EAC1	486409
B2H202	331109
B2H3J4	331109
B2H5H9	331109
B2JT32	391038
B2MUB4	1927
B2N3D7	344610
B2NNP0	444451
B2P3S5	444452
B2PE96	444453
B2PYK0	471874
B2Q0U5	471874
B2UCC4	402626
B3A8X0	478004
B3AMW7	478005
B3B0F4	478006
B3BIW2	478008
B3BV53	478007
B3C8T9	471870
B3G1D0	287
B3HF54	340184
B3HUI0	340197
B3HXE7	340197
B3I872	340185
B3II87	340186
B3J111	405536

B3JAA6	405536
B3JJD8	470145
B3Q4L3	491916
B3WKD4	344601
B3WYA3	358708
B3XDN3	358709
B3XRK5	349123
B3YCM1	439842
B3YEV5	439842
B3YNY5	405917
B3YTX0	405917
B3Z5U0	451707
B3ZKE9	451709
B3ZMV9	451709
B4A5P6	454168
B4A9F8	454168
B4AIZ8	536229
B4ARI7	545422
B4AWT9	497965
B4BM42	495036
B4CW35	497964
B4CZA6	497964
B4D5G4	497964
B4ELJ8	216591
B4EPL9	216591
B4FLW2	4577
B4RZW6	314275
B4V207	465541
B4VGT1	465541
B4W7G2	391600
B4W8Y7	391600
B4WRF7	91464
B5BX60	439846
B5C2M7	439846
B5CGS2	454165
B5CTH5	471875
B5CVS8	484018
B5GIM4	465543
B5GYM1	443255
B5H1S3	443255
B5HEV1	457429
B5HF22	457429
B5HH16	457429
B5HK97	457429
B5HR40	463191
B5HRE3	463191
B5HXJ9	463191
B5I6V3	463191
B5IJE1	180281
B5ITB9	391623
B5J1L6	391626
B5J651	391626
B5JGR0	382464
B5JRZ9	391615

B5JTB9	391615
B5K0E3	391616
B5KE45	391616
B5MHP0	439847
B5MI96	439847
B5MWS9	440534
B5MZE3	440534
B5NFU1	454167
B5NRG3	454231
B5NSY7	454231
B5P1Z5	454164
B5P592	454164
B5PA51	465518
B5PR36	465516
B5PTH2	465516
B5Q1P1	465517
B5Q7W2	465517
B5QIT6	478547
B5QP82	486408
B5RVX5	305
B5RZJ9	305
B5S3R7	305
B5S5X2	305
B5S916	305
B5SDP9	305
B5SH52	564066
B5SHD8	564066
B5SHS7	564066
B5SM69	564066
B5UUN0	405533
B5UWV6	405533
B5V0N4	451708
B5VCI4	451708
B5WDG7	516466
B5WE31	516466
B5WKB8	516466
B5WN74	516466
B5WQ23	516466
B5WS27	516466
B6AKG1	419541
B6AV07	314270
B6AY12	314270
B6B1V6	314270
B6BCI7	439496
B6BND2	439483
B6BPE7	439493
B6BS33	439493
B6C693	473788
B6FRG4	500632
B6FY66	500633
B6JA54	504832
B6KFM9	508771
B6QX39	439495
B6R7G0	439495

B6VZH1	483217
B6W4A4	483217
B6WR23	411464
B6XF15	520999
B6XVB1	566552
B6Y6C0	569881
B6YY53	439495
B6ZWZ8	502346
B7A769	498848
B7AJW3	483216
B7ALN9	483216
B7B920	537006
B7CIA2	557724
B7CS30	557724
B7D091	557724
B7DM57	543302
B7DS76	543302
B7P667	6945
B7QQY7	439497
B7QYR3	439497
B7R6L6	391606
B7RE49	443254
B7RLX2	391589
B7RLX3	391589
B7RMR3	391589
B7RNB7	391589
B7RTH2	247634
B7WVE3	399795
B7X5U7	399795
B8IIG9	460265
B8IIJ4	460265
B8ISI9	460265
B8ISK7	460265
B8ITG9	460265
B8IX71	460265
B8IY15	460265
B8K4H7	391586
B8KE08	391586
B8KF22	566466
B8KQB5	565045
B8L258	391601
B8LB00	391601
B8M4J1	441959
B9AEG4	483214
B9B0R3	513051
B9B353	513051
B9B5Z7	513051
B9BLF1	513052
B9BLW4	513052
B9BP73	513052
B9BUB6	513052
B9BVX7	513052
B9C5F6	513053
B9C5X0	513053

B9C9G3	513053
B9CCW8	513053
B9CEK7	513053
B9CKR8	553184
B9CP57	553184
B9CRV3	553212
B9D2Z5	553218
B9GBB2	39947
B9NJB5	3694
B9NJT3	3694
B9NLW1	467661
B9NVW9	467661
B9NXJ3	467661
B9NY47	467661
B9NYZ6	93058
B9PPF1	5811
B9QJ99	432359
B9QUZ4	244592
B9QYU8	244592
B9R3Q0	244592
B9R4E2	244592
B9WTD3	286604
B9XSF8	320771
B9XT90	544405
B9XY66	544406
B9Z075	279714
C0AQE1	471881
C0AUB0	471881
C0B3H1	471881
C0BAM6	470146
C0BHS2	487796
C0BN46	487797
C0BRF6	547043
C0BRW0	547043
C0BYQ8	553973
C0BYZ0	553973
C0C033	553973
C0CGR1	476272
C0CJV0	476272
C0CV47	518636
C0D119	518636
C0D2X4	518636
C0D4B5	518636
C0DTL3	546274
C0E2J0	566549
C0EF08	537013
C0EFH2	537013
C0EZF7	411469
C0F8S4	77037
C0FNL6	622312
C0G532	595497
C0G9H7	595497
C0G9Y2	595497
C0GIY1	555088

C0N9M4	637616
C0VLX6	525244
C0VUB8	548477
C0VVI6	548477
C0W1Z6	525245
C0W2G3	525246
C0WGE4	525260
C0WKP2	525260
C0WYW7	525325
C0X4N2	491074
C0XSG0	525263
C0Y028	595498
C0Y073	595498
C0YCU3	595498
C0YGJ3	595498
C0YKU0	525257
C0YSG8	525257
C0Z0S5	585517
C1HG39	469598
C1HNX4	469598
C1HRE8	469598
C1HS33	469598
C1I601	457396
C1M7V6	469595
C1MC25	469595
C1MFW1	469595
C1V4A0	469382
C2BNZ8	525264
C2C225	525367
C2CB94	592313
C2CNY6	525268
C2DDP5	525278
C2DLG5	525281
C2DV56	525281
C2E692	525330
C2E8G3	525362
C2EML3	525365
C2ESS4	525366
C2EWS6	491077
C2F3S4	548485
C2FAU8	525337
C2FHV1	525338
C2FK34	525338
C2G407	525372
C2GC85	548473
C2GIA0	548478
C2GJT4	548478
C2GQH5	525341
C2GUU8	548480
C2H5V1	525271
C2HNU7	525306
C2HUK8	593585
C2I6X3	593586
C2IIN0	593589

C2IR98	593590
C2JG78	593587
C2JJ22	491075
C2K0T9	525361
C2KC71	491076
C2KKV3	586220
C2KQM6	585199
C2KVM1	585501
C2LEQ3	525369
C2LG14	525369
C2LH50	525369
C2LTG1	596322
C2M0I2	629742
C2M8Q9	553178
C2MLU8	526973
C2MPC9	526973
C2N218	526980
C2N4U0	526980
C2NIT2	526970
C2NLK2	526970
C2NZP6	526967
C2P2E3	526967
C2PG44	526971
C2PIW4	526971
C2PW69	526972
C2PZE9	526972
C2QCX1	526968
C2QFL3	526968
C2QU71	526977
C2QWX0	526977
C2R9A1	526969
C2RBZ3	526969
C2RP57	526974
C2RRV2	526974
C2S4I3	526975
C2S7J6	526975
C2SLC1	526976
C2SNU8	526976
C2T260	526978
C2T4Q2	526978
C2TH89	526979
C2TKB9	526979
C2TYP0	526981
C2U135	526981
C2UEZ9	526982
C2UHR6	526982
C2UWH6	526983
C2UZ38	526983
C2VD14	526984
C2VFL9	526984
C2VUW6	526985
C2VXL0	526985
C2WAU3	526986
C2WDH4	526986

C2WFB7	526986
C2WFN4	526986
C2WNL5	526987
C2WRH7	526987
C2XCP4	526989
C2XFF7	526989
C2XV81	526990
C2XXQ9	526990
C2YBK8	526991
C2YE49	526991
C2YSQ1	526992
C2YV81	526992
C2Z910	526993
C2ZBI8	526993
C2ZQQ4	526994
C2ZT21	526994
C3A711	526997
C3A9I8	526997
C3AM36	526998
C3AVI7	526998
C3B4C2	526999
C3BDU2	526999
C3BL56	527000
C3BNI6	527000
C3C3H8	527024
C3C6C2	527024
C3CJU3	527021
C3CME6	527021
C3D2U6	527025
C3D5H8	527025
C3DKW3	527026
C3DNJ9	527026
C3E4I5	527027
C3E741	527027
C3ELS9	527023
C3EPB0	527023
C3F2R5	527022
C3F5R1	527022
C3FLB0	527031
C3FW65	527031
C3G418	527032
C3G6U9	527032
C3GK03	527029
C3GMQ3	527029
C3H219	527030
C3H4W0	527030
C3HJM9	527028
C3HMG1	527028
C3I1W2	527019
C3I4R8	527019
C3IKF9	527020
C3IN05	527020
C3JIX1	596309
C3JKE1	596309

C3JV64	596309
C3JX61	596309
C3M8R5	394
C3PYN1	457395
C3Q2D9	457395
C3QKN0	556258
C3R413	469590
C3R4Z1	556260
C3RAU6	556260
C3T0C7	562
C3WAI9	469616
C3WBV1	469616
C3X4S5	556268
C3XAX8	556269
C3XHS0	613026
C3XNE8	556267
C4AMM2	320390
C4AQ56	320390
C4EYX7	521005
C4F596	521004
C4FBG3	521003
C4FDZ4	518635
C4FQU2	546273
C4G894	626523
C4GRS9	377628
C4H8W5	547047
C4HL61	547046
C4HXA4	545431
C4I7K0	536230
C4ID44	632245
C4IIZ4	632245
C4IN57	632245
C4IPU6	641140
C4IW29	641140
C4IWF8	641140
C4PML7	672161
C4R7K9	644223
C4RD90	219305
C4S006	349968
C4SGQ6	349967
C4SMZ5	349966
C4SYW3	349965
C4TXU6	527012
C4UDS5	527002
C4UMN5	527005
C4UX83	527004
C4V3M9	638302
C4VF92	553209
C4WAV5	596319
C4WE40	641118
C4WI29	641118
C4WLR1	641118
C4WMW4	641118
C4Y315	306902

C4YZ54	444612
C5AGF9	626418
C5E841	537937
C5ELY3	457421
C5F0V4	537972
C5F625	537973
C5F759	537973
C5N5F5	450394
C5NB39	436115
C5NLH6	436115
C5PQA8	525373
C5Q054	548474
C5Q930	525374
C5QJ26	548475
C5QSC9	525378
C5QX05	525376
C5RGJ0	573061
C5RQP4	573061
C5RTN8	573062
C5S2F4	637911
C5SLY5	573065
C5T3W8	573060
C5TEI4	555217
C5TI17	555217
C5UBF4	509193
C5URK8	536233
C5UZK0	536233
C5VCP3	553207
C5VKH9	553174
C5VTQ4	592027
C5W7I2	511693
C5ZAJ3	357347
C5ZIB6	357347
C5ZMD4	357347
C5ZYB2	537970
C6BDT2	428406
C6EKN3	469008
C6I8P2	457392
C6IG30	469586
C6IJM9	469586
C6IW19	621372
C6JE56	457412
C6JJI2	469618
C6JNE5	469618
C6M9W2	547045
C6MVY6	443143
C6N3Z7	658187
C6PQF9	536227
C6QAV1	582899
C6R2H4	553201
C6RAG4	553206
C6RF87	553219
C6RXD8	661513
C6TRR9	320371

C6TVH2	320371
C6U7Q0	320371
C6YDJ4	345072
C6YTA2	539329
C6Z2K3	457394
C6Z7M4	457394
C7CTQ7	565636
C7D4D7	565637
C7D5L6	633131
C7D606	633131
C7GAJ1	536231
C7H7M9	411483
C7HHP4	572545
C7HL88	588857
C7IFT8	588581
C7IGT3	588581
C7ILN9	588581
C7IQ79	589861
C7JMQ9	634453
C7JWX5	634454
C7K769	634455
C7KGD8	634456
C7KQQ2	634457
C7L017	634458
C7L2B6	634459
C7S9X0	562
C7U9W9	565638
C7UIL1	565642
C7UQS1	565641
C7UUE4	565650
C7V4U9	565640
C7VBV0	565644
C7VP77	565646
C7VWU5	565649
C7W3H3	565647
C7W6R4	565648
C7WJU6	565643
C7WNB1	565651
C7X2S3	565645
C7X796	563193
C7XGG2	575595
C7XU15	575594
C7Y2G6	575596
C7Y9W5	565639
C7Z3C3	660122
C7ZX03	585143
C8A3U0	585145
C8ABR9	585146
C8AKS9	585153
C8ART5	585158
C8JTE7	393124
C8K0L5	393125
C8K8R6	393128
C8KPG9	452948

C8KW83	455227
C8KXB4	591023
C8L4M4	553565
C8LCL6	553567
C8LK18	553568
C8LVE7	553571
C8M342	553573
C8M624	553581
C8ME59	553583
C8MKP2	553588
C8MVR4	553592
C8N5H1	553596
C8NA86	638300
C8NPH1	196164
C8NU12	585529
C8P7R0	525309
C8PFV3	553220
C8PXT2	553217
C8Q4E6	592316
C8Q9D6	592316
C8Q9X8	592316
C8QAG6	592316
C8QBH3	592316
C8QFB9	592316
C8QFR5	592316
C8RTN7	525262
C8S4E5	371731
C8SMC7	536019
C8SNR4	536019
C8SU66	536019
C8T3U9	667127
C8T7J2	667127
C8T9Y7	667127
C9A3J6	565653
C9CSV2	644076
C9CTU5	644076
C9CY43	644076
C9D1N4	644076
C9D1U1	644076
C9KK83	500635
C9KXD0	483215
C9L415	537007
C9LHF9	626522
C9LRX0	546271
C9M0D4	585520
C9M5X3	645512
C9MDU0	656913
C9MIA8	656912
C9MQE7	649761
C9MWZ1	634994
C9NF41	591167
C9NF51	591167
C9NGX2	591167
C9NH48	591167

C9NL43	591167
C9NT73	675814
C9NZA0	675814
C9P393	675813
C9PB15	675811
C9PMD2	667128
C9PZI9	619693
C9Q202	675810
C9QEL3	675816
C9QLI4	675816
C9QRC9	536056
C9QT20	536056
C9ST72	526221
C9T077	520459
C9T0P2	520459
C9T5A6	520459
C9T9H4	520460
C9T9Y6	520460
C9TFI7	520460
C9TKI1	520463
C9TKX1	520463
C9TPD7	520463
C9TUC5	520461
C9U0P8	520461
C9U142	520461
C9U2N9	520454
C9UA93	520454
C9UAN6	520454
C9UBV8	520452
C9UH40	520452
C9UJV5	520452
C9UL34	520451
C9UT01	520451
C9UTE7	520451
C9UX41	520450
C9V2U5	520450
C9V391	520450
C9V676	520456
C9V6N2	520456
C9V9I0	520456
C9VEC3	520457
C9VET0	520457
C9VHV9	520457
C9VQC4	520455
C9VQM4	520455
C9VRT0	520455
C9X004	604162
C9X6V5	568708
C9XC11	568708
C9Y6K4	667019
C9YCK6	667019
C9YGB2	667019
D0AUB2	575591
D0AUP1	575591

D0AX20	575591
D0B282	224914
D0B6K4	224914
D0BC98	520488
D0BHB8	520488
D0BHR7	520488
D0C1R3	575564
D0C1U3	575564
D0C8L8	575584
D0C9Y4	575584
D0CB82	575584
D0CM56	166314
D0CP12	644107
D0CUC6	644107
D0D6K7	501479
D0DAY5	501479
D0DEW8	575597
D0DRF3	575599
D0GBG0	520465
D0GBU8	520465
D0GGB2	520465
D0GLI5	596323
D0GRY8	675806
D0H2N3	675807
D0HJM0	675820
D0HLU7	675808
D0I1F6	675809
D0ICG7	675812
D0ILG5	675815
D0IRT9	290847
D0JFN3	637382
D0JPQ4	637385
D0JYX6	684950
D0KQP5	555311
D0KUS0	555311
D0N460	403677
D0P5V8	520489
D0P6A1	520489
D0PBX9	520489
D0PEV4	520487
D0PFB8	520487
D0PJW9	520487
D0RD86	437701
D0RDP8	437701
D0RGG8	437701
D0RNT5	684719
D0RUY2	469609
D0S0J1	575585
D0S5W6	575585
D0SFH3	575586
D0SYA0	575588
D0T9B5	469589
D0TPY0	469588
D0WKM0	649743

D0X0V3	674977
D0X3W7	674977
D0X8I1	673519
D0XD03	673519
D0XKL1	633149
D0YQT8	596328
D0YWF7	675817
D0ZJG6	588858
D0ZRQ3	588858
D0ZYW8	406984
D1CX18	520449
D1CZZ0	520449
D1D1N9	520449
D1DNL0	528356
D1EF03	528360
D1EIR1	520462
D1EJ51	520462
D1ERE2	520462
D1ETF8	520464
D1ETU5	520464
D1EY64	520464
D1F4B0	520466
D1F4Q2	520466
D1F7R4	520466
D1FCX9	520458
D1FDD4	520458
D1FEI5	520458
D1GU92	663951
D1J9N6	115547
D1JB00	115547
D1JNN6	469587
D1JXU3	457391
D1K4Q6	457391
D1KCJ7	655186
D1NIR9	492476
D1NTB9	561180
D1NX47	561180
D1P519	500637
D1P9U6	537011
D1PKT2	411471
D1PYS3	585502
D1QDL6	553594
D1QLX6	553601
D1QUX6	649760
D1QX77	553574
D1RPE5	682634
D1RQF2	682634
D1RRN9	682634
D1RYG4	682634
D1RYZ9	682634
D1SAA0	644283
D1SDC5	644283
D1SR75	643561
D1SUI8	643561

D1SUT1	643561
D1SXY1	643561
D1TUX2	687916
D1UCQ2	640510
D1UCR7	640510
D1UIC4	640510
D1UJD7	640510
D1USN3	640510
D1UTB2	640510
D1VB94	298654
D1VX50	679189
D1W4U4	679190
D1WNM6	596317
D1WRH7	649189
D1WW05	649189
D1WX68	649189
D1X0Q7	649189
D1X0R6	649189
D1X910	649189
D1XFW4	647653
D1XFX9	647653
D1XNX6	647653
D1XNY7	647653
D1XUY9	647653
D1XYB8	553171
D1YBB7	679194
D1YDX4	679194
D1YR94	686660
D2EQY3	563037
D2F2G6	585543
D2F6V3	585149
D2FCZ9	585151
D2FLT0	585152
D2FV84	585159
D2G1Q6	585160
D2GAK5	585161
D2GG36	585148
D2GRE5	585150
D2L037	644968
D2LCQ4	648757
D2M6M5	652103
D2M9A2	652103
D2MCT0	652103
D2MDM1	652103
D2MRX7	683082
D2MVK7	683082
D2MXU0	683083
D2N722	523796
D2NB24	431946
D2NF47	431946
D2NGW4	431946
D2NL47	431946
D2T2R3	644651
D2T6K7	644651

D2T6S6	644651
D2U224	638
D2UM16	585155
D2US13	585147
D2YFK7	671074
D2YN29	671076
D2ZA74	500639
D2ZAS9	500639
D2ZFA0	500639
D2ZQ43	521002
D2ZSD4	546266
D3AD93	566550
D3AGU3	566550
D3AI99	566550
D3AN10	566550
D3C5L7	648999
D3CGG7	648999
D3CGL5	648999
D3D7A1	102897
D3EWD7	703339
D3FKV9	567106
D3FM27	567106
D3H2X1	216592
D3HVY4	575611
D3I4B2	575612
D3IAH5	575614
D3IH03	575615
D3KQ00	393121
D3L182	592015
D3L8Q6	655225
D3LMY6	596312
D3LPF4	596312
D3M8A9	656024
D3MBB2	679195
D3MEB4	679195
D3MM18	686659
D3MM67	686659
D3MXG3	547146
D3N143	640512
D3N1H9	640512
D3N4E0	640512
D3N9Z3	640512
D3NKY6	663278
D3R6I6	552531
D3UUH5	634464
D4B6U7	500640
D4B9P1	500640
D4BA44	500640
D4BDK1	500640
D4BDK7	500640
D4BEF1	500640
D4BGK5	500640
D4BLW2	518634
D4BMW3	518634

D4BQE4	518634
D4BRN9	518634
D4BT26	521000
D4C3E3	521000
D4CDN2	411486
D4DTJ0	546263
D4E351	667129
D4E489	667129
D4E6U5	667129
D4EK55	699185
D4EUB8	699186
D4F3A3	500638
D4FGR2	679188
D4FIV0	525375
D4FSR3	655813
D4IXD8	657324
D4IZM0	657324
D4J4Q0	717962
D4JC29	717962
D4JIW4	657317
D4JJL1	657317
D4JM28	657317
D4JSV1	657319
D4K4E3	718252
D4K6B9	657322
D4KAX2	657322
D4KIB7	657316
D4KNP2	657315
D4KVB4	718255
D4LEM2	213810
D4LKN1	657323
D4LQA0	657314
D4LRH7	657314
D4M2V1	657313
D4MH88	657310
D4MMF7	717961
D4MPE8	245012
D4MSX6	245012
D4MZG2	245018
D4PK83	393117
D4PTT6	393131
D4Q520	401650
D4S162	511680
D4S4K8	585503
D4SCG6	585531
D4SVX7	427081
D4SX75	427081
D4T7N5	427082
D4TAZ9	427082
D4THW7	533240
D4TUI7	533247
D4UAC7	553590
D4UEW3	553580
D4USQ6	246199

D4V1I7	791166
D4V7F0	702446
D4V9C5	702446
D4W5Y0	702450
D4WHH9	702443
D4WQI7	702444
D4X3Q8	742159
D4X524	742159
D4X7E6	742159
D4XFS7	742159
D4XKS0	707232
D4XZ18	552811
D4YHE6	655812
D4YRI1	585524
D4ZX95	696747
D5AH50	423211
D5AX19	449216
D5CWS0	714962
D5CXB5	714962
D5D333	714962
D5H844	761659
D5H943	761659
D5HHC8	751585
D5MIK9	671143
D5N760	243261
D5N823	243261
D5NCB4	243261
D5NED2	243261
D5NFC0	243261
D5NUE5	649754
D5NXN3	649754
D5NZ89	649754
D5NZS8	649754
D5PGP9	525368
D5PI22	525368
D5PJJ1	525368
D5PMP8	525370
D5PZ19	525370
D5Q7R2	525259
D5Q7R4	525259
D5Q8F2	525259
D5Q9T9	525259
D5QEC5	714995
D5QFJ4	714995
D5QFT4	714995
D5QL76	595536
D5QW73	595536
D5RB61	525283
D5RJU6	525371
D5RMX3	525371
D5RPW9	525371
D5RS03	525371
D5RV08	525258
D5S0N8	525258

D5S0P0	525258
D5S1S2	525258
D5SCL8	762948
D5SPZ8	521674
D5TIE8	573236
D5VSK6	573063
D5VVU8	758678
D5XX86	515617
D5Y7M9	520141
D5YI53	520140
D5YUM5	515616
D5Z6T2	537209
D5ZJT3	537210
D5ZNJ8	566461
D5ZNL7	566461
D5ZT51	566461
D6AM04	457431
D6AQ53	457431
D6AS43	457431
D6AU82	457431
D6BA16	457425
D6CKX1	426114
D6D6S6	657309
D6D9P4	722911
D6DH14	717608
D6DNH5	718254
D6DS90	718254
D6E154	657318
D6E3Z6	657318
D6E6Q4	657308
D6E9R8	657308
D6EJK7	457428
D6ELA7	457428
D6ELD1	457428
D6EQ51	457428
D6FJX7	611303
D6FNF9	611304
D6GB51	469608
D6GC73	469608
D6GDH9	469608
D6GEP3	469608
D6GLK4	469608
D6GMZ2	469608
D6GYT4	585156
D6H8H7	528348
D6HGP5	585144
D6HJH1	552396
D6HZP2	550672
D6IAA5	550676
D6IJD8	656380
D6ISJ4	656380
D6J058	585157
D6JCT2	550677
D6JME6	528351

D6JRZ5	575565
D6JUZ1	575565
D6K2V4	645465
D6KAD4	645465
D6KHS8	457416
D6KMY7	450749
D6KUZ1	641146
D6L160	641147
D6LLS7	520448
D6LS38	520448
D6LTL8	520448
D6LXN9	585154
D6RZB8	525284
D6S0F5	525284
D6SH16	548470
D6SM00	555779
D6T4M7	553577
D6UAM3	762962
D6UHI6	585535
D6VA00	666684
D6VAA2	666684
D6VCX4	596153
D6VFA7	596153
D6VK15	596153
D6XP48	637913
D6XUK6	439292
D6YDC5	707184
D6YFG8	707185
D6YI34	707186
D6YKQ9	707187
D6YMH3	707183
D6YY45	718219
D7ADC8	663917
D7C0P5	749414
D7C6Q4	749414
D7CAC5	749414
D7CGU8	749414
D7DDI3	759363
D7DGV8	759364
D7EV93	515615
D7H2J1	520453
D7H4M6	520453
D7H508	520453
D7HLP4	412967
D7HXK7	693985
D7IDG5	469585
D7IEN5	469585
D7IQ94	469592
D7JAG8	585544
D7JIX3	656379
D7JRP4	656379
D7JZU4	457390
D7MYX6	641149
D7N9B9	563008

O85839	48935
Q02J95	208963
Q05UM7	221359
Q062N4	313625
Q07607	382
Q09C54	378806
Q0ASZ7	394221
Q0B3G8	339670
Q0B5V1	339670
Q0BA71	339670
Q0CZA4	341663
Q0EYP1	314345
Q0FEF6	367336
Q0FHL3	314265
Q0FKR4	314265
Q0FMG1	314265
Q0FPQ1	314265
Q0FSQ4	314265
Q0FT89	314265
Q0FX00	314265
Q0G1X1	314231
Q0G2A1	314231
Q0G4M0	314231
Q0G5L1	314231
Q0YU46	377431
Q11B87	266779
Q12FP7	296591
Q1BGI9	331271
Q1BGK9	331271
Q1BJW5	331271
Q1GKD3	292414
Q1JUQ0	192
Q1K3A8	281689
Q1NP13	262489
Q1NRT8	262489
Q1PYF4	174633
Q1UZB4	314261
Q1V0T0	314261
Q1VDQ0	314288
Q1VEU7	314288
Q1VSL1	313595
Q1VV29	313595
Q1YEJ2	287752
Q1YEZ7	287752
Q1YHC4	287752
Q1YHR3	287752
Q1YIT8	287752
Q1YKR5	287752
Q1YTD8	314287
Q1YYP2	314280
Q1ZJN8	314292
Q26BR2	156586
Q28WG2	290400
Q2C1A4	121723

Q2CDL0	314256
Q2CGC0	314256
Q2CI18	314256
Q2RSM9	269796
Q2T4S5	271848
Q39BK2	269483
Q3EJB0	339854
Q3KDV6	205922
Q3R3L8	155920
Q3R810	155920
Q3RCS7	155919
Q40KG7	332415
Q4C2C6	165597
Q4E8D5	77038
Q4EIP4	267410
Q4ETU1	267409
Q4HE01	306254
Q4HIA4	306254
Q4HQN7	306264
Q4JMV0	332980
Q4MK96	269801
Q4MP68	269801
Q546B6	1718
Q54A52	1360
Q573K6	77133
Q5B9Q2	162425
Q5J5B7	615
Q5LKY5	89184
Q5NVU8	115547
Q5PXM6	666
Q5UCH7	29486
Q5XQ83	285359
Q647W4	285356
Q648S7	285368
Q64BW6	285387
Q64D46	285362
Q6BQZ9	4959
Q6W269	394
Q732S5	222523
Q7PBV3	272951
Q7VVK3	520
Q7W621	519
Q7WHY6	518
Q7X387	562
Q89Q18	375
Q8H725	4787
Q8KJM5	381
Q8RKE9	28223
Q8YB94	29459
Q93IH0	630
Q93IK7	161725
Q977P8	2265
Q9AKJ9	783
Q9AKQ3	33991

Q9RQD0	542

dihydrodipicolinate reductase		LL-diaminopimelate aminotransferase		diaminopimelate epimerase	
**UniProt**	**TXID**	**UniProt**	**TXID**	**UniProt**	**TXID**
Q9FJ82	3702	Q93ZN9	3702	Q9LFG2	3702
D7MRM8	81972	D7MGH0	81972	D7LUJ8	81972
B9ID05	3694	A9PAK9	3694	B9T434	3988
B9R9G4	3988	B9S7T6	3988	C6T990	3847
A5BWJ3	29760	C5WNE7	4558	D2DKE8	3847
B8ALW1	39946	Q10MQ2	39947	C6TKD3	3847
Q10SL6	39947	Q6VMN8	39947	B9HCY9	3694
Q10SL7	39947	Q10MQ1	39947	Q2QNF7	39947
Q8H053	39947	B8AM24	39946	A2ZLS4	39946
B6TCY5	4577	C0PB44	4577	B4F833	4577
B6TVJ6	4577	B6TIG6	4577	B4FVI8	4577
C5X107	4558	B8LPM3	3332	C4J3L7	4577
B8HRK9	395961	A9RZ64	145481	B8LLS8	3332
Q8YU19	103690	A8IW39	3055	A8J8B3	3055
Q3MFY8	240292	C1FDC5	296587	P74667	1148
B4AZ18	497965	C1ML22	564608	A4RTF7	436017
Q9S3W8	83541	A4RTW6	436017	Q8YVD0	103690
B2J0A9	63737	Q8F814	173	Q01DU1	70448
B7FJZ3	3880	B2URC5	349741	Q3M8I9	240292
Q10YI1	203124	Q04UL5	355277	Q116P1	203124
A8YA16	267872	Q4BZ78	165597	Q5N013	269084
B4VQ48	118168	C9KJX2	500635	Q31LW2	1140
A0YTJ0	313612	D4LU96	657314	B1WRQ3	43989
D7DW84	551115	D4TD09	533240	Q4C9K0	165597
D4ZRB5	696747	Q3MAL4	240292	A0ZFP0	313624
A0ZBM1	313624	D4TN53	533247	C1E847	296587
P72642	1148	A2C4T7	167555	D7E3Y4	551115
D3ER31	713887	A5ZXT2	411459	A3IPP9	391612
C7QT35	395962	Q46IX2	59920	B2J3A7	63737
B7JW41	41431	D4LJ76	657323	D4ZQS6	696747
B7KBV6	65393	A8YN93	267872	B7K1E7	41431
Q4C722	165597	C6JBM2	457412	C7QMM0	395962
B0JU93	449447	A0ZK97	313624	B1XP39	32049
B5VUP2	513049	D3LWE8	699218	B5W4D7	513049
B1WVV1	43989	Q55828	1148	A0YIC3	313612
A3IP23	391612	B0JUM0	449447	D7G034	2880
D4TJK8	533240	A3PEY9	167546	D4TCL8	533240
D4TPX1	533247	Q7VA14	1219	B0JJ58	449447
Q8DHH2	197221	Q05QI0	221359	B7KH06	65393
Q31LA3	1140	B5JK20	382464	B0C312	329726
Q5N0M4	269084	C0B4X7	470146	A1KYM7	309035
B1XIL4	32049	C6LHV7	478749	C1MZF9	564608

B0CE31	329726	B8IZX8	525146	B4WKJ6	91464
Q2JUX2	321327	C7LN57	525897	A8YK88	267872
Q7NLC0	33072	Q01D71	70448	B8HU20	395961
Q2JMX5	321332	B2A0D8	452637	Q2JLR0	321332
Q0IAU2	64471	C8X2J2	485915	D3EP27	713887
A2BWF3	167542	D4J5D6	717962	Q8DGM2	197221
Q7V1N1	59919	A0LEA5	335543	Q2JUV6	321327
B4WJU3	91464	A1ATI6	338966	D4TMN5	533247
A5GLQ0	32051	A1VDD3	391774	B7FV89	556484
Q05SI1	221359	A2BT75	146891	Q7NMF1	33072
A3ZAN8	221360	A2BYM6	167542	B8LBN1	296543
A5GSL5	316278	A2CC97	59922	A4CUI5	59931
A2C9I1	59922	A3DK17	203119	A5GT84	316278
B9P225	93058	A4J569	349161	C9KKR6	500635
A8G504	93060	A5D192	370438	A5GL73	32051
A4CVA2	59931	A5FRC5	216389	B5IJR7	180281
Q7V7D4	74547	A5GD93	351605	C9LXX1	546271
Q3AYN5	316279	A5GIN1	32051	A3YZK9	69042
B5IMN8	180281	A5GW23	316278	Q24TW0	138119
C9RB80	429009	A5UN82	420247	C5YPW9	4558
A2BRB1	146891	A6L7E4	435590	D1NN49	492476
Q7VC38	1219	A6L8U2	435591	C7HJ64	572545
Q31AR7	74546	A8G700	93060	A3DK16	203119
Q7U807	84588	A9BCJ1	93059	B7FIP6	3880
Q065M4	313625	A9KJ19	357809	B8FSD4	272564
A3PD36	167546	B0B7W0	471472	A6VFK8	426368
B1X5K1	39717	B0BC25	471473	B0TGR9	498761
Q3AIK8	110662	B0CDH5	329726	C8W080	485916
A3Z0A9	69042	B0SEH8	355278	A9AB27	444158
D0CKU0	166314	B0SMK7	456481	Q6LYS1	39152
A2C2A9	167555	B0TA38	498761	C9LLL5	592028
Q46KV7	59920	B1GYM9	471821	A4FXV0	402880
D5WPZ0	562970	B1I544	477974	Q067B7	313625
C9LLD1	592028	B1WSG7	43989	Q0IAE7	64471
D5XF32	635013	B1XKF6	32049	Q7U6T2	84588
C8W4M5	485916	B2A250	457570	A3Z6S8	221360
B1I379	477974	B2J2U3	63737	D5X7U3	635013
A5D2T4	370438	B2KDH1	445932	C7IDA2	588581
Q24UK0	138119	B3E933	398767	Q05SY3	221359
B8FQZ3	272564	B4U7R3	380749	B1X3T5	39717
A3CMU3	388919	B5EGX2	404380	A1HMY7	401526
D0RTK6	469609	B5YE44	309799	C6D4H0	324057
C7V5N1	565640	B5YHG6	289376	Q3AJV7	110662
B0THS7	498761	B6YRL2	511995	Q3AXV6	316279
Q8EQD3	182710	B7G959	556484	A6UN47	406327
D5S7G9	760570	B7JVL5	41431	D2R217	530564

D3UPL1	683837	B7KL61	65393	B1C3N1	428126
C7W2L8	565647	B8BT02	296543	B0N3X2	445974
D4MBC3	657310	B8CX89	373903	C3RLM7	556270
C7VCN7	565644	B8DJJ6	883	A2C9T8	59922
Q834S6	1351	B8E282	515635	C4V4G3	638302
D4ETL6	699186	B8FH35	439235	B1I545	477974
D4EQD6	699185	B8HJY4	395961	D0CJ51	166314
C7YBB4	565639	B9M384	316067	D4S5H7	585503
C7X1X8	565645	B9T7N8	3988	A7AZS1	411470
C7WKP6	565643	C0QFJ4	177437	Q2RK34	264732
C7WA99	565648	C4PR66	580049	Q7V7M5	74547
C7UV05	565650	C4XSJ5	573370	C6J2A7	621372
C7UNT8	565641	C4Z4Y1	515620	B8IZX7	525146
C7UGU3	565642	C4ZG66	515619	D3E5S4	481743
C7CSV9	565636	C5WUP9	4558	A7VC93	411489
C2JK61	491075	C6BUK3	526222	A5D193	370438
C2H4Z5	525271	C6E9Q7	443144	A6CA20	344747
C2DCT5	525278	C7NIU7	478801	D4IXE0	657324
C0X5E5	491074	C7QUY5	395962	B9MLW7	521460
C7VVS6	565649	C8W082	485916	Q3AC09	246194
C7D3K6	565637	C9R854	429009	A3ZPZ9	314230
A4J5W0	349161	C9RS70	59374	D3NM52	663278
D3KQF3	393121	D2BHI6	311424	B5CS56	471875
B8DC19	552536	D2RNW3	591001	A4J568	349161
D4Q2V0	401650	D3DG71	608538	C0EZ50	411469
C4VH41	553209	D3E2N1	634498	D4M005	657313
Q71YA8	265669	D3EQB9	713887	A4XJ32	351627
Q4EFL4	267410	D3FE59	469383	B0SJ91	456481
D4PND0	393117	D3SIJ0	633145	B0SC36	355278
Q92AA1	1642	D3SN17	638303	B8CX90	373903
C8JRY6	393124	D5EUE6	264731	C4G3I1	592010
A9BAK4	93059	D5SY13	521674	C0C049	553973
C7VNE0	565646	D5X7U2	635013	B6FUM8	500632
A0AK12	386043	D6YVH0	716544	B0G1Z4	411461
C7WP49	565651	D6Z5R9	589865	B3EBJ0	398767
C7U826	565638	D7CLP6	643648	A6BDH2	411462
C6J791	621372	D7E3Y9	551115	B7ASA9	483218
D6KHS6	457416	O26158	187420	C4Z900	515619
D4V320	791166	O66630	63363	A5Z6H7	411463
Q8Y5Z6	1639	O84395	813	D5SNB0	521674
Q4EQT8	267409	Q04YV8	355276	C9R855	429009
D4PUV5	393131	Q0ID68	64471	D1N121	340101
D2P6L5	637381	Q10ZC3	203124	D4JIW6	657317
D2NV90	653938	Q118Y1	203124	C4Z3I0	515620
C8KBJ1	393128	Q18T09	272564	B6YS11	511995
C0GGI1	555088	Q1MR87	363253	B5YL43	289376

B4BKX2	495036	Q24S01	138119	D4MWP8	245018
A4IQ67	420246	Q253K9	264202	C6LHV8	478749
D6KMY5	450749	Q2JLL9	321332	B0P3K7	411484
D5AHF0	423211	Q2JS04	321327	D3AG19	566550
C6GTD5	568814	Q2LYC4	56780	D6E3Z8	657318
C6GRR1	568813	Q2NFU1	339860	B2KDH2	445932
C5VW49	218494	Q2RK33	264732	A4CFE1	87626
A4W0U7	391296	Q30ZX9	207559	B9M4N3	316067
A4VUK5	391295	Q318P3	74546	A5G8E2	351605
B9WSD6	286604	Q31PY6	1140	D7CLP5	643648
C9S0M7	544556	Q39Z65	269799	Q39ZZ8	338963
C3IYW1	550542	Q3A1U5	338963	C0GG15	555088
D3LUA6	699218	Q3AC10	246194	B0NK22	411468
C3BIB4	527000	Q3AMU5	110662	Q3IEW3	326442
D1YR92	686660	Q3AW44	316279	A0Y0F0	156578
C4FQU4	546273	Q3KLW3	315277	Q74FS5	35554
Q2B3W1	313627	Q3MDN5	240292	C6MV42	443143
D1BMK2	479436	Q3Z8H5	243164	A9BAE5	93059
B5YEB2	309799	Q3ZXC8	255470	Q2SQ64	349521
C3B1K3	526999	Q5L6M0	83555	A1AUI6	338966
Q5KXW6	1462	Q5LC03	272559	C0QAP3	177437
D7D4X4	691437	Q5N492	269084	B0ME98	411490
B6J2F3	434923	Q64SY6	817	B5EAT0	404380
D3HE35	637909	Q6AL81	84980	D4LKA8	657323
A9ZGK8	360117	Q6MDE0	264201	D4RZ76	511680
A1HSC9	401526	Q72BI1	882	C5BUL6	377629
B1HTF0	444177	Q74GT3	35554	Q39RB7	269799
D2RKF5	591001	Q7NDX4	33072	B3PEI8	498211
A7GN70	315749	Q7U4C3	84588	B6WRE7	411464
D2EQ55	563037	Q7UZZ3	59919	B9P1W1	93058
D3G0K0	398511	Q7V4Z3	74547	C6DYE5	443144
D4FU46	655813	Q824A4	83557	A5ZMM8	411459
P24703	777	Q8AAB8	818	A5KLX2	411460
B6J4U6	434924	Q8DH57	197221	Q46L14	59920
A9NA71	360115	Q8YM38	103690	A2C243	167555
A9KC25	434922	Q8YP73	103690	D3LTT0	699218
Q4MSU5	269801	Q9PK04	83560	Q7V1I3	59919
C8K292	393125	Q9Z856	83558	D7AF85	663917
B1SCD8	471872	A0B7B6	349307	Q04X60	355276
Q63DK0	288681	A0YL74	313612	Q04W97	355277
C2W655	526986	A0YXK2	313612	A2BR46	146891
Q73AW1	222523	A1HSP1	401526	Q0ACL9	187272
Q8DUL9	1309	A2SR50	410358	A8G4T8	93060
C2QQS5	526977	A3CWU1	368407	Q8F9V5	173
C3HG81	527028	A3EPN1	419542	D4XXY4	552811
C3GGK4	527029	A3INN1	391612	Q7VBZ4	1219

C3G0Q9	527032	A3YX64	69042	A8GZG9	398579
C3EZD9	527022	A3Z2H0	69042	C6Z1D8	457394
C3C042	527024	A3Z8Q5	221360	Q31AX3	74546
C2S1H6	526975	A4CST4	59931	A1TXX7	351348
B9IVQ5	361100	A5BGZ4	29760	A3JE40	270374
B7HL46	405534	A5Z615	411463	A3Q9P7	323850
B5V2V4	451708	A5ZC45	411901	D4LQR2	657314
C3IH91	527020	A6BDH3	411462	A7JUW9	272629
C3HY69	527019	A6C2S7	344747	D4V4I9	702446
C3DHM5	527026	A6NZA0	411467	A6L7E5	435590
B7GHS4	491915	A7AFG3	411477	B8F878	557723
Q02Y20	272622	A7B080	411470	B0QSW6	456298
A3IG33	388400	A7IA49	456442	A6F216	443152
C2MIJ6	526973	A7M0Y2	411476	Q1K3Q6	281689
D3HA89	365659	A7V2U1	411479	Q8R9S4	119072
Q81FP4	226900	A7VC94	411489	C0N8U7	637616
D5TV19	714359	A8RQU9	411902	Q21P78	203122
C3E1B9	527027	A8S7U3	411485	Q2BRG0	207954
C2UBN0	526982	A8SUI4	411474	Q0AIP0	335283
C2SYR0	526978	A8UWT7	392423	C5S4E6	637911
C2RKV5	526974	A8ZXV5	96561	C9L533	537007
C2BZ67	525367	B0G1W3	411461	A3PCW9	167546
C2VRG3	526985	B0M8Z2	411490	D4LE47	213810
C2TEB2	526979	B0MN95	428128	B7S2B6	247634
C2NFD8	526970	B0NK21	411468	C8KY75	591023
C1EN29	572264	B0NTM8	449673	C6PE47	580327
B7JH17	405535	B0NXC1	411484	B2A663	457570
B3ZTX3	451709	B0PBH5	445972	Q0BAY6	339670
B3Z7I6	451707	B1X4W1	39717	A3N2H9	416269
B3YRE5	405917	B3C8K3	471870	B9Z0T9	279714
A0RBY6	412694	B3JFW8	470145	A2BWL6	167542
C3FHY1	527031	B3JK42	470145	C6AQB3	634176
C3CZF2	527025	B4AW92	497965	B0TJ49	458817
C3CGJ9	527021	B4CY42	497964	P57962	747
C3GYT4	527030	B4VUN1	118168	B4S2D9	314275
C3EIW9	527023	B4WNA0	91464	Q0HQJ2	60481
C2X9L2	526989	B5CQ88	471875	Q0HN95	60480
C2WK80	526987	B5CZ47	484018	B3GYL3	537457
C2R5V6	526969	B5D1T9	484018	Q8E9H5	70863
C2MYM4	526980	B5IPW6	180281	C0CQW1	476272
B7IPB0	405531	B5W7X0	513049	C1D8Q0	557598
B7HHT6	405532	B5W8E7	513049	B9BGP9	513051
B5URT8	405533	B6AM89	419541	B3CVY6	395019
C2NWN4	526967	B6FUM7	500632	A9AC26	395019
B1S145	453362	B6W4A0	483217	D4EES7	694569
B5E6K0	512566	B6WRE6	411464	A3N4H9	320373

A2RJS9	416870	B7AKB1	483216	C7ITQ2	589861
C6SR04	511691	B7ASB1	483218	C7HR72	588857
C2YPF3	526992	B7BAG4	537006	C5UAG1	509193
C2PCQ0	526971	B8AQ26	39946	A0KS65	94122
C2Y8G8	526991	B8GI82	521011	Q5FQZ4	442
Q5WGQ4	66692	B9AE27	483214	Q39BY7	269483
B8E2S7	515635	B9HFK8	3694	D0KXR5	555778
B2IR75	516950	B9MXH8	3694	Q1PZ14	174633
B2DMV4	453363	B9NYK1	93058	D5C3E8	472759
A5LSC2	406558	B9RJE0	3988	C5V604	395494
C1C8E8	488221	C0C048	553973	B0BRC5	434271
B2DUA2	453364	C0CJ66	476272	C9R2C0	668336
B2DJ43	453361	C0CXE4	518636	C7R6T0	523791
B1ICX7	487214	C0EAG2	537013	A8KK52	331978
C2UT52	526983	C0EZ51	411469	A4YBF7	319224
B2E398	453366	C0FNL3	622312	A2UYN5	399804
A0B7E0	349307	C0GG14	555088	A1RPD2	351745
A0JUX2	290399	C0WB45	563191	D0Z0U1	675817
A0K4H7	331272	C2KW99	585501	Q0BS04	391165
A0KLT2	380703	C3PYN4	457395	B8CHW4	225849
A0KTT2	94122	C3QJF9	556258	B1K0F5	406425
A0L4Z4	156889	C3QYL3	469590	D1BPE4	479436
A0LEA6	335543	C3R4Z4	556260	A0AKC4	386043
A0LV18	351607	C4FSJ0	546273	A0B3V6	331272
A0LZI4	411154	C4G914	626523	A0B6C1	349307
A0PQF7	362242	C4PMP7	672161	A0JUY8	290399
A0PQF8	362242	C5EHK7	457421	A0KBF6	331272
A0PZM2	386415	C5TVY7	573059	A0KFI1	380703
A0Q8L6	401614	C6HWT7	412449	A0LE31	156889
A0Q9R9	243243	C6ICR6	457392	A0LEA8	335543
A0QBL2	243243	C6IH47	469586	A0LUZ5	351607
A0QDJ4	243243	C6MVY8	443143	A0LZ07	411154
A0QDR5	243243	C6PMM1	536227	A0PT80	362242
A0QEP8	243243	C6TCD0	3847	A0Q076	386415
A0QIV1	243243	C6Z1D9	457394	A0QIQ8	243243
A0QIV4	243243	C7GD62	536231	A0QVY0	246196
A0QMI2	243243	C7H522	411483	A0RKC7	412694
A0QPY3	246196	C7HJ63	572545	A0RRQ3	360106
A0QQF1	246196	C7IDA1	588581	A1A2L5	367928
A0QVR3	246196	C7XE85	563193	A1AHX7	405955
A0QXI7	246196	C9KW44	483215	A1AWJ5	413404
A0R0D7	246196	C9L460	537007	A1AZ15	318586
A0R2Z5	246196	C9LFU6	626522	A1BD08	290317
A0RMR7	360106	C9LL97	592028	A1JI90	393305
A1A301	367928	C9LSX6	546271	A1K306	62928
A1A780	405955	D0CL43	166314	A1KM64	410289

A1ATI7	338966	D0TIE2	469589	A1KT22	272831
A1AU34	338966	D0TNY6	469588	A1R552	290340
A1AX89	413404	D1A0J5	406984	A1SAP5	326297
A1B5T8	318586	D1BR52	479436	A1SNF4	196162
A1BI80	290317	D1JPF0	469587	A1SQZ9	357804
A1JJE5	393305	D1JXU6	457391	A1SZV6	357804
A1K8N8	62928	D1N122	340101	A1T7U8	350058
A1KMB5	410289	D1NN50	492476	A1TKA9	397945
A1KRN3	272831	D1PHW5	537011	A1UEZ9	189918
A1R536	290340	D1PNX9	411471	A1UQV5	360095
A1RGQ6	351745	D1U3Q1	643562	A1V7L3	320388
A1S8K1	326297	D1YNA0	686660	A1VD00	391774
A1SDT3	196162	D1YWW0	304371	A1VSV4	365044
A1SLI4	196162	D2F273	585543	A1W1D0	354242
A1T0R6	357804	D2L7V1	644968	A1WC24	232721
A1T7N8	350058	D2RDI5	572546	A1WRJ4	391735
A1TBK3	350058	D2ZQH7	521002	A1WWB3	349124
A1TEU5	350058	D3AG20	566550	A2S862	412022
A1TVV2	397945	D3MWB4	547146	A2SKV9	420662
A1U612	351348	D3NM51	663278	A2SQX8	410358
A1UEU3	189918	D3RWW4	589924	A3CXI3	368407
A1UII6	189918	D3UUK5	634464	A3D9Q0	325240
A1ULD2	189918	D4C7W9	411486	A3M804	400667
A1UTM3	360095	D4CM94	608534	A3MRQ6	320389
A1V0H0	320388	D4IZ77	657324	A3NQ71	357348
A1VDM6	391774	D4JM32	657317	A3PMY1	349101
A1VTV7	365044	D4JQX4	657319	A3PYF9	164757
A1VXS5	354242	D4JYF2	718252	A3UAS7	216432
A1WCQ3	232721	D4KBN2	657322	A4CLX3	313596
A1WGT9	391735	D4KDG0	657316	A4FAJ6	405948
A1WX29	349124	D4KN13	657315	A4G980	204773
A2S5J7	412022	D4KZK4	718255	A4ISE8	420246
A2SC94	420662	D4L4P5	657321	A4JIQ6	269482
A2SQU7	410358	D4LE48	213810	A4QEV1	340322
A3CVI6	368407	D4MID7	717961	A4SGM1	290318
A3D7S3	325240	D4MZD8	245018	A4SRW9	382245
A3DE18	203119	D4RZ75	511680	A4T0F3	312153
A3DEM3	203119	D4UR02	246199	A4TCN3	350054
A3DKP5	399550	D4V4J0	702446	A4TRA3	386656
A3MA87	400667	D4VH64	702447	A4VGW5	379731
A3MPI2	320389	D4WM69	702443	A4WG08	399742
A3N048	416269	D4WYW4	702444	A4WWV1	349102
A3NDK8	320373	D4YDS2	655812	A4X4W2	369723
A3NZB9	357348	D4ZR68	696747	A4XNX4	399739
A3PNF3	349101	D4ZUW6	696747	A4YKA5	114615
A3PYA0	164757	D5E6U9	547558	A5CSL3	443906

A3Q2C7	164757
A3Q2H3	164757
A3Q5T1	164757
A3QGV8	323850
A3U8E2	216432
A4CQ00	313596
A4FM20	405948
A4FXV6	402880
A4G8E2	204773
A4JBH8	269482
A4QEY0	340322
A4S841	436017
A4SD53	290318
A4SLF8	382245
A4SVD8	312153
A4T266	350054
A4T275	350054
A4T700	350054
A4TCI6	350054
A4TQE9	386656
A4VPQ3	379731
A4W6E7	399742
A4WWP7	349102
A4X4Q0	369723
A4XJT9	351627
A4XYF4	399739
A4Y9M3	319224
A4YJP9	114615
A5CCS0	357244
A5CSN2	443906
A5CW12	412965
A5E8K0	288000
A5EVH3	246195
A5FEM3	376686
A5FNI0	376686
A5FQT3	216389
A5FXD9	349163
A5GD90	351605
A5HZY4	441771
A5I6N3	441771
A5I9V1	400673
A5IM63	390874
A5ISS8	359786
A5N115	431943
A5N262	431943
A5U6C6	419947
D5HHF3	751585
D5MIK6	671143
D5R8I6	642492
D6D4K6	657309
D6DI91	717608
D6E150	657318
D6E6I8	657308
D6KL85	457416
D6KR81	450749
D6SLY7	555779
D6YDF3	707184
D6YFJ7	707185
D6YI63	707186
D6YKT8	707187
D6YMK1	707183
D6YY75	718219
D7ADD0	663917
D7EAR9	644295
D7II92	469585
D7IVX4	469592
D7J761	585544
D7JF59	575590
D7K9E0	457390
O29838	2234
Q061A0	313625
Q0W3B8	351160
Q12X74	259564
Q1K3A6	281689
Q1NV23	262489
Q2FU41	323259
Q469C7	269797
Q647Q2	285401
Q6VMN7	39947
Q8H7W8	39947
Q8PTR4	2209
Q8TQ40	2214
A5CWN5	412965
A5E915	288000
A5EWY0	246195
A5FP09	376686
A5FRC6	216389
A5G1F7	349163
A5I336	441771
A5IHM9	400673
A5IM61	390874
A5N7G4	431943
A5U678	419947
A5UDX5	374930
A5UHQ4	374931
A5UMZ9	420247
A5VAG4	392499
A5VJ53	557436
A5VSQ7	444178
A5W425	351746
A5WAU5	351746
A5WCB3	349106
A5WQX7	336982
A6GYX5	402612
A6L8U3	435591
A6LP57	391009
A6LTU4	290402
A6Q160	387092
A6QCY7	387093
A6T2Z4	375286
A6TGJ2	272620
A6TRX5	293826
A6UE39	366394
A6UTH4	419665
A6VE52	381754
A6VQR8	339671
A6VT35	400668
A6W1B0	400668
A6W850	266940
A6WTF5	402882
A6WXE4	439375
A7FD74	349747
A7FUW7	441770
A7GEM4	441772
A7GUE1	315749
A7H5P5	360109
A7H7Y0	404589
A7HJ55	381764

A5UCB3	374930	A7HT02	402881
A5UF10	374931	A7HZM9	360107
A5ULF7	420247	A7IB34	456442
A5UY21	357808	A7ICC6	78245
A5V2Q9	392499	A7K5A2	150340
A5V819	392499	A7MQK2	290339
A5VJ59	557436	A7N0W0	338187
A5VVV4	444178	A7Z8D3	326423
A5W9A1	351746	A7ZAX9	360104
A5WBG0	349106	A7ZU14	331111
A5WR24	336982	A8A6R7	331112
A6GYP2	402612	A8ACV7	290338
A6KZT3	435590	A8ER55	367737
A6LFU6	435591	A8EZ08	293613
A6LLG5	391009	A8F1I9	416276
A6LP59	391009	A8F8L9	416591
A6LUD6	290402	A8FH15	315750
A6LXJ7	290402	A8FNJ1	407148
A6Q596	387092	A8G0W0	425104
A6Q6T6	387093	A8G852	399741
A6QGU7	426430	A8GND7	293614
A6T235	375286	A8GS10	392021
A6T4H8	272620	A8GWH4	391896
A6TQQ3	293826	A8IN67	438753
A6TT16	293826	A8L6K2	298653
A6U1L7	359787	A8LMN6	398580
A6UEW2	366394	A8M7X0	391037
A6UNQ4	406327	A8MH77	350688
A6UUT2	419665	A8YUS7	405566
A6VCL6	381754	A8Z693	444179
A6VFL4	426368	A9A044	96561
A6VPE4	339671	A9BUI9	398578
A6VUF0	400668	A9FMF9	448385
A6W817	266940	A9HM33	272568
A6WRU0	402882	A9IH28	340100
A6WYJ3	439375	A9IZW1	382640
A7FMD2	349747	A9KGY8	434922
A7FS48	441770	A9KJ18	357809
A7FYA2	441770	A9KKW6	357809
A7GBI9	441772	A9L5F5	399599
A7GI20	441772	A9M3J3	374833
A7GWC2	360105	A9M8R8	483179
A7H1R4	360109	A9MY70	272994
A7H774	404589	A9N9J6	360115
A7H7A8	404589	A9R8K5	349746
A7HJ57	381764	A9VN01	315730

A7HNJ8	381764	A9W6A5	419610
A7HZ36	402881	A9WQ49	288705
A7I316	360107	B0B802	471472
A7I854	456442	B0BC67	471473
A7IC74	78245	B0BXH3	452659
A7K451	150340	B0CIT6	470137
A7MI96	290339	B0K299	399726
A7MXR0	338187	B0KA18	340099
A7NN21	383372	B0KQ41	76869
A7X273	418127	B0RHB0	31964
A7Z600	326423	B0RNK1	509169
A7ZB70	360104	B0T542	366602
A7ZHC0	331111	B0U6U5	405440
A7ZVX9	331112	B0UCI8	426117
A8ALS4	290338	B0UWU6	228400
A8EWI3	367737	B0VDN4	509173
A8EXL6	293613	B0VT30	509170
A8F0Q6	416276	B1GYN2	471821
A8F564	416591	B1IMS5	498213
A8FEI3	315750	B1IW95	481805
A8FJZ8	407148	B1J1W0	390235
A8FYT6	425104	B1JPE1	502800
A8G9M8	399741	B1K2V7	406425
A8GMB6	293614	B1KQD0	392500
A8GQX3	392021	B1KTZ0	498214
A8GV42	391896	B1LBR2	126740
A8H756	398579	B1LLY0	439855
A8IH01	438753	B1LY98	426355
A8J8G6	3055	B1MD00	561007
A8L6G3	298653	B1VXU0	455632
A8L9J9	298653	B1XAH6	316385
A8LL24	398580	B1XSI3	452638
A8M760	391037	B1Y6S6	395495
A8MDJ0	397948	B1YPQ6	398577
A8MF41	350688	B1ZDT1	441620
A8MGL6	350688	B1ZZ82	452637
A8YUT4	405566	B2AGE5	164546
A8Z3X4	451516	B2FUW0	522373
A8Z627	444179	B2G6M4	557433
A9AB21	444158	B2GC06	334390
A9AHB2	395019	B2GKG8	378753
A9B6A5	316274	B2HL16	216594
A9BHR7	403833	B2HX82	405416
A9C1H2	398578	B2IA16	405441
A9FHA9	448385	B2IEM1	395963
A9HE99	272568	B2JJY4	391038

A9IGA9	340100
A9IXE5	382640
A9KHY5	357809
A9L0Q8	399599
A9M3H7	374833
A9MCX7	483179
A9MYI6	272994
A9NGC1	441768
A9R003	349746
A9T9L2	145481
A9VME3	315730
A9W5P6	419610
A9WK53	324602
A9WQ18	288705
A9WW61	470137
B0B7T3	471472
B0BBZ8	471473
B0BNW4	434271
B0BWB9	452659
B0K0N9	399726
B0K4I4	399726
B0K982	340099
B0KAL8	340099
B0KIS3	76869
B0RGX5	31964
B0RSP5	509169
B0SCT1	355278
B0SL59	456481
B0T684	366602
B0TQB8	458817
B0TWJ3	484022
B0U565	405440
B0UBD3	426117
B0UU27	228400
B0VA26	509173
B0VPZ8	509170
B1GYN0	471821
B1IFB1	498213
B1IMW9	498213
B1IRE5	481805
B1J256	390235
B1JKZ4	502800
B1JVG6	406425
B1KRS8	392500
B1KWZ3	498214
B1L0V2	498214

B2K072	502801
B2S885	430066
B2SMW6	360094
B2T1P5	398527
B2TRP9	508765
B2TUW5	344609
B2UD25	402626
B2ULA2	349741
B2UTR2	512562
B2V4U0	508767
B2V734	436114
B2VI49	338565
B3CME7	570417
B3DQ52	205913
B3DY27	481448
B3EEP7	290315
B3EL46	331678
B3PR12	491916
B3Q277	491916
B3Q862	395960
B3QL52	517417
B3QXC3	517418
B3W7F0	543734
B4E7Z8	216591
B4ELE6	216591
B4F1X7	529507
B4RCE2	450851
B4RK32	521006
B4S482	290512
B4SGB7	324925
B4SJN4	391008
B4SZ51	423368
B4TB37	454169
B4TNV9	439843
B4U9P0	380749
B4UCM0	447217
B5BIV6	554290
B5ENS1	380394
B5EZS3	454166
B5FCR0	388396
B5FNU5	439851
B5QW52	550537
B5RFP9	550538
B5XWW5	507522
B5XYK7	507522
B5YD87	309799

B1LBR0	126740		B5YY56	444450
B1LFW1	439855		B5Z6T7	563041
B1M6A6	426355		B5ZD84	272568
B1MD52	561007		B5ZTH4	395492
B1MI01	561007		B6EP85	316275
B1MZM7	349519		B6I4F0	409438
B1VDC2	504474		B6IP82	414684
B1VXZ4	455632		B6J395	434923
B1XBF6	316385		B6J630	434924
B1XT71	452638		B6JAP3	504832
B1XXY2	395495		B6JLE8	570508
B1YJ40	262543		B6VLR4	553480
B1YT08	398577		B7GKM1	491915
B1ZA87	441620		B7GPZ2	391904
B1ZN18	452637		B7GY71	557600
B2A1K9	457570		B7HBI0	405532
B2FQ70	522373		B7HUS8	405534
B2G6N0	557433		B7I5Y5	480119
B2GC12	334390		B7IF12	484019
B2GKI1	378753		B7IMV5	405531
B2HHT7	216594		B7J8H2	243159
B2HKW4	216594		B7JDJ2	405535
B2HKW6	216594		B7KQM2	440085
B2I2G4	405416		B7L969	585055
B2I841	405441		B7LU47	585054
B2IBR8	395963		B7M611	585034
B2JGX9	391038		B7MH73	585035
B2K3N1	502801		B7MR29	585397
B2KAQ8	445932		B7NFB1	585056
B2RM71	431947		B7NTD3	585057
B2SC36	430066		B7UNC6	574521
B2SV83	360094		B7V5G9	557722
B2SYN9	398527		B7VMD4	575788
B2TS69	508765		B8D8A8	561501
B2U250	344609		B8D8F1	563178
B2UCA2	402626		B8DBR3	552536
B2UMF1	349741		B8DKT9	883
B2UTX8	512562		B8DT02	442563
B2V5A9	508767		B8DZX7	515635
B2V6A7	436114		B8E690	407976
B2VH06	338565		B8EM14	395965
B3CN84	570417		B8FH34	439235
B3CQD8	334380		B8GJ70	521011
B3DS96	205913		B8GT89	396588
B3DTC9	205913		B8GVW2	565050
B3DY25	481448		B8HG93	452863

B3E935	398767
B3EG74	290315
B3EN04	331678
B3ER88	452471
B3H1A7	537457
B3PBC1	498211
B3PSH1	491916
B3PXK4	491916
B3Q978	395960
B3QKY9	517417
B3QYM8	517418
B3R6R4	164546
B3R731	164546
B3W7E4	543734
B4EEW4	216591
B4F2T9	529507
B4RC37	450851
B4RR35	521006
B4S0T3	314275
B4S4K2	290512
B4SEU9	324925
B4SHT7	391008
B4T6J1	423368
B4TIG4	454169
B4TWQ6	439843
B4U6J3	380749
B4UHB6	447217
B4UHF2	447217
B5BL41	554290
B5EGX3	404380
B5ENA1	380394
B5F744	454166
B5FA64	388396
B5FHF8	439851
B5R1Q4	550537
B5RG99	550538
B5Y210	507522
B5YGJ4	289376
B5YYC3	444450
B5Z6N2	563041
B5ZTW4	395492
B5ZWT3	395492
B6ENC8	316275
B6HYX9	409438
B6IW52	414684
B6JCI8	504832
B8I9H6	460265
B8JC32	455488
B8ZQU1	561304
B9E0X7	583346
B9J3D8	361100
B9JCL3	311403
B9JUB9	311402
B9K864	309803
B9KED6	306263
B9KHB4	320483
B9KN64	557760
B9L726	598659
B9LUB6	416348
B9MFT0	535289
C0Q3B8	476213
C0QQ59	123214
C0QZX1	565034
C0R4N2	66084
C0RFH7	546272
C0ZDI1	358681
C0ZH35	358681
C0ZYN8	234621
C1A8U3	379066
C1AFI5	561275
C1B393	632772
C1DJ56	322710
C1DV08	204536
C1EX98	572264
C1F6C9	240015
C1FPD9	536232
C3K436	216595
C3K7V6	216595
C3KXG8	515621
C3LC72	568206
C3LPW8	579112
C3MAG8	394
C3NUA6	593588
C3PDH0	592021
C3PGX6	548476
C3PNE1	347255
C4K2A3	562019
C4K4D9	572265
C4KD15	85643
C4KMC0	536230
C4LDU8	595494
C4LJ46	645127

B6JL92	570508	C4PRA8	580049
B6SNS9	4577	C4WZT7	484021
B6YQZ6	511995	C4XHA3	573370
B7GUF6	391904	C4ZZ73	595496
B7GV10	557600	C5AEZ7	626418
B7I2B0	480119	C5AUQ9	272630
B7IF14	484019	C5BBD4	634503
B7IHP8	484019	C5BWM2	471853
B7J7X7	243159	C5C9V7	465515
B7KNY0	440085	C5CHY0	521045
B7L4F3	585055	C5CND4	543728
B7LWM1	585054	C5D729	471223
B7M0C7	585034	C6A919	580050
B7MAF2	585035	C6AB14	634504
B7MNN7	585397	C6AF61	555970
B7N7Q5	585056	C6AZ03	395491
B7NHD5	585057	C6BK76	428406
B7UI76	574521	C6BU44	526222
B7V1H1	557722	C6C7D1	579405
B7VJ13	575788	C6CGK6	561229
B8C5E0	35128	C6DHB7	561230
B8CKF7	225849	C6DLI2	478434
B8CWF1	373903	C6S628	662598
B8D005	373903	C6UIQ4	413997
B8D751	561501	C6UZU7	544404
B8D8U7	563178	C6VRA1	644042
B8DJT5	883	C6W7U7	471854
B8DT87	442563	C6WAC3	446462
B8DWI3	442563	C6WT34	583345
B8E4S9	407976	C6X3R8	531844
B8EIQ3	395965	C6X8F3	582744
B8F8Q1	557723	C6XFN0	537021
B8FI97	439235	C6XKY1	582402
B8GKG6	521011	C6XYG7	485917
B8GNX3	396588	C7C055	592205
B8H668	565050	C7C8I1	661410
B8HG77	452863	C7JG03	634452
B8I6K9	394503	C7LEG1	568815
B8I6L5	394503	C7LKJ5	595499
B8IC99	460265	C7LN01	525897
B8J0P9	525146	C7LY03	525909
B8J4D1	525146	C7M779	521097
B8JAK0	455488	C7MBD3	446465
B8JAN4	455488	C7MVB6	471857
B8ZLL9	561276	C7NAR4	523794
B9DP24	396513	C7P6F3	573064

B9DRX8	218495
B9E4W6	583346
B9E5W4	583346
B9EBZ7	458233
B9J6R4	311403
B9JYQ3	311402
B9K866	309803
B9KDT6	306263
B9KJ33	320483
B9KP40	557760
B9KY70	309801
B9KZJ1	309801
B9L6C6	598659
B9LC21	480224
B9LUB3	416348
B9M381	316067
B9MH71	535289
B9MRD0	521460
B9N8M7	3694
C0Q4K5	476213
C0QFZ5	177437
C0QU79	123214
C0R175	565034
C0R2S2	66084
C0RMJ0	546272
C0ZCG4	358681
C0ZYG3	234621
C1A410	379066
C1AFN6	561275
C1B356	632772
C1B6H2	632772
C1CFE0	488222
C1CLQ6	488223
C1CSH7	487213
C1D7H9	557598
C1DFM1	322710
C1DWF3	204536
C1F656	240015
C1FL30	536232
C1FUM4	536232
C3KTL5	515621
C3L287	515621
C3L8Q7	568206
C3LQT6	579112
C3MBY7	394
C3NVK7	593588
C7PKR5	485918
C7QBG7	479433
C7R3G9	471856
C7RPB8	522306
C7T8R6	568703
C7TFG1	568704
C8TL08	573235
C8TYP7	585395
C8UJJ1	585396
C8WFV8	622759
C8WXU3	521098
C8X410	485915
C8XH43	479431
C9RGT5	579137
C9RS69	59374
C9RUH8	544556
C9XMV9	645462
C9Y4V6	413502
C9YPG3	645463
C9Z0N1	680198
D0FMZ4	79967
D0J7E6	688245
D0J951	600809
D0JB68	331104
D0KBL9	561231
D0LBP8	526226
D0LTS4	502025
D0MG97	518766
D0R4B9	633699
D0Z995	498217
D1AAA7	471852
D1ANM8	526218
D1ATB2	574556
D1AYT7	519441
D1AYX2	525898
D1B7Q0	525903
D1BJ30	446469
D1C095	446471
D1C945	479434
D1YWV8	304371
D2AZH2	479432
D2BHI7	311424
D2BYP5	590409
D2C3R8	590168
D2NNR0	680646
D2NVY8	653938

C3P5Q2	592021
C3PH06	548476
C3PMI6	347255
C4K0R8	562019
C4K485	572265
C4KV80	536230
C4L2D3	360911
C4LCQ1	595494
C4LCT8	595494
C4LJ69	645127
C4PR39	580049
C4T9D7	573
C4XRI5	573370
C4Z2K4	515620
C4ZG59	515619
C4ZPV7	595496
C5AAZ6	626418
C5ARE3	272630
C5B7N2	634503
C5BQ31	377629
C5BWQ6	471853
C5C9U2	465515
C5CDJ7	521045
C5CHX8	521045
C5CJ71	543728
C5D3B9	471223
C5WQZ8	4558
C6A981	580050
C6ADF5	634504
C6AFC3	555970
C6AMN0	634176
C6B275	395491
C6B2X6	395491
C6BDR0	428406
C6BTW9	526222
C6C9F1	579405
C6CJ56	561229
C6CUF6	324057
C6DF00	561230
C6DX71	478434
C6E3G6	443144
C6E9Q8	443144
C6S997	662598
C6ULD4	413997
C6UVL5	544404
C6VQJ8	644042

D2P6X7	637381
D2PZC1	479435
D2Q5K6	401473
D2QN21	504472
D2RGB0	572546
D2RNW2	591001
D2RX74	543526
D2SDR0	526225
D2TV21	637910
D2UF95	380358
D3DK68	608538
D3DYT5	634498
D3FE58	469383
D3FYZ9	398511
D3HPP4	661367
D3NSZ4	137722
D3PAV2	639282
D3Q6H1	446470
D3QXA1	701177
D3RDI8	640131
D3RH88	640131
D3RV79	572477
D3S0G1	589924
D3S6T6	644281
D3SCB6	396595
D3SIJ1	633145
D3SMD8	638303
D3T2Y1	580331
D3UFT0	679897
D3UPW8	683837
D3V6I8	406818
D3VHZ7	406817
D4G844	515618
D4GGN1	706191
D4GW20	309800
D4H4C1	522772
D4HDH9	553199
D4HUK9	665029
D4ICJ0	716540
D4Z4Q2	452662
D4ZCR3	637905
D5ASV9	272942
D5B588	637386
D5BKJ9	655815
D5BML2	488538
D5CFU1	716541

C6VX07	471854	D5CU55	580332
C6WDJ5	446462	D5D900	641892
C6WXG3	583345	D5DM00	592022
C6X3R2	531844	D5DV76	545693
C6X470	531844	D5ECU4	572547
C6X6X9	582744	D5EJL8	583355
C6XHK9	537021	D5EUD3	264731
C6XII4	582402	D5H2T4	748671
C6XTE2	485917	D5T7Z0	423212
C7BJY6	553480	D5TN00	714359
C7BZY4	592205	D5U513	526224
C7CMB1	661410	D5UDP8	446466
C7JC27	634452	D5UMD7	521096
C7LJG9	568815	D5V4F8	572480
C7LKC2	595499	D5V853	749219
C7LNT8	525897	D5VQ30	509190
C7LXZ1	525909	D5W9V5	640511
C7M8H8	521097	D5WP68	562970
C7MBB5	446465	D5X3Z2	75379
C7MNH3	469378	D6XWT0	439292
C7MQW5	471857	D6Y834	469371
C7N470	471855	D6YRW3	716544
C7N8S9	523794	D6Z6B6	589865
C7NIU9	478801	D6ZEP6	640132
C7NV79	519442	D6ZVC2	759350
C7P2G0	485914	D7A8U5	639283
C7P974	573064	D7APG8	583358
C7PCE0	485918	D7AYD7	446468
C7QF62	479433	D7D0R8	691437
C7QFC0	479433	D7DKX7	666681
C7R3E7	471856	D7DTY6	456320
C7R906	523791	D7E856	644295
C7RKR9	522306	D7FDR3	693745
C7T8R0	568703	D7GE64	754252
C7TFF4	568704	O25290	210
C8TGF9	573235	O27389	187420
C8U1A4	585395	O29511	2234
C8UG03	585396	O32114	1423
C8WA09	521095	O67693	63363
C8WBM7	622759	O69969	1902
C8WHK4	479437	O84437	813
C8WLB0	479437	P44859	727
C8WX46	521098	P46357	632
C8X0Z7	485915	P46814	1769
C8XJD9	479431	P54897	103690
C9R3J7	668336	P59582	224915

C9RI57	579137	P63897	1773
C9RPN4	59374	P63898	1765
C9XKA1	645462	Q01XQ8	234267
C9XRH5	645462	Q02E84	208963
C9XRH8	645462	Q03CV8	321967
C9XVJ2	413502	Q03HS7	278197
C9YIJ1	645463	Q056W9	372461
C9YR68	645463	Q05FG2	387662
C9YR71	645463	Q07UV4	316055
C9Z238	680198	Q088K8	318167
D0FPD8	79967	Q0BWE3	228405
D0IX77	688245	Q0I572	205914
D0J8Q2	600809	Q0KF42	381666
D0JBL4	331104	Q0RDX7	326424
D0K4X1	681288	Q0S1N7	101510
D0KMM5	561231	Q0SRX8	289380
D0L121	555778	Q0SZ00	373384
D0L5Y3	526226	Q0TAR6	362663
D0LBK6	526226	Q0TPB1	195103
D0LJC9	502025	Q0VM18	393595
D0MJ85	518766	Q0W3B6	351160
D0R4B3	633699	Q11CW3	266779
D0ZC37	498217	Q11YU6	269798
D1AAE6	471852	Q12EN2	296591
D1ANL6	526218	Q12S90	318161
D1AU63	574556	Q13DT7	316057
D1AYU4	519441	Q146W2	266265
D1AZ73	525898	Q15NG5	342610
D1B723	525903	Q16C03	375451
D1BJ56	446469	Q17XQ0	382638
D1C061	446471	Q182T1	272563
D1CED2	525904	Q18K13	362976
D1YV25	304371	Q1AZV3	266117
D2AXU7	479432	Q1BA13	164756
D2BI22	311424	Q1BJS1	331271
D2BKJ8	684738	Q1BSN5	331271
D2BWR0	590409	Q1CBM6	360102
D2C3S0	590168	Q1CNH8	377628
D2NNS8	680646	Q1CTW0	357544
D2Q1N8	479435	Q1D2B4	246197
D2Q5Y5	401473	Q1GCS5	292414
D2QD79	504472	Q1GPI7	117207
D2R4F5	530564	Q1H4X0	265072
D2RG37	572546	Q1I303	384676
D2RX77	543526	Q1INC7	204669
D2SDV2	526225	Q1LS37	266264

D2TGL8	637910
D2UAC7	380358
D3DFK6	608538
D3DYX0	634498
D3E7M7	481743
D3FE36	469383
D3HMN3	661367
D3P5H5	137722
D3PAV4	639282
D3QAU9	446470
D3QCX7	698737
D3QVB5	701177
D3RCJ3	640131
D3RUB7	572477
D3S0A3	589924
D3S698	644281
D3SBD0	396595
D3SJ24	633145
D3SLK9	638303
D3SZN9	547559
D3T7X4	580331
D3UIA9	679897
D3V283	406818
D3VB50	406817
D4G8Y0	515618
D4GJN0	706191
D4GW26	309800
D4H4C3	522772
D4HBK2	553199
D4HF16	553199
D4I0H2	665029
D4IF21	716540
D4Z2F5	452662
D4ZB83	637905
D5ALX0	272942
D5B0B5	637386
D5BKC0	655815
D5BUA9	488538
D5C1C6	472759
D5CH23	716541
D5CQQ3	580332
D5D8T4	641892
D5DBW5	592022
D5E0F3	545693
D5EC22	547558
D5EC79	572547

Q1LTX0	374463
Q1MAK9	216596
Q1MPX8	363253
Q1QE99	335284
Q1QHJ3	323097
Q1QSV1	290398
Q1R4C5	364106
Q1RIT1	336407
Q1WTU5	362948
Q21CU6	316056
Q21SV0	338969
Q253I6	264202
Q2G8H7	279238
Q2GI52	205920
Q2ING7	290397
Q2J390	316058
Q2K386	347834
Q2L1A2	360910
Q2LV89	56780
Q2LYC5	56780
Q2NBR6	314225
Q2NE08	339860
Q2NQF4	343509
Q2NYV5	342109
Q2RV61	269796
Q2S2P2	309807
Q2SG43	349521
Q2T269	271848
Q2WAV6	342108
Q2Y5Z0	323848
Q2YLR4	359391
Q30NW1	326298
Q310Q1	207559
Q31IM2	317025
Q31UH9	300268
Q329Y5	300267
Q39BE9	269483
Q3ATR0	340177
Q3B1G2	319225
Q3BXU3	316273
Q3IZB6	272943
Q3JEA2	323261
Q3K4R8	205922
Q3KLS0	315277
Q3MBA2	240292
Q3SFX7	292415

D5EJ56	583355
D5EWH1	264731
D5H2U0	748671
D5STR2	521674
D5T5I2	762051
D5T6E9	423212
D5UAX3	526224
D5UD82	446466
D5UM94	521096
D5V5S9	572480
D5V9G8	749219
D5VDV8	509190
D5WD67	640511
D5X2V6	75379
D6XUX6	439292
D6Y7X3	469371
D6YVH2	716544
D6Z6B8	589865
D6Z922	640132
D6ZMA7	525381
D6ZSW0	759350
D7A243	639283
D7A7X5	639283
D7AU45	583358
D7AYA2	446468
D7CM63	643648
D7DCF5	591019
D7DK14	666681
D7DTZ2	456320
D7EB03	644295
D7FDJ4	693745
D7GEJ4	754252
O26891	187420
O29353	2234
O67061	63363
O84369	813
O86836	1902
P38103	287
P40110	1718
P42976	1423
P45153	727
P57867	747
P58210	381
P58211	381
P58326	382
P59474	224915

Q3SNW2	323098
Q3YT78	269484
Q3YVF7	300269
Q3Z8H4	243164
Q3ZXC7	255470
Q46185	1502
Q476V3	264198
Q47JN0	159087
Q47RR3	269800
Q48AR8	167879
Q48C02	264730
Q491Z7	291272
Q4FP10	198252
Q4FV85	259536
Q4JV83	306537
Q4K3W2	220664
Q4KDN7	220664
Q4QME8	281310
Q4ULS7	42862
Q4UYY2	314565
Q500B6	205918
Q51564	287
Q54HH7	44689
Q55CJ6	44689
Q55CJ7	44689
Q55GS8	44689
Q57AW6	235
Q57HR0	28901
Q58519	2190
Q5E1W7	312309
Q5F9Q4	242231
Q5FCG3	254945
Q5FF25	302409
Q5FKR4	1579
Q5GSB8	292805
Q5HCE3	254945
Q5HSQ5	195099
Q5KVP4	1462
Q5L6P2	83555
Q5LC04	272559
Q5LX66	89184
Q5NNL4	542
Q5P2V2	76114
Q5P9B8	234826
Q5QUS6	135577
Q5WDU2	66692

P63893	158878
P63894	158879
P63895	1313
P63896	171101
P72024	1773
P94844	210
Q01R26	234267
Q02FR3	208963
Q03CW4	321967
Q03HT3	278197
Q03M29	322159
Q03YE1	203120
Q04FS0	203123
Q04JJ1	373153
Q04U79	355277
Q053P2	355276
Q057Y0	372461
Q05FS6	387662
Q07UT2	316055
Q086I8	318167
Q0A7E5	187272
Q0AER1	335283
Q0AKB5	394221
Q0AXH2	335541
Q0BIB8	339670
Q0BPE6	391165
Q0C526	228405
Q0HLM3	60480
Q0HY04	60481
Q0I3N1	205914
Q0K6F4	381666
Q0K705	381666
Q0RDT3	326424
Q0S1S5	101510
Q0SRS4	289380
Q0T8G3	373384
Q0TLW0	362663
Q0TP54	195103
Q0VST4	393595
Q0W676	351160
Q10P67	39947
Q11CB4	266779
Q11RZ8	269798
Q122Q1	296591
Q12QJ4	318161
Q12ZG1	259564
Q5WZH5	297245
Q5X822	297246
Q5YT14	37329
Q5ZYK6	272624
Q60AJ4	414
Q62EZ3	13373
Q632C7	288681
Q63YH7	28450
Q64SY7	817
Q65FD6	279010
Q65RL9	221988
Q66FZ5	633
Q67SX4	2734
Q68WW4	785
Q6A8Z4	1747
Q6AE05	59736
Q6AR62	84980
Q6CZG8	29471
Q6F950	62977
Q6FYC7	803
Q6G1L7	38323
Q6LLN5	74109
Q6ME33	264201
Q6MRN0	959
Q6ND59	1076
Q6NGR6	1717
Q71Y02	265669
Q72AX3	882
Q72YE7	222523
Q73FW5	163164
Q73W21	1770
Q7M7Q4	844
Q7MQC1	196600
Q7MYN5	141679
Q7NV18	536
Q7VFH5	32025
Q7VPN0	730
Q7VRM5	203907
Q7VUL2	520
Q7W203	519
Q7WQY1	518
Q816D1	226900
Q81XR2	1392
Q824C6	83557
Q82KD0	33903
Q82U84	915

Q13E66	316057	Q838I3	1351
Q13U79	266265	Q83AD4	777
Q15TQ4	342610	Q83PH3	623
Q16CE5	375451	Q87DI4	183190
Q17XF1	382638	Q87KJ4	670
Q17ZX1	272563	Q88CF3	160488
Q17ZX4	272563	Q88GD4	160488
Q18K16	362976	Q88V90	1590
Q1AZV2	266117	Q89X45	375
Q1B3R6	164756	Q8AAB7	818
Q1B609	164756	Q8D2T4	36870
Q1B655	164756	Q8DD81	672
Q1B6I9	164756	Q8ENX2	182710
Q1BA69	164756	Q8FBN6	217992
Q1BZ73	331271	Q8FPE1	152794
Q1C0I8	360102	Q8FYF0	29461
Q1CMU5	377628	Q8G7Z7	216816
Q1CU21	357544	Q8KAX9	1097
Q1CZV9	246197	Q8NP73	1718
Q1GKJ6	292414	Q8TY71	2320
Q1GPH0	117207	Q8UC03	176299
Q1H392	265072	Q8Y344	305
Q1IF57	384676	Q8Y5N9	1639
Q1INQ7	204669	Q8YJF0	29459
Q1LIE4	266264	Q929Z7	1642
Q1LJ29	266264	Q92I41	781
Q1MEY4	216596	Q92L46	382
Q1MMY3	216596	Q93H44	33903
Q1MRS7	363253	Q97FV2	1488
Q1QET4	335284	Q98EB6	381
Q1QRT8	323097	Q9A280	155892
Q1QSX7	290398	Q9K7F0	86665
Q1RGH0	364106	Q9KVL6	666
Q1RHE5	336407	Q9PD98	2371
Q1WU20	362948	Q9PJW2	83560
Q21CH6	316056	Q9PMD8	197
Q21H39	203122	Q9X1L0	2336
Q220P8	338969	Q9Z833	83558
Q24QU8	138119	Q9ZDB7	782
Q255G3	264202	Q9ZLR5	85963
Q28K08	290400	A0NX08	384765
Q2FNQ9	323259	A0Y9E2	247633
Q2GCF7	279238	A0Z3C4	247639
Q2GH22	205920	A1EP63	345076
Q2IGU5	290397	A1F6L5	412614
Q2IGX0	290397	A1ZUT8	313606

Q2J314	316058	A2P8C0	412966
Q2J737	106370	A2PW90	412883
Q2K721	347834	A2TUS9	313590
Q2KDU1	347834	A2U473	313598
Q2KW85	360910	A2VL64	348776
Q2LTA0	56780	A2VUK2	350702
Q2N7Y2	314225	A2W2B9	350702
Q2NHW0	339860	A2W6M9	350701
Q2NVY2	343509	A3EMM0	345075
Q2P1T1	342109	A3EPU4	419542
Q2RJL2	264732	A3GPX0	417399
Q2RY36	269796	A3GZK8	417400
Q2S3M0	309807	A3HUK5	388413
Q2SMM6	349521	A3J518	391598
Q2SZ94	271848	A3JWF3	388401
Q2YBT6	323848	A3K5H0	388399
Q2YJN7	359391	A3L1Y6	350704
Q2YXX3	273036	A3LIJ5	350703
Q30PD7	326298	A3RPS1	342110
Q30ZK7	207559	A3SA00	52598
Q31HA5	317025	A3SII1	89187
Q326J2	300268	A3SUW3	314267
Q32K66	300267	A3UXP4	314291
Q39JM8	269483	A3V2L6	314232
Q39TG7	269799	A3VGT7	314271
Q39Z66	269799	A3VZH0	314264
Q3A1U6	338963	A3W9K3	237727
Q3ACY8	246194	A3WKF2	314276
Q3APL9	340177	A3WTJ1	314253
Q3B619	319225	A3X6Z2	314262
Q3BUC6	316273	A3XMA0	398720
Q3IKQ7	326442	A3Y2W5	314290
Q3ISP9	348780	A3YDJ7	314277
Q3IYU4	272943	A3YJM4	360111
Q3J7E0	323261	A3YSC4	360108
Q3JNM7	320372	A3ZCN1	360112
Q3KI98	205922	A3ZGD1	360110
Q3KLZ2	315277	A4A9F2	314285
Q3SJS2	292415	A4AHJ7	312284
Q3SW72	323098	A4AQ09	313603
Q3YRP2	269484	A4B9F1	314283
Q3Z5X9	300269	A4BTZ5	314278
Q3Z7V5	243164	A4C2N1	313594
Q3ZXV5	255470	A4EGP7	391593
Q46DC3	269797	A4F0J3	388739
Q46WS0	264198	A4KK04	395095

Q46XD7	264198	A4LTV7	425067
Q47HI9	159087	A4MWY3	374927
Q47RU3	269800	A4N2Y7	375432
Q47YI6	167879	A4N7F2	375177
Q48E64	264730	A4NF18	374928
Q493S0	291272	A4NPD8	374933
Q49XJ8	342451	A4NWV8	375063
Q4FNQ1	198252	A4TUU7	55518
Q4FVQ6	259536	A5F4I6	345073
Q4JV62	306537	A5J6J2	334802
Q4KIG9	220664	A5KEL2	398000
Q4L6A1	279808	A5L4A6	391574
Q4QKM7	281310	A5PE46	161528
Q4UKE7	42862	A5TPJ6	370895
Q4UU71	314565	A5XSH3	334803
Q4ZNP9	205918	A5ZC46	411901
Q576R4	235	A6A4H1	417398
Q57865	2190	A6AF63	417397
Q57TJ7	28901	A6AWH1	410291
Q5E7N0	312309	A6B528	419109
Q5F5Y7	242231	A6BJ94	411462
Q5FFT0	302409	A6BX28	412420
Q5FKQ8	1579	A6D6V2	391591
Q5FSK8	442	A6DCU0	391592
Q5GTA6	292805	A6E6D0	391613
Q5HAV4	254945	A6EAU6	391596
Q5HG24	93062	A6ESV5	50743
Q5HPE6	176279	A6F901	58051
Q5HWX1	195099	A6FK80	351016
Q5L5G4	83555	A6GU48	391597
Q5LIV1	272559	A6NZA1	411467
Q5LLT7	89184	A6XZR8	404974
Q5M154	299768	A6Y5Y9	345074
Q5M5P2	264199	A7A8W1	411481
Q5NPM9	542	A7AFG2	411477
Q5P1G6	76114	A7B0M4	411470
Q5PA93	234826	A7GVX7	360105
Q5QXL5	135577	A7I0N9	360107
Q5V5D5	2238	A7M0Y1	411476
Q5X090	297245	A7V2U2	411479
Q5X8U7	297246	A7VVM6	428125
Q5YSW2	37329	A8E8B9	360118
Q5ZZ80	272624	A8RQV0	411902
Q607A7	414	A8RQV5	411902
Q62H17	13373	A8S7U5	411485
Q63QT3	28450	A8TB81	314289

Q64ZZ5	817
Q65I50	279010
Q65TY2	221988
Q66ER9	633
Q67P64	2734
Q68XM0	785
Q6A5I1	1747
Q6A7P7	1747
Q6ADZ0	59736
Q6AR64	84980
Q6D0C7	29471
Q6F6R2	62977
Q6FZ13	803
Q6G2G3	38323
Q6G9G5	282459
Q6GH12	282458
Q6LUK7	74109
Q6LYR5	39152
Q6MDD8	264201
Q6MRM8	959
Q6NCX8	1076
Q6NGP2	1717
Q72BM6	882
Q73I13	163164
Q73VY3	1770
Q74GT5	35554
Q7MA63	844
Q7MNU2	196600
Q7MTF9	837
Q7N8W3	141679
Q7NX34	536
Q7TXX0	1765
Q7UJD7	265606
Q7VIW7	32025
Q7VLM6	730
Q7VQK8	203907
Q7W510	519
Q7WGH5	518
Q81SU0	1392
Q822G7	83557
Q82K82	33903
Q82WP9	915
Q83SQ9	623
Q84CP9	216595
Q87EC0	183190
Q87SF5	670
A8TYW8	331869
A8UBP7	333990
A8UN00	391603
A8UU47	392423
A9CU05	411684
A9CX84	314608
A9DYX0	391587
A9DZY2	391624
A9F9Y7	383629
A9G5C0	391619
A9HHQ8	391595
A9K039	412021
A9V9Z1	81824
A9ZC25	373665
A9ZZQ3	404214
B0ARN4	486624
B0G8B3	411461
B0GKM8	404218
B0GUK6	404217
B0H953	404216
B0HL25	404215
B0HZE4	360099
B0NTM7	449673
B0PBH6	445972
B0Q4G0	486619
B0QKD3	486621
B1B991	445337
B1BJ14	445334
B1BT63	451755
B1ERF6	502347
B1EZP7	486623
B1FII7	396596
B1G3K4	396598
B1GF36	486620
B1HH03	320374
B1QDT3	445335
B1QQT9	445336
B1QW39	447214
B1R8W4	451754
B1RI72	451756
B1RKY9	451757
B1S6P6	473819
B1TAX6	396597
B1URC5	486622
B1V3U2	488537
B2EB16	486409

Q88DU4	160488
Q88W01	1590
Q891S2	1513
Q89WK2	375
Q8A2I5	818
Q8CP95	176280
Q8D3H6	36870
Q8DEM0	672
Q8EHS7	70863
Q8F133	173
Q8FLB4	217992
Q8FV07	29461
Q8G526	216816
Q8KBD8	1097
Q8NWS4	196620
Q8PXL6	2209
Q8RBI6	119072
Q8RQN0	152794
Q8THP0	2214
Q8TVG7	2320
Q8UIV8	176299
Q8XJ55	1502
Q8XVT2	305
Q8YDC8	29459
Q8ZIL6	632
Q92J79	781
Q97GI8	1488
Q9A2L1	155892
Q9CFC0	1360
Q9EZ11	93061
Q9KC93	86665
Q9KPH7	666
Q9PEC3	2371
Q9PIT2	197
Q9PK30	83560
Q9X1K8	2336
Q9X6Y9	520
Q9Z6L2	83558
Q9ZE14	782
Q9ZLW6	85963
A0NJU3	379360
A0NUK6	384765
A0PTZ4	362242
A0PVI6	362242
A0Q9Q2	243243
A0QMW6	243243
B2H2N6	331109
B2N5E6	344610
B2NTP2	444451
B2P8D9	444452
B2PK61	444453
B2Q4N0	471874
B3A0Z3	478004
B3ALM4	478005
B3B7J6	478006
B3BGA0	478008
B3C0S4	478007
B3C8K4	471870
B3ESH9	452471
B3HAU8	340184
B3HY99	340197
B3IAW8	340185
B3IH21	340186
B3J6N0	405536
B3JK41	470145
B3RRW0	10228
B3TCQ1	455614
B3WQU6	344601
B3X3W8	358708
B3XGF8	358709
B3XRK0	349123
B3YFL6	439842
B3YQY6	405917
B3ZBK0	451707
B3ZLH7	451709
B4ABF7	454168
B4AG10	536229
B4BSF6	495036
B4CW33	497964
B4FQ56	4577
B4VD84	465541
B4W5R9	391600
B4WYX4	236097
B5C656	439846
B5CH97	454165
B5CZ46	484018
B5G787	465543
B5GY97	443255
B5HF74	457429
B5J0B4	391626
B5JD45	382464
B5JX79	391615

A0R3T2	246196	B5JYL7	391616
A0Y0B3	156578	B5MTH3	439847
A0YHG2	247633	B5N4A4	440534
A0Z1I8	247639	B5NHM7	454167
A1EM33	345076	B5NVU7	454231
A1F959	412614	B5P8H4	454164
A1KH58	410289	B5PM52	465518
A1KHJ7	410289	B5PXL6	465516
A1T1R5	350058	B5Q5I6	465517
A1T9F6	350058	B5QIF0	478547
A1TBL5	350058	B5QQ70	486408
A1TBZ7	350058	B5S7L4	305
A1TE67	350058	B5SE92	564066
A1TF02	350058	B5UPC0	405533
A1TGK4	350058	B5V7M9	451708
A1UGJ2	189918	B5WAE8	516466
A1UIY1	189918	B5WTK7	516466
A1UJ27	189918	B6AMF7	419541
A1UKS3	189918	B6AUD3	314270
A1UMY4	189918	B6B533	439496
A1ZZK8	313606	B6BRV9	439493
A2BMP4	415426	B6C3A3	473788
A2PCP0	412966	B6FV69	500632
A2PT94	412883	B6R1X8	439495
A2RPW1	964	B6W4A1	483217
A2TT27	313590	B6XD12	520999
A2U0H9	313598	B6XT80	566552
A2UYC1	399804	B6Y6Q3	569881
A2VGM3	348776	B6ZPT7	502346
A2VGY7	348776	B7AKB0	483216
A2VLA9	348776	B7BAG5	537006
A2VSY6	350702	B7CYA6	557724
A2WCQ1	350701	B7DN20	543302
A3CQD6	388919	B7QTH2	439497
A3ELC6	345075	B7RE50	443254
A3F361	535026	B7RMJ4	391589
A3F364	1423	B7WTE4	399795
A3F373	1423	B8KD33	391586
A3F382	535024	B8KKH4	566466
A3GSX1	417399	B8KSV1	565045
A3H2G9	417400	B8L8U4	391601
A3HZF5	388413	B9AG14	483214
A3J4Z3	391598	B9BW48	513052
A3J979	270374	B9CI10	513053
A3JWE2	388401	B9NV10	467661
A3K3W9	388399	B9TEQ5	3988

A3L3B7	350704	B9XTX9	544405
A3LK00	350703	B9Y073	544406
A3Q045	164757	B9Y074	544406
A3Q1Z9	164757	C0AA30	278957
A3Q474	164757	C0ARX0	471881
A3Q4T9	164757	C0ARX1	471881
A3Q7B8	164757	C0B4X8	470146
A3RS10	342110	C0BSH9	547043
A3S8T3	52598	C0CXE0	518636
A3SKM6	89187	C0CXE3	518636
A3STP6	314267	C0DT95	546274
A3TMH7	313589	C0E2L5	566549
A3TW10	252305	C0EAG3	537013
A3UWR3	314291	C0EJP0	546264
A3V318	314232	C0F9X3	77037
A3VCD0	314271	C0G7W2	595497
A3VZ71	314264	C0VID7	525244
A3WFG8	237727	C0VW53	548477
A3WPM2	314276	C0WB46	563191
A3WSF7	314253	C0WRR2	525318
A3XDM2	314262	C0WYX2	525325
A3XPF8	398720	C0X6H4	491074
A3Y0Q6	314290	C0XH29	525327
A3YCC7	314277	C0YF85	595498
A3YKG9	360111	C0YJ58	525257
A3YQD5	360108	C0Z0S0	585517
A3ZF37	360112	C1HTK1	469598
A3ZJS5	360110	C1HW98	528345
A3ZUG3	314230	C1I657	457396
A4AHH7	312284	C1MG58	469595
A4AST4	313603	C1NGC4	457400
A4BK75	314283	C1V496	469382
A4BRX7	314278	C2BP22	525264
A4BZ80	313594	C2BYY0	525367
A4C5M4	87626	C2C565	592313
A4EN49	391593	C2CP13	525268
A4EZX4	388739	C2D2Q2	525310
A4F3T8	36809	C2DI06	525278
A4KFM8	395095	C2DY94	525281
A4KFZ7	395095	C2E687	525330
A4KK47	395095	C2EFD5	525364
A4MF59	425067	C2EMK8	525365
A4MZT9	374927	C2EST0	525366
A4N3U8	375432	C2EWS1	491077
A4NAL3	375177	C2F3R9	548485
A4NFE5	374928	C2FAU3	525337

A4NIP8	374932
A4NRC1	374933
A4NZ18	375063
A4T2H3	350054
A4T3W9	350054
A4T633	350054
A4T760	350054
A4T9E6	350054
A4TAK7	350054
A4ZH46	326425
A5A9S4	1774
A5F5N9	345073
A5J930	334802
A5KGI3	398000
A5KKP0	411460
A5L5S6	391574
A5LCW4	406556
A5LL42	406557
A5M083	406559
A5M957	406560
A5MHW6	406561
A5MN42	406562
A5MW91	406563
A5PCR7	161528
A5U0W6	419947
A5U1A5	419947
A5WKU8	336982
A5WL81	336982
A5XV22	334803
A5ZAT1	411463
A5ZN97	411459
A6A3C1	417398
A6ABP3	417397
A6ASX9	410291
A6B462	419109
A6BFW0	411462
A6BHQ4	411462
A6BV25	412420
A6CFA6	344747
A6CRX4	161544
A6D9G0	391591
A6DB77	391592
A6E2I3	391613
A6EIN7	391596
A6EPG2	50743
A6EYL6	443152
C2FHM5	525338
C2FRQ3	525372
C2GI63	548478
C2GQI0	525341
C2GW60	548480
C2H0B3	525271
C2HNU2	525306
C2HT65	593585
C2I1U0	593586
C2IED7	593589
C2IX91	593590
C2IYH6	417400
C2JI59	593587
C2JLI4	491075
C2JTL3	525361
C2KC66	491076
C2KX10	585501
C2LKM8	525369
C2M7N1	553178
C2MSM1	526973
C2N8G3	526980
C2NPR6	526970
C2P5N7	526967
C2PM65	526971
C2Q2P9	526972
C2QJ47	526968
C2R062	526977
C2RF53	526969
C2RUY9	526974
C2SAU1	526975
C2SS20	526976
C2T7W0	526978
C2TNL3	526979
C2U4F4	526981
C2UKZ2	526982
C2V2E8	526983
C2VIT0	526984
C2W0V8	526985
C2W0V9	526985
C2WDT1	526986
C2WUK9	526987
C2X3P9	526988
C2XIL4	526989
C2Y131	526990
C2YHB2	526991
C2YYE9	526992

A6F9K6	58051	C2ZEN3	526993
A6FKV8	351016	C2ZW81	526994
A6GNF8	391597	C3ACN8	526997
A6NV43	411467	C3ASE3	526998
A6XUL7	404974	C3B9Q0	526999
A7A7F2	411481	C3BRU0	527000
A7A9V9	411477	C3C9T3	527024
A7B1V2	411470	C3CR54	527021
A7B7M1	411470	C3D900	527025
A7BD67	411466	C3DS60	527026
A7BR58	422289	C3EAD1	527027
A7JF58	430558	C3ESG4	527023
A7JP24	442346	C3F8Z3	527022
A7JWF3	272629	C3FSD1	527031
A7V0E9	411479	C3GA10	527032
A7VCL6	411489	C3GQU9	527029
A7VSZ0	428125	C3H847	527030
A7VX18	428125	C3HQL9	527028
A8CF58	3711	C3I856	527019
A8EID3	360118	C3IR60	527020
A8PKP3	59196	C3IYB8	550542
A8RUZ5	411902	C3JKA7	596309
A8S283	411902	C3PYN3	457395
A8SFA7	411485	C3QJF8	556258
A8STN5	411474	C3QYL4	469590
A8U063	331869	C3R4Z3	556260
A8UAQ6	333990	C3SKE2	562
A8UKJ9	391603	C3X748	556268
A8USA6	392423	C3XD32	556269
A8UZ01	392423	C3XKU6	556267
A8ZYU5	96561	C4B3I0	320390
A9BHL2	403833	C4EXB2	521005
A9CWD4	411684	C4F4B0	521004
A9DBS9	314608	C4FJG4	432331
A9DXZ5	391624	C4FN33	546273
A9EAW0	391587	C4HF22	547046
A9FJ29	383629	C4IL55	632245
A9GNQ6	391619	C4ITD0	641140
A9HI76	391595	C4MCM5	326425
A9LB44	412021	C4PMT9	672161
A9QPC7	481448	C4RDF5	219305
A9Z8C6	373665	C4RIV3	219305
B0A2J9	404214	C4S2H1	349968
B0AM96	486624	C4SGC0	349967
B0G382	411461	C4SRG5	349966
B0GJR8	404218	C4T339	349965

B0GWX0	404217
B0H8K4	404216
B0HGU0	404215
B0HRV7	360099
B0MPQ9	428128
B0MSX7	445970
B0N3V7	445974
B0NA59	411468
B0NJJ9	411468
B0NSI4	449673
B0NXE5	411484
B0P8I0	445972
B0PB22	445972
B0Q264	486619
B0QG82	486621
B0QUF1	456298
B1BIV7	445334
B1BYB3	451755
B1EMN6	502347
B1EYJ8	486623
B1FRY1	396596
B1G7K4	396598
B1GFK7	486620
B1MFF8	561007
B1ML57	561007
B1N232	320374
B1Q6R7	445335
B1QA86	445335
B1QLY0	445336
B1QNF4	445336
B1QXK0	447214
B1R338	451754
B1RHN9	451756
B1RL47	451757
B1S567	473819
B1TGU3	396597
B1UMV1	486622
B1V7F7	488537
B2DYV6	453365
B2E862	486409
B2EAC2	486409
B2H0F3	331109
B2HEG2	216594
B2HLC3	216594
B2HLF2	216594
B2HLV4	216594
C4TXV1	527012
C4UCW3	527002
C4UN76	527005
C4UTB7	527004
C4VI64	553209
C4WJY3	641118
C4YZ75	444612
C5EDZ9	537937
C5EHK8	457421
C5F630	537973
C5NJD9	436115
C5PKV0	525373
C5RFH7	573061
C5RXV2	573062
C5SIP8	573065
C5TBF1	573060
C5TGG8	555217
C5TP14	596320
C5TVE1	573059
C5URZ8	536233
C5VCL9	553207
C5VT09	592027
C5WB43	511693
C5ZH24	357347
C5ZXM8	537970
C6EG05	469008
C6HWR9	412449
C6ICR7	457392
C6IH46	469586
C6JEJ3	457412
C6L106	77133
C6M1B8	547045
C6MFM8	153948
C6MY21	658187
C6NUR4	637389
C6PNU2	536227
C6PNU3	536227
C6PQU3	536227
C6PQU4	536227
C6QFI0	582899
C6QQE5	581103
C6R2G0	553201
C6RAE2	553206
C6RQS2	596318
C6RTZ2	661513
C6SAH3	663926

B2HMD7	216594
B2HT97	216594
B2KCB5	445932
B2ND55	344610
B2NWW9	444451
B2PBP0	444452
B2PMF6	444453
B2Q6T3	471874
B3AAC5	478004
B3AI81	478005
B3B4R2	478006
B3BBT4	478008
B3C2M5	478007
B3CA29	471870
B3HIN7	340184
B3HXV7	340197
B3ICA4	340185
B3IQL2	340186
B3JAR6	405536
B3JK81	470145
B3WSK2	344601
B3X584	358708
B3XJF1	358709
B3XRK6	349123
B3YKF9	439842
B4A3S6	454168
B4AKL2	536229
B4ARI8	545422
B4CW36	497964
B4FH75	4577
B4FRB7	4577
B4VF10	465541
B4W6A6	391600
B4X4P6	236097
B5BW65	439846
B5C8J1	454165
B5CL60	471875
B5CNW7	471875
B5CTW3	484018
B5GIM9	465543
B5H1S8	443255
B5HE40	457429
B5HEU6	457429
B5I6U8	463191
B5IZ69	391626
B5JGR1	382464
C6TQH5	320371
C6YLU3	345072
C7CPR6	565636
C7CWX9	565637
C7DDL7	633131
C7H523	411483
C7JR95	634453
C7K0G2	634454
C7K378	634455
C7KCE7	634456
C7KLR3	634457
C7KW28	634458
C7L5X3	634459
C7U4H1	565638
C7UD17	565642
C7UK90	565641
C7UT59	565650
C7V155	565640
C7V8H8	565644
C7VHZ3	565646
C7VR34	565649
C7VYT0	565647
C7WCX2	565648
C7WGV5	565643
C7WSL0	565651
C7WVF4	565645
C7XE84	563193
C7XGF7	575595
C7Y2H1	575596
C7Y8S6	565639
C8JRM3	393124
C8K6X9	393125
C8KBV5	393128
C8N6K0	638300
C8NPE5	196164
C8PV24	553217
C8QCW6	592316
C8RTL6	525262
C8RWV3	371731
C8SSI8	536019
C8T0I1	667127
C9D144	644076
C9KW45	483215
C9LFU7	626522
C9MEM8	656913
C9MFE2	656912

B5JRY5	391615
B5K5N7	391616
B5MLA1	439847
B5MV26	440534
B5N7S8	454167
B5NM76	454231
B5P5Y1	454164
B5PI04	465518
B5PVD9	465516
B5Q2H5	465517
B5QI72	478547
B5QQ64	486408
B5RVS6	305
B5SI80	564066
B5WHW2	516466
B6AUZ5	314270
B6BEZ8	439496
B6BI09	439483
B6BRK7	439493
B6BWT4	314607
B6C561	473788
B6FRG5	500632
B6FY63	500633
B6FY65	500633
B6R1W0	439495
B6TBG3	4577
B6TNS3	4577
B6VXM3	483217
B6WT30	411464
B6XA20	520999
B6XFS4	520999
B6XVB3	566552
B6Y899	569881
B6ZSJ1	502346
B7AM63	483216
B7AS81	483218
B7B5Y2	537006
B7CLK9	557724
B7DPU3	543302
B7EB68	39947
B7ESW8	39947
B7QSY9	439497
B7R6L7	391606
B7RE48	443254
B7RKS3	391589
B7S1Z6	247634
C9MWZ2	634994
C9NBC7	591167
C9NLU7	675814
C9P0S0	675813
C9PAI5	675811
C9PM03	667128
C9Q2S5	675810
C9QBS4	675816
C9QWE3	536056
C9T3T3	520459
C9TD19	520460
C9TP23	520463
C9TVS2	520461
C9U641	520454
C9UFB6	520452
C9UPK4	520451
C9UUJ6	520450
C9VC44	520456
C9VIG1	520457
C9VWA1	520455
C9X0F3	604162
C9XAF4	568708
C9Y7F4	667019
D0AY82	575591
D0B3I0	224914
D0BEK7	520488
D0BWM7	575564
D0CBH2	575584
D0CRZ0	644107
D0D311	501479
D0DEW3	575597
D0DRF8	575599
D0GCX0	520465
D0GLI4	596323
D0GWF8	675806
D0H1B0	675807
D0HAY3	675820
D0HKW3	675808
D0HVR7	675809
D0I356	675812
D0IMG8	675815
D0ISR4	290847
D0JJK3	637382
D0JTM9	637385
D0JZV0	684950
D0P9D6	520489

B7S2E4	247634
B7X378	399795
B8AGP3	39946
B8AK10	39946
B8GKG4	521011
B8KAG8	391586
B8KWU8	565045
B8KYP4	391601
B8LM55	3332
B9AEG3	483214
B9B993	513051
B9BXI4	513052
B9CJL4	513053
B9CKR7	553184
B9CRV4	553212
B9CYY0	553218
B9HGA9	3694
B9I4G3	3694
B9NTS8	467661
B9R006	244592
B9XSF7	320771
B9XWD0	544405
B9Y1E8	544406
B9Z4Q3	279714
C0AZ29	471881
C0BAM7	470146
C0BJJ0	487796
C0BRV7	547043
C0BYQ6	553973
C0BZL9	553973
C0CJV1	476272
C0D118	518636
C0D1J1	518636
C0DX90	546274
C0EF07	537013
C0ERH7	546264
C0EZF8	411469
C0FAC7	77037
C0FNL5	622312
C0N5R5	637616
C0VIV4	525244
C0VLV4	525244
C0VVI4	548477
C0W7E1	525246
C0WKP0	525260
C0WRR8	525318
D0PM18	520487
D0RKA5	437701
D0S155	575585
D0SBU7	575586
D0SK96	575587
D0SWR1	575588
D0T4W5	575589
D0TIE1	469589
D0TNY7	469588
D0W4N6	546262
D0W824	546265
D0WTN0	674977
D0XB20	673519
D0XJ63	633149
D0ZNS4	588858
D1A0H3	406984
D1CU47	520449
D1D4K7	528346
D1DBI9	528352
D1DHW1	528354
D1DN03	528356
D1DUT3	528355
D1E1B7	528358
D1E7P4	528359
D1EE37	528360
D1ENY7	520462
D1F082	520464
D1F565	520466
D1FGD9	520458
D1JPE9	469587
D1JXU5	457391
D1KD54	655186
D1P2X2	500637
D1PHW6	537011
D1Q3G8	547047
D1Q5T5	545431
D1RI90	638315
D1RNA9	682634
D1RXY0	682634
D1SAF7	644283
D1T0B5	643561
D1U0N8	687916
D1UBQ7	643562
D1UFK3	640510
D1WVM8	649189
D1XHE5	647653

C0WYW6	525325
C0XH35	525327
C0XSF8	525263
C0Y6N2	595498
C0Z0S6	585517
C1ALR0	561275
C1AM50	561275
C1B278	632772
C1B4F2	632772
C1DCP4	557598
C1HPI3	469598
C1I0A7	528345
C1I600	457396
C1MCE0	469595
C1MYX7	564608
C1NBX5	457400
C1PF16	345219
C1V499	469382
C2BNZ6	525264
C2CAF6	592313
C2CNY4	525268
C2D2Q9	525310
C2DN61	525281
C2E693	525330
C2E969	525362
C2EF47	525364
C2EML4	525365
C2ESS3	525366
C2EWS7	491077
C2F3S5	548485
C2FAU9	525337
C2FIE4	525338
C2FRS2	525372
C2GC84	548473
C2GIA2	548478
C2GQH4	525341
C2GTG2	548480
C2GUU9	548480
C2HNU8	525306
C2HW00	593585
C2I523	593586
C2IJD5	593589
C2IRW5	593590
C2J789	417400
C2JFL3	593587
C2JTM1	525361
D1YCN5	679194
D1YPZ9	686660
D2ABY2	591020
D2F274	585543
D2LAK6	644968
D2LCK1	648757
D2M248	649639
D2MFM5	652103
D2MUQ9	683082
D2MWB4	683083
D2NDM9	431946
D2TCR7	644651
D2TWV3	638
D2YCP4	671074
D2YT21	671076
D2Z026	58340
D2ZMD0	500639
D2ZNL3	521002
D2ZUS1	546266
D3A2V0	546268
D3C679	648999
D3D4N2	102897
D3FP06	567106
D3H421	216592
D3LQU2	596312
D3MCM1	679195
D3MKJ6	686659
D3MV28	547146
D3N760	640512
D3R6B5	552531
D3UUP7	634464
D4BE39	500640
D4BND6	518634
D4C3Z6	521000
D4CDB6	411486
D4CP29	608534
D4DPB0	546263
D4E035	667129
D4EPC3	699185
D4EYP1	699186
D4F9V4	500638
D4FGQ7	679188
D4G0X5	645657
D4JYF3	718252
D4KBN1	657322
D4MER1	657310

C2KC72	491076
C2KKV2	586220
C2KNJ1	585199
C2LNI8	525369
C2LTS7	596322
C2M0I3	629742
C2M1X7	553178
C2PTS2	526972
C2Q9R8	526968
C2SHM6	526976
C2TV30	526981
C2V9K0	526984
C2XRR9	526990
C2Z5N7	526993
C2ZMA3	526994
C3A3T0	526997
C3AJQ0	526998
C3JKI5	596309
C3JMU0	596309
C3Q4J5	457395
C3REC7	556260
C3RLL3	556270
C3TRE2	562
C3X6F0	556268
C3XCM0	556269
C3XFZ3	613026
C3XJV5	556267
C4AP03	320390
C4F1W1	521005
C4F5Q9	521004
C4FDZ3	518635
C4G654	592010
C4G893	626523
C4GHW4	629741
C4H1M7	547047
C4HJB1	547046
C4IIZ5	632245
C4IWK6	641140
C4PMM0	672161
C4RD81	219305
C4RWG3	349968
C4SDA1	349967
C4SIW2	349966
C4T7L0	349965
C4T9G8	573
C4U0R7	527012
D4MSP7	245012
D4PN22	393117
D4PV74	393131
D4Q330	401650
D4SBJ5	585531
D4SQ79	427081
D4SUH7	427081
D4T8Z4	427082
D4V0E7	791166
D4V9C3	702446
D4VH65	702447
D4WM68	702443
D4WYW5	702444
D4X4W0	742159
D4XSH0	707232
D4Y9C0	634956
D4YMF1	585530
D4YRH5	585524
D5AX04	449216
D5D586	714962
D5H923	761659
D5MIL0	671143
D5N1M4	703612
D5NM52	243261
D5NUH2	649754
D5PJL6	525368
D5PMK7	525370
D5Q255	525259
D5QCE4	714995
D5QPX3	595536
D5R8I5	642492
D5RHB4	525371
D5RX59	525258
D5SCN1	762948
D5TI87	573236
D5W033	758678
D5XX56	515617
D5Y7K3	520141
D5YKV6	520140
D5YUJ7	515616
D5Z6Q4	537209
D5ZJQ4	537210
D5ZT11	566461
D6AS89	457431
D6BA75	457425
D6CV77	426114

C4U9J1	527002	D6D4K7	657309
C4UL35	527005	D6DBA2	722911
C4UXT2	527004	D6DEQ4	717608
C4V3Y9	638302	D6DP58	718254
C4WAV6	596319	D6EP80	457428
C4WGN3	641118	D6FJU9	611303
C4X337	484021	D6FND2	611304
C4ZKG5	85643	D6GCP5	469608
C5E842	537937	D6GP91	469608
C5EAS9	537937	D6HB90	528348
C5EJK6	457421	D6I365	550672
C5EU99	457421	D6IG44	550676
C5EYX2	537972	D6IWP4	656380
C5F624	537973	D6JHC4	550677
C5N5F6	450394	D6JNA0	528351
C5N7J2	436115	D6JSY3	575565
C5NUT9	546270	D6K3D1	645465
C5PKT1	525373	D6KJW7	457416
C5Q053	548474	D6KQD6	450749
C5Q931	525374	D6KXY5	641147
C5QJ27	548475	D6LQF3	520448
C5QSD0	525378	D6MCC9	536227
C5QX06	525376	D6SLZ9	555779
C5RCN1	585506	D6UJP4	585535
C5RQP3	573061	D6V142	666684
C5RTE4	573062	D6VHS1	596153
C5RTN9	573062	D6XAV5	463191
C5S265	637911	D6XPG0	637913
C5TCL1	573060	D6YDJ5	707184
C5TEJ6	555217	D6YFN9	707185
C5TKI0	596320	D6YIA6	707186
C5TW33	573059	D6YKY1	707187
C5UA40	509193	D6YMP3	707183
C5UBF3	509193	D6YYB8	718219
C5URK7	536233	D7CA85	749414
C5V752	395494	D7DDQ2	759363
C5VCP5	553207	D7DH27	759364
C5VGE5	553174	D7EV68	515615
C5VTQ5	592027	D7H046	520453
C5W1Z3	511693	D7HSL1	412967
C5ZCF4	357347	D7I6G6	693985
C5ZY79	537970	D7II93	469585
C6A8N2	580050	D7IVX5	469592
C6AJ90	555970	D7J762	585544
C6DVN8	478434	D7JGK6	575590
C6DWG4	478434	D7JVR8	656379

C6EB22	469008
C6I2T4	457392
C6IIM9	469586
C6JE57	457412
C6LB89	478749
C6M3K8	547045
C6MHN9	153948
C6MVY7	443143
C6MWF2	443143
C6N249	658187
C6PQF8	536227
C6PQL1	536227
C6PUK4	536227
C6PZ64	536227
C6QFG9	582899
C6QSG9	581103
C6R2H6	553201
C6RAG6	553206
C6RHQ9	553219
C6RJC8	596318
C6S2K9	661513
C6SCI1	663926
C6SHG4	295996
C6TYA7	320371
C6YG47	345072
C6YTA1	539329
C6YZI3	457394
C6ZDI8	1288
C7DDA5	633131
C7GAJ2	536231
C7H7M8	411483
C7HHP5	572545
C7HIS3	572545
C7HL89	588857
C7HN48	588857
C7IFT9	588581
C7IM98	588581
C7IQ80	589861
C7JK68	634453
C7JUD6	634454
C7K4M8	634455
C7KDU7	634456
C7KN63	634457
C7KXH8	634458
C7L7A2	634459
C7SZQ3	668607
D7K9E1	457390
D7N1D2	641149
O05322	294
Q05KS1	1396
Q08NY3	378806
Q0FDI0	367336
Q0FIU2	314265
Q0G537	314231
Q1N419	207949
Q1NGC0	314266
Q1NP11	262489
Q1NRT7	262489
Q1NYP8	374675
Q1V113	314261
Q1V618	314288
Q1VXI7	313595
Q1YDS2	287752
Q1YSU8	314287
Q1YY73	314280
Q1ZDC8	314282
Q1ZLA3	314292
Q26EQ0	156586
Q28VM7	290400
Q29XU8	197
Q2C2Q2	121723
Q2CK90	314256
Q2FSY3	323259
Q2J775	106370
Q2PYI1	360423
Q3EZF0	339854
Q3RBF8	155920
Q3RHE4	155919
Q40JB0	332415
Q4ED36	307502
Q4EHD0	267410
Q4EQE1	267409
Q4HDX9	306254
Q4HQC0	306264
Q4MR97	269801
Q540H4	1718
Q6J694	158899
Q7PBT8	272951

C7XFL4	563193
C7XGG3	575595
C7XU16	575594
C7Y2G5	575596
C7ZX04	585143
C8A3U1	585145
C8ABS0	585146
C8AKT0	585153
C8ART6	585158
C8KPH0	452948
C8KW82	455227
C8KWV7	591023
C8L4M3	553565
C8LCL5	553567
C8LK17	553568
C8LVE6	553571
C8M343	553573
C8M623	553581
C8ME60	553583
C8MKP3	553588
C8MVR3	553592
C8N5H0	553596
C8N7S6	638300
C8NPH3	196164
C8NU10	585529
C8P351	525280
C8PF81	553220
C8PZ29	553217
C8QA86	592316
C8RTN9	525262
C8RXG0	371731
C8SQA9	536019
C8SQI4	536019
C8TBJ5	667127
C9CZ14	644076
C9KK12	500635
C9L413	537007
C9LH89	626522
C9LRG0	546271
C9M0D3	585520
C9MCR2	656913
C9MJN2	656912
C9MP29	649761
C9MUA8	634994
C9NH53	591167
C9NNS1	675814

C9P2D0	675813
C9P9W0	675811
C9PQP8	667128
C9PX53	619693
C9Q6I9	675810
C9QMS8	675816
C9QSC1	536056
C9T2F1	520459
C9TBN9	520460
C9TIQ9	520463
C9TZT3	520461
C9U8J9	520454
C9UH88	520452
C9URX6	520451
C9V3D9	520450
C9V4G3	520456
C9VGJ4	520457
C9VMR8	520455
C9X289	604162
C9X5J9	568708
C9YDD2	667019
D0AUT7	575591
D0B887	224914
D0BFN3	520488
D0C441	575564
D0CFA0	575584
D0CTV5	644107
D0D6L8	501479
D0DEW9	575597
D0DRF2	575599
D0G9Q1	520465
D0GLW1	596323
D0GQU3	675806
D0H536	675807
D0HHB1	675820
D0HSJ7	675808
D0HV70	675809
D0I805	675812
D0IM13	675815
D0ISW9	290847
D0JEJ3	637382
D0JNN1	637385
D0K002	684950
D0L3Q3	526226
D0L8V6	526226
D0P814	520489
D0PHB9	520487
D0RFL8	437701
D0RQL3	684719

D0RSX4	469609
D0S564	575585
D0SFF0	575586
D0SP42	575587
D0SXE0	575588
D0T811	575589
D0TJG8	469589
D0W2C5	546262
D0W9Z5	546265
D0WHJ1	649764
D0WR09	649743
D0X0B6	674977
D0X7E6	673519
D0YQH1	596328
D0YZY4	675817
D0ZJN3	588858
D0ZYX1	406984
D1CZN5	520449
D1D676	528346
D1DE05	528352
D1DKF3	528354
D1DS40	528356
D1DYK3	528355
D1E524	528358
D1E624	528359
D1EHW5	528360
D1EK63	520462
D1EVK8	520464
D1F2J5	520466
D1FB55	520458
D1GU93	663951
D1JQH6	469587
D1K2J2	457391
D1NHD1	456482
D1NIS0	492476
D1NJ17	492476
D1NTB8	561180
D1P1P1	500637
D1P6N5	500637
D1PGA2	537011
D1PTF5	585502
D1Q548	545431
D1QDL7	553594
D1QLW5	553601
D1QRV9	649760
D1QX78	553574
D1RDQ3	638315
D1RWW9	682634
D1SA92	644283
D1SVD2	643561
D1TUL0	687916
D1UAC0	643562
D1USU6	640510
D1VB83	298654

D1VCU6	298654
D1VJZ2	298654
D1VYZ2	679189
D1W2X5	679190
D1WNM5	596317
D1X905	649189
D1XUY4	647653
D1XWE6	553171
D1Y1I3	352165
D1Y8U0	679194
D1YDS2	679194
D2AHR2	591020
D2AYE0	479432
D2EWN3	585543
D2F6V4	585149
D2FD00	585151
D2FLT1	585152
D2FV85	585159
D2G1Q7	585160
D2GAK6	585161
D2GG37	585148
D2GRE6	585150
D2L1J5	644968
D2LAR6	648757
D2LTT8	649639
D2ME05	652103
D2MR57	683082
D2N0L2	683083
D2N723	523796
D2NAY5	431946
D2T4F4	644651
D2TW83	638
D2UM17	585155
D2US14	585147
D2Y997	671074
D2YMI1	671076
D2Z5Z0	469381
D2Z9P7	500639
D2ZQ44	521002
D2ZWP3	546266
D3AE01	566550
D3BWB2	653733
D3CGF9	648999
D3CVP2	102897
D3D7B0	102897
D3EWD8	703339
D3FLI0	567106
D3H3B7	216592
D3HX29	575611
D3I2Q2	575612
D3IC55	575614
D3ILZ8	575615
D3L8Q7	655225
D3LMN9	596312

D3M5R5	656024
D3MCS6	679195
D3MGI5	679195
D3MIC4	686659
D3MP45	686659
D3MW04	547146
D3N408	640512
D3NKY5	663278
D3R5W5	552531
D3R6I7	552531
D3UUH8	634464
D4BF18	500640
D4BMW2	518634
D4C1H7	521000
D4CA90	411486
D4CSC8	546275
D4DMS8	546263
D4DXB7	667129
D4EBN8	694569
D4F213	500638
D4FGR3	679188
D4FIU9	525375
D4FY15	645657
D4J0K0	657324
D4JC30	717962
D4JM27	657317
D4JSV0	657319
D4K4E2	718252
D4K6B8	657322
D4KDR2	657316
D4KNP3	657315
D4KVB5	718255
D4L874	657321
D4LEM1	213810
D4LKN0	657323
D4LQA1	657314
D4LZ16	657313
D4M7J4	651822
D4MMF8	717961
D4MPE7	245012
D4MZG3	245018
D4S161	511680
D4S502	585503
D4SCH2	585531
D4SHL4	585531
D4SHS1	585531
D4SRM6	427081
D4T8N0	427082
D4U0J4	649742
D4UAC8	553590
D4UEW4	553580
D4V3P4	702446
D4X888	742159
D4XKU2	707232

D4XL90	707232
D4Y2C7	634956
D4YII0	655812
D4YMC1	585530
D4YRI2	585524
D5AW69	449216
D5CUW1	714962
D5H845	761659
D5HHC7	751585
D5N528	703612
D5NJR0	243261
D5NUE3	649754
D5P798	525368
D5P9C8	525368
D5P9F2	525368
D5PAK0	525368
D5PAP6	525368
D5PEZ9	525368
D5PHA0	525368
D5PJG5	525368
D5PJG8	525368
D5PMQ6	525370
D5PZY0	525259
D5Q7Q8	525259
D5Q7R1	525259
D5QVG7	595536
D5R627	642492
D5RUA6	525371
D5S0P1	525258
D5S0P4	525258
D5S433	525258
D5SCL6	762948
D5THU6	573236
D5TIE9	573236
D5VS27	573063
D5VV85	758678
D5VVU9	758678
D5XRM6	515617
D5XS18	515617
D5XXA6	515617
D5Y1Q4	520141
D5Y235	520141
D5Y7P9	520141
D5YDA3	520140
D5YDP3	520140
D5YI74	520140
D5YPM8	515616
D5YQ33	515616
D5YUP5	515616
D5Z1G7	537209
D5Z1V6	537209
D5Z6V2	537209
D5ZDZ8	537210
D5ZEE8	537210

D5ZJV4	537210
D5ZT56	566461
D6AS38	457431
D6BA11	457425
D6BCX0	556264
D6CVM2	426114
D6D8S9	722911
D6D9P5	722911
D6DIN9	717608
D6DTB7	718254
D6E155	657318
D6E7P8	657308
D6EQ56	457428
D6F2K9	611302
D6F304	611302
D6FJZ8	611303
D6FNI0	611304
D6FP32	611304
D6FQ95	611304
D6GMQ3	469608
D6GYT5	585156
D6H593	528348
D6HGP6	585144
D6I4S7	550672
D6IHZ1	550676
D6IJT9	656380
D6J059	585157
D6J6F8	550677
D6JJH6	528351
D6JW73	575565
D6K2U9	645465
D6L242	641147
D6LTV7	520448
D6LXP0	585154
D6SH15	548470
D6SLT8	555779
D6T4M8	553577
D6UAM2	762962
D6UJV4	585535
D6V1M7	666684
D6VK41	596153
D6XP95	637913
D6YDC8	707184
D6YFH1	707185
D6YI37	707186
D6YKR2	707187
D6YMH6	707183
D6YY48	718219
D6ZU23	759350
D7ADC9	663917
D7CAD0	749414
D7DDI6	759363
D7DGW1	759364
D7EP09	515615

D7EPE8	515615
D7EVB2	515615
D7H611	520453
D7HEG2	345074
D7HQ58	412967
D7I3X9	693985
D7IHJ1	469585
D7IWY7	469592
D7JHJ4	656379
D7LKV2	81972
D7LWI7	81972
D7N4B4	641149
D7NDI3	563008
O05918	1773
O29621	2234
O53407	1773
O80574	3702
P46829	1765
Q00UJ4	70448
Q094T5	378806
Q09C55	378806
Q0EZ45	314345
Q0G5X7	314231
Q0RIJ9	326424
Q0S194	101510
Q0SDQ1	101510
Q0YUC5	377431
Q188N4	272563
Q1B266	164756
Q1B4A6	164756
Q1B8G5	164756
Q1D3D3	246197
Q1K3A7	281689
Q1N387	207949
Q1NAI0	314266
Q1NP14	262489
Q1NRT9	262489
Q1PWY2	174633
Q1V1C4	314261
Q1V754	314288
Q1W0A9	313595
Q1YF36	287752
Q1YSX3	314287
Q1Z376	314280
Q1ZHJ8	314282
Q1ZMQ6	314292
Q26DM1	156586
Q2BRA6	207954
Q2C7U9	121723
Q2CKF6	314256
Q3L886	246196
Q3R6G7	155920
Q3RBB1	155920
Q3RDT8	155919

Q4ECH2	307502
Q4HGG1	306254
Q4HQ53	306264
Q5NVU9	115547
Q5YW71	37329
Q5YWK8	37329
Q5YWX6	37329
Q5Z0L8	37329
Q67W29	39947
Q6VAY4	91504
Q6VRJ8	210
Q73TU9	1770
Q73TZ2	1770
Q73VY0	1770
Q73XS2	1770
Q73YE3	1770
Q742G5	1770
Q744J2	1770
Q744K9	1770
Q76D21	1590
Q76E50	17
Q7D8X3	1773
Q7PAT9	272951
Q7U0R7	1765
Q7U105	1765
Q8G614	216816
Q8LB01	3702
Q8RAI4	119072

**diaminopimelate decarboxylase**		**aromatic amino acid aminotransferase I**		**aromatic amino acid aminotransferase II**	
**UniProt**	**TXID**	**UniProt**	**TXID**	**UniProt**	**TXID**
Q949X7	3702	P53090	4932	P38840	4932
D7L343	81972	D6VTV2	559292	D3DL86	559292
Q6ZG77	39947	C8Z853	643680	B3LSQ1	285006
B7ED21	39947	C7GWZ3	574961	C8Z9T2	643680
A9SFK1	145481	B3LHQ0	285006	A6ZT45	307796
A9U0R2	145481	A6ZTY7	307796	C7GTW4	574961
A8HNC0	3055	A7TT91	436907	Q6FT20	5478
C3ZAT5	7739	Q6FTM8	5478	A7TKJ5	436907
C1DTF1	204536	C5DT73	559307	C5DZL2	559307
C6HWT2	412449	C5DLN0	559295	Q6CR82	28985
Q3A1U8	338963	Q6CKK3	28985	C5DCW2	559295
B5YL42	289376	Q752M6	33169	B9WGK8	573826
Q1N418	207949	Q6BYU5	4959	A5DQ29	4929
Q2IGX9	290397	A3LMX7	4924	A3GF91	4924
Q1AZX2	266117	C4XXW0	306902	A3GGR2	4924
D3PAV1	639282	B9WA90	573826	C5MDV9	294747
D4H4C0	522772	C4YJ02	5476	C4YGT4	5476
B2V8C1	436114	Q5ADA2	5476	Q5A472	5476
B8JAN6	455488	Q5ACW9	5476	Q5A4C4	5476

B4UHY2	447217	A5DV12	36914	C4Y8G5	306902
Q2BRG1	207954	C5M5E9	294747	A5E7Q6	36914
B6AKF9	419541	A5DFM7	4929	C4R6Z7	644223
A1TXX6	351348	C4QYZ3	644223	Q6CCZ6	4952
D3MWE9	547146	Q6C551	4952	Q9HIY3	2303
D2LC45	648757	Q5AZE2	162425	C1B176	632772
C4FKX0	432331	Q5B339	162425	C1AWU9	632772
A3EQW3	419542	C8V102	227321	D1C6M2	479434
D5MIL1	671143	C8V843	227321	D1C6Z3	479434
A7H772	404589	Q2UFD9	5062	D1CA43	479434
D0LZL7	502025	Q2U485	5062	C6BS59	526222
B4U7U8	380749	A2QD20	425011	C6C265	526222
Q0HQJ1	60481	A2R526	425011	C3JGE5	596309
Q1YSU7	314287	B8NHI9	332952	C3JRF8	596309
Q0HN96	60480	B8NTN9	332952	C0ZWQ5	234621
Q0VM22	393595	C1H4Q3	502779	C0ZTG6	234621
A0KS64	94122	C0SF57	482561	Q125E7	296591
B9M379	316067	C1GK60	502780	Q129Y0	296591
A3Q9P6	323850	A1C7J1	5057	Q124B0	296591
D1U3P2	643562	Q4X0F7	5085	Q129G7	296591
B4WYX0	236097	B0XTG0	451804	Q12A65	296591
C0QR61	123214	A1DHS0	331117	B5VK71	545124
A5GD88	351605	B2WC58	426418		
A6F217	443152	B2WP52	426418		
D3A0Z3	546266	C5JQK8	559298		
A8GZG8	398579	C5GBH3	559297		
B5EGX5	404380	B6Q744	441960		
B0TJ50	458817	C0NBP4	447093		
C6MVY5	443143	B6GY09	500485		
D6KZ89	641147	Q0CY85	341663		
D4X558	742159	A6R4Q9	339724		
A8UQK3	392423	A6S3P7	332648		
C8SU06	536019	B8MRR4	441959		
C3MHU3	394	B8MRR5	441959		
C6M1E7	547045	B8MI34	441959		
Q98G34	381	A7EXH0	665079		
B7V5G8	557722	C5P1D9	222929		
A3LIJ6	350703	C7YK49	660122		
Q02E85	208963	C7ZH25	660122		
A3L1Y7	350704	C7ZNJ6	660122		
A3RW12	342110	C7ZF95	660122		
A6UCI8	366394	A4R0K4	148305		
P19572	287	C9SIQ8	526221		
B8LIU0	39645	Q0UVF5	13684		
B8CHW3	225849	B6K3W8	402676		
A1SAP6	326297	D5GJ27	39416		

A9CFF5	176299	C5FSY7	554155
D7N292	641149	D4CYX5	663202
A3JE41	270374	D4AYN7	663331
C5CQG1	543728	C4JJL9	336963
A6QBZ0	387093	Q7S0F9	5141
D3A1S9	546268	O14192	4896
A9M0Q2	374833	Q9Y7S6	4896
A6WXL4	439375	O94570	4896
Q2L205	360910	C6H9Z4	544712
Q2LTA2	56780	Q2HDU3	38033
B8DJI8	883	Q5KEQ7	5207
Q92MG9	382	Q55XW3	5207
Q8XV52	305	Q5KM31	5207
B5RWS9	305	B2AY10	5145
Q74GT7	35554	D1ZQT4	5147
D7ADC7	663917	Q5M8X7	8364
D0WD05	546265	Q28I58	8364
C5TLQ1	596320	D3B4I4	670386
B5SJG8	564066	B0CY75	486041
Q5F538	242231	Q6PB16	8355
D6JIV7	528351	Q6IR98	8355
D6H9R0	528348	Q54K00	44689
D1ECU0	528360	Q86AG8	44689
D1E681	528359	Q5E9N4	9913
D1E011	528358	Q8N5Z0	9606
D1DT35	528355	Q4W5N8	9606
D1DLQ3	528356	Q9WVM8	10090
D1DAB0	528352	Q3UNS8	10090
D1D6C7	528346	B7STY2	80604
B2UEH9	402626	Q64602	10116
C6S4U0	662598	A8NRI2	240176
D2L1N1	644968	D6RP20	240176
Q21P77	203122	D6RPR8	240176
C0EPQ3	546264	Q2KEC9	242507
C9X2J8	604162	Q4PDK9	5270
C6SDQ9	663926	Q4P8M5	5270
A3YDJ6	314277	D6WQD2	7070
C4WJB4	641118	C1BQK8	217165
B5JSZ4	391615	B8P756	561896
Q1NP10	262489	Q8U4G7	2261
C6SNL9	295996	A2BJX0	415426
B4S2E0	314275	O57946	53953
A1KW56	272831	Q9V282	29292
Q6G1Q7	38323	D3L4X1	592015
A1UQZ1	360095	D3L5H5	592015
B9JTJ4	311402	B6YXH1	523850

A1TKV2	397945	B6YWH1	523850
D1DGM1	528354	A9WLC6	288705
C1HV26	528345	C6A1W9	604354
B4RRA6	521006	C6A3Y8	604354
A6W1B1	400668	A0K277	290399
C7LTT9	525897	A0JVT5	290399
Q1QSV2	290398	D2Z555	469381
Q30ZX1	207559	D2Z554	469381
Q11DW3	266779	D2Z551	469381
D4DVJ8	546263	A7VP07	428125
A0NX43	384765	D1B8E9	525903
A4VGW6	379731	D1B975	525903
D5V5Q5	572480	D1B793	525903
B9QWS7	244592	B8H7W2	452863
C9Y829	667019	C8XE84	479431
D0W418	546262	C8XDZ4	479431
Q88CF4	160488	B5ISJ9	391623
A5WAU4	351746	B5IT16	391623
Q2YBM2	323848	Q5JFM8	311400
Q12S91	318161	B2KBF6	445932
Q2K3P1	347834	D1Y263	352165
B9JBQ7	311403	D1Y271	352165
Q3SUR2	323098	D1Y6G1	352165
A7BZP9	422289	C0CQY1	476272
C0N8U8	637616	C0CJZ7	476272
A9CX82	314608	C0CQB6	476272
Q1ZDC7	314282	C0CQU0	476272
C6BFD8	428406	C0CJ86	476272
B8KD34	391586	A8AAT9	453591
D3SCB8	396595	A1RTC0	384616
B3PPC3	491916	C7INH2	588581
A9D7X2	411684	C7INH3	588581
A0Y9E3	247633	C7ICQ2	588581
A0LEA9	335543	C4KJ65	426118
Q4K3W3	220664	C4KJT5	426118
A6F900	58051	C3MYX7	427317
A6FHR9	58051	C3MRR9	427317
Q1ZLA4	314292	C3N025	427318
Q47JL5	159087	C3N0Q8	427318
D3BWB5	653733	D3LTA0	699218
B5ZRV7	395492	D3LVS5	699218
Q0G118	314231	A0LWW4	351607
Q2C2Q3	121723	A3MWQ9	410359
Q1MB71	216596	D2PE97	425944
C6MEQ0	153948	D2PEY3	425944
D7I6G7	693985	C3N8A8	439386

Q13CL3	316057	C3N8Z4	439386
Q8DD82	672	D5U1G5	633148
C6AXH9	395491	D5U0R0	633148
Q7MQC2	196600	Q9C4M4	2265
Q6FYE6	803	Q4J8Q1	2285
A4XNX5	399739	Q4JA09	2285
A4B9F0	314283	A8MBP7	397948
C9VC92	520456	A8MD57	397948
D5X6D1	75379	B7R0U3	246969
B0CIY6	470137	C3NMS8	419942
A4A9F3	314285	C3NMH5	419942
A0AK64	386043	C5A5P6	593117
A0B1U1	331272	C3MJ85	429572
A0B761	349307	C3MJW8	429572
A0DDH5	5888	A6VI85	426368
A0KC32	331272	Q6M122	39152
A0KFI0	380703	A6TJA6	293826
A0L1I2	94122	A6TSX6	293826
A0LE32	156889	Q9V2W5	49899
A0LSJ2	351607	C0WAF8	563191
A0LZD1	411154	C5EEF6	457421
A0PUL7	362242	C5EEF5	457421
A0PYI3	386415	B9DYW0	583346
A0Q0N7	386415	B9E558	583346
A0Q830	401614	B9E5R4	583346
A0Q8L3	401614	B9E3D4	583346
A0QBX1	243243	A5N5B2	431943
A0QCV9	243243	A5N1C7	431943
A0R5H9	246196	A5N212	431943
A0RBN1	412694	A5MZE0	431943
A0RMP2	360106	D2Q549	479435
A1A3B5	367928	D2PT02	479435
A1AF49	405955	D2PSN8	479435
A1AQI3	338966	C6VLC9	644042
A1AVE2	413404	C6VPE9	644042
A1B1K1	318586	D7K5E5	457390
A1B3L8	318586	A7LYW3	411476
A1BH64	290317	A7LYW4	411476
A1JPF4	393305	D1YCT0	679194
A1KAK8	62928	P95957	2287
A1KBR4	62928	Q97TY7	2287
A1KI81	410289	D7J839	585544
A1R7X1	290340	D7J840	585544
A1RP45	351745	D0KRF4	555311
A1RPD3	351745	D0KR52	555311
A1SHH1	196162	Q88T05	1590

A1SR00	357804	Q88X39	1590
A1TD76	350058	C2FLB9	525338
A1TTR2	397945	C2FJI6	525338
A1UK05	189918	D4WXH3	702444
A1UZS7	320388	D4WXH2	702444
A1V8C7	320388	D4VSS7	702447
A1VB88	391774	D4VSS8	702447
A1VDE1	391774	D0TQ15	469588
A1VK10	365044	D0TQ16	469588
A1VY33	354242	C3QK73	556258
A1W3Y5	232721	C3QK74	556258
A1WS52	391735	D5DH59	592022
A1WWB2	349124	D5DI05	592022
A1WXB7	349124	D5DZF6	545693
A2BRR9	146891	D5DW32	545693
A2BX77	167542	D6D823	657309
A2C3I7	167555	D6D824	657309
A2C8D2	59922	C3R2N5	469590
A2FSX2	5722	C3R2N6	469590
A2RKG8	416870	D4WDF0	702443
A2S1Y0	412022	D4WDF1	702443
A2S673	412022	D1BRV3	446471
A2S7F3	412022		
A2SKI0	420662		
A2SR51	410358		
A3CPR4	388919		
A3CWU2	368407		
A3D9B2	325240		
A3DD89	203119		
A3M805	400667		
A3MAA9	400667		
A3MCQ5	320389		
A3MQ72	320389		
A3MRT1	320389		
A3N2H3	416269		
A3N5F6	320373		
A3NEE1	320373		
A3NGR2	320373		
A3NR54	357348		
A3P075	357348		
A3P2B4	357348		
A3PDJ5	167546		
A3PMC0	349101		
A3Q3D2	164757		
A3UA07	216432		
A3UBR4	216432		

A4CMN0	313596
A4FBI8	405948
A4FN46	405948
A4FWX7	402880
A4G9Q1	204773
A4IQH6	420246
A4IVY8	418136
A4IXJ3	418136
A4J3L1	349161
A4J9J6	349161
A4JPW2	269482
A4JQY9	269482
A4QDE2	340322
A4QFJ9	340322
A4S9T6	436017
A4SE11	290318
A4SRW8	382245
A4SUZ4	312153
A4T8M5	350054
A4TLD6	386656
A4VX35	391295
A4W3D9	391296
A4WE18	399742
A4WVC2	349102
A4X2Q8	369723
A4XAY4	369723
A4XI14	351627
A4Y2U0	319224
A4YBF8	319224
A4Z1S2	114615
A5CQ43	443906
A5CXU3	412965
A5D2V3	370438
A5E9Q7	288000
A5EB52	288000
A5FHY9	376686
A5FRS3	216389
A5FWI2	349163
A5GLZ4	32051
A5GS34	316278
A5I760	441771
A5IM66	390874
A5INZ4	359786
A5IST2	359786
A5N5T1	431943
A5U1Z2	419947

A5UDZ8	374930
A5UHM9	374931
A5UMZ8	420247
A5UQD4	357808
A5V6E2	392499
A5VA98	392499
A5VDK8	392499
A5VJ55	557436
A5VSV3	444178
A5W6M7	351746
A5WCB2	349106
A5WLX4	336982
A6L6Z6	435590
A6L7I0	435590
A6LDJ1	435591
A6LIK7	435591
A6LLV3	391009
A6M2R4	290402
A6Q1Z2	387092
A6Q2A3	387092
A6QDA7	426430
A6QGV1	426430
A6T3G4	375286
A6TDH9	272620
A6TW43	293826
A6TXQ8	359787
A6U1M1	359787
A6UP95	406327
A6UPK0	406327
A6UTH7	419665
A6V5X0	381754
A6VE51	381754
A6VGE0	426368
A6VKS1	339671
A6VWU5	400668
A6W7F0	266940
A6WTF6	402882
A7FFC8	349747
A7FYT2	441770
A7GIS2	441772
A7GMV8	315749
A7H086	360105
A7H561	360109
A7HK57	381764
A7HTI6	402881
A7I2W1	360107

A7IA50	456442
A7IG42	78245
A7JXK4	150340
A7K5A1	150340
A7MR54	290339
A7N0W1	338187
A7N9W3	458234
A7NEL4	458234
A7NI38	383372
A7WX79	418127
A7X278	418127
A7Z683	326423
A7ZCF1	360104
A7ZQU4	331111
A8A3X2	331112
A8AP60	290338
A8EWM7	367737
A8F6N9	416591
A8FES1	315750
A8FKA3	407148
A8G0W1	425104
A8G5G0	93060
A8GIJ2	399741
A8I031	438753
A8KYH2	298653
A8KYM6	298653
A8LL43	398580
A8M094	391037
A8M2L5	391037
A8M854	391037
A8MC62	397948
A8YUT0	405566
A8YZ48	451516
A8Z3X8	451516
A8Z5Z1	444179
A8ZUA7	96561
A9A0C7	96561
A9AAB3	444158
A9B4A8	316274
A9BB15	93059
A9BFP3	403833
A9BUZ8	398578
A9G338	448385
A9GY99	448385
A9HRI0	272568
A9IHQ0	340100

A9IZG4	382640
A9KSP5	357809
A9M970	483179
A9N3I2	272994
A9N485	272994
A9NGC5	441768
A9R2S4	349746
A9VLI7	315730
A9W3N0	419610
A9W7P2	419610
A9WER2	324602
A9WMT0	288705
A9WNA8	288705
B0BRB8	434271
B0CAX9	329726
B0JI64	449447
B0K665	399726
B0K776	340099
B0KG72	76869
B0KQ40	76869
B0RAP9	31964
B0RPV3	509169
B0SGT1	355278
B0SPX6	456481
B0T2T9	366602
B0TFB9	498761
B0TWJ6	484022
B0TX75	484022
B0UCU0	426117
B0UWI0	228400
B0VDN3	509173
B0VT29	509170
B1GYN3	471821
B1HRM7	444177
B1I3Y4	477974
B1I8T0	487214
B1IFH3	498213
B1IU06	481805
B1J1W1	390235
B1JQB8	502800
B1K7Y1	406425
B1K8I0	406425
B1KBM2	406425
B1KQC9	392500
B1L216	498214
B1L5D9	374847

B1LBQ7	126740
B1LR36	439855
B1M0A5	426355
B1MKF2	561007
B1MLU3	561007
B1MZN1	349519
B1VFW4	504474
B1VSP8	455632
B1W0C3	455632
B1W3K3	455632
B1WWH7	43989
B1XDP4	316385
B1XLF3	32049
B1XST4	452638
B1Y312	395495
B1YJ44	262543
B1YYI9	398577
B1Z7G6	441620
B1ZHE8	441620
B1ZTL0	452637
B2A6F2	457570
B2G6M6	557433
B2GC08	334390
B2GLW9	378753
B2HR80	216594
B2HX83	405416
B2IB09	395963
B2IM60	516950
B2ITZ1	63737
B2J2S8	63737
B2JI28	391038
B2JZ78	502801
B2K220	502801
B2KCK2	445932
B2RGX4	431947
B2S8P0	430066
B2SEU5	441952
B2SJ64	360094
B2T014	398527
B2T713	398527
B2TA75	398527
B2TQL5	508765
B2TYQ3	344609
B2UQ22	349741
B2USC1	512562
B2UZA5	508767

B2VFT7	338565
B3DPJ2	205913
B3DUY0	481448
B3E926	398767
B3EDH7	290315
B3EPY7	331678
B3GYK6	537457
B3PEI9	498211
B3QCB0	395960
B3QMQ0	517417
B3QT24	517418
B3QUA3	517418
B3R7C8	164546
B3W7E8	543734
B4EFE4	216591
B4EUH0	529507
B4EUS9	529507
B4FT32	4577
B4RFG7	450851
B4S8Y2	290512
B4SAJ9	324925
B4SZN2	423368
B4T507	423368
B4TBM9	454169
B4TGR9	454169
B4TQ96	439843
B4TUN3	439843
B5BCI6	554290
B5BFH9	554290
B5E2G9	512566
B5EIG1	404380
B5ERL5	380394
B5EZP4	454166
B5F4V8	454166
B5FCR1	388396
B5FPK4	439851
B5FUC3	439851
B5QWU5	550537
B5R341	550537
B5RCI5	550538
B5RDY9	550538
B5XUN5	507522
B5YEX1	309799
B5Z4F6	444450
B5ZA56	563041
B5ZM03	272568

B6EP84	316275
B6I6W3	409438
B6ISK3	414684
B6IYR6	414684
B6JDJ0	504832
B6JKL9	570508
B6YR99	511995
B7G3A2	556484
B7GKH5	491915
B7GNL9	391904
B7GY70	557600
B7HHH4	405532
B7HKE3	405534
B7I5Y6	480119
B7ICP3	484019
B7INB2	405531
B7JBF7	243159
B7JG04	405535
B7K1S5	41431
B7KH89	65393
B7KX28	440085
B7KX96	440085
B7LF15	585055
B7LP52	585054
B7LY94	585034
B7MM44	585035
B7MZD8	585397
B7N770	585056
B7NVY4	585057
B7UHQ6	574521
B7VMD5	575788
B7VSB3	575788
B8BT13	296543
B8CGU1	225849
B8CW02	373903
B8D7W8	561501
B8D9L6	563178
B8DBX4	552536
B8DPQ8	883
B8DWQ9	442563
B8E0J4	515635
B8E691	407976
B8EML9	395965
B8F6T0	557723
B8FA18	439235
B8FH33	439235

B8FLY1	439235
B8G0D4	272564
B8G8Y4	326427
B8GI83	521011
B8GT85	396588
B8GY54	565050
B8HB08	452863
B8HLE2	395961
B8I674	394503
B8I697	394503
B8IHN5	460265
B8IQX0	460265
B8J168	525146
B8ZNY2	561276
B8ZR25	561304
B9DP04	396513
B9DTR7	218495
B9DZB2	583346
B9EC01	458233
B9H0T6	3694
B9HRK5	3694
B9IV09	361100
B9JI17	311403
B9K1Q3	311402
B9K869	309803
B9KDN9	306263
B9KLS3	557760
B9L103	309801
B9L907	598659
B9LA44	598659
B9LJM6	480224
B9LUB5	416348
B9M393	316067
B9MDE3	535289
B9MQB3	521460
B9RH82	3988
C0PXK1	476213
C0Q004	476213
C0QAL6	177437
C0QL78	177437
C0QL89	177437
C0QPS5	123214
C0QZY6	565034
C0RFM6	546272
C0ZC72	358681
C0ZHU8	358681

C1A1T4	234621
C1A1Z6	234621
C1A3W9	379066
C1AMT7	561275
C1AW16	632772
C1CA22	488221
C1CGQ8	488222
C1CMS4	488223
C1CTN2	487213
C1CVI7	546414
C1CZG9	546414
C1D9B8	557598
C1DJ55	322710
C1DMY9	322710
C1EMR6	572264
C1F969	240015
C1FM02	536232
C3K435	216595
C3KUV0	515621
C3L9N8	568206
C3LPW7	579112
C3NUA5	593588
C3P514	592021
C3PFN9	548476
C3PKL9	548476
C4K7C4	572265
C4KPG7	536230
C4KWT6	536230
C4L2D7	360911
C4LDU9	595494
C4LJN1	645127
C4WYS1	484021
C4XS91	573370
C4XS98	573370
C4Z038	515620
C4Z1W2	515620
C4Z8Y0	515619
C4ZGX3	515619
C4ZZZ1	595496
C5A6G0	593117
C5A9J5	626418
C5AZ53	272630
C5B0Z8	272630
C5B900	634503
C5BUL5	377629
C5C1S2	471853

C5CA61	465515
C5CGJ8	521045
C5D3N5	471223
C5VZH0	218494
C5YS29	4558
C6A9G7	580050
C6AAX5	634504
C6AFK9	555970
C6AK40	634176
C6BUL1	526222
C6C6L3	579405
C6CA89	579405
C6CCR1	579405
C6CG08	561229
C6CKW3	561229
C6CMX9	561229
C6CU63	324057
C6DE66	561230
C6DGF6	561230
C6DU77	478434
C6E4B6	443144
C6E9R0	443144
C6GPR0	568813
C6GW44	568814
C6SNY5	511691
C6UCW4	413997
C6USF2	544404
C6VQ64	644042
C6W5G4	471854
C6W8P3	446462
C6WHL5	446462
C6WTN8	583345
C6WZM6	531844
C6XB67	582744
C6XHV1	537021
C6XLN3	582402
C6XYQ4	485917
C7BI15	553480
C7BKN6	553480
C7BYN0	592205
C7C8B6	661410
C7CDU6	661410
C7JFI9	634452
C7LEL2	568815
C7LK87	595499
C7M180	525909

C7M3Q4	521097
C7MDU4	446465
C7MLU2	469378
C7MX58	471857
C7N472	471855
C7N753	471855
C7NAR6	523794
C7NIU6	478801
C7NQC6	519442
C7P2F6	485914
C7P7Q9	573064
C7PA05	485918
C7PRL3	485918
C7Q1P8	479433
C7Q1Q2	479433
C7Q567	479433
C7Q626	479433
C7QQZ6	395962
C7QZD7	471856
C7R3M4	471856
C7R6Q1	523791
C7RKU0	522306
C7RTW3	522306
C7T8R4	568703
C7TFF9	568704
C8TG88	573235
C8UAI7	585395
C8UF47	585396
C8VZ05	485916
C8WA07	521095
C8WE60	622759
C8WJL6	479437
C8WK38	479437
C8WTN4	521098
C8X2H8	485915
C8XGU3	479431
C8XIA2	479431
C9R2T9	668336
C9R7Y2	429009
C9RI02	579137
C9RRE8	59374
C9RZX8	544556
C9XJR8	645462
C9XSS6	645462
C9XZL8	413502
C9YM99	645463

C9YN01	645463
C9YUF6	680198
C9YZE3	680198
C9Z012	680198
C9Z332	680198
C9Z8G9	680198
D0FVK6	79967
D0J5X7	688245
D0J9B3	600809
D0JB04	331104
D0K4X5	681288
D0K7A6	681288
D0KLN2	561231
D0KW89	555778
D0L1T4	555778
D0L9G7	526226
D0R4B7	633699
D0Z8R0	498217
D0ZDW5	498217
D0ZF68	498217
D1A870	471852
D1AE43	471852
D1ANN0	526218
D1B0D3	525898
D1BCT2	446469
D1BMV6	479436
D1BWD6	446471
D1C1P8	479434
D1C814	479434
D1CFP5	525904
D1YWV9	304371
D2ARU9	479432
D2ATF5	479432
D2B0K1	479432
D2BFH3	479432
D2BH29	311424
D2BR53	684738
D2BVW0	590409
D2C146	590409
D2C348	590409
D2C3S3	590168
D2NU16	680646
D2NVD5	653938
D2P6R0	637381
D2Q3G0	479435
D2Q6E3	401473

D2QCI1	504472
D2R0H9	530564
D2RI67	572546
D2RLN7	591001
D2RX75	543526
D2SET1	526225
D2TJI4	637910
D2UCF6	380358
D3DFE6	608538
D3DYT4	634498
D3E7E4	481743
D3ENK8	713887
D3F242	469383
D3FZU0	398511
D3H6T3	365659
D3HFI6	637909
D3P5L0	137722
D3PPF4	504728
D3PWV4	446470
D3PXR2	446470
D3QCY1	698737
D3QQG9	701177
D3RHR6	640131
D3RU77	572477
D3RV77	572477
D3RWW3	589924
D3S4Y4	644281
D3SEW3	396595
D3SI33	633145
D3SL32	638303
D3SZN6	547559
D3T0U5	547559
D3T474	580331
D3UJ93	679897
D3UPQ7	683837
D3V470	406818
D3VK37	406817
D4GLN6	706191
D4GW22	309800
D4H5F8	522772
D4HE67	553199
D4HWD8	665029
D4IA97	716540
D4Z152	452662
D4Z519	452662
D4ZCR4	637905

D5AR90	272942
D5B338	637386
D5BBR6	655815
D5BLL2	655815
D5BMR6	488538
D5C3F0	472759
D5C540	472759
D5C994	716541
D5CMA2	580332
D5D8P6	641892
D5DFM9	592022
D5DL71	592022
D5DRL4	545693
D5DS07	545693
D5EB64	547558
D5ECU2	572547
D5EK78	583355
D5EXM0	264731
D5H2T6	748671
D5SXN8	521674
D5T5H8	762051
D5TUC4	714359
D5U5R5	526224
D5ULB6	446466
D5UW53	521096
D5UZ09	521096
D5V852	749219
D5VLD9	509190
D5W5L7	640511
D5WE19	640511
D5WQB9	562970
D5WSY6	562970
D5X7C0	635013
D6XVG1	439292
D6Y6N0	469371
D6Y7F9	469371
D6Z6B5	589865
D6ZBD5	640132
D6ZD99	640132
D6ZDJ1	640132
D6ZPC8	525381
D6ZWL0	759350
D7A9C1	639283
D7AQZ2	583358
D7AVA4	446468
D7BHM1	526227

D7CKT4	643648
D7CXF2	649638
D7D019	691437
D7D496	691437
D7DNH8	666681
D7DS01	456320
D7DXV4	551115
D7EAT6	644295
D7FF72	693745
D7GDE3	754252
O27390	187420
O29458	2234
O67262	63363
P09890	1718
P0A5M4	1773
P0A5M5	1765
P23630	1423
P44316	727
P55709	394
P56129	210
Q025Z2	234267
Q02YQ1	272622
Q03CW0	321967
Q03HS9	278197
Q03MG4	322159
Q03YE5	203120
Q04A09	321956
Q04FS4	203123
Q04IJ1	373153
Q04VQ9	355277
Q04XR2	355276
Q057G6	372461
Q05FG3	387662
Q07H25	316055
Q07XM3	318167
Q0AC73	187272
Q0ACF6	187272
Q0AFA5	335283
Q0AG67	335283
Q0AIY9	335283
Q0AX25	335541
Q0B2E0	339670
Q0B7S4	339670
Q0BK47	393011
Q0BNP8	393011
Q0BVX5	391165

Q0BWK8	228405
Q0I5V6	205914
Q0IBD1	64471
Q0K660	381666
Q0RBY7	326424
Q0RD94	326424
Q0SGN2	101510
Q0SVG1	289380
Q0T126	373384
Q0TDZ4	362663
Q0TTL1	195103
Q0W3B7	351160
Q112C4	203124
Q11PY5	269798
Q121Y1	296591
Q12FF9	296591
Q12Y77	259564
Q13HW4	266265
Q13TK7	266265
Q14K42	393115
Q15EG8	264
Q15NG6	342610
Q169X1	375451
Q17YA6	382638
Q187B7	272563
Q188C1	272563
Q18K14	362976
Q1B532	164756
Q1BIN8	331271
Q1BPS8	331271
Q1CAT1	360102
Q1CFA3	377628
Q1CUL3	357544
Q1CZV7	246197
Q1D9U5	246197
Q1GLJ9	292414
Q1GRT1	117207
Q1GWP5	117207
Q1GYH9	265072
Q1I304	384676
Q1I7P4	384676
Q1IL09	204669
Q1J092	319795
Q1LI78	266264
Q1LPD0	266264
Q1MQX0	363253

Q1QB97	335284
Q1QEA0	335284
Q1QHU3	323097
Q1R7H3	364106
Q1WTC4	362948
Q20WU1	316056
Q21UC6	338969
Q220V2	338969
Q24CD6	312017
Q24MH6	138119
Q28V64	290400
Q2A1E6	376619
Q2A5D5	376619
Q2FYN4	93061
Q2G1M6	93061
Q2G4W2	279238
Q2G5U9	279238
Q2GGY2	205920
Q2IYC6	316058
Q2J1X0	316058
Q2J5N9	106370
Q2J6L4	106370
Q2JL19	321332
Q2JQX7	321327
Q2NA26	314225
Q2NCU4	314225
Q2NE09	339860
Q2NQ25	343509
Q2NRG2	343509
Q2P628	342109
Q2RIR2	264732
Q2RPL5	269796
Q2RXE4	269796
Q2SFZ2	349521
Q2SQ65	349521
Q2SU64	271848
Q2VYN0	342108
Q2Y5L5	323848
Q2YR85	359391
Q2YUV8	273036
Q2YY19	273036
Q30TD2	326298
Q30XY6	207559
Q31AD5	74546
Q31EI4	317025
Q31XE9	300268

Q32C83	300267
Q39M50	269483
Q39U26	269799
Q39Z59	269799
Q3AAR4	246194
Q3AJ54	110662
Q3ARK4	340177
Q3AX85	316279
Q3B374	319225
Q3BQC1	316273
Q3IEW2	326442
Q3IJ29	326442
Q3ISH2	348780
Q3IZX9	272943
Q3J9S7	323261
Q3JEA4	323261
Q3JHE3	320372
Q3JMV2	320372
Q3JWD0	320372
Q3MEQ0	240292
Q3SKV2	292415
Q3SM71	292415
Q3YRS8	269484
Q3YY19	300269
Q3Z921	243164
Q3ZZR5	255470
Q46C06	269797
Q46K29	59920
Q46WI4	264198
Q47DB1	159087
Q47FI8	159087
Q47M64	269800
Q47VN2	167879
Q480D9	167879
Q486N2	167879
Q48C01	264730
Q48I40	264730
Q493E0	291272
Q49XK2	342451
Q4FNK5	198252
Q4FV86	259536
Q4JUI2	306537
Q4KEA8	220664
Q4L6A5	279808
Q4QMG9	281310
Q4UXQ0	314565

Q4ZT98	205918
Q500B7	205918
Q50140	1769
Q55484	1148
Q57AS1	235
Q57KA4	28901
Q57LZ4	28901
Q58497	2190
Q5E1W6	312309
Q5FEQ0	254945
Q5FFJ9	302409
Q5FKR2	1579
Q5FPL4	442
Q5HAZ7	254945
Q5HG20	93062
Q5HJP6	93062
Q5HPE2	176279
Q5HWF7	195099
Q5KXK1	1462
Q5LBE1	272559
Q5LXB8	89184
Q5M1I8	299768
Q5M630	264199
Q5NIN9	119856
Q5P2A8	76114
Q5P5N4	76114
Q5QUS5	135577
Q5V5E3	2238
Q5WH19	66692
Q5Z0Z8	37329
Q60AJ5	414
Q62B72	13373
Q62F85	13373
Q62GA0	13373
Q63DW0	288681
Q63NJ6	28450
Q63Q48	28450
Q63XQ4	28450
Q64RT6	817
Q65HV3	279010
Q65QR9	221988
Q667E8	633
Q66B85	633
Q67NE8	2734
Q6A8A9	1747
Q6AG76	59736

Q6AIZ1	84980
Q6CZN7	29471
Q6D0Z6	29471
Q6F949	62977
Q6G9G1	282459
Q6GD02	282459
Q6GH08	282458
Q6GKI2	282458
Q6L350	263820
Q6LLN4	74109
Q6LXZ3	39152
Q6M3U1	1718
Q6MM66	959
Q6N0M1	1076
Q6NHU5	1717
Q6W1T9	394
Q71Y62	265669
Q728N7	882
Q72BI9	882
Q73B78	222523
Q73X41	1770
Q741V1	1770
Q7A0X5	196620
Q7A2R9	158878
Q7A5P5	158879
Q7A862	158879
Q7CGZ1	632
Q7MAC7	844
Q7MT14	837
Q7N7F9	141679
Q7N8H2	141679
Q7NPE5	33072
Q7NRM3	536
Q7TTP9	265606
Q7U7P5	84588
Q7ULZ0	265606
Q7USP5	265606
Q7V104	59919
Q7V6T5	74547
Q7VBI7	1219
Q7VG17	32025
Q7VPM5	730
Q7VRF4	203907
Q7VT97	520
Q7W2C0	519
Q7WR88	518

Q7WXK3	381666
Q81FZ3	226900
Q81T51	1392
Q825X0	33903
Q82J63	33903
Q82PI6	33903
Q82TE0	915
Q82VG2	915
Q834X3	1351
Q83QB7	623
Q84BW0	87883
Q87FL7	670
Q87KJ3	670
Q88L58	160488
Q88WC9	1590
Q891C4	1513
Q89AC6	224915
Q89UN3	375
Q8A800	818
Q8CP93	176280
Q8DI31	197221
Q8DND9	171101
Q8DSQ3	1309
Q8E9H4	70863
Q8EQ80	182710
Q8F7A1	173
Q8FEA2	217992
Q8FPS1	152794
Q8FYA2	29461
Q8G4V1	216816
Q8KCP2	1097
Q8NNQ3	1718
Q8NYS3	196620
Q8PVS6	2209
Q8R8L4	119072
Q8RD37	119072
Q8RQM6	152794
Q8TSR9	2214
Q8TY23	2320
Q8XMU3	1502
Q8XSP3	305
Q8Y5V3	1639
Q8YJJ9	29459
Q8YST7	103690
Q92A56	1642
Q94A94	3702

Q97AJ0	273116
Q97LF3	1488
Q97NP2	1313
Q99X92	158878
Q9A681	155892
Q9ADB5	1902
Q9CG26	1360
Q9CL23	747
Q9HK20	2303
Q9KCM5	86665
Q9KVL7	666
Q9PII5	197
Q9RTK2	1299
Q9X1K5	2336
Q9X5M1	246196
Q9Z661	542
Q9ZBH5	1902
Q9ZME5	85963
A0JY83	290399
A0NJU7	379360
A0Q8H6	401614
A0XX42	156578
A0Y0E9	156578
A0YPC8	313612
A0Z3C3	247639
A0ZL18	313624
A1EP64	345076
A1F6L6	412614
A1HSP2	401526
A1S2L4	326297
A1SUR8	357804
A1TWR5	351348
A1X4N8	663
A2P8B9	412966
A2PW91	412883
A2TUE7	313590
A2U2G1	313598
A2UWC1	399804
A2UYN6	399804
A2VHI9	348776
A2W1H3	350702
A2W3W5	350702
A2W4V3	350702
A2WFV9	350701
A3D9Q1	325240
A3EML9	345075

A3GPW9	417399
A3GZK9	417400
A3HV93	388413
A3I5M2	388400
A3ITL5	391612
A3J0X7	391598
A3JBA8	270374
A3JRD4	388401
A3K2P4	388399
A3L484	350704
A3LKX9	350703
A3M7U4	400667
A3QIG0	323850
A3RSX0	342110
A3SGH4	52598
A3SLW6	89187
A3SVN1	314267
A3TGG2	313589
A3TV61	252305
A3UL16	314291
A3UQW6	314291
A3UXP5	314291
A3V518	314232
A3VBL3	314271
A3VQK4	314260
A3W4J8	314264
A3W9W4	237727
A3WBU7	237727
A3WKF0	314276
A3WU29	314253
A3WUU9	314253
A3XCL2	314262
A3XMR1	398720
A3Y2X4	314290
A3YJE5	360111
A3YQN3	360108
A3Z1N0	69042
A3Z7Y6	221360
A3ZES6	360112
A3ZJH2	360110
A3ZKV7	314230
A3ZS84	314230
A4AVG8	313603
A4BTZ6	314278
A4C0A8	313594
A4C7U9	87626

A4CFE0	87626
A4CVJ0	59931
A4E7M5	411903
A4EHP2	391593
A4EZ79	388739
A4KGL4	395095
A4KPQ7	412422
A4KT88	412422
A4LJK5	425067
A4LL37	425067
A4LT04	425067
A4MX08	374927
A4MX09	374927
A4N2V8	375432
A4N7C5	375177
A4NEZ3	374928
A4NN88	374932
A4NPB9	374933
A4NWZ0	375063
A4NWZ1	375063
A4U0A0	55518
A4VI90	379731
A4XQF3	399739
A5EB60	288000
A5F4I7	345073
A5J3X1	334802
A5J559	334802
A5J691	334802
A5KGN1	398000
A5KKF4	411460
A5L0U2	391574
A5L4A7	391574
A5LEK1	406556
A5LPK4	406557
A5LTS8	406558
A5M300	406559
A5MAA3	406560
A5MHP7	406561
A5MPG5	406562
A5MWG6	406563
A5P7W8	161528
A5PBB4	161528
A5TIH6	370895
A5TMM0	370895
A5VYU6	351746
A5XKY6	334803

A5XR52	334803
A5XS75	334803
A5Z3D4	411463
A5ZHY8	411901
A5ZY96	411459
A6A4H0	417398
A6AF64	417397
A6B3D2	419109
A6B527	419109
A6BEF6	411462
A6BU78	412420
A6CEG9	344747
A6CMX8	161544
A6D6V1	391591
A6DC22	391592
A6DEC2	391592
A6E0S4	391613
A6EJX5	391596
A6EP02	50743
A6EUN2	443152
A6F5E8	443152
A6FLT1	351016
A6G555	391625
A6GPJ4	391597
A6GSE4	391597
A6P1N2	411467
A6VBN6	381754
A6VXB8	400668
A6WIJ4	402882
A6XZR7	404974
A6Y5Y8	345074
A7A6V4	411481
A7AJE9	411477
A7AZ25	411470
A7JF25	430557
A7JF55	430558
A7JJE0	430558
A7JJS3	430558
A7JNJ3	442346
A7JNY4	442346
A7JP21	442346
A7JQU4	272629
A7LQZ2	411476
A7V9W6	411479
A7VDF4	411489
A7VUY3	428125

A7YRW9	430556
A7YS29	430556
A8E959	360118
A8EIU8	360118
A8EM67	360118
A8EMT0	360118
A8KJ75	331978
A8KPM9	331978
A8KSQ6	331978
A8PN39	59196
A8S4F7	411902
A8SAU4	411485
A8SS00	411474
A8TB82	314289
A8TNR1	331869
A8UCI1	333990
A8UGH0	391603
A8YKN7	267872
A9ANZ8	395019
A9DSM6	391624
A9EQA8	383629
A9GFU0	391619
A9HH37	391595
A9JZU1	412021
A9K0L6	412021
A9K589	412021
A9KYS0	399599
A9L5F6	399599
A9Z747	373665
A9ZVI9	404214
B0AC60	445973
B0AQL1	486624
B0G5A3	411461
B0GFA5	404218
B0GN15	404217
B0H6V3	404216
B0HJ07	404215
B0HVF1	360099
B0KPJ9	76869
B0MEI8	411490
B0MSA7	428128
B0NB28	411468
B0NLI0	449673
B0NXU3	411484
B0P7M2	445972
B0Q2E6	486619

B0QJ43	486621
B0QSI4	456298
B0VEY4	456827
B1B7K6	445337
B1B9R6	445337
B1BEY0	445334
B1BUS5	451755
B1EG20	502347
B1EXL6	486623
B1FER2	396596
B1FHY3	396596
B1G466	396598
B1G8L2	396598
B1GH84	486620
B1HBV6	320374
B1HG54	320374
B1HL52	320374
B1JBH4	390235
B1Q9P7	445335
B1QI20	445336
B1QWR5	447214
B1RB75	451754
B1RJB5	451756
B1RSS7	451757
B1S2U4	453362
B1SAL6	473819
B1SH80	471872
B1TAU8	396597
B1UPQ0	486622
B1V3V1	488537
B1X5I9	39717
B2DJT7	453361
B2DQR1	453363
B2DVI4	453364
B2E229	453365
B2E6Q5	453366
B2ECA2	486409
B2GZP2	331109
B2H1K8	331109
B2HAP8	331109
B2NDG0	344610
B2NTU1	444451
B2P1U0	444452
B2PER0	444453
B2PU27	471874
B3A2K1	478004

B3AJ43	478005
B3AXL3	478006
B3BES0	478008
B3BXU2	478007
B3CB81	471870
B3HAH4	340184
B3HVB2	340197
B3I4K7	340185
B3IRT8	340186
B3J978	405536
B3JMK2	470145
B3WHW3	344601
B3X0S7	358708
B3XK53	358709
B3XRK2	349123
B3YDC1	439842
B3YK29	439842
B3YWZ7	405917
B3ZGE0	451707
B3ZVX8	451709
B4A1X1	454168
B4A7C9	454168
B4ALF6	536229
B4APU6	545422
B4ARI5	545422
B4ASZ2	545422
B4B8Q9	497965
B4BL88	495036
B4EPX7	216591
B4F9R7	4577
B4V2J2	465541
B4V9V1	465541
B4VE07	465541
B4VWB2	118168
B4VYU6	118168
B4WEK0	391600
B4WRA5	91464
B4XMC6	210
B5BZJ9	439846
B5C7E9	439846
B5CB71	454165
B5CER2	454165
B5CR34	471875
B5CZK4	484018
B5GAH7	465543
B5GL73	443255

B5GRM0	443255
B5GTG4	443255
B5GZR7	443255
B5H3F3	443255
B5H987	457429
B5HMS8	463191
B5HX53	463191
B5INA1	180281
B5J0K7	391626
B5JFX5	382464
B5KDN8	391616
B5MIL5	439847
B5MJQ8	439847
B5N2D1	440534
B5N4Q6	440534
B5NBY3	454167
B5NEZ2	454167
B5NKS5	454231
B5NP50	454231
B5P3C5	454164
B5P430	454164
B5PAH5	465518
B5PGB6	465518
B5PTU2	465516
B5PV06	465516
B5Q2B8	465517
B5Q6Z3	465517
B5QGQ6	478547
B5QQ68	486408
B5RXQ9	305
B5RXR0	305
B5S9Z9	305
B5UIE1	405533
B5VAZ7	451708
B5W5Y6	513049
B5W648	513049
B5WSN3	516466
B6B0R6	314270
B6BAW2	439496
B6BME5	439483
B6BRG3	439493
B6BY43	473788
B6C3A1	473788
B6FN26	500632
B6FZT5	500633
B6R679	439495

B6T3H3	4577
B6T9Q7	4577
B6VUM4	483217
B6WRV9	411464
B6XAQ2	520999
B6XSN3	566552
B6ZVW8	502346
B7AGZ2	483216
B7AS94	483218
B7ATX9	483218
B7BDF8	537006
B7BDF9	537006
B7CJK7	557724
B7CS75	557724
B7CXX4	557724
B7DQ73	543302
B7QRY8	439497
B7R4N6	246969
B7R5Y8	391606
B7RE46	443254
B7RJG9	391589
B7S2B5	247634
B7V059	557722
B7WVG8	399795
B8AH36	39946
B8E6G0	407976
B8KKH3	566466
B8KSV0	565045
B8LMH4	3332
B8LRZ6	3332
B9AG13	483214
B9AYY7	513051
B9BS89	513052
B9CE35	513053
B9CKR9	553184
B9CRV8	553212
B9CXD3	591023
B9CYR9	553218
B9F5G8	39947
B9NL48	467661
B9P2H6	93058
B9XQ08	320771
B9XTN7	544405
B9XXR4	544406
B9Z1R7	279714
C0A5W7	278957

C0AXW5	471881
C0AXW6	471881
C0B5E3	470146
C0BJE3	487796
C0BMM9	487797
C0BUZ9	547043
C0C3Y7	553973
C0CHB0	476272
C0E777	566549
C0EJ57	537013
C0ETH0	411469
C0FV71	622312
C0G813	595497
C0GG16	555088
C0PQH1	3332
C0QL45	177437
C0VID6	525244
C0VTF5	548477
C0WES1	563191
C0WFN9	525260
C0WRR4	525318
C0WYX0	525325
C0X595	491074
C0XH31	525327
C0XPG3	525263
C0Y0S4	595498
C0YAH0	595498
C0YE75	595498
C0YLK8	525257
C0Z0S2	585517
C1DQ07	322710
C1E2J1	296587
C1HIR6	469598
C1IBA2	457396
C1MB16	469595
C1N8V1	564608
C1ND51	457400
C1PEU8	345219
C1V497	469382
C2BKB7	525264
C2BQS4	525264
C2BZ29	525367
C2C566	592313
C2CL27	525268
C2CPV8	525268
C2D2Q4	525310

C2DCZ1	525278
C2DXP2	525281
C2E689	525330
C2E916	525362
C2EFS4	525364
C2EML0	525365
C2ESS7	525366
C2EWS3	491077
C2F3S1	548485
C2FAU5	525337
C2FIS8	525338
C2FUS3	525372
C2G765	548473
C2GC80	548473
C2GJ15	548478
C2GQH8	525341
C2GWM6	548480
C2H549	525271
C2HNU4	525306
C2HT64	593585
C2I1U1	593586
C2IED8	593589
C2IX92	593590
C2JI58	593587
C2JK10	491075
C2JTL6	525361
C2KC68	491076
C2KKV6	586220
C2LMI4	525369
C2LMX9	525369
C2LPV4	596322
C2M0I7	629742
C2M4Z7	553178
C2MI83	526973
C2MYB2	526980
C2NF29	526970
C2NWC1	526967
C2PCD9	526971
C2PTF4	526972
C2Q9F6	526968
C2QQG6	526977
C2R5J8	526969
C2RKJ5	526974
C2S188	526975
C2SHR9	526976
C2SYF0	526978

C2TE01	526979
C2TUR7	526981
C2UBC0	526982
C2USU1	526983
C2V989	526984
C2VR48	526985
C2W5T8	526986
C2WJX0	526987
C2WZ98	526988
C2X9A0	526989
C2XRG0	526990
C2Y812	526991
C2YP42	526992
C2Z5C4	526993
C2ZLZ0	526994
C3A3H5	526997
C3AJG4	526998
C3B0U9	526999
C3BHZ5	527000
C3BZS6	527024
C3CG86	527021
C3CZ42	527025
C3DHB0	527026
C3E108	527027
C3EIA1	527023
C3EZ29	527022
C3FHM2	527031
C3G0E7	527032
C3GG94	527029
C3GYH2	527030
C3HFX9	527028
C3HXV7	527019
C3IGX9	527020
C3IYJ7	550542
C3JI44	596309
C3JU72	596309
C3K4N0	216595
C3PUW8	457395
C3QFQ5	556258
C3R1I7	469590
C3R725	556260
C3SWE2	562
C3SWE3	562
C3SWE5	562
C3WCK4	469616
C3X6K1	556268

C3XCS2	556269
C3XP10	556267
C4APP0	320390
C4AUT1	320390
C4B1W1	320390
C4EYS3	521005
C4F487	521004
C4FDJ7	518635
C4FQE6	546273
C4G7A9	592010
C4G9C1	626523
C4GL29	629741
C4H1T9	547047
C4HL01	547046
C4HS89	545431
C4I721	536230
C4I9W1	536230
C4ID32	632245
C4ITI2	641140
C4MCM3	326425
C4RH45	219305
C4RNE3	219305
C4RQ99	219305
C4RZK2	349968
C4SAM9	349967
C4SKL4	349966
C4T0W3	349965
C4U144	527012
C4U9A8	527002
C4UIA2	527005
C4UVM1	527004
C4V4W4	638302
C4VE38	553209
C4WAW0	596319
C4ZLI7	85643
C4ZNI8	85643
C5EDD7	537937
C5EEK2	457421
C5F203	537972
C5F628	537973
C5N0Z7	450394
C5N5G0	450394
C5NAN0	436115
C5NE62	436115
C5NLS6	436115
C5PUM7	525373

C5Q1C2	548474
C5Q935	525374
C5QEI3	548475
C5QJ31	548475
C5QSD5	525378
C5QX10	525376
C5RCN5	585506
C5RHU1	573061
C5RSK1	573062
C5RYI0	637911
C5SPM6	573065
C5T915	573060
C5TGW6	555217
C5TVZ5	573059
C5U105	573059
C5UBB0	509193
C5UWR5	536233
C5V146	395494
C5VDY6	553207
C5VIM1	553174
C5W8E0	511693
C5ZEG4	357347
C5ZJ63	357347
C5ZMH3	357347
C5ZXT6	537970
C6EJ30	469008
C6I6B5	457392
C6ISS0	469586
C6J3Y5	621372
C6JBB7	457412
C6JKY2	469618
C6LBQ5	478749
C6MBL4	153948
C6MBM0	153948
C6MKX0	443143
C6NSY6	637389
C6PB15	580327
C6PNR4	536227
C6PQG3	536227
C6PQG4	536227
C6QHI8	582899
C6QPA6	581103
C6R264	553201
C6R8I6	553206
C6R938	553206
C6RE63	553219
C6RQS3	596318
C6RTZ3	661513
C6TA11	3847
C6TSL8	320371
C6TZY4	320371
C6U7K7	320371
C6YLU2	345072

C6YMH1	543737
C6YR44	543737
C6YTA4	539329
C6YTY0	539329
C6Z1U7	457394
C7CT11	565636
C7D3Q8	565637
C7DCH9	633131
C7G8Y6	536231
C7G8Y7	536231
C7HDU3	572545
C7HKU5	588857
C7IJ27	588581
C7IP24	589861
C7JLT4	634453
C7JW02	634454
C7K694	634455
C7KFG3	634456
C7KPS9	634457
C7KZ44	634458
C7L8W8	634459
C7U7M3	565638
C7UGJ8	565642
C7UNZ2	565641
C7UV60	565650
C7V5H9	565640
C7VCI2	565644
C7VNJ2	565646
C7VVX7	565649
C7W2R5	565647
C7WA47	565648
C7WKJ4	565643
C7WP03	565651
C7X230	565645
C7XCF6	563193
C7XGF9	575595
C7XU12	575594
C7Y2G9	575596
C7YAT7	565639
C7ZTC9	585143
C7ZX08	585143
C8A109	585145
C8A3U5	585145
C8A7E3	585146
C8ABS4	585146
C8AFX4	585153
C8AKT4	585153
C8AP92	585158
C8ARU0	585158
C8JRU0	393124
C8K2E1	393125
C8KBN7	393128
C8KLN0	452948
C8KPH4	452948

C8KUK9	455227
C8KW78	455227
C8L4L9	553565
C8L6Z5	553565
C8LCL1	553567
C8LCT6	553567
C8LFN0	553568
C8LK13	553568
C8LP03	553571
C8LVE2	553571
C8M0R7	553573
C8M347	553573
C8M619	553581
C8M8R4	553581
C8ME64	553583
C8MFT1	553583
C8MIR7	553588
C8MKP7	553588
C8MT33	553592
C8MVQ9	553592
C8N2E4	553596
C8N5G6	553596
C8NBU4	638300
C8NKX1	196164
C8NLB5	196164
C8NVB0	585529
C8P7Q7	525309
C8PNW2	596324
C8PV25	553217
C8QF20	592316
C8RPY7	525262
C8S2D2	371731
C8SXQ4	667127
C9CSI7	644076
C9KMP8	500635
C9KRP7	483215
C9LB40	537007
C9M0D7	585520
C9MEQ0	656913
C9MFC0	656912
C9MKZ9	649761
C9MWZ6	634994
C9N540	591167
C9NAG3	591167
C9NBP0	591167
C9NLU8	675814
C9P0R9	675813
C9PAI4	675811
C9PNJ3	667128
C9PZB1	619693
C9Q2S6	675810
C9QBT8	675816
C9QZS5	536056
C9T3N5	520459

C9TD67	520460
C9TNX6	520463
C9TW50	520461
C9U690	520454
C9UFG5	520452
C9UPQ4	520451
C9UUP5	520450
C9VIK8	520457
C9VWE9	520455
C9XB49	568708
C9XG46	568708
D0AYD0	575591
D0B3M9	224914
D0BEG1	520488
D0BWM6	575564
D0CBH1	575584
D0CK19	166314
D0CQ97	644107
D0DAH9	501479
D0DEW5	575597
D0DRF6	575599
D0GCS1	520465
D0GLI2	596323
D0GWF7	675806
D0H1B1	675807
D0HAY2	675820
D0HKW4	675808
D0I357	675812
D0IMG7	675815
D0IRF8	290847
D0JGY7	637382
D0JRF7	637385
D0JYJ1	684950
D0P9I5	520489
D0PM67	520487
D0RKF3	437701
D0RWE2	469609
D0S154	575585
D0SBU6	575586
D0SK97	575587
D0SWR2	575588
D0SXG2	575588
D0T4W4	575589
D0TGT2	469589
D0TW93	469588
D0WHI9	649764
D0WRR7	649743
D0WTM8	674977
D0WTM9	674977
D0WTR9	674977
D0XB21	673519
D0XJL9	633149
D0Z0U2	675817
D0ZQL6	588858

D0ZVX2	588858
D1B959	525903
D1CU91	520449
D1EP32	520462
D1F0C8	520464
D1F5B4	520466
D1FGI7	520458
D1GU97	663951
D1GUN4	663951
D1JE14	115547
D1JTE9	469587
D1K0H3	457391
D1K122	457391
D1N960	340101
D1NE24	456482
D1NMD4	492476
D1P5X3	500637
D1P964	537011
D1PL49	411471
D1PV64	585502
D1QAX5	553594
D1QDM1	553594
D1QJI8	553601
D1QLW9	553601
D1QMU6	649760
D1QX82	553574
D1R1C9	553574
D1RZM0	682634
D1S852	644283
D1SFJ6	644283
D1STP1	643561
D1TRJ9	687916
D1USD5	640510
D1V7I9	298654
D1VJ48	298654
D1W172	679189
D1W584	679190
D1WNM1	596317
D1WTQ2	649189
D1WZC0	649189
D1X416	649189
D1XDG0	647653
D1XI32	647653
D1XTN2	647653
D1XXR7	553171
D1YBY7	679194
D1YRJ4	686660
D2A6Z4	591020
D2AKM3	510831
D2DKE7	3847
D2ENA8	563037
D2F0K1	585543
D2F3E3	585149
D2F6V8	585149

D2FD04	585151
D2FHG7	585151
D2FIH3	585152
D2FRT6	585159
D2FV89	585159
D2G1R1	585160
D2G535	585160
D2GAL0	585161
D2GDJ9	585161
D2GG41	585148
D2GIF3	585148
D2GMT9	585150
D2GRF0	585150
D2M0A7	649639
D2M0A8	649639
D2M823	652103
D2MRG4	683082
D2N049	683083
D2N3H5	523796
D2N727	523796
D2NM19	431946
D2T693	644651
D2U064	638
D2UIQ5	585155
D2UM21	585155
D2US18	585147
D2UW17	585147
D2W6V1	5762
D2XE03	703222
D2YCP3	671074
D2YT22	671076
D2Z890	469381
D2ZJU8	500639
D2ZNL4	521002
D3A9H4	566550
D3C3P7	648999
D3CDG1	648999
D3ERG2	703339
D3EWE2	703339
D3FLR8	567106
D3GST5	216592
D3HZA7	575611
D3I626	575612
D3IG37	575615
D3KJD2	393121
D3L8Q3	655225
D3LQM1	596312
D3LUB1	699218
D3MGB9	679195
D3ML93	686659
D3NET4	640512
D3NMG0	663278
D3R6S2	552531
D4BGS3	500640

D4BQC8	518634
D4C4J3	521000
D4CGN9	411486
D4CJS2	608534
D4E278	667129
D4EDE9	694569
D4EQ82	699185
D4ERM0	699186
D4FGQ9	679188
D4FIU5	525375
D4FR00	655813
D4FYB1	645657
D4IWH0	657324
D4J9X1	717962
D4JIY5	657317
D4JMB3	657317
D4JUK6	657319
D4JZ96	718252
D4KBX6	657322
D4KHP3	657316
D4KKM4	657315
D4KUX8	718255
D4L842	657321
D4LB01	213810
D4LSM2	657314
D4M510	657313
D4MAX0	651822
D4MB85	657310
D4MMW1	717961
D4MRM5	245012
D4MY95	245018
D4PN82	393117
D4PV03	393131
D4Q2Q3	401650
D4RW46	511680
D4SCQ2	585531
D4SUC3	427081
D4T6T8	427082
D4THX9	533240
D4TPG0	533247
D4TPL2	533247
D4U5M9	553590
D4UAD2	553590
D4UC68	553580
D4UEW8	553580
D4UKT4	246199
D4UVZ3	791166
D4V6J6	702446
D4VE40	702446
D4VTA0	702447
D4W5Y3	702450
D4WL26	702443
D4WSX3	702444
D4XFE2	742159

D4XSH1	707232
D4XXX9	552811
D4Y2P3	634956
D4YHF9	655812
D4YKA1	585530
D4YRH8	585524
D4ZRL6	696747
D5A557	696747
D5AJI0	423211
D5CZR9	714962
D5ED53	572547
D5HEL0	751585
D5N2Z0	703612
D5NGU5	243261
D5NNG9	243261
D5NZH9	649754
D5PGH4	525368
D5PPX5	525370
D5PVI3	525370
D5Q3R4	525259
D5QDC7	714995
D5QMM5	595536
D5R473	642492
D5RT72	525371
D5RZW8	525258
D5S6J4	760570
D5SGF2	762948
D5TIN5	573236
D5V9D2	749219
D5VR93	573063
D5VXB0	758678
D5XSS2	515617
D5Y2T5	520141
D5YEE1	520140
D5YQS3	515616
D5Z2K5	537209
D5ZF55	537210
D5ZXE6	566461
D5ZXV9	566461
D6AH11	457431
D6ANM1	457431
D6APU5	457431
D6B2Y5	457425
D6B6T2	457425
D6CKT3	426114
D6DEY3	717608
D6DXG1	718254
D6E416	657318
D6EIA0	457428
D6ETB7	457428
D6F3Q0	611302
D6FRD7	611304
D6GIQ4	469608
D6H2R7	585156

D6HDC3	585144
D6HGQ0	585144
D6I0F9	550672
D6ICR6	550676
D6IUH3	656380
D6J063	585157
D6J3U3	585157
D6JEG8	550677
D6JSY4	575565
D6KFN6	645465
D6KFZ4	645465
D6KI23	457416
D6KN79	450749
D6KUZ4	641146
D6LQJ9	520448
D6LU57	585154
D6LXP4	585154
D6M569	465543
D6MHI8	536227
D6SE70	548470
D6SH11	548470
D6SK87	555779
D6T4N2	553577
D6T595	553577
D6TNL9	485913
D6UAL8	762962
D6UEL5	762962
D6UJ06	585535
D6URU4	682795
D6V3L9	666684
D6VC20	596153
D6VG96	596153
D6XNQ5	637913
D7BR62	749414
D7BUY5	749414
D7BX85	749414
D7C4F7	749414
D7C6L9	749414
D7CG10	749414
D7EQ83	515615
D7H090	520453
D7HSL2	412967
D7I0M9	693985
D7I3R2	693985
D7IF77	469585
D7IN94	469592
D7IS37	469592
D7J439	585544
D7JTD0	656379
D7K2K7	457390
D7M4D7	81972
D7NAV0	563008
O05321	294
O69203	42198

P41023	1471
Q00SQ4	70448
Q02GS2	208963
Q05XX0	221359
Q05ZQ5	313625
Q088K9	318167
Q09C53	378806
Q0EXI7	314345
Q0FD96	367336
Q0FQL9	314265
Q0HEE4	60480
Q0HZK7	60481
Q12JM3	318161
Q12RQ2	318161
Q1AHV3	1901
Q1IEG8	384676
Q1N7A0	314266
Q1N9M1	314266
Q1QET2	335284
Q1V1H0	314261
Q1V617	314288
Q1VG54	314288
Q1W0J1	313595
Q1YG96	287752
Q1YY74	314280
Q1ZRK8	314292
Q26EF7	156586
Q2B690	313627
Q2BI40	207954
Q2CDR0	314256
Q2FU42	323259
Q2PYI0	360423
Q2T3G6	271848
Q2YZL0	120858
Q31RM5	1140
Q399B0	269483
Q3EVC5	339854
Q3K4R9	205922
Q3KI62	205922
Q40KA9	332415
Q45R97	1906
Q46AI7	269797
Q46UZ1	264198
Q48DZ9	264730
Q4C249	165597
Q4C4S2	165597
Q4EGU3	267410
Q4EP55	267409
Q4FVQ5	259536
Q4HG49	306254
Q4HT95	306264
Q4KID4	220664
Q4LC06	63612
Q4MSP6	269801

Q4ZNJ5	205918
Q56ZB3	3702
Q5N2M9	269084
Q5SEM5	305
Q5YQ26	37329
Q648W0	285373
Q64AF8	285379
Q64CN4	286721
Q64DW7	285367
Q69AA7	715
Q6F8V3	62977
Q6X7U0	1280
Q73NP3	158
Q76E49	17
Q7CYS1	176299
Q82BT8	33903
Q82HL1	33903
Q88PI6	160488
Q8E9X6	70863
Q8GRA5	670
Q8PS97	2209
Q8TMD1	2214
Q93AT7	556
Q9EV35	1280
Q9F0I3	380
Q9HVQ3	287

aminoadipate-semialdehyde dehydrogenase large subunit

UniProt	TXID
P07702	4932
D6VQB4	559292
B5VE77	545124
D3UEK8	643680
C7GUL2	574961
C5DUG5	559307
Q6CVV0	28985
Q8NJ21	28985
Q6FMI5	5478
C5DL66	559295
A7TQ42	436907
Q5AJ13	5476
Q5AI83	5476
C4YEZ3	5476
B9W763	573826
C4QXL0	644223
A5DVY1	36914
A3LX21	4924
Q9P3Y3	4920

aminoadipate-semialdehyde dehydrogenase small subunit

UniProt	TXID
P50113	4932
D6VTZ7	559292
A6ZU31	307796
C8Z897	643680
C7GPQ2	574961
B5VIH5	545124
B3LHK7	285006
C5DPD8	559307
Q6CNY7	28985
Q6FV34	5478
C5DMR6	559295
A7TIP9	436907
Q74Z24	33169
C4Y3S8	306902
B5RUX9	4959

bifunctional aspartokinase/homoserine dehydrogenase 1

UniProt	TXID
Q9SA18	3702
D7KK95	81972
C1EGY7	296587
Q011B0	70448
B8LAH8	391601
A0M2U0	411154
B3EL41	331678
A4AQY1	313603
Q1VVK5	313595
A7JPY9	272629
C6C9D2	579405
C7PUU9	485918
D0ZC11	498217
D3V4Q4	406818
C5S1U6	637911
D4IF04	716540
Q493T0	291272
B9CWP1	591023
B8F6B2	557723

C4Y8B8	306902	B0QSA9	456298
B5RTF4	4959	D4EGM0	694569
A5DNA8	4929	C9R4Y7	668336
Q75BB3	33169	Q0I3T2	205914
Q6C6T5	4952	A0KMM1	380703
A6ZL61	307796	A0KUA5	94122
B6JWU4	402676	A0Q5A5	401614
Q1W284	4944	A1A755	405955
P40976	4896	A1BJF3	290317
Q5B1H0	162425	A1JJC6	393305
C8VFY8	227321	A1RHH5	351745
C4JRL7	336963	A1S406	326297
C5FUL3	554155	A1SUD7	357804
B6HVW2	500485	A3D1Y2	325240
O74298	5076	A3MYX2	416269
A1CWM6	331117	A3QBV2	323850
Q4WQ17	5085	A3U8K7	216432
B0Y6V5	451804	A4CK37	313596
Q0CQS7	341663	A4IZJ0	418136
Q2UJS3	5062	A4S2Z7	436017
C5PGC9	222929	A4SGM7	290318
A1CI56	5057	A4SQ69	382245
A2QJ10	425011	A4TQH0	386656
D4DCW8	663202	A4W6C1	399742
D4B1F0	663331	A4Y921	319224
B8N3B0	332952	A5FGQ9	376686
C5MF39	294747	A5FJU6	376686
B2W755	426418	A5IJM8	390874
Q0UKD0	13684	A5UB53	374930
B0D6R6	486041	A6GW97	402612
A7ES80	665079	A6L201	435590
Q5KEK6	5207	A6L8N2	435591
Q4PDW6	5270	A6T4E2	272620
A6SKS3	332648	A6VMH4	339671
D5GM33	39416	A6WKT3	402882
D6RQF5	240176	A7FMF3	349747
B6Q471	441960	A7K433	150340
Q9P3Q7	5141	A7MIJ8	290339
Q2KGK3	242507	A7MXN1	338187
A4RCF1	148305	A7NAJ4	458234
C1GLF6	502780	A7ZH91	331111
Q2HER4	38033	A7ZVW1	331112
D2EBC1	31870	A8ALV5	290338
C0SCF5	482561	A8F6Y7	416591
C1HB82	502779	A8FSG3	425104
C5JVP0	559298	A8G9J9	399741

C5GNN8	559297
B2AAT6	5145
D1Z4D9	5147
C0NYE7	447093
C6HQN3	544712
A6RHI4	339724
C9SKY6	526221
C7Z7H8	660122
B8M7P6	441959
A8PSD0	425265
D2UYT1	5762
Q84BC7	76335
Q84BC8	76335
A0ZLL9	313624
A0Z9S0	313624
Q70LM4	54914
Q70LM5	54914
C0ZDR8	358681
A8YL71	267872
A8YH74	267872
B0JHR0	449447
B7KLZ3	65393
B7KLZ5	65393
B7KM98	65393
Q93TX2	41
Q9F636	41
A8ZKN8	329726
Q5V8A8	158786
Q1D3T0	246197
Q1D693	246197
D3P854	137722
B9K534	311402
Q50858	34
C2NRS5	526970
C3GUE4	527029
A1TW55	397945
C8TER5	329562
D0N1R2	403677
A4XD37	369723
C0YUT9	525257
C0YPM9	525257
A8LVF1	391037
B1K8A4	406425
D3NBE6	640512
B0CN27	1914
Q1BKK7	331271
A8H1F6	398579
A8J1S7	3055
A8Z601	444179
A9L5R2	399599
A9MXH0	272994
A9R026	349746
A9SKI1	145481
A9TAK5	145481
B0RSK5	509169
B0TJ92	458817
B0TZ90	484022
B0U3B9	405440
B0UWP5	228400
B1IRH1	481805
B1JL14	502800
B1KI84	392500
B1L8U8	126740
B1LF71	439855
B1VJ39	529507
B1XBC7	316385
B2FPL2	522373
B2I5Y7	405441
B2K3L1	502801
B2SFN8	441952
B2SLB1	360094
B2U218	344609
B2VH19	338565
B3EHJ1	290315
B3H092	537457
B3QL61	517417
B3QZD1	517418
B4RXF4	314275
B4S3X5	290512
B4SGC5	324925
B4STM9	391008
B4T6C5	423368
B4TIA3	454169
B4TVY4	439843
B5BLG8	554290
B5F6C1	454166
B5FAK2	388396
B5FH96	439851
B5R5H4	550537
B5REZ8	550538
B5Y255	507522
B5YNN0	35128

A0AX07	331272	B5YY96	444450
Q09E86	378806	B6EKY8	316275
Q08XI8	378806	B6HYZ9	409438
Q2VQ12	327382	B6YRU8	511995
D3VBI5	406817	B7L4C7	585055
D3VB99	406817	B7LWR0	585054
D3VJG1	406817	B7M099	585034
B4EHM7	216591	B7M9R5	585035
D1XGV9	647653	B7N2U1	585397
A7GKR8	315749	B7N7M7	585056
A7GPK0	315749	B7NHA5	585057
C6N0N1	658187	B7UI47	574521
C6MZK3	658187	B7VJ35	575788
Q5JCL8	294	B8CSE2	225849
B1HQ62	444177	B8D798	561501
B5STA5	443925	B8D8Z3	563178
A9CFI7	176299	B8EB29	407976
Q9RAH2	76334	B9IQ92	3694
Q9RAH4	76334	B9KBA1	309803
A7IZW2	329558	B9SHP2	3988
B7IJ64	405531	C0Q4E2	476213
B7IVT2	405531	C1A849	379066
C0LTS3	67255	C3LQD6	579112
C4SU96	349966	C3NVN1	593588
A4Z4I7	59625	C4K7E7	572265
C3AUX9	526998	C4LAP6	595494
C3AVQ4	526998	C4X311	484021
C3AKA4	526998	C4ZPS9	595496
C9ZGA0	680198	C5B7K4	634503
B2HPM8	216594	C5X893	4558
B2JBV4	63737	C6CJ36	561229
B2J682	63737	C6DF19	561230
A3I9A7	388400	C6ULA0	413997
C6SSJ2	511691	C6UVI7	544404
C3H9Z8	527030	C6W787	471854
C3HA00	527030	C6XUY7	485917
C3H168	527030	C7BJV9	553480
C6D408	324057	C7LK98	595499
B9X1I0	600778	C7M6I6	521097
C2U7I8	526981	C8TGD3	573235
C2STM5	526976	C8U177	585395
C2SIR2	526976	C8UFX7	585396
Q9K5M2	46234	C9XVF4	413502
Q9K5M1	46234	D0FPC1	79967
C3HSB5	527028	D0J8U6	600809
C3AE78	526997	D0JBH0	331104

C5RP77	573061
A9VUL4	315730
A9VQN3	315730
B9XAW2	320771
B9XJE5	320771
B9XIK9	320771
B9X9N1	320771
B4W3U5	118168
B4W3U1	118168
Q3M5N4	240292
Q3M1M7	240292
D7DWA7	551115
C2WM97	526987
C3EKM3	527023
C2Y3X7	526990
C2XKD9	526989
B5UTF6	405533
B9TTF3	1406
B9TTF7	1406
C2NYV7	526967
C4TZF3	527012
C3I0Y2	527019
C3FVB0	527031
C3FW25	527031
C3DBJ5	527025
C3DAI1	527025
C2YJP6	526991
Q5D6C9	5016
C3DWM3	527026
C3DJP6	527026
C3DKL0	527026
D5TKP2	714359
C2RNA0	526974
B7H7R5	405532
C2T1D0	526978
C2UMF6	526982
C2R8B3	526969
A3IP47	391612
C3BDU6	526999
C3E3N5	527027
C2N0R0	526980
C2NB23	526980
C2NB24	526980
C2NB42	526980
B8I982	394503
B8I5D1	394503

D0KMP5	561231
D0MDV7	518766
D2BX58	590409
D2C644	590168
D2QMT1	504472
D2TGQ2	637910
D2UA20	380358
D3QUG7	701177
D3RCN5	640131
D3VJK7	406817
D4G8W9	515618
D4GJL2	706191
D4I0J0	665029
D4ZGZ7	637905
D5B095	637386
D5BBZ2	655815
D5CG97	716541
D5D8Q6	641892
D5ERU4	264731
O81852	3702
P44505	727
P49079	4577
P49080	4577
Q057U6	372461
Q07Z19	318167
Q0HXE7	60481
Q0T8I6	373384
Q0TLY7	362663
Q0WJK7	632
Q11YZ9	269798
Q12KK7	318161
Q15PV6	342610
Q1C0L0	360102
Q1CMW7	377628
Q1DA24	246197
Q1DDP1	246197
Q1RGK2	364106
Q2NVZ6	343509
Q2P3L5	342109
Q2S4Q2	309807
Q2S5A4	309807
Q326M0	300268
Q32KB6	300267
Q3AUA5	340177
Q3B1F6	319225
Q3BUG6	316273

A6P624	1126
D4TRQ6	533247
C3CIZ6	527021
C3CVA4	527021
C3CIM3	527021
C3CJJ1	527021
Q2XP40	1423
Q2XP38	1423
D3V3G3	406818
D3V3G2	406818
B4B5I8	497965
B4B5I6	497965
B4B5I7	497965
C3IJN6	527020
C3IWG6	527020
C3IJC7	527020
C3IVW9	527020
C3IK59	527020
D3EB42	481743
D3EA29	481743
A5A9R5	1774
A5A9R4	1774
O68006	1402
O68008	1402
Q81DB7	226900
Q81DB4	226900
Q81DQ0	226900
Q8YTS1	103690
A9FNK4	448385
A9FNI5	448385
A9G1U1	448385
A9FNH7	448385
A9EPS4	448385
Q110E8	203124
Q4ZQ18	205918
A6ZL60	307796
Q12572	5476

Q3II20	326442
Q3Z612	300269
Q47W81	167879
Q4QP95	281310
Q4UU31	314565
Q57TQ3	28901
Q5E2Y3	312309
Q5LHR8	272559
Q64YR9	817
Q65RV0	221988
Q66ET9	633
Q69LG7	39947
Q6D0A8	29471
Q6LUQ0	74109
Q7MNR5	196600
Q7N8Z6	141679
Q7VQL7	203907
Q83MI0	623
Q87SD0	670
Q89AR4	224915
Q8A541	818
Q8D1W5	36870
Q8DEP2	672
Q8EBT4	70863
Q8FLD9	217992
Q8KAX1	1097
Q9CPD4	747
Q9KPK3	666
Q9PBB9	2371
Q9WZ17	2336
A0LY59	411154
A0Y5M3	156578
A1EM59	345076
A1FAN1	412614
A1ZLX2	313606
A2PCA5	412966
A2PWB8	412883
A2TPL3	313590
A2TXI3	313598
A2TZS9	313598
A2V048	399804
A2YZH8	39946
A3C5X9	39947
A3H4W8	417400
A3I255	388413
A3J2B4	391598

A3UWT6	314291
A3XKM8	398720
A3XM14	398720
A3Y0N4	314290
A4ATS2	313603
A4BWA7	313594
A4BY77	313594
A4C6Q1	87626
A4CPR7	313596
A4KQ81	412422
A4MYU6	374927
A4N5X2	375432
A4N5X3	375432
A4NBZ4	375177
A4NH10	374928
A4NN27	374932
A4NT79	374933
A4P0B9	375063
A5APD8	29760
A5F5S0	345073
A5FJU4	376686
A5L837	391574
A6A3E4	417398
A6ABR7	417397
A6BV47	412420
A6D3N0	391591
A6EBG6	391596
A6EME0	50743
A6EME4	50743
A6F9I2	58051
A6XUN9	404974
A7JGM5	430558
A7JKT1	442346
A8TAE4	314289
A8UJS7	391603
A9DAP5	314608
A9DJH2	391587
A9E3R4	391587
A9SKH8	145481
A9Z4W1	373665
B0A2H5	404214
B0GJP4	404218
B0GNF9	404217
B0H8M8	404216
B0HGR7	404215
B0HRY0	360099

B1EMR5	502347
B2N0A0	344610
B2NW37	444451
B2P9V5	444452
B2PMC9	444453
B2Q6N9	471874
B3AA99	478004
B3AI54	478005
B3BBQ7	478008
B3BSW2	478007
B3HEK5	340184
B3HV43	340197
B3IAR0	340185
B3IPJ3	340186
B3WQQ0	344601
B3X452	358708
B3XIB2	358709
B3YK95	439842
B4A3Y8	454168
B4AQ93	545422
B5BVJ7	439846
B5C8C3	454165
B5MPN9	439847
B5MUW4	440534
B5N9Q1	454167
B5NM12	454231
B5P861	454164
B5PKT6	465518
B5PVK2	465516
B5Q2N5	465517
B5Y6I9	309798
B6W1M1	483217
B6X9Z6	520999
B6ZSL9	502346
B7RB92	443254
B8A1Q6	4577
B8B9V9	39946
B8K9E0	391586
B9G0G2	39947
B9G2N4	39947
C0AZ70	471881
C0AZ71	471881
C0AZ72	471881
C0AZ73	471881
C0BH86	487796
C0BMV9	487797

C0PFV7	4577
C0YN60	525257
C0YW15	525257
C1HPF2	469598
C1MCB5	469595
C1NBU4	457400
C2CAH9	592313
C2DN92	525281
C2FT78	525372
C2HVX8	593585
C2I4Z9	593586
C2IJB1	593589
C2IRU2	593590
C2J710	417400
C2JFN7	593587
C2LNK8	525369
C2M454	553178
C3Q6K7	457395
C3RFB5	556260
C3TRR2	562
C3TRR5	562
C4F0K4	521005
C4F6Q0	521004
C4H1K4	547047
C4HJ88	547046
C4RWI4	349968
C4SDC5	349967
C4SIU2	349966
C4T737	349965
C4U0P8	527012
C4U9H1	527002
C4UL16	527005
C4UXR4	527004
C5PP68	525373
C5VKG7	553174
C5W226	511693
C6EBG7	469008
C6I1L5	457392
C6IS94	469586
C6S2N1	661513
C6YG19	345072
C6YNG7	543737
C6YWY8	539329
C6Z5L4	457394
C7XEF7	563193
C8QA67	592316

C8TBM1	667127
C9L1C7	483215
C9MBD5	656913
C9MHK5	656912
C9NMS3	675814
C9P6K7	675813
C9P9Y7	675811
C9PPY1	667128
C9PYZ7	619693
C9Q6G9	675810
C9QN00	675816
C9QSR6	536056
C9X5D6	568708
D0GQW3	675806
D0H513	675807
D0HH93	675820
D0HSH4	675808
D0I1I4	675809
D0I847	675812
D0ILZ3	675815
D0JEH3	637382
D0JEH4	637382
D0JNL1	637385
D0TIL4	469589
D0WSM4	674977
D0X5M7	673519
D0Z019	675817
D0ZIQ1	588858
D1JRM0	469587
D1K0B4	457391
D1P6E1	500637
D1PFP6	537011
D1PV40	585502
D1Q570	545431
D1QUK8	649760
D1RWU7	682634
D1TUN5	687916
D1VXQ7	679189
D1W2I1	679190
D1XYV0	553171
D2AHN6	591020
D2ALT6	510831
D2NAV4	431946
D2T4D4	644651
D2TW52	638
D2Y9B8	671074

D2YMK1	671076
D2Z9T1	500639
D3H385	216592
D3HVW1	575611
D3I4C6	575612
D3IGI4	575615
D4BEY9	500640
D4C1L0	521000
D4DXE1	667129
D4F1Y5	500638
D4SSY2	427081
D4T3K9	427082
D4V870	702446
D4WDD5	702443
D5BKM1	655815
D5CUT5	714962
D5H628	761659
D5H6Y3	761659
D6DT91	718254
D6GMS9	469608
D6I4Q1	550672
D6IHW3	550676
D6IJR0	656380
D6J6C9	550677
D7HED7	345074
D7HQ84	412967
D7IK30	469585
D7IVR4	469592
D7J824	585544
D7JHG5	656379
D7MG88	81972
D7N9H5	563008
O63067	3847
P27725	615
P93402	4530
Q0HL49	60480
Q0WRP9	3702
Q0YTN6	377431
Q1V779	314288
Q1Z189	314280
Q1ZCV7	314282
Q1ZMJ9	314292
Q2C7Y8	121723
Q3R3T0	155920
Q3R9F6	155920
Q3RIE3	155919

aspartate kinase

UniProt	TXID
A0AHX6	386043
A0AIN9	386043
A0AL59	386043
A0B8K8	349307
A0JSD1	290399
A0K8J8	331272
A0KLC9	380703
A0KPG0	380703
A0KSX1	94122
A0KU95	94122
A0L691	156889
A0LKA3	335543
A0LWH4	351607
A0M1M8	411154
A0M761	411154
A0NKI3	379360
A0NNX3	384765
A0PVE4	362242
A0Q0P9	386415
A0Q239	386415
A0Q8L7	401614
A0Q9U0	243243
A0R5N8	246196
A0RCH9	412694
A0RHH1	412694
A0RPZ1	360106
A0RXH8	46770
A0Y5U6	156578
A0YHT2	247633
A0YJJ7	313612
A0YY20	313612
A0Z876	247639
A0ZDZ8	313624
A0ZZN7	367928
A0ZZN8	367928
A1AIK2	405955

aspartate kinase

UniProt	TXID
P10869	4932
D3DLV4	559292
C7GSK1	574961
C8Z756	643680
B3LS65	285006
A6ZR03	307796
Q6FPL5	5478
A7TNL6	436907
C5DWQ0	559307
C5DBN1	559295
Q6CID1	28985
Q75AL3	33169
Q6BUX0	4959
A3GF89	4924
C4Y8G7	306902
C5MDW1	294747
A5DQ27	4929
B9WGK6	573826
Q5A474	5476
C4YGT6	5476
Q59ME9	5476
C4R700	644223
A5E7Q4	36914
O60163	4896
A1C9Q1	5057
Q5AS71	162425
C8V9C0	227321
C5P7Y6	222929
C4JMH7	336963
C1GF52	502780
B6JWV6	402676
Q0CA14	341663
A2R9Q7	425011
Q2UTJ9	5062
C5FIE4	554155
B6HG11	500485

Q6ZD09	39947
Q6ZD10	39947
Q74Q04	632
Q87C23	183190
Q8CZP2	632
Q8RMX0	562

aspartate-semialdehyde dehydrogenase

UniProt	TXID
A1CJF2	5057
A1D827	331117
A2QXM6	425011
A3LUL1	4924
A4RAZ2	148305
A4RR15	436017
A5AHX9	29760
A5DBS5	4929
A5DTR6	36914
A6QXL7	339724
A6SS50	332648
A6ZYB6	307796
A7F6D3	665079
A7TTV1	436907
A8J173	3055
A8P6J3	240176
A8QAI1	425265
A9TM50	145481
A9V5G9	81824
B0CZF4	486041
B0XYV5	451804
B1X4K9	39717
B2AT83	5145
B2WBK5	426418
B3LGC6	285006
B4F8G4	4577
B5VG51	545124
B6HF52	500485
B6JV33	402676
B6KIC8	508771
B6QSX9	441960
B6TWW1	4577
B8AK88	39946
B8MMS3	441959
B8MN42	441959
B8NUP8	332952

A1AR99	338966	Q6C9F1	4952	B9F5U0	39947
A1AWM9	413404	B6QGS4	441960	B9HJH4	3694
A1B0C9	318586	B8MBY8	441959	B9HVU3	3694
A1BJJ4	290317	C5JYP2	559298	B9P5X5	3694
A1C9Q1	5057	C5GM03	559297	B9QF78	432359
A1CZK2	331117	Q4WTP2	5085	B9T4M3	3988
A1ELH7	345076	B0Y369	451804	B9WAT0	573826
A1F694	412614	D4AXF4	663331	C0NEA4	447093
A1F7V9	412614	A1CZK2	331117	C0SI01	482561
A1HRY3	401526	D4DL49	663202	C1FDY1	296587
A1HSD1	401526	B8NSE7	332952	C1GLY5	502780
A1JRW0	393305	Q0UFQ9	13684	C1HBC4	502779
A1KA76	62928	C0NE81	447093	C1MKQ2	564608
A1KQ41	410289	A6R0E4	339724	C4JN68	336963
A1KUT8	272831	B2VS61	426418	C4R4N4	644223
A1R2K3	290340	B0DWL9	486041	C4XW14	306902
A1RHG6	351745	Q8NIW0	5141	C4YJJ0	5476
A1RNQ8	351745	A6S199	332648	C5DMV0	559295
A1RR70	384616	Q2GXN2	38033	C5DYK4	559307
A1S355	326297	A7E5J9	665079	C5FPC3	554155
A1S4F0	326297	D1ZV99	5147	C5GC63	559297
A1SDI0	196162	C7YTJ1	660122	C5JN67	559298
A1T000	357804	A4R1N1	148305	C5M6A4	294747
A1TGH0	350058	B8P2F0	561896	C5PIU4	222929
A1TPL2	397945	Q55N96	5207	C5X054	4558
A1TWY7	351348	Q5KBM8	5207	C6HN59	544712
A1TZ93	351348	Q4PG91	5270	C7GX38	574961
A1UMV9	189918	A8QDH2	425265	C7YLT4	660122
A1URT6	360095	A8P5G4	240176	C7YWU0	660122
A1V5G3	320388	A8PCQ5	240176	C8VAM5	227321
A1VCV4	391774	C9SG47	526221	C8Z573	643680
A1VRB0	365044	C1H9E9	502779	C9SC07	526221
A1VYU2	354242	C6HG88	544712	D0MXD8	403677
A1W6E5	232721	A9UQW7	81824	D1Z4H6	5147
A1WSM0	391735	C0SD16	482561	D3B6X7	670386
A1WXK4	349124	B7G5H9	556484	D4AM80	663331
A1ZLK8	313606	B8C772	35128	D4DGR6	663202
A2BN07	415426	A8I0V1	3055	D5A9F9	3332
A2BTM9	146891	Q6YS33	39947	D5GMF0	39416
A2BZ34	167542	Q6YS32	39947	D6VSD8	559292
A2C5B2	167555	B9EW03	39947	D7KCK0	81972
A2C5S9	59922	A9TQ86	145481	P13663	4932
A2P9B5	412966	A9T456	145481	P78780	4896
A2P9X5	412966	B4FA80	4577	Q01GI9	70448
A2PTS2	412883	C4IZ68	4577	Q0CC47	341663
A2PV45	412883	A3RL74	4577	Q0V6X4	13684

A2Q1Y6	3880
A2R9Q7	425011
A2RM64	416870
A2SAY3	412022
A2SIL8	420662
A2SPR3	410358
A2TRG8	313590
A2TUZ9	313590
A2TYX2	313598
A2UWR4	399804
A2UZ62	399804
A2VM92	348776
A2VX76	350702
A2W955	350701
A3CQ63	388919
A3CS06	368407
A3D0Q0	325240
A3D782	325240
A3DF78	203119
A3EL14	345075
A3ENB0	345075
A3EV90	419542
A3GF89	4924
A3GMZ4	417399
A3GZD2	417400
A3H4N9	417400
A3HVH3	388413
A3ICN2	388400
A3IKB3	391612
A3J2M8	391598
A3JB05	270374
A3JGQ4	270374
A3JR41	388401
A3K071	388399
A3K8R5	388399
A3KLD2	1390
A3L5G7	350704
A3LMF0	350703
A3M3S8	400667
A3ML38	320389
A3MT45	410359
A3N074	416269
A3NB45	320373
A3NWX3	357348
A3PFD6	167546
A3PGZ8	349101
D7M5Z6	81972
D7L9I8	81972
D7M587	81972
Q9LYU8	3702
Q9S702	3702
O23653	3702
B9Q868	432359
B9PMV9	5811
B6KET9	508771
C5WSV9	4558
C5XH02	4558
C5X492	4558
A9NUI1	3332
B8A8I6	39946
B8ANP4	39946
D7FGV8	2880
Q9XHC5	3847
B9RGY9	3988
C1FDY8	296587
D0NKQ0	403677
D0NN44	403677
A4RQT3	436017
B7ZYQ0	4577
Q5JK18	39947
A2Q1Y6	3880
B1X4L4	39717
B7ZZJ1	4577
B8AKZ6	39946
B8ANP3	39946
B9F9Y5	39947
B9H1N5	3694
B9NDG6	3694
C0PTM7	3332
C4J2N3	4577
Q10AJ5	39947
Q851Z6	39947
Q852E4	39947
Q2H525	38033
Q2KF40	242507
Q2U4J7	5062
Q4P4R4	5270
Q4WWR8	5085
Q560L0	5207
Q5ALM0	5476
Q5B3T7	162425
Q5KPK7	5207
Q6BS59	4959
Q6C968	4952
Q6CQJ0	28985
Q6FVK7	5478
Q750W4	33169
Q7SF95	5141
Q8VYI4	3702
Q93Y73	39947
Q99103	5270
Q9LQU9	3702

A3Q793	164757
A3QB03	323850
A3RL74	4577
A3RW41	342110
A3S7C9	52598
A3SHR7	89187
A3SJD3	89187
A3SS91	314267
A3TI68	313589
A3TST3	252305
A3TZ70	252305
A3U5N4	216432
A3UGH9	314254
A3UHE7	314254
A3UV44	314291
A3UZX6	314291
A3V784	314232
A3VAQ5	314271
A3VFP4	314271
A3VUX3	314260
A3W4E5	314264
A3W8G6	314264
A3WF70	237727
A3WKY2	314276
A3WR07	314253
A3X4H9	314262
A3XH83	398720
A3XHB5	398720
A3XSQ5	314290
A3XXN2	314290
A3Y1V9	314290
A3YL18	360111
A3YRL8	360108
A3YWN5	69042
A3Z426	221360
A3ZDM7	360112
A3ZIQ7	360110
A4A2N2	314230
A4ACW7	314285
A4AKA7	312284
A4AN99	313603
A4BA98	314283
A4BCJ3	314283
A4BKP3	314283
A4BU40	314278
A4BWM5	313594

A4CCF9	87626
A4CFG7	87626
A4CH35	313596
A4CYH4	59931
A4E939	411903
A4EED8	391593
A4EY38	388739
A4F6F7	405948
A4FXG2	402880
A4G4M6	204773
A4IMF0	420246
A4IT05	420246
A4J3P8	349161
A4J5V6	349161
A4JFC6	269482
A4KMH7	395095
A4LD52	425067
A4NFS3	374928
A4QAP5	340322
A4R1N1	148305
A4RQT3	436017
A4SGV3	290318
A4SNY2	382245
A4SSA0	382245
A4SX66	312153
A4T601	350054
A4TH38	386656
A4TYG9	55518
A4VFY3	379731
A4VJB4	379731
A4VXD8	391295
A4W3N4	391296
A4W5D9	399742
A4WJH1	340102
A4WQ78	349102
A4X1G1	369723
A4XI70	351627
A4XJT8	351627
A4XWE1	399739
A4Y377	319224
A4Y930	319224
A4YHF2	399549
A4YKT4	114615
A5CEL9	357244
A5CPE1	443906
A5CWK6	412965

A5D1T6	370438
A5D2Q4	370438
A5DQ27	4929
A5E7Q4	36914
A5ETF5	288000
A5EWA4	246195
A5F0I0	345073
A5F8T6	345073
A5F9B9	345073
A5FMJ7	376686
A5FPB7	216389
A5FX93	349163
A5G2X0	349163
A5G3X9	351605
A5GHU2	32051
A5GQ19	316278
A5I2B0	441771
A5I4I4	441771
A5I761	441771
A5ICW8	400673
A5IM65	390874
A5ISK9	359786
A5ISS5	359786
A5KFB7	398000
A5KLH8	411460
A5L289	391574
A5L311	391574
A5L9Y3	406556
A5LJX9	406557
A5LVH5	406558
A5LYY5	406559
A5M9Y2	406560
A5MJ05	406561
A5MNX4	406562
A5MX81	406563
A5N5T0	431943
A5N851	431943
A5P8N4	161528
A5TJV1	370895
A5U935	419947
A5ULF9	420247
A5UUW3	357808
A5UXT9	357808
A5VEX5	392499
A5VJ54	557436
A5VSK2	444178

A5W0E0	351746
A5WFW2	349106
A5WTS7	336982
A5XH62	334802
A5XYL4	334803
A5Z523	411463
A5ZHY7	411901
A5ZN67	411459
A5ZX96	411459
A6A2L1	417398
A6A6C7	417398
A6A7C1	417398
A6AD32	417397
A6AFJ3	417397
A6ARL0	410291
A6AW08	410291
A6AXY6	419109
A6BE05	411462
A6BHP7	411462
A6BXE9	412420
A6CCL9	344747
A6CQ71	161544
A6CSE9	161544
A6D492	391591
A6DC58	391592
A6DZ35	391613
A6E4Q6	391613
A6EJC9	391596
A6EJX7	391596
A6ELU0	50743
A6F3M2	443152
A6F5K2	443152
A6FG66	58051
A6FTB0	351016
A6FVL0	351016
A6FXK7	391625
A6GNX5	391597
A6GWE2	402612
A6H562	1718
A6L6Z5	435590
A6LDJ0	435591
A6LIV2	435591
A6LKZ7	391009
A6LP61	391009
A6LSR3	290402
A6M137	290402

A6P129	411467
A6Q440	387092
A6Q7F7	387093
A6QGM9	426430
A6QGU4	426430
A6R0E4	339724
A6S199	332648
A6SZU7	375286
A6TGT3	272620
A6TLT7	293826
A6TT14	293826
A6TTL7	293826
A6U1E7	359787
A6U1L4	359787
A6UCF4	366394
A6UP33	406327
A6UUT8	419665
A6VA70	381754
A6VFV3	426368
A6VMU6	339671
A6VUG2	400668
A6W1F0	400668
A6W564	266940
A6WR15	402882
A6WSQ2	402882
A6WXQ1	439375
A6XQC5	404974
A6XXZ2	404974
A6Y3J9	345074
A6Y6F5	345074
A6ZR03	307796
A7A3F1	411481
A7A3F2	411481
A7AJF1	411477
A7ALK7	411477
A7B7L2	411470
A7BA97	411466
A7C2B7	422289
A7E5J9	665079
A7FDG7	349747
A7FUE6	441770
A7FVY4	441770
A7FYT3	441770
A7GDW7	441772
A7GFZ7	441772
A7GIS3	441772

A7GNP1	315749
A7GRD0	315749
A7GXR9	360105
A7H3U8	360109
A7HBV8	404589
A7HGP3	404589
A7HJ59	381764
A7HMJ6	381764
A7HTX9	402881
A7I219	360107
A7I4F0	456442
A7IIM4	78245
A7JF59	430558
A7JP25	442346
A7JRW3	272629
A7K083	150340
A7K2W3	150340
A7LQZ3	411476
A7MPC0	290339
A7MU63	338187
A7MVY6	338187
A7MYT4	338187
A7NNC9	383372
A7NNU7	383372
A7TNL6	436907
A7UXV0	411479
A7V9W7	411479
A7VEN4	411489
A7VVX2	428125
A7X1X8	418127
A7X268	418127
A7Z1C7	326423
A7Z4U7	326423
A7Z7D6	326423
A7ZEE3	360104
A7ZUP2	331111
A8A7C3	331112
A8A8Y1	453591
A8ANA7	290338
A8EC50	360118
A8EU35	367737
A8EZV3	293613
A8F2N8	416276
A8F563	416591
A8F6Y7	416591
A8F9Y4	315750

A8FDE5	315750
A8FG00	315750
A8FL06	407148
A8FRF5	425104
A8G7F3	93060
A8GKD3	399741
A8GPP7	293614
A8GTK8	392021
A8GYB2	391896
A8H0H5	398579
A8HYR1	438753
A8I0V1	3055
A8KVW7	331978
A8KYC0	298653
A8LPU4	398580
A8LYI6	391037
A8M9I2	397948
A8MLJ3	350688
A8P5G4	240176
A8PCQ5	240176
A8QDH2	425265
A8RFV4	428127
A8RKF7	411902
A8RPC8	411902
A8SAP6	411485
A8SVF6	411474
A8SWN4	411474
A8T920	314289
A8T9C1	314289
A8TN89	331869
A8U5B0	333990
A8U8Z2	333990
A8UF44	391603
A8UQW2	392423
A8YNK2	267872
A8YUS9	405566
A8Z209	451516
A8Z3X1	451516
A8Z5R6	444179
A8ZVC2	96561
A8ZWG1	96561
A9A2X3	436308
A9AAT1	444158
A9AHP0	395019
A9AZT6	316274
A9BD92	93059

A9BHR5	403833
A9BZ49	398578
A9CG68	176299
A9CV01	314608
A9D7Q9	411684
A9DLD8	391587
A9DXS7	391624
A9E1D4	391624
A9EKH3	383629
A9G265	391619
A9G6H5	448385
A9GE28	448385
A9GH52	448385
A9HBT2	391595
A9HJ44	272568
A9HXJ3	340100
A9IQF8	382640
A9K9D9	412021
A9KG29	434922
A9KIT6	357809
A9KKY1	357809
A9KYF7	399599
A9L371	399599
A9M0T4	374833
A9M8K7	483179
A9N1J4	272994
A9NCN8	360115
A9NGC4	441768
A9NUI1	3332
A9R537	349746
A9T456	145481
A9TQ86	145481
A9UQW7	81824
A9VPK3	315730
A9VS42	315730
A9W1A7	419610
A9WE20	324602
A9WV49	288705
A9ZDY0	373665
A9ZH60	360117
B0A466	404214
B0AAJ7	445973
B0AC61	445973
B0AMV6	486624
B0AWN3	486624
B0B7T1	471472

B0BBZ6	471473
B0BV45	452659
B0CFC7	329726
B0CIC5	470137
B0DWL9	486041
B0G375	411461
B0G5N1	411461
B0GIZ4	404218
B0GSQ6	404217
B0HB27	404216
B0HKQ7	404215
B0I0J0	360099
B0JW35	449447
B0K1C5	399726
B0K9N9	340099
B0KR42	76869
B0MH07	411490
B0MQZ2	428128
B0N3M5	445974
B0NAL0	411468
B0NJL0	411468
B0NL25	449673
B0NLI1	449673
B0P2P4	411484
B0PHG2	445972
B0Q157	486619
B0Q4A0	486619
B0QFI6	486621
B0QN02	486621
B0R7Q7	478009
B0RIH0	31964
B0S3W1	334413
B0SA46	355278
B0SK85	456481
B0SW83	366602
B0TFR7	498761
B0THT1	498761
B0TTM0	458817
B0TWJ2	484022
B0UAV2	426117
B0VAI4	509173
B0VL28	509170
B0Y369	451804
B1BA09	445337
B1BG33	445334
B1BWW7	451755

B1BZW6	428126
B1EQI2	502347
B1EZ82	486623
B1F0J1	486623
B1FKJ7	396596
B1G6I2	396598
B1GG61	486620
B1GKS8	486620
B1GYX2	471821
B1HKT6	320374
B1HW85	444177
B1HX07	444177
B1I3M6	477974
B1I9K7	487214
B1IFH4	498213
B1II40	498213
B1ILR1	498213
B1IUM8	481805
B1JCG8	390235
B1JJM6	502800
B1JUK4	406425
B1KEK8	392500
B1KS26	498214
B1KWJ8	498214
B1L217	498214
B1L7E2	374847
B1L7R4	374847
B1LBQ8	126740
B1LPI8	439855
B1M764	426355
B1MFP2	561007
B1MYF1	349519
B1Q9P6	445335
B1QD88	445335
B1QED8	445335
B1QI19	445336
B1QQ19	445336
B1QQA6	445336
B1QUX2	447214
B1QXV2	447214
B1RAI4	451754
B1RIJ0	451756
B1RQ46	451757
B1RYZ5	453362
B1S7G7	473819
B1S7G8	473819

B1SBX6	471872
B1T5V9	396597
B1UM92	486622
B1UR09	486622
B1V5N7	488537
B1VI49	504474
B1VM91	455632
B1WUK6	43989
B1X4L4	39717
B1XC23	316385
B1XL09	32049
B1XUY4	452638
B1XY40	395495
B1YBF5	444157
B1YJ37	262543
B1YSM4	398577
B1Z916	441620
B1ZVC9	452637
B2A3B1	457570
B2DG78	453361
B2DLU1	453363
B2DRR0	453364
B2E1H5	453365
B2E7A0	453366
B2EAZ6	486409
B2EAZ7	486409
B2G6M5	557433
B2GC07	334390
B2GFE8	378753
B2H9W6	331109
B2HKD0	216594
B2HWK6	405416
B2IHL9	395963
B2ILV9	516950
B2IWV0	63737
B2JIR7	391038
B2K4Y7	502801
B2KAQ6	445932
B2N308	344610
B2NQI0	444451
B2P9L0	444452
B2PJ63	444453
B2Q557	471874
B2RGX0	431947
B2S827	430066
B2SXP5	398527

B2TJ64	508765
B2TN16	508765
B2TX49	344609
B2U9Q2	402626
B2UNR2	349741
B2UUZ1	512562
B2V362	508767
B2V4H8	508767
B2V9P0	436114
B2VKB2	338565
B2VS61	426418
B3A628	478004
B3AH24	478005
B3AZW5	478006
B3BBY1	478008
B3BTU6	478007
B3C6K3	471870
B3CB82	471870
B3CM91	570417
B3CRW7	334380
B3DUB0	205913
B3DUB1	205913
B3DZ52	481448
B3E389	398767
B3E698	398767
B3EI31	290315
B3EPV0	331678
B3ERE0	452471
B3GXE5	537457
B3HEP7	340184
B3HRP9	340197
B3I357	340185
B3IGL5	340186
B3J3Z5	405536
B3JAA7	405536
B3JMK1	470145
B3LS65	285006
B3PCZ7	498211
B3PP82	491916
B3QCF4	395960
B3QLQ6	517417
B3QZB7	517418
B3R4D5	164546
B3T3S9	455570
B3TAR4	455605
B3W7E9	543734

B3W9N2	543734
B3WJ86	344601
B3WYM4	358708
B3XCA1	358709
B3XRK1	349123
B3YH84	439842
B3YRS1	405917
B3YTW9	405917
B3Z5U1	451707
B3ZDS3	451707
B3ZMW0	451709
B3ZPL9	451709
B4A9S5	454168
B4ADT4	536229
B4AIT7	536229
B4AM64	536229
B4ARI9	545422
B4AVB2	497965
B4BIY5	495036
B4BNE7	495036
B4CYH0	497964
B4ED69	216591
B4EYR9	529507
B4FA80	4577
B4R983	450851
B4RGD1	450851
B4RL30	521006
B4RXP4	314275
B4S677	290512
B4SGX9	324925
B4T1R4	423368
B4TDK3	454169
B4TQN7	439843
B4U547	552526
B4U8L3	380749
B4UC42	447217
B4UD69	447217
B4VBV4	465541
B4VQZ0	118168
B4W759	391600
B4WSH8	91464
B4X4V9	236097
B5BJU3	554290
B5BVW1	439846
B5CLV9	471875
B5CZK5	484018

B5E7F4	512566
B5EI19	404380
B5EME6	380394
B5EVR3	388396
B5F1N9	454166
B5FAD2	388396
B5FGL5	388396
B5FQP4	439851
B5GJ96	465543
B5H3B4	443255
B5HA41	457429
B5HKD1	457429
B5HVP8	463191
B5ITZ0	391623
B5IXZ5	391626
B5JEX7	382464
B5JV97	391615
B5K6K3	391616
B5MQJ9	439847
B5MYF8	440534
B5N7P8	454167
B5NPR7	454231
B5P649	454164
B5PIY7	465518
B5PYA3	465516
B5Q4N1	465517
B5QF05	478547
B5QKT0	486408
B5QQ69	486408
B5QYI3	550537
B5R7R9	550538
B5RZM6	305
B5SM42	564066
B5UR66	405533
B5UWV7	405533
B5V3G4	451708
B5VCI3	451708
B5W4M6	513049
B5WJ98	516466
B5XY04	507522
B5Y6I9	309798
B5YEX6	309799
B5YGC1	289376
B5Z0C3	444450
B5Z8M4	563041
B5ZJZ3	272568

B5ZRR6	395492
B6ANX7	419541
B6B1B3	314270
B6B8G8	439496
B6BIN7	439483
B6BWK1	314607
B6BZW5	473788
B6C0I4	473788
B6ENI0	316275
B6FNG8	500632
B6FRH4	500632
B6FZC6	500633
B6FZT4	500633
B6G839	445975
B6HG11	500485
B6I5N6	409438
B6IUT9	414684
B6J045	434923
B6J6Z0	434924
B6JA03	504832
B6JN69	570508
B6KET9	508771
B6QGS4	441960
B6R5C2	439495
B6VUM5	483217
B6WTF7	411464
B6XE58	520999
B6XXL9	566552
B6XXM0	566552
B6Y6J9	569881
B6YRS7	511995
B6ZVK1	502346
B7AGZ1	483216
B7AMN6	483216
B7ANB7	483218
B7BA50	537006
B7BDF7	537006
B7C765	518637
B7CLT8	557724
B7DRK9	543302
B7DS75	543302
B7G5H9	556484
B7GG62	491915
B7GM98	491915
B7GM99	491915
B7GT70	391904

B7GT71	391904
B7GWX5	557600
B7HDS3	405532
B7HIG3	405532
B7HLD3	405534
B7HMJ1	405534
B7I9N9	480119
B7IF16	484019
B7IHJ1	484019
B7IRB2	405531
B7ITQ0	405531
B7J4F0	243159
B7JIX6	405535
B7JJ77	405535
B7K279	41431
B7KCU2	65393
B7L249	440085
B7LAW9	585055
B7LKZ7	585054
B7M7T3	585034
B7MJ14	585035
B7N2N1	585397
B7NFW7	585056
B7NRY9	585057
B7QX91	439497
B7R8S8	391606
B7RE47	443254
B7RNK8	391589
B7RTN9	247634
B7UPI5	574521
B7UY30	557722
B7VI89	575788
B7VK43	575788
B7VQ40	575788
B7X5P9	399795
B7ZYQ0	4577
B7ZZJ1	4577
B8A8I6	39946
B8AKZ6	39946
B8ANP3	39946
B8ANP4	39946
B8C772	35128
B8CT83	225849
B8CW92	373903
B8CWF5	373903
B8DDI0	552536

B8DE73	552536
B8DHZ7	552536
B8DPE6	883
B8DVK4	442563
B8DVK5	442563
B8E0J9	515635
B8E8U5	407976
B8EB85	407976
B8EI30	395965
B8F9C8	439235
B8FJ92	439235
B8FQY9	272564
B8G1K9	272564
B8GB89	326427
B8GJX1	521011
B8GQV6	396588
B8H212	565050
B8HBX6	452863
B8I6L4	394503
B8I6L8	394503
B8IUB2	460265
B8IZW8	525146
B8J6T3	455488
B8J949	455488
B8K3C0	391586
B8KF70	566466
B8KTM3	565045
B8KY69	565045
B8MBY8	441959
B8NSE7	332952
B8P2F0	561896
B8Y631	1471
B8Y634	1471
B8ZLI0	561276
B8ZT15	561304
B9AEG5	483214
B9BGL6	513051
B9C002	513052
B9C7Q3	513053
B9CKN6	553184
B9CRN6	553212
B9CRV1	553212
B9D1I1	553218
B9DP21	396513
B9DP88	396513
B9DTI9	218495

B9DZB1	583346
B9E1L1	583346
B9EBT0	458233
B9EBZ4	458233
B9EW03	39947
B9F9Y5	39947
B9H1N5	3694
B9IV89	361100
B9IX38	361100
B9JB77	311403
B9JT15	311402
B9K868	309803
B9KG13	306263
B9KGP2	320483
B9KM19	557760
B9L3Q0	309801
B9LAF0	598659
B9LGR6	480224
B9LUP7	416348
B9M2T6	316067
B9MAU0	535289
B9ML58	521460
B9MRC9	521460
B9NDG6	3694
B9NPL0	467661
B9NYZ1	93058
B9PMV9	5811
B9Q868	432359
B9QSN0	244592
B9RGY9	3988
B9WGK6	573826
B9WWV7	286604
B9XB17	320771
B9XVH8	544405
B9XZT6	544406
B9YCJ4	545696
B9Z6F9	279714
C0ARP1	471881
C0BAN7	470146
C0BCI4	470146
C0BI19	487796
C0BP82	487797
C0BR48	547043
C0BR49	547043
C0BYP5	553973
C0CMH9	476272

C0D843	518636
C0DV21	546274
C0DZQ5	566549
C0EEV4	537013
C0EJK3	546264
C0EZ73	411469
C0FWA6	622312
C0G7Q6	595497
C0GCS3	555088
C0M8L5	553482
C0MFQ0	553483
C0N1T9	637616
C0N7M2	637616
C0NE81	447093
C0PTM7	3332
C0Q4C5	476213
C0Q9T4	177437
C0QG57	177437
C0QRV4	123214
C0R233	565034
C0R3Y5	66084
C0RFB7	546272
C0VK18	525244
C0VRK3	548477
C0WBE1	563191
C0WHW5	525260
C0WRR3	525318
C0WYX1	525325
C0X696	491074
C0XBX1	525326
C0XH30	525327
C0XU53	525263
C0YCK9	595498
C0YKJ2	525257
C0Z0S1	585517
C0Z9I8	358681
C0ZF36	358681
C0ZKZ0	358681
C0ZN70	234621
C1A411	379066
C1AIG3	561275
C1B9U9	632772
C1C5F0	488221
C1CCH9	488222
C1CIR4	488223
C1CPS3	487213

C1CV31	546414
C1D4K5	557598
C1DQF8	322710
C1DWH7	204536
C1EP22	572264
C1EPH7	572264
C1F655	240015
C1FDY8	296587
C1FM03	536232
C1FNA6	536232
C1FS50	536232
C1GF52	502780
C1H9E9	502779
C1HQ45	469598
C1HX96	528345
C1I573	457396
C1I7H4	457396
C1M5C5	469595
C1MFF3	469595
C1MSF0	564608
C1NAP5	457400
C1PAD2	345219
C1V8K8	469382
C2BEW6	525254
C2BKS8	525264
C2C141	525367
C2C224	525367
C2C3H4	525367
C2C8K2	592313
C2CE13	592313
C2CED7	592313
C2CS84	525268
C2D2Q3	525310
C2DIA0	525278
C2DTK9	525281
C2E688	525330
C2E8A9	525362
C2EE06	525362
C2EFX9	525364
C2EK91	525365
C2EMK9	525365
C2ESS9	525366
C2EWS2	491077
C2F3S0	548485
C2FAU4	525337
C2FC13	525337

C2FNC6	525338
C2FP75	525338
C2FUG1	525372
C2FUS4	525372
C2GC87	548473
C2GCF0	548473
C2GJE2	548478
C2GQH9	525341
C2GSR0	548480
C2GSR1	548480
C2H019	525271
C2HNU3	525306
C2HPZ2	525306
C2HTK8	593585
C2HUC3	593585
C2HZW4	593585
C2I1Q7	593586
C2I4B6	593586
C2IGF8	593589
C2IK37	593589
C2IKT0	593589
C2IN69	593590
C2INK6	593590
C2ISP0	593590
C2IY76	417400
C2IYN8	417400
C2JBD5	593587
C2JE88	593587
C2JEL6	593587
C2JL92	491075
C2JTL4	525361
C2JZF5	525361
C2KC67	491076
C2KFR1	491076
C2KKI5	586220
C2KSX4	585199
C2KVF0	585501
C2LM14	525369
C2LTW1	596322
C2LYR1	629742
C2M0I0	629742
C2M5D0	553178
C2MJ68	526973
C2MPD0	526973
C2MZB1	526980
C2N4U1	526980

C2NG04	526970
C2NLK3	526970
C2NXH5	526967
C2P2E4	526967
C2PDD2	526971
C2PIW5	526971
C2PUE7	526972
C2PZF0	526972
C2QAE7	526968
C2QFL4	526968
C2QRF7	526977
C2QWX1	526977
C2R6I4	526969
C2RBZ4	526969
C2RLI3	526974
C2RRV3	526974
C2S244	526975
C2S7J7	526975
C2SIG4	526976
C2SNU9	526976
C2SZD0	526978
C2T4Q3	526978
C2TEY6	526979
C2TKC0	526979
C2TVP4	526981
C2U136	526981
C2UCA5	526982
C2UHR7	526982
C2UTR6	526983
C2UZ39	526983
C2VA66	526984
C2VFM0	526984
C2VS39	526985
C2VXL1	526985
C2W6L1	526986
C2WAU4	526986
C2WKV4	526987
C2WRH8	526987
C2WZH3	526988
C2XA85	526989
C2XFF8	526989
C2XSF4	526990
C2XXR0	526990
C2Y942	526991
C2YE50	526991
C2YQ26	526992

C2YV82	526992
C2ZBI9	526993
C2ZMZ0	526994
C2ZT22	526994
C3A4E1	526997
C3A9I9	526997
C3AK76	526998
C3AVI6	526998
C3B240	526999
C3BDU1	526999
C3BIU2	527000
C3BNI7	527000
C3C0S0	527024
C3C6C3	527024
C3CH72	527021
C3CME7	527021
C3D029	527025
C3D5H9	527025
C3DIB2	527026
C3DNK0	527026
C3E1Z4	527027
C3E742	527027
C3EJ84	527023
C3EPB1	527023
C3F016	527022
C3F5R2	527022
C3FIK7	527031
C3FW64	527031
C3G1C2	527032
C3G6V0	527032
C3GMQ4	527029
C3GTH6	527029
C3GZF3	527030
C3H4W1	527030
C3HGV1	527028
C3HMG2	527028
C3HYZ9	527019
C3I4R9	527019
C3IHW1	527020
C3IN06	527020
C3J4H1	550542
C3J5C1	550542
C3JNY9	596309
C3JXY0	216595
C3KUV1	515621
C3KW45	515621

C3L0A7	515621
C3L6T7	568206
C3L7C7	568206
C3LRC2	579112
C3LS37	579112
C3LW86	579112
C3MHQ7	394
C3MPX3	429572
C3MVA0	427317
C3N5J9	427318
C3NE63	439386
C3NHK0	419942
C3NS49	593588
C3NW33	593588
C3NWG8	593588
C3P5K3	592021
C3P6P0	592021
C3PJR3	548476
C3PLN0	347255
C3PUW7	457395
C3QFQ4	556258
C3R1I6	469590
C3R724	556260
C3RLD1	556270
C3SHV7	562
C3X6X8	556268
C3X7C3	556269
C3XIA9	613026
C3XK11	556267
C4AQZ6	320390
C4FBS6	521003
C4FCI1	518635
C4FCI2	518635
C4FIV0	432331
C4FPT6	546273
C4FXW4	592026
C4G635	592010
C4GBQ7	626523
C4GHX0	629741
C4HAZ3	547047
C4HE98	547046
C4HRJ3	545431
C4IH31	632245
C4IJA9	632245
C4IT74	641140
C4IZ68	4577

C4J2N3	4577
C4JMH7	336963
C4K1G5	562019
C4KH64	426118
C4KPW6	536230
C4L2D0	360911
C4L8A4	595494
C4LGL9	645127
C4MCM4	326425
C4PML8	672161
C4PR37	580049
C4R700	644223
C4RE98	219305
C4RX34	349968
C4SHY8	349967
C4ST45	349966
C4T675	349965
C4TZB5	527012
C4UDY7	527002
C4UIR8	527005
C4UXJ0	527004
C4V2Z3	638302
C4VHX4	553209
C4VS19	630527
C4WAN8	596319
C4WAV3	596319
C4WJ76	641118
C4X024	484021
C4XQB5	573370
C4Y8G7	306902
C4YGT6	5476
C4YYG6	444612
C4Z302	515620
C4ZHN9	515619
C4ZM70	85643
C4ZMU8	85643
C5A0W1	595496
C5A1P0	593117
C5A978	626418
C5AVJ3	272630
C5B6Y8	634503
C5B6Y9	634503
C5BMP9	377629
C5BQN6	377629
C5BXS8	471853
C5C8L8	465515

C5CHX6	521045
C5CPW6	543728
C5D5N5	471223
C5D8G2	471223
C5D9E3	471223
C5DBN1	559295
C5DWQ0	559307
C5E9E5	537937
C5E9E6	537937
C5ELE2	457421
C5EU25	457421
C5EYE1	537972
C5F629	537973
C5F8N0	537973
C5FIE4	554155
C5GM03	559297
C5JYP2	559298
C5MDW1	294747
C5N582	450394
C5N5F3	450394
C5NFL5	436115
C5P7Y6	222929
C5PUM6	525373
C5PUV0	525373
C5Q056	548474
C5Q0C3	548474
C5Q8W7	525374
C5Q928	525374
C5QIV5	548475
C5QJ24	548475
C5QS60	525378
C5QSC7	525378
C5QWU0	525376
C5QX03	525376
C5RCN7	585506
C5RGM8	573061
C5RGM9	573061
C5RHU0	573061
C5RVV3	573062
C5S296	637911
C5SMM0	573065
C5T3D5	573060
C5TH79	555217
C5TNX7	596320
C5TV95	573059
C5UB33	509193

C5UX60	536233
C5UYS8	536233
C5V5J1	395494
C5VA09	553207
C5VIM0	553174
C5VSK7	592027
C5VZX6	218494
C5WBQ8	511693
C5WEL3	486410
C5WSV9	4558
C5X492	4558
C5XH02	4558
C5ZJK3	357347
C5ZWN7	537970
C6A3H8	604354
C6A4V8	604354
C6A658	580050
C6A659	580050
C6AC51	634504
C6AGR6	555970
C6AGR7	555970
C6APJ2	634176
C6AXD7	395491
C6BG99	428406
C6BV82	526222
C6C8Q2	579405
C6CI16	561229
C6CU07	324057
C6D2E0	324057
C6DFP5	561230
C6DNE9	478434
C6E2T8	443144
C6EE00	469008
C6GN14	568813
C6GWP0	568814
C6HG88	544712
C6HZV4	412449
C6I1P7	457392
C6I6B6	457392
C6ISS1	469586
C6IW18	621372
C6J3S6	621372
C6JCT0	457412
C6JE71	457412
C6JPW0	469618
C6LCF7	478749

C6M9R2	547045
C6MFN5	153948
C6MN14	443143
C6N2R9	658187
C6NSA4	637389
C6PN65	536227
C6PQG2	536227
C6QIY0	582899
C6QKD6	581103
C6QMF1	581103
C6QRP3	581103
C6R643	553201
C6R7K7	553206
C6RCV4	553219
C6RNV7	596318
C6RTL4	661513
C6RUV9	661513
C6S1R1	661513
C6S7V8	662598
C6SD68	663926
C6SKK1	295996
C6SNV9	511691
C6TTL4	320371
C6UJP8	413997
C6V1Y4	544404
C6VI55	644042
C6VN31	644042
C6VWJ0	471854
C6WF56	446462
C6WY10	583345
C6X1M4	531844
C6X7H1	582744
C6XG43	537021
C6XPY2	582402
C6XYQ5	485917
C6Y1Y1	485917
C6YIH3	345072
C6YJA6	345072
C6YK51	345072
C6YTA0	539329
C6Z1U8	457394
C7BNG6	553480
C7C0D3	592205
C7CE18	661410
C7CPH6	565636
C7CWN8	565637

C7DBA8	633131
C7G9N5	536231
C7GSK1	574961
C7H759	411483
C7HGN3	572545
C7HKA9	588857
C7IFT6	588581
C7IFU0	588581
C7IPL5	589861
C7JGR6	634452
C7JMK1	634453
C7JWR7	634454
C7K711	634455
C7KG80	634456
C7KQJ4	634457
C7KZV9	634458
C7L258	634459
C7LE98	568815
C7LK10	595499
C7LN53	525897
C7LSQ4	525897
C7M1N2	525909
C7M5N6	521097
C7MHT0	446465
C7MMI9	469378
C7MQR4	471857
C7N6U0	471855
C7N9C3	523794
C7NAR5	523794
C7NGA3	478801
C7NKC4	478801
C7NN19	519442
C7NZY1	485914
C7P9D0	573064
C7PP97	485918
C7PQ37	485918
C7Q5M7	479433
C7QKA7	479433
C7QN85	395962
C7QZS8	471856
C7RNF3	522306
C7T8R5	568703
C7TEK8	568703
C7TFG0	568704
C7TLB8	568704
C7U460	565638

C7UCE0	565642
C7UK02	565641
C7USX1	565650
C7V0W8	565640
C7V889	565644
C7VHQ2	565646
C7VQU4	565649
C7VYK2	565647
C7WCN6	565648
C7WH61	565643
C7WSD2	565651
C7WV76	565645
C7X6K7	563193
C7XCF7	563193
C7XGF8	575595
C7XI43	575595
C7XTT4	575594
C7XU11	575594
C7Y2H0	575596
C7Y5E3	575596
C7Y8I8	565639
C7YTJ1	660122
C7ZWT7	585143
C7ZX01	585143
C8A3M5	585145
C8A3T8	585145
C8ABK4	585146
C8ABR7	585146
C8AKL4	585153
C8AKS7	585153
C8ARM0	585158
C8ART3	585158
C8JTE8	393124
C8JVY8	393124
C8JYS1	393124
C8K0L6	393125
C8K2I5	393125
C8K780	393125
C8K859	393128
C8K8R7	393128
C8KG31	393128
C8KPG7	452948
C8KPZ0	455227
C8KW85	455227
C8KX06	591023
C8L3W2	553565

C8L4M6	553565
C8LBA3	553567
C8LCL8	553567
C8LK20	553568
C8LK85	553568
C8LVE9	553571
C8LVL4	553571
C8M2X6	553573
C8M340	553573
C8M5N3	553581
C8M626	553581
C8ME57	553583
C8MHF8	553583
C8MKH2	553588
C8MKP0	553588
C8MVR6	553592
C8MXL6	553592
C8N5H3	553596
C8N5N7	553596
C8N9W5	638300
C8NE69	638301
C8NR81	196164
C8NXJ0	585529
C8P7Q6	525309
C8PLG7	553220
C8PWG0	553217
C8QG07	592316
C8RV45	525262
C8S594	371731
C8SVB8	536019
C8TMR7	573235
C8TZV3	585395
C8UKT1	585396
C8V9C0	227321
C8VZ36	485916
C8W4M1	485916
C8WA34	521095
C8WEG6	622759
C8WMF6	479437
C8WVY7	521098
C8WWK3	521098
C8X3J2	485915
C8X8D2	479431
C8Z756	643680
C9A058	565653
C9A556	565655

C9ATP2	565652
C9CHB9	565654
C9CWE0	644076
C9CZ91	644076
C9KIU3	500635
C9KRP8	483215
C9L843	537007
C9L8F7	537007
C9LKK5	626522
C9LL43	592028
C9LTP2	546271
C9MKZ8	649761
C9MVL8	634994
C9MVL9	634994
C9MWZ5	634994
C9NH17	591167
C9NZT0	675814
C9P2H6	675813
C9P2V7	675813
C9P8C3	675813
C9P9I5	675811
C9P9V3	675811
C9PHS5	675811
C9PN34	667128
C9PZB2	619693
C9Q268	675810
C9Q2M6	675810
C9QAC6	675810
C9QF32	675816
C9QMF5	675816
C9QV55	536056
C9RCP5	429009
C9RE81	579137
C9RQY6	59374
C9RVY1	544556
C9S0D9	544556
C9T3Z2	520459
C9TCW1	520460
C9TP82	520463
C9TVL3	520461
C9U5Y5	520454
C9UF58	520452
C9UPE5	520451
C9UUD8	520450
C9VBY7	520456
C9VIA1	520457

C9VW43	520455
C9WYL4	604162
C9XCA5	568708
C9XJR9	645462
C9XPI4	645462
C9Y1H9	413502
C9YAV2	667019
C9YKR1	645463
C9YN02	645463
C9Z3Y3	680198
D0AY26	575591
D0B3C6	224914
D0BER3	520488
D0BL62	626369
D0BYI0	575564
D0CE96	575584
D0CM52	166314
D0CWV3	644107
D0D7X0	501479
D0D8J7	501479
D0DEW4	575597
D0DHK9	575597
D0DRF7	575599
D0FWR8	79967
D0GD28	520465
D0GJX6	596323
D0GTI7	675806
D0GUJ3	675806
D0GYU1	675806
D0H312	675807
D0H754	675807
D0H9B1	675807
D0HBX4	675820
D0HCA3	675820
D0HCR1	675820
D0HKH8	675808
D0HM52	675808
D0HNV2	675808
D0HZF4	675809
D0I571	675812
D0I9M7	675812
D0ICZ1	675815
D0IDL6	675815
D0ITS5	290847
D0J2I6	688245
D0JIU6	637382

D0JSY0	637385
D0K0U4	684950
D0K4Q1	681288
D0K4W8	681288
D0KAM2	561231
D0KTK5	555311
D0KXP8	555778
D0LDH5	526226
D0LLE6	502025
D0LME8	502025
D0LQD5	502025
D0LU72	502025
D0LXY0	502025
D0M309	150340
D0M6H7	150340
D0M7Q5	150340
D0MH40	518766
D0P977	520489
D0PLV8	520487
D0R4B8	633699
D0RK46	437701
D0RWG5	469609
D0RZQ6	575585
D0SC44	575586
D0SMN4	575587
D0STI3	575588
D0T248	575589
D0TCN3	469589
D0TGT1	469589
D0TW94	469588
D0W0K7	546262
D0W935	546265
D0WJW1	649743
D0WUF9	674977
D0WXR1	674977
D0X0X5	674977
D0X0X6	674977
D0X7S5	673519
D0XAC1	673519
D0XI13	673519
D0XIR7	633149
D0YP70	596328
D0YW37	675817
D0Z1R7	675817
D0Z4G7	675817
D0Z9V8	498217

D0ZR31	588858
D0ZYW9	406984
D1A888	471852
D1ANM9	526218
D1ANQ2	526218
D1ATW7	574556
D1AYU1	519441
D1B2S7	525898
D1B629	525903
D1BKF9	446469
D1BN50	479436
D1BUT0	446471
D1C8E5	479434
D1CFQ4	525904
D1CTZ4	520449
D1D5C5	528346
D1D8Y1	528352
D1DIQ2	528354
D1DNK3	528356
D1DVH0	528355
D1E277	528358
D1E8J0	528359
D1EEZ6	528360
D1ENS9	520462
D1F025	520464
D1F506	520466
D1FG80	520458
D1GTM3	663951
D1GU90	663951
D1JRI7	469587
D1JTE8	469587
D1K0H2	457391
D1NJH5	492476
D1NVW8	561180
D1NVW9	561180
D1P754	500637
D1P965	537011
D1PMY0	411471
D1PV65	585502
D1Q7K9	553594
D1QDL4	553594
D1QLD8	553601
D1QLX4	553601
D1QMU5	649760
D1QX06	553574
D1QX75	553574

D1R4Q1	159254
D1RG75	638315
D1RUY2	682634
D1S8Q3	644283
D1T2T9	643561
D1TXH8	687916
D1U5I0	643562
D1UI81	640510
D1V9Y3	298654
D1W171	679189
D1W583	679190
D1WNM8	596317
D1WNU7	596317
D1WRU2	649189
D1XKT9	647653
D1XXR8	553171
D1Y8A5	352165
D1YEB6	679194
D1YIS3	679196
D1YRU0	686660
D1Z1L3	304371
D2ADZ7	591020
D2BCV9	479432
D2BJW1	311424
D2BPM0	684738
D2BXE2	590409
D2C3S2	590168
D2ERT1	563037
D2EVT8	585543
D2F0K0	585543
D2F6N7	585149
D2F6V1	585149
D2FCT3	585151
D2FCZ7	585151
D2FLK8	585152
D2FLS8	585152
D2FV18	585159
D2FV82	585159
D2G0Z0	585160
D2G1Q4	585160
D2G9E1	585161
D2GAK3	585161
D2GFW9	585148
D2GG34	585148
D2GR78	585150
D2GRE3	585150

D2LFQ0	648757
D2LSC0	649639
D2LTI3	649639
D2M012	649639
D2M2Y9	652103
D2MHF7	700750
D2MK45	700750
D2MS67	683082
D2MX53	683083
D2N6V5	523796
D2N720	523796
D2NER3	431946
D2NPN8	680646
D2NXQ8	653938
D2P1E4	653938
D2P261	653938
D2P480	637381
D2P4Z7	637381
D2P933	637381
D2PK17	425944
D2PUM8	479435
D2Q7U0	401473
D2Q7U1	401473
D2QRU9	504472
D2QS15	504472
D2R4Y9	530564
D2RHJ7	572546
D2RKF3	591001
D2RSU3	543526
D2S669	526225
D2T8N4	644651
D2TSP0	637910
D2TWH3	638
D2ULU5	585155
D2UM14	585155
D2URU8	585147
D2US11	585147
D2Y941	671074
D2YHU1	671074
D2YJ96	671074
D2YU32	671076
D2YUG1	671076
D2YUX7	671076
D2Z763	469381
D2ZHN1	500639
D2ZQ42	521002
D2ZZE0	546266
D3A2Z7	546268

D3AK14	566550
D3C1C0	653733
D3C955	648999
D3CZI4	102897
D3DGF2	608538
D3DLV4	559292
D3DYX2	634498
D3E6K6	481743
D3E6N5	481743
D3EQ47	713887
D3EW70	703339
D3EWD5	703339
D3FFA3	469383
D3FKA2	567106
D3FTZ1	398511
D3FWG1	398511
D3FZ68	398511
D3GT05	216592
D3HAM9	365659
D3HH74	637909
D3HRC9	661367
D3HZA6	575611
D3I625	575612
D3I9Y3	575614
D3IG36	575615
D3KMP3	393121
D3KQ01	393121
D3KQ95	393121
D3L1V0	592015
D3L2V5	592015
D3LB41	655225
D3LPM3	596312
D3LUC6	699218
D3M4R6	656024
D3MEQ6	679195
D3MMX6	686659
D3MYQ8	547146
D3NA37	640512
D3NNU3	663278
D3NQS3	137722
D3PDH4	639282
D3PQK8	504728
D3Q5Q9	446470
D3QCX4	698737
D3QD59	698737
D3QK31	701177
D3R269	699246
D3R3B3	552531
D3R3B4	552531
D3RG76	640131
D3RRY6	572477
D3RWV9	589924
D3S5V6	644281
D3SAQ5	396595

D3SKS4	633145
D3SPA2	638303
D3SV98	547559
D3T907	580331
D3UFY6	679897
D3UMD5	683837
D3UN23	683837
D3UR60	683837
D3UUH6	634464
D3V642	406818
D3VBY0	406817
D4AXF4	663331
D4B8P4	500640
D4BKW4	500640
D4BLU7	518634
D4BLU8	518634
D4BZT7	521000
D4CH20	411486
D4CHG2	411486
D4CQB1	608534
D4DL49	663202
D4DTA9	546263
D4E0J1	667129
D4EIK8	699185
D4EX33	699186
D4F073	500638
D4FCL6	679188
D4FGQ8	679188
D4FIV2	525375
D4FJC8	525375
D4FRA1	655813
D4FWS5	645657
D4FZS2	645657
D4G551	645657
D4GH99	706191
D4GYK3	309800
D4H838	522772
D4HB04	553199
D4I2T1	665029
D4ID84	716540
D4IYV9	657324
D4J5I5	717962
D4JME0	657317
D4JVY5	657319
D4K3V9	718252
D4KBU6	657322
D4KID4	657316
D4KLF7	657315
D4L4Q9	657321
D4LAL9	213810
D4LJF8	657323
D4LKL9	657323
D4LQD9	657314
D4LXX7	657314

D4M5L9	657313
D4M5X0	657313
D4MAZ8	651822
D4MJG1	717961
D4MQ89	245012
D4MYN6	245018
D4PJ42	393117
D4PK82	393117
D4PQJ4	393117
D4PT73	393131
D4PTT7	393131
D4PZH6	393131
D4Q521	401650
D4Q5T2	401650
D4Q7D3	401650
D4RZA5	511680
D4S8N3	585503
D4SH06	585531
D4TEP6	533240
D4TQ88	533247
D4U0C1	649742
D4U3K5	553590
D4UAC5	553590
D4UEN9	553580
D4UEW1	553580
D4UR74	246199
D4V056	791166
D4V6J4	702446
D4VT99	702447
D4W5Y2	702450
D4WL27	702443
D4WSX4	702444
D4XGB6	742159
D4XPC5	707232
D4XZF5	552811
D4Y3U2	634956
D4Y4Q1	634956
D4Y861	634956
D4YEG4	655812
D4YMN5	585530
D4YRH6	585524
D4Z3Y4	452662
D4ZC21	637905
D4ZT24	696747
D5AJS2	423211
D5AMP7	272942
D5AY21	449216
D5B4I1	637386
D5BEE1	655815
D5BEU4	655815
D5BMX7	488538
D5BZJ7	472759
D5C3W2	472759
D5CBP5	716541

D5CS07	580332
D5D6M2	714962
D5D8H3	641892
D5DFE2	592022
D5DKV9	592022
D5DLR0	592022
D5DQ49	545693
D5DR12	545693
D5DTQ3	545693
D5E9A3	547558
D5EC81	572547
D5EPB1	583355
D5EXM1	264731
D5GY22	748671
D5H2T5	748671
D5H627	761659
D5HJ58	751585
D5MES8	671143
D5MZ24	703612
D5N131	703612
D5N4B0	703612
D5NF78	243261
D5NVD5	649754
D5P918	525368
D5PKB1	525370
D5Q3R3	525259
D5Q5X2	525259
D5QFW3	714995
D5QME0	595536
D5R0H6	642492
D5RJC4	525371
D5RZW9	525258
D5S2N4	525258
D5S9Q9	760570
D5SDJ1	762948
D5SVI9	521674
D5T4P6	762051
D5TE68	423212
D5TFC0	573236
D5TFC1	573236
D5TUT5	714359
D5TWJ2	714359
D5UAA4	526224
D5ULP5	446466
D5UN58	521096
D5UZ64	572480
D5V1F3	572480
D5VB02	749219
D5VEY2	509190
D5VRZ1	573063
D5VXB1	758678
D5VZE9	758678
D5W207	758678
D5W8Y7	640511

D5WPL9	562970
D5WUK5	562970
D5X513	75379
D5XAE4	635013
D5XF36	635013
D5XNL3	515617
D5Y9W1	520141
D5YLF9	520140
D5YXY2	515616
D5ZAT4	537209
D5ZMQ5	537210
D6A946	566461
D6AV84	457431
D6AWB1	457425
D6CRF5	426114
D6D486	657309
D6DAM2	722911
D6DDK3	717608
D6DPS3	718254
D6E0T6	657318
D6E7I6	657308
D6EPL3	457428
D6FTX4	611304
D6GM48	469608
D6GPY6	546269
D6GYL8	585156
D6GYT2	585156
D6H8I6	528348
D6HGI0	585144
D6HGP3	585144
D6I3R4	550672
D6IGW4	550676
D6IIJ8	656380
D6IZZ3	585157
D6J056	585157
D6J510	550677
D6JMF3	528351
D6JRB7	575565
D6K920	645465
D6KIB4	457416
D6KNG9	450749
D6KVP7	641146
D6L1X8	641147
D6LQ93	520448
D6LXH3	585154
D6LXN7	585154
D6MHI9	536227
D6RYY3	525284
D6RYY4	525284
D6SH18	548470
D6SH82	548470
D6SQU7	555779
D6SWP1	682147
D6T1A1	682148

D6T481	553577
D6T4M5	553577
D6UAM5	762962
D6UAU1	762962
D6UKS3	585535
D6UYZ5	682795
D6V0F7	666684
D6VNV5	596153
D6XRA1	637913
D6XSX5	439292
D6XU18	439292
D6Y1G2	439292
D6YAG3	469371
D6YDC6	707184
D6YFG9	707185
D6YI35	707186
D6YKR0	707187
D6YMH4	707183
D6YVR7	716544
D6YY46	718219
D6Z1T7	589865
D6ZC84	640132
D6ZJS1	548479
D6ZQP3	525381
D6ZXJ4	759350
D6ZXJ5	759350
D7A5C4	639283
D7AJN0	663917
D7AP26	583358
D7B0A9	446468
D7BFP1	526227
D7C517	749414
D7CNE5	643648
D7CUW5	649638
D7CZH9	691437
D7D4H0	691437
D7DDI4	759363
D7DGV9	759364
D7DL85	666681
D7DTR1	456320
D7DXE1	551115
D7EBL4	644295
D7EX22	515615
D7FCA6	693745
D7FXQ9	2880
D7GIJ9	754252
D7GZZ1	520453
D7HC03	345074
D7HNC1	412967
D7HS58	412967
D7HSI2	412967
D7I350	693985
D7IF76	469585
D7ING2	469592

D7IS38	469592
D7J440	585544
D7JI36	656379
D7K2K8	457390
D7L9I8	81972
D7M587	81972
D7M5Z6	81972
D7N0C8	641149
D7NAV1	563008
O23653	3702
O25827	210
O26893	187420
O29558	2234
O58588	53953
O58813	53953
O60163	4896
O67221	63363
O69077	287
O84367	813
P08495	1423
P0A4Z8	1773
P0A4Z9	1765
P10869	4932
P26512	1718
P41398	28028
P41403	1772
P53553	1422
P61488	262724
P61489	274
P74569	1148
P94417	1423
Q01GS4	70448
Q01R25	234267
Q02I64	208963
Q030N1	272622
Q036H1	321967
Q03CV9	321967
Q03HS8	278197
Q03M59	322159
Q03Y29	203120
Q043C1	324831
Q04795	1423
Q049T8	321956
Q04DS2	203123
Q04M61	373153
Q04V37	355277
Q04YE0	355276
Q05FN2	387662
Q05UL7	221359
Q062N7	313625
Q07VB5	316055
Q088B9	318167
Q08PK2	378806
Q08XR7	378806

Q0A8K8	187272
Q0AEC6	335283
Q0AME2	394221
Q0AQP6	394221
Q0AXC9	335541
Q0BDV8	339670
Q0BUK4	391165
Q0C1P2	228405
Q0C4J6	228405
Q0CA14	341663
Q0EYM5	314345
Q0F0P2	314345
Q0F3Y6	367336
Q0FI20	314265
Q0FMZ9	314265
Q0G7Y6	314231
Q0HL59	60480
Q0HMI4	60480
Q0HRA4	60481
Q0HXF7	60481
Q0IE09	64471
Q0KCA5	381666
Q0KJ33	68570
Q0PAT5	197
Q0RN33	326424
Q0RT24	326424
Q0S8Q4	101510
Q0SSE2	289380
Q0SXP2	373384
Q0TA37	362663
Q0TPS5	195103
Q0UFQ9	13684
Q0VNK3	393595
Q0W3M8	351160
Q0YTF2	377431
Q10AJ5	39947
Q11A81	203124
Q11E02	266779
Q11NQ9	269798
Q129L7	296591
Q12JW6	318161
Q12UL7	259564
Q13F00	316057
Q13X64	266265
Q15QA7	342610
Q167U1	375451
Q17VH3	382638
Q188C0	272563
Q18BI5	272563
Q18DI7	362976
Q1AX22	266117
Q1AZV0	266117
Q1B291	164756
Q1BHN7	331271

Q1CC28	360102
Q1CNS5	377628
Q1CS34	357544
Q1D2T9	246197
Q1D6R0	246197
Q1G9N2	390333
Q1GF99	292414
Q1GJ79	292414
Q1GNW4	117207
Q1GR69	117207
Q1H3V0	265072
Q1I6Z9	384676
Q1INQ8	204669
Q1IRL6	204669
Q1IZB0	319795
Q1JY31	281689
Q1LPF1	266264
Q1MBB0	216596
Q1MPM0	363253
Q1MZC2	207949
Q1N045	207949
Q1NBF6	314266
Q1NEV2	314266
Q1NSC0	262489
Q1Q5R5	174633
Q1QA87	335284
Q1QQY5	323097
Q1QZX0	290398
Q1R3R4	364106
Q1RGM9	336407
Q1V1S8	314261
Q1V9B4	314288
Q1VDX5	314288
Q1VZ05	313595
Q1WT92	362948
Q1YGE6	287752
Q1YHV7	287752
Q1YR95	314287
Q1Z1U0	314280
Q1Z482	314280
Q1Z755	314280
Q1ZDH4	314282
Q1ZN91	314292
Q1ZNV7	314292
Q21BZ7	316056
Q21L67	203122
Q21LH5	203122
Q21YR6	338969
Q24UK4	138119
Q24VS6	138119
Q255G1	264202
Q26E20	156586
Q28MF4	290400
Q2B0K5	313627

Q2B6P8	313627
Q2BCH7	313627
Q2BGE5	207954
Q2BQV4	207954
Q2C0E2	121723
Q2C126	121723
Q2CDC1	314256
Q2CFH7	314256
Q2FT50	323259
Q2FYP1	93061
Q2FYV5	93061
Q2G334	279238
Q2GFJ5	205920
Q2GXN2	38033
Q2IFJ6	290397
Q2IIV3	290397
Q2J421	316058
Q2JB98	106370
Q2JGE2	106370
Q2JPR1	321332
Q2JTT1	321327
Q2K3S9	347834
Q2KX43	360910
Q2LTK0	56780
Q2N5P0	314225
Q2NHW4	339860
Q2NR03	343509
Q2RIX2	264732
Q2RJK8	264732
Q2RWE8	269796
Q2S1R8	309807
Q2S5A5	309807
Q2SBU0	349521
Q2SX74	271848
Q2UTJ9	5062
Q2W6V9	342108
Q2Y519	115547
Q2Y7N5	323848
Q2YLN6	359391
Q2YXT9	273036
Q2YXX6	273036
Q30Q04	326298
Q30ZQ1	207559
Q317Z4	74546
Q31F92	317025
Q31IA6	317025
Q31PI8	1140
Q31TX2	300268
Q328X6	300267
Q39EZ2	269483
Q39UG5	269799
Q3A5V0	338963
Q3AAV6	246194
Q3ACY4	246194

Q3ANI2	110662
Q3AP04	340177
Q3B0T2	316279
Q3B0T5	316279
Q3B179	319225
Q3CZ22	342615
Q3DA78	342616
Q3DEV1	342617
Q3DJR2	342614
Q3DPN8	342613
Q3DT23	342613
Q3EXH1	339854
Q3IKT4	326442
Q3ILF2	326442
Q3IU17	348780
Q3J5D6	272943
Q3JCA9	323261
Q3JCK7	323261
Q3JQU0	320372
Q3K893	205922
Q3KLZ4	315277
Q3M6Y8	240292
Q3SK68	292415
Q3SVP5	323098
Q3YR16	269484
Q3YUW1	300269
Q3Z622	243164
Q3ZZW8	255470
Q46133	1718
Q46E57	269797
Q46IE2	59920
Q473C9	264198
Q47D33	159087
Q47TY3	269800
Q483N2	167879
Q48G26	264730
Q49XB4	342451
Q49XJ5	342451
Q4BZN9	165597
Q4EAE6	307502
Q4EEU1	267410
Q4EIP5	267410
Q4ENE2	267409
Q4ESL2	267409
Q4ETU2	267409
Q4FNA1	198252
Q4FRP9	259536
Q4HH89	306254
Q4HTE4	306264
Q4J8Y8	2285
Q4JQJ2	95640
Q4JSM7	306537
Q4K844	220664
Q4L636	279808

Q4L698	279808
Q4MK97	269801
Q4MUL1	269801
Q4PG91	5270
Q4UK84	42862
Q4WTP2	5085
Q4ZQI5	205918
Q55N96	5207
Q57991	2190
Q57B20	235
Q57H07	28901
Q59229	126782
Q59ME9	5476
Q5A474	5476
Q5AS71	162425
Q5DYF1	312309
Q5E7G4	312309
Q5E813	312309
Q5F842	242231
Q5FGC6	302409
Q5FJS4	1579
Q5FKR3	1579
Q5FUT5	442
Q5GSJ5	292805
Q5HAA9	254945
Q5HG27	93062
Q5HG94	93062
Q5HPE9	176279
Q5HPL5	176279
Q5HVI8	195099
Q5JH52	311400
Q5JK18	39947
Q5KBM8	5207
Q5KV06	1462
Q5L0H5	1462
Q5L5G6	83555
Q5LBE0	272559
Q5LHU9	272559
Q5LP17	89184
Q5M188	299768
Q5M5S5	264199
Q5N4N5	269084
Q5NLY3	542
Q5P8A6	76114
Q5P9Z2	234826
Q5QUY3	135577
Q5SKV9	300852
Q5UF02	295349
Q5UZ62	2238
Q5WFV5	66692
Q5WH57	66692
Q5X4A6	297246
Q5Z331	37329
Q5ZUI7	272624

Q60BS5	414
Q62J41	13373
Q636M6	288681
Q63D00	288681
Q63ST2	28450
Q64CS2	290244
Q64D96	285393
Q64RT5	817
Q64YV1	817
Q65GF1	279010
Q65JG8	279010
Q65NE6	279010
Q65S40	221988
Q664W8	633
Q67NS2	2734
Q67P60	2734
Q68VZ7	785
Q6A5V8	1747
Q6AGY4	59736
Q6AR44	84980
Q6BUX0	4959
Q6C9F1	4952
Q6CID1	28985
Q6D020	29471
Q6FCT3	62977
Q6FPL5	5478
Q6G0I0	803
Q6G5K2	38323
Q6G9G8	282459
Q6G9N1	282459
Q6GH15	282458
Q6GH79	282458
Q6KZ99	263820
Q6LH77	74109
Q6LM79	74109
Q6LMU4	74109
Q6LYH4	39152
Q6MD60	264201
Q6MQE3	959
Q6MRF3	959
Q6NC68	1076
Q6NJW6	1717
Q6SHD3	257390
Q6SHR1	257386
Q6YS32	39947
Q6YS33	39947
Q71X54	265669
Q71ZN4	265669
Q720J4	265669
Q72AS8	882
Q732S3	222523
Q73A99	222523
Q73GJ2	163164
Q744N0	1770

Q74C75	35554
Q75AL3	33169
Q76E53	17
Q7A0Z4	196620
Q7A5P9	158879
Q7A5T7	158879
Q7CLD9	632
Q7M8C7	844
Q7MHB2	196600
Q7MHR7	196600
Q7MT13	837
Q7MZB3	141679
Q7NJQ8	33072
Q7NZA4	536
Q7P9R4	272951
Q7UA30	84588
Q7UMB6	265606
Q7UZL4	59919
Q7V983	74547
Q7V9M2	1219
Q7VII7	32025
Q7VLP7	730
Q7VX91	520
Q7W858	519
Q7WLK6	518
Q819Z8	226900
Q81F53	226900
Q81S62	1392
Q81WN6	1392
Q822G9	83557
Q82EQ5	33903
Q82T09	915
Q838R7	1351
Q83CQ7	777
Q83FL3	203267
Q83HB4	218496
Q83IN8	623
Q851Z6	39947
Q852E4	39947
Q87L96	670
Q87LR4	670
Q87NZ9	670
Q88EI9	160488
Q88UY8	1590
Q88Y22	1590
Q891L5	1513
Q895J1	1513
Q8A7Z9	818
Q8CSM8	176280
Q8CSQ3	176280
Q8DC48	672
Q8DCJ6	672
Q8DHW0	197221
Q8DR20	171101

Q8DSM8	1309
Q8EAC1	70863
Q8EBS2	70863
Q8EQ50	182710
Q8EQT0	182710
Q8F865	173
Q8FB45	217992
Q8FYK1	29461
Q8G6Y6	216816
Q8G6Y7	216816
Q8GIQ5	159599
Q8KG73	1097
Q8KQ28	1901
Q8KQ29	1901
Q8NIW0	5141
Q8NWS7	196620
Q8PX05	2209
Q8RA48	119072
Q8RQN1	152794
Q8RU02	406
Q8TUD6	2214
Q8TZ30	2320
Q8U200	2261
Q8U201	2261
Q8XJS6	1502
Q8Y073	305
Q8Y4R0	1639
Q8Y765	1639
Q8Y7N9	1639
Q8YJ97	29459
Q8YR14	103690
Q8ZUA3	13773
Q928Q9	1642
Q92BR9	1642
Q92CH4	1642
Q92GF9	781
Q92MK3	382
Q93C54	168810
Q93C55	168810
Q93C56	168810
Q93C57	168810
Q93TX4	41
Q971Y7	111955
Q97I43	1488
Q97MC0	1488
Q97SF8	1313
Q97ZL7	2287
Q98G91	381
Q99U91	158878
Q99UE9	158878
Q9A9W8	155892
Q9AE84	1913
Q9AE85	1913
Q9CB77	1769

Q9CHJ1	1360
Q9CM97	747
Q9EV19	375
Q9EV36	1280
Q9EZA8	542
Q9HL67	2303
Q9HMV1	2242
Q9K8B0	86665
Q9KA90	86665
Q9KCR9	86665
Q9KLC4	666
Q9KUH4	666
Q9KUW8	666
Q9LYU8	3702
Q9PK32	83560
Q9RQ24	33910
Q9RQ25	33910
Q9RUL9	1299
Q9S702	3702
Q9UZV5	29292
Q9UZV6	29292
Q9X1K6	2336
Q9XAI7	1902
Q9XHC5	3847
Q9YCX2	56636
Q9Z6L0	83558
Q9ZCI7	782
Q9ZJZ7	85963

**homoserine dehydrogenase**		**homoserine O-acetyltransferase**		**cystathionine gamma-synthase**	
**UniProt**	**TXID**	**UniProt**	**TXID**	**UniProt**	**TXID**
P31116	4932	P08465	4932	A1C3X3	5057
D6VWV8	559292	D6W0R8	559292	A1CBW5	5057
C8ZBT8	643680	C8ZGP5	643680	A1CG82	5057
C7GM73	574961	C7GP87	574961	A1CJ22	5057
B3LQM1	285006	B3LPB5	285006	A1D8F6	331117
A6ZQ95	307796	A6ZRG8	307796	A1D936	331117
B5VLS9	545124	Q65C71	4931	A1DB44	331117
C5DRI7	559307	Q7ZA31	4931	A1DDE0	331117
Q75DV3	33169	Q65C69	27292	A2QA31	425011
Q6BN01	4959	Q65C70	27292	A2QIU2	425011
A3LRB1	4924	Q06736	27292	A2QY34	425011
A5DL31	4929	Q65C68	230603	A2QYG1	425011
A5DW64	36914	Q6FNX4	5478	A2R8X8	425011
C4QXA7	644223	C5DYZ8	559307	A2Z702	39946
C4Y071	306902	B2G456	4956	A2Z703	39946
C9STI6	526221	A7TF85	436907	A2Z705	39946
Q0UIN5	13684	C5DK61	559295	A3LSH7	4924
C7ZBU6	660122	Q6CUV3	28985	A3LTQ0	4924
C7Z8D8	660122	Q751Z3	33169	A3LX62	4924

C7Z5E0	660122	Q6C7H3	4952	A4HMZ0	5660
Q2GS85	38033	C9W9H0	4905	A4IBL4	5671
D5GJK0	39416	Q96UK3	5141	A4QRH4	148305
B8NT37	332952	Q1K8F9	5141	A4RMG5	148305
B8N1N2	332952	Q5MCQ5	5191	A4RNB9	148305
Q2UUX3	5062	P12917	5191	A4S2B4	436017
Q2UKP9	5062	Q5FY74	4922	A4S3Y0	436017
O94671	4896	C4R960	644223	A5DN62	4929
B8M599	441959	B2ASE7	5145	A5DVE9	36914
Q4WY36	5085	C7YYD0	660122	A5E6Z6	36914
B0XXC0	451804	Q2U8Z3	5062	A6R1N2	339724
B6Q4L3	441960	Q9Y875	162425	A6R497	339724
Q5B998	162425	C8VMS1	227321	A6RIC4	332648
C8VJA1	227321	B8NDD4	332952	A6S4T4	332648
C5G7G1	559297	Q0CAF3	341663	A6SDV0	332648
B6K3G8	402676	A1CA53	5057	A6ZLY2	307796
B6H4I9	500485	B8Y203	35717	A6ZQ85	307796
Q96VZ7	5207	Q4WU51	5085	A7A0F7	307796
Q5KAD9	5207	B0Y3M8	451804	A7A0K2	307796
A4S2Z7	436017	A1DFA6	331117	A7E8S4	665079
B0DNV1	486041	A4RKK0	148305	A7EFY4	665079
O63067	3847	A3LR19	4924	A7F083	665079
B9IQ92	3694	A2R9A0	425011	A7TG47	436907
A5APD8	29760	Q2H2M9	38033	A7TP41	436907
C1MP14	564608	B6Q1S6	441960	A7WPD7	51657
D7MG88	81972	D1ZDE4	5147	A8J355	3055
D7KK95	81972	B8MR86	441959	A8NBT0	240176
C5X893	4558	C5JN63	559298	A8PQX0	425265
A9SKI1	145481	Q0U2Y1	13684	A9PID6	3694
A9TAK5	145481	C5GC66	559297	A9S5H9	145481
D3BT16	670386	C0NQJ9	447093	A9T1Q4	145481
A1CKR4	5057	C5FW90	554155	B0DAR0	486041
A1D6Q9	331117	C6HSC8	544712	B0DFR8	486041
A2QDK6	425011	A7EBN8	665079	B0DR59	486041
A4H4X1	5660	B6HNU8	500485	B0DR66	486041
A4HT44	5671	D4D3Z4	663202	B0EJN7	370354
A4RDD3	148305	A6QVN0	339724	B0ELR1	370354
A6S8E9	332648	Q6BN89	4959	B0EN45	370354
A7F9Z3	665079	O60062	4896	B0XZ86	451804
A7TLG4	436907	C5P7A4	222929	B0Y277	451804
A8DWA3	45351	D4AKG0	663331	B0YA29	451804
A8NBZ4	240176	C4JNA0	336963	B0YBX6	451804
A8Q3I6	425265	B6JVI4	402676	B1N417	5759
B2AR90	5145	B9WL30	573826	B2AUC6	5145
B9W745	573826	A5DNC0	4929	B2AYE2	5145
C4JZ21	336963	B0DA11	486041	B2G4B3	4956

C5DFS1	559295	Q2HZ32	5480	B2LXT6	162425
C5MEY6	294747	D5GD22	39416	B2VLA4	515849
Q4PDV5	5270	Q5A948	5476	B2VXP0	426418
Q4QIR8	5664	C1H1Y9	502779	B2WCB8	426418
Q5AIA2	5476	A8N0C2	240176	B3LLH3	285006
Q6CAP2	4952	A6STB1	332648	B3LQL2	285006
Q6CWI3	28985	C1G658	502780	B3LTC0	285006
Q6FK85	5478	C5M4B1	294747	B3LUU9	285006
Q7RZC2	5141	Q5K8P2	5207	B3MDE6	7217
A0SXJ9	4932	B2WIA3	426418	B3NK88	7220
A6QRK8	339724	Q4P8Y8	5270	B3S3W7	10228
B2WEK6	426418	C9S846	526221	B4F935	4577
C0NUY8	447093	Q7LLJ3	5044	B4FJ26	4577
C4YF11	5476	P39058	5044	B4FNW6	4577
C5JUC3	559298	C0RXV2	482561	B4HNZ0	7238
C5P9N4	222929	C1E6A7	296587	B4P4B7	7245
C6HRL1	544712	C1DZ30	296587	B4QD03	7240
Q0CNN4	341663	C4Y791	306902	B5VDL1	545124
		C1N2Q8	564608	B5VMP7	545124
		A5DRU6	36914	B5VP46	545124
		D0NF03	403677	B6HAD5	500485
		Q019D4	70448	B6HEW5	500485
		A8Q4H2	425265	B6HMV3	500485
		B7GCV4	556484	B6K3B8	402676
		A1CAJ8	5057	B6K8Y1	508771
		A1CNS2	5057	B6QM86	441960
		A1D1N4	331117	B6QTJ7	441960
		A1DEX1	331117	B6QUU5	441960
		A2QB20	425011	B7GC92	556484
		A3LRS6	4924	B8AQ99	39946
		A4RAE8	148305	B8B4G5	39946
		A5DRD9	4929	B8CCE6	35128
		A5E1K9	36914	B8LL39	3332
		A6S9Y5	332648	B8MDX9	441959
		A7EDB6	665079	B8MNR4	441959
		A7EQ10	665079	B8N392	332952
		A7TBR2	45351	B8N7B3	332952
		A8NX22	240176	B8NK20	332952
		A8QBJ7	425265	B8NV95	332952
		A9URI4	81824	B8NYK4	332952
		A9V9I0	81824	B9F8N9	39947
		B0CXX6	486041	B9IK07	3694
		B2ATF5	5145	B9PIG1	5811
		B6GW62	500485	B9Q9E9	432359
		B6HRJ0	500485	B9W7F1	573826
		B6KPQ7	508771	B9WMM0	573826

B8CGK6	35128	B9WMS1	573826
B8MZD4	332952	C0NB75	447093
B8NKM7	332952	C0NZA4	447093
B9W9G2	573826	C0S0K0	482561
C3YTV6	7739	C0SD44	482561
C4JLJ3	336963	C0Z354	3702
C4JY13	336963	C1EHF0	296587
C4R0I1	644223	C1GAS0	502780
C4YC90	306902	C1GF82	502780
C5DHB5	559295	C1H7U4	502779
C5DPX0	559307	C1HDT5	502779
C5MBP3	294747	C1MNK0	564608
C8VET6	227321	C4JJ69	336963
C8VPZ5	227321	C4JWP3	336963
Q0U5T8	13684	C4MAS9	5759
Q0UEF0	13684	C4QYL9	644223
Q0V150	13684	C4R1M9	644223
Q10341	4896	C4R3L3	644223
Q2U6J5	5062	C4Y2M1	306902
Q2UQM7	5062	C4Y8G1	306902
Q4P1G6	5270	C4YBC9	306902
Q4WRR7	5085	C4YEQ7	5476
Q4WUI7	5085	C5DFU1	559295
Q55SA6	5207	C5DG64	559295
Q59TT5	5476	C5DJ03	559295
Q5KGQ3	5207	C5DLF9	559295
Q6CAU6	4952	C5DRG8	559307
Q6CJ47	28985	C5DY28	559307
Q757W0	33169	C5DYT7	559307
Q7SDK8	5141	C5FSN0	554155
A2QTP2	425011	C5G096	554155
A4QQH5	148305	C5GQ38	559297
A6QTK6	339724	C5GR68	559297
A6R0Z4	339724	C5JR79	559298
B0XN80	451804	C5JRR5	559298
B0Y412	451804	C5KHB3	423536
B2WHD7	426418	C5KL47	423536
B2WJG2	426418	C5LFG5	423536
B2WJM7	426418	C5MAJ9	294747
B6K6H3	402676	C5ME56	294747
B6QEF8	441960	C5MIQ7	294747
B8LWJ3	441959	C5P1R8	222929
B8MD92	441959	C5PEF2	222929
B9QIH4	432359	C5WYM5	4558
C0NQG2	447093	C6H9G8	544712
C0P0M1	447093	C6HP80	544712

C0S7T0	482561
C0SFX7	482561
C4YCP2	5476
C5FBM9	554155
C5FH78	554155
C5GC45	559297
C5GJL0	559297
C5JER7	559298
C5JN65	559298
C5P566	222929
C5PAM2	222929
C6HEZ1	544712
C6HS94	544712
C7YSW6	660122
C7Z3S1	660122
C9S9N2	526221
D4B0W4	663331
D4D358	663202
O13389	162425
Q0CKK2	341663
Q0CM27	341663
Q2KEH4	242507
Q5AT15	162425
Q5BF05	162425
Q6BVG4	4959

C7GM83	574961
C7GND7	574961
C7GPM2	574961
C7GW18	574961
C7YHE2	660122
C7YKF3	660122
C7YP17	660122
C8VHB0	227321
C8VRR3	227321
C8Z3L8	643680
C8ZBS8	643680
C8ZCR8	643680
C8ZEC2	643680
C9SXQ4	526221
C9ZZ26	679716
D1ZTJ9	5147
D2V6M2	5762
D3BFM3	670386
D4AVM8	663331
D4B279	663331
D4DDM4	663202
D4DEE8	663202
D5GJJ5	39416
D6VPK6	559292
D6VWU8	559292
D6VXV0	559292
D6W0K0	559292
D7LAJ0	81972
O04981	4577
O04982	4577
O23944	57918
O42851	4896
O74314	4896
O81334	3544
O97121	7227
P31373	4932
P32929	9606
P38675	5141
P47164	4932
P55217	3702
Q012C1	70448
Q04533	4932
Q0C8C6	341663
Q0CBE7	341663
Q0CX10	341663
Q0D1Y8	341663

Q0GC25	3879
Q0TXK2	13684
Q0TZA5	13684
Q10KP2	39947
Q10KP3	39947
Q10KP4	39947
Q12198	4932
Q2H5Y9	38033
Q2HFS9	38033
Q2HH43	38033
Q2TYY9	5062
Q2U2B2	5062
Q2U598	5062
Q2UBS4	5062
Q2UJR0	5062
Q38DH3	5691
Q4DUG8	5693
Q4FWE7	347515
Q4P760	5270
Q4WAK1	5085
Q4WCI1	5085
Q4WVZ1	5085
Q4WWD8	5085
Q55DV9	44689
Q55V77	5207
Q59VR0	5476
Q59VV6	5476
Q5A362	5476
Q5A410	5476
Q5ARD6	162425
Q5B7M4	162425
Q5BDD4	162425
Q5BFJ9	162425
Q5KL05	5207
Q5RFI1	9601
Q60HG7	9541
Q6BSE9	4959
Q6BTV4	4959
Q6BZI1	4959
Q6BZJ6	4959
Q6C2Q9	4952
Q6C8S4	4952
Q6CB42	4952
Q6CKV1	28985
Q6CMC4	28985
Q6CWG2	28985

UniProt	TXID
Q6FK75	5478
Q6FR34	5478
Q6FRS2	5478
Q6R8F6	4081
Q756K4	33169
Q756W2	33169
Q758U3	33169
Q7JXZ2	7227
Q7QHF8	7165
Q86D27	5759
Q86D28	5759
Q86ZL4	5145
Q8S7G4	4530
Q9C816	3702
Q9C876	3702
Q9MT29	4113
Q9MT30	4113
Q9P417	5044
Q9SPT6	4113
Q9XFG0	3847
Q9ZPL5	4097

**cystathionine beta-lyase**		**5-methyltetrahydropteroyltriglutamate--homocysteine methyltransferase**		**O-acetylhomoserine (thiol)-lyase**	
**UniProt**	**TXID**	**UniProt**	**TXID**	**UniProt**	**TXID**
A1C977	5057	A1CH47	5057	P06106	4932
A1CBW5	5057	A1CXP1	331117	D6VYU6	559292
A1CG82	5057	A2ZMX3	39946	C8ZDR6	643680
A1D936	331117	A3LVA0	4924	B5VNK3	545124
A1D9N1	331117	A4HIW5	5660	A7A1I7	307796
A1DDE0	331117	A4I695	5671	B3RHI1	285006
A2QIU2	425011	A4QPX4	148305	C7GVT7	574961
A2QYG1	425011	A4R296	148305	A7TLB1	436907
A2R2J2	425011	A5AAM9	425011	Q6FVR1	5478
A3LMX1	4924	A5BSS2	29760	Q92441	28985
A3LTQ0	4924	A5C7K7	29760	C5DGA4	559295
A4DA77	5085	A5DFQ0	4929	C5E1M5	559307
A4H7K6	5660	A5DS66	36914	Q75AE8	33169
A4HKQ5	5660	A6R7F7	339724	C4XWE8	306902
A4HVY9	5671	A6SHK9	332648	Q6BME7	4959
A4I885	5671	A6YGE7	3649	C5M1S7	294747
A4RHJ5	148305	A6ZR47	307796	B9WF95	573826
A4S2R2	436017	A7F002	665079	Q9UVM2	5476
A5DFM4	4929	A7RIK3	45351	Q9UR58	5476
A5DV17	36914	A7T6T8	45351	Q59US5	5476
A6R497	339724	A7TMV4	436907	A3LST3	4924
A6REG4	339724	A8NXT9	240176	C4R7J7	644223

A6RIC4	332648	A8PX29	425265	A5E3W7	36914
A6RQY9	332648	A9P805	3694	Q9C123	4922
A6ZU04	307796	A9PBM8	3694	A5DDM8	4929
A7A294	307796	A9PBU6	3694	Q6C7U9	4952
A7EFY4	665079	A9PII3	3694	Q2GNW9	38033
A7EX64	665079	A9RT52	145481	C5P7U2	222929
A7YVL5	4905	A9RWS2	145481	C4JMM4	336963
A8CEI3	3866	A9U0A5	145481	Q9P3D4	5141
A8JB47	3055	A9U742	145481	Q1K4Z1	5141
A8NU12	240176	B0CPI5	486041	B6GZ70	500485
A9SF32	145481	B0CYJ4	486041	B6HWB3	500485
B0Y277	451804	B0Y5S8	451804	A2QUT4	425011
B0YBX6	451804	B2AF53	5145	D5GKD1	39416
B0YEG1	451804	B2AWS2	5145	B2B0Q4	5145
B2B313	5145	B2VQE0	46066	C5JTC9	559298
B2WMP8	426418	B2W1D5	426418	A8Q079	425265
B3LUR6	285006	B3LRK9	285006	P50125	162425
B4FU01	4577	B4G1T4	4577	C8V3P9	227321
B4FXS6	4577	B6HV10	500485	C1GM15	502780
B5VI80	545124	B6K226	402676	C1HBF9	502779
B6H390	500485	B6QFW8	441960	Q2U9X2	5062
B6HAD5	500485	B6UF55	4577	B8NRD7	332952
B6HMV3	500485	B8A1R8	4577	C0SI30	482561
B6K4V7	402676	B8BN41	39946	B2W2J7	426418
B6QGC7	441960	B8LKF4	3332	A1C4R2	5057
B6QUU5	441960	B8LPY2	3332	C7F7S7	42251
B8MDQ7	441959	B8LQU7	3332	O13326	4896
B8MDQ8	441959	B8M9S4	441959	B0DT91	486041
B8N7B3	332952	B8N9V1	332952	A4RHY9	148305
B8NCS0	332952	B9HQI3	3694	B6QHT5	441960
B8NK20	332952	B9I615	3694	B6K2Z3	402676
B9DGA0	3702	B9IPT1	3694	A6R9L3	339724
B9FRT6	39947	B9MYM2	3694	Q0V2F1	13684
B9NJ56	3694	B9PD83	3694	A8P274	240176
B9RGV9	3988	B9RQ33	3988	A6S2U3	332648
B9WA95	573826	B9SI90	3988	D1ZA82	5147
C0NB75	447093	B9WKZ1	573826	B8MIN1	441959
C0NVJ3	447093	C0NIW4	447093	C7YWL1	660122
C0S0K0	482561	C0P5Y3	4577	Q4WEU7	5085
C0SJG2	482561	C0SIT3	482561	B0Y332	451804
C1EH62	296587	C1GLT7	502780	A7EEJ4	665079
C1GAS0	502780	C1HAH2	502779	Q4P2T2	5270
C1GN02	502780	C3TS12	3827	C0P079	447093
C1H7U4	502779	C3ZTE9	7739	A4S765	436017
C1HCB4	502779	C4JJV3	336963	A1CZP1	331117
C4JH93	336963	C4QZU2	644223	C1EIP1	296587

C4JJ69	336963	C4XWS2	306902	Q00VC8	70448
C4R0F4	644223	C4YKZ8	5476	D4ARJ4	663331
C4XXW4	306902	C5DE81	559295	D4DLR3	663202
C5DG64	559295	C5DQU3	559307	C1N079	564608
C5DHS0	559295	C5FEZ0	554155	C7ZGA9	660122
C5E197	559307	C5GE72	559297	C7ZGU9	660122
C5FG62	554155	C5JJ61	559298	C9SCV3	526221
C5FSN0	554155	C5M4D8	294747	Q0CE96	341663
C5GQ38	559297	C5P8V3	222929	Q0UQZ1	13684
C5GRM3	559297	C6H889	544712	B8BHC6	39946
C5JRR5	559298	C7GRS4	574961	C1EFZ8	296587
C5JX53	559298	C7Z0Z4	660122	C4YI55	5476
C5KH60	423536	C8Z7A8	643680		
C5M5H9	294747	C9SLY5	526221		
C5P1R8	222929	C9ZV24	679716		
C5P3Y5	222929	D3DLZ8	559292		
C5Z5C5	4558	D4AZF7	663331		
C6H9G8	544712	D4D8Y9	663202		
C6HHA1	544712	D5A7T4	3332		
C6TAH0	3847	D5GAV4	39416		
C6TG29	3847	D7L198	81972		
C7GX10	574961	D7LXP0	81972		
C7GYF8	574961	D7M065	81972		
C7YHE2	660122	O50008	3702		
C7ZPP7	660122	P05694	4932		
C8VB52	227321	P82610	5476		
C8VRR3	227321	P93263	3544		
C8Z7Z2	643680	Q069K2	200320		
C8Z868	643680	Q0CLG2	341663		
C9SJ68	526221	Q0U9I2	13684		
D0NLW7	403677	Q0UTW0	13684		
D1ZFS7	5147	Q0WNZ5	3702		
D3BFM3	670386	Q27JJ6	4932		
D4AVM8	663331	Q27JK0	4932		
D4B502	663331	Q2GPU2	38033		
D4D1Q8	663202	Q2H923	38033		
D4DDM4	663202	Q2QLY4	39947		
D5GE29	39416	Q2QLY5	39947		
D6VTT7	559292	Q2UGH7	5062		
D6VTX0	559292	Q42662	4142		
D7FL39	2880	Q42699	4058		
D7LVN0	81972	Q4H1G2	161934		
O94350	4896	Q4LB12	4513		
P43623	4932	Q4LB13	4513		
P53101	4932	Q4P6X9	5270		
P53780	3702	Q4PG29	5270		

Q011L0	70448	Q4Q6R3	5664
Q0C8C6	341663	Q4WNY2	5085
Q0CVN8	341663	Q54X49	44689
Q0CX10	341663	Q57XC8	5691
Q0U3T3	13684	Q5B4T7	162425
Q12607	162425	Q5EMU4	148305
Q2H105	38033	Q5K9D7	5207
Q2U0S6	5062	Q6BCT3	4513
Q2U2B2	5062	Q6BYS1	4959
Q2UBS4	5062	Q6C638	4952
Q3EAI6	3702	Q6CMI0	28985
Q4P317	5270	Q6FQM7	5478
Q4Q511	5664	Q6GYJ7	4922
Q4QFT8	5664	Q6KCR0	3702
Q4WAK1	5085	Q6KCR2	3702
Q4WVZ1	5085	Q71EW8	3847
Q55DV9	44689	Q75D10	33169
Q5ACX5	5476	Q7S0I6	5141
Q5AXC9	162425	Q8H162	3702
Q5BFJ9	162425	Q8W0Q7	4558
Q5K9J3	5207	Q8X1E4	5141
Q5K9J4	5207	Q93ZK3	3702
Q6BYU9	4959	Q94BN4	3702
Q6BZI1	4959	Q9LM03	4113
Q6BZJ6	4959	Q9P444	162425
Q6CAR4	4952	Q9P8N9	5499
Q6CSX8	28985	Q9SRV5	3702
Q6FL54	5478	Q9UT19	4896
Q756K4	33169		
Q75CH8	33169		
Q96VU8	5141		
Q9LWJ2	39947		
Q9LWJ5	39947		
Q9MT31	4113		
Q9UV02	40559		

**cystathionine gamma-lyase**		**homocysteine S-methyltransferase**		**betaine-homocysteine methyltransferase**	
**UniProt**	**TXID**	**UniProt**	**TXID**	**UniProt**	**TXID**
P31373	4932	Q8LAX0	3702	Q93088	9606
D6VPK6	559292	D7L2F0	81972	Q5RFG2	9601
C8Z3L8	643680	B9RTM3	3988	O35490	10090
C7GPM2	574961	B9HUS6	3694	A2ALP9	10090
B5VDL1	545124	B9HK01	3694	Q5I597	9913
B3LUU9	285006	C6TII2	3847	O09171	10116
A7A0F7	307796	C6TCM1	3847	Q5M8Z0	8364
Q6FRS2	5478	Q0TXM4	13684	Q5XGM3	8355

A7TP41	436907	D3TPQ2	37546	Q32LQ4	7955
Q6CKV1	28985	D3TRN2	37546	B5DGE7	8030
Q758U3	33169	B4MZM7	7260	A7XLV2	8167
C5DVD2	559307	B4MZM6	7260	A2TF10	8255
C5DG64	559295	Q29LT6	46245	Q1KMD2	8175
C5MAJ9	294747	Q29LT7	46245	A7RLS1	45351
B9WMM0	573826	B4G7F6	7234	C3KIA2	229290
A5E0C2	36914	B4G7F5	7234	B3RR62	10228
Q6BTV4	4959	B4Q902	7240	B3RR63	10228
Q5A362	5476	B4Q903	7240	C3Y1N2	7739
A3LSH7	4924	B4JBY2	7222	B7P2U0	6945
C4Y2M1	306902	B4JBY1	7222	A7RIN5	45351
Q6C2Q9	4952	A2R696	425011	B2RCQ6	9606
B2WCB8	426418	A3LQC9	4924	B2RDF4	9606
D5GM51	39416	A4R5G4	148305	B4DPF0	9606
Q2TYY9	5062	A8KB71	7955	B7Z516	9606
B8NYK4	332952	A8WG15	7955	C3YKD9	7739
A4RNB9	148305	B3S7K9	10228	C3YKZ2	7739
C5GU28	559297	B6KB25	508771	C3YN42	7739
A2R8X8	425011	B9H8B8	3694	C3ZZV0	7739
C1H272	502779	B9HWH8	3694	Q3UEP0	10090
C5JP51	559298	C3ZEE1	7739	Q5RF32	9601
C1G5K1	502780	C8VHP4	227321	Q68FT5	10116
C0NFW6	447093	D6WLX1	7070	Q91WS4	10090
Q0D1Y8	341663	Q10PU4	39947	Q9H2M3	9606
A1C3X3	5057	Q22HI1	312017		
C8VM61	227321	Q2TXK9	5062		
B2LXT6	162425	Q4CP82	5693		
Q5BDD4	162425	Q4DI99	5693		
B6HHI9	500485	Q4GZ92	5691		
A1DB44	331117	Q4PDM6	5270		
B8MDX9	441959	Q4Q0C9	5664		
A6S4T4	332648	Q4WE23	5085		
C5P2T0	222929	Q5KA93	5207		
Q4WCI1	5085	Q5ZBZ6	39947		
B0YA29	451804	Q6C0D6	4952		
A7E8S4	665079	Q6PBE1	8364		
B2AYE2	5145	Q753B4	33169		
B6QM86	441960	Q7SFT2	5141		
C4JIG7	336963	Q7XEH2	39947		
C7YKF3	660122	Q9M1W4	3702		
C5FX28	554155	Q9SDL7	3702		
C4QYL9	644223	A1C5J4	5057		
D1ZJF8	5147	A1D0G5	331117		
Q96VU7	5141	A2Z7F0	39946		
Q0TZA5	13684	A2ZMI7	39946		

Q2HFS9	38033	A4HQE8	5660
Q9P417	5044	A4IE37	5671
C0RXA9	482561	A4ZGQ8	36774
C6HL43	544712	A9RGI7	145481
D4AQG6	663331	A9RI39	145481
C3YTG3	7739	A9SFD6	145481
D1FNY6	168717	B0WAH1	7176
D2V6M2	5762	B0WQ03	7176
Q55DV9	44689	B0Y1P6	451804
B5X1V6	8030	B2AAZ0	5145
Q4PG01	5270	B3MPE3	7217
Q6P849	8364	B3NL78	7220
A9Z022	8022	B4FK22	4577
D3BFM3	670386	B4I5M7	7238
A6QZA3	339724	B4KJP9	7230
A7S143	45351	B4LSJ7	7244
Q640H4	8355	B4PAD4	7245
Q6NWE3	7955	B5X0U2	8030
Q6PHI6	7955	B6HVM8	500485
Q68EI1	7955	B6TK96	4577
C3KJ67	229290	B6TYG7	4577
Q4SDU9	99883	B8A042	4577
B0DR66	486041	B8AA61	39946
B0DR59	486041	B8AQV5	39946
B0DFR8	486041	B8NVJ1	332952
Q60HG7	9541	B9GSK5	3694
Q19QT7	9823	B9PL90	5811
B8P570	561896	B9QBQ1	432359
Q9EQS4	10116	B9SFH3	3988
D3ZQX6	10116	B9T060	3988
P18757	10116	C0PRQ1	3332
Q17DR0	7159	C5P4X0	222929
Q17DR1	7159	C5WSP6	4558
D3Z570	10090	C5XLK6	4558
Q8VCN5	10090	C5YRH9	4558
B3S3W7	10228	C7YSG1	660122
B3S3V3	10228	C8CGW4	668559
A0BJI2	5888	C8CGW5	668558
A0E9Q1	5888	C8CGW6	90165
A0D3Z7	5888	C8CGW7	668556
Q7QHF8	7165	C8CGW8	668557
D0NAM5	403677	C9ZI75	679716
Q7JXZ2	7227	D1ZZE7	7070
O97121	7227	D7LQW5	81972
B7GAW0	556484	D7LTP3	81972
B3NK88	7220	P56707	20406

P32929	9606	Q0CYN5	341663
B4E1R2	9606	Q0WTD0	3702
B0W7Q2	7176	Q17C46	7159
B0WHB0	7176	Q1HL00	4442
B4P4B7	7245	Q2F5Q8	7091
B0JYP8	9913	Q2QME6	39947
Q58DW2	9913	Q2QME7	39947
Q2F6B7	7091	Q2QME8	39947
D6X383	7070	Q3EAF5	3702
B3MDE6	7217	Q3EB03	3702
B4HNZ0	7238	Q4VNK0	36774
B4QD03	7240	Q5B7V2	162425
B4MG50	7244	Q8H825	39947
B4M0Z9	7244	Q8MQN1	7227
B4JPP3	7222	Q9FUM7	4577
Q5RFI1	9601	Q9FUM8	4577
D3TMY0	37546	Q9FUM9	4577
A8WLD9	6238	Q9FUN0	4577
Q23CV0	312017	Q9VJ32	7227
B4MP07	7260		
B4N4K0	7260		
P55216	6239		
O45391	6239		
C4WW23	7029		
C4WW93	7029		
C1C2T8	344056		
A9VCT5	81824		
C9SMM7	526221		
A2QA31	425011		
A2QY34	425011		
A3LTQ0	4924		
A3LX62	4924		
A4QRH4	148305		
A4S2B4	436017		
A4S3Y0	436017		
A5DVE9	36914		
A7F083	665079		
A8J355	3055		
A9S5H9	145481		
B2VLA4	515849		
B6HEW5	500485		
B7GC92	556484		
B9IK07	3694		
B9W7F1	573826		
C4MAS9	5759		
C4R1M9	644223		

D6VWU8	559292
D6VXV0	559292
D6W0K0	559292
O42851	4896
O74314	4896
P38675	5141
P55217	3702
Q2U598	5062
Q2UJR0	5062
Q4FWE7	347515
Q4P760	5270
Q4WWD8	5085
Q59VR0	5476
Q5A410	5476
Q5B7M4	162425
Q5KL05	5207
Q6C8S4	4952
Q6CB42	4952
Q6CMC4	28985
Q6CWG2	28985
Q6FK75	5478
Q6FR34	5478
Q756W2	33169
Q86D27	5759
A1CBW5	5057
A1CG82	5057
A1CJ22	5057
A1D8F6	331117
A1D936	331117
A1DDE0	331117
A2QIU2	425011
A2QYG1	425011
A4HMZ0	5660
A4IBL4	5671
A4RMG5	148305
A5DN62	4929
A5E6Z6	36914
A6R1N2	339724
A6R497	339724
A6RIC4	332648
A6SDV0	332648
A6ZLY2	307796
A6ZQ85	307796
A7A0K2	307796
A7EFY4	665079
A7TG47	436907

A7WPD7	51657
A9PID6	3694
A9T1Q4	145481
B0DAR0	486041
B0EJN7	370354
B0ELR1	370354
B0EN45	370354
B0XZ86	451804
B0Y277	451804
B0YBX6	451804
B1N417	5759
B2AUC6	5145
B2G4B3	4956
B3LLH3	285006
B3LQL2	285006
B3LTC0	285006
B4F935	4577
B4FJ26	4577
B4FNW6	4577
B5VMP7	545124
B5VP46	545124
B6HAD5	500485
B6HMV3	500485
B6K3B8	402676
B6K8Y1	508771
B6QTJ7	441960
B6QUU5	441960
B8AQ99	39946
B8CCE6	35128
B8LL39	3332
B8MNR4	441959
B8N392	332952
B8N7B3	332952
B8NK20	332952
B8NV95	332952
B9F8N9	39947
B9PIG1	5811
B9Q9E9	432359
B9WMS1	573826
C0NB75	447093
C0NZA4	447093
C0S0K0	482561
C0SD44	482561
C0Z354	3702
C1EHF0	296587
C1GAS0	502780

C1GF82	502780
C1H7U4	502779
C1HDT5	502779
C1MNK0	564608
C4JJ69	336963
C4JWP3	336963
C4R3L3	644223
C4Y8G1	306902
C4YBC9	306902
C4YEQ7	5476
C5DFU1	559295
C5DJ03	559295
C5DLF9	559295
C5DRG8	559307
C5DY28	559307
C5DYT7	559307
C5FSN0	554155
C5G096	554155
C5GQ38	559297
C5GR68	559297
C5JR79	559298
C5JRR5	559298
C5KHB3	423536
C5KL47	423536
C5LFG5	423536
C5ME56	294747
C5MIQ7	294747
C5P1R8	222929
C5PEF2	222929
C5WYM5	4558
C6H9G8	544712
C6HP80	544712
C7GM83	574961
C7GND7	574961
C7GW18	574961
C7YHE2	660122
C7YP17	660122
C8VRR3	227321
C8ZBS8	643680
C8ZCR8	643680
C8ZEC2	643680
C9SXQ4	526221
C9ZZ26	679716
D4AVM8	663331
D4B279	663331
D4DDM4	663202

D4DEE8	663202
D5GJJ5	39416
D7LAJ0	81972
O04981	4577
O04982	4577
O23944	57918
O81334	3544
P47164	4932
Q012C1	70448
Q04533	4932
Q0C8C6	341663
Q0CX10	341663
Q0GC25	3879
Q0TXK2	13684
Q10KP2	39947
Q10KP3	39947
Q10KP4	39947
Q12198	4932
Q2H5Y9	38033
Q2HH43	38033
Q2U2B2	5062
Q2UBS4	5062
Q38DH3	5691
Q4DUG8	5693
Q4WAK1	5085
Q4WVZ1	5085
Q55V77	5207
Q59VV6	5476
Q5ARD6	162425
Q5BFJ9	162425
Q6BSE9	4959
Q6BZI1	4959
Q6BZJ6	4959
Q6R8F6	4081
Q756K4	33169
Q86D28	5759
Q86ZL4	5145
Q8S7G4	4530
Q9C816	3702
Q9C876	3702
Q9MT29	4113
Q9MT30	4113
Q9SPT6	4113
Q9XFG0	3847
Q9ZPL5	4097
A5JYW2	6239

C4QJR9	6183
Q5K6Y9	5207
A2FEV4	5722
B1X4E2	39717
B4HBA6	7234
B4KJJ5	7230
B5DS13	46245
C1BU59	72036
O02215	6239
O15564	5722
O15565	5722

5-methyltetrahydrofolate--homocysteine methyltransferase		serine O-acetyltransferase		cysteine synthase A	
**UniProt**	**TXID**	**UniProt**	**TXID**	**UniProt**	**TXID**
Q99707	9606	A2WUJ7	39946	A0E1N6	5888
Q4JIJ3	9913	A2X259	39946	A1C462	5057
Q9Z2Q4	10116	A2XC56	39946	A1C4R2	5057
A6H5Y3	10090	A2XDJ1	39946	A1C5C7	5057
Q5ZIC7	9031	A2Y6U9	39946	A1CZP1	331117
Q8JIY6	7955	A4IA64	5671	A1D097	331117
C3YH00	7739	A4S0T9	436017	A1D9N4	331117
A7SKT1	45351	A5AZH7	29760	A1DBC7	331117
Q09582	6239	A5C9C1	29760	A2DFV0	5722
A8XY95	6238	A8JDD3	3055	A2E119	5722
A9V1Z5	81824	A9NXF4	3332	A2EI82	5722
D3BM55	670386	A9PJW7	3695	A2EQY3	5722
Q54P92	44689	A9TE87	145481	A2EUZ5	5722
D2UYB1	5762	A9TFY0	145481	A2GMG5	5722
C1EAX2	296587	A9U0K5	145481	A2QUT4	425011
A4HT60	5671	B0E8A7	370354	A2R8L0	425011
A8HYR2	3055	B0EDF4	370354	A2Y9E5	39946
C1MHB3	564608	B0EMQ1	370354	A2ZMY2	39946
Q4QIT5	5664	B0EPH7	370354	A3AQX8	39947
A4H4V2	5660	B0ETS9	370354	A3BCT7	39947
A4RUZ2	436017	B4FLW0	4577	A3CJM0	39947
C5K5V1	423536	B4FRV1	4577	A3LRL7	4924
C5KDF9	423536	B4FU68	4577	A3LST3	4924
B7GBG7	556484	B6TDM6	4577	A3LWK4	4924
D7FUT0	2880	B6TFX8	4577	A3RM03	3847
B8C9X1	35128	B7FKI8	3880	A3RM04	3847
B7ZLW8	9606	B8LM71	3332	A3RM05	3847
B9TKY6	3988	B8LMI3	3332	A3RM06	3847
Q5R872	9601	B9F3W8	39947	A4HPM6	5660
		B9FHA5	39947	A4ID39	5671

B9G4N2	39947	A4QRE5	148305
B9GFK2	3694	A4R8I4	148305
B9H655	3694	A4RHY9	148305
B9I1J9	3694	A4RXR0	436017
B9IDB2	3694	A4RZI1	436017
B9MWB2	3694	A4S621	436017
B9P7Y5	3694	A4S765	436017
B9RKA6	3988	A5AAI2	425011
B9S6T2	3988	A5AEP0	29760
C1EAI1	296587	A5AFH5	29760
C1MHL4	564608	A5AVW3	29760
C5WUP1	4558	A5AXK6	29760
C5WZ15	4558	A5B2H2	29760
C5XID2	4558	A5BDL2	29760
C5YUG1	4558	A5C056	29760
D7FYH8	2880	A5C903	29760
D7KN39	81972	A5DDM8	4929
D7L0N8	81972	A5DIR7	4929
D7L9G7	81972	A5DLM4	4929
D7MCE5	81972	A5E3W7	36914
D7MM67	81972	A5E599	36914
P93544	3562	A5E7I6	36914
Q014F1	70448	A5HKN4	3848
Q0DGG8	39947	A5YT86	3847
Q0DUI1	39947	A5YT88	3847
Q10QH1	39947	A6N135	39946
Q10S58	39947	A6QSN0	339724
Q1EP44	52838	A6QZJ2	339724
Q39218	3702	A6R9L3	339724
Q39533	3654	A6RRM2	332648
Q401L4	5759	A6S2U3	332648
Q401L5	5759	A6SJC6	332648
Q42538	3702	A6ZV03	307796
Q42588	3702	A7A1I7	307796
Q4D6P5	5693	A7EEJ4	665079
Q4Q2Q9	5664	A7ELJ2	665079
Q6IWF8	60913	A7EQU1	665079
Q6IWF9	60913	A7MBF8	9913
Q6IWG0	60913	A7TKV2	436907
Q6ZHU3	39947	A7TLB1	436907
Q84VU9	161934	A7WQ39	2966
Q8GZP7	4577	A7YXT6	342587
Q8GZP8	4577	A8IEE5	3055
Q8GZP9	4577	A8ISA9	3055
Q8LPN9	3055	A8ISB0	3055
Q8S895	3702	A8J434	3055

Q8W0E4	39947
Q8W199	3847
Q8W2B8	3702
Q9BKB1	5693
Q9MAZ3	4683
Q9SDP2	4679
Q9SLZ8	45157
Q9U8X1	46681
Q9U8X2	5759
Q9US33	4896

A8JFA4	3055
A8N6U5	240176
A8P274	240176
A8PT64	425265
A8PY60	425265
A8Q079	425265
A8WP61	6238
A8XB74	6238
A9JT04	7955
A9JX40	13684
A9NR69	3332
A9NRJ4	3332
A9NS10	3332
A9PA30	3694
A9PGL6	3694
A9PJI4	3695
A9RMD3	145481
A9SEU2	145481
A9SQU9	145481
A9T924	145481
A9TXJ4	145481
A9V9E5	81824
A9VB30	81824
A9VDJ3	81824
A9Y098	4182
B0D0C2	486041
B0D119	486041
B0DT91	486041
B0E5R8	370354
B0E6A6	370354
B0E9N8	370354
B0FTX3	3983
B0Y229	451804
B0Y332	451804
B0Y9U7	451804
B0YEG4	451804
B2AXA0	5145
B2AYH9	5145
B2B0Q4	5145
B2GVN3	3702
B2W2J7	426418
B2WDB4	426418
B2WLZ1	426418
B2Z452	328376
B3LIK5	285006
B3RHI1	285006

B3RT96	10228
B4FF56	4577
B4FKU8	4577
B4FR08	4577
B5RT41	4959
B5U9U9	3562
B5U9V0	3562
B5VIX1	545124
B5VNK3	545124
B6GZ70	500485
B6H387	500485
B6HEU9	500485
B6HFY6	500485
B6HJ05	500485
B6HWB3	500485
B6JW15	402676
B6JZ56	402676
B6K2Z3	402676
B6KRX1	508771
B6QF88	441960
B6QHT5	441960
B6QLW8	441960
B6TBZ1	4577
B6U9G4	4577
B6V3I4	2788
B7F3B0	39947
B7FKU7	3880
B7G7B7	556484
B7S487	556484
B7Z2D6	9606
B7Z6W5	9606
B7ZV69	7955
B8A367	4577
B8A377	4577
B8AA84	39946
B8ABA1	39946
B8AEL2	39946
B8AJV7	39946
B8B2R6	39946
B8BHC6	39946
B8BQD5	296543
B8BUL0	35128
B8LQB8	3332
B8MBE4	441959
B8MF97	441959
B8MIN1	441959

B8MXT9	332952
B8NCT4	332952
B8NR08	332952
B8NRD7	332952
B8P1T0	561896
B9DHF3	3702
B9F4C4	39947
B9F673	39947
B9FBT5	39947
B9GFL5	3694
B9GQA5	3694
B9GQA6	3694
B9HJY5	3694
B9I8A4	3694
B9MZH9	3694
B9MZI1	3694
B9MZI2	3694
B9MZI5	3694
B9NJL3	3694
B9P570	3694
B9RDU1	3988
B9RET4	3988
B9RET5	3988
B9RET6	3988
B9RET7	3988
B9RET8	3988
B9RET9	3988
B9RTR4	3988
B9RZ17	3988
B9S9T9	3988
B9SFU8	3988
B9SY80	3988
B9WF95	573826
B9WIY6	573826
B9WJY1	573826
C0NG61	447093
C0NT80	447093
C0P079	447093
C0P823	4577
C0PCX2	4577
C0RXJ3	482561
C0RY11	482561
C0SDA9	482561
C0SI30	482561
C0Z3K2	3702
C1DZ19	296587

C1DZY8	296587
C1ED55	296587
C1EFZ8	296587
C1EIP1	296587
C1G5U2	502780
C1G6C1	502780
C1GFF5	502780
C1GM15	502780
C1H1L8	502779
C1H5G7	502779
C1HA84	502779
C1HBF9	502779
C1MIX4	564608
C1N079	564608
C1N0F7	564608
C1N5N0	564608
C4JIL4	336963
C4JMC2	336963
C4JMM4	336963
C4M0H9	5759
C4MAH0	5759
C4QVR5	644223
C4R122	644223
C4R7J7	644223
C4XWE8	306902
C4Y3V6	306902
C4YBX3	306902
C4YI55	5476
C4YSM3	5476
C5DGA4	559295
C5DI11	559295
C5E0A9	559307
C5E1G7	559307
C5E1M5	559307
C5E370	559295
C5FIF7	554155
C5FXI1	554155
C5FZ17	554155
C5GIL6	559297
C5GP88	559297
C5GU14	559297
C5JFV5	559298
C5JP42	559298
C5JTC9	559298
C5JVI7	559298
C5LC28	423536

C5M1S7	294747
C5M8Q0	294747
C5MFY7	294747
C5P096	222929
C5P4F3	222929
C5P7U2	222929
C5WSS1	4558
C5XFP1	4558
C5XN68	4558
C5YC80	4558
C6HFC9	544712
C6HLE4	544712
C6T9Z8	3847
C6TDJ4	3847
C6TH76	3847
C6TIP9	3847
C6TJE5	3847
C6TMX6	3847
C7F7S7	42251
C7GK81	574961
C7GVT7	574961
C7YI90	660122
C7YR17	660122
C7YWL1	660122
C7Z8Q0	660122
C7ZGA9	660122
C7ZGU9	660122
C7ZJ43	660122
C8V3P9	227321
C8V672	227321
C8VMP5	227321
C8Z8Q6	643680
C8ZDR6	643680
C9SCB2	526221
C9SCV3	526221
C9SGZ8	526221
D0N3C1	403677
D0N4G9	403677
D0NL09	403677
D0NL10	403677
D0V0B2	93385
D0V0B3	93385
D1ZA82	5147
D1ZN84	5147
D2VTF3	5762
D3BV85	670386

D3DSK4	9606
D4AQQ5	663331
D4ARJ4	663331
D4AX74	663331
D4D0U1	663202
D4DBB4	663202
D4DLR3	663202
D5G3W6	39416
D5GJA2	39416
D5GKD1	39416
D6QX85	4565
D6QX86	4513
D6VUE8	559292
D6VYU6	559292
D7G6V6	2880
D7KN34	81972
D7L179	81972
D7L1Q3	81972
D7L305	81972
D7L832	81972
D7LKI2	81972
D7LS98	81972
D7LWG7	81972
D7M6S7	81972
D7M6S9	81972
D7MBA7	81972
D7MN67	81972
O01592	6239
O13326	4896
O15570	5759
O15635	5759
O16282	6239
O16284	6239
O22682	3702
O23733	3707
O23735	3707
O45679	6239
O59701	4896
O81154	4113
O81155	4113
O81523	3055
P06106	4932
P31300	4072
P32232	10116
P32260	3562
P35520	9606

P38076	4565
P47998	3702
P47999	3702
P50125	162425
P50867	162425
P53206	4932
P80608	4577
P87131	4896
Q00834	3562
Q00VC8	70448
Q00XK0	70448
Q016B2	70448
Q018U5	70448
Q0CAW1	341663
Q0CE96	341663
Q0D1N1	341663
Q0TYJ1	13684
Q0UQZ1	13684
Q0V2F1	13684
Q0WLF5	3702
Q0WW95	3702
Q0WWQ5	3702
Q10CX4	39947
Q10CX6	39947
Q10PZ5	39947
Q1K4Z1	5141
Q1KLZ1	3750
Q1KLZ2	3750
Q2GNW9	38033
Q2H6K3	38033
Q2HFV4	38033
Q2I306	231512
Q2QLX5	39947
Q2U0R3	5062
Q2U9X2	5062
Q2UAR3	5062
Q2URZ7	5062
Q3E8Z3	3702
Q3EAH2	3702
Q3EAH3	3702
Q3ECN8	3702
Q3L195	4682
Q3L196	4682
Q3L197	4682
Q3LAG5	4097
Q401L7	5759

Q43153	3562
Q43317	3654
Q43725	3702
Q43726	3702
Q4CS97	5693
Q4CST7	5693
Q4P0L8	5270
Q4P2T2	5270
Q4PCE5	5270
Q4Q159	5664
Q4W9S1	5085
Q4WC99	5085
Q4WE91	5085
Q4WEU7	5085
Q54CN7	44689
Q55IS7	5207
Q570E6	3702
Q58EC2	7955
Q58H57	9541
Q59US5	5476
Q5A0E3	5476
Q5ABX6	5476
Q5AC97	5476
Q5BD67	162425
Q5EI65	7955
Q5JNB0	39947
Q5K8F5	5207
Q5KCX2	5207
Q5N761	39947
Q5UJF9	39946
Q5VLJ3	3981
Q5VLJ4	3981
Q5VND2	39947
Q5VND3	39947
Q640V0	8364
Q6BL57	4959
Q6BME7	4959
Q6C1R7	4952
Q6C6L3	4952
Q6C7U9	4952
Q6CLM7	28985
Q6CST5	28985
Q6DDN9	8355
Q6FTU3	5478
Q6FVR1	5478
Q6NKY5	3702

Q750D4	33169
Q758B3	33169
Q75AE8	33169
Q767A2	3562
Q76KF8	5076
Q76MX2	4113
Q7RYW6	5141
Q7SHQ1	5141
Q7XBB5	2787
Q7XS58	39947
Q7Y256	3505
Q7ZXW6	8355
Q86NC5	6239
Q86NC6	6239
Q8LAQ6	3702
Q8LEC3	3702
Q8W1A0	3847
Q91WT9	10090
Q92441	28985
Q93244	6239
Q9C123	4922
Q9C262	5141
Q9CAV8	3702
Q9FS25	4113
Q9FS26	4113
Q9FS27	4113
Q9FS29	4113
Q9FSF5	4097
Q9LG24	3702
Q9LJA0	3702
Q9MAZ2	4683
Q9N0V7	9986
Q9P3D4	5141
Q9S6Z7	3702
Q9S757	3702
Q9SSV8	45157
Q9SSV9	45157
Q9SXS7	3702
Q9U5E4	46681
Q9U5E5	46681
Q9UR58	5476
Q9UVM2	5476
Q9XEA6	39947
Q9XEA8	39947
Q9XEA9	4530

cystathionine beta-synthase		chorismate mutase		prephenate dehydratase	
**UniProt**	**TXID**	**UniProt**	**TXID**	**UniProt**	**TXID**
P32582	4932	P32178	4932	P32452	4932
D6VUT5	559292	D6W465	559292	D6W0M9	559292
C8Z949	643680	C8ZJ85	643680	A6ZSG4	307796
C7GQ63	574961	C7GY46	574961	C8ZGK9	643680
B5VJ98	545124	B3LLB4	285006	C7GWR3	574961
B3LI75	285006	A6ZWU8	307796	B3LPF0	285006
A6ZUG6	307796	A7TRW1	436907	C5DSU3	559307
Q6FVB8	5478	C5DUE5	559307	A7THC9	436907
A7TNI3	436907	Q6CVY3	28985	Q6FJS1	5478
C5DGC1	559295	Q6FLZ7	5478	Q6CP67	28985
Q6CKN5	28985	C5DIU4	559295	C5DJU5	559295
Q750E3	33169	Q75BG5	33169	Q75AV0	33169
C5DZT7	559307	A3LWZ3	4924	B9WDL3	573826
C4XVN8	306902	B9W9F0	573826	C4R7W3	644223
A5DW54	36914	Q59TS4	5476	Q6BKZ2	4959
A5DCN0	4929	C5MCK6	294747	B6QGT5	441960
B2AVE1	5145	C4R6Q3	644223	C4YQ16	5476
C4R2R4	644223	A5DU51	36914	Q5AEN4	5476
Q96V35	4922	B5RUN3	4959	A3LZW3	4924
Q59T95	5476	C4Y2Y8	306902	B8MBZ7	441959
C4YF81	5476	A5DB21	4929	A2R9Q0	425011
Q2H8M0	38033	Q9P4D8	4905	Q2UTK6	5062
C5MEV0	294747	Q6C5J7	4952	B8NSF5	332952
A3LRC4	4924	C7Z4Q5	660122	C4JMH0	336963
B9W6X5	573826	Q6L8Q0	241238	Q30KV5	162425
Q6BU02	4959	D5GKL1	39416	C8V9C8	227321
Q6C6J2	4952	D1ZMF9	5147	C5P7Z3	222929
D5GFI0	39416	A4RJV4	148305	Q6CEA0	4952
Q8NJZ9	148305	Q7S8R4	5141	A1C9Q8	5057
A4R9L9	148305	B6Q7S9	441960	C1GF42	502780
C0NMD6	447093	C9S7U9	526221	C0SD07	482561
C6HT32	544712	B8MRH6	441959	Q4WTQ1	5085
C7Z7Y2	660122	C5JG06	559298	B0Y378	451804
B2LXT5	162425	C5GIY2	559297	B2VUD6	426418
Q5B0W0	162425	B6HLL8	500485	D4AXE7	663331
A7EUA9	665079	D4B2M3	663331	D4DL43	663202
B6Q8U6	441960	A7ETB4	665079	A6S156	332648
B2WMD2	426418	A1DDL7	331117	B6HG19	500485
C5PHL8	222929	D4D3S7	663202	C4Y7N5	306902
A1CSJ1	5057	Q0CJD7	341663	C7YYW1	660122
C8V083	227321	B0Y1U1	451804	C5FIF2	554155
D1ZVF0	5147	Q4WVS3	5085	Q0CA21	341663
B8NBZ9	332952	A1CBP7	5057	C6HG79	544712
B0XW58	451804	C1H355	502779	C0NE70	447093

Q7S3M7	5141	C1GBQ7	502780	C5JYQ4	559298
Q4X256	5085	C0SAC6	482561	C5GLZ1	559297
A1DG77	331117	C6HF62	544712	C1H9D9	502779
B8M103	441959	C0NT20	447093	A6R093	339724
C5FCC8	554155	A6S4C4	332648	D5G7A6	39416
B6H2J3	500485	A6QSD4	339724	Q0UFM9	13684
C4JF01	336963	C5FK82	554155	C9SPD4	526221
Q0U679	13684	A2R3Z4	425011	A7E5N7	665079
Q0CHH3	341663	B2WGT6	426418	B6K3E0	402676
C1GDG5	502780	Q2U5Z9	5062	O14361	4896
A2QKG7	425011	B8NLW5	332952	A1CZJ7	331117
C5K3P5	559298	C5P6C8	222929	A5DXH5	36914
A6RCS6	339724	C4JQB9	336963	A8PZM1	425265
C1HDZ3	502779	Q0U2T2	13684	B0CQ85	486041
C5G712	559297	Q9Y7B2	162425	C5MAB3	294747
D4AU51	663331	C8V2P3	227321	Q55YQ5	5207
D4D1T3	663202	D0MV05	403677	Q5KN26	5207
D3BRT1	670386	A4S1M4	436017	Q7RYQ1	5141
C3XZE8	7739	A8J7L1	3055	Q5AF21	5476
Q8H710	4787	C1FGP1	296587	A4RQP2	436017
D0NQV7	403677	B9RNW0	3988	Q10N17	39947
D0NLJ6	403677	B9RCY0	3988	A2Q4I2	3880
B3S7J1	10228	B9T3T6	3988	A2XT43	39946
B3RP93	10228	A9S498	145481	A2YQ89	39946
B3S262	10228	O13739	4896	A2Z452	39946
P46794	44689	C6THT1	3847	A2Z456	39946
Q91WT9	10090	C6TEQ5	3847	A3ATK7	39947
D3ZPH9	10116	C6TBF4	3847	A3C1L2	39947
P32232	10116	B6JVV2	402676	A5BWG3	29760
D3ZSC5	10116	Q5K7Y8	5207	A8CF65	39947
A7MBF8	9913	Q96VZ8	5207	A8HXC5	3055
Q58H57	9541	B5AAU1	112509	A9NXE9	3332
C0SAH1	482561	B2B2X4	5145	A9PHG2	3694
P35520	9606	Q4P6P3	5270	A9RME6	145481
D3DSK4	9606	A5AJI5	29760	A9RP56	145481
B7Z2D6	9606	B9VU83	29760	B1X4W3	39717
A9Z023	8022	B9IK27	3694	B4FGT4	4577
Q9N0V7	9986	B9IK28	3694	B4FQG2	4577
Q2TZA8	5062	B9HXM7	3694	B4FSJ7	4577
Q16YQ8	7159	B9IL55	3694	B5LAT0	4072
Q16YQ7	7159	P42738	3702	B6SYB7	4577
B7ZV69	7955	Q29Q24	3702	B7X943	3981
A9JT04	7955	B5LAU1	4072	B7X944	3981
Q5EI65	7955	B4FNK8	4577	B8A9D0	39946
Q58EC2	7955	D7LNU9	81972	B8ALJ8	39946
D3TMI8	37546	D7KX08	81972	B8LLZ1	3332

Q6DDN9	8355
Q7ZXW6	8355
Q640V0	8364
B4M0J8	7244
A7RYI9	45351
A7S1D2	45351
D2VBK6	5762
B4PYL9	7245
B0X072	7176
B0X071	7176
Q9VRD9	7227
B3NY62	7220
B4I6D4	7238
B4R3C5	7240
Q29FV1	46245
B7G0B1	556484
B3N0J1	7217
B4K570	7230
B4JYX7	7222
Q9YHU3	31033
Q2V0C9	7460
B4NCR6	7260
B4GWP0	7234
B9QBC8	432359
B6KBE8	508771
B9PKX0	5811
D6WU53	7070
Q9N4K2	6239
Q23264	6239
C5LTC2	423536
C5K8R7	423536
A0D2L1	5888
A0D3Z0	5888
A0DFI6	5888
Q9BKB2	5693
Q384R5	5691
D0A7T9	679716
A4HX92	5671
B6RB68	91233
Q4QEG9	5664
A8WRM3	6238
A8PS25	6279
C4PYD1	6183
C4PYD2	6183
Q5KNK5	5207
A8N8W2	240176
D7M3J4	81972
D2CSU4	4102
D2CSU5	4102
B7FR81	556484
B7S3U0	556484
Q2QN58	39947
B9GDY1	39947
Q5JN19	39947
C5XKW2	4558
C5XWV4	4558
C5YLL7	4558
D7FQ27	2880
A9V4X1	81824
A2WVD6	39946
A2X1M3	39946
A2YVM5	39946
Q9STB2	4081
A8NSD2	240176
A8PTC4	425265
Q30CZ6	28930
C5LJG6	423536
C5L754	423536
B0CVP9	486041
Q013A6	70448
Q15FD1	4170
Q9C544	3702
Q6H819	39947
Q9S7H4	3702
A3BTJ8	39947
B4G0R4	4577
B5VTL1	545124
B6TF54	4577
B6TU00	4577
B8A1Z5	4577
C0PPE1	4577
Q6Z9E6	39947
Q9XF60	3702
B8LQ85	3332
B9F7Q4	39947
B9FV22	39947
B9G110	39947
B9G554	39947
B9H107	3694
B9HM73	3694
B9HQT5	3694
B9HZ50	3694
B9RXK2	3988
B9SN95	3988
B9SUJ5	3988
C0PQ13	3332
C1FED1	296587
C5WNL7	4558
C5X5W2	4558
C5YFR9	4558
D3U715	4102
D3U716	4102
D3U717	4102
D7KI05	81972
D7L6M9	81972
D7LIZ1	81972
D7LML1	81972
D7M1H4	81972
O22241	3702
Q01L56	4530
Q0IZJ9	39947
Q0JDF7	39947
Q650V6	39947
Q650W1	39947
Q6JJ29	35884
Q6Z3Y3	39947
Q7XLY2	4530
Q9FNJ8	3702
Q9SGD6	3702
Q9SSE7	3702
Q9ZUY3	3702

Q9NG81	5689
B0CTT6	486041
A6SDF3	332648
B7QJ62	6945
Q4CRW9	5693
Q4CXR9	5693
Q4CYV4	5693
Q4DF37	5693
Q4DF38	5693
Q4E2W1	5693
Q4PAS1	5270
Q9BH24	5693
A0CDH1	5888
A7TZ44	72036
A9TXJ4	145481
B0D119	486041
B7Z6W5	9606
C5P7B8	222929
C5PCI8	222929
C7ZEF7	660122
O59701	4896
O94073	4932
Q4DF36	5693
Q55IS7	5207
Q5KCX2	5207
Q9SSV8	45157

**aspartate aminotransferase**		**histidinol-phosphate aminotransferase**		**aromatic amino acid aminotransferase I**	
**UniProt**	**TXID**	**UniProt**	**TXID**	**UniProt**	**TXID**
A0C550	5888	A1CP53	5057	P53090	4932
A0CRJ1	5888	A1D207	331117	D6VTV2	559292
A0CXN8	5888	A2QAL3	425011	C8Z853	643680
A0CYK9	5888	A3LSV4	4924	C7GWZ3	574961
A0E429	5888	A4R5D4	148305	B3LHQ0	285006
A0E7H1	5888	A4RSZ6	436017	A6ZTY7	307796
A1C625	5057	A5DDL3	4929	A7TT91	436907
A1CHY4	5057	A5E3V6	36914	Q6FTM8	5478
A1CMR9	5057	A6R8T8	339724	C5DT73	559307
A1CRM0	5057	A6RQF2	332648	C5DLN0	559295
A1CWS6	331117	A6ZVG1	307796	Q6CKK3	28985
A1D4K6	331117	A7E9J3	665079	Q752M6	33169
A1DGQ3	331117	A7THE9	436907	Q6BYU5	4959
A1DL69	331117	A8J3D3	3055	A3LMX7	4924
A1YQJ2	39947	A8MRI5	3702	C4XXW0	306902
A1ZAA5	7227	A8P5U9	240176	B9WA90	573826
A2DLF1	5722	A8PCL5	240176	C4YJ02	5476

A2F7X5	5722	A8Q8A0	425265	Q5ADA2	5476
A2G7J5	5722	A9NX10	3332	Q5ACW9	5476
A2QJA6	425011	A9T6Y6	145481	A5DV12	36914
A2R099	425011	A9VDN0	81824	C5M5E9	294747
A2R823	425011	B0DTT8	486041	A5DFM7	4929
A2WVA6	39946	B0XMN9	451804	C4QYZ3	644223
A3GGD7	4924	B1X4B7	39717	Q6C551	4952
A3GGR0	4924	B2AS75	5145	Q5AZE2	162425
A3LZ01	4924	B2VYI8	426418	Q5B339	162425
A4HD93	5660	B3LTW3	285006	C8V102	227321
A4HMB3	5660	B4FAW7	4577	C8V843	227321
A4R592	148305	B5VKJ3	545124	Q2UFD9	5062
A4R8A4	148305	B6HAF7	500485	Q2U485	5062
A4RX79	436017	B6K373	402676	A2QD20	425011
A5A6K8	9598	B6QBB4	441960	A2R526	425011
A5AZ93	29760	B6TRS1	4577	B8NHI9	332952
A5BPA3	29760	B7G747	556484	B8NTN9	332952
A5DH26	4929	B8AHA9	39946	C1H4Q3	502779
A5DJN4	4929	B8BPZ8	296543	C0SF57	482561
A5DPU3	4929	B8M3C4	441959	C1GK60	502780
A5DTD8	36914	B8N6W4	332952	A1C7J1	5057
A5E0D3	36914	B8P2C7	561896	Q4X0F7	5085
A5E3K7	36914	B9DHD3	3702	B0XTG0	451804
A5KBJ3	5855	B9SB59	3988	A1DHS0	331117
A6R293	339724	B9WFD8	573826	B2WC58	426418
A6RAW4	339724	C0NL35	447093	B2WP52	426418
A6RGB2	339724	C0S401	482561	C5JQK8	559298
A6SNC6	332648	C1FED0	296587	C5GBH3	559297
A6SR37	332648	C1FYG5	502780	B6Q744	441960
A6ZZL1	307796	C1GUI5	502779	C0NBP4	447093
A7A0T3	307796	C1ML32	564608	B6GY09	500485
A7AQ14	5865	C3SA65	15368	Q0CY85	341663
A7EET7	665079	C4JYD9	336963	A6R4Q9	339724
A7F916	665079	C4R1F7	644223	A6S3P7	332648
A7RMR5	45351	C4XWD3	306902	B8MRR4	441959
A7SPW2	45351	C4YI23	5476	B8MRR5	441959
A7TKU3	436907	C5DMK2	559295	B8MI34	441959
A8B1V5	184922	C5DPA3	559307	A7EXH0	665079
A8HXW8	3055	C5FC63	554155	C5P1D9	222929
A8I263	3055	C5G7N6	559297	C7YK49	660122
A8J129	3055	C5JKH7	559298	C7ZH25	660122
A8K482	9606	C5M214	294747	C7ZNJ6	660122
A8N1I2	240176	C5PAB2	222929	C7ZF95	660122
A8NFP4	240176	C5Y092	4558	A4R0K4	148305
A8PVH6	425265	C6TN17	3847	C9SIQ8	526221
A8Q9P5	425265	C7Z4V1	660122	Q0UVF5	13684

A8WYZ3	6238	C8VRC2	227321	B6K3W8	402676
A8XNT6	6238	C8ZAC8	643680	D5GJ27	39416
A9NUJ0	3332	C9S832	526221	C5FSY7	554155
A9NVA2	3332	D0MY30	403677	D4CYX5	663202
A9PHH1	3694	D1ZCJ6	5147	D4AYN7	663331
A9SXD7	145481	D4AV67	663331	C4JJL9	336963
A9TG09	145481	D4D1H8	663202	Q7S0F9	5141
A9U4V4	145481	D5GI57	39416	O14192	4896
A9UVY5	81824	D6VVH1	559292	Q9Y7S6	4896
B0CQ48	486041	D7FPJ8	2880	O94570	4896
B0CS62	486041	D7KZ00	81972	C6H9Z4	544712
B0WHA4	7176	O82030	4097	Q2HDU3	38033
B0WKE5	7176	P07172	4932	Q5KEQ7	5207
B0XND0	451804	P36605	4896	Q55XW3	5207
B0XS08	451804	P56099	5479	Q5KM31	5207
B0Y6M8	451804	Q0D0C3	341663	B2AY10	5145
B0YDJ9	451804	Q0DY86	39947	D1ZQT4	5147
B2ACQ8	5145	Q0UQ19	13684	Q5M8X7	8364
B2ACV8	5145	Q27GM5	3702	Q28I58	8364
B2W091	426418	Q2H2S0	38033	D3B4I4	670386
B2W5S5	426418	Q2UCT8	5062	B0CY75	486041
B3L0V6	5851	Q4P634	5270	Q6PB16	8355
B3LQZ0	285006	Q4WS41	5085	Q6IR98	8355
B3LT00	285006	Q595W3	4922	Q54K00	44689
B3MGP3	7217	Q59SJ5	5476	Q86AG8	44689
B3MJE1	7217	Q5BFG3	162425	Q5E9N4	9913
B3N8Z8	7220	Q5KM46	5207	Q8N5Z0	9606
B3NPW9	7220	Q6BMG0	4959	Q4W5N8	9606
B3RIR4	10228	Q6C7F4	4952	Q9WVM8	10090
B3SB31	10228	Q6CLF0	28985	Q3UNS8	10090
B4DJA6	9606	Q6FX16	5478	B7STY2	80604
B4DY39	9606	Q6MY81	5085	Q64602	10116
B4F9G1	4577	Q6YV10	39947	A8NRI2	240176
B4FUH2	4577	Q75B26	33169	D6RP20	240176
B4G803	7234	Q7S9U6	5141	D6RPR8	240176
B4H584	7234	Q8S3S8	39947	Q2KEC9	242507
B4HSK3	7238	Q949X3	3702	Q4PDK9	5270
B4J5U2	7222	Q9FEW2	4092	Q4P8M5	5270
B4JZ99	7222			D6WQD2	7070
B4KKW3	7230			C1BQK8	217165
B4KMG1	7230			B8P756	561896
B4KZL9	7230				
B4LCJ4	7244				
B4LNV2	7244				
B4LVI1	7244				
B4MQX8	7260				

B4N192	7260
B4NW28	7245
B4P6Q8	7245
B4Q7L0	7240
B4QHF7	7240
B5E189	46245
B5VM51	545124
B5X142	8030
B5X205	8030
B5X2F0	8030
B5X3Z1	8030
B5YN39	35128
B6HSS5	500485
B6HVF7	500485
B6HW12	500485
B6HW52	500485
B6JWD9	402676
B6K3W4	402676
B6KH83	508771
B6Q2G9	441960
B6QDD0	441960
B6QPX5	441960
B6T9J4	4577
B6VBA1	135651
B7G9Y7	556484
B7GDE4	556484
B7Q1F6	6945
B7XA48	89462
B7Z1I2	9606
B7Z7E9	9606
B7ZXK1	4577
B8A0T6	4577
B8AEH4	39946
B8AEL7	39946
B8B3J3	39946
B8BQR8	296543
B8LKC5	3332
B8LLB8	3332
B8LWS2	441959
B8M6F1	441959
B8MC93	441959
B8MC94	441959
B8N0W2	332952
B8NBC7	332952
B8NKG3	332952
B9DGA8	3702

B9H919	3694
B9H920	3694
B9HAW0	3694
B9IA57	3694
B9IL28	3694
B9PQT7	5811
B9QD75	432359
B9RKN9	3988
B9RNY6	3988
B9RS46	3988
B9RS47	3988
B9SW33	3988
B9WBI3	573826
B9WFI3	573826
B9WMG0	573826
C0NCV0	447093
C0NMW6	447093
C0PSQ5	3332
C0PSV4	3332
C0S2A4	482561
C0S545	482561
C0S650	482561
C1BMI0	217165
C1BNE7	217165
C1BS16	72036
C1C1U3	344056
C1E2Z1	296587
C1G388	502780
C1G3V5	502780
C1G8V0	502780
C1GRJ9	502779
C1GVQ8	502779
C1H6W5	502779
C1L3B5	5127
C1N9D2	564608
C3YIV5	7739
C3ZZL9	7739
C4J030	4577
C4JEM6	336963
C4JFL7	336963
C4JRQ5	336963
C4QAH1	6183
C4QEQ2	6183
C4QWE4	644223
C4R862	644223
C4XWU9	306902

C4XZV4	306902
C4Y805	306902
C4YHW0	5476
C4YIK0	5476
C5DGC6	559295
C5DGI0	559295
C5DRL3	559307
C5DZC7	559307
C5FPT6	554155
C5FQ07	554155
C5FUQ2	554155
C5GEK9	559297
C5GFQ6	559297
C5GS95	559297
C5JII9	559298
C5JS27	559298
C5JWL7	559298
C5KR26	423536
C5KTB2	423536
C5KTB3	423536
C5LTG3	423536
C5LX48	423536
C5M0L9	423536
C5M0M0	423536
C5M290	294747
C5M6K3	294747
C5M8E6	294747
C5PAS0	222929
C5PGG6	222929
C5PHA1	222929
C6H2K1	544712
C6HI41	544712
C6HJN1	544712
C6LVT5	598745
C6THE3	3847
C6TNL0	3847
C7GVK5	574961
C7GXH3	574961
C7Z199	660122
C7Z7G7	660122
C8V2V4	227321
C8VA37	227321
C8VL70	227321
C8ZC70	643680
C8ZCZ9	643680
C9SFD1	526221

D0A277	679716
D0A706	679716
D0MVV7	403677
D2UYK0	5762
D2V5T4	5762
D3TRZ7	37546
D4AKW1	663331
D4ALE1	663331
D4B5K0	663331
D4DD02	663202
D4DGI1	663202
D4DKV6	663202
D5GE31	39416
D6VXI2	559292
D6VY29	559292
D6WJR3	7070
D6WLK0	7070
D7KU69	81972
D7LCW9	81972
D7LZ93	81972
D7M3R5	81972
D7MAT0	81972
O01804	6239
O20099	28957
O22618	47247
O42652	4896
O48548	3847
O48599	39947
O94320	4896
O96142	36329
P00503	9823
P00504	9031
P00505	9606
P00506	9823
P00507	10116
P00508	9031
P05201	10090
P05202	10090
P08906	9796
P12344	9913
P13221	10116
P17174	9606
P23542	4932
P28011	3879
P28734	4039
P33097	9913

P37833	39947
P46248	3702
P46643	3702
P46644	3702
P46645	3702
P46646	3702
P93763	28957
Q01802	4932
Q019P1	70448
Q0CGI9	341663
Q0CGM4	341663
Q0CPI2	341663
Q0CZI5	341663
Q0DBN4	39947
Q0GA62	5037
Q0JJ47	39947
Q0UL49	13684
Q0WSV8	3702
Q16LN3	7159
Q17983	6239
Q17994	6239
Q18L70	40286
Q18L71	40284
Q18L72	5664
Q1EBW2	3702
Q1HQC7	7091
Q22066	6239
Q22067	6239
Q23JV0	312017
Q28F67	8364
Q29LN4	46245
Q2PD61	5667
Q2PD62	5666
Q2PD75	5661
Q2PD87	5661
Q2PD89	5661
Q2PD92	5671
Q2TU84	9606
Q2TZX5	5062
Q2U6Q7	5062
Q2UJK8	5062
Q2V3D0	3702
Q2XTE6	4113
Q385Q9	5691
Q3ECJ9	3702
Q3UJH8	10090

Q40107	3871
Q40108	3871
Q40325	3879
Q41199	3847
Q42391	4540
Q42425	4540
Q42794	3847
Q42803	3847
Q43057	4540
Q43305	4540
Q43781	34305
Q4CRK6	5693
Q4D080	5693
Q4D1Q4	5693
Q4FX34	347515
Q4JHH9	6282
Q4KYM6	29159
Q4N691	5875
Q4P5Q1	5270
Q4PH18	5270
Q4QAU4	5664
Q4R559	9541
Q4R5L1	9541
Q4SXT0	99883
Q4UDB3	5874
Q4WCU8	5085
Q4WK05	5085
Q4WPT7	5085
Q4X1K5	5085
Q4YZE6	5821
Q54SF7	44689
Q55F21	44689
Q55II6	5207
Q56YR4	3702
Q59N40	5476
Q59ZG5	5476
Q59ZM9	5476
Q5AMH7	5476
Q5ASM1	162425
Q5B082	162425
Q5BBT7	162425
Q5DBC8	6182
Q5K6H3	7159
Q5K6H4	7159
Q5K7X5	5207
Q5K826	5207

Q5NVH2	9601
Q5R691	9601
Q5REB0	9601
Q5Z7I8	39947
Q6BQN8	4959
Q6BV55	4959
Q6BXH3	4959
Q6BZZ9	4952
Q6CFZ7	4952
Q6CJL3	28985
Q6CK45	28985
Q6EUS6	39947
Q6FMF0	5478
Q6FPE0	5478
Q6FW98	5478
Q6KAJ2	39947
Q6NX26	8364
Q753W1	33169
Q75DQ5	33169
Q7G1G6	3847
Q7K221	7227
Q7PM06	7165
Q7RR40	73239
Q7S9X7	5141
Q7SC43	5141
Q7SYK7	7955
Q7SZS3	8355
Q7YWB4	7227
Q7ZTK9	8355
Q7ZUW8	7955
Q7ZWF5	7955
Q86E27	6182
Q8HQQ0	3885
Q8IPY3	7227
Q8L7N7	3702
Q8T0M9	7227
Q95VP1	6279
Q964E9	5741
Q964F0	5691
Q964F1	5691
Q964F2	5656
Q9VQ61	7227

**tyrosine aminotransferase**		**D-3-phosphoglycerate dehydrogenase**		**phosphoserine aminotransferase**	
**UniProt**	**TXID**	**UniProt**	**TXID**	**UniProt**	**TXID**
Q9LVY1	3702	A0SXJ4	4932	P33330	4932

D7MI01	81972	A1C9P2	5057	D6W2P0	559292
B9MYE9	3694	A1CIB1	5057	C7GLR0	574961
B9MYF0	3694	A1CP23	5057	B5VS68	545124
B9MYF1	3694	A1CWE9	331117	B3LJL4	285006
B9HZQ9	3694	A1CZL2	331117	A6ZP29	307796
A9PF65	3694	A1D1X7	331117	C8ZGU3	643680
A5AY27	29760	A1DFM4	331117	C5DWG5	559307
A5BQ34	29760	A1L3N1	8355	A7THM8	436907
Q6K5U4	39947	A2DLU8	5722	Q6FSL3	5478
B9F563	39947	A2F266	5722	C5DN86	559295
Q5Z7R3	39947	A2F8V0	5722	Q6CUH4	28985
Q6K4T7	39947	A2Q988	425011	Q74ZB1	33169
B7FA99	39947	A2QU03	425011	B9WBR9	573826
A2X3T3	39946	A2R9R5	425011	Q59P52	5476
A2X3V3	39946	A2XYC2	39946	C4R4X8	644223
B8B1P8	39946	A2YFS1	39946	A3LW21	4924
A2ZFA2	39946	A2YVQ8	39946	Q6BMW0	4959
C5YQ31	4558	A3BE72	39947	C4Y8M3	306902
C5Y4D9	4558	A3LPQ2	4924	A5DWY8	36914
C5XA85	4558	A4H3H6	5660	Q6C2N1	4952
C5Y4D8	4558	A4HZ28	5671	A5DG71	4929
C5XPF1	4558	A4R1I1	148305	C7YUS7	660122
C6T727	3847	A4RI06	148305	A1CM37	5057
Q6DID9	8364	A4RX85	436017	A1DLY7	331117
A0JMN6	7955	A4S901	436017	Q2GU63	38033
Q16X98	7159	A5A6P1	9598	Q4WDJ5	5085
Q4SKB4	99883	A5C3X6	29760	B0YCV4	451804
B3NVU4	7220	A5DEL5	4929	Q10349	4896
Q01FZ1	70448	A5DSV7	36914	B2B5S0	5145
B0W9G3	7176	A5GFY8	9823	Q7RWH9	5141
D3B9D9	670386	A6QVW0	339724	Q871N3	5141
B4MTG1	7260	A6QY69	339724	A4R4Y8	148305
B4Q2S3	7245	A6R0F4	339724	D4D9Z2	663202
Q7QF69	7165	A6RHA3	339724	A2QBQ7	425011
Q9VY42	7227	A6RR69	332648	A7EUP1	665079
B4IGC3	7238	A6RX75	332648	D4AIQ9	663331
B4L2T7	7230	A6RZS6	332648	C5P2B0	222929
B4M2W5	7244	A6ZR37	307796	B8N7T9	332952
C1MLQ1	564608	A6ZVJ9	307796	C9S8M5	526221
B4H054	7234	A7E4H2	665079	Q2UID4	5062
Q29H80	46245	A7EN32	665079	A6R965	339724
B3RJH2	10228	A7EW07	665079	B6K167	402676
B3SCB8	10228	A7TRN8	436907	C1DZS4	296587
B4JJ14	7222	A7TRX8	436907	B6QEJ9	441960
Q4E4E4	5693	A8DWN5	45351	Q0CZ29	341663
Q4E4E9	5693	A8ITU2	3055	C4JP59	336963

Q9GUA4	5698	A8ITU6	3055	C5FRR6	554155
Q23DS3	312017	A8ITU7	3055	B8MCI4	441959
A0E687	5888	A8N1B6	240176	A9SH82	145481
Q6BG48	5888	A8PWQ2	425265	B3S7Q4	10228
A4HPA3	5660	A8XYH5	6238	D5GHM5	39416
A4IDL0	5671	A9NXI5	3332	A2XCQ4	39946
A7S6Z0	45351	A9PEK1	3694	Q8LMR0	39947
A8PPR4	6279	A9RC42	145481	C3Z0U0	7739
A8X4H6	6238	A9RIS6	145481	C5GXJ2	559297
A9SUV2	145481	A9RZW6	145481	Q9SHP0	3702
B3MVS7	7217	A9SKA3	145481	Q0WPU8	3702
B4FEZ7	4577	A9V4F4	81824	D6W9Q3	7070
B7QLV1	6945	B0CPN5	486041	C1GXN9	502779
B7ZWW7	4577	B0EH41	370354	B4FRI1	4577
B8C0X0	35128	B0WPU3	7176	B6TXR8	4577
B9RVV0	3988	B0XMR9	451804	D7L9G6	81972
B9SBC0	3988	B0XV36	451804	D7MCE6	81972
C3ZBR3	7739	B0Y361	451804	C8VNW8	227321
D2W1X5	5762	B0Y712	451804	B6RB66	91233
D6WN14	7070	B2ACR5	5145	B9H657	3694
P04694	10116	B2AQW9	5145	Q803I7	7955
P17735	9606	B2B5Q8	5145	Q6P2U8	7955
Q22UJ3	312017	B2VUB9	426418	A4RUK4	436017
Q4CVI0	5693	B2VUF1	426418	B8LRT6	3332
Q4CYW7	5693	B2W9P7	426418	A6QR28	9913
Q4D3Y8	5693	B3LRJ9	285006	B2W2S6	426418
Q4E4E7	5693	B3LTS5	285006	A8IH03	3055
Q4Q1I5	5664	B3MMW3	7217	C1BJA5	8014
Q54K95	44689	B3N4K2	7220	B9S6T4	3988
Q58CZ9	9913	B3RTC6	10228	Q96255	3702
Q8QZR1	10090	B4FWI4	4577	Q1EBU1	3702
Q93703	6239	B4G011	4577	Q8L7P0	3702
Q9FN30	3702	B4G9U8	7234	Q8LBA3	3702
A1L4G7	9606	B4HX27	7238	Q640D3	8355
A3CCA4	39947	B4JPK0	7222	Q28I26	8364
A5BY00	29760	B4KHA6	7230	A0JPC0	8364
A6H7B2	9913	B4LQG8	7244	B4K5X3	7230
A7TAK3	45351	B4MWE0	7260	P52877	3562
A9CM06	39947	B4P181	7245	A9UW97	81824
A9X448	226208	B4QAC2	7240	A0BLK8	5888
B0I503	4577	B5VHK9	545124	A2DW27	5722
B4F891	4577	B6H0R3	500485	A2FXW5	5722
B8A175	4577	B6HAI9	500485	A6SD91	332648
B8AFY0	39946	B6HFP1	500485	A7S471	45351
B8AFY1	39946	B6HTX7	500485	A8NH91	240176
B8LLE0	3332	B6JXX0	402676	A8PRI6	425265

B9F575	39947	B6K248	402676	A8Y1C7	6238
B9HGQ0	3694	B6QBG9	441960	B0D3P8	486041
B9NGM4	3694	B6QGR6	441960	B0EQ60	370354
B9RVU9	3988	B6QQP8	441960	B0WL19	7176
D3DWS2	9606	B6SKK1	4577	B3M1I2	7217
D3K4J1	3469	B6SL40	4577	B3P5J6	7220
D4NYR2	39984	B6SYR1	4577	B4GPH5	7234
D7F4K3	3656	B7FY72	556484	B4HZ76	7238
D7MTM9	81972	B7FY73	556484	B4JYI7	7222
P33447	5693	B8C3X1	35128	B4MBU3	7244
Q3UEH2	10090	B8LCI4	296543	B4NFM9	7260
Q4JR87	3880	B8LWC9	441959	B4PQ55	7245
Q4ZJF7	3847	B8M1U8	441959	B6GXM1	500485
Q5Z7R2	39947	B8MBX9	441959	B6KS29	508771
Q6K2Y8	39947	B8N110	332952	B7FRD6	556484
Q8GUE9	4142	B8NCW5	332952	B7Q0D6	6945
Q8H146	3702	B8NSD9	332952	B9TIH1	3988
Q8W462	3702	B9FCV6	39947	C5M730	294747
Q9QWS4	10116	B9G167	39947	C5M731	294747
Q9SMG7	4513	B9GNT1	3694	P91856	6239
Q9ST02	4513	B9HK69	3694	Q0V4D2	13684
Q9ST03	4513	B9HVD9	3694	Q16LP7	7159
Q9ST44	4513	B9MVW0	3694	Q22NW6	312017
Q9XSW4	452646	B9P8C8	3694	Q29BY2	46245
		B9P8U3	3694	Q4P2Y2	5270
		B9P9I0	3694	Q55CQ6	44689
		B9P9W6	3694	Q5BAM1	162425
		B9R765	3988	Q5KCD9	5207
		B9RYA3	3988	Q5TRW7	7165
		B9SG91	3988	Q60I38	5759
		B9W9J7	573826	Q68FU2	10116
		C0NE90	447093	Q99K85	10090
		C0NIB7	447093	Q9VAN0	7227
		C0NJP0	447093	Q9Y617	9606
		C0PKN2	4577	A2D968	5722
		C0PLN9	4577	B0ETT3	370354
		C0S590	482561	B0ETT4	370354
		C0SD24	482561	B1N376	5759
		C0SE91	482561	B4DHX7	9606
		C1E6U3	296587	B9PQI1	5811
		C1G4B3	502780	B9QLX4	432359
		C1GF60	502780	C4WYG7	7029
		C1GHI9	502780	C5L0M1	423536
		C1GN15	502780	C5LFP4	423536
		C1GW57	502779	C6T8T9	3847
		C1HD60	502779	D0MSZ0	403677

C1HDR7	502779
C1ML13	564608
C3YSM4	7739
C4J316	4577
C4JGI0	336963
C4JMI7	336963
C4JS89	336963
C4R1C8	644223
C4WRS4	7029
C4Y6R5	306902
C5DBS7	559295
C5DTI9	559307
C5FID3	554155
C5FUA8	554155
C5FV36	554155
C5G128	554155
C5GK58	559297
C5GM12	559297
C5GTT1	559297
C5GWS0	559297
C5JNH3	559298
C5JTY2	559298
C5JXG8	559298
C5JYN3	559298
C5MBK5	294747
C5P7X8	222929
C5PAN5	222929
C5PG42	222929
C5PGY2	222929
C5Y9E6	4558
C5YLQ3	4558
C5Z776	4558
C6HCU8	544712
C6HG97	544712
C6HJ26	544712
C6HNQ7	544712
C7GKK5	574961
C7GRR4	574961
C7YSG8	660122
C7YTE6	660122
C7YYH4	660122
C7Z561	660122
C8VG78	227321
C8VQV6	227321
C8Z793	643680
C8ZAH1	643680

D2I860	9646
D3TLH6	37546
P10658	9986
Q01CH3	70448
Q1HRL1	7159
Q2F5M8	7091
Q3U6K9	10090
Q3ULZ3	10090
Q543K5	10090
Q80YG1	10116
Q8BTJ1	10090
Q99JU9	10090

C9S872	526221
C9S898	526221
C9ST75	526221
D1ZVD8	5147
D2D316	3635
D2VJX3	5762
D3DLY8	559292
D3TPA7	37546
D3TRK9	37546
D4AUH4	663331
D4AW29	663331
D4AXG5	663331
D4CZF5	663202
D4D1K2	663202
D4DBZ4	663202
D4DGY5	663202
D5G7B2	39416
D5GIQ2	39416
D6VVL0	559292
D6WJ70	7070
D7KFI9	81972
D7L3K7	81972
D7L9Z5	81972
D7MF42	81972
O04130	3702
O08651	10116
O17626	6239
O43175	9606
O49485	3702
P40054	4932
P40510	4932
P87228	4896
Q00TL2	70448
Q019N3	70448
Q019N5	70448
Q0CA06	341663
Q0CR03	341663
Q0D094	341663
Q0DAG1	39947
Q0QJL3	49495
Q0U504	13684
Q0UFP9	13684
Q0VFV3	8364
Q17AF8	7159
Q1HR10	7159
Q25AL6	4530

	Q28CH6	8364	
	Q29M32	46245	
	Q2GSY9	38033	
	Q2GW64	38033	
	Q2GZK2	38033	
	Q2U0N3	5062	
	Q2UJX7	5062	
	Q2UTJ2	5062	
	Q4PF80	5270	
	Q4WHR3	5085	
	Q4WQ76	5085	
	Q4WTN4	5085	
	Q54UH8	44689	
	Q55YV2	5207	
	Q56WY7	3702	
	Q5A3K7	5476	
	Q5AS64	162425	
	Q5B1P6	162425	
	Q5BFA5	162425	
	Q5EAD2	9913	
	Q5K657	121759	
	Q5KN70	5207	
	Q5R7M2	9601	
	Q5RF12	9601	
	Q60HD7	9541	
	Q61753	10090	
	Q67W99	39947	
	Q6BWI7	4959	
	Q6C284	4952	
	Q6CTX5	28985	
	Q6FIP3	5478	
	Q6MY49	5085	
	Q6PHU9	7955	
	Q6ZAA5	39947	
	Q75CF2	33169	
	Q76KF5	5759	
	Q7PWU4	7165	
	Q7RWH3	5141	
	Q7XMP6	39947	
	Q871M4	5141	
	Q8C5X4	10090	
	Q8LGJ6	3702	
	Q9LT69	3702	
	Q9TXJ5	5664	
	Q9VKI8	7227	
**phosphoserine phosphatase**	**L-serine dehydratase**		**homoserine kinase**

**UniProt**	**TXID**	**UniProt**	**TXID**	**UniProt**	**TXID**
A0JCK1	51655	P25379	4932	A1C9S0	5057
A1CJC3	5057	D6VQV4	559292	A1CZI7	331117
A1D856	331117	C8Z427	643680	A2R9N8	425011
A3CD22	39947	B3LU22	285006	A2XBC6	39946
A3LND7	4924	C7GT17	574961	A3AD11	39947
A4QR74	148305	A6ZTD3	307796	A3LZ82	4924
A4S0L9	436017	B5VEP8	545124	A4HIP2	5660
A5ABM6	425011	A7TFC2	436907	A4I5Y9	5671
A5DIA2	4929	Q6FX72	5478	A4QUJ7	148305
A5DUQ3	36914	C5DYU2	559307	A5B5G1	29760
A6QXL5	339724	B2G4A8	4956	A5DJG5	4929
A6SIF0	332648	Q751S7	33169	A5E2A2	36914
A6ZUL9	307796	Q6CV26	28985	A6RK72	332648
A7EG80	665079	C5DJY9	559295	A6ZST1	307796
A7SA51	45351	C4R1X7	644223	A7E4T3	665079
A7TEU0	436907	A3LQ20	4924	A7TH47	436907
A8JEM3	3055	A3LN10	4924	A8I826	3055
A8QCA2	6279	Q6BZE0	4959	A8N251	240176
A8WQV3	6238	Q6BTC6	4959	A8Q9N6	425265
A9PCH8	3694	Q5A307	5476	A9T5Q1	145481
A9SSV1	145481	A4QUX7	148305	B0CQV8	486041
A9UX52	81824	A4R1F1	148305	B0Y391	451804
B0WEJ6	7176	Q55SE3	5207	B1X5R3	39717
B0XYY3	451804	Q5KGT7	5207	B2B725	5145
B2AUN4	5145	Q5K9Z3	5207	B2WL10	426418
B2LU26	9940	Q55JK8	5207	B3LSE6	285006
B2VRC7	426418	B9WAH5	573826	B4FKF4	4577
B3LI28	285006	B9WEK5	573826	B5VJV5	545124
B3M6G1	7217	B8M831	441959	B6HGD7	500485
B3NCF9	7220	B8MK73	441959	B6K3F7	402676
B3RK08	10228	C4XZ64	306902	B6KT41	508771
B4H1L4	7234	C4Y2U5	306902	B6QCY9	441960
B4HKQ3	7238	A6RLV9	332648	B6UB59	4577
B4IYA6	7222	A5DUW2	36914	B7G1B0	556484
B4KVV4	7230	A5DZC0	36914	B8C5L6	35128
B4LHQ1	7244	A7EL82	665079	B8LR75	3332
B4MKL7	7260	C7YLG5	660122	B8MC15	441959
B4NV80	7240	C7YTU4	660122	B8NSG6	332952
B4PEX1	7245	A1KYC1	6500	B9MZ08	3694
B4QN61	7240	B2WFR0	426418	B9Q0E9	5811
B5X9K1	8030	Q0CTG7	341663	B9QNJ0	432359
B6HFQ2	500485	Q0CLL4	341663	B9RVC5	3988
B6K6U9	402676	Q86B06	44689	B9RVC9	3988
B6QSZ5	441960	B2B5V5	5145	B9WH06	573826
B6SU38	4577	A1L2T1	8355	C0NIL8	447093

B7FV03	556484	A1L2R8	8355	C0S9H3	482561
B7PHY5	6945	Q6PAE9	8355	C1GC00	502780
B8BLF8	39946	Q5M8H3	8364	C1H0Q4	502779
B8BPT0	39946	C9SDW2	526221	C4R5X1	644223
B8BTL3	35128	D1Z612	5147	C4Y9P3	306902
B8MLU6	441959	Q8R238	10090	C4YQW2	5476
B8NUL7	332952	Q3UEN6	10090	C5DM98	559295
B9ELP5	8030	Q8VBT2	10090	C5E0V8	559307
B9NH06	3694	A2QI44	425011	C5FF32	554155
B9RLN8	3988	B3S1X5	10228	C5G891	559297
B9WAX5	573826	Q9C2I1	5141	C5JPN9	559298
C0NEA3	447093	D3B7Q8	670386	C5MGS8	294747
C0SDH0	482561	A7RWI2	45351	C5P8T2	222929
C1BLX3	8014	P09367	10116	C5XWS4	4558
C1GFM4	502780	D3ZHV7	10116	C6H7Y8	544712
C1H593	502779	C5PE25	222929	C7GTV5	574961
C1MZD9	564608	A5PKH5	9913	C7YS28	660122
C3KJZ8	229290	Q0VCW4	9913	C8V9I7	227321
C3YY11	7739	P20132	9606	C8ZA93	643680
C4JE76	336963	Q8WW81	9606	C9SB40	526221
C4QEG1	6183	Q96GA7	9606	D0MVQ7	403677
C4R7E7	644223	Q2GXT3	38033	D1ZMW5	5147
C4WU94	7029	B7PN84	6945	D2DKF1	3847
C4WU95	7029	A8NGQ4	240176	D3BU36	670386
C4Y8K3	306902	Q5B5G3	162425	D3DKX2	559292
C4YJL9	5476	A1CR51	5057	D4AZS6	663331
C5DC98	559295	B0XPH8	451804	D4DJ16	663202
C5DX25	559307	B6H8Y6	500485	D5GIV1	39416
C5FZJ1	554155	B8NST7	332952	D7L8T5	81972
C5GPL3	559297	Q2UUE9	5062	O43056	4896
C5JVC0	559298	Q4WJF6	5085	P17423	4932
C5KQK8	423536	Q6CPW0	28985	Q0CA33	341663
C5M656	294747	A0BE77	5888	Q0UEC5	13684
C5PIN9	222929	A0CM66	5888	Q2GY67	38033
C5Y6W1	4558	A0DHM0	5888	Q2UTL6	5062
C6HN58	544712	A1CHC6	5057	Q4CME2	5693
C6K7H8	51655	A1CLE7	5057	Q4DQQ2	5693
C6TJ83	3847	A1CXG1	331117	Q4P5L6	5270
C7GRP1	574961	A1D5X3	331117	Q4Q6Y2	5664
C7YML7	660122	A2FA06	5722	Q4WTR4	5085
C8Z9A1	643680	A2FRC0	5722	Q587A9	5691
C9JBI3	9606	A2QI80	425011	Q5AS87	162425
C9SNI7	526221	A3LW23	4924	Q5KBD4	5207
D0MT22	403677	A4H4N7	5660	Q6BNS6	4959
D1ZKP4	5147	A4HSW5	5671	Q6C1T8	4952
D2A0T2	7070	A4RI26	148305	Q6CNL8	28985

D3NQB6	6239
D3TSE0	37546
D4ASR0	663331
D4D4I9	663202
D5AB90	3332
D5G8E5	39416
D6VUZ1	559292
D7FPS7	2880
O74382	4896
O82796	3702
P42941	4932
P78330	9606
Q014M9	70448
Q0CC21	341663
Q0IHZ7	8364
Q0MYU8	2903
Q0U8Y6	13684
Q17M48	7159
Q17M49	7159
Q17M50	7159
Q26545	6183
Q29FA2	46245
Q2H472	38033
Q2KHU0	9913
Q2QQ95	39947
Q2R0W7	39947
Q2U4H1	5062
Q498V7	7955
Q4WWP0	5085
Q54ES3	44689
Q5A0H3	5476
Q5M819	10116
Q5RB83	9601
Q5RCQ7	9601
Q6BJA8	4959
Q6CDX6	4952
Q6CN02	28985
Q6DFU1	8355
Q6FND4	5478
Q75CP9	33169
Q7SFM8	5141
Q95Q10	6239
Q99LS3	10090
Q9U1W6	6239
Q9U1W7	6239
Q9VSY6	7227
A4S5M8	436017
A5DG69	4929
A5DWY6	36914
A6RUK3	332648
A6SSX5	332648
A7EEW4	665079
A7F680	665079
A7TKZ0	436907
A8BFR5	184922
A8I9R1	3055
A8N6Z1	240176
A8PY36	425265
A8WNC1	6238
A9TYA8	145481
B0D0M2	486041
B0E7X3	370354
B0WUZ1	7176
B3M069	7217
B3P1R0	7220
B4FIW6	4577
B4GP49	7234
B4HKF1	7238
B4JI58	7222
B4K4Y7	7230
B4LW10	7244
B4NAJ0	7260
B4PUM3	7245
B4QWN7	7240
B6GW79	500485
B6H312	500485
B6H461	500485
B7FV67	556484
B8N9L9	332952
B8NEE4	332952
B8P126	561896
B8PIK6	561896
B9MTL7	3694
B9SRK6	3988
B9WBR7	573826
C3Y9T1	7739
C4JHR3	336963
C4JWT5	336963
C4LUI9	5759
C4LVZ3	5759
C4Q4Y7	6183
C4QXV5	644223
Q6FPV4	5478
Q6K969	39947
Q75C47	33169
Q7FLV1	3702
Q8L7R2	3702
Q92209	5476
Q9HEA2	5141
Q9XEE0	3702

Q9XEQ8	4558	C4Y8M5	306902
		C5DCQ0	559295
		C5DQT2	559307
		C5M6K9	294747
		C5WN25	4558
		D2VDG8	5762
		D3DLZ2	559292
		O94634	4896
		Q0U0P1	13684
		Q0V4E4	13684
		Q17F43	7159
		Q17F44	7159
		Q21080	6239
		Q22B57	312017
		Q23ML3	312017
		Q23WP3	312017
		Q29BL4	46245
		Q2UGQ4	5062
		Q4P450	5270
		Q4WP28	5085
		Q4WP60	5085
		Q4WYW2	5085
		Q55JX2	5207
		Q5B6K0	162425
		Q5K9M8	5207
		Q6CAI4	4952
		Q6CMJ3	28985
		Q6FQM1	5478
		Q75BN9	33169
		Q7PQF6	7165
		Q7S456	5141
		Q86AP7	44689
		Q86ZL8	5145
		Q95XY8	6239
		Q9VHF0	7227
		Q9ZSS6	3702
		A0D3F4	5888
		A0D573	5888
		A0E6U7	5888
		A0FKE6	4081
		A1CH94	5057
		A1CXJ1	331117
		A1D414	331117
		A2FH13	5722
		A2QU10	425011
		A2XWA9	39946

A4QUE4	148305
A5HUN0	4513
A6RBB5	339724
A6RGX2	339724
A6ZR41	307796
A7A071	307796
A7RTQ7	45351
A8J977	3055
A9NZE1	3332
A9PI64	3694
A9T7L7	145481
B0EJK9	370354
B0EK81	370354
B0XZX9	451804
B0Y5X4	451804
B0Y607	451804
B1N2N4	5759
B1X3V5	39717
B2WAH4	426418
B2WP86	426418
B3LQP1	285006
B3LRK3	285006
B5VHL2	545124
B5VLU7	545124
B6H3S9	500485
B6QHL6	441960
B6QVU8	441960
B7FKK4	3880
B7G9B5	556484
B8C651	35128
B8CA45	35128
B8LQZ5	3332
B8LR80	3332
B8MJJ4	441959
B8MTE8	441959
B8N9Q5	332952
B8NXW6	332952
B9FB69	39947
B9GKJ4	3694
B9P9D4	3694
B9RC97	3988
B9TLI3	3988
C0NUL1	447093
C0NZL1	447093
C0RYW0	482561
C0SHC7	482561

C1E7U4	296587
C1G792	502780
C1GGJ3	502780
C1GX28	502779
C1GYL4	502779
C1MX69	564608
C1MZ44	564608
C4YIT5	5476
C5FMW6	554155
C5FSQ8	554155
C5GDJ4	559297
C5GRI2	559297
C5JIT2	559298
C5JRJ5	559298
C5P446	222929
C5PCI1	222929
C6H216	544712
C6HPI2	544712
C6LRX6	598745
C7GIZ5	574961
C7GRR8	574961
C7YIV4	660122
C7YRV2	660122
C7ZH37	660122
C8V6G4	227321
C8VPK7	227321
C8Z799	643680
C8ZBW2	643680
D0MT19	403677
D0N7P9	403677
D3B961	670386
D4AVK5	663331
D4DDK1	663202
D6VWY5	559292
D6WSN2	7070
D7L8V0	81972
D7LZN3	81972
O42615	37620
O59791	4896
P00927	4932
P25306	4081
P36007	4932
Q0CLP5	341663
Q0CS26	341663
Q10DZ5	39947
Q2GMH0	38033

Q2HGP8	38033
Q2PGG3	3702
Q2TYD7	5062
Q2U839	5062
Q2UGM2	5062
Q39469	3827
Q4QJ04	5664
Q59P56	5476
Q5B6G4	162425
Q5BAA5	162425
Q6BMV7	4959
Q6CE17	4952
Q6JAG1	4558
Q7XSN8	39947
Q86QZ7	5741
Q8GUG5	3702
Q8GW70	3702
Q8W314	39947
Q8X0J0	5141
Q9AUQ1	4530
Q9AXU4	49451
Q9T0D1	3702

**threonine synthase**		**3-deoxy-7-phosphoheptulonate synthase**		**3-dehydroquinate synthase**	
**UniProt**	**TXID**	**UniProt**	**TXID**	**UniProt**	**TXID**
A1CK16	5057	A0BP53	5888	Q8VYV7	3702
A1D7F2	331117	A0D7I3	5888	D7MN11	81972
A2ETD5	5722	A0DJH3	5888	Q30D01	28930
A2R773	425011	A0MH68	4058	Q8RU74	4081
A2ZWT5	39947	A1CCF4	5057	B9VU86	29760
A3LVC4	4924	A1CD93	5057	A5B6G3	29760
A4H7J4	5660	A1CHI4	5057	B9MXD6	3694
A4HVX7	5671	A1CS77	5057	A9PE59	3694
A4RLU0	148305	A1CTE9	5057	B9MXD7	3694
A4RVS2	436017	A1CUX4	331117	C6TGX4	3847
A4RZC1	436017	A1CX96	331117	B7FJL5	3880
A5DAD5	4929	A1D550	331117	A2Z3J8	39946
A5E244	36914	A1DC71	331117	B4FS26	4577
A6QTT7	339724	A2Q900	425011	B6SJH0	4577
A6S215	332648	A2QII3	425011	C5X6V7	4558
A6ZTN2	307796	A2R1A6	425011	Q651F0	39947
A7F7A3	665079	A2YNU0	39946	A9NUF2	3332
A7TT30	436907	A2YWD9	39946	B8LRA2	3332
A8HZZ1	3055	A2ZAB4	39946	A9RZH3	145481
A8NZN1	240176	A3C7D9	39947	A8J463	3055

A8QD54	425265
A9S5B7	145481
A9SSV5	145481
A9TLR9	145481
A9V4R9	81824
B0D421	486041
B0XY96	451804
B1X4H4	39717
B2B1N9	5145
B2G3X2	4956
B2G4S4	4956
B2VTP3	426418
B3LUB6	285006
B6HD87	500485
B6JVL7	402676
B6KQ11	508771
B6QVH8	441960
B6THZ8	4577
B6TPP7	4577
B7G9C6	556484
B8A807	39946
B8AWH3	39946
B8BRC6	296543
B8CBR8	35128
B8MTK3	441959
B8NMR6	332952
B9N1A4	3694
B9Q3R4	5811
B9QH30	432359
B9RK44	3988
B9S5U4	3988
B9WHF0	573826
C0NNN6	447093
C0P6C5	4577
C0RY59	482561
C1E0J2	296587
C1FFY5	296587
C1G6H2	502780
C1HA31	502779
C1MQ40	564608
C4JTQ2	336963
C4R057	644223
C4Y559	306902
C4YRB0	5476
C5DBY5	559295
C5DVQ5	559307
A3GG00	4924
A3LTF4	4924
A4QYK1	148305
A4RQ73	148305
A4RZ54	436017
A5C138	29760
A5DA85	4929
A5DHG5	4929
A5DUT1	36914
A5DWM2	36914
A6R0W3	339724
A6RB56	339724
A6REQ1	339724
A6RHV6	332648
A6RIN5	332648
A6RX11	332648
A6ZLI8	307796
A6ZY03	307796
A7EFK5	665079
A7EHE7	665079
A7F5B6	665079
A7TEB1	436907
A7TTE0	436907
A8J6Q7	3055
A8NJE5	240176
A8NR52	240176
A8PZM8	425265
A8Q6A5	425265
A8QAQ6	425265
A8QD78	425265
A9NUF5	3332
A9NUN2	3332
A9NZV0	3332
A9PF21	3694
A9RCS4	145481
A9RQX9	145481
A9RYQ7	145481
A9S036	145481
A9SGL2	145481
A9TK65	145481
A9TZP0	145481
A9VE96	81824
B0CVS7	486041
B0D7J1	486041
B0DV44	486041
B0XRH5	451804
C1FE63	296587
A4RXC8	436017
Q019I7	70448
B8BZI6	35128
C1MKZ4	564608
B9SUG6	3988
B7G177	556484
A1C9L3	5057
A4RGL5	148305
A6RS33	332648
A7ESN1	665079
B6TGN6	4577
B8BVV0	35128
C8V0N3	227321
Q4PE60	5270
Q5AME2	5476
A5H2P4	36914
A5H2Q8	36914
B1X3Q4	39717
B2VZE7	426418
B4G168	4577
B4G1Z7	4577
B7ZXL4	4577
B9WFG1	573826
C4R4R8	644223
C5M1X2	294747
C7ZD11	660122
C9SUJ9	526221
D5G8D9	39416
Q0UXE1	13684
Q5AMU6	5476
Q5AZ77	162425
Q6C1X5	4952
Q8LFK9	3702
Q9FKX0	3702

C5FH22	554155	B0Y669	451804
C5GIS3	559297	B0YCB5	451804
C5JGG1	559298	B0YF87	451804
C5KRV1	423536	B1X4G2	39717
C5MHG4	294747	B2AAC1	5145
C5P123	222929	B2AQP5	5145
C5XHG5	4558	B2AYX6	5145
C5YUZ5	4558	B2VX29	426418
C6HFX5	544712	B2VZZ7	426418
C7GJT3	574961	B2WBX0	426418
C7Z694	660122	B2WF53	426418
C8VIU2	227321	B3LGN0	285006
C8Z4E5	643680	B3LMR3	285006
C9ZT46	679716	B4FMU0	4577
D0NJP0	403677	B4FZ59	4577
D1ZU84	5147	B5VEJ9	545124
D3BUK1	670386	B5VFT8	545124
D4B5N4	663331	B6HB61	500485
D4D679	663202	B6HMQ2	500485
D5GJK7	39416	B6HTS2	500485
D6VR61	559292	B6JV24	402676
D7KR40	81972	B6K4L8	402676
D7MCH0	81972	B6KQ33	508771
P16120	4932	B6Q8J3	441960
Q00063	33169	B6QE84	441960
Q01B87	70448	B6QLB1	441960
Q0CIS5	341663	B6T4J7	4577
Q0UMX8	13684	B6TUB8	4577
Q2GV42	38033	B6UAK5	4577
Q2Q021	3847	B7FRJ9	556484
Q2U324	5062	B8ARY1	39946
Q42598	4896	B8BVC7	35128
Q4DIK8	5693	B8LDP4	296543
Q4DLL4	5693	B8LMP9	3332
Q4P6C2	5270	B8LZB9	441959
Q4QFV0	5664	B8MB35	441959
Q4WXC3	5085	B8MB36	441959
Q57X55	5691	B8MB37	441959
Q5AGE6	5476	B8MHZ1	441959
Q5B8U9	162425	B8MVY2	332952
Q5KMM7	5207	B8MYJ8	332952
Q66RI3	4932	B8N9E7	332952
Q6BKV1	4959	B8PCB8	561896
Q6C0N3	4952	B9GUY5	3694
Q6CTY8	28985	B9MUW0	3694
Q6FMB9	5478	B9PYJ3	5811

Q6L4H5	39947	B9RJN2	3988
Q8LJJ2	39947	B9SZ06	3988
Q8X0K4	5141	B9T0A7	3988
Q96VZ4	5207	B9VU85	29760
Q9MT28	4113	B9W7T3	573826
Q9S7B5	3702	B9WAP2	573826
Q9SSP5	3702	C0NQD6	447093
		C0NV96	447093
		C0P1C9	447093
		C0RZ23	482561
		C0S2T2	482561
		C0SFV6	482561
		C1DYV8	296587
		C1G0J6	502780
		C1G7F9	502780
		C1GS88	502779
		C1GXV4	502779
		C1H5Y6	502779
		C1N1Q4	564608
		C4J284	4577
		C4JG84	336963
		C4JJ23	336963
		C4JU93	336963
		C4QXT0	644223
		C4R611	644223
		C4XWJ8	306902
		C4XX57	306902
		C4YED0	5476
		C5DE97	559295
		C5DX11	559307
		C5E1L8	559307
		C5E2I8	559295
		C5FC05	554155
		C5FGE6	554155
		C5GPH0	559297
		C5GSH6	559297
		C5GTI8	559297
		C5JDU9	559298
		C5JL00	559298
		C5JWU9	559298
		C5LMC8	423536
		C5M0P6	423536
		C5M6E4	294747
		C5MEN4	294747
		C5NZE4	222929
		C5P018	222929

C5PJP4	222929
C5WRG8	4558
C5WXQ3	4558
C5X2H5	4558
C5YJQ4	4558
C6H299	544712
C6HHK1	544712
C6HS65	544712
C6TJC2	3847
C7GMY2	574961
C7GQ13	574961
C7YHC2	660122
C7YLD6	660122
C7YWA2	660122
C8VFP0	227321
C8VNS1	227321
C8Z4V7	643680
C9K7C8	5599
C9SIM7	526221
C9SQE6	526221
C9SQE7	526221
D0MU15	403677
D0NYT8	403677
D0VBC5	29760
D1Z3F5	5147
D3UEZ6	643680
D3YIJ4	119830
D4ANK0	663331
D4ANV0	663331
D4CZ80	663202
D4DBP2	663202
D5G4I3	39416
D5G834	39416
D5GF45	39416
D6RL96	240176
D6VQP5	559292
D6VS23	559292
D7KLQ8	81972
D7M8Q4	81972
D7M981	81972
O22407	4043
O24046	43522
O24051	43522
O24052	43522
P14843	4932
P21357	4113

P27608	4097
P29976	3702
P32449	4932
P34725	5476
P37215	4081
P37216	4081
P37822	4113
P79023	5476
P80576	5141
Q00218	3702
Q09755	4896
Q0CB67	341663
Q0CN52	341663
Q0D4J5	39947
Q0DRF3	39947
Q0UWH1	13684
Q0V1K6	13684
Q0V4Y8	13684
Q0VHY4	5911
Q10KC0	39947
Q2A710	120017
Q2GTA2	38033
Q2HDD0	38033
Q2HG98	38033
Q2UGW7	5062
Q2URC2	5062
Q2UTB3	5062
Q30D02	28930
Q336R7	39947
Q4G2J0	71150
Q4P9Q3	5270
Q4PBX8	5270
Q4PD03	5270
Q4W9G6	5085
Q4WG78	5085
Q4WKK9	5085
Q4WPC4	5085
Q55MJ1	5207
Q55W33	5207
Q570U9	3702
Q5ALQ9	5476
Q5BCQ7	162425
Q5BGH6	162425
Q5EMW0	148305
Q5K879	5207
Q5KHW1	5207

Q5KK60	5207
Q6BIP1	4959
Q6BZ40	4959
Q6CCS4	4952
Q6CDP1	4952
Q6CDZ3	4952
Q6CJG9	28985
Q6CL12	28985
Q6FRA6	5478
Q6FX01	5478
Q6YTT3	39947
Q754W4	33169
Q75DX5	33169
Q75LR2	39947
Q75W16	39947
Q7S715	5141
Q7SCQ0	5141
Q7YW56	5811
Q8H708	4787
Q8H9C9	4113
Q8LAR5	3702
Q9AY42	4530
Q9HEZ8	162425
Q9HEZ9	162425
Q9SK84	3702
Q9UT09	4896

**Pentafunctional AROM polypeptide**		**3-dehydroquinate dehydratase I/shikimate 5-dehydrogenase**		**shikimate kinase**	
**UniProt**	**TXID**	**UniProt**	**TXID**	**UniProt**	**TXID**
P08566	4932	Q9SQT8	3702	A8MRG1	3702
D6VSB3	559292	D7L5G1	81972	Q9SJ05	3702
C8Z543	643680	B9RSY7	3988	Q8GY88	3702
B3LGE9	285006	B9HSF2	3694	Q9SVA4	3702
A6ZY89	307796	Q30D00	28930	D7LCM2	81972
C7GIN5	574961	A5ATW2	29760	Q30CZ9	28930
C5DVG6	559307	D2KBA9	4113	A5AZY1	29760
Q6FIV4	5478	O65917	4081	B9VU88	29760
C5DN02	559295	Q6PUF9	4097	B9HG21	3694
Q6CJC4	28985	C8KH58	35925	Q00497	4081
Q74ZZ1	33169	B8LLR2	3332	C6TLJ4	3847
C5M1X2	294747	A9NUR4	3332	B4F8E9	4577
C4R4R8	644223	B6TSW1	4577	B6TNA7	4577
C4Y9D5	306902	B4FAK2	4577	Q7X7H9	39947
B9WFG1	573826	B4FH30	4577	Q01HY3	4530

A3LSZ2	4924	B4FN22	4577	Q5JQX9	4530
Q5AMU6	5476	B6UCV5	4577	B8B486	39946
Q5AME2	5476	B8BM99	39946	A2X9M5	39946
A5H2Q8	36914	B8A8A3	39946	B8AV75	39946
A5H2P4	36914	C5YPB6	4558	C5Y8Y1	4558
Q6C1X5	4952	C5XLB0	4558	B9T204	3988
C7YZ74	660122	B9GDI1	39947	A9NUN9	3332
D5G8D9	39416	Q5ZCV1	39947	A8IGF0	3055
Q8X071	5141	A9S495	145481	A9SQ82	145481
D1ZA70	5147	C1FDQ3	296587	C1EGE2	296587
A7F7H0	665079	A8IZ72	3055	B7GAC8	556484
B2B223	5145	A4S5X6	436017	B8C8C4	296543
Q5XNP0	5180	Q00XP3	70448	D7M8K9	81972
Q9P7R0	4896	B3VLC1	332125	Q00VP6	70448
A4RD09	148305	B9I9X4	3694	B9GT47	3694
B6JVD0	402676	B9REX9	3988	Q0DXJ9	39947
C9SE96	526221	A2WQ65	39946	Q5AME2	5476
B6QCA7	441960	A5ATW0	29760	A5DK69	4929
B6QWH9	441960	A5ATW1	29760	A5H2P4	36914
A1D244	331117	B9I835	3694	A5H2Q8	36914
B8M0U4	441959	B9MZN1	3694	B4FPS0	4577
B8N4Q9	332952	B9REY0	3988	B6UDH4	4577
C5PA86	222929	B9REY1	3988	B9F302	39947
Q4WS76	5085	B9REY2	3988	B9FSA8	39947
Q8TFZ0	5085	B9S3X5	3988	B9NKZ2	3694
B0XRM8	451804	C5YPB7	4558	B9WFG1	573826
B6HAA7	500485	Q0JMN4	39947	C0PNT9	4577
P07547	162425	Q6PUG0	4097	C4R4R8	644223
Q2UCP6	5062			C5M1X2	294747
A1CP85	5057			C6THS3	3847
Q0D0F3	341663			D5G8D9	39416
D4D596	663202			Q5AMU6	5476
D4ALM9	663331			Q5NTH3	39947
C5FQ73	554155			Q5NTH4	39947
C0S433	482561			Q6C1X5	4952
Q0V3H0	13684				
A5DK69	4929				
C4JYG6	336963				
C5JKE6	559298				
C5G8R4	559297				
C0NL63	447093				
C6HCG7	544712				
Q8J294	107832				
B0D6H2	486041				
Q5KME5	5207				
Q2GP00	38033				

Q12659	4754
A8NMB4	240176
Q4P8F6	5270
A8QCB2	425265
A9V1N3	81824
D0NXA2	403677
C1FYJ9	502780
C1H8L1	502779
A6R8Q8	339724
B2VVY2	426418
Q0VIN9	5911
A0C9W9	5888
A0DH09	5888
C5LBY1	423536
B7FY07	556484
A5DK70	4929
B2VVY3	426418
B8BW72	35128
C8VRD2	227321

3-phosphoshikimate 1-carboxyvinyltransferase

UniProt	TXID
Q9FVP6	3702
D7KD66	81972
A4SB81	436017
A8JH48	3055
A9SYF9	145481
B7FSS5	556484
B8BW66	35128
B9GPE8	3694
B9RDA3	3988
C5Z3D1	4558
P05466	3702
Q5AME2	5476
Q5VNW0	39947
A2T9R8	3046
A2Y8X9	39946
A3B841	39947
A5C3Y3	29760
A5DK69	4929
A5H2P4	36914
A5H2Q8	36914
A7Y7Y2	3635
A9P0E4	3332

chorismate synthase

UniProt	TXID
P28777	4932
D6VU01	559292
C8Z8A2	643680
C7GPQ5	574961
B5VII2	545124
B3LHK3	285006
A6ZU35	307796
Q6FV38	5478
Q6CNY1	28985
C5DL16	559295
A7TIP8	436907
C5DPE2	559307
Q75B59	33169
C4XZV9	306902
Q6BQN1	4959
A5DJM9	4929
C4R1A7	644223
B9WMG9	573826
Q59K34	5476
C4YMI5	5476
A3LYZ8	4924
C5M8C8	294747

bifunctional indole-3-glycerol phosphate synthase/anthranilate synthase component II

UniProt	TXID
P00937	4932
D6VWZ2	559292
C8ZBW9	643680
B5VLV6	545124
B3LQP8	285006
A7A078	307796
C7GJ02	574961
C5DZ89	559307
Q6FUV9	5478
A7TE28	436907
Q6CV69	28985
C5DC85	559295
Q6C9H7	4952
C4R637	644223
Q755S2	33169
P20409	4837
B9W9L6	573826
Q5A3M7	5476
C4YGA0	5476
C5MBQ8	294747
D5GHJ8	39416
B2AXK8	5145

B2C1I9	3635	A5E0D5	36914	P00908	5141
B2CM20	480913	Q6C8Q1	4952	Q2H6W2	38033
B3GGJ9	4123	B6H629	500485	C9SP56	526221
B5M6A1	4565	D5GMG1	39416	B2WJ92	426418
B8XU22	3635	B6K4C9	402676	A7F349	665079
B9WFG1	573826	A1CQW8	5057	A6RML4	332648
C1EG02	296587	O74413	4896	C7YYQ5	660122
C1MWX5	564608	C0S9E6	482561	C4XX83	306902
C4R4R8	644223	A6RUL9	332648	A3LYT4	4924
C5M1X2	294747	Q4WJ77	5085	Q0UCF0	13684
C8YXI5	107608	B0XPR6	451804	A4R8N0	148305
C8YXI6	107608	C1GBW5	502780	C4JLE2	336963
C8YYL6	277990	C5JIX7	559298	C5FHC2	554155
C8YYL7	277990	C5GEA6	559297	P24773	5076
D0VBC7	29760	A1D3T1	331117	B6H5A3	500485
D3JPP9	309978	C5P8X2	222929	B6QCL2	441960
D3JPQ0	309978	A7EEZ6	665079	B8M271	441959
D5G8D9	39416	B8ML69	441959	A5DSY8	36914
D7LCH4	81972	B8LU03	441959	A1CPF3	5057
O80428	39947	B6QWH7	441960	C5JGB7	559298
P10748	4081	B6Q851	441960	C5GII0	559297
P11043	4102	C5FEW3	554155	Q0D0N0	341663
P17688	3708	C0NGC3	447093	C5P5A9	222929
P23981	4097	C4JJX7	336963	P05328	5061
Q00VW9	70448	Q12640	5141	D4DA85	663202
Q0PKR0	3885	A6R7D8	339724	D4ARE5	663331
Q1KPV4	91198	C6H8B7	544712	A1D2B1	331117
Q1M157	16922	Q5B149	162425	B0XR95	451804
Q30CZ8	28930	C8VFK9	227321	Q6MYH9	5085
Q3ECU2	3702	D1ZLW0	5147	Q6BWE2	4959
Q587N9	3847	Q2UMA1	5062	C1HD27	502779
Q5AMU6	5476	B8N4L8	332952	A2QRH6	425011
Q6C1X5	4952	A4R7D4	148305	C6HFS8	544712
Q6E6L4	72917	Q2GNE9	38033	C0NNJ0	447093
Q6E6L5	72917	B2W1P0	426418	C1GJ38	502780
Q6JDV0	3711	A8N225	240176	C0S7N5	482561
Q71LY8	3847	C8Z2I6	100787	P18483	105351
Q8LC97	3702	A8PWP1	425265	Q2UCI0	5062
Q8W1M5	71234	B8P2V9	561896	Q96VL5	71696
Q93VK6	4530	B0CQS1	486041	B8N4X9	332952
Q946U9	173162	C7YR83	660122	D1ZBC5	5147
Q946V0	173162	Q5KCY4	5207	P06531	162425
		D4D2D9	663202	Q92370	4896
		C1HAK5	502779	Q76JQ3	5334
		D4AVG4	663331	A5DER2	4929
		B2B197	5145	A8N699	240176

Q0UTJ3	13684
A9V1N2	81824
B9SUA2	3988
Q10NY1	39947
B8AKA5	39946
B4FLA2	4577
B6T4U1	4577
B4FRH6	4577
B4FTP6	4577
C5WQV1	4558
B5AAU3	112509
Q30CZ7	28930
Q42884	4081
Q42885	4081
B8LL06	3332
B8BT00	296543
A9RTV8	145481
P27793	3464
B7FS76	556484
A0DHI8	5888
A1XYU5	39717
A5APN1	29760
B9VU84	29760
A8J2Z6	3055
A9PGP7	3694
B9HLM0	3694
C1E5R8	296587
A4RTB7	436017
D7KD60	81972
Q0WUP7	3702
P57720	3702
Q2V4H9	3702
Q2V4I0	3702
Q38RG6	3847
C1MMY6	564608
A7T8F5	45351
B3L7H9	5851
B6KIM6	508771
C6KT66	36329
Q23FV7	312017
Q4PER1	5270
Q4XVI2	5825
Q4Z596	5821
Q7RHB8	73239
Q8WRC8	5855
B9PUU8	5811
Q96WP2	5341
B6JXZ5	402676
P25170	5306
Q6KCN7	76867
Q873X5	5530
P09575	4905
B0D0Y7	486041
Q9HGK1	38945
Q5KC22	5207
Q4PBY7	5270
A8PZV8	425265
Q92411	5016
C8VS06	227321
A6QTP2	339724
B8M8Z8	441959
B8M8Z9	441959
P48261	2762

B9QG39	432359
C9SJN6	526221
D0MU16	403677
O02607	5811
O15864	5833
Q0CTR0	341663
Q4X9S6	5825

anthranilate phosphoribosyltransferase

UniProt	TXID
A1CIC4	5057
A1CWD6	331117
A2Q9B1	425011
A2XBZ0	39946
A3GEY1	4924
A4QT64	148305
A4RYP9	436017
A5B8K2	29760
A5DM37	4929
A5E7Q9	36914
A6QVU0	339724
A6RR68	332648
A6ZYV4	307796
A7EN33	665079
A7THU8	436907
A8IBN0	3055
A8NRZ3	240176
A8PWM8	425265
A9T6I7	145481
B0DQ35	486041
B0Y4G1	451804
B2B367	5145
B2VTL9	426418
B3LFU7	285006
B4FB65	4577
B5VGN6	545124
B6HUC6	500485
B6K3I2	402676
B6QQI1	441960
B7G4F0	556484
B8A0L5	4577
B8LWE3	441959
B8N131	332952
B9NGG3	3694
B9RB03	3988
B9WF64	573826

phosphoribosylanthranilate isomerase

UniProt	TXID
A3GFA8	4924
A4Z4U7	4911
A5DK25	4929
A5DVR7	36914
A6ZXX3	307796
A7T441	45351
A7TI73	436907
A7ULH8	272698
A8DWA1	45351
A8J650	3055
A9UKH6	4905
B3H4J9	3702
B3LGQ1	285006
B4FHC1	4577
B4FZQ3	4577
B5VFR6	545124
B6U7W9	4577
B7FWK1	556484
B8AFB5	39946
B9HHQ7	3694
B9I455	3694
B9SQM9	3988
B9W6H2	573826
C4J8W8	4577
C4QV11	644223
C4Y5L4	306902
C4YFW5	5476
C5DNE4	559295
C5DPN7	559307
C5MDZ4	294747
C5XZK5	4558
C5Z371	4558
C7GXY3	574961
C8Z4S7	643680
D6VRZ5	559292
D7LYY8	81972

indole-3-glycerol phosphate synthase

UniProt	TXID
P49572	3702
Q1EBW5	3702
Q8GYM9	3702
Q9LUB2	3702
A8MS79	3702
D7L7H9	81972
B9G099	39947
Q8H3R5	39947
Q6K307	39947
Q6K306	39947
C1N2H5	564608
D7ML37	81972
B9T1U0	3988
B7FI09	3880
C0PGV5	4577
B4G1Z5	4577
C5YA91	4558
Q01IS1	4530
Q7XJW1	4530
A9NUS4	3332
B8AV46	39946
B9IAU9	3694
A9TUX8	145481
C1DZA7	296587
A4RZE7	436017
A8HT60	3055
B4FS35	4577
B6UGU8	4577
B8CAE6	35128
C5X8Y0	4558
C5Y188	4558
B4FLA1	4577
B7FXG0	556484
B8B9Q2	39946
B8BDF0	39946
B9FFL9	39947

C0NJM9	447093
C0S5A0	482561
C1E0Z8	296587
C1G4C3	502780
C1GW67	502779
C4JSB2	336963
C4R7F6	644223
C4Y377	306902
C5DJH7	559295
C5DY18	559307
C5FU89	554155
C5GWR0	559297
C5JXH8	559298
C5PG24	222929
C5X013	4558
C6HJ38	544712
C7GMI0	574961
C7YH07	660122
C8V439	227321
C8Z5R5	643680
C9S867	526221
D0NUM7	403677
D1ZI58	5147
D4AW48	663331
D4DBX8	663202
D5G442	39416
D6VSY4	559292
D7FW59	2880
D7LC24	81972
D7LDH9	81972
D7LXP9	81972
O60122	4896
P07285	4932
Q017G3	70448
Q02166	3702
Q0CR19	341663
Q0UME3	13684
Q10SD5	39947
Q10SD6	39947
Q2H180	38033
Q2UJZ6	5062
Q4PH95	5270
Q4WQ89	5085
Q547D7	3702
Q5A0Z7	5476
Q5A168	5476
O13504	4922
P00912	4932
P13997	28985
P43073	5476
P50857	5478
Q01128	29829
Q1M2R6	37620
Q39245	3702
Q39246	3702
Q3E9L0	3702
Q42440	3702
Q42527	3702
Q42528	3702
Q5AB75	5476
Q5XQP9	114524
Q6BY13	4959
Q6ETX4	39947
Q6UYC0	3702
Q757J9	33169
Q875I3	4927
Q8LPI9	3702
Q8VY51	3702
Q8X1D9	4952
Q9FFF7	3702
Q9HFW8	4954
Q9S731	3702
Q9SPV7	3702
Q9SPV8	3702
Q9SPV9	3702
Q9SPW0	3702
Q9SPW1	3702
B9NGG4	3694
C0P9Q8	4577
C4J6J4	4577
C5YJF0	4558
C8TFG9	39946

Q5B746	162425
Q5KN34	5207
Q6BI72	4959
Q6C7L2	4952
Q6CMB3	28985
Q6FY26	5478
Q756L5	33169
Q7RZ63	5141
Q8S5W5	39947

**tryptophan synthase**		**anthranilate synthase component I**		**anthranilate synthase component II**	
**UniProt**	**TXID**	**UniProt**	**TXID**	**UniProt**	**TXID**
A1C701	5057	P00899	4932	Q9FXK1	3702
A1C8N4	5057	D3DLZ7	559292	Q9FE37	3702
A1CUJ3	5057	C8Z7A6	643680	A4S3X3	436017
A1DHM9	331117	C7GRS3	574961	A8HP28	3055
A1DLE7	331117	B5VHL7	545124	A9TZ60	145481
A1DMZ6	331117	B3LRK8	285006	B6UGS8	4577
A1DP73	331117	A6ZR46	307796	B9HVR6	3694
A2QC38	425011	Q6FQM6	5478	B9SNH9	3988
A2QZ07	425011	A7TMV6	436907	C5WN01	4558
A2R771	425011	C5DQU0	559307	Q42565	3702
A2RAW9	425011	Q6CMI3	28985	Q764B9	39947
A2XMX3	39946	C5DE84	559295	Q7XUS2	39947
A2XMX4	39946	C5M4E1	294747	Q9FJM5	3702
A2XMX5	39946	Q6BYR7	4959	A1CPF3	5057
A2YFA8	39946	A3M0I8	4924	A1D2B1	331117
A2YIT3	39946	B9WKY8	573826	A2QRH6	425011
A2YR01	39946	C4YKZ6	5476	A2XUC4	39946
A3ANM5	39947	Q5A9L8	5476	A4R8N0	148305
A3ANM6	39947	Q5A9B9	5476	A6QTP2	339724
A3BDS5	39947	C4XWR7	306902	A6RML4	332648
A3BH76	39947	C4QZU0	644223	A7F349	665079
A3GH63	4924	A5DEH6	4929	B0XR95	451804
A4R8A0	148305	Q75D13	33169	B1X4B6	39717
A4RVE9	436017	Q6C5V8	4952	B2AXK8	5145
A4S0C1	436017	Q2ULK9	5062	B2WJ92	426418
A5AI98	29760	B8N253	332952	B6H5A3	500485
A5E4I2	36914	A2QLM4	425011	B6QCL2	441960
A6RAG5	339724	A1CU99	5057	B6T2R9	4577
A6RUK6	332648	C1H2L2	502779	B6TJE4	4577
A6ZUW8	307796	C5JD45	559298	B7FJB9	3880
A7ES09	665079	C5GVJ5	559297	B8AQP1	39946
A7TKX5	436907	B8M905	441959	B8M271	441959
A7UX21	5141	B8MAL9	441959	B8M8Z8	441959
A8IFZ9	3055	C1GB53	502780	B8M8Z9	441959

A8J599	3055	C0S905	482561	B8N4X9	332952
A8NEP3	240176	A1DNY2	331117	B9FFK4	39947
A8PRB2	425265	Q4WLR6	5085	B9TB57	3988
A9NUV8	3332	B0Y8U4	451804	C0NNJ0	447093
A9PGZ5	3694	C4JSG9	336963	C0PIP8	4577
A9PJJ9	3695	Q0CSS2	341663	C0S7N5	482561
A9RDX2	145481	B6QDK2	441960	C1ECT8	296587
A9RJ02	145481	A6R6G6	339724	C1GJ38	502780
A9RJP2	145481	D4DH22	663202	C1HD27	502779
A9RTF1	145481	A4R7P3	148305	C1N9X4	564608
A9S4R5	145481	C5PFW8	222929	C4JBA3	4577
A9V2F2	81824	C5FL75	554155	C4JLE2	336963
B0CS16	486041	C9SKS1	526221	C4R637	644223
B0XM82	451804	C8V7L2	227321	C5DC85	559295
B0XTB9	451804	C0NHA0	447093	C5DZ89	559307
B0ZSJ6	3847	A7E6R5	665079	C5FHC2	554155
B2ACV4	5145	D5GN36	39416	C5GII0	559297
B2W653	426418	Q5B6Y5	162425	C5JGB7	559298
B2Y0K4	4577	B6HT18	500485	C5P5A9	222929
B3LIN9	285006	O94582	4896	C6HFS8	544712
B4F800	4577	C6H6M2	544712	C7YYQ5	660122
B4FGW7	4577	B6K2N8	402676	C9SP56	526221
B4FIR7	4577	Q0UL85	13684	D4ARE5	663331
B4FTB8	4577	Q4PHV9	5270	D4DA85	663202
B6HLH5	500485	A8NHG9	240176	D5GHJ8	39416
B6HT75	500485	B0D3J2	486041	D7KAK0	81972
B6K8C9	402676	A5DS63	36914	D7LCU7	81972
B6QC07	441960	D4ATA7	663331	D7LP35	81972
B6SCC6	103766	B2ADS2	5145	D7LQ09	81972
B6SCC7	103766	Q55R50	5207	P00908	5141
B6SCC8	565971	Q5KF19	5207	P05328	5061
B6SCC9	565971	Q872P4	5141	P06531	162425
B6SCD0	565971	C7Z803	660122	P18483	105351
B6SCD2	53161	A8PQY2	425265	P20409	4837
B6TI69	4577	D1ZJV6	5147	P24773	5076
B6TS31	4577	D0MTW7	403677	P48261	2762
B6U662	4577	B2W891	426418	P51362	2787
B7FQI2	556484	Q2GU28	38033	Q00ZS5	70448
B8ALB1	39946	A6S6K0	332648	Q0D0N0	341663
B8CEN4	35128	A9SGW3	145481	Q0UCF0	13684
B8LQ55	3332	A9T285	145481	Q1XDC5	2788
B8M3L9	441959	A9T2B2	145481	Q25A90	4530
B8MZR1	332952	B9SI04	3988	Q2H6W2	38033
B8N9Y5	332952	B9SLL9	3988	Q2UCI0	5062
B8NGL5	332952	C5WU43	4558	Q4PBY7	5270
B8NW60	332952	P32068	3702	Q5KC22	5207

B9GS77	3694
B9H8G5	3694
B9HZK8	3694
B9I0B9	3694
B9I7M4	3694
B9PET1	3694
B9PFJ8	3694
B9RXQ0	3988
B9S4R8	3988
B9SAI8	3988
B9WGQ5	573826
C0HGY6	4577
C0NSJ2	447093
C0P7Z2	4577
C0SC97	482561
C1DY75	296587
C1DZ74	296587
C1FGK1	296587
C1GI69	502780
C1H5U4	502779
C1MQU0	564608
C4J9I9	4577
C4JV91	336963
C4R1F2	644223
C4R547	644223
C4XZK1	306902
C5DHV1	559295
C5E048	559307
C5FPG0	554155
C5GT68	559297
C5JLJ6	559298
C5MCT3	294747
C5P0D6	222929
C5WX66	4558
C5WX67	4558
C5WX69	4558
C5XB29	4558
C5Z690	4558
C6HDU0	544712
C6T703	3847
C6TF21	3847
C6THT5	3847
C6TKV4	3847
C7GS99	574961
C7Z1A2	660122
C8V1R2	227321
P32069	3702
Q10B97	39947
Q45KJ2	4577
Q9XJ29	39947
A2XNK3	39946
A5BV77	29760
A6S6J9	332648
B1X521	39717
B4F8P4	4577
B8AKD7	39946
B9F770	39947
C1E0B2	296587
C1MRE5	564608
C4JBB1	4577
D7LLC3	81972
D7LV96	81972
D7LZ03	81972
O81533	4097
Q015Q7	70448
Q0KIX3	4498
Q0KIX4	4498
Q0KIX5	4498
Q10NN4	39947
Q10NN5	39947
Q10NN7	39947
Q10NN8	39947
Q41155	37565
Q41156	37565
Q5XLF2	16922
Q5XLF3	16922
Q84QB7	39947
Q944Q5	3702
Q94GF1	4530
Q9AW95	4058
Q9LY58	3702
Q9XJ30	39947
Q6B925	285951
Q6MYH9	5085
Q873X5	5530
Q8L9I8	3702
Q92370	4896
Q92411	5016

C8VGH6	227321
C8Z8L6	643680
C9SFC8	526221
D0AB52	39946
D0AB53	39946
D0N5Q5	403677
D1ZU10	5147
D3AW64	670386
D4AMB4	663331
D4D9I8	663202
D5ABD1	3332
D5G583	39416
D5GHY3	39416
D6RKG0	240176
D6VUB2	559292
D7KMG3	81972
D7LUW2	81972
D7M3G0	81972
D7MEJ4	81972
D7MJX6	81972
D7MUW5	81972
O04225	4530
O13831	4896
O22765	3702
O50046	16922
P00931	4932
P13228	5141
P14671	3702
P16578	5346
P25269	3702
P42390	4577
P43283	4577
P43284	4577
Q014Y8	70448
Q01BL5	70448
Q0CF14	341663
Q0CIQ9	341663
Q0CYD3	341663
Q0ULK5	13684
Q0WS13	3702
Q0WUI8	3702
Q0WWE0	3702
Q2GMW7	38033
Q2TX96	5062
Q2UFA5	5062
Q2UGE4	5062

Q2UQB8	5062
Q42529	3702
Q4P5M9	5270
Q4WLH4	5085
Q4X0J6	5085
Q55Z78	5207
Q56YC7	3702
Q58A35	4565
Q58A36	4565
Q59KP3	5476
Q5AZP9	162425
Q5B1Y6	162425
Q5CDU1	237895
Q5KNJ3	5207
Q5KT13	96455
Q659I8	161756
Q67VM1	39947
Q67VM2	39947
Q6BS14	4959
Q6C0G7	4952
Q6CNQ8	28985
Q6FXZ3	5478
Q6YYB9	39947
Q6ZL61	39947
Q752T8	33169
Q7XAK6	4565
Q7Y1H9	39947
Q7Y1I6	39947
Q7Y1I9	39947
Q7Y1J1	39947
Q874B5	96454
Q8GWQ8	3702
Q8LA83	3702
Q8W0T4	4558
Q9FFW8	3702
Q9FQ75	4577
Q9FQ77	4577
Q9P4D5	162425

**pyruvate dehydrogenase**		**GDH2**		**GLUD1**	
**UniProt**	**TXID**	**UniProt**	**TXID**	**UniProt**	**TXID**
A0BMJ5	5888	P33327	4932	A0AG12	386043
A0BWV5	5888	D6VRD9	559292	A0AQX1	1599
A0BYJ3	5888	B3LH95	285006	A0BLL2	5888
A0C608	5888	A6ZXB8	307796	A0BRD6	5888
A0C609	5888	C8Z6Q4	643680	A0D0R3	5888

A0CAD2	5888	C7GSW2	574961	A0DCF5	5888
A0CTC0	5888	Q6FT35	5478	A0K0R0	290399
A0DIQ3	5888	C5DTM8	559307	A0K4J4	331272
A0DRP7	5888	A7TL77	436907	A0LNB5	335543
A1CC50	5057	C5DD00	559295	A0LND6	335543
A1CEI6	5057	Q750J1	33169	A0LZA8	411154
A1CQW6	5057	Q6CRJ9	28985	A0M5G0	411154
A1D3S9	331117	A5DKC9	4929	A0M685	411154
A1DFQ7	331117	C4YB08	306902	A0Q1V4	386415
A1DKN9	331117	C4YK87	5476	A0Q832	401614
A2CI50	3144	Q5A2L3	5476	A0R3E3	246196
A2Q7C0	425011	Q5A2S3	5476	A0RBU7	412694
A2QPI1	425011	A3LVW5	4924	A0RR57	360106
A2QWB4	425011	B8YJM3	4924	A0RU01	46770
A2XPT6	39946	Q6BUJ8	4959	A0YQX1	313612
A2YB83	39946	C5M741	294747	A0YYR7	313612
A2YXH5	39946	B9WC72	573826	A0ZB83	313624
A2Z2Z0	39946	A5DX46	36914	A0ZZA6	367928
A3CJH1	39947	C4R0A2	644223	A1ABU2	405955
A3GEX9	4924	D1ZFZ8	5147	A1APQ5	338966
A3LYM2	4924	P00365	5141	A1AUR9	338966
A4H9P1	5660	B2WN29	426418	A1B8U4	318586
A4HEH1	5660	Q0UEU1	13684	A1BCV7	290317
A4HY08	5671	B6Q390	441960	A1HLZ4	401526
A4I1L9	5671	B6Q389	441960	A1HSF6	401526
A4R8U2	148305	B2AW77	5145	A1KVB4	272831
A4RNF5	148305	B8LT73	441959	A1R9V9	290340
A4RRY3	436017	D5GF65	39416	A1RT74	384616
A4RYZ2	436017	C5PHJ9	222929	A1RVI3	384616
A5A6L0	9598	A4QSW7	148305	A1RYG4	368408
A5ACP6	29760	Q9C2U8	5127	A1SH86	196162
A5B2Z7	29760	B0XVW9	451804	A1TEH7	350058
A5DKG2	4929	Q4WHB4	5085	A1TIL2	397945
A5DM34	4929	A1DG18	331117	A1TXD9	351348
A5E4A4	36914	B6HS95	500485	A1V0F0	320388
A5E7Q7	36914	A1C5Y4	5057	A1VGK8	391774
A5JTL8	7091	Q6XNK7	162425	A1VM77	365044
A5K3U7	5855	Q5AW79	162425	A1W2B5	232721
A5K4Q5	5855	C8VBC6	227321	A1W4P2	232721
A5YVE9	9606	Q0CHP4	341663	A1WIK0	391735
A6QVF3	339724	Q2UMK8	5062	A1WT43	349124
A6RDB1	339724	Q6C6H1	4952	A1ZDU1	313606
A6RQX9	332648	C5GF51	559297	A1ZG68	313606
A6S8P7	332648	C1GR57	502779	A1ZR86	313606
A6ZLG0	307796	C1G989	502780	A2BL51	415426
A6ZRD5	307796	C4JF29	336963	A2CA49	59922

A7EWB7	665079	A2QFH7	425011	A2D8B4	5722
A7F173	665079	D4DHP3	663202	A2DWI3	5722
A7MB35	9913	D4AL96	663331	A2G1Y8	5722
A7S6S9	45351	C0S0Y3	482561	A2S5H7	412022
A7SRY1	45351	C5FNT6	554155	A2SMA9	420662
A7TJM1	436907	C5JT14	559298	A2SRC2	410358
A7TM51	436907	A6R1Z2	339724	A2TQI4	313590
A7UUV1	7165	C0NCJ6	447093	A2TSV1	313590
A8IWK9	3055	Q4P1M9	5270	A2TYE4	313598
A8IXJ9	3711	B0CUZ3	486041	A2U018	313598
A8JBC6	3055	B0DSY6	486041	A2VSW9	350702
A8JBC7	3055	Q9Y8G5	5341	A2WCN3	350701
A8JCF6	3055	B8NPE3	332952	A2X7V1	39946
A8NXQ0	240176	A8NSP9	240176	A2XMV1	39946
A8P325	240176	Q5KJ92	5207	A2XW22	39946
A8P370	6279	Q9USN5	4896	A3ANK4	39947
A8P5C2	6279	A8PWR2	425265	A3CKW9	388919
A8PHS0	6279	D3BT84	670386	A3DCD3	203119
A8PHS1	6279	Q54VI3	44689	A3DLU5	399550
A8PIV1	6279	Q2H9P5	38033	A3HU52	388413
A8Q0F0	425265	A6SRU5	332648	A3HW69	388413
A8Q2M5	425265	D0NHJ6	403677	A3IB39	388400
A8XZA8	6238	A9URB8	81824	A3IFY6	388400
A9NWC1	3332	B9PR57	5811	A3IL52	391612
A9NWJ6	3332	B9QD05	432359	A3J255	391598
A9NWM3	3332	B6KHF0	508771	A3J2Y4	391598
A9NWY7	3332	C5LBR2	423536	A3J3F7	391598
A9P841	3694	C5L8Y0	423536	A3JK00	270374
A9P9K0	3694	C5L543	423536	A3JWL9	388401
A9PF50	3694	A7APP0	5865	A3K3C4	388399
A9S485	145481	A7E3Y0	665079	A3M3G4	400667
A9S8R2	145481	Q38EX6	5691	A3M9D7	400667
A9SPL8	145481	O99877	5691	A3MPG2	320389
A9SXT8	145481	C9ZXU0	679716	A3MUY9	410359
A9T5Q6	145481	Q25415	5689	A3MWK6	410359
A9TBP7	145481	Q4QF83	5664	A3MZE5	416269
A9TC13	145481	A4H874	5660	A3NDI7	320373
A9TTX3	145481	Q4N166	5875	A3NZ98	357348
A9TY50	145481	Q4U8H1	5874	A3PLD8	349101
A9V0I3	81824	A4HWJ9	5671	A3RRM9	342110
A9VB15	81824	C8WZJ8	485915	A3S765	52598
B0CYG4	486041	B8DRK8	883	A3SMT7	89187
B0DCT7	486041	B7FWY4	556484	A3T1P0	314267
B0W2T1	7176	B8BVC6	35128	A3U211	252305
B0WB92	7176	Q72DG5	882	A3U5S5	216432
B0X5L5	7176	C5U0R2	573059	A3U848	216432

B0XA87	7176	Q312G5	207559	A3V3Q4	314232
B0XPR8	451804	D1U2S7	643562	A3VPB7	314260
B0XVD8	451804	C4MA71	5759	A3W2R2	314264
B0XZB4	451804	A1VF24	391774	A3X055	314253
B1X3R0	39717	D0LMG4	502025	A3X6R2	314262
B1X423	39717	B3KZC7	5851	A3X960	314262
B2ATG7	5145	D2L065	644968	A3XI35	398720
B2AXC0	5145	D6YSD5	716544	A3XQE9	398720
B2R5P7	9606	B0ENR3	370354	A3ZUB7	314230
B2WEK4	426418	Q7RIC4	73239	A4A3G4	314285
B2WLB2	426418	Q4YNP5	5821	A4AE18	314285
B3L549	5851	Q6MB29	264201	A4APW2	313603
B3L9V4	5851	A5KCJ5	5855	A4ATX0	313603
B3LMU1	285006	D1R8E4	159254	A4BV92	314278
B3LRT8	285006	C0QAW6	177437	A4BYJ2	313594
B3MM14	7217	C0QBC7	177437	A4CJJ4	313596
B3MT41	7217	C0QD21	177437	A4CMC0	313596
B3MZ63	7217	C0QGJ6	177437	A4ENJ3	391593
B3MZ64	7217	A5GDM2	351605	A4EU82	388739
B3NU60	7220	A5GB85	351605	A4FGG9	405948
B3NU61	7220	C6MU48	443143	A4FGH8	405948
B3P5L5	7220	C6MRG2	443143	A4FM84	405948
B3RI95	10228	Q74CV7	35554	A4GJ67	415439
B3RY46	10228	D7AIZ4	663917	A4HGZ4	5660
B4DDD7	9606	B5EHY2	404380	A4I426	5671
B4E2N3	9606	C6E4K3	443144	A4IQB6	420246
B4F8B8	4577	B3E403	398767	A4IT39	420246
B4FGJ4	4577	B9M6R2	316067	A4IXJ1	418136
B4FML9	4577	Q39UW5	269799	A4JBJ3	269482
B4G2H5	7234	Q11CT2	266779	A4KPQ5	412422
B4G974	7234	A0B191	331272	A4MF39	425067
B4GV47	7234	A0KKL0	380703	A4MXA4	374927
B4GV48	7234	A0KW49	94122	A4N597	375432
B4HZ58	7238	A0L7M2	156889	A4NC90	375177
B4I0V9	7238	A0LVR1	351607	A4NHF6	374928
B4I0W0	7238	A0QDD7	243243	A4NM81	374932
B4J5T9	7222	A0R1C2	246196	A4NTY9	374933
B4JLC4	7222	A0RBU7	412694	A4NYZ3	375063
B4JRR3	7222	A1KLH2	410289	A4QFE5	340322
B4K754	7230	A1KUS5	272831	A4QHY0	340322
B4KK39	7230	A1RJ05	351745	A4T6P7	350054
B4L471	7230	A1S5Z3	326297	A4VI30	379731
B4L472	7230	A1SG04	196162	A4W9I4	399742
B4LTB1	7244	A1ST78	357804	A4WM05	340102
B4M0B1	7244	A1U206	351348	A4WMJ4	340102
B4M3I9	7244	A1UJ73	189918	A4WQS9	349102

B4M3J0	7244	A1VP09	365044	A4YIG2	399549
B4MWW8	7260	A3MUY9	410359	A4YQZ0	114615
B4NBF7	7260	A3Q2L9	164757	A5BSM5	29760
B4NEE4	7260	A3QDX7	323850	A5EFM4	288000
B4NEE5	7260	A4F9C3	405948	A5FBS3	376686
B4PQ74	7245	A4FH38	405948	A5FMS4	376686
B4Q027	7245	A4IQB6	420246	A5G5A5	351605
B4Q028	7245	A4JPF2	269482	A5G5J9	351605
B4QZG5	7240	A4SME1	382245	A5I2T3	441771
B4R4P7	7240	A4T7V6	350054	A5IFK8	400673
B4R4P8	7240	A4VM44	379731	A5ING2	390874
B5DIC8	46245	A4XAT7	369723	A5IRD6	359786
B5G1H2	59729	A4XWG7	399739	A5J911	334802
B5LAW2	4072	A4Y7I3	319224	A5K0U3	5855
B5LAW3	4072	A5FYF5	349163	A5K164	5855
B5VEH3	545124	A5I2T3	441771	A5KL31	411460
B5VHW0	545124	A5IC79	400673	A5LDP8	406556
B5X4R5	8030	A5IRD6	359786	A5LN10	406557
B6GZG1	500485	A5U5H2	419947	A5LVW0	406558
B6H5P2	500485	A5VF76	392499	A5M484	406559
B6HQM6	500485	A5VSF9	444178	A5M7R4	406560
B6JZG7	402676	A5W6M4	351746	A5MGJ7	406561
B6K381	402676	A5WEF7	349106	A5MRJ7	406562
B6KDD9	508771	A5WQ88	336982	A5MSW6	406563
B6QAQ6	441960	A6QFG8	426430	A5N1R2	431943
B6QI45	441960	A6TLH2	293826	A5P6Y6	161528
B6QTP8	441960	A6U065	359787	A5UAW4	374930
B6T565	4577	A6UED1	366394	A5ULL5	420247
B6T6H3	4577	A6V310	381754	A5UU09	357808
B6TC14	4577	A6VUB2	400668	A5V1G5	357808
B6TD83	4577	A6WEM1	266940	A5V3C0	392499
B6TD94	4577	A6WP31	402882	A5VGL3	392499
B6TKX6	4577	A6WXV0	439375	A5VNF6	444178
B6TMS5	4577	A7FUM1	441770	A5XV02	334803
B6TP75	4577	A7GE56	441772	A5Z9F7	411463
B6TQ36	4577	A7GN28	315749	A5ZR80	411459
B6UI91	4577	A7IF87	78245	A6BKL3	411462
B7E707	39947	A7N0L5	338187	A6C0C8	344747
B7EWY7	39947	A7X0H1	418127	A6CBA5	344747
B7FJJ4	3880	A7Z646	326423	A6CG24	344747
B7FZN6	556484	A7ZA02	326423	A6CK18	161544
B7GAB0	556484	A8EZV8	293613	A6CP62	161544
B7PR86	6945	A8F2P6	416276	A6CR94	161544
B7PZE3	6945	A8FEN2	315750	A6DKS2	313628
B7Z3T7	9606	A8FW62	425104	A6DTG1	313628
B7Z3X5	9606	A8GPN7	293614	A6DYL7	391613

B7ZWU6	4577	A8GTM0	392021	A6E717	391596
B8ANM1	39946	A8GUP5	391896	A6EMP5	50743
B8B945	39946	A8H3X7	398579	A6EN51	50743
B8BN11	39946	A8HYW7	438753	A6FQ52	351016
B8BVN4	35128	A8M2G7	391037	A6H0W7	402612
B8LC08	296543	A8MJK9	350688	A6LIH9	435591
B8LPU2	3332	A8Z064	451516	A6LJS6	391009
B8LRC3	3332	A9AQQ3	395019	A6LKL9	391009
B8M064	441959	A9CHD5	176299	A6NVM8	411467
B8MLB2	441959	A9IZE8	382640	A6QBL6	387093
B8MNW2	441959	A9KFS5	434922	A6QFG8	426430
B8N4B6	332952	A9L4D1	399599	A6SUM1	375286
B8N7H4	332952	A9M0J4	374833	A6SXZ7	375286
B8NL85	332952	A9M857	483179	A6T7S2	272620
B9FSH9	39947	A9NDT8	360115	A6T8K3	272620
B9GMQ4	3694	A9VLQ8	315730	A6TLH2	293826
B9GRR1	3694	A9WMN1	288705	A6U065	359787
B9HLV5	3694	A9WWU4	470137	A6VLA6	339671
B9HU92	3694	B0BV56	452659	A6WVM7	439375
B9IJS2	3694	B0K183	399726	A6X7S8	439375
B9PIC5	5811	B0K9J6	340099	A7A2X9	411481
B9Q6G0	432359	B0KG75	76869	A7AJB2	411477
B9RFW4	3988	B0RRS4	509169	A7AS73	5865
B9RNK3	3988	B0S1R7	334413	A7AYC2	411470
B9S0Z5	3988	B0T423	366602	A7B911	411466
B9S2H9	3988	B0TIY1	458817	A7FUM1	441770
B9TI82	3988	B0U746	405440	A7GE56	441772
B9WF66	573826	B0UCK2	426117	A7GN28	315749
B9WG75	573826	B1HXF2	444177	A7GWG7	360105
C0HAN7	8030	B1IM79	498213	A7HC09	404589
C0L943	65672	B1J592	390235	A7HEQ1	404589
C0NY78	447093	B1K2B6	406425	A7HLX9	381764
C0NZ02	447093	B1KDM7	392500	A7HS59	402881
C0P3K5	4577	B1KSB4	498214	A7I0A6	360107
C0S739	482561	B1M669	426355	A7JB60	430557
C0S8J0	482561	B1MMT4	561007	A7JJE2	430558
C0Z306	3702	B1VUY9	455632	A7JNJ5	442346
C1BKT8	8014	B1Y879	395495	A7JY81	150340
C1BUV4	72036	B1YI38	262543	A7N9W0	458234
C1BUZ3	72036	B1YXD5	398577	A7NH98	383372
C1BXA8	8010	B2A3W6	457570	A7NKS4	383372
C1C0S3	344056	B2FLA4	522373	A7S6F3	45351
C1C2R8	344056	B2GGY9	378753	A7SE06	45351
C1EJE1	296587	B2HNI6	216594	A7VHP8	411489
C1FHD4	296587	B2IAI7	405441	A7VIA6	411489
C1G056	502780	B2JR70	391038	A7VQT4	428125

C1G547	502780	B2S7P3	430066	A7X0H1	418127
C1GSN9	502779	B2SW49	360094	A7YRY8	430556
C1HC04	502779	B2TGT9	398527	A7YVW3	3625
C1MUI7	564608	B2UK21	402626	A7YVW4	3625
C1N531	564608	B3CPG6	570417	A7Z646	326423
C3ZAR7	7739	B3CVC9	334380	A7ZA02	326423
C3ZNR2	7739	B3D838	395019	A7ZB01	360104
C4JGN6	336963	B3PIM1	498211	A7ZMN0	331111
C4JQ30	336963	B3PS34	491916	A8A0V4	331112
C4PXN7	6183	B3R4P0	164546	A8AHF5	290338
C4QIQ4	6183	B4ELY2	216591	A8BFF8	184922
C4QYX8	644223	B4RCX5	450851	A8EIB4	360118
C4R290	644223	B4RKS4	521006	A8F3U9	416591
C4WXZ1	7029	B4RZ46	314275	A8FEN2	315750
C4Y374	306902	B4SPB0	391008	A8FIU2	315750
C4Y4H6	306902	B5FE16	388396	A8GEN4	399741
C5DN45	559295	B5Y6Y1	309798	A8GKZ5	399741
C5DQ72	559307	B5ZV29	395492	A8JGD1	3055
C5DSP9	559307	B6IUZ5	414684	A8KUC1	331978
C5E2N9	559295	B6IZQ2	434923	A8LCL1	298653
C5FGS2	554155	B6J7N6	434924	A8M7B0	391037
C5FRR0	554155	B7GHM2	491915	A8MAL7	397948
C5GNW4	559297	B7HHP4	405532	A8MJK9	350688
C5GYE7	559297	B7HKZ9	405534	A8NRG3	6279
C5JVW9	559298	B7IP68	405531	A8RDE0	428127
C5K304	559298	B7JGX6	405535	A8RJF0	411902
C5MDI0	294747	B7UVX9	557722	A8RQ20	411902
C5MI45	294747	B7VNK8	575788	A8RVA7	411902
C5P3C0	222929	B8CPG1	225849	A8SQ78	411474
C5P3J7	222929	B8E983	407976	A8U8B8	333990
C5WR68	4558	B8GXH7	565050	A8UDU7	391603
C5X5A2	4558	B8HBK7	452863	A8X0P1	6238
C5XZ73	4558	B8ICZ7	460265	A8YF61	267872
C5YBS3	4558	B8ZRA5	561304	A8Z064	451516
C5YSC6	4558	B9DIU1	396513	A9A0F1	96561
C5Z7K8	4558	B9EAN0	458233	A9A2G9	436308
C6H5C5	544712	B9IVL3	361100	A9AH92	395019
C6T827	3847	B9JEU7	311403	A9AZT9	316274
C6T902	3847	B9JV35	311402	A9BGN9	403833
C6TDD9	3847	B9KIA8	320483	A9BJ14	403833
C6TGZ8	3847	B9L9E1	598659	A9BN74	398578
C6TL67	3847	C0R5K1	66084	A9BPJ0	398578
C6TMA9	3847	C0RF69	546272	A9DMC0	391587
C7GMT2	574961	C0Z4V2	358681	A9EC10	391624
C7GS31	574961	C0ZCA2	358681	A9F2A8	448385
C7YYZ0	660122	C1A1Q4	234621	A9F5T9	383629

C7ZKY6	660122	C1AEU3	561275	A9GCU4	391619
C8VF33	227321	C1AVS4	632772	A9GCX2	448385
C8Z7K2	643680	C1D8T1	557598	A9GGT2	448385
C9SBS2	526221	C1DDX7	322710	A9HFA8	391595
C9SUX1	526221	C1EMY8	572264	A9I0G0	340100
C9ZK62	679716	C1FNV0	536232	A9INH5	340100
D0A589	679716	C3KD82	216595	A9KKP2	357809
D0MZU0	403677	C3KX46	515621	A9LB64	412021
D0NYZ7	403677	C3L9G7	568206	A9M1S9	374833
D1ZML0	5147	C3LMH5	579112	A9M7R4	483179
D2D323	3635	C3MAR3	394	A9MVX6	272994
D2VQJ9	5762	C3NQI3	593588	A9N282	272994
D3AX60	670386	C3P579	592021	A9NH73	441768
D3BCH5	670386	C3PLN7	347255	A9NXM5	3332
D3BML8	670386	C4K1F8	562019	A9SZX5	145481
D3DM87	559292	C4L6L5	360911	A9V780	81824
D3PGB6	72036	C4XRI8	573370	A9VLQ8	315730
D3PI89	72036	C5BKZ4	377629	A9WCC4	324602
D3PJ65	72036	C5C3D2	471853	A9WEM0	324602
D3TMY3	37546	C5D3H7	471223	B0A793	445973
D3TPF3	37546	C5D7D8	471223	B0A794	445973
D3UEW6	643680	C6AAW5	634504	B0AM54	486624
D4A5G8	10116	C6B069	395491	B0BTT0	434271
D4AIR7	663331	C6BLR2	428406	B0CFT6	329726
D4DFT9	663202	C6CU94	324057	B0CJ82	470137
D5G7G7	39416	C6DN06	478434	B0G3F6	411461
D5GGK4	39416	C6S7U3	662598	B0JQI9	449447
D6VQL7	559292	C6V502	434131	B0K183	399726
D6WH06	7070	C6WQ28	446462	B0K9J6	340099
D6WHR4	7070	C6XFI2	537021	B0MHT7	411490
D6X401	7070	C6XNG9	582402	B0N555	445974
D7FIP4	2880	C7LE50	568815	B0NA80	411468
D7KEQ8	81972	C7M2J7	525909	B0NCN8	411468
D7KMA1	81972	C7MZW4	471857	B0P2A6	411484
D7KPH3	81972	C7NDS3	523794	B0PDC5	445972
D7KQB5	81972	C7NFJ5	478801	B0Q2D0	486619
D7KXE8	81972	C7QFE1	479433	B0R2S8	478009
D7KXX5	81972	C7R6H0	523791	B0R3T5	478009
D7L1E7	81972	C7RFH9	525919	B0R537	478009
D7LH23	81972	C7RQT3	522306	B0TX73	484022
D7MQB7	81972	C8WW69	521098	B0UW88	228400
O13366	28985	C8XGM6	479431	B0V7W9	509173
O13392	4924	C9RV07	544556	B0V9Q6	509173
O16144	6253	C9S041	544556	B0VJV7	456827
O24457	3702	C9XIV3	645462	B0VQ84	509170
O24458	3702	C9YHY9	645463	B0VSA6	509170

O44451	6239	C9YZL4	680198	B0WNE2	7176
O64688	3702	D0B9Y5	224914	B0X5C8	7176
O82450	3702	D0K3M6	681288	B1B9U1	445337
O96865	5693	D0LAG6	526226	B1C1G6	428126
P08559	9606	D0M259	150340	B1C5T8	445971
P11177	9606	D1AEZ6	471852	B1ENR6	502347
P11966	9913	D1ASA8	574556	B1EYP0	486623
P16387	4932	D1YWP3	304371	B1FPM6	396596
P26267	6253	D2BET0	479432	B1G7C9	396598
P26268	6253	D2NQG0	680646	B1GFG5	486620
P26269	6253	D2PMN3	479435	B1HV12	444177
P26284	10116	D2RM10	591001	B1HXF2	444177
P29803	9606	D2S5H1	526225	B1ICB8	487214
P29804	9823	D2UDX7	380358	B1IM79	498213
P32473	4932	D3FPS8	398511	B1IPG9	481805
P35486	10090	D3G018	398511	B1JVT3	406425
P35487	10090	D3HTD9	661367	B1KSB4	498214
P49432	10116	D3P1Y0	137722	B1L7M0	374847
P51266	2787	D3P9I2	639282	B1L891	126740
P51267	2787	D3PZE9	446470	B1LDW9	439855
P52899	6239	D3QE37	698737	B1MGD0	561007
P52900	9302	D3T8X3	580331	B1N211	320374
P52901	3702	D4H5Z4	522772	B1QDG7	445335
P52902	3888	D4HEB1	553199	B1QQR1	445336
P52903	4113	D4YZH1	452662	B1S3K0	453362
P52904	3888	D4ZJH9	637905	B1S8L5	473819
Q016W5	70448	D5BVG7	472759	B1SCE7	471872
Q01MR6	4530	D5DFY2	592022	B1TC47	396597
Q06437	10116	D5DL42	592022	B1UMZ3	486622
Q09171	4896	D5DRI5	545693	B1VG71	504474
Q0CJX0	341663	D5DSM6	545693	B1VMK1	455632
Q0CPX2	341663	D5TDI0	423212	B1W2R9	455632
Q0CRE7	341663	D5TUX8	714359	B1WT18	43989
Q0J0H4	39947	D5UWZ1	521096	B1XGM2	316385
Q0U5F0	13684	D5V4J4	572480	B1XI27	32049
Q0UIH2	13684	D5VDR1	509190	B1XXB2	395495
Q10489	4896	D5WIV3	640511	B1Y209	395495
Q10G38	39947	D5WQR8	562970	B1Y8Z9	444157
Q10G39	39947	D5WSM1	562970	B1YI38	262543
Q16IL3	7159	D6XVC0	439292	B1YTC1	398577
Q17AH1	7159	D6Y6E1	469371	B1ZWE8	452637
Q17AH2	7159	D6Z8R5	640132	B2A3W6	457570
Q17D51	7159	D7A5Y6	639283	B2AH46	164546
Q1ACL0	55564	D7ANZ2	583358	B2DRM3	453363
Q1EGE2	70075	D7B5A2	446468	B2DTN0	453364
Q1EGE3	70075	D7CRN5	649638	B2DY74	453365

Q1EGE4	70075	D7CYM9	691437	B2E593	453366
Q1EGE5	291624	D7D4S4	691437	B2GH78	378753
Q1EGH7	70075	O53203	1773	B2H0H3	331109
Q1EGI1	70075	P39633	1423	B2HVT4	405416
Q1EGI2	291624	P50735	1423	B2HZS7	405416
Q1HPJ1	7091	Q02PN0	208963	B2IEJ1	395963
Q1KSF0	5811	Q0A4W8	187272	B2IQA4	516950
Q1LVS0	7955	Q0AKI2	394221	B2J2W4	63737
Q1XDM0	2788	Q0B893	339670	B2JGW2	391038
Q1XDM1	2788	Q0BXL2	228405	B2KCG1	445932
Q231N2	312017	Q0HJL1	60480	B2N2M1	344610
Q23JH9	312017	Q0KBX7	381666	B2NS03	444451
Q23KL2	312017	Q0RUA8	326424	B2P625	444452
Q27YD8	4922	Q0VP55	393595	B2PNK5	444453
Q28G62	8364	Q128V8	296591	B2Q5T9	471874
Q29AC1	46245	Q12NG3	318161	B2RKJ1	431947
Q29IF6	46245	Q13LC7	266265	B2S8W5	430066
Q29IF7	46245	Q15UB8	342610	B2SDV3	441952
Q2H5C3	38033	Q18CS0	272563	B2SYL9	398527
Q2H6J1	38033	Q1AT32	266117	B2TLD1	508765
Q2HTC6	3880	Q1B5W3	164756	B2U3E5	344609
Q2KH68	242507	Q1BP79	331271	B2UFJ1	402626
Q2QM55	39947	Q1ICM8	384676	B2UP90	349741
Q2T9Y3	9913	Q1MA37	216596	B2UUI0	512562
Q2U738	5062	Q1QAX4	335284	B2V1W6	508767
Q2UBL6	5062	Q21JX1	203122	B2VEU7	338565
Q2ULS2	5062	Q21YB1	338969	B3AA50	478004
Q32RM2	35869	Q2FZU5	93061	B3ATA1	478005
Q32RS0	102822	Q2GBW4	279238	B3B351	478006
Q38799	3702	Q2K2T8	347834	B3BJS9	478008
Q388X3	5691	Q2P230	342109	B3BY31	478007
Q3B8A4	8355	Q2RN38	269796	B3DP13	205913
Q3HVN3	4113	Q2SD03	349521	B3DXA7	481448
Q3UFJ3	10090	Q2WBE0	342108	B3E388	398767
Q4DB91	5693	Q2YLG9	359391	B3EEP5	290315
Q4DXQ0	5693	Q2YWS8	273036	B3ESG9	452471
Q4FZZ4	10116	Q397W0	269483	B3EUE2	452471
Q4P1A8	5270	Q3BS58	316273	B3H0U1	537457
Q4P7Q9	5270	Q3IGZ0	326442	B3HJ16	340184
Q4Q9R8	5664	Q3ILZ7	348780	B3HR05	340197
Q4QDQ1	5664	Q3J6X6	323261	B3IB67	340185
Q4SFM3	99883	Q3YSF5	269484	B3IJU9	340186
Q4WEY1	5085	Q472M5	264198	B3JAM7	405536
Q4WHM5	5085	Q47M08	269800	B3KT18	9606
Q4WJ75	5085	Q480K5	167879	B3KV55	9606
Q4Y6X7	5825	Q48FX7	264730	B3LA84	5851

Q4YXR1	5821	Q49V91	342451	B3LAJ5	5851
Q54C70	44689	Q49W97	342451	B3LV21	7217
Q55M90	5207	Q4KD35	220664	B3M317	7217
Q57ZD2	5691	Q4L4X4	279808	B3P7D7	7220
Q5A0Z9	5476	Q4UVV9	314565	B3P8B7	7220
Q5A5V6	5476	Q4ZVQ0	205918	B3PIL2	498211
Q5AQM7	162425	Q57B70	235	B3QL55	517417
Q5B2R8	162425	Q5E5B7	312309	B3QU23	517418
Q5BKI5	8364	Q5HBK7	254945	B3RBG0	164546
Q5BSR1	6182	Q5HHC7	93062	B3SB32	10228
Q5DFX8	6182	Q5HQK3	176279	B3T4F8	455574
Q5DLW4	70075	Q5KXR6	1462	B3TAG7	455603
Q5ECP6	4102	Q5L3K6	1462	B3TCA2	455612
Q5F426	9031	Q5P3E4	76114	B3W6Y4	543734
Q5JPU0	9606	Q5QX66	135577	B3WNY1	344601
Q5JPU3	9606	Q5WGV8	66692	B3X2B4	358708
Q5K8I1	5207	Q5WWK9	297245	B3XG27	358709
Q5KES4	5207	Q5X4Y1	297246	B3XZH7	86029
Q5R490	9601	Q5ZV59	272624	B3XZH8	86029
Q5R755	9601	Q607S1	414	B3YG31	439842
Q5RE79	9601	Q60A41	414	B3YKI7	439842
Q5VGY3	5833	Q63DP1	288681	B3YRI6	405917
Q5VGY4	5833	Q65I01	279010	B3Z7M7	451707
Q5XGY5	8355	Q67Q62	2734	B3ZU13	451709
Q654V6	39947	Q6FYF6	803	B3ZZ86	454168
Q66JA7	8355	Q6G5B9	38323	B4A127	454168
Q6B8T1	285951	Q6GAW8	282459	B4AJQ9	536229
Q6B8T2	285951	Q6GID0	282458	B4AKG2	536229
Q6BI75	4959	Q6LRA3	74109	B4APU8	545422
Q6BUP4	4959	Q6MPX2	959	B4B6W9	497965
Q6C0Y7	4952	Q73B03	222523	B4BL27	495036
Q6C4G4	4952	Q7A1B9	196620	B4BQY6	495036
Q6CKM3	28985	Q7A6H8	158879	B4D5I8	497964
Q6DEB0	8355	Q7D728	1773	B4DGN5	9606
Q6DGZ9	7955	Q7MKX5	196600	B4DMF5	9606
Q6FKF1	5478	Q7NTH1	536	B4DMG8	9606
Q6FMM4	5478	Q7TYH9	1765	B4E0N9	9606
Q6GR17	8355	Q81FS8	226900	B4EEU7	216591
Q6KCM1	3039	Q81SY3	1392	B4F018	529507
Q6NX32	8364	Q82DA7	33903	B4F9J5	4577
Q6P948	7955	Q83CA2	777	B4FFP2	4577
Q6Z1G7	39947	Q87DA1	183190	B4G3Y5	7234
Q6Z5N4	39947	Q87PB5	670	B4G4C1	7234
Q6ZYP7	5061	Q88L55	160488	B4HFD8	7238
Q74ZU5	33169	Q895H9	1513	B4HG10	7238
Q75EB0	33169	Q89C03	375	B4JRH4	7222

Q7K5K3	7227
Q7KVX1	7227
Q7Q2S3	7165
Q7R908	73239
Q7RRB2	73239
Q7RYJ2	5141
Q7T368	7955
Q7XTJ3	39947
Q7YU05	7227
Q852R9	161934
Q852S0	161934
Q85FX1	45157
Q85FX2	45157
Q86HX0	44689
Q8H1Y0	3702
Q8HXW9	9541
Q8I111	6239
Q8IGJ4	7227
Q8IIB8	36329
Q8IL09	36329
Q8LAI3	3702
Q8MA03	96477
Q8SQM8	6035
Q8SS26	6035
Q95VS5	278021
Q9C6Z3	3702
Q9D051	10090
Q9D9X9	10090
Q9LDY2	3702
Q9MAM6	3702
Q9MUR4	41882
Q9P3R3	5141
Q9SAV3	3702
Q9TLS2	2771
Q9TLS3	2771
Q9W4H4	7227
Q9W4H6	7227
Q9ZQY0	4577
Q9ZQY1	4577
Q9ZQY2	4577
Q9ZQY3	4577
Q8CJY0	1902
Q8CPU5	176280
Q8D9G3	672
Q8D9G4	672
Q8EDZ7	70863
Q8EQ98	182710
Q8FYP5	29461
Q8IAM0	36329
Q8RA84	119072
Q8RAK8	119072
Q8RG30	76856
Q92KX8	382
Q98ES5	381
Q99VD0	158878
Q9ABY2	155892
Q9HZE0	287
Q9K5Z3	86665
Q9K9D0	86665
Q9KCE9	86665
Q9KRZ1	666
Q9PBP9	2371
A0JYF8	290399
A0LNB5	335543
A0NWT6	384765
A0Y1E5	156578
A0YYR7	313612
A1APQ5	338966
A1EQD0	345076
A1F7S7	412614
A1HLZ4	401526
A1HSF6	401526
A1RT74	384616
A1RVI3	384616
A1TCE6	350058
A1UR01	360095
A1WVU5	349124
A2BL51	415426
A2PAL3	412966
A2PQR5	412883
A2V2U0	399804
A2VKJ9	348776
A2VY28	350702
A2WFG3	350701
A3D5C0	325240
A3EMV6	345075
A3GPF0	417399
B4JRQ0	7222
B4K740	7230
B4KAW1	7230
B4KAW2	7230
B4LXF2	7244
B4LXF3	7244
B4M0C5	7244
B4N918	7260
B4N9E8	7260
B4PL48	7245
B4PN68	7245
B4QSD4	7240
B4R1F7	7240
B4R8T2	450851
B4RGD7	450851
B4RN81	521006
B4RV54	314275
B4S8U0	290512
B4SUI7	423368
B4T3Y9	423368
B4TGD6	454169
B4TKC5	454169
B4TUB6	439843
B4TXW5	439843
B4U375	552526
B4UC71	447217
B4UL58	447217
B4V7F3	465541
B4VK35	118168
B4WTC6	91464
B4WX92	236097
B5BA76	554290
B5BI57	554290
B5BZB3	439846
B5C299	439846
B5CFX7	454165
B5CIL9	454165
B5CM20	471875
B5DPT4	46245
B5E539	512566
B5E987	404380
B5F4F2	454166
B5F852	454166
B5FJE2	439851
B5FTN9	439851
B5GKV5	465543

A3H2C3	417400	B5HT00	463191
A3IB39	388400	B5ICF2	439481
A3IL52	391612	B5IVF4	391623
A3JK00	270374	B5J9T0	391626
A3KWA1	350704	B5JE84	382464
A3LA48	350703	B5JF32	382464
A3MWK6	410359	B5K4W5	391616
A3RTJ2	342110	B5KBH2	391616
A3TQZ7	313589	B5L6W8	6689
A3UEH2	314254	B5MQ26	439847
A3UZH4	314291	B5MSW9	439847
A3WPC1	314276	B5MZL9	440534
A3Y3X2	314290	B5N2U8	440534
A3YHB0	314277	B5NA84	454167
A3ZUB7	314230	B5NAD5	454167
A4A3G4	314285	B5NU80	454231
A4BNN6	314278	B5NVR5	454231
A4BV92	314278	B5P0Q1	454164
A4CC90	87626	B5P7Y8	454164
A4KJM5	395095	B5PH00	465518
A4WM05	340102	B5PKB9	465518
A4WMJ4	340102	B5PP90	465516
A5CFM8	357244	B5PZA5	465516
A5F840	345073	B5QAA0	465517
A5G5J9	351605	B5QDE1	465517
A5IFK8	400673	B5QT82	1582
A5ING2	390874	B5QWJ4	550537
A5L4F1	391574	B5R2X5	550537
A5P981	161528	B5R905	550538
A5TX97	393480	B5S8H2	305
A5UU09	357808	B5SG61	564066
A5VNF6	444178	B5URX9	405533
A6A6L5	417398	B5W8D8	513049
A6B6T9	419109	B5WHU7	516466
A6CBA5	344747	B5X0T5	8030
A6CK18	161544	B5X1B8	8030
A6CP62	161544	B5XRH1	507522
A6CR94	161544	B5XS60	507522
A6D6G0	391591	B5Y6Y1	309798
A6EXA6	443152	B5YQ50	444450
A6F0Y7	443152	B5Z871	563041
A6LJS6	391009	B6AZK9	314270
A6LKL9	391009	B6BA15	439496
A6WVM7	439375	B6BG40	439496
A6XUY9	404974	B6BKM6	439483
A6Y606	345074	B6BYE6	473788

A7BKE8	422288	B6BZE1	473788
A7HLX9	381764	B6FJG4	500632
A7K626	150340	B6FVZ6	500633
A7NKS4	383372	B6GCU1	445975
A8F3U9	416591	B6IBI2	409438
A8MAL7	397948	B6JIA2	504832
A8PNX1	59196	B6JMR5	570508
A8T0Y8	314289	B6KQJ7	508771
A8TXS5	331869	B6WSB9	411464
A8YF61	267872	B6XAW7	520999
A9AZT9	316274	B6XSI8	566552
A9CUL4	411684	B6YSV5	523850
A9EJA0	314608	B7A1X9	502346
A9M7R4	483179	B7A595	498848
A9VKT4	315730	B7A596	498848
A9WCC4	324602	B7AWD8	483218
A9WEM0	324602	B7BDK4	537006
B0A7U3	445973	B7C7D7	518637
B0AM54	486624	B7CLI9	557724
B0CFT6	329726	B7DT50	543302
B0CJ82	470137	B7GHM2	491915
B0DLZ4	486041	B7GSG9	391904
B0JQI9	449447	B7GVU6	557600
B0K190	399726	B7GXT1	557600
B0Q2D0	486619	B7HHP4	405532
B0R2S8	478009	B7HKZ9	405534
B0R537	478009	B7I8U8	480119
B0UFW9	426117	B7IAQ4	480119
B1EYP0	486623	B7IG24	484019
B1FDF3	396596	B7IP68	405531
B1G0J4	396598	B7JGX6	405535
B1GFG5	486620	B7JYG4	41431
B1L891	126740	B7K9R8	65393
B1QDG7	445335	B7L300	440085
B1QQR1	445336	B7L6N4	585055
B1T670	396597	B7LQ31	585054
B1UMZ3	486622	B7M1H3	585034
B1WT18	43989	B7MAX1	585035
B1XI27	32049	B7MVN9	585397
B1Y8Z9	444157	B7N596	585056
B1ZWE8	452637	B7NT17	585057
B2IEJ1	395963	B7P4E1	6945
B2S8W5	430066	B7QWT2	439497
B3DXA7	481448	B7R2V8	246969
B3JAM7	405536	B7R7L2	391606
B3XZH7	86029	B7RCI9	443254

B3XZH8	86029	B7RH26	391589
B3YRI6	405917	B7STY6	4442
B3Z7M7	451707	B7USE2	574521
B3ZU13	451709	B7WR10	399795
B4AKG2	536229	B7X125	399795
B4B6W9	497965	B7ZZ39	4577
B4BL27	495036	B8A4B0	7955
B4D5I8	497964	B8D6P6	490899
B4RV54	314275	B8DA65	552536
B4VG18	465541	B8G0B1	272564
B4VK35	118168	B8G4T1	326427
B4W5N0	391600	B8G8N4	326427
B4WTC6	91464	B8GSN0	396588
B4X3K2	236097	B8H6K7	452863
B5GHN1	465543	B8HN81	395961
B5H187	443255	B8I5M4	394503
B5HD85	457429	B8IGL7	460265
B5I834	463191	B8IZX9	525146
B5IVF4	391623	B8J999	455488
B5JF32	382464	B8JGR7	455488
B5S4E1	305	B8KKZ0	566466
B5SCB2	305	B8KWA2	565045
B5URX9	405533	B8Y4H6	1894
B5WP29	516466	B8ZK25	561276
B6BG40	439496	B9AEN0	483214
B6C5Q5	473788	B9B972	513051
B6FVZ6	500633	B9BXG1	513052
B6R1Y8	439495	B9CJN7	513053
B6W713	561177	B9CKM5	553184
B6Y9H2	569881	B9CQM1	553212
B6YSV5	523850	B9CX16	591023
B7DT50	543302	B9DIU1	396513
B7DUC0	543302	B9DRY4	218495
B7IG24	484019	B9E5H1	583346
B7JYG4	41431	B9EAN0	458233
B7K9R8	65393	B9FGE7	39947
B7L300	440085	B9I5Y2	3694
B7R2V8	246969	B9ICE5	3694
B7R7L2	391606	B9IPQ2	3694
B7RCI9	443254	B9IVL3	361100
B8G4T1	326427	B9K9V6	309803
B8G8N4	326427	B9KDG9	306263
B8HN81	395961	B9KKE0	557760
B8IGL7	460265	B9KZK6	309801
B8KE79	391586	B9LED0	480224
B8L4T8	391601	B9LH26	480224

B9AZL8	513051	B9LN57	416348
B9BHG0	513052	B9LUS4	416348
B9C1G4	513053	B9LZJ9	316067
B9CQM1	553212	B9MAJ1	535289
B9K9V6	309803	B9MEN4	535289
B9KZK6	309801	B9MHY9	535289
B9LED0	480224	B9MUV2	3694
B9LH26	480224	B9NAP2	3694
B9LUS4	416348	B9NP16	467661
B9QVX9	244592	B9PY05	5811
B9WPP9	2252	B9QLJ6	432359
B9XLJ6	320771	B9RA12	3988
C0DS07	546274	B9SEE3	3988
C0EQX2	546264	B9SHF8	3988
C0G3Z3	595497	B9WPP9	2252
C0GCY7	555088	B9XLJ6	320771
C0GDU8	555088	B9XW63	544405
C0RGS5	546272	B9XZU7	544406
C0WE93	563191	C0ATZ8	471881
C1B758	632772	C0BB24	470146
C1BAD2	632772	C0BIH7	487796
C1PEX5	345219	C0BLL5	487797
C1V8W8	469382	C0BUC3	547043
C1V8W9	469382	C0BX65	553973
C2BEX3	525254	C0BZQ2	553973
C2CA41	592313	C0E4U7	566549
C2CJ21	525255	C0E9U2	537013
C2G976	548473	C0ENR3	546264
C2HRQ4	593585	C0ETW9	411469
C2I332	593586	C0FPZ9	622312
C2JIL3	593587	C0G3Z3	595497
C2LVV9	629742	C0GCY7	555088
C2MIF5	526973	C0GDU8	555088
C2MYI3	526980	C0HA51	8030
C2NFA2	526970	C0M727	553482
C2NWJ2	526967	C0MEK1	553483
C2PCK9	526971	C0Q339	476213
C2PT40	526972	C0Q6Y4	476213
C2PTM6	526972	C0QGH3	177437
C2Q9M6	526968	C0QWK1	565034
C2QQN5	526977	C0RGS5	546272
C2R5R6	526969	C0VJB9	525244
C2RKR4	526974	C0VLI3	525244
C2S1D5	526975	C0VV97	548477
C2SH92	526976	C0W243	525245
C2SHI1	526976	C0W5L5	525246

C2SYL9	526978	C0WKF5	525260
C2TE71	526979	C0X578	491074
C2TUJ7	526981	C0XIV4	525327
C2TUY9	526981	C0XS81	525263
C2UBI8	526982	C0Y6L1	595498
C2UT11	526983	C0YUW1	525257
C2V554	526983	C0Z4V2	358681
C2V919	526984	C0ZCA2	358681
C2V9F9	526984	C1A1R9	234621
C2VRC2	526985	C1A8S6	379066
C2W5M3	526986	C1ARQ2	632772
C2W609	526986	C1AV88	632772
C2WK38	526987	C1AVT4	632772
C2X518	526988	C1B589	632772
C2X9H1	526989	C1B5G6	632772
C2XRM5	526990	C1B6F7	632772
C2Y8C7	526991	C1C7T1	488221
C2YPB2	526992	C1CEQ8	488222
C2Z5J5	526993	C1CL37	488223
C2ZHT5	526993	C1CR03	487213
C2ZLS1	526994	C1CX96	546414
C2ZM61	526994	C1CX97	546414
C3A345	526997	C1D8T0	557598
C3A3A4	526997	C1DWD7	204536
C3AJ94	526998	C1EMY8	572264
C3AJL7	526998	C1F3U4	240015
C3B148	526999	C1FNV0	536232
C3B1F8	526999	C1HLD1	469598
C3BHS2	527000	C1HYU7	528345
C3BI67	527000	C1I8B3	457396
C3BZZ6	527024	C1M3R4	469595
C3CGF9	527021	C1M5E3	469595
C3CZB2	527025	C1PEX5	345219
C3DHI5	527026	C1V8W8	469382
C3E178	527027	C1V8W9	469382
C3EIH0	527023	C2BNQ5	525264
C3EZ98	527022	C2BYG3	525367
C3FHU1	527031	C2CNP2	525268
C3G0L7	527032	C2CYM6	525310
C3GGG3	527029	C2D848	525256
C3GYP2	527030	C2DD07	525278
C3HG40	527028	C2DQA1	525281
C3HY27	527019	C2EDD3	525362
C3IH83	527020	C2EJJ3	525364
C3IYR0	550542	C2FDD8	525337
C3J4A4	550542	C2FKR1	525338

C3JRR2	596309	C2FU68	525372
C3KKH2	394	C2FVZ9	525372
C3WHI8	469599	C2FZV1	525372
C3WRU1	469606	C2G976	548473
C3WWJ5	457405	C2GKR8	548478
C4IN97	641140	C2GY27	548480
C4IT22	641140	C2H567	525271
C4RQ40	219305	C2HC73	525279
C4W997	596319	C2JJR6	491075
C4WH45	641118	C2KTF9	585199
C4WJ25	641118	C2L0W2	585501
C4ZJN3	85643	C2LEJ3	525369
C5A1Q6	593117	C2LTS1	596322
C5N438	450394	C2LVV9	629742
C5NXG6	546270	C2M4R2	553178
C5PY83	548474	C2MIF5	526973
C5Q7N8	525374	C2MYI3	526980
C5QHR1	548475	C2NFA2	526970
C5QQR1	525378	C2NWJ2	526967
C5QVT1	525376	C2PCK9	526971
C5RT38	573062	C2PTM6	526972
C5RT45	573062	C2Q9M6	526968
C5SJK8	573065	C2QQN5	526977
C5TNM1	596320	C2R5R6	526969
C5U9S6	509193	C2RKR4	526974
C5UB71	509193	C2S1D5	526975
C6A3G9	604354	C2SHI1	526976
C6HJC6	544712	C2SYL9	526978
C6HJC8	544712	C2TE71	526979
C6IXD6	621372	C2TUJ7	526981
C6JLG8	469618	C2TUY9	526981
C6M3Y3	547045	C2UBI8	526982
C6MNF5	443143	C2UT11	526983
C6N0S9	658187	C2V554	526983
C6N5E8	658187	C2V919	526984
C6N7I2	658187	C2V9F9	526984
C6QPG8	581103	C2VRC2	526985
C6R2N3	553201	C2W5M3	526986
C6RX96	661513	C2W609	526986
C6S9S7	663926	C2WK38	526987
C6SNJ8	295996	C2X9H1	526989
C6YC53	345072	C2XRM5	526990
C7CFH7	661410	C2Y8C7	526991
C7HK66	588857	C2YPB2	526992
C7HK73	588857	C2Z5J5	526993
C7HVG3	655811	C2ZM61	526994

C7IT23	589861	C3A345	526997
C7IVB2	589861	C3AJ94	526998
C7LFT0	568815	C3AJL7	526998
C7P2C4	485914	C3B148	526999
C7P570	485914	C3B1F8	526999
C7QXG9	395962	C3BHS2	527000
C7R731	523791	C3BI67	527000
C7XMM1	469604	C3BZZ6	527024
C7ZVH1	585143	C3CGF9	527021
C8A309	585145	C3CZB2	527025
C8AAH8	585146	C3DHI5	527026
C8AI52	585153	C3E178	527027
C8AQJ4	585158	C3EIH0	527023
C8KLW3	452948	C3EZ98	527022
C8KUA8	455227	C3FHU1	527031
C8L6H9	553565	C3G0L7	527032
C8LCF4	553567	C3GGG3	527029
C8LM30	553568	C3GYP2	527030
C8LUV0	553571	C3HG40	527028
C8LWX5	553573	C3HY27	527019
C8M6F6	553581	C3IH83	527020
C8MG48	553583	C3IYR0	550542
C8MPL0	553588	C3J4A4	550542
C8MRD9	553592	C3JU89	596309
C8N4S8	553596	C3K2C9	216595
C8PS27	596324	C3KKH2	394
C8SWI0	536019	C3KX46	515621
C8VY59	485916	C3L9G7	568206
C8WXA1	521098	C3MNL1	429572
C9MYP5	634994	C3MVT6	427317
C9N8V6	591167	C3MX56	427317
C9NX03	675814	C3N3F5	427318
C9P4T8	675813	C3N4K0	427318
C9PDH6	675811	C3NBJ1	439386
C9Q6W9	675810	C3ND80	439386
C9QI10	675816	C3NIH5	419942
C9SP25	526221	C3NKV6	419942
C9T509	520459	C3P579	592021
C9T7Z1	520459	C3PH96	548476
C9TED0	520460	C3RKH6	556270
C9TGP3	520460	C3T742	562
C9TMR2	520463	C3WAP6	469616
C9TRC8	520463	C3X4W9	556268
C9TSR0	520461	C3XB31	556269
C9TT50	520461	C3XFD4	613026
C9U5T7	520454	C3XJS4	556267

C9U7G1	520454	C3XQA6	7739
C9UF10	520452	C4ANY3	320390
C9UGN5	520452	C4F1M2	521005
C9UP98	520451	C4F5W7	521004
C9UQX5	520451	C4F820	521003
C9UVW3	520450	C4FD46	518635
C9V082	520450	C4FN67	546273
C9V8A3	520456	C4G6Y9	592010
C9VDB8	520456	C4GAE3	626523
C9VL93	520457	C4GF98	629741
C9VMG9	520457	C4IN97	641140
C9VUW6	520455	C4KFD0	426118
C9VVX2	520455	C4KG51	426118
C9WYM9	604162	C4KV59	536230
D0AZH6	575591	C4L6L5	360911
D0B174	575591	C4LHR0	645127
D0B4S8	224914	C4QM82	6183
D0BDB8	520488	C4V2L2	638302
D0BJ17	520488	C4VE20	553209
D0BRP7	469603	C4W997	596319
D0GD78	520465	C4WH45	641118
D0GHI0	520465	C4X724	484021
D0GLV1	596323	C4X7R3	484021
D0GVJ2	675806	C4Y7X8	306902
D0H8D0	675807	C4Z318	515620
D0HGS6	675820	C4Z8N8	515619
D0HMR1	675808	C4ZIU4	85643
D0HWZ5	675809	C4ZM37	85643
D0IAI4	675812	C4ZZB7	595496
D0IET5	675815	C5A1Q5	593117
D0P8N9	520489	C5A1Q6	593117
D0PAQ5	520489	C5AB11	626418
D0PHZ3	520487	C5BB17	634503
D0PNE2	520487	C5BWF2	471853
D0RLL9	437701	C5C851	465515
D0W0I2	546262	C5C933	465515
D0WBV1	546265	C5CD78	521045
D0X0V8	674977	C5CDZ3	521045
D0XD73	673519	C5CN50	543728
D0XD74	673519	C5D3H7	471223
D0XIV4	633149	C5D7D8	471223
D1C4B5	479434	C5E973	537937
D1CFL6	525904	C5EXX2	537972
D1CVG1	520449	C5F9Y0	537973
D1CZI5	520449	C5N438	450394
D1EM50	520462	C5N7H2	436115

D1EQ98	520462	C5PM11	525373
D1EZW6	520464	C5PTG7	525373
D1F1J5	520464	C5PV37	525373
D1F6I8	520466	C5PY83	548474
D1FAV2	520466	C5Q7N8	525374
D1FHQ5	520458	C5QHR1	548475
D1FK13	520458	C5QQR1	525378
D1GRD2	663951	C5QVT1	525376
D1JAA5	115547	C5RB90	585506
D1Q9J2	553594	C5RID0	573061
D1QGJ0	553601	C5RT38	573062
D1R0C0	553574	C5S0Z8	637911
D1RMG8	638315	C5TA65	573060
D1S597	644283	C5TB05	573060
D1UCM5	640510	C5TKE9	596320
D1VUR4	596330	C5U059	573059
D1WLB4	596317	C5U9S6	509193
D1WVF2	649189	C5UPY2	536233
D1XQ50	647653	C5VD14	553207
D1YBK5	679194	C5VK09	553174
D2C507	590168	C5VLX4	553174
D2F573	585149	C5VRL1	592027
D2FBQ2	585151	C5W4T4	511693
D2FKI1	585152	C5WG08	486410
D2FTY3	585159	C5YD28	4558
D2FZW5	585160	C5ZCD3	357347
D2G8B3	585161	C5ZXL8	537970
D2GES1	585148	C6A3G9	604354
D2GP28	585150	C6AJR8	634176
D2N5S0	523796	C6BC43	428406
D2R268	530564	C6BS98	526222
D2RXF9	543526	C6CCZ3	579405
D2RY65	543526	C6CU94	324057
D2RZ42	543526	C6CXF6	324057
D2RZC1	543526	C6DJB5	561230
D2RZC6	543526	C6DZH6	443144
D2S0I1	543526	C6E8X1	443144
D2UKS3	585155	C6ECJ6	469008
D2UQQ4	585147	C6IXQ9	621372
D2YH77	671074	C6J7W9	457412
D2YT37	671076	C6LMB6	478749
D2ZSM5	546266	C6M0A8	598745
D3A3B0	546268	C6MB43	153948
D3C3F6	648999	C6MNF5	443143
D3EV51	703339	C6N0S9	658187
D3MF09	679195	C6N5E8	658187

D3MF10	679195	C6P885	580327
D3MLG6	686659	C6PTN9	536227
D3MSI0	596329	C6QID7	582899
D3N116	640512	C6QPG8	581103
D3RWS3	589924	C6RAQ4	553206
D3SX02	547559	C6RHN9	553219
D3SX05	547559	C6RJT5	596318
D3T0V9	547559	C6RMH6	596318
D3T144	547559	C6RQU4	596318
D4CUY0	546275	C6S8E8	662598
D4FNV8	525375	C6SBQ2	663926
D4FY67	645657	C6SL48	295996
D4G2W2	645657	C6SQZ5	511691
D4GPR3	309800	C6TDZ0	3847
D4GY63	309800	C6TY86	320371
D4GY69	309800	C6UJ75	413997
D4SQM9	427081	C6UX17	544404
D4T216	427082	C6VNY5	644042
D4U8Z7	553590	C6VSW5	471854
D4UDF8	553580	C6VYT1	471854
D4Y2I2	634956	C6WZG7	531844
D5AY29	449216	C6XS85	485917
D5BVC3	472759	C6XXL3	485917
D5MHU6	671143	C6Y3X1	485917
D5N0E4	703612	C6YTX8	539329
D5N4X9	703612	C7BU49	553480
D5N7Z3	243261	C7BXF1	592205
D5PH40	525368	C7CFH7	661410
D5PPU3	525370	C7CT29	565636
D5PT98	525370	C7D3S6	565637
D5Q9B1	525259	C7DAG8	633131
D5QPZ9	595536	C7DHY8	425595
D5RE87	525283	C7G5J4	536231
D5S4M2	525258	C7GZY3	592031
D5SC00	762948	C7HHQ3	572545
D5VZM8	758678	C7HK66	588857
D5XVQ2	515617	C7IDC0	588581
D5Y565	520141	C7IT23	589861
D5YU92	515616	C7LFT0	568815
D5Z602	537209	C7LQK9	525897
D5ZH62	537210	C7MAV2	446465
D5ZZ71	566461	C7MNC8	469378
D6AHH9	457431	C7MXH8	471857
D6B3K5	457425	C7MZK2	471857
D6BDQ7	556264	C7N599	471855
D6EV17	457428	C7NKM4	478801

D6FJ37	611303	C7NSW5	519442
D6FYK6	611304	C7P2A4	485914
D6GU59	546269	C7P2C4	485914
D6GYE6	585156	C7P570	485914
D6HFF7	585144	C7PSY3	485918
D6IYV2	585157	C7QXG9	395962
D6K2E3	645465	C7R2K2	471856
D6LCM6	469602	C7R731	523791
D6LGV7	469621	C7U7K5	565638
D6LKG7	520448	C7UGI0	565642
D6LLF8	520448	C7UP10	565641
D6LW48	585154	C7UV78	565650
D6SB14	525282	C7V5G4	565640
D6SFN9	548470	C7VCG4	565644
D6T6S5	553577	C7VNK8	565646
D6UBX8	762962	C7VW65	565649
D7BVP8	749414	C7W2T1	565647
D7E021	551115	C7W9W7	565648
D7ETI9	515615	C7WKH6	565643
D7H1D6	520453	C7WNY5	565651
D7H499	520453	C7X246	565645
D7HJJ1	412967	C7X6E5	563193
D7I303	693985	C7YAS1	565639
D7N0C9	641149	C7ZVH1	585143
D7N8L1	575609	C8A309	585145
O52310	53953	C8AAH8	585146
O59650	311400	C8AI52	585153
O74024	49899	C8AQJ4	585158
P27346	1496	C8JVB1	393124
P28997	1258	C8K3J9	393125
P29051	2242	C8K7X8	393128
P54386	1148	C8KLW3	452948
P80319	2261	C8KUA8	455227
P96110	2336	C8L6H9	553565
Q02D11	234267	C8LCF4	553567
Q081J8	318167	C8LM30	553568
Q0E5I0	1570	C8LUV0	553571
Q0HVV7	60481	C8LWX5	553573
Q0SB12	101510	C8M6F6	553581
Q0SGX1	101510	C8MG48	553583
Q0W8B2	351160	C8MPL0	553588
Q0W8B3	351160	C8MRD9	553592
Q10WR8	203124	C8N4S8	553596
Q12UM4	259564	C8N8B6	638300
Q18J04	362976	C8NNN6	196164
Q1GQV5	117207	C8NPT9	196164

Q1LP59	266264	C8NTT8	585529
Q1N646	207949	C8QEF4	592316
Q1NA38	314266	C8RTU9	525262
Q1QXW3	290398	C8S361	371731
Q1RKJ5	336407	C8T607	667127
Q1V5E2	314288	C8T6I9	667127
Q1Z9A6	314280	C8TSS7	573235
Q1ZD05	314282	C8UA24	585395
Q1ZD06	314282	C8UBL5	585396
Q1ZQY0	314292	C8VY59	485916
Q2B639	313627	C8WAA2	521095
Q2BHA6	207954	C8WI25	479437
Q2BMD5	207954	C8WW69	521098
Q2BXZ9	121723	C9A1D2	565653
Q2GDP8	222891	C9ACQ0	565655
Q2GG64	205920	C9ADJ6	565662
Q2GKF6	212042	C9AMB6	565663
Q2N6W6	314225	C9B0M9	565652
Q2YP74	359391	C9B4P5	565658
Q31EJ5	317025	C9BEH6	565659
Q3A571	338963	C9BQJ0	565657
Q3EN96	339854	C9BZD6	565661
Q3IS94	348780	C9C7M6	565660
Q3ISK4	348780	C9CAF1	565656
Q3KBB0	205922	C9CNZ8	565654
Q3M5M1	240292	C9CVM8	644076
Q3R4W8	155920	C9KN37	500635
Q3RAJ7	155920	C9LB68	537007
Q3RC72	155919	C9LQX0	592028
Q47950	29292	C9LXX3	546271
Q47951	39456	C9M5D0	645512
Q4C653	165597	C9MCJ1	656913
Q4ED18	307502	C9MIH4	656912
Q4FSU4	259536	C9MRD2	649761
Q4MGB5	269801	C9NDK4	591167
Q4UK71	42862	C9P7J4	675813
Q56304	2265	C9PLW8	675811
Q57FF3	235	C9PN71	667128
Q5FHJ1	302409	C9QTU7	536056
Q5GTA1	292805	C9R6N5	668336
Q5PB16	234826	C9RNS6	59374
Q5V3Y8	2238	C9RV07	544556
Q5V4X6	2238	C9S041	544556
Q5V6I7	2238	C9T509	520459
Q5X2P3	297246	C9TED0	520460
Q5Z090	37329	C9TMR2	520463

Q5Z0H0	37329
Q64BT4	285389
Q64CH4	286719
Q64CM6	286721
Q67KK8	2734
Q68VZ1	785
Q6A837	1747
Q6ANZ7	84980
Q6KZF2	263820
Q73IE5	163164
Q73XL5	1770
Q7P4E5	209882
Q7P9S4	272951
Q8G2T7	29461
Q8PZY2	2209
Q8TL71	2214
Q8YF04	29459
Q8YJ55	29459
Q8ZT48	13773
Q8ZW33	13773
Q92GE9	781
Q977U6	2252
Q977X9	90909
Q97AN9	273116
Q9F0J1	1901
Q9HK32	2303
Q9HQE1	2242
Q9HRM7	2242
Q9HSM4	2242
Q9NGT0	5833
Q9X7B2	1769
Q9Y8I4	2277
Q9ZCI2	782

C9TT50	520461
C9U7G1	520454
C9UGN5	520452
C9UQX5	520451
C9UVW3	520450
C9V8A3	520456
C9VL93	520457
C9VVX2	520455
C9WY24	604162
C9X5X2	568708
C9XFX9	568708
C9XIV3	645462
C9YGH6	667019
C9YHY9	645463
C9Z6P4	680198
D0ADQ1	565666
D0ALR7	565664
D0AZH6	575591
D0B4S8	224914
D0BDB8	520488
D0BYB4	575564
D0C0M9	575564
D0C906	575584
D0CCU3	575584
D0CSZ8	644107
D0CZL4	501479
D0FSJ4	79967
D0GHI0	520465
D0GUP7	675806
D0HHK9	675820
D0ITD0	290847
D0IVD0	688245
D0J1V2	688245
D0J9U8	600809
D0JAG4	331104
D0K0F2	684950
D0K3M6	681288
D0KE64	561231
D0KPB0	555311
D0KPS0	555311
D0KV60	555311
D0L7K3	526226
D0LGK4	502025
D0LHW9	502025
D0LJJ2	502025
D0MD96	518766

D0PAQ5	520489
D0PNE2	520487
D0RLL9	437701
D0RSL1	469609
D0S072	575585
D0S496	575585
D0S8G5	575586
D0SAL3	575586
D0SJC0	575587
D0SKD0	575587
D0SMD0	575587
D0SUU2	575588
D0SWA0	575588
D0T4P8	575589
D0T4U3	575589
D0T5I3	575589
D0TCW4	469589
D0W527	546262
D0WEM6	649764
D0WLT4	649743
D0WS85	674977
D0YPN7	596328
D0YSR2	596328
D0ZGL3	498217
D0ZJ71	588858
D0ZW30	588858
D1A2H6	471852
D1AKZ0	526218
D1B531	525898
D1B825	525903
D1B8F3	525903
D1BC07	446469
D1BEM5	446469
D1BPB9	479436
D1BVJ5	446471
D1BX67	446471
D1C4B5	479434
D1C557	479434
D1CFL6	525904
D1CVG1	520449
D1D6W1	528346
D1DA26	528352
D1DGC3	528354
D1DQN2	528356
D1DXB4	528355
D1E3Z9	528358

D1EA59	528359
D1EGY9	528360
D1EQ98	520462
D1F1J5	520464
D1F6I8	520466
D1FHQ5	520458
D1GRD2	663951
D1JAA5	115547
D1NLN9	492476
D1NWC2	561180
D1P0C7	500637
D1PC66	537011
D1PIU8	411471
D1PUP4	585502
D1PVJ6	585502
D1Q9J2	553594
D1QGJ0	553601
D1QQK2	649760
D1R0C0	553574
D1RRM8	682634
D1RYM0	682634
D1SQ63	643561
D1UHF4	640510
D1VNC1	298654
D1VUR4	596330
D1VYL3	679189
D1W6L1	679190
D1WLB4	596317
D1WTX5	649189
D1WUS2	649189
D1XQ85	647653
D1Y2A2	352165
D1YDA3	679194
D1YQ21	686660
D1YWP3	304371
D2AFF8	591020
D2ALP0	510831
D2AZR2	479432
D2BFC1	479432
D2C507	590168
D2EQG4	563037
D2F573	585149
D2FBQ2	585151
D2FKI1	585152
D2FTY3	585159
D2FZW5	585160

D2G8B3	585161
D2GES1	585148
D2GP28	585150
D2LZH8	649639
D2MN04	679192
D2N5S0	523796
D2NJ38	431946
D2NY56	653938
D2PA11	637381
D2PHJ8	425944
D2PI86	425944
D2Q6I5	401473
D2QS31	504472
D2R268	530564
D2RXF9	543526
D2RY65	543526
D2RZ42	543526
D2RZC1	543526
D2RZC6	543526
D2S0I1	543526
D2SGC4	526225
D2TB98	644651
D2TUK6	637910
D2U087	638
D2UKS3	585155
D2UQQ4	585147
D2V2V3	5762
D2V6F5	5762
D2YJ06	671074
D2YQC3	671076
D2Z542	469381
D2ZB99	500639
D2ZPY7	521002
D2ZZV4	546266
D3AI09	566550
D3C045	653733
D3D3X2	102897
D3DZ61	634498
D3EV51	703339
D3F4P9	469383
D3F9G9	469383
D3FPS8	398511
D3FUG4	398511
D3FWN6	398511
D3G018	398511
D3GVH4	216592

D3H869	365659
D3HE25	637909
D3I165	575611
D3I787	575612
D3I8X6	575612
D3IAQ1	575614
D3KL75	393121
D3L5E6	592015
D3LG75	565665
D3LKL2	596312
D3LS85	596312
D3MGM7	679195
D3MP04	686659
D3MSI0	596329
D3N3Y9	640512
D3PM55	504728
D3PM56	504728
D3QE37	698737
D3QUV4	701177
D3RAM2	640131
D3RH42	640131
D3RJ65	640131
D3RWS3	589924
D3SX02	547559
D3SX05	547559
D3T0V9	547559
D3T144	547559
D3T8X3	580331
D3TA98	439481
D3TQR2	37546
D3UGI7	679897
D3USF6	683837
D3UY64	406818
D3VF16	406817
D4B8M6	500640
D4BAI2	500640
D4BLG5	518634
D4C241	521000
D4CDT0	411486
D4CKH1	608534
D4DN64	546263
D4DYU0	667129
D4ED00	694569
D4EPZ4	699185
D4ERV7	699186
D4FNV8	525375

D4FTM1	655813
D4FY67	645657
D4G2W2	645657
D4GEM7	706191
D4GPR3	309800
D4GY63	309800
D4GY69	309800
D4H2E9	522772
D4HFC6	553199
D4I2J9	665029
D4ICQ6	716540
D4IQD3	717959
D4J2B6	657324
D4J437	717962
D4JCV2	717960
D4JI76	657317
D4KF87	657316
D4KKW8	657315
D4L424	718255
D4L843	657321
D4LFA8	213810
D4LPM4	657323
D4LSA1	657314
D4M5L5	657313
D4M9E2	651822
D4MXD7	245018
D4N6X9	684057
D4N759	684059
D4PIU4	393117
D4PR78	393131
D4Q3R2	401650
D4QHV0	529885
D4QQR5	535205
D4R228	535206
D4R6G3	544875
D4RLZ6	546340
D4RMW1	547468
D4S0B6	511680
D4S8A3	585503
D4SAK9	585531
D4SLZ9	645663
D4TWN4	649742
D4U8Z7	553590
D4UDF8	553580
D4UMW4	246199
D4UN78	246199

D4UYQ9	791166
D4VVZ9	791161
D4XC87	742159
D4XNM2	707232
D4XP09	707232
D4XRH8	707232
D4Y2I2	634956
D4YH29	655812
D4YQB5	585530
D4ZR61	696747
D5BBT4	655815
D5BCK8	655815
D5BM99	655815
D5BQY2	488538
D5BVC3	472759
D5BY05	472759
D5CCV4	716541
D5D411	714962
D5DDK5	592022
D5DFY2	592022
D5DL42	592022
D5DRI5	545693
D5DSM6	545693
D5E439	545693
D5ED85	572547
D5EJE7	583355
D5EUQ2	264731
D5HBJ8	761659
D5HDH9	751585
D5MHU6	671143
D5N0E4	703612
D5N4X9	703612
D5NRK3	243261
D5NU55	649754
D5PPW1	525370
D5Q9B1	525259
D5QPZ9	595536
D5R8K4	642492
D5S4M2	525258
D5S619	760570
D5TUX8	714359
D5U2J2	633148
D5U7Q3	526224
D5UHH3	446466
D5VAL1	749219
D5VZM8	758678

D5WD52	640511
D5WQR8	562970
D5WSM1	562970
D5WYN1	75379
D6A4N4	566461
D6A5F7	566461
D6AI30	457431
D6B9J6	457425
D6CN89	426114
D6DAY9	722911
D6DGX1	717608
D6DTW7	718254
D6E321	657318
D6E6Z1	657308
D6EE73	457428
D6GDX8	469608
D6GN49	469608
D6GYE6	585156
D6H6N1	528348
D6HFF7	585144
D6HL81	552396
D6HXP2	550672
D6IB74	550676
D6IQP0	656380
D6IYV2	585157
D6JA48	550677
D6JKW7	528351
D6JRN2	575565
D6JX36	575565
D6KBG9	645465
D6KJY8	457416
D6KQF5	450749
D6KTP9	641146
D6L4N0	641144
D6LLF8	520448
D6LW48	585154
D6SFN9	548470
D6T6S5	553577
D6THA7	485913
D6TL40	485913
D6UBX8	762962
D6UKB7	585535
D6V8X3	666684
D6VEJ3	596153
D6VIC2	596153
D6WCS6	7070

D6X9Z0	457429
D6XQU5	637913
D6XUQ4	439292
D6XVC0	439292
D6XX88	439292
D6Y2I7	469371
D6YQU5	712898
D6Z5V7	589865
D6ZKH1	548479
D6ZS37	525381
D6ZX62	759350
D7AI85	663917
D7ANZ2	583358
D7BF42	526227
D7BF43	526227
D7C6S8	749414
D7C9D5	749414
D7CNN7	643648
D7CYM9	691437
D7D4S4	691437
D7DB91	591019
D7E021	551115
D7EIW1	7070
D7FCS9	693745
D7GFF9	754252
D7H1D6	520453
D7IN64	469592
D7JR10	656379
D7L1B5	81972
D7LXS2	81972
D7M0I9	81972
D7N3R1	641149
D7NA33	563008
O02375	6289
O04937	4092
O52310	53953
O59650	311400
O60995	5693
O60996	5693
O61083	5693
O74024	49899
O87403	1402
O96940	5833
P00366	9913
P00367	9606
P00368	9031

P0C934	837
P10860	10116
P14657	2725
P26443	10090
P27346	1496
P28724	5741
P29051	2242
P31026	1718
P39633	1423
P43793	727
P49448	9606
P50735	1423
P52596	29760
P54385	7227
P54386	1148
P55990	210
P80053	2287
P80319	2261
P82264	36190
P93541	4081
P96110	2336
Q01X95	234267
Q02D11	234267
Q03M22	322159
Q04K22	373153
Q095H5	378806
Q095S1	378806
Q0A6S2	187272
Q0AIP4	335283
Q0AUZ3	335541
Q0BIA1	339670
Q0BNP9	393011
Q0E5I0	1570
Q0FF08	367336
Q0FNM4	314265
Q0I4P4	205914
Q0IG14	7159
Q0JBB6	39947
Q0JZU7	381666
Q0KEF0	381666
Q0KHB4	6689
Q0RB72	326424
Q0RY06	101510
Q0SBJ5	101510
Q0SC90	101510
Q0SGW0	101510

Q0SJ78	101510
Q0SJW1	101510
Q0T4W7	373384
Q0TH69	362663
Q0USN5	13684
Q0VMQ0	393595
Q0W8B2	351160
Q0W8B3	351160
Q10WR8	203124
Q127Y9	296591
Q12UM4	259564
Q13UA2	266265
Q14J69	393115
Q15Z70	342610
Q169D8	375451
Q16SW1	7159
Q17YH3	382638
Q18CS0	272563
Q18J04	362976
Q1BZ56	331271
Q1CSJ3	357544
Q1D013	246197
Q1D4C2	246197
Q1GEN4	292414
Q1GPE6	117207
Q1H5A3	3702
Q1HDV6	29760
Q1IJ35	204669
Q1IT55	204669
Q1J137	319795
Q1J138	319795
Q1JT17	383379
Q1K016	281689
Q1KYN6	222440
Q1LD03	266264
Q1LRE2	266264
Q1NJF0	262489
Q1NW86	262489
Q1PVP6	174633
Q1QJW6	323097
Q1QPK1	323097
Q1RB31	364106
Q1VBD1	314288
Q1VPY8	313595
Q1WSM5	362948
Q23621	6239

Q23ZD8	312017
Q24BW7	312017
Q24BX6	312017
Q24MK0	138119
Q26B44	156586
Q26BC3	156586
Q28LQ5	290400
Q298M7	46245
Q29A69	46245
Q2A5D8	376619
Q2B639	313627
Q2CDB1	314256
Q2F5R4	7091
Q2FZU5	93061
Q2G8V7	279238
Q2IDY1	290397
Q2IIQ6	290397
Q2J4Q6	106370
Q2KQ98	7159
Q2KU68	360910
Q2L0R9	360910
Q2LQG2	56780
Q2LW88	56780
Q2NEG6	339860
Q2NWM5	343509
Q2S0C1	309807
Q2S582	309807
Q2SZ78	271848
Q2YP74	359391
Q2YWS8	273036
Q30QE4	326298
Q31EJ5	317025
Q321Q3	300268
Q32GA3	300267
Q33E23	39947
Q38946	3702
Q39JL1	269483
Q39WF1	269799
Q3A571	338963
Q3D0Y7	342615
Q3D6F6	342616
Q3DFQ8	342617
Q3DJH6	342614
Q3DQR9	342613
Q3EN96	339854
Q3IL85	326442

Q3ILZ7	348780
Q3IS94	348780
Q3ISK4	348780
Q3J0W2	272943
Q3J9I2	323261
Q3JCR9	323261
Q3JNP7	320372
Q3M5M1	240292
Q3MDV8	240292
Q3SQA1	323098
Q3XZ31	333849
Q3Z2A7	300269
Q43260	4577
Q43314	3702
Q467V9	269797
Q46UV2	264198
Q475U5	264198
Q47950	29292
Q47951	39456
Q47CI0	159087
Q47Q60	269800
Q49V91	342451
Q49W97	342451
Q4C653	165597
Q4D5C2	5693
Q4DWV8	5693
Q4EID3	267410
Q4EVW3	267409
Q4FLE4	198252
Q4JCA3	2285
Q4JV01	306537
Q4K5S2	220664
Q4L4X4	279808
Q4MGB5	269801
Q4Q7X1	5664
Q4QP01	281310
Q4S2P6	99883
Q4X2W2	5825
Q53199	394
Q54KB7	44689
Q56304	2265
Q57FF3	235
Q57NL7	28901
Q57PY4	28901
Q5F2M8	3847
Q5F2M9	3847

Q5F731	242231
Q5HHC7	93062
Q5HQK3	176279
Q5KTR6	151740
Q5KXR6	1462
Q5L3K6	1462
Q5LSM4	89184
Q5M148	299768
Q5M5N6	264199
Q5MBG2	2242
Q5NHR7	119856
Q5P0F8	76114
Q5QDM6	3873
Q5QVZ2	135577
Q5SI03	300852
Q5SI04	300852
Q5V3Y8	2238
Q5V4X6	2238
Q5V6I7	2238
Q5WCI5	66692
Q5WGV8	66692
Q5WMA2	146919
Q5X2P3	297246
Q5XGI4	8364
Q5YQU8	37329
Q60A41	414
Q62H34	13373
Q63DP1	288681
Q63QU9	28450
Q64BT4	285389
Q64CH4	286719
Q64CM6	286721
Q64HZ8	9598
Q64HZ9	9580
Q64I00	9600
Q64I01	9595
Q65GI2	279010
Q65I01	279010
Q65W57	221988
Q67KK8	2734
Q6A7K5	1747
Q6AJB1	84980
Q6ANZ7	84980
Q6AZJ0	8355
Q6DB50	29471
Q6F931	62977

Q6FD67	62977
Q6GAW8	282459
Q6GID0	282458
Q6H3Y7	39947
Q6J667	158899
Q6KZF2	263820
Q6MPX2	959
Q6NZ29	7955
Q6P3L9	7955
Q6SI43	257382
Q722Z0	265669
Q72F40	882
Q72IC0	262724
Q72IC1	262724
Q73B03	222523
Q73P03	158
Q74DL1	35554
Q74MJ2	228908
Q7A1B9	196620
Q7A6H8	158879
Q7M823	844
Q7NA25	141679
Q7NLJ0	33072
Q7PHE4	7165
Q7RGT5	73239
Q7RQ39	73239
Q7TPI5	10116
Q7UPH7	265606
Q7V7V9	74547
Q7VGS9	32025
Q7VSN6	520
Q7VXC5	520
Q7WA25	519
Q7WEU7	518
Q7WJ54	518
Q7XN06	39947
Q7YWC6	6832
Q7ZZK8	8030
Q7ZZK9	8030
Q7ZZL0	8030
Q803T3	7955
Q81FS8	226900
Q81SY3	1392
Q82MM4	33903
Q82U80	915
Q835G2	1351

Q83RH0	623
Q852M0	39947
Q88XM9	1590
Q8CPU5	176280
Q8DPG0	171101
Q8DUL2	1309
Q8EQ98	182710
Q8FGY8	217992
Q8FTJ1	152794
Q8G2T7	29461
Q8G6L0	216816
Q8GR85	274
Q8GR86	274
Q8ILF7	36329
Q8ILT0	36329
Q8IMY1	7227
Q8JHX9	8022
Q8JHY1	8022
Q8KAX7	1097
Q8L6A0	4097
Q8PRZ1	2209
Q8PZY2	2209
Q8RA84	119072
Q8RC70	119072
Q8RQP4	152794
Q8T453	7227
Q8TL71	2214
Q8W1X4	4081
Q8Y256	305
Q8Y9G8	1639
Q8YF04	29459
Q8YPE2	103690
Q8ZT48	13773
Q8ZW33	13773
Q92E91	1642
Q930S3	382
Q93IM2	274
Q94AA6	3702
Q94F14	3702
Q94IA5	3708
Q94IH7	3120
Q94IH8	3120
Q96YC6	111955
Q977U6	2252
Q977X9	90909
Q97AN9	273116

Q97AP0	273116
Q97L29	1488
Q97QB4	1313
Q97WS2	2287
Q97X22	2287
Q97Y81	2287
Q99VD0	158878
Q9AGH6	28451
Q9BSD0	9606
Q9CPJ4	747
Q9FES0	29760
Q9GTK5	5833
Q9HK32	2303
Q9HKG4	2303
Q9HQE1	2242
Q9HRM7	2242
Q9HSM4	2242
Q9K5Z3	86665
Q9K9D0	86665
Q9KB34	86665
Q9KCE9	86665
Q9KEM8	86665
Q9L0F9	1902
Q9LEC8	4092
Q9RTN9	1299
Q9RVP5	1299
Q9S7A0	3702
Q9TVN3	47911
Q9TXS8	5833
Q9VCN3	7227
Q9Y8I4	2277
Q9YC65	56636
Q9ZKD8	85963
Q9ZRZ0	4686

GDH1/3

UniProt	TXID
A0AG12	386043
A0AQX1	1599
A0K0R0	290399
A0KIR1	380703
A0M685	411154
A0Q1V4	386415
A0Q832	401614
A0R3E3	246196
A0RR57	360106
A0YQX1	313612
A0YYR7	313612
A0ZB83	313624
A0ZZA6	367928

A1ABU2	405955
A1APQ5	338966
A1AUR9	338966
A1B8U4	318586
A1CGX0	5057
A1CXW1	331117
A1JSP8	393305
A1KVB4	272831
A1R9V9	290340
A1RT74	384616
A1RVI3	384616
A1SH86	196162
A1TEH7	350058
A1TXD9	351348
A1UL36	189918
A1W4P2	232721
A1WT43	349124
A1ZR86	313606
A2BL51	415426
A2CA49	59922
A2D8B4	5722
A2DWI3	5722
A2FCY9	5722
A2G1Y8	5722
A2QHT6	425011
A2TF52	1307
A2TF53	1307
A2TQI4	313590
A2TYE4	313598
A3CKW9	388919
A3DCD3	203119
A3IFY6	388400
A3IL52	391612
A3J2Y4	391598
A3J3F7	391598
A3JK00	270374
A3L3S9	350704
A3LKH9	350703
A3LS93	4924
A3M3G4	400667
A3MUY9	410359
A3MWK6	410359
A3MZE5	416269
A3Q5I4	164757
A3U5S5	216432
A3WHR0	237727

A3WLP4	314276
A3XQE9	398720
A3ZUB7	314230
A4A3G4	314285
A4AE18	314285
A4APW2	313603
A4BI48	314283
A4BV92	314278
A4BX97	313594
A4CIM6	313596
A4E704	411903
A4ENJ3	391593
A4FM84	405948
A4G269	204773
A4HGZ4	5660
A4I426	5671
A4IT39	420246
A4IXJ1	418136
A4KPQ5	412422
A4MXA4	374927
A4N597	375432
A4NC90	375177
A4NHF6	374928
A4NM81	374932
A4NTY9	374933
A4NYZ3	375063
A4QFE5	340322
A4RGW8	148305
A4SPC8	382245
A4T6P7	350054
A4TQX9	386656
A4VI30	379731
A4VSY1	391295
A4VZ67	391296
A4W9I4	399742
A4WM05	340102
A4WMJ4	340102
A4XT69	399739
A4ZYU8	562
A5APE2	29760
A5D6B0	370438
A5DKU4	4929
A5E4Q2	36914
A5FM33	376686
A5G5A5	351605
A5G5J9	351605

A5IFK8	400673
A5ING2	390874
A5K0U3	5855
A5K164	5855
A5KL31	411460
A5LDP8	406556
A5LN10	406557
A5LVW0	406558
A5M484	406559
A5M7R4	406560
A5MGJ7	406561
A5MRJ7	406562
A5MSW6	406563
A5N1R2	431943
A5P6Y6	161528
A5UAW4	374930
A5ULL5	420247
A5UU09	357808
A5V3C0	392499
A5V5X9	392499
A5VGL3	392499
A5VNF6	444178
A5VYB0	351746
A5WEK2	349106
A5Z9F7	411463
A5ZLC7	411901
A5ZLD5	411901
A5ZR80	411459
A6BKL3	411462
A6BWR4	412420
A6CBA5	344747
A6DKS2	313628
A6EQC4	50743
A6GWB2	402612
A6KX04	435590
A6KX09	435590
A6LIH4	435591
A6LIH9	435591
A6LJS6	391009
A6NVM8	411467
A6QBL6	387093
A6R7P3	339724
A6SLQ7	332648
A6SXZ7	375286
A6T7S2	272620
A6USH6	406327

A6VBX5	381754
A6VLA6	339671
A6VWJ0	400668
A6WVM7	439375
A6ZPK3	307796
A7A0A7	307796
A7A2X9	411481
A7AJ97	411477
A7AJB2	411477
A7AS73	5865
A7AYC2	411470
A7B911	411466
A7EUW2	665079
A7FP04	349747
A7GWG7	360105
A7HEQ1	404589
A7HLX9	381764
A7I0A6	360107
A7JB60	430557
A7JJE2	430558
A7JNJ5	442346
A7JY81	150340
A7LQM4	411476
A7LSB5	411476
A7N9W0	458234
A7NKS4	383372
A7RFX2	45351
A7SE06	45351
A7TKG3	436907
A7V1Y1	411479
A7V203	411479
A7VHP8	411489
A7VQT4	428125
A7YRY8	430556
A7ZB01	360104
A7ZMN0	331111
A8A0V4	331112
A8AHF5	290338
A8BFF8	184922
A8ERI8	367737
A8F3U9	416591
A8FIU2	315750
A8GKZ5	399741
A8GZW8	398579
A8LTK4	398580
A8MAL7	397948

A8P5F5	240176
A8RDE0	428127
A8RQ20	411902
A8S385	411902
A8SAT6	411485
A8SQ78	411474
A8U8B8	333990
A8UIQ6	391603
A8YF61	267872
A9A0F1	96561
A9AZT9	316274
A9BGN9	403833
A9BJ14	403833
A9BN74	398578
A9DQI4	391587
A9GCX2	448385
A9H304	391595
A9I0G0	340100
A9KKP2	357809
A9M1S9	374833
A9M7R4	483179
A9N282	272994
A9NH73	441768
A9R5J0	349746
A9RG07	145481
A9RTS6	145481
A9RXU2	145481
A9WCC4	324602
A9WEM0	324602
A9Z5Q1	373665
B0A1H9	404214
B0A793	445973
B0A794	445973
B0BTT0	434271
B0CFT6	329726
B0CJ82	470137
B0G3F6	411461
B0GH05	404218
B0GU84	404217
B0HAC4	404216
B0HKL2	404215
B0HTX0	360099
B0JQI9	449447
B0KME1	76869
B0MHT7	411490
B0MRS7	428128

B0MVZ4	445970
B0N555	445974
B0NCN8	411468
B0NU92	449673
B0NUB9	449673
B0P2A6	411484
B0PDC5	445972
B0R2S8	478009
B0R537	478009
B0RZ86	59201
B0TS30	458817
B0TX73	484022
B0UW88	228400
B0V9Q6	509173
B0VJV7	456827
B0VSA6	509170
B0Y5K4	451804
B1B9U1	445337
B1BJJ0	445334
B1BWI0	451755
B1C1G6	428126
B1C5T8	445971
B1ENR6	502347
B1HV12	444177
B1ICB8	487214
B1IPG9	481805
B1JF25	390235
B1JHU9	502800
B1L891	126740
B1LDW9	439855
B1MGD0	561007
B1QXD1	447214
B1R556	451754
B1RGN1	451756
B1RPY4	451757
B1S3K0	453362
B1S8L5	473819
B1SCE7	471872
B1V119	488537
B1VG71	504474
B1W2R9	455632
B1WT18	43989
B1XGM2	316385
B1XI27	32049
B1Y8Z9	444157
B1ZVK9	452637

B1ZWE8	452637
B2B5X0	5145
B2DRM3	453363
B2DTN0	453364
B2DY74	453365
B2E593	453366
B2EBY1	486409
B2GH78	378753
B2HVT4	405416
B2IEJ1	395963
B2IQA4	516950
B2J2W4	63737
B2K6K4	502801
B2KCG1	445932
B2N2M1	344610
B2NS03	444451
B2P625	444452
B2PNK5	444453
B2Q5S7	471874
B2Q5T9	471874
B2RKJ1	431947
B2S8W5	430066
B2SDV3	441952
B2TLD1	508765
B2U3E5	344609
B2UP90	349741
B2UUI0	512562
B2V1W6	508767
B2W0U7	426418
B3AA50	478004
B3ATA1	478005
B3B351	478006
B3BJS9	478008
B3BY31	478007
B3CIV7	471870
B3CIY0	471870
B3DP13	205913
B3DXA7	481448
B3E388	398767
B3H0U1	537457
B3HJ16	340184
B3HR05	340197
B3IB67	340185
B3IJU9	340186
B3JKL2	470145
B3JKM7	470145

B3LA84	5851
B3LAJ5	5851
B3LK29	285006
B3LUZ4	285006
B3PIL2	498211
B3RBG0	164546
B3W6Y4	543734
B3WNY1	344601
B3X2B4	358708
B3XG27	358709
B3YKI7	439842
B4A127	454168
B4AJQ9	536229
B4APU8	545422
B4B6W9	497965
B4BQY6	495036
B4D5I8	497964
B4F018	529507
B4R8T2	450851
B4RN81	521006
B4RV54	314275
B4RXA2	314275
B4S8U0	290512
B4T3Y9	423368
B4TGD6	454169
B4TUB6	439843
B4U375	552526
B4UL58	447217
B4V7F3	465541
B4VK35	118168
B4WTC6	91464
B4WX92	236097
B5ATN7	1307
B5BA76	554290
B5BZB3	439846
B5CIL9	454165
B5CM20	471875
B5CYJ2	484018
B5CYL1	484018
B5E539	512566
B5E987	404380
B5F852	454166
B5FJE2	439851
B5GKV5	465543
B5ICF2	439481
B5JE84	382464

B5JF32	382464
B5MQ26	439847
B5MZL9	440534
B5NAD5	454167
B5NU80	454231
B5P7Y8	454164
B5PKB9	465518
B5PZA5	465516
B5QDE1	465517
B5QJG0	486408
B5QT82	1582
B5QWJ4	550537
B5VDG0	545124
B5W8D8	513049
B5XS60	507522
B5YQ50	444450
B5Z871	563041
B6BG40	439496
B6BKM6	439483
B6BZE1	473788
B6FJG4	500632
B6GCU1	445975
B6HSV9	500485
B6IBI2	409438
B6JIA2	504832
B6JMR5	570508
B6K3S5	402676
B6KQJ7	508771
B6Q267	441960
B6V7E4	5061
B6VS76	483217
B6VSG6	483217
B6WSB9	411464
B6XAW7	520999
B6XSI8	566552
B7A1X9	502346
B7AD89	483216
B7ADB1	483216
B7AWD8	483218
B7BDK4	537006
B7BDL4	537006
B7C7D7	518637
B7G3X3	556484
B7GSG9	391904
B7GXT1	557600
B7I8U8	480119

B7IG24	484019
B7JYG4	41431
B7K9R8	65393
B7L300	440085
B7L6N4	585055
B7LQ31	585054
B7M1H3	585034
B7MAX1	585035
B7MVN9	585397
B7N596	585056
B7NT17	585057
B7RCI9	443254
B7U3Y2	1307
B7USE2	574521
B7V0D1	557722
B7WR10	399795
B8AA37	39946
B8DA65	552536
B8DV15	442563
B8G0B1	272564
B8G4T1	326427
B8G8N4	326427
B8H6K7	452863
B8HN81	395961
B8I5M4	394503
B8IGL7	460265
B8IZX9	525146
B8JGR7	455488
B8KKZ0	566466
B8KWA2	565045
B8LUC1	441959
B8NA36	332952
B8Y4H6	1894
B8ZK25	561276
B9AEN0	483214
B9CKM5	553184
B9CX16	591023
B9DRY4	218495
B9E5H1	583346
B9K9V6	309803
B9KDG9	306263
B9KZK6	309801
B9LED0	480224
B9LH26	480224
B9LUS4	416348
B9LZJ9	316067

B9MEN4	535289
B9MHY9	535289
B9N1A0	3694
B9PY05	5811
B9QLJ6	432359
B9QUN2	244592
B9RQ63	3988
B9WGQ6	573826
B9WPP9	2252
B9WW23	286604
B9XLJ6	320771
B9XW63	544405
B9XZU7	544406
B9Y2T7	545696
C0A9E2	278957
C0ATZ7	471881
C0ATZ8	471881
C0BB24	470146
C0BIH7	487796
C0BLL5	487797
C0BUC3	547043
C0BX65	553973
C0CS54	476272
C0CTM3	518636
C0E4U7	566549
C0E9U2	537013
C0ENR2	546264
C0ENR3	546264
C0ETW9	411469
C0FPZ9	622312
C0G3Z3	595497
C0M727	553482
C0MEK1	553483
C0N2Z5	637616
C0NIM4	447093
C0Q6Y4	476213
C0QGH3	177437
C0QWK1	565034
C0RGS5	546272
C0S9G7	482561
C0VV97	548477
C0W243	525245
C0W5L5	525246
C0WKF5	525260
C0X578	491074
C0XIV4	525327

C0XS81	525263
C0YUW1	525257
C1A1R9	234621
C1AVT4	632772
C1C7T1	488221
C1CEQ8	488222
C1CL37	488223
C1CR03	487213
C1D8T0	557598
C1GBZ4	502780
C1HAN5	502779
C1HLD1	469598
C1HYU7	528345
C1I8B3	457396
C1M3R4	469595
C1V8W8	469382
C1V8W9	469382
C2BNQ5	525264
C2BYG3	525367
C2CNP2	525268
C2CYM6	525310
C2D848	525256
C2DD07	525278
C2DQA1	525281
C2EDD3	525362
C2EJJ3	525364
C2FDD8	525337
C2FKR1	525338
C2FVZ9	525372
C2GKR8	548478
C2GY27	548480
C2H567	525271
C2HC73	525279
C2JJR6	491075
C2K157	525361
C2KTF9	585199
C2L0W2	585501
C2LEJ3	525369
C2LTS1	596322
C2M4R2	553178
C2M4S2	553178
C2ME01	596327
C3J8J3	553175
C3JU89	596309
C3K2C9	216595
C3KKH2	394

C3PH96	548476
C3Q3H0	457395
C3Q3H5	457395
C3QI46	556258
C3QI53	556258
C3QX18	469590
C3QX38	469590
C3RBL6	556260
C3RBM1	556260
C3RKH6	556270
C3T742	562
C3WAP6	469616
C3X4W9	556268
C3XB31	556269
C3XFD4	613026
C3XJS4	556267
C4F1M2	521005
C4F5W7	521004
C4F820	521003
C4FD46	518635
C4G6Y9	592010
C4GAE3	626523
C4GF98	629741
C4GYZ8	547047
C4HFN0	547046
C4IDT1	632245
C4IN97	641140
C4JAW3	4577
C4JW59	336963
C4LHR0	645127
C4QW46	644223
C4S086	349968
C4SC10	349967
C4SLU2	349966
C4T263	349965
C4TR58	527012
C4U520	527002
C4UN23	527005
C4UUT2	527004
C4V2L2	638302
C4VE20	553209
C4WH45	641118
C4X724	484021
C4Y7X8	306902
C4YGN3	5476
C4Z318	515620

C4Z8N8	515619
C4ZIU4	85643
C4ZZB7	595496
C5BB17	634503
C5BWF2	471853
C5C933	465515
C5D7D8	471223
C5DFW7	559295
C5DSI5	559307
C5E973	537937
C5ER19	457421
C5EXX2	537972
C5F9Y0	537973
C5FF36	554155
C5G895	559297
C5JPP3	559298
C5MCT4	294747
C5P8S8	222929
C5PTG7	525373
C5RB90	585506
C5RID0	573061
C5S0Z7	637911
C5S0Z8	637911
C5TA65	573060
C5TKE9	596320
C5UPY2	536233
C5VD14	553207
C5VK09	553174
C5VLX4	553174
C5VRL1	592027
C5VYI0	218494
C5W4T4	511693
C5WG08	486410
C5XN30	4558
C5ZXL8	537970
C6A5L0	580050
C6AG68	555970
C6AJR8	634176
C6BS98	526222
C6CXF6	324057
C6DJB5	561230
C6E8X1	443144
C6ECJ6	469008
C6EVR5	5421
C6GQT9	568813
C6GUU4	568814

C6H7Z4	544712
C6I9Z8	457392
C6IA26	457392
C6IME1	469586
C6IMJ7	469586
C6IXQ9	621372
C6J7W9	457412
C6LMB6	478749
C6M0A8	598745
C6M581	547045
C6MB43	153948
C6MNF5	443143
C6N0S9	658187
C6N5E8	658187
C6P885	580327
C6PTN9	536227
C6QID7	582899
C6RAQ4	553206
C6RHN9	553219
C6RMH6	596318
C6S8E8	662598
C6SBQ2	663926
C6SL48	295996
C6SQZ5	511691
C6UJ75	413997
C6UX17	544404
C6VNY5	644042
C6VYT1	471854
C6WZG7	531844
C6XS85	485917
C6YTX8	539329
C6Z9F0	457394
C6Z9F5	457394
C7BU49	553480
C7BXF1	592205
C7CFH7	661410
C7CT29	565636
C7D3S6	565637
C7G5J4	536231
C7GPH9	574961
C7GUL4	574961
C7GZY3	592031
C7H7A0	411483
C7HHQ3	572545
C7IDC0	588581
C7LFT0	568815

C7LQK9	525897
C7M8B3	521097
C7MAV2	446465
C7MNC8	469378
C7MZK2	471857
C7N599	471855
C7NHY3	478801
C7P570	485914
C7QXG9	395962
C7R2K2	471856
C7R731	523791
C7TA59	568703
C7TGU1	568704
C7U7K5	565638
C7UGI0	565642
C7UP10	565641
C7UV78	565650
C7V5G4	565640
C7VCG4	565644
C7VNK8	565646
C7VW65	565649
C7W2T1	565647
C7W9W7	565648
C7WKH6	565643
C7WNY5	565651
C7X246	565645
C7X6E0	563193
C7X6E5	563193
C7YAS1	565639
C7YTV6	660122
C8JVB1	393124
C8K3J9	393125
C8K7X8	393128
C8N8B6	638300
C8NPT9	196164
C8NTT8	585529
C8PF63	553220
C8PZ35	553217
C8RTU9	525262
C8T6I9	667127
C8TSS7	573235
C8UA24	585395
C8UBL5	585396
C8V8W7	227321
C8WAA2	521095
C8WI25	479437

C8Z3M7	643680
C8ZHC3	643680
C9A1D2	565653
C9ACQ0	565655
C9ADJ6	565662
C9AMB6	565663
C9B0M9	565652
C9B4P5	565658
C9BEH6	565659
C9BQJ0	565657
C9BZD6	565661
C9C7M6	565660
C9CAF1	565656
C9CNZ8	565654
C9KN37	500635
C9KV79	483215
C9KV88	483215
C9LB68	537007
C9LHG2	626522
C9LXX3	546271
C9MCJ1	656913
C9MIH4	656912
C9MRD2	649761
C9NDK4	591167
C9P7J4	675813
C9PLW8	675811
C9PN71	667128
C9PVK5	619693
C9QTU7	536056
C9R6N5	668336
C9RNS6	59374
C9T509	520459
C9TED0	520460
C9TMR2	520463
C9TT50	520461
C9U7G1	520454
C9UGN5	520452
C9UQX5	520451
C9UVW3	520450
C9V8A3	520456
C9VL93	520457
C9VVX2	520455
C9WY24	604162
C9XFX9	568708
D0ADQ1	565666
D0ALR7	565664

D0AZH6	575591
D0B4S8	224914
D0BDB8	520488
D0C0M9	575564
D0C906	575584
D0GHI0	520465
D0GUP7	675806
D0HHK9	675820
D0ITD0	290847
D0J1V2	688245
D0JK87	637382
D0JUB8	637385
D0K0F2	684950
D0KE64	561231
D0L7K3	526226
D0LGK4	502025
D0PAQ5	520489
D0PNE2	520487
D0RLL9	437701
D0RSL1	469609
D0S072	575585
D0SAL3	575586
D0SMD0	575587
D0SUU2	575588
D0T4P8	575589
D0TCW4	469589
D0TCW9	469589
D0TR30	469588
D0TR38	469588
D0W527	546262
D0W6E5	546265
D0WEM6	649764
D0WLT4	649743
D0WS85	674977
D0YPN7	596328
D0ZGL3	498217
D0ZW30	588858
D1A2H6	471852
D1AKZ0	526218
D1B531	525898
D1BEM5	446469
D1BVJ5	446471
D1C4B5	479434
D1CFL6	525904
D1CVG1	520449
D1D6W1	528346

D1DA26	528352
D1DGC3	528354
D1DQN2	528356
D1DXB4	528355
D1E3Z9	528358
D1EA59	528359
D1EGY9	528360
D1EQ98	520462
D1F1J5	520464
D1F6I8	520466
D1FHQ5	520458
D1JAA5	115547
D1JUC7	469587
D1JUF1	469587
D1K6K4	457391
D1K6L0	457391
D1NBA2	340101
D1NLN9	492476
D1NWC2	561180
D1P0C7	500637
D1PC66	537011
D1PIU8	411471
D1PUP4	585502
D1PVJ6	585502
D1Q465	545431
D1QQK2	649760
D1RYM0	682634
D1SE87	644283
D1TVF9	687916
D1VYL3	679189
D1W6L1	679190
D1WTX5	649189
D1XQ85	647653
D1XVC1	553171
D1YDA3	679194
D1ZT36	5147
D2AFF8	591020
D2ALP0	510831
D2BFC1	479432
D2C507	590168
D2EQG4	563037
D2EZH8	585543
D2EZM0	585543
D2KKK9	1307
D2KKL0	1307
D2KKL1	1307

D2LZH8	649639
D2MN04	679192
D2NJ38	431946
D2NY56	653938
D2PA11	637381
D2Q6I5	401473
D2R268	530564
D2RXF9	543526
D2RY65	543526
D2RZ42	543526
D2RZC6	543526
D2S0I1	543526
D2SGC4	526225
D2TUK6	637910
D2U087	638
D2YJ06	671074
D2YQC3	671076
D2ZB99	500639
D2ZPY7	521002
D2ZZV4	546266
D3A3C1	546268
D3AI09	566550
D3C045	653733
D3CE47	648999
D3DZ61	634498
D3EDK3	481743
D3FWN6	398511
D3GVH4	216592
D3H869	365659
D3HE25	637909
D3HXZ4	575611
D3I165	575611
D3I787	575612
D3I8X6	575612
D3IAQ1	575614
D3IEB0	575614
D3IK01	575615
D3KL75	393121
D3LG75	565665
D3LKL2	596312
D3LW10	699218
D3MGM7	679195
D3MP04	686659
D3QUV4	701177
D3R7E3	552531
D3RJ65	640131

D3SX02	547559
D3T0V9	547559
D3T144	547559
D3TA98	439481
D3UGI7	679897
D3USF6	683837
D3UY64	406818
D3VF16	406817
D4B2H4	663331
D4BAI2	500640
D4BLG5	518634
D4C241	521000
D4CDT0	411486
D4CKH1	608534
D4DLM1	663202
D4DN64	546263
D4DYU0	667129
D4ED00	694569
D4EPZ4	699185
D4ERV7	699186
D4FAH4	500638
D4FTM1	655813
D4G8V5	515618
D4GPR3	309800
D4GY63	309800
D4GY69	309800
D4H2E9	522772
D4HFC6	553199
D4INE4	717959
D4IQD3	717959
D4J2B6	657324
D4J437	717962
D4JCV2	717960
D4JI76	657317
D4JS51	657319
D4JZ94	718252
D4KBX4	657322
D4KF87	657316
D4KKW8	657315
D4L424	718255
D4L843	657321
D4LFA8	213810
D4LPM4	657323
D4LSA1	657314
D4M5L5	657313
D4MB72	657310

D4MKI4	717961
D4MXD7	245018
D4PIU4	393117
D4PR78	393131
D4Q3R2	401650
D4QHV0	529885
D4QQR5	535205
D4R228	535206
D4R6G3	544875
D4RLZ6	546340
D4RMW1	547468
D4S0B6	511680
D4S8A3	585503
D4SAK9	585531
D4SLZ9	645663
D4TWN4	649742
D4UMW4	246199
D4UN78	246199
D4UYQ9	791166
D4VC17	702446
D4VC22	702446
D4VQU5	702447
D4VQV2	702447
D4VVZ9	791161
D4WGG5	702443
D4WGH6	702443
D4X2R5	702444
D4X2S2	702444
D4X4P0	742159
D4XP09	707232
D4YH29	655812
D4YQB5	585530
D4ZR61	696747
D5AFS8	423211
D5B872	637386
D5BCK8	655815
D5BVC3	472759
D5CCV4	716541
D5D411	714962
D5DDK5	592022
D5E439	545693
D5ESC7	264731
D5EUQ2	264731
D5G4A5	39416
D5HDH9	751585
D5MHU6	671143

D5NU55	649754
D5PPW1	525370
D5QPZ9	595536
D5R8K4	642492
D5S619	760570
D5TES2	573236
D5U7Q3	526224
D5UKD3	446466
D5VAL1	749219
D6A4N4	566461
D6AI30	457431
D6B9J6	457425
D6CW74	657309
D6CW84	657309
D6DAY9	722911
D6DGX1	717608
D6DTW7	718254
D6E321	657318
D6E6Z1	657308
D6EE73	457428
D6GN49	469608
D6H6N1	528348
D6HL81	552396
D6HXP2	550672
D6IB74	550676
D6IQP0	656380
D6JA48	550677
D6JKW7	528351
D6JRN2	575565
D6KBG9	645465
D6KTP9	641146
D6L4N0	641144
D6LLF8	520448
D6UKB7	585535
D6V8X3	666684
D6VEJ3	596153
D6VPF7	559292
D6W368	559292
D6X9Z0	457429
D6XQU5	637913
D6XUQ4	439292
D6Y2I7	469371
D6Z5V7	589865
D6ZKH1	548479
D6ZS37	525381
D6ZX62	759350

D7AI85	663917
D7B798	446468
D7BM14	644284
D7BUM8	749414
D7C6S8	749414
D7CNN7	643648
D7E021	551115
D7FCS9	693745
D7GFF9	754252
D7H1D6	520453
D7IC11	469585
D7IC14	469585
D7IN59	469592
D7IN64	469592
D7IY50	585544
D7IY62	585544
D7JGE2	575590
D7JR10	656379
D7K6C0	457390
D7K6D0	457390
D7KHV1	81972
D7N3R1	641149
D7NA33	563008
O60995	5693
O60996	5693
O61083	5693
O87403	1402
O93934	40559
O96940	5833
P00369	5141
P07262	4932
P0C934	837
P14657	2725
P18819	162425
P24295	1512
P28724	5741
P28998	3076
P29051	2242
P29507	27300
P31026	1718
P39708	4932
P43793	727
P54386	1148
P54387	5341
P54388	486041
P55990	210

P78804	4896
P94316	817
P94598	818
P95544	839
P96110	2336
Q02D11	234267
Q02G89	208963
Q03M22	322159
Q04K22	373153
Q0A6S2	187272
Q0AIP4	335283
Q0BNP9	393011
Q0CL71	341663
Q0I4P4	205914
Q0JZU7	381666
Q0SGW0	101510
Q0SST9	289380
Q0T4W7	373384
Q0TH69	362663
Q0TQ84	195103
Q0USN4	13684
Q0USN5	13684
Q0VMQ0	393595
Q0VMQ1	393595
Q0W981	5127
Q0WA46	632
Q10WR8	203124
Q14J69	393115
Q15Z70	342610
Q166S7	375451
Q17YH3	382638
Q18J04	362976
Q1B412	164756
Q1C1B1	360102
Q1CDI3	377628
Q1CSJ3	357544
Q1GPE6	117207
Q1H1T3	265072
Q1IF28	384676
Q1IT55	204669
Q1JT17	383379
Q1K016	281689
Q1KYN6	222440
Q1LD03	266264
Q1N3D2	207949
Q1NJF0	262489

Q1NW86	262489
Q1Q9E1	335284
Q1QPK1	323097
Q1RB31	364106
Q1VBD1	314288
Q1VY22	313595
Q1WSM5	362948
Q24MK0	138119
Q26GG7	156586
Q2A5D8	376619
Q2G8V7	279238
Q2GWE1	38033
Q2IDY1	290397
Q2KU68	360910
Q2LC62	5551
Q2LQG2	56780
Q2NBY5	314225
Q2NEG6	339860
Q2UG98	5062
Q2Y685	323848
Q2YP74	359391
Q30QE4	326298
Q31EJ5	317025
Q321Q3	300268
Q32GA3	300267
Q39WF1	269799
Q3A571	338963
Q3D0Y7	342615
Q3D6F6	342616
Q3DFQ8	342617
Q3DJH6	342614
Q3DQR9	342613
Q3IL85	326442
Q3ILZ7	348780
Q3IS94	348780
Q3ISK4	348780
Q3JCR9	323261
Q3K6J3	205922
Q3M5M1	240292
Q3MDV8	240292
Q3XZ31	333849
Q3Z2A7	300269
Q46UV2	264198
Q47Q60	269800
Q49JG7	58096
Q4C653	165597

Q4D5C2	5693
Q4DWV8	5693
Q4EID3	267410
Q4EVW3	267409
Q4FR40	259536
Q4JV01	306537
Q4K5S2	220664
Q4KTJ5	1358
Q4PAR2	5270
Q4Q7X1	5664
Q4QP01	281310
Q4WNQ8	5085
Q4X2W2	5825
Q57FF3	235
Q57PY4	28901
Q59KP4	5476
Q5F731	242231
Q5JKR7	39947
Q5KL32	5207
Q5L9U9	272559
Q5L9X6	272559
Q5M148	299768
Q5M5N6	264199
Q5NHR7	119856
Q5P0F8	76114
Q5QVZ2	135577
Q5V3Y8	2238
Q5V4X6	2238
Q5V6I7	2238
Q5WCI5	66692
Q5X2P3	297246
Q5YQU8	37329
Q60A41	414
Q64BT4	285389
Q64CH4	286719
Q64CM6	286721
Q64Q81	817
Q65C63	230603
Q65C64	55189
Q65C65	27292
Q65C67	4931
Q65GI2	279010
Q65W57	221988
Q664F6	633
Q6A7K5	1747
Q6AF05	59736

Q6AJB1	84980
Q6ANZ7	84980
Q6BTN2	4959
Q6C1A9	4952
Q6CLT3	28985
Q6DB50	29471
Q6FD67	62977
Q6FWH3	5478
Q6KZF2	263820
Q6MPX2	959
Q6NGH0	1717
Q6SI43	257382
Q722Z0	265669
Q74DL1	35554
Q7M823	844
Q7NA25	141679
Q7NLJ0	33072
Q7RGT5	73239
Q7RQ39	73239
Q7V7V9	74547
Q7VGS9	32025
Q7VSN6	520
Q7WEU7	518
Q7XXT6	2788
Q82U80	915
Q835G2	1351
Q83RH0	623
Q84WI8	3702
Q88Q23	160488
Q88XM9	1590
Q8A6B2	818
Q8CZK9	632
Q8DPG0	171101
Q8DUL2	1309
Q8FGY8	217992
Q8G2T7	29461
Q8G6L0	216816
Q8GE72	1265
Q8ILF7	36329
Q8ILT0	36329
Q8PZY2	2209
Q8RQP4	152794
Q8TFF6	4931
Q8TL71	2214
Q8XK85	1502
Q8Y9G8	1639

Q8YF04	29459
Q8YPE2	103690
Q8ZT48	13773
Q8ZW33	13773
Q92E91	1642
Q930S3	382
Q96UJ9	76867
Q96VJ7	5127
Q977U6	2252
Q97AN9	273116
Q97L29	1488
Q97QB4	1313
Q9AGH6	28451
Q9AIW1	1307
Q9C8I0	3702
Q9CPJ4	747
Q9GTK5	5833
Q9HFR6	42251
Q9HGS2	4959
Q9HGU3	40126
Q9HGU4	5142
Q9HK32	2303
Q9HQE1	2242
Q9HRM7	2242
Q9HSM4	2242
Q9HVJ7	287
Q9K580	573
Q9KB34	86665
Q9L0F9	1902
Q9RTN9	1299
Q9S1F9	81861
Q9TVN3	47911
Q9TXS8	5833
Q9URS1	5076
Q9Y8I4	2277
Q9Z3C4	287
Q9ZKD8	85963
